# An extensive common‐garden study with domesticated and wild Atlantic salmon in the wild reveals impact on smolt production and shifts in fitness traits

**DOI:** 10.1111/eva.12777

**Published:** 2019-03-06

**Authors:** Øystein Skaala, Francois Besnier, Reidar Borgstrøm, BjørnTorgeir Barlaup, Anne Grete Sørvik, Eirik Normann, Britt Iren Østebø, Michael Møller Hansen, Kevin Alan Glover

**Affiliations:** ^1^ Institute of Marine Research Nordnes, Bergen Norway; ^2^ Faculty of Environmental Sciences and Natural Resource Management Ås Norway; ^3^ NORCE Environment, LFI Bergen Norway; ^4^ Department of Bioscience Aarhus University Aarhus C Denmark; ^5^ Department of Biological Sciences University of Bergen Bergen Norway

**Keywords:** aquaculture, competition, fitness, genetic, hybridization, introgression, salmon

## Abstract

Interactions between domesticated escapees and wild conspecifics represent a threat to the genetic integrity and fitness of native populations. For Atlantic salmon, the recurrent presence of large numbers of domesticated escapees in the wild makes it necessary to better understand their impacts on native populations. We planted 254,400 eggs from 75 families of domesticated, F1‐hybrid, and wild salmon in a river containing up‐ and downstream traps. Additionally, 41,630 hatchery smolts of the same pedigrees were released into the river. Over 8 years, 6,669 out‐migrating smolts and 356 returning adults were recaptured and identified to their families of origin with DNA. In comparison with wild salmon, domesticated fish had substantially lower egg to smolt survival (1.8% vs. 3.8% across cohorts), they migrated earlier in the year (11.8 days earlier across years), but they only displayed marginally larger smolt sizes and marginally lower smolt ages. Upon return to freshwater, domesticated salmon were substantially larger at age than wild salmon (2.4 vs. 2.0, 4.8 vs. 3.2, and 8.5 vs. 5.6 kg across sexes for 1, 2, and 3 sea‐winter fish) and displayed substantially lower released smolt to adult survival (0.41% vs. 0.94% across releases). Overall, egg‐to‐returning adult survival ratios were 1:0.76:0.30 and 1:0.44:0.21 for wild:F1‐hybrid:domesticated salmon, respectively, using two different types of data. This study represents the most updated and extensive analysis of domesticated, hybrid, and wild salmon in the wild and provides the first documentation of a clear genetic difference in the timing of smolt migration—an adaptive trait presumed to be linked with optimal timing of entry to seawater. We conclude that spawning and hybridization of domesticated escapees can lead to (i) reduced wild smolt output and therefore wild adult abundance, through resource competition in freshwater, (ii) reduced total adult abundance due to freshwater competition and reduced marine survival of domesticated salmon, and (iii) maladaptive changes in phenotypic traits.

## 1 INTRODUCTION

Atlantic salmon (*Salmo salar*) has been the subject of research for over a century, generating knowledge of its extensive biological and life‐history variation within and among populations. During the freshwater stage of its primarily anadromous life cycle, it inhabits a wide range of rivers from temperate to arctic regions, and during the marine stage, it migrates to offshore oceanic areas. A combination of natal homing and associated low straying rates (Jonsson, Jonsson, & Hansen, 2003; Stabell, 1984) has permitted genetic differentiation to emerge among populations throughout its native range (Bourret et al., 2013; King, Kalinowski, Schill, Spidle, & Lubinski, 2001; Ståhl, 1987; Verspoor et al., 2005). In addition, wild salmon populations have been shaped by natural selection, through competition for resources like food, space, and mates, and synchrony to the contrasting environments in which they live. Consistent with this is the accumulating evidence that at least part of the observed phenotypic differentiation among salmon populations reflects adaptations to local environments (Fraser, Weir, Bernatchez, Hansen, & Taylor, 2011; Garcia de Leaniz et al., 2007; Taylor, 1991). Due to the above attributes, Atlantic salmon serves as an ideal species in which to study evolutionary processes including selection and adaptation at the individual and population levels.

Each year, thousands or hundreds of thousands of domesticated Atlantic salmon escape from aquaculture facilities into the wild and thereafter enter rivers (Diserud, 2018; Glover, 2018; Morris et al., 2008). Consequently, genetic interactions between domesticated escapees and wild conspecifics represent one of the major challenges to environmentally sustainable aquaculture (Forseth et al., 2017; Glover et al., 2017; Taranger et al., 2015). At present, hundreds of animal and plant species, including 362 finfishes, are being domesticated around the world (FAO, 2016). Of the finfish species farmed for food, Atlantic salmon is among those that have been subject to the longest and most intense domestication regimes (Gjedrem, 2010; Teletchea & Fontaine, 2014). As a result of nearly 50 years of directional selection regimes for traits of economic importance, inadvertent domestication selection, and relaxed natural selection, domesticated salmon now display a wide variety of genetic differences to wild conspecifics (Glover et al., 2017). Of these differences, the documented lower survival of domesticated salmon offspring in the natural environment (Fleming et al., 2000; McGinnity et al., 2003; Skaala et al., 2012) provides some of the most compelling evidence that introgression of domesticated escapees in native populations is likely to lead to negative consequences. Making this more pertinent is the fact that genetic changes in wild populations, caused by spawning of domesticated escapees, have been documented in a number of rivers and regions (Bourret, O'Reilly, Carr, Berg, & Bernatchez, 2011; Clifford, McGinnity, & Ferguson, 1998; Crozier, 1993; Glover et al., 2012; Skaala, Wennevik, & Glover, 2006; Verspoor, Knox, & Marshall, 2016), and, that domestication–admixture has been detected in a large and increasing number of wild populations (Glover et al., 2013; Karlsson, Diserud, Fiske, & Hindar, 2016; Keyser et al., 2018). Consequently, there are widespread concerns over the evolutionary trajectory and persistence of wild salmonid populations faced with spawning intrusion from domesticated escapees (Ferguson et al., 2007; Glover et al., 2017; Hindar, Ryman, & Utter, 1991; Naylor et al., 2005). For other fish species being domesticated, these concerns also exist, but the background data are more scarce and as such Atlantic salmon is regarded as the model species of domesticated and wild fish interactions (Bekkevold, Hansen, & Nielsen, 2006).

Investigations of fitness in the natural environment are logistically demanding, and permissions may be difficult to obtain due to ethical and political reasons. Such studies are therefore scarce. At the present, only five domesticated–wild studies have been conducted for Atlantic salmon in the wild. The first one was conducted by releasing and following families of domesticated, hybrid, and wild salmon in the Burrishoole River in Ireland in a “common‐garden” type experiment (McGinnityet al., 2003, 1997; Reed et al., 2015). Two generations of results demonstrated reduced performance of all domestic‐influenced offspring groups compared with wild salmon, and a “lifetime” (eggs planted to returning adult) success of only 2% of domesticated offspring compared with wild salmon. In the second of the studies, conducted by permitting and following upstream migration, spawning and survival of domesticated escapees entering the River Imsa in Norway, Fleming et al. (2000) found that gene flow from escapees to the wild population was sex biased and predominantly via wild males and domesticated females. They also reported a “lifetime” (escapees in river to returning adults in the following cohort) success of domesticated offspring of 16% compared with wild salmon. In the third study, consisting of an extensive release of domesticated, hybrid, and wild eggs from 69 family groups in the Norwegian River Guddalselva, Skaala et al. (2012) reported large among‐family variation in egg to smolt survival of 0.17%–6.2%. However, also here, the offspring from domesticated parents had on average a significantly lower survival compared to that of wild offspring. In the fourth study (Jonsson & Jonsson, 2017), releases of hatchery‐produced domesticated, hybrid, and wild salmon smolts from the River Imsa revealed lower marine survival, increased straying rates, and generally larger size at age in comparison with wild salmon. In addition to these experimental studies, Bolstad et al. (2017) investigated life‐history differences between naturally recruited wild and domestication‐admixed salmon in 62 Norwegian salmon populations. Their approach unveiled differences between admixed and wild salmon in both size at adult age as well as age at maturity, although this varied between population types and phylogenetic regions.

The studies highlighted above have provided a major contribution to our understanding of the genetic differences between domesticated and wild fish in the natural environment. However, several aspects of the genetic differences between domesticated and wild salmon in the natural environment are still poorly studied, and some of the data from the above studies are outdated. For example, two of the above studies (Fleming et al., 2000; McGinnityet al., 2003, 1997) were conducted nearly three decades ago which means that domesticated salmon have been selected through 5–7 generations more since those pioneering investigations. Also, in the case of the work previously conducted in the River Guddalselva, a local wild population was not available and a proxy was used from the living gene bank which may have influenced the observed differences between domesticated and wild salmon (Skaala et al., 2012).

Investigations of fitness differences between domesticated and wild salmon in the natural environment are not only important to quantify the type and magnitude of differences; they provide data to parameterize quantitative models projecting the long‐term consequences of admixture (Castellaniet al., 2015, 2018). Therefore, building upon the limitations of previous studies, we conducted the most up to date and detailed common‐garden study of domesticated, F1‐hybrid, and wild salmon in the natural environment thus far. This included the release of large numbers of pedigree‐controlled eggs and hatchery‐produced smolts from multiple cohorts and families into the River Guddalselva. Over eight years, DNA parentage testing was successfully used to resolve the pedigree of out‐migrating smolts resulting from the egg plantings, and adult recaptures originating from the naturally migrating smolts and the hatchery‐reared smolts released into the river. The overall aim of the study was to provide extensive up to date data on the type and magnitude of genetic differences in a range of key fitness‐related traits between domesticated and wild salmon in the natural environment—including both the freshwater and the poorly studied thus far marine stage.

## 2 MATERIALS AND METHODS

### 2.1 The experimental river

**Figure 1:**
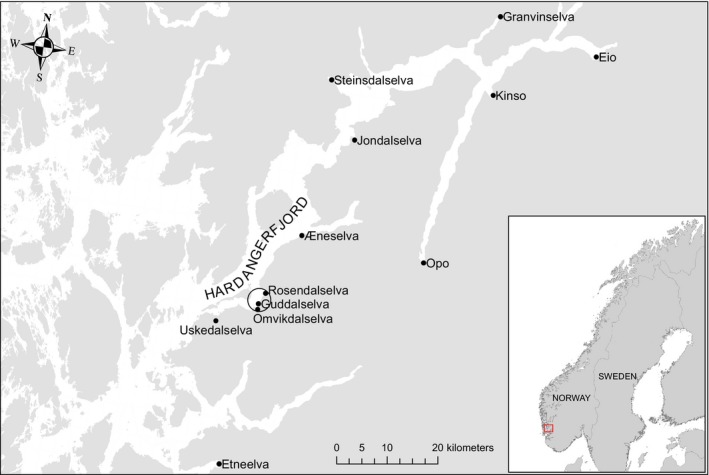
Map of the Hardangerfjord and location of the major rivers with Atlantic salmon ascendance

The experiment was conducted in the River Guddalselva which flows into the central part of the Hardangerfjord, Western Norway (Figure 1). A full overview of the river system, including photographs of the traps and stretches of river, is available (Supporting Information, Figure S1). The river has a small population of anadromous trout, *Salmo trutta, *with annual catch reports varying from 0 to 200 fish (average 26) in the period 1969 to 2008 and from 0 to 26 (average 6) in the period 1998 to 2008 (https://www.ssb.no/statbank/list/elvefiske). The annual catch reports for Atlantic salmon in the river varied from 0 to 200 fish (average 14) between 1969 and 2008 and from 1 to 22 (average 8) between 1998 and 2008. The number of naturally recruited salmon smolts captured in the trap from 2001 to 2005 varied from 32 to 241 (average 145). The catch reports and smolt counts therefore indicate the absence of a local and self‐sustaining salmon population.

The river has a permanently installed Wolf trap that captures most of the descending smolts, and two fish ladders with traps that capture all ascending Atlantic salmon and sea trout. The river has a 37 km^2^ catchment area and a mean water discharge ~4 m^3^/s (typically ranging 0.5–16 m^3^/s). The lower section of ~2 km is accessible for anadromous fish up to a waterfall (Liarefossen) that is impassable for anadromous fish. Above the waterfall, there is a further ~2 km (about 1.5 ha) of habitat inhabited by resident brown trout.

### 2.2 Experimental strains and comparison of performance in the freshwater phase

Three cohorts of domesticated, hybrid, and wild Atlantic salmon families were established in the river by planting eyed eggs in late winter, with hatching in spring 2008, 2010, and 2011 (hereafter referred to as C2008, C2010, and C2011). Because the River Guddalselva has no native salmon population, wild salmon from the nearby river Etne, the largest salmon population in the Hardangerfjord, was used as a “wild‐type” substitute (Figure 1). Eggs and milt from the domesticated Mowi salmon strain were provided by Marine harvest. This domesticated strain has been subject to more than 12 generations of domestication and directional selection for a variety of traits of importance in aquaculture. Further details of this strain, including the degree of genetic differences it displays relative to wild salmon populations, can be gained from multiple sources (Bicskei, Bron, Glover, & Taggart, 2014; Glover et al., 2009; Harvey, Glover, Taylor, Creer, & Carvalho, 2016; Skaala et al., 2012; Solberg, Glover, Nilsen, & Skaala, 2013; Solberg, Zhang, Nilsen, & Glover, 2013).

Fertilized eggs of the three cohorts were established in 2007 (19–20 November), 2009 (17 November), and 2010 (16 November) and incubated in single‐family incubators in the hatchery at the research station of IMR at Matre, until they reached the eyed stage. The families produced were a combination of full‐ (domesticated and wild) and half‐sibs. Due to logistical constraints, F1‐hybrids (hereon referred to as hybrids for simplicity) only using a domesticated mother and a wild father were produced. The size ranges of wild parental fish were 1.4–12.0 kg (males) and 3.2–10 kg (females), while the domesticated fish were in the size range 10–18 kg after 2 years in sea cages.

Egg weight and size were measured before they were counted, randomized, and thereafter planted into the river bed in the upper reaches of the river in Whitlock‐Vibert boxes (500 eggs/box). A total of 254,400 eyed eggs from 75 families (Supporting information Table S1) were planted, with 69,800 eggs in 2008 (C2008), 106,000 eggs in 2010 (C2010), and 78,600 eggs in 2011 (C2011), giving densities of 4.7, 7.1, and 5.2 eggs per m^2^ for the three cohorts, respectively.

### 2.3 Comparison of performance in the marine phase

Two complimentary experimental approaches were used to investigate marine survival and growth: (a) Returning anadromous spawners that originated from the eggs planted into the river (marine survival seen in relation to the recorded family/type smolt output), and (b) returning anadromous spawners originating from hatchery‐produced 1‐year smolts released in the outlet of the River Guddalselva in spring 2011 and 2012. For the latter experimental approach, the genetic material was identical to that used in the egg plantings in 2010 and 2011, respectively. These hatchery smolts were produced at Matre under standard rearing conditions and natural light regimes. In spring 2011, 17,630 smolts were transferred to a holding cage at the outlet of the river where they were held for 2 days before release, while in spring 2012, 24,000 smolts were released directly in the Sahølen pool in Guddalselva below the ascendance traps. All smolts were coded wire tagged (CWT) and adipose fin clipped. For all adult salmon returning to the River Guddalselva, the age of returning spawners was determined, and smolt lengths back‐calculated, by reading fish scales using the methodology described by Lea‐Dahl (Dahl, 1910; Lea, 1910).

### 2.4 Sampling migrating smolts and returning adults

Depending on water discharge, the Wolf smolt trap was mounted each spring around 1 April. The trap was monitored daily during the whole smolt run and a further few weeks, usually to the end of June. Each smolt was sedated before length and weight were recorded, and the adipose fin was removed. Thereafter, smolts were transferred to a recovery tank for some hours before being released back into the river to continue their seaward migration.

The two upstream traps were operated from before the beginning of ascendance of fish from the fjord, usually in the middle of May, and to the middle of November. Captured fish were sedated before length and weight were recorded and inspected for external and internal tags. All tagged fish resulting from our experiment were killed in order to avoid spawning by offspring from domesticated or hybrid ancestry in the river. This was done in agreement with the management authorities and a condition for the research permit for the experiments. In order to detect returning experimental fish which strayed to other rivers in the region, the occurrence of adipose clipped fish was recorded during the annual spawning counts conducted by snorkeling the rivers in this region. Detected fish were captured and killed, and scale and tissue samples and other biological information were delivered to IMR.

### 2.5 Family assignment

All recaptured fish (i.e., smolts and adults) were identified to their families and thus genetic groups of origin using DNA parentage testing. This was conducted through the analysis of six highly polymorphic microsatellites at the molecular genetics laboratory of the IMR and thereafter an exclusion‐based approach for identification in the program FAP (Taggart, 2007). These markers have been used extensively for pedigree reconstruction of common‐garden experiments in this laboratory where full genotyping details are provided (Solberg, Dyrhovden, Matre, & Glover, 2016; Solberg, Glover et al., 2013; Solberg, Zhang et al., 2013). These genetic markers are routinely used at IMR in association with a genotyping service to identify the farm of origin for domesticated salmon escapees in Norway (Glover, 2010; Glover, Skilbrei, & Skaala, 2008), with documented low genotyping error rates (Glover et al., 2010).

### 2.6 Statistical analyses

All data analyses were performed in R (R Development Core Team, 2014), and summary statistics in the text and tables are given in the format: mean ± *SD*. For normally distributed variables such as length and weight, linear models were used to predict the response variable from covariates such as fish type (domesticated/hybrid/wild) or cohort (C2008, C2010, C2011). For the survival/mortality data, a binominal GLM was used to predict the survival rate in each family as a response to cohort, fish type, and egg size. Significance of covariates was tested with the ANOVA as implemented in R.

The difference in survival between half‐sib offspring was also tested. For this purpose, it was assumed that under the null hypothesis, half‐sibs from a wild male had the same probability to survive as half‐sibs from the same female and a domesticated male. Therefore, the number of families where the wild half‐sib have better survival should follow a binomial distribution with probability *p* = 0.5. *p* value for the test was directly obtained from the binominal distribution (pbinom function) in R.

### 2.7 Permits

The study was conducted in agreement with the Hordaland County Governor, the Norwegian Environment Agency and Norwegian Food Safety Authority with permits to plant out eggs, in addition to capture, tag, and release smolt.

## 3 RESULTS

### 3.1 Survival from eyed egg to smolt

**Figure 2:**
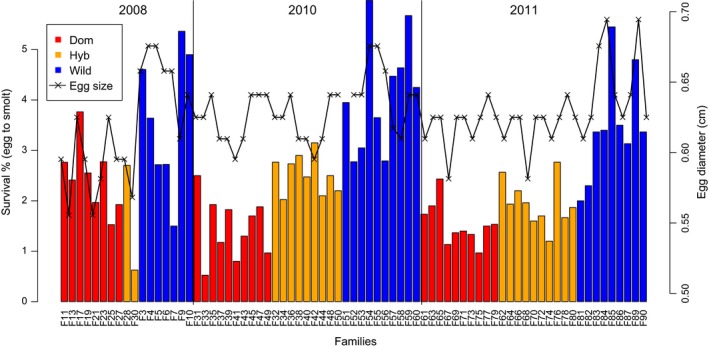
Survival from eyed egg to smolt and egg diameter by cohort in 75 family groups (domesticated, hybrid, and wild), in the River Guddalselva

**Figure 3:**
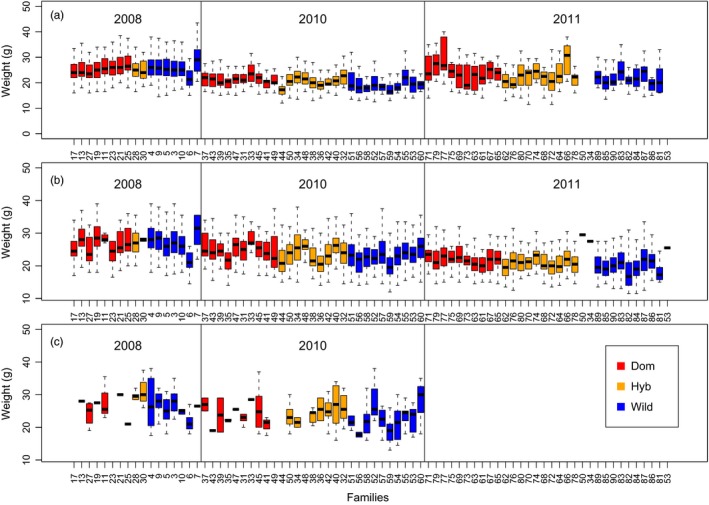
Mean smolt weight per family, ordered by type, cohort, and age. (a) 3 winter smolt, (b) 4 winter smolt, (c) 5 winter smolt of the cohorts C2008, C2010, and C2011 in the River Guddalselva

In the period 2011–2015, a total of 6,669 smolts were captured in the Wolf trap and identified to family, giving an overall survival from egg to smolt of 2.6%. Overall survival was 2.8%, 2.7%, and 2.2% for the three cohorts, respectively (*df *= 2, χ^2^ = 112, *p* < 2.2^−16^). All families produced one or more smolts, with survival varying greatly among types (*df* = 2, χ^2^ = 806, *p* < 2.2^−16^) (Figures 2 and 3, Table 1; Supporting information Table S2). Domesticated family survival ranged from 0.5% to 3.7% (average 1.8%), hybrid family survival ranged from 0.6% to 3.1% (average 2.2%), and wild family survival ranged from 1.5% to 6.0% (average 3.8%).

**Table 1:** Numbers and weights (g) of domesticated (Dom), hybrid (Hyb), and wild (Wil) smolts recaptured in the Wolf trap in the River Guddalselva resulting from the planting of 254,400 eyed eggs from 75 families in the three cohorts C2008, C2010, and C2011, and survival (S) from egg planting to smolt migration

Cohort	Type	2 Winters		3 Winters		4 Winters		5 Winters		Total		Eggs	% Survival
		*n*	W ± *SD*	*n*	W ± *SD*	*n*	W ± *SD*	*n*	W ± *SD*	*n*	W ± *SD*		
2008	D	na	na	587	25.7 ± 4.7	104	27.3 ± 5.5	11	26.1 ± 4.5	702	26.0 ± 4.9	28,600	2.4
2008	H	na	na	137	25.5 ± 4.7	24	27.3 ± 4.1	10	30.3 ± 3.4	171	25.9 ± 4.7	9,400	1.8
2008	W	na	na	694	26.4 ± 5.6	473	27.2 ± 5.2	65	26.5 ± 4.8	1,232	26.7 ± 5.4	31,800	3.8
2010	D	5	25.1 ± 5.8	248	21.4 ± 3.4	299	24.7 ± 4.6	26	24.0 ± 5.4	578	23.3 ± 4.5	39,200	1.5
2010	H	10	23.6 ± 6.1	254	20.5 ± 3.9	502	23.7 ± 4.6	41	24.8 ± 4.4	807	22.7 ± 4.6	32,000	2.5
2010	W	11	21.9 ± 4.2	284	19.3 ± 4.1	1,077	23.1 ± 4.6	100	21.9 ± 4.9	1,472	22.3 ± 4.7	34,800	4.2
2011	D	16	20.8 ± 3.4	108	24.3 ± 6.0	335	22.1 ± 3.9	na	na	459	22.5 ± 4.5	30,000	1.5
2011	H	14	19.9 ± 3.4	170	22.4 ± 5.5	393	21.1 ± 3.6	na	na	577	21.4 ± 4.3	29,600	1.9
2011	W	7	18.2 ± 3.4	162	21.9 ± 4.2	502	20.4 ± 3.9	na	na	671	20.7 ± 4.1	19,000	3.5

Note

Winters indicate smolt age.

Mean domesticated family survival varied from 2.4% to 1.5%, and 1.5% over the three cohorts, while mean wild family survival varied from 3.8% to 4.2%, and 3.5% over the same period. Thus, the wild:domesticated survival ratio was 1:0.63, 1:0.35, and 1:0.43 across the three respective cohorts (Table 1).

**Figure 4:**
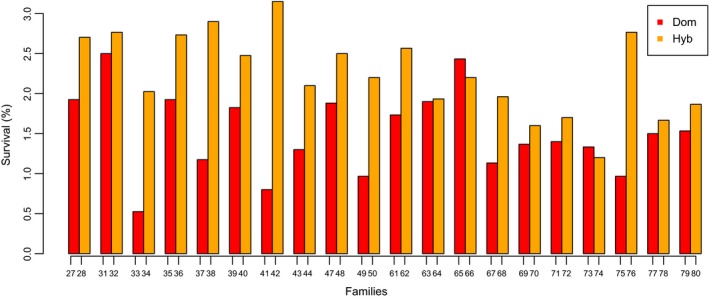
Survival from eyed egg to smolt in 20 half‐sib group comparisons in the River Guddalselva. Each group of two bars represents the survival of two families originating from the same domesticated mother

Smolt type and egg size within type had a strong effect on survival, with offspring of wild parents (*df* = 2, χ^2^ = 806, *p* < 2.2^−16^) and larger eggs (*df* = 1, χ^2^ = 5.7, *p* = 0.016) surviving better. In 18 of the 20 half‐sib comparisons with eggs from domesticated females, hybrid families sired by wild males had significantly higher survival than full domesticated families sired by domesticated males (*p* = 2.4^−4^; Figure 4). Freshwater survival of domesticated families ranged from 0.5% to 2.5% (average 1.5%), while survival of their half‐sib hybrid families ranged from 1.3% to 3.3% (average 2.3%).

### 3.2 Smolt weight

Smolt weight varied significantly among cohorts (*df* = 2, *f* = 547, *p* < 2.2^−16^), with bigger smolts arising from C2008, and smaller from C2010 (Figure 3). Smolt weight was also significantly influenced by type (*df* = 2, *f* = 39, *p* < 2.2^−16^), with average weights varying from 24.2 ± 4.9 g for the domesticated fish, 23.7 ± 5.5 g for the wild fish, and 22.8 ± 4.8 g for the hybrids.

### 3.3 Timing of smolt migration

Smolt age ranged from two to five years (Table 1) and varied among cohorts (*df* = 2, *f* = 306, *p* < 2.2^−16^). Most families in C2008 migrated at the age of 3 years, while most smolts in C2010 and C2011 migrated at 4 years of age (Table 1; Supporting information Table S2). Smolt age was also influenced by type (*df* = 2, *f* = 139, *p* < 2.2^−16^), with domesticated, hybrid, and wild smolts displaying means across cohorts of 1,429, 1,501, and 1,532 days from fertilization to smoltification.

**Figure 5:**
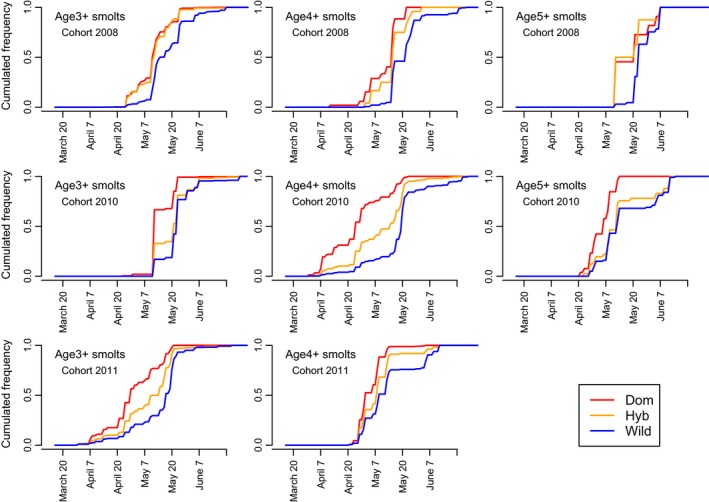
Cumulative smolt migration from the River Guddalselva of the C2008, C2010, and C2011 cohorts in regard to smolt age and type (domesticated, hybrid, wild)

The onset of the smolt migration varied among the years, from 31 March to 24 April, and lasted to 11–26 June (Figure 5). Date of migration (within year) was influenced by type, smolt weight and age, and cohort. Domesticated fish migrated earliest, with hybrid and wild smolts on average 5.5 and 11.8 days later respectively across all three cohorts (hybrid to domesticated *df* = 1, *f* = 99, *p* < 2^−16^; wild to domesticated *df* = 1, *f* = 999, *p* < 2^−16^). The difference in date of migration among types varied among years. For example, the difference in migration time between wild and domesticated smolts was approximately three weeks in 2014, both for 3‐ (originating C2011) and 4‐year‐old smolts (originating C2010; Figure 5).

**Figure 6:**
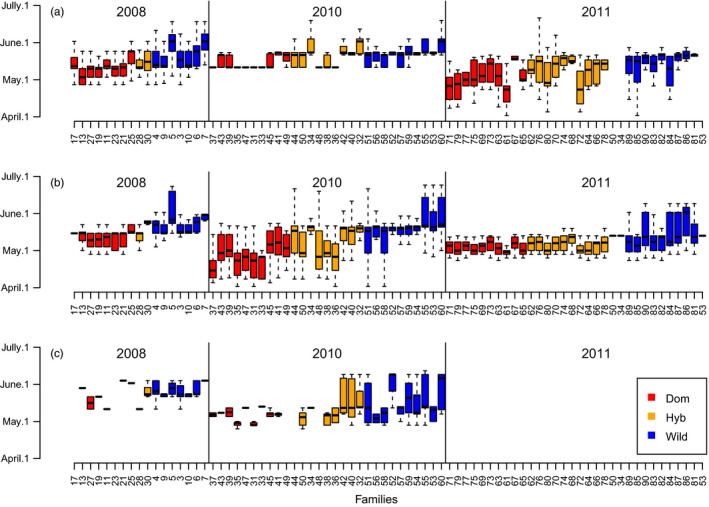
Smolt migration date in the River Guddalselva for each family ordered by type (domesticated, hybrid, wild), cohort (C2008, C2010, C2011), and smolt age. (a) 3 winter smolt, (b) 4 winter smolt, (c) 5 winter smolt

On average, larger smolts migrated later (~0.4 days delay per gram, *df* = 1, *f* = 141, *p* < 2.0^−16^), and older smolts migrated earlier (~2.7 days earlier for each winter spent in the river *df* = 1, *f* = 122, *p* < 2^−16^; Figure 3, 6; Supporting information Figure S1). However, for all age classes of smolt, the relationship between weight and date of migration varied among types, with the steepest slope for wild fish and almost no slope for domesticated fish (Supporting information Figure S1). This difference in slope between types was tested by including an interaction effect between type and fish weight in the linear model that predicts migration day as a response to type, weight, and cohort (*df *= 2, *f* = 15, *p* = 1.9^−7^).

### 3.4 Marine survival and growth of smolts arising from eggs planted into the river

Of the 6,669 smolts migrating out of the river in 2011–2015, originating from planted eggs, a total of 73 adult salmon were recaptured in the River Guddalselva in the period 2013 to 2017 (1.1% survival across all years), while one adult domesticated fish and one adult wild fish were recaptured in neighboring rivers (Table 2). The recaptures represented 1, 2, and 3 SW salmon from the 2011 to 2014 smolt runs and 1 and 2 SW salmon from the 2015 smolt run (thus excluding ≥3 SW salmon from the 2015 smolt run, and fish >3 SW from the 2014 smolt run). The observed marine survival rates for domesticated (0.75%), hybrid (1.54%), and wild salmon (1.13%) were not significantly different (*df *= 2, χ^2^ = 4.05, *p* = 0.13), and there was also no significant difference in survival between males and females (*df *= 1, χ^2^ = 1.3, *p* = 0.24).

**Table 2:** Weight (g) at recapture and recapture rate of domesticated, hybrid, and wild types from release of hatchery (H) produced smolt in 2011 and 2012 (a)^a^ and from naturally (Nat) migrating smolts (b) from the River Guddalselva

(a)										
Type	Smolt *n*	1SW (*n*)		2SW (*n*)		3SW (*n*)		Tot *n* a	Recapture %	
		Males	Females	Males	Females	Males	Females		Smolt 2011	Smolt 2012
Dom 2011 + 2012 H	6,576 + 8,000	1,952 (21)	2,753 (3)	4,783 (12)	4,838 (8)	8,250 (6)	8,500 (8)	8 + 52	0.12	0.65
Hyb 2011 + 2012 H	7,576 + 8,000	1,407 (37)	(0)	3,972 (26)	3,956 (24)	7,367 (6)	7,215 (20)	8 + 105	0.11	1.31
Wild 2011 + 2012 H	3,478 + 8,000	1,717 (22)	2,333 (3)	3,005 (22)	3,368 (19)	5,140 (7)	6,122 (29)	15 + 93	0.44	1.16

(b)				
Type	Smolt n	Mean weight at recapture (g)	Tot n	Recapture %
Dom Nat	1,739	4,920 ± 2,770	13	0.75
Hyb Nat	1,555	3,740 ± 1,811	24	1.54
Wild Nat	3,375	3,850 ± 2,040	38	1.13

a Seven individuals with incomplete age data were removed.

Adult recaptured females were larger than males (4,620 ± 1790 g Vs. 2,940 ± 2,290 g, *df *= 1, *f* = 12, *p* = 0.0008), and domesticated fish were larger than hybrid and wild fish (4,920 ± 2,770 g vs. 3,740 ± 1811 g vs. 3,850 ± 2040 g, respectively, *df *= 2, *f* = 3.5, *p* = 0.03). However, no difference in adult age was detected among types (*df *= 1, *f* = 0.07, *p* = 0.78). Cohort displayed a significant influence on adult weight (*df *= 2, *f* = 4.3, *p* = 0.01), with larger fish arising from C2011 (2,950 ± 1,490 g C2008, 3,740 ± 1,185 g C2010, 5,010 ± 2,600 g C2011). Cohort also displayed a significant influence on overall marine survival (*df *= 2, χ^2^ = 22, *p* = 0.00001), being by far lowest from C2008 (Table 2).

### 3.5 Marine survival and growth of hatchery‐produced smolts released into the river

The mean weights of the hatchery‐produced smolts upon release were 89, 50, and 36 g (2011) and 98, 85, and 49 g (2012) for domesticated, hybrid, and wild salmon, respectively. Overall survival of released smolts was lower than for the naturally produced smolts, with just 281 recaptures (0.68%) from 41,630 smolts released in total (Table 2). The marine survival for the two releases was however very different, with only 31 (0.18%) recaptures from the 2011 release, and 250 (1.04%) from the 2012 release.

For the 2011 release, the survival of wild fish (0.44%) was significantly higher than for domesticated (0.12%) and hybrids (0.11%; *df *= 2, χ^2^ = 12.8, *p* = 1.5^−3^), but hybrids and domesticated fish were not different (Table 2). For the 2012 release, both wild (1.16%) (*df *= 1, χ^2^ = 12, *p* = 5.7^−4^) and hybrid (1.31%; *df *= 1, χ^2^ = 18, *p* = 1.7^−5^) types displayed higher survival than the domesticated type (0.65%) (Table 2), while the difference in survival between hybrid and wild types was not significant (*df *= 1, χ^2^ = 0.7, *p* = 0.39).

The majority of the returning spawners were captured at the release site in the River Guddalselva (77%) and in the neighboring rivers (18%) which are located within a 3 km radius. Approximately 5% of the recaptured adults had strayed to other rivers in the Hardangerfjord basin.

**Figure 7:**
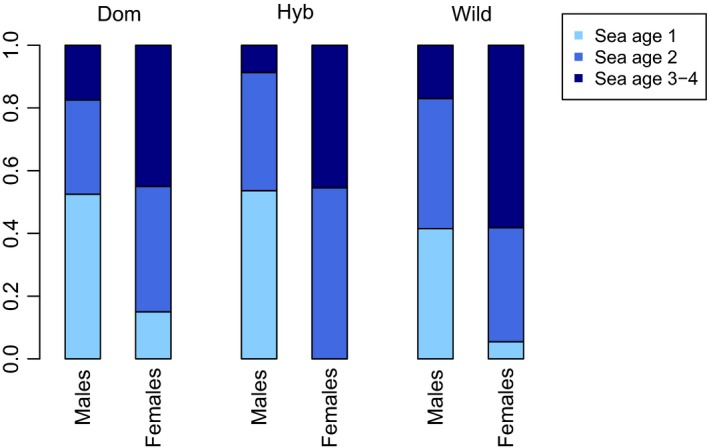
Fraction of males and females returning to the River Guddalselva as 1SW, 2SW, and 3&4SW in domesticated, hybrid, and wild types after release as smolt in spring 2011 and 2012

Males returned to spawn on average at a lower sea age 1.7 (±*SD *= 0.8) than females 2.5 (±*SD *= 0.7; *df* = 1, *f* = 63, *p* = 1.1^−13^), and among the 1SW recaptures, males dominated. Among the 2SW fish, the sex ratio of recaptures was approximately 50/50 in all three types, while among older spawners, females dominated in all three types, but most pronounced in wild and hybrid types (Figure 7; Table 2). When sex was accounted for, comparisons showed significant differences among types (*df *= 2, *f* = 3.3, *p* = 0.039), with wild fish staying longer at sea than hybrids and domesticated fish.

Both sex (*df *= 1, *f* = 68, *p* = 8.3^−15^) and type (*df *= 2, *f* = 4.6, *p* = 0.010) had influenced weight at age upon return to freshwater. Females were on average larger (5,469 g ± 2,152 g) than males (3,098 g ± 2,297 g), and domesticated fish were heavier than hybrids and wild types (Table 2). For both sexes, and all three age groups, domesticated fish were significantly larger than wild fish. In most comparisons, hybrids displayed intermediate size at age.

### 3.6 Planted egg‐to‐returning adult survival ratios

We computed the planted egg‐to‐returning adult survival ratios among the three genetic groups by (a) multiplying the overall freshwater survival across the three egg‐release cohorts with the overall marine survival of the naturally recruited smolts and by (b) multiplying the overall freshwater survival across the three egg‐release cohorts with the overall marine survival of the two hatchery‐released smolt cohorts. This gave the following planted egg‐to‐returning adult survival ratios for approach (a) wild = 1, hybrid = 0.76, and domesticated = 0.30, and for approach (b) wild = 1, hybrid = 0.44, and domesticated 0.21.

## 4 DISCUSSION

This study represents the most detailed and up to date comparison of domesticated, F1‐hybrid, and wild Atlantic salmon in the natural environment. The results demonstrate that spawning and hybridization of domesticated salmon in native populations can (a) reduce the production of wild salmon smolts and therefore wild adult abundance, through resource competition in freshwater, (b) reduce the total adult abundance through a combination of resource competition in freshwater and reduced marine survival of domesticated salmon, and (c) influence the recipient population's phenotypic (size, growth, and age of maturation) and phenological (date of smolt migration) characteristics. Therefore, when taken together with data from the low number of studies previously conducted in the natural environment, we demonstrate that domestication introgression and hybridization may lead to less productive and more fragile wild populations.

### 4.1 Freshwater survival

The significantly higher survival from egg to smolt of wild fish compared to domesticated fish is qualitatively consistent with earlier studies, albeit with different survival ratios (Fleming et al., 2000; McGinnityet al., 2003, 1997; Skaala et al., 2012). Variation in the genetic material used (e.g., degree of local adaptation and state of domestication), numbers and density of eggs planted, and the prevailing environmental and physical conditions of the rivers in which these studies were conducted, will have contributed to this variation among studies. Nevertheless, they unequivocally demonstrate that the offspring of domesticated salmon, and their hybrids, display reduced freshwater survival in comparison with wild salmon.

A greater difference in survival between domesticated and wild fish was observed in the present study compared to the previous study conducted in the River Guddalselva (Skaala et al., 2012). We suggest that there are primarily two interlinked reasons for this. First, as the River Guddalselva did not support a wild salmon population at the time of both studies, broodfish from a wild population originating from another fjord system that had been held for ~1–2 generations in the Norwegian Gene Bank was used as a proxy for wild salmon in the first study. Thus, the wild fish group used was not a locally adapted population as may be the case for many wild populations (Fraser et al., 2011; Garcia de Leaniz et al., 2007; Taylor, 1991). Furthermore, although the Norwegian Gene Bank does not practice directional selection, it is likely that a degree of inadvertent domestication had occurred in the wild salmon proxy used in the first study, which would likely display a lower fitness in the wild (Araki, Berejikian, Ford, & Blouin, 2008; Araki, Cooper, & Blouin, 2007). In the present study, we used wild brood fish collected directly from the nearby river Etne. Consequently, nonlocal and inadvertent domestication mechanisms may have contributed to the observed differences in relative survival between domesticated and wild salmon between these studies. Second, the domesticated salmon eggs planted in the first study were slightly larger than the wild salmon eggs, while in the second study, this situation was reversed. Egg size is a plastic trait, but in general, smaller eggs are observed in hatchery‐produced or farmed fish versus wild‐reared fish (Fleming, Lamberg, & Jonsson, 1997; Solberg, Fjelldal, Nilsen, & Glover, 2014). Egg size is positively correlated with alevin size upon emergence (Solberg et al., 2014) and with survival in freshwater as reported in the present study and previously (Einum & Fleming, 2000; Skaala et al., 2012). In turn, this gave the domesticated salmon an initial maternal advantage in the first egg planting study in the River Guddalselva, but not in the present. Indeed, once egg size was controlled for in the half‐sibling design in both studies; survival differences between hybrid and domesticated salmon sharing the same eggs were more aligned between these studies. That is, there was a clear additive genetic effect of sire component in both studies. Isolation of the paternal additive genetic effect has also been demonstrated in a detailed re‐analysis of the initial data from the Burrishoole planting experiments (Reed et al., 2015) and in a study of the quantitative genetic variation in survival of domesticated, hybrid, and wild salmon in the natural habitat (Besnier et al., 2015).

In both the present and the previous study conducted in the River Guddalselva, the highest relative survival rates for domesticated fish were observed in the cohorts with lowest density of planted eggs, and vice versa. For wild fish however, the highest survival for a single family (6.0%), as well as the highest average survival, was found in C2010 with the highest (7.1 eggs per m^2^) egg density. Collectively, these observations suggest that as egg and juvenile density increase, with a corresponding increase in intraspecific competition, the survival difference between domesticated and wild salmon increases. These indications are consistent with data from studies that have revealed relatively higher introgression of domesticated salmon in low‐density or numerically small wild populations (Glover et al., 2013; Heino, Svåsand, Wennevik, & Glover, 2015).

Collectively, these results also demonstrate how spawning of domesticated salmon may reduce the natural output of wild smolt from populations through resource competition in river habitats, even without genetic introgression. Therefore, the results underline the importance of fulfilling wild population spawning targets in order to reduce spawning success of domesticated salmon, thereby securing a high density of juveniles with a correspondingly high level of competition, and a reduced introgression of genetic material from escaped domesticated salmon.

### 4.2 Timing of smolt migration

Domesticated and hybrid smolts migrated earlier in the year than wild smolts, a trend consistent across all smolt ages and cohorts, but greatest in magnitude for the 4‐year‐old smolts resulting from the 2010 cohort where the difference was ~3 weeks (Figure 5). Results from the first study conducted in the River Guddalselva (Skaala et al., 2012), and the study conducted in the River Imsa (Fleming et al., 2000) also suggested an earlier migration timing in smolts of domesticated parents. In addition, investigations in the Burrishoole system in Ireland revealed differences in the size of the window of smolt migration between domesticated and wild types (McGinnity et al., 2007). Differences observed in the experiment in the River Imsa, based upon a few hundred smolts, were 17 and 4 days for age 1 and 2 winter smolts, respectively (Ian Fleming personal communication). In the previous study in the River Guddalselva, the differences were small and not statistically significant. Consequently, the present study, based upon timing of downstream migration of 6,999 pedigree‐identified smolts over 7 years, represents the first comprehensive documentation of this phenomenon. When combined with the fact that the hybrids tended to display intermediate migrating timing, our data suggest a degree of additive genetic variation for this trait. Other genetic‐based phenological differences reported between domesticated and wild salmon include egg development timing (Fraser, Minto, Calvert, Eddington, & Hutchings, 2010; Solberg et al., 2014).

A possible explanation for the genetic difference in smolt migration timing revealed here could be that domesticated fish are farmed under strict day‐length regimes in order to synchronize and speed up smolt production (Thrush, Duncan, & Bromage, 1994). This may have altered the domesticated salmon's hormone system involved in smoltification in such a manner that domesticated smolts are more readily triggered by a change in photoperiod in the spring, and thus migrate earlier. This suggestion is supported by the observed relationship between individual smolt weight and migration date, and how this varied within and among the different types. In the wild type, larger smolts emigrated later than smaller smolts, which was not the case for the domesticated smolts.

Timing of smolt migration is likely to be adaptive to local conditions (McLennan, Rush, McKelvey, & Metcalfe, 2018; Stewart, Middlemas, & Youngson, 2006), and therefore, phenological changes imposed upon natural populations following introgression and hybridization of domesticated salmon are likely to be maladaptive. However, while earlier migration implies that domesticated or admixed salmon smolts enter the marine environment earlier than wild smolts, the ultimate consequences of this change on marine survival are unknown and may vary in time and space. For example, in coastal areas with intensive salmon farming and high infestation pressure of salmon lice (*Lepeophtheirus salmonis*) (Taranger et al., 2015; Vollset et al., 2016), it could be advantageous to migrate out of coastal areas before the lice population builds up in spring and early summer, thus providing domesticated smolts with an earlier time of migration an “artificial advantage”.

### 4.3 Marine survival and straying

Atlantic salmon return rates are known to vary greatly among years and populations (1%–3% for MSW salmon vs. 3%–10% for 1SW salmon) and with lower recapture rates for hatchery‐produced smolts (Chaput, 2012). Marine survival (i.e., return rate) of the hatchery‐produced smolts released into the River Guddalselva in 2011 and 2012 was low compared with other studies (Chaput, 2012; Jonsson & Jonsson, 2017). Nevertheless, for the two releases combined, survival from smolt to adult was lowest in the domesticated salmon (0.41%) and highest for the hybrid (0.73%) and wild (0.94%) types. Therefore, the current study shows that while in the freshwater phase, hybrids commonly perform intermediate between domesticated and wild salmon, in the marine phase, hybrids may perform quite well, suggesting a degree of nonadditive variation for this trait.

The lower survival of domesticated salmon observed here is in agreement with hatchery‐produced smolt‐release studies conducted in the River Burrishoole in Ireland (McGinnityet al., 2003, 1997) and in the River Imsa in Norway (Jonsson & Jonsson, 2017). In contrast, an earlier study conducted in the River Imsa based upon the release of domesticated spawners into the river system, and natural production of wild, hybrid, and domesticated smolts thereafter, did not find differences in marine survival among domesticated and wild salmon (Fleming et al., 2000). Studying marine survival is, however, highly challenging. These challenges involve the pros and cons of studying hatchery releases versus natural smolt migrations and the associated sample size issues, and the fact that marine experiments take many years to conduct and require special sampling infrastructure. Furthermore, fish may return to spawn after 1, 2, 3, or more years in the sea, a trait known to be under the strong genetic influence of the *vgll3* locus (Ayllon et al., 2015; Barson et al., 2015). The previous studies conducted in both the Burrishoole and the Imsa rivers involved comparisons to wild grilse populations where the majority of salmon return to spawn after one year in the sea. Those studies found that the wild type dominated completely among salmon that returned as 1SW fish while among 2SW fish, hybrids and domesticated type dominated. In the salmon population inhabiting the river Etne however, the majority of salmon returned after 2 years in the sea (Harvey, Tang, Wennevik, Skaala, & Glover, 2017). As a result, we observed that the wild type tended to be older than hybrids and domesticated types at return to spawn, albeit with sex differences. Differences in age upon return from the sea between domesticated‐admixed and wild salmon have been shown to be different among wild populations (Bolstad et al., 2017) and are therefore likely to vary greatly in time and space.

The recent release of hatchery‐produced smolts into the River Imsa (Jonsson & Jonsson, 2017) revealed survival rates for domesticated (0.6%), hybrid (1.0%–1.96%), and wild salmon (1.5%) that are comparable, but slightly higher than the survival rates observed in our study (Table 2). This probably reflects population‐specific differences, year differences, and/or environmental differences in the marine migration routes. The highest marine survival of fish released from the River Imsa was observed for hybrids with wild mothers. Interestingly, the highest survival for the hatchery‐produced smolt in our study was found for the hybrid group released in 2012, although not significantly higher than that of the wild type. In our study, the hybrids were produced with domesticated mothers sired with wild fathers which are likely to be the most common type of F1‐hybrids (Fleming et al., 2000; Fleming, Jonsson, Gross, & Lamberg, 1996). This means that once hybrids have survived the freshwater stage, their survival during ocean migration may be similar to or higher than that of wild salmon, and in turn act as a highway for further introgression and admixture. Furthermore, the nonintermediate survival of F1‐hybrids in comparison with wild and domesticated salmon suggests that the results of introgression and admixture may be challenging to predict in the marine phase.

Despite extensive checks of wild and tagged spawning salmon in other rivers in the Hardangerfjord during the summer and pre‐spawning period, ~95% of the controlled spawners were caught in Guddalselva, that is, the release river, and in neighboring rivers within a 3 km radius. Significantly, we found no evidence of differences in straying rates among types, which stands in contrast to the results from the River Imsa (Jonsson & Jonsson, 2017) where increased straying was reported in domesticated salmon and hybrids where the mother was of domesticated origin.

### 4.4 Freshwater and marine growth

Although it varied between age groups, cohorts, and life stages investigated, in comparison with wild salmon, domesticated fish only displayed marginally larger size at age in freshwater, yet far greater size at age in the marine environment. Typically, hybrids displayed intermediate size at age. Domesticated salmon have undergone directional selection for fast growth since the first breeding populations were established in the early 1970s (Gjedrem, 2000, 2010), and as a consequence, are typically 2–4 times larger than wild salmon when reared together under aquaculture conditions in both the freshwater and marine environments (Glover et al., 2009; Harvey et al., 2016; Solberg, Glover et al., 2013; Solberg, Zhang et al., 2013). In studies conducted in rivers where food resources are potentially limited and density‐dependent survival exists (Bacon et al., 2015; Jonsson, Jonsson, & Hansen, 1998), the full growth potential of domesticated salmon is typically not realized, and the growth rate of offspring of domesticated parents is only marginally higher than that of wild, as revealed here, and in previous studies (Besnier et al., 2015; Einum & Fleming, 2000; Fleming et al., 2000; Reed et al., 2015; Skaala et al., 2012). Recently, a study demonstrated that a combination of energy‐budget plasticity, combined with selection against fast growing domesticated fish in the wild, is responsible for the large differences in growth reaction norms between domesticated and wild salmon across the farming and natural environments (Glover, Solberg, Besnier, & Skaala, 2018). Or put alternatively, these authors suggested that in the wild, offspring of domesticated salmon do not have the ability to acquire enough energy in relation to their expenditure, and simultaneously, domesticated salmon displaying the greatest genetic growth potential are more likely to take risks, as indicated from behavioral studies (Fleming & Einum, 1997; Houde, Fraser, & Hutchings, 2010), and therefore succumb to predation. The higher mortality rates of domesticated salmon in freshwater, as revealed by several studies, support this suggestion.

In the context of migrating salmon, the marine environment is unlikely to exhibit the same density‐dependent constraints on survival and growth that are observed in rivers with finite production capacities (Jonsson et al., 1998). Although domesticated fish of both sexes outgrew wild salmon at sea age 1, 2, and 3, their marine survival rate was lower, suggesting adequate feeding behavior, but a dysfunction in some other behavioral components. In comparison with freshwater experiments, there are few data on the marine growth of domesticated and wild salmon. Therefore, data from the present study extend upon knowledge from previous studies to also include wild MSW salmon populations, and it confirms the observed reduced survival and increased growth rate in domesticated salmon compared with wild salmon in earlier studies (Fleming et al., 2000; Jonsson & Jonsson, 2017; McGinnityet al., 2003, 1997).

### 4.5 “Lifetime” fitness differences between domesticated and wild salmon

Atlantic salmon has a life cycle involving freshwater–marine–freshwater transitions within a generation, but also life‐history variations such as precocious male parr that can complete the life cycle in freshwater alone. Consequently, computing “lifetime” fitness differences between domesticated and wild salmon is not straightforward, and it is also conditioned by the start‐point chosen. In the River Imsa, by permitting farmed escapees to ascend the river to spawn naturally with wild fish, and monitoring offspring survival up to and including adult ascendance, the relative “lifetime” fitness of domesticated salmon was computed as 16% of wild fish (Fleming et al., 2000). However, large differences in initial spawning success had a major influence on the result, and therefore, that result is not directly comparable with the “lifetime” fitness result from the study conducted in the River Burrishoole where the relative “lifetime” fitness of domesticated fish was computed as 2% of wild fish from eggs through to returning adult survival, including fertilization differences (McGinnity et al., 2003). In the present study, we computed the relative “lifetime” fitness differences in domesticated to wild as roughly 21% or 30% depending on data used (egg to returning adult). Our result is likely to represent a minimum estimate of the fitness differential between domesticated and wild because we have not used a locally adapted wild reference population. Therefore, our fitness differences between domesticated and wild are more conservative than in the Irish study, which was primarily caused through differences in results in the marine phase. What factors may have influenced this, for example, multi‐sea winter (here) versus grilse (Burrishoole) populations, and/or Norwegian domesticated salmon in a Norwegian wild (here) versus an Irish wild (Burrishoole) population, or other factors, remains difficult to entangle.

### 4.6 Management implications

Introgression of domesticated escapees in native salmon populations represents a challenge to the genetic integrity, productivity, life‐history characteristics, and evolutionary trajectory of wild populations (Glover et al., 2017). Important advances in knowledge have been gained in recent years, such as the widespread documentation of introgression levels in native populations (Glover et al., 2013; Karlsson et al., 2016; Keyser et al., 2018), evidence of life‐history changes as a result of introgression and admixture (Bolstad et al., 2017), and estimates of long‐term responses in demographic and life‐history traits projected through eco‐genetic modeling (Castellani et al., 2018). However, while much attention has focused on the direct genetic effects via introgression and hybridization, less attention has been paid to the potential reduction in wild smolt production, simply due to the presence of domesticated offspring and direct resource competition in rivers. The present study provides increased detail in the type and magnitude of genetic differences between domesticated and wild salmon in the freshwater and marine environment, and how they may affect wild populations either through resource competition and reduced smolt output, and/or by reducing the fitness of the wild population through introgression and hybridization. Collectively, all evidence points toward negative effects of domestication‐admixture. Therefore, increased efforts to minimize further introgression and admixture, through a combination of reduced escape from farms, and prespawning removal of escapees from wild populations are recommended.

### CONFLICT OF INTEREST

None declared.

## DATA ARCHIVING

The raw data underlying the study consist of data of broodstock and biological data, etc. for 6,669 individual salmon smolts. These, and metadata, will be archived and made accessible at the storage facilities at Norwegian Marine Data center (NMDC) at the Institute of Marine Research, Bergen, Norway: https://doi.org/10.21335/NMDC-806700432.
