# Supplementary figures and images for: An extensive common‐garden study with domesticated and wild Atlantic salmon in the wild reveals impact on smolt production and shifts in fitness traits
Source: Evol Appl. 2019 Mar 6;12(5):1001–16. doi: 10.1111/eva.12777 (PMC6503829; doi:10.1111/eva.12777)

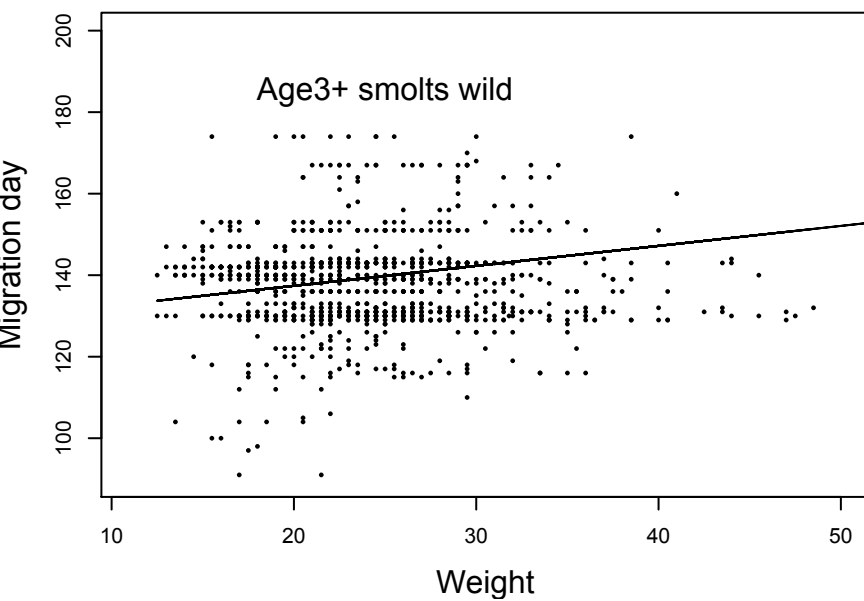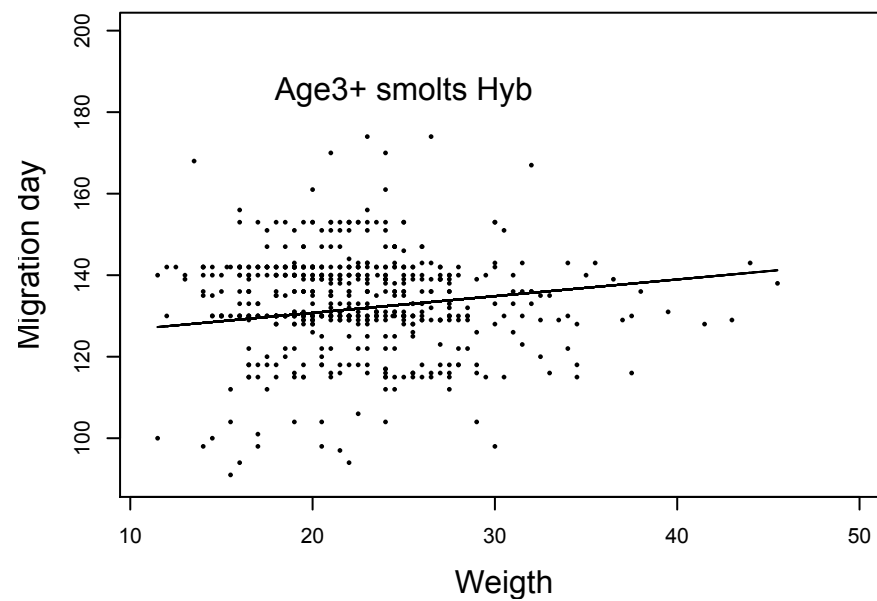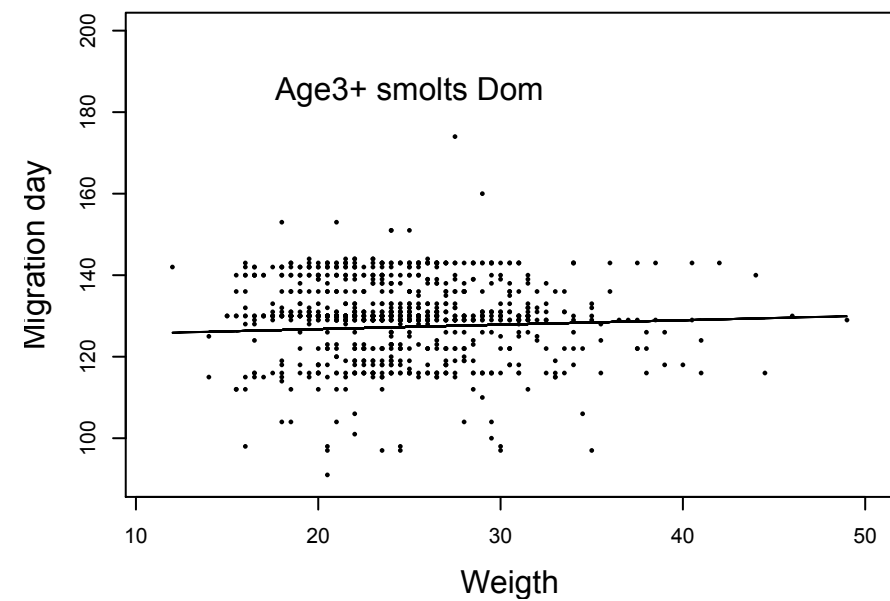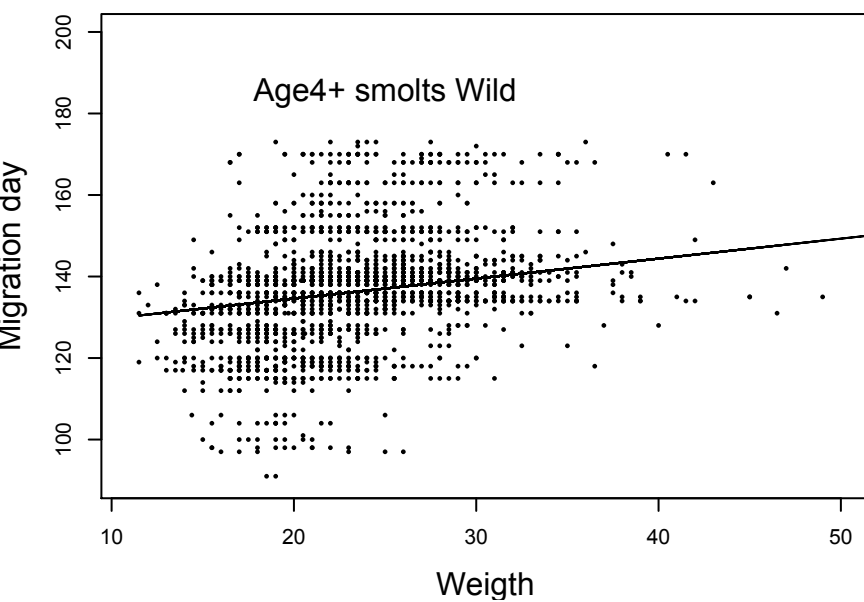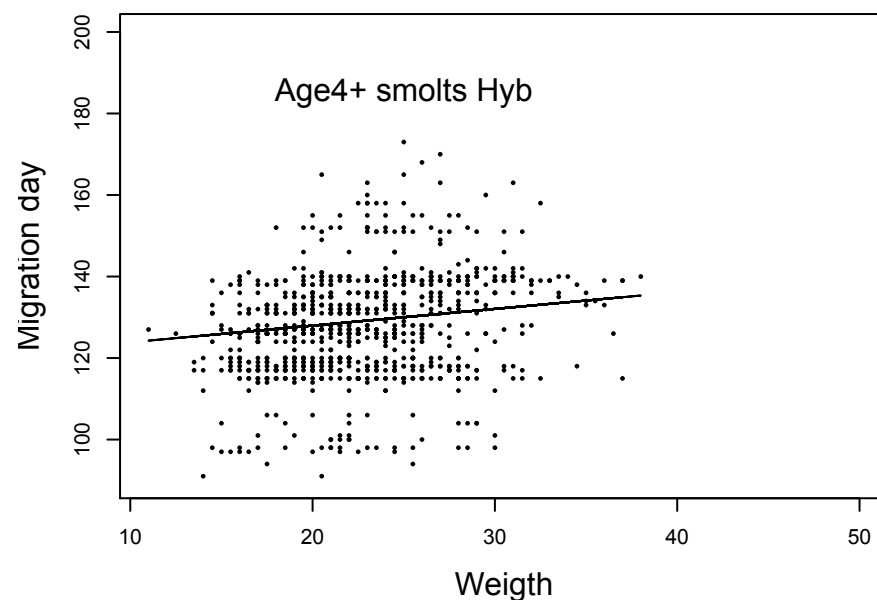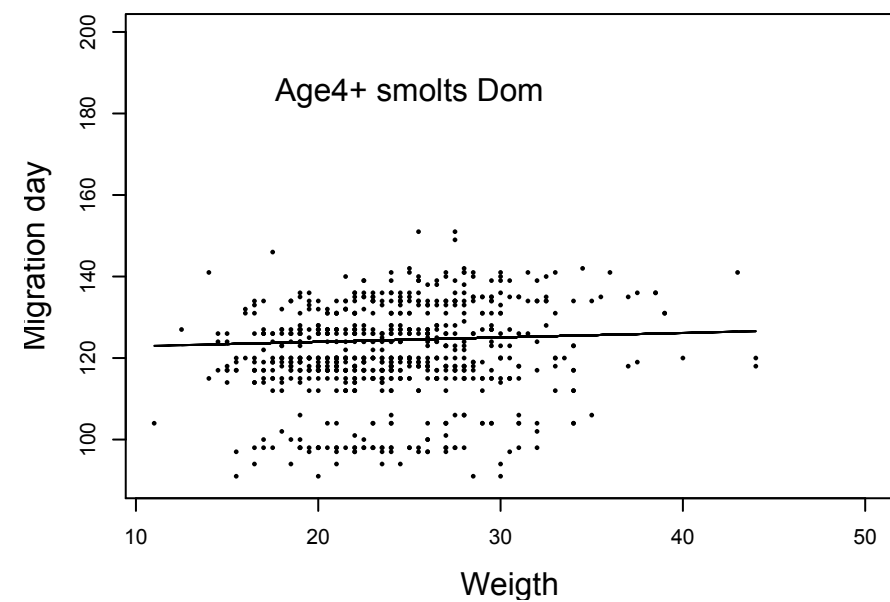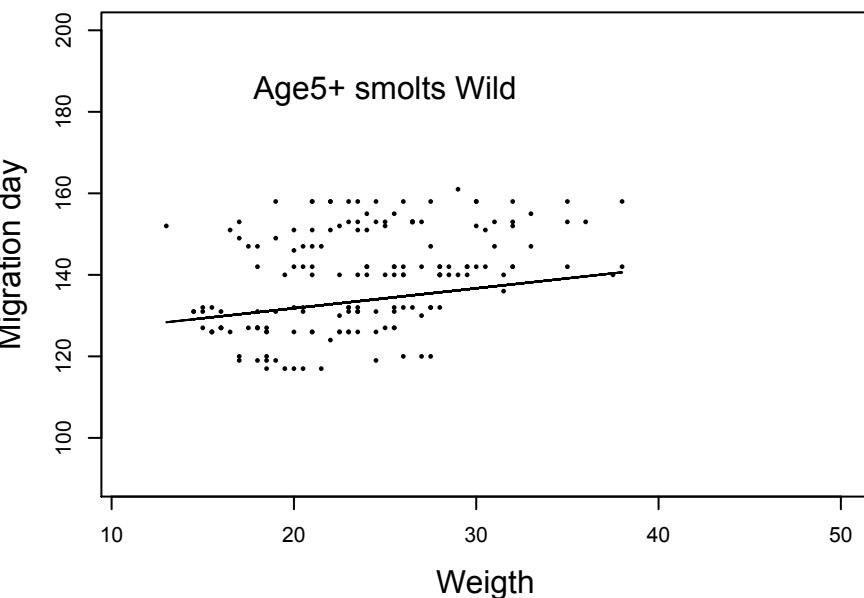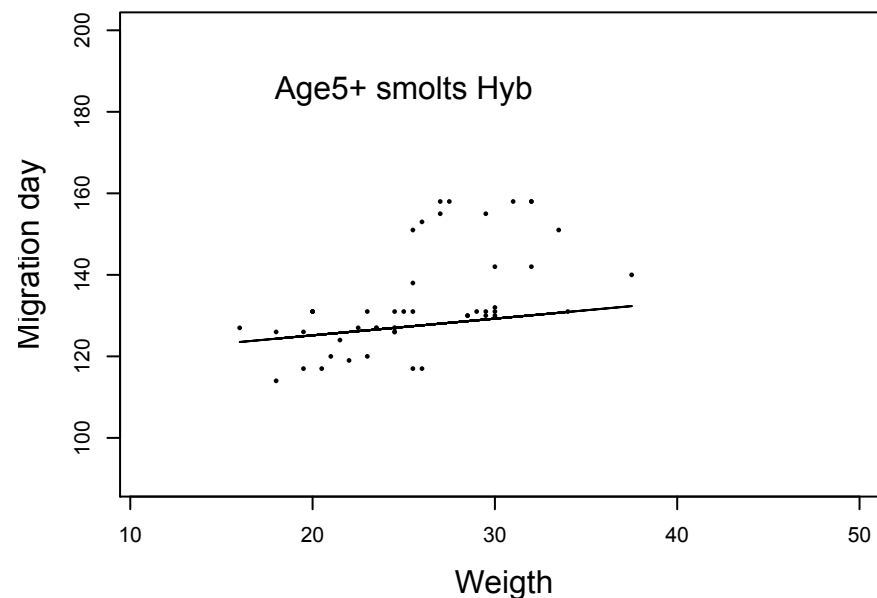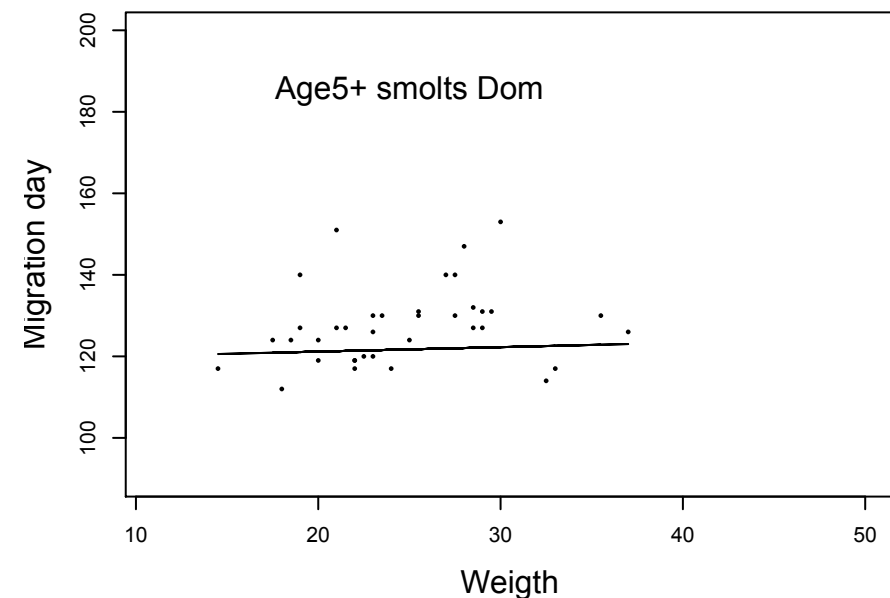

Supplement: Supplementary file 1 [file EVA-12-1001-s001.pdf]
