# Supplementary material for: An extensive common‐garden study with domesticated and wild Atlantic salmon in the wild reveals impact on smolt production and shifts in fitness traits
Source: Evol Appl. 2019 Mar 6;12(5):1001–16. doi: 10.1111/eva.12777 (PMC6503829; doi:10.1111/eva.12777)
Supplement: Supplementary file 2 [file EVA-12-1001-s002.pdf]

Guddalselva – The River Laboratory

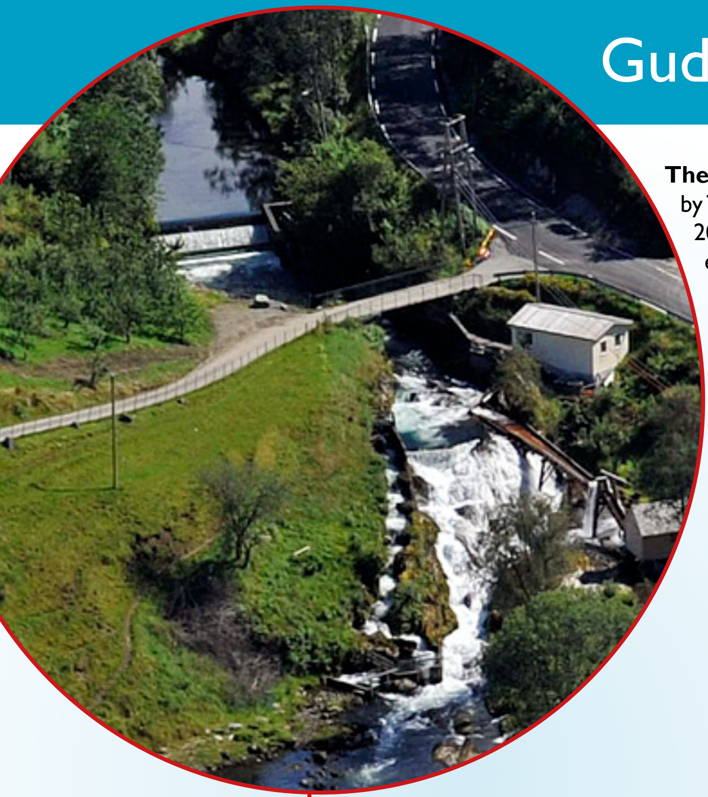

The River Laboratory was established by The Institute of Marine Research in 2000 in collaboration with the Guddalselva River Owners, The Norwegian Water Resources and Energy Directorate, The Norwegian Environment Agency, The Norwegian Directorate of Fisheries and The Hordaland County Governor in order to conduct common garden studies and monitoring of physical and biological parameters.

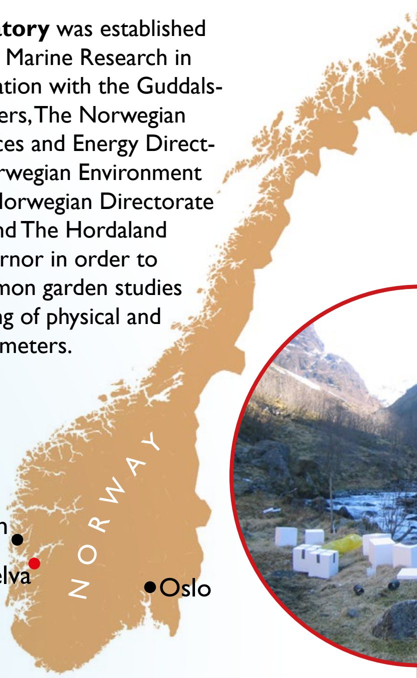

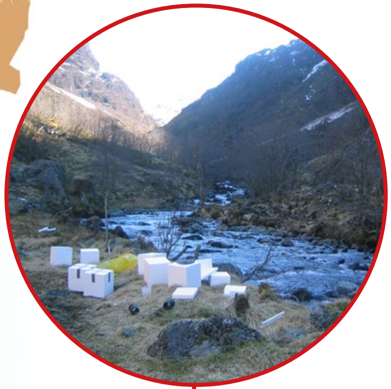

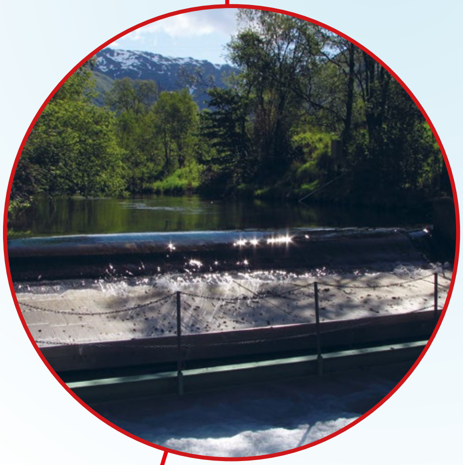

The station and the traps.

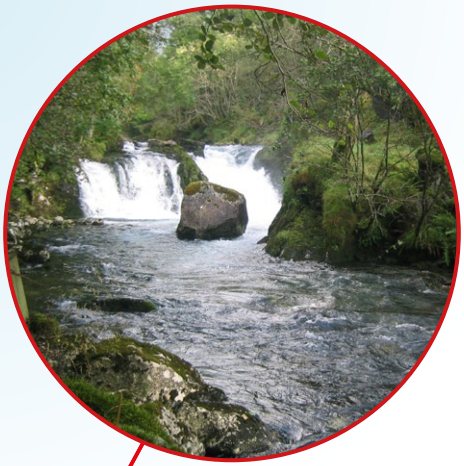

The Liarefossen waterfall.

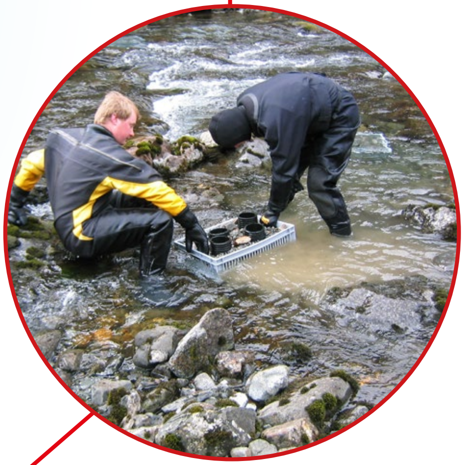

From the egg-planting area.

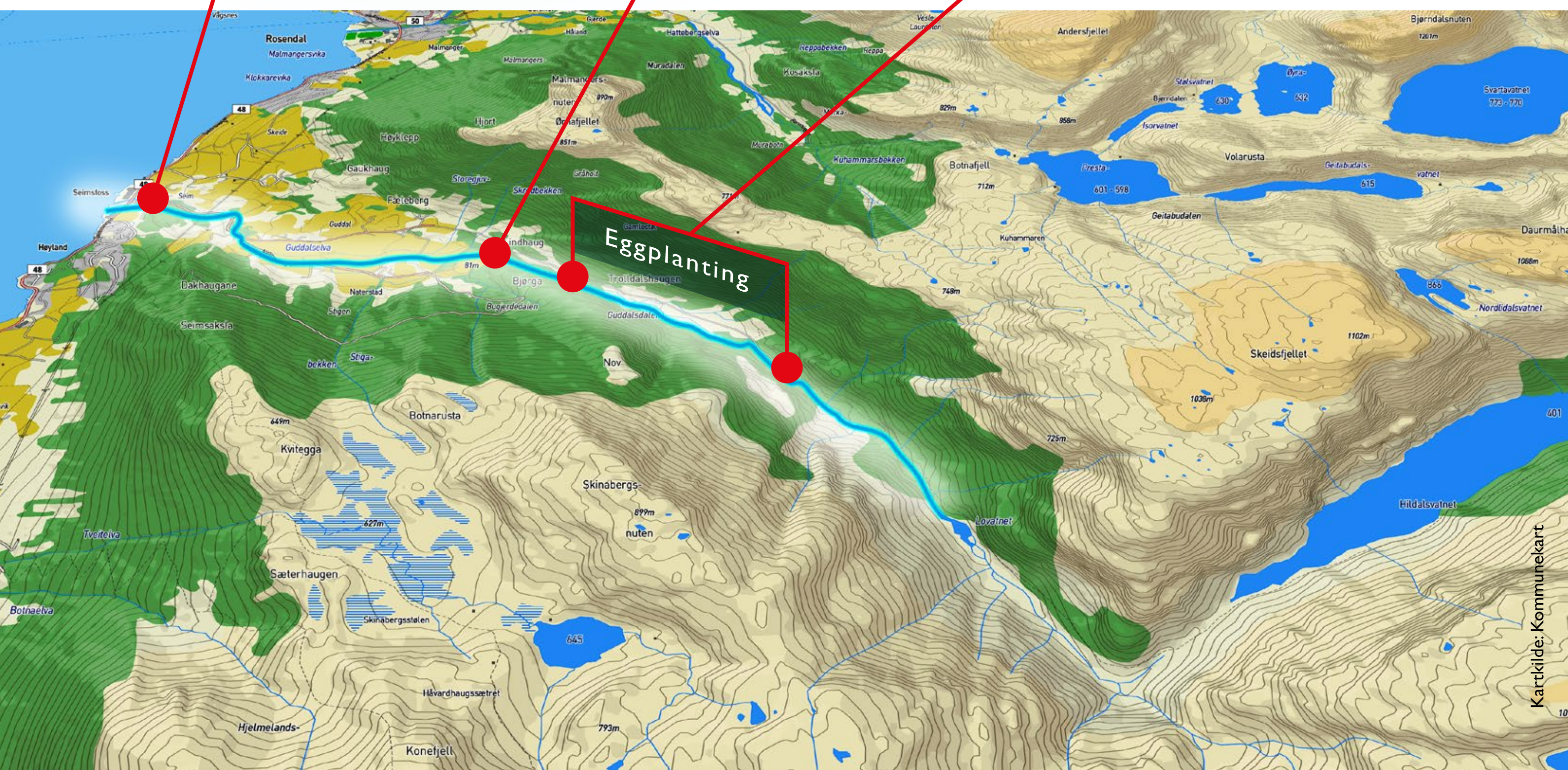

Kartkilde: Kommunekart
