# Supplementary material for: An extensive common‐garden study with domesticated and wild Atlantic salmon in the wild reveals impact on smolt production and shifts in fitness traits
Source: Evol Appl. 2019 Mar 6;12(5):1001–16. doi: 10.1111/eva.12777 (PMC6503829; doi:10.1111/eva.12777)
Supplement: Supplementary file 3 [file EVA-12-1001-s003.pdf]

Supplementary Table S1. Broodfish 2007, 2009, 2010 for egg planting in R Guddalelva 2008, 2010 and 2011, and for releases of hatchery produ

Supplementary Table 1. Brood stock year, stock, date of fertilization, family numbering, type, number of eggs planted and size of eggs.

Br year	Stock	Female	New female numb (DNA tube)	Stock	Male	New male numb (DNA tube)	Fertilisation date	Fam nr.	Expt. Group	To Guddal	Eggs 25cm	ıeter 1 egg (cm)
2007	Etne	11	2	Etne	7	1	19.nov.07	0	Wild	---	---	---
2007	Etne	18	3	Etne	7	1	19.nov.07	0	Wild	---	---	---
2007	Etne	14	4	Etne	5	7	19.nov.07	3	Wild	5600	38	0,657895
2007	Etne	23	5	Etne	5	7	19.nov.07	4	Wild	6400	37	0,675676
2007	Etne	20	6	Etne	2	9	19.nov.07	5	Wild	6000	37	0,675676
2007	Etne	22	8	Etne	2	9	19.nov.07	6	Wild	1800	38	0,657895
2007	Etne	21	10	Etne	3	11	19.nov.07	7	Wild	2400	38	0,657895
2007	Etne	19	13	Etne	9	12	19.nov.07	0	Wild	---	---	---
2007	Etne	25	15	Etne	6	14	19.nov.07	9	Wild	6600	41	0,609756
2007	Etne	24	17	Etne	8	16	19.nov.07	10	Wild	3000	39	0,641026
2007	Mowi	1	18	Mowi	1	28	20.nov.07	11	Farm	3400	42	0,595238
2007	Mowi	1	18	Etne	3	11	20.nov.07	0	Hybrid	---	---	---
2007	Mowi	2	19	Mowi	2	29	20.nov.07	13	Farm	3400	45	0,555556
2007	Mowi	2	19	Etne	3	11	20.nov.07	0	Hybrid	---	---	---
2007	Mowi	5	20	Mowi	3	30	20.nov.07	0	Farm	---	---	---
2007	Mowi	5	20	Etne	9	12	20.nov.07	0	Hybrid	---	---	---
2007	Mowi	6	21	Mowi	4	31	20.nov.07	17	Farm	3400	40	0,625
2007	Mowi	6	21	Etne	9	12	20.nov.07	0	Hybrid	---	---	---
2007	Mowi	7	22	Mowi	5	32	20.nov.07	19	Farm	3800	42	0,595238
2007	Mowi	7	22	Etne	6	14	20.nov.07	0	Hybrid	---	---	---
2007	Mowi	8	23	Mowi	6	33	20.nov.07	21	Farm	3000	45	0,555556
2007	Mowi	8	23	Etne	6	14	20.nov.07	0	Hybrid	---	---	---
2007	Mowi	9	24	Mowi	7	34	20.nov.07	23	Farm	4000	43	0,581395
2007	Mowi	9	24	Etne	8	16	20.nov.07	0	Hybrid	---	---	---
2007	Mowi	10	25	Mowi	8	35	20.nov.07	25	Farm	3600	40	0,625
2007	Mowi	10	25	Etne	7	1	20.nov.07	0	Hybrid	---	---	---
2007	Mowi	11	26	Mowi	9	36	20.nov.07	27	Farm	4000	42	0,595238
2007	Mowi	11	26	Etne	5	7	20.nov.07	28	Hybrid	5400	42	0,595238

2007	Mowi	12	27	Mowi	10	37	20.nov.07	0	Farm	---	---	---
2007	Mowi	12	27	Etne	2	9	20.nov.07	30	Hybrid	4000	44	0,568182
2009	Mowi	M1	1	Mowi	M11	11	17.11.2009	31	F	4000	40	
2009	Mowi	M1	1	Etne	969	27	17.11.2009	32	H	3000		
2009	Mowi	M2	2	Mowi	M12	12	17.11.2009	33	F	4000	40	
2009	Mowi	M2	2	Etne	969	27	17.11.2009	34	H	4000		
2009	Mowi	M3	3	Mowi	M13	13	17.11.2009	35	F	4000	39	
2009	Mowi	M3	3	Etne	102	0	17.11.2009	0	H	3000		
2009	Mowi	M4	4	Mowi	M14	14	17.11.2009	37	F	4000	41	
2009	Mowi	M4	4	Etne	102	0	17.11.2009	0	H	4000		
2009	Mowi	M5	5	Mowi	M15	15	17.11.2009	39	F	4000	41	
2009	Mowi	M5	5	Etne	3	0	17.11.2009	0	H	4000		
2009	Mowi	M6	6	Mowi	M16	16	17.11.2009	41	F	4000	42	
2009	Mowi	M6	6	Etne	8	0	17.11.2009	0	H	4000	41	
2009	Mowi	M7	7	Mowi	M17	17	17.11.2009	43	F	4000	41	
2009	Mowi	M7	7	Etne	10	38	17.11.2009	44	H	4000		
2009	Mowi	M8	8	Mowi	M18	18	17.11.2009	45	F	4000	39	
2009	Mowi	M9	9	Mowi	M19	19	17.11.2009	47	F	4200		
2009	Mowi	M9	9	Etne	131	28	17.11.2009	48	H	2000	39	
2009	Mowi	M10	10	Mowi	M20	20	17.11.2009	0	F	3000		
2009	Mowi	M10	10	Etne	343	35	17.11.2009	50	H	4000	39	
2009	Etne	17	33	Etne	343	35	17.11.2009	51	W	4000		
2009	Etne	100	21	Etne	343	35	17.11.2009	52	W	4000	39	
2009	Etne	18	31	Etne	3	0	17.11.2009	0	W	2000	39	
2009	Etne	225	0	Etne	3	0	17.11.2009	0	W	4000	37	
2009	Etne	246	29	Etne	969	27	17.11.2009	55	W	4000	37	
2009	Etne	404	32	Etne	10	38	17.11.2009	56	W	2400	38	
2009	Etne	207	24	Etne	131	28	17.11.2009	57	W	3600	40,5	
2009	Etne	462	22	Etne	263	23	17.11.2009	58	W	3600	41	
2009	Etne	387	30	Etne	102	0	17.11.2009	0	W	4000	39	
2009	Etne	20	25	Etne	8	0	17.11.2009	0	W	3200	39	

2010	Mowi	1	1	Mowi	M11	11	16.11.2010	61	F	3000	41
2010	Mowi	1	1	Etne	E28	28	16.11.2010	62	H	3000	
2010	Mowi	2	2	Mowi	M12	12	16.11.2010	63	F	3000	40
2010	Mowi	2	2	Etne	E29	29	16.11.2010	64	H	3000	
2010	Mowi	3	3	Mowi	M13	13	16.11.2010	65	F	3000	40
2010	Mowi	3	3	Etne	E30	30	16.11.2010	66	H	3000	
2010	Mowi	4	4	Mowi	M14	14	16.11.2010	67	F	3000	43
2010	Mowi	4	4	Etne	E36	36	16.11.2010	68	H	2600	
2010	Mowi	5	5	Mowi	M15	15	16.11.2010	69	F	3000	40
2010	Mowi	5	5	Etne	E31	31	16.11.2010	70	H	3000	
2010	Mowi	6	6	Mowi	M16	16	16.11.2010	71	F	3000	40
2010	Mowi	6	6	Etne	E32	32	16.11.2010	72	H	3000	
2010	Mowi	7	7	Mowi	M17	17	16.11.2010	73	F	3000	41
2010	Mowi	7	7	Etne	E33	33	16.11.2010	74	H	3000	
2010	Mowi	8	8	Mowi	M18	18	16.11.2010	75	F	3000	40
2010	Mowi	8	8	Etne	E35	35	16.11.2010	76	H	3000	
2010	Mowi	9	9	Mowi	M19	19	16.11.2010	77	F	3000	39
2010	Mowi	9	9	Etne	E37	37	16.11.2010	78	H	3000	
2010	Mowi	10	10	Mowi	M20	20	16.11.2010	79	F	3000	40
2010	Mowi	10	10	Etne	E34	34	16.11.2010	80	H	3000	
2010	Etne	402	21	Etne	E28	28	16.11.2010	81	W	1000	41
2010	Etne	150	22	Etne	E29	29	16.11.2010	82	W	2000	40
2010	Etne	187	23	Etne	E30	30	16.11.2010	83	W	3000	37
2010	Etne	33	24	Etne	E31	31	16.11.2010	84	W	2000	36
2010	Etne	21	25	Etne	E32	32	16.11.2010	85	W	2000	39
2010	Etne	120	26	Etne	E33	33	16.11.2010	86	W	1000	40
2010	Etne	80	27	Etne	E34	34	16.11.2010	87	W	3000	39
2010			0	Etne	E35	0	16.11.2010	0		0	
2010	Etne	33	24	Etne	E36	36	16.11.2010	89	W	2000	36
2010	Etne	21	25	Etne	E37	37	16.11.2010	90	W	3000	40
