# Supplementary material for: An extensive common‐garden study with domesticated and wild Atlantic salmon in the wild reveals impact on smolt production and shifts in fitness traits
Source: Evol Appl. 2019 Mar 6;12(5):1001–16. doi: 10.1111/eva.12777 (PMC6503829; doi:10.1111/eva.12777)
Supplement: Supplementary file 4 [file EVA-12-1001-s004.pdf]

Individ_nr_fra_Ge	Year_smol	Dipl_Trip	Day_smolt_e	Bio-Lop	Length	Weight	Family-27_200	Gen-Lop	Sorting	Sorting	Fam_n	Brood_st	W/Farm/I
2171_1081_S1_B(	2011	D	15.04.2011	S1	13.8	20.5	Fam-04_2007_	S1	1	1	4	2007	W
2171_1081_S2_C(	2011	D	20.04.2011	S2	15.7	29	Fam-17_2007_	S2	2	2	17	2007	F
2171_1081_S3_D(	2011	D	20.04.2011	S3	15.7	29.5	Fam-09_2007_	S3	3	3	9	2007	W
2171_1081_S5_F(	2011	D	26.04.2011	S5	14.5	24.5	Fam-13_2007_	S5	5	4	13	2007	F
2171_1081_S6_G(	2011	D	26.04.2011	S6	15	26	Fam-09_2007_	S6	6	5	9	2007	W
2171_1081_S7_H(	2011	D	26.04.2011	S7	14.8	25	Fam-27_2007_	S7	7	6	27	2007	F
2171_1081_S8_A(	2011	D	26.04.2011	S8	15.5	30	Fam-19_2007_	S8	8	7	19	2007	F
2171_1081_S9_B(	2011	D	26.04.2011	S9	14.2	22.5	Fam-17_2007_	S9	9	8	17	2007	F
2171_1081_S10_(	2011	D	26.04.2011	S10	14.4	24	Fam-19_2007_	S10	10	9	19	2007	F
2171_1081_S11_I	2011	D	26.04.2011	S11	15	27	Fam-28_2007_	S11	11	10	28	2007	H
2171_1081_S12_E	2011	D	26.04.2011	S12	14.2	23	Fam-11_2007_	S12	12	11	11	2007	F
2171_1081_S13_F	2011	D	26.04.2011	S13	13	18	Fam-13_2007_	S13	13	12	13	2007	F
2171_1081_S14_(	2011	D	26.04.2011	S14	16.9	36	Fam-04_2007_	S14	14	13	4	2007	W

2171_1081_S15_H	2011	D	26.04.2011	S15	14.5	25	Fam-19_2007_S15	15	14	19	2007 F
2171_1081_S16_A	2011	D	26.04.2011	S16	18.2	44.5	Fam-23_2007_S16	16	15	23	2007 F
2171_1081_S17_E	2011	D	26.04.2011	S17	16.3	33.5	Fam-13_2007_S17	17	16	13	2007 F
2171_1081_S18_C	2011	D	26.04.2011	S18	14.1	22	Fam-27_2007_S18	18	17	27	2007 F
2171_1081_S19_D	2011	D	26.04.2011	S19	14.4	22	Fam-19_2007_S19	19	18	19	2007 F
2171_1081_S20_E	2011	D	26.04.2011	S20	14.6	26	Fam-11_2007_S20	20	19	11	2007 F
2171_1081_S21_F	2011	D	26.04.2011	S21	14.7	24	Fam-13_2007_S21	21	20	13	2007 F
2171_1081_S22_G	2011	D	26.04.2011	S22	16.1	33	Fam-21_2007_S22	22	21	21	2007 F
2171_1081_S23_H	2011	D	26.04.2011	S23	14.6	25	Fam-13_2007_S23	23	22	13	2007 F
2171_1081_S25_E	2011	D	26.04.2011	S25	15.1	27.5	Fam-13_2007_S25	25	23	13	2007 F
2171_1081_S26_C	2011	D	26.04.2011	S26	15.1	26.5	Fam-28_2007_S26	26	24	28	2007 H
2171_1081_S27_D	2011	D	26.04.2011	S27	16	32.5	Fam-19_2007_S27	27	25	19	2007 F
2171_1081_S28_E	2011	D	26.04.2011	S28	13.1	20	Fam-19_2007_S28	28	26	19	2007 F
2171_1081_S29_F	2011	D	26.04.2011	S29	13.6	19.5	Fam-27_2007_S29	29	27	27	2007 F

2171_1081_S31_H	2011	D	26.04.2011	S31	14.1	22	Fam-19_2007_	S31	31	28	19	2007	F
2171_1081_S32_A	2011	D	26.04.2011	S32	14.8	25.5	Fam-28_2007_	S32	32	29	28	2007	H
2171_1081_S33_B	2011	D	26.04.2011	S33	14	21.5	Fam-27_2007_	S33	33	30	27	2007	F
2171_1081_S34_C	2011	D	26.04.2011	S34	15.4	29.5	Fam-13_2007_	S34	34	31	13	2007	F
2171_1081_S35_D	2011	D	26.04.2011	S35	16.4	31	Fam-23_2007_	S35	35	32	23	2007	F
2171_1081_S36_E	2011	D	26.04.2011	S36	15.8	29.5	Fam-21_2007_	S36	36	33	21	2007	F
2171_1081_S37_F	2011	D	26.04.2011	S37	16.4	33	Fam-28_2007_	S37	37	34	28	2007	H
2171_1081_S38_G	2011	D	26.04.2011	S38	14.7	24	Fam-13_2007_	S38	38	35	13	2007	F
2171_1081_S39_H	2011	D	26.04.2011	S39	14.4	23	Fam-27_2007_	S39	39	36	27	2007	F
2171_1081_S40_A	2011	D	26.04.2011	S40	13	17.5	Fam-11_2007_	S40	40	37	11	2007	F
2171_1081_S41_B	2011	D	26.04.2011	S41	17.3	41	Fam-27_2007_	S41	41	38	27	2007	F
2171_1081_S42_C	2011	D	26.04.2011	S42	13.4	16.5	Fam-11_2007_	S42	42	39	11	2007	F
2171_1081_S43_D	2011	D	26.04.2011	S43	15.4	28.5	Fam-23_2007_	S43	43	40	23	2007	F
2171_1081_S44_E	2011	D	26.04.2011	S44	14.2	21	Fam-23_2007_	S44	44	41	23	2007	F

2171_1081_S45_F	2011	D	26.04.2011	S45	13.6	19	Fam-28_2007_	S45	45	42	28	2007	H
2171_1081_S46_G	2011	D	26.04.2011	S46	13.6	20	Fam-27_2007_	S46	46	43	27	2007	F
2171_1081_S47_H	2011	D	26.04.2011	S47	14.8	27	Fam-21_2007_	S47	47	44	21	2007	F
2171_1081_S48_A	2011	D	26.04.2011	S48	14.5	23	Fam-23_2007_	S48	48	45	23	2007	F
2171_1081_S49_B	2011	D	26.04.2011	S49	14.9	27	Fam-23_2007_	S49	49	46	23	2007	F
2171_1081_S51_D	2011	D	26.04.2011	S51	14.5	25.5	Fam-17_2007_	S51	51	47	17	2007	F
2171_1081_S52_E	2011	D	26.04.2011	S52	15.2	31	Fam-11_2007_	S52	52	48	11	2007	F
2171_1081_S53_F	2011	D	26.04.2011	S53	14.3	23.5	Fam-13_2007_	S53	53	49	13	2007	F
2171_1081_S54_G	2011	D	26.04.2011	S54	15.5	28.5	Fam-09_2007_	S54	54	50	9	2007	W
2171_1081_S55_H	2011	D	26.04.2011	S55	14.3	22.5	Fam-13_2007_	S55	55	51	13	2007	F
2171_1081_S56_A	2011	D	26.04.2011	S56	14.4	24	Fam-28_2007_	S56	56	52	28	2007	H
2171_1081_S57_B	2011	D	26.04.2011	S57	14.9	26.5	Fam-19_2007_	S57	57	53	19	2007	F
2171_1081_S58_C	2011	D	26.04.2011	S58	13.9	22	Fam-04_2007_	S58	58	54	4	2007	W
2171_1081_S59_D	2011	D	26.04.2011	S59	15.7	29	Fam-23_2007_	S59	59	55	23	2007	F

2171_1081_S60_E	2011	D	26.04.2011	S60	14.2	21.5	Fam-28_2007_	S60	60	56	28	2007	H
2171_1081_S61_F	2011	D	26.04.2011	S61	13.7	21	Fam-30_2007_	S61	61	57	30	2007	H
2171_1081_S62_G	2011	D	26.04.2011	S62	15	27.5	Fam-05_2007_	S62	62	58	5	2007	W
2171_1081_S63_H	2011	D	26.04.2011	S63	13.9	20	Fam-23_2007_	S63	63	59	23	2007	F
2171_1081_S64_A	2011	D	26.04.2011	S64	14.1	23	Fam-21_2007_	S64	64	60	21	2007	F
2171_1081_S65_E	2011	D	26.04.2011	S65	13.5	20	Fam-13_2007_	S65	65	61	13	2007	F
2171_1081_S67_D	2011	D	26.04.2011	S67	14.8	24.5	Fam-11_2007_	S67	67	62	11	2007	F
2171_1081_S68_E	2011	D	26.04.2011	S68	15.2	29	Fam-19_2007_	S68	68	63	19	2007	F
2171_1081_S69_F	2011	D	26.04.2011	S69	14.9	26	Fam-23_2007_	S69	69	64	23	2007	F
2171_1081_S70_G	2011	D	26.04.2011	S70	15.5	29	Fam-28_2007_	S70	70	65	28	2007	H
2171_1081_S71_H	2011	D	26.04.2011	S71	16	33.5	Fam-09_2007_	S71	71	66	9	2007	W
2171_1081_S72_A	2011	D	26.04.2011	S72	15	26.5	Fam-23_2007_	S72	72	67	23	2007	F
2171_1081_S73_E	2011	D	26.04.2011	S73	16.2	35	Fam-04_2007_	S73	73	68	4	2007	W
2171_1081_S75_D	2011	D	26.04.2011	S75	15	25	Fam-21_2007_	S75	75	69	21	2007	F

2171_1081_S76_E	2011 D	26.04.2011 S76	15.4	28.5	Fam-21_2007_S76	76	70	21	2007	F
2171_1081_S77_F	2011 D	26.04.2011 S77	17	38	Fam-13_2007_S77	77	71	13	2007	F
2171_1081_S78_G	2011 D	26.04.2011 S78	14.8	24	Fam-23_2007_S78	78	72	23	2007	F
2171_1081_S79_H	2011 D	26.04.2011 S79	16.9	35.5	Fam-13_2007_S79	79	73	13	2007	F
2171_1081_S80_A	2011 D	26.04.2011 S80	15.4	29.5	Fam-11_2007_S80	80	74	11	2007	F
2172_1081_S81_C	2011 D	26.04.2011 S81	14.7	25.5	Fam-28_2007_S81	81	75	28	2007	H
2172_1081_S82_D	2011 D	26.04.2011 S82	14	22.5	Fam-13_2007_S82	82	76	13	2007	F
2172_1081_S84_F	2011 D	26.04.2011 S84	15.1	27.5	Fam-28_2007_S84	84	77	28	2007	H
2172_1081_S85_G	2011 D	26.04.2011 S85	13.5	19	Fam-19_2007_S85	85	78	19	2007	F
2172_1081_S86_H	2011 D	26.04.2011 S86	15	26	Fam-23_2007_S86	86	79	23	2007	F
2172_1081_S87_A	2011 D	26.04.2011 S87	15.5	29	Fam-13_2007_S87	87	80	13	2007	F
2172_1081_S88_B	2011 D	26.04.2011 S88	16.9	37.5	Fam-28_2007_S88	88	81	28	2007	H
2172_1081_S89_C	2011 D	26.04.2011 S89	14.3	23	Fam-21_2007_S89	89	82	21	2007	F
2172_1081_S90_D	2011 D	26.04.2011 S90	14.1	22.5	Fam-19_2007_S90	90	83	19	2007	F

2172_1081_S91_E	2011	D	26.04.2011	S91	15.2	29.5	Fam-09_2007_	S91	91	84	9	2007	W
2172_1081_S92_F	2011	D	26.04.2011	S92	14.4	22	Fam-17_2007_	S92	92	85	17	2007	F
2172_1081_S93_G	2011	D	26.04.2011	S93	14.6	23.5	Fam-09_2007_	S93	93	86	9	2007	W
2172_1081_S94_H	2011	D	26.04.2011	S94	12.8	16.5	Fam-19_2007_	S94	94	87	19	2007	F
2172_1082_S95_E	2011	D	26.04.2011	S95	15.1	26.5	Fam-04_2007_	S95	95	88	4	2007	W
2172_1082_S96_C	2011	D	26.04.2011	S96	14.7	26.5	Fam-27_2007_	S96	96	89	27	2007	F
2172_1082_S97_D	2011	D	26.04.2011	S97	14	21	Fam-17_2007_	S97	97	90	17	2007	F
2172_1082_S98_E	2011	D	26.04.2011	S98	15.2	27	Fam-27_2007_	S98	98	91	27	2007	F
2172_1082_S99_F	2011	D	26.04.2011	S99	14.6	23	Fam-28_2007_	S99	99	92	28	2007	H
2172_1082_S100_	2011	D	26.04.2011	S100	13	17.5	Fam-03_2007_	S100	100	93	3	2007	W
2172_1082_S101_	2011	D	26.04.2011	S101	16.3	33.5	Fam-09_2007_	S101	101	94	9	2007	W
2172_1082_S103_	2011	D	27.04.2011	S103	14.7	25.5	Fam-09_2007_	S103	103	95	9	2007	W
2172_1082_S104_	2011	D	27.04.2011	S104	14.7	25	Fam-09_2007_	S104	104	96	9	2007	W
2172_1082_S105_	2011	D	27.04.2011	S105	15	25	Fam-13_2007_	S105	105	97	13	2007	F

2172_1082_S106_	2011	D	27.04.2011	S106	14.1	24	Fam-28_2007_	S106	106	98	28	2007	H
2172_1082_S108_	2011	D	27.04.2011	S108	14.8	24	Fam-30_2007_	S108	108	99	30	2007	H
2172_1082_S109_	2011	D	27.04.2011	S109	15.4	29.5	Fam-09_2007_	S109	109	100	9	2007	W
2172_1082_S110_	2011	D	27.04.2011	S110	14.5	22.5	Fam-09_2007_	S110	110	101	9	2007	W
2172_1082_S111_	2011	D	27.04.2011	S111	14.3	24.5	Fam-09_2007_	S111	111	102	9	2007	W
2172_1082_S114_	2011	D	28.04.2011	S114	14.6	23.5	Fam-13_2007_	S114	114	103	13	2007	F
2172_1082_S115_	2011	D	28.04.2011	S115	13.9	20	Fam-21_2007_	S115	115	104	21	2007	F
2172_1082_S116_	2011	D	28.04.2011	S116	14.7	24	Fam-13_2007_	S116	116	105	13	2007	F
2172_1082_S117_	2011	D	28.04.2011	S117	13.9	20.5	Fam-13_2007_	S117	117	106	13	2007	F
2172_1082_S118_	2011	D	28.04.2011	S118	16.7	34	Fam-19_2007_	S118	118	107	19	2007	F
2172_1082_S119_	2011	D	28.04.2011	S119	13.4	18	Fam-27_2007_	S119	119	108	27	2007	F
2172_1082_S120_	2011	D	28.04.2011	S120	13.7	20	Fam-13_2007_	S120	120	109	13	2007	F
2172_1082_S121_	2011	D	28.04.2011	S121	15.5	27.5	Fam-28_2007_	S121	121	110	28	2007	H
2172_1082_S122_	2011	D	28.04.2011	S122	14.5	24.5	Fam-13_2007_	S122	122	111	13	2007	F

2172_1082_S123_	2011	D	28.04.2011	S123	15.2	28	Fam-28_2007_S123	123	112	28	2007 H
2172_1082_S124_	2011	D	28.04.2011	S124	14.8	26	Fam-28_2007_S124	124	113	28	2007 H
2172_1082_S125_	2011	D	28.04.2011	S125	13.4	19.5	Fam-28_2007_S125	125	114	28	2007 H
2172_1082_S126_	2011	D	28.04.2011	S126	15.5	29.5	Fam-09_2007_S126	126	115	9	2007 W
2172_1082_S127_	2011	D	28.04.2011	S127	13.5	20	Fam-17_2007_S127	127	116	17	2007 F
2172_1082_S129_	2011	D	28.04.2011	S129	13.1	17.5	Fam-28_2007_S129	129	117	28	2007 H
2172_1082_S130_	2011	D	28.04.2011	S130	15.4	29	Fam-28_2007_S130	130	118	28	2007 H
2172_1082_S131_	2011	D	29.04.2011	S131	13.8	22.5	Fam-13_2007_S131	131	119	13	2007 F
2172_1082_S132_	2011	D	29.04.2011	S132	14.4	23	Fam-04_2007_S132	132	120	4	2007 W
2172_1082_S134_	2011	D	29.04.2011	S134	13.3	18	Fam-25_2007_S134	134	121	25	2007 F
2172_1082_S135_	2011	D	29.04.2011	S135	14.2	23	Fam-17_2007_S135	135	122	17	2007 F
2172_1082_S136_	2011	D	29.04.2011	S136	14.9	28	Fam-19_2007_S136	136	123	19	2007 F
2172_1082_S137_	2011	D	29.04.2011	S137	14.1	23	Fam-13_2007_S137	137	124	13	2007 F
2172_1082_S138_	2011	D	29.04.2011	S138	15.5	28.5	Fam-23_2007_S138	138	125	23	2007 F

2172_1082_S139_	2011	D	29.04.2011	S139	14.8	27	Fam-19_2007_	S139	139	126	19	2007	F
2172_1082_S140_	2011	D	29.04.2011	S140	14.4	22	Fam-17_2007_	S140	140	127	17	2007	F
2172_1082_S141_	2011	D	29.04.2011	S141	15.7	32	Fam-09_2007_	S141	141	128	9	2007	W
2172_1082_S142_	2011	D	29.04.2011	S142	15.2	28	Fam-09_2007_	S142	142	129	9	2007	W
2172_1082_S143_	2011	D	29.04.2011	S143	14.1	25	Fam-19_2007_	S143	143	130	19	2007	F
2172_1082_S144_	2011	D	29.04.2011	S144	15	28	Fam-13_2007_	S144	144	131	13	2007	F
2172_1082_S145_	2011	D	29.04.2011	S145	16	33	Fam-19_2007_	S145	145	132	19	2007	F
2172_1082_S146_	2011	D	29.04.2011	S146	15.1	27.5	Fam-23_2007_	S146	146	133	23	2007	F
2172_1082_S147_	2011	D	29.04.2011	S147	14.6	24	Fam-23_2007_	S147	147	134	23	2007	F
2172_1082_S148_	2011	D	29.04.2011	S148	14.1	22.5	Fam-13_2007_	S148	148	135	13	2007	F
2172_1082_S149_	2011	D	29.04.2011	S149	14	21.5	Fam-23_2007_	S149	149	136	23	2007	F
2172_1082_S150_	2011	D	02.05.2011	S150	16.2	34	Fam-19_2007_	S150	150	137	19	2007	F
2172_1082_S151_	2011	D	02.05.2011	S151	14.5	24	Fam-04_2007_	S151	151	138	4	2007	W
2172_1082_S152_	2011	D	02.05.2011	S152	14.1	22.5	Fam-19_2007_	S152	152	139	19	2007	F

2172_1082_S153_	2011	D	02.05.2011	S153	14.5	25	Fam-19_2007_S153	153	140	19	2007	F
2172_1082_S154_	2011	D	02.05.2011	S154	13.2	19	Fam-10_2007_S154	154	141	10	2007	W
2172_1082_S155_	2011	D	02.05.2011	S155	13.4	20	Fam-13_2007_S155	155	142	13	2007	F
2172_1082_S156_	2011	D	02.05.2011	S156	15.2	27.5	Fam-27_2007_S156	156	143	27	2007	F
2172_1082_S157_	2011	D	02.05.2011	S157	14.6	23.5	Fam-21_2007_S157	157	144	21	2007	F
2172_1082_S158_	2011	D	02.05.2011	S158	14.5	24.5	Fam-27_2007_S158	158	145	27	2007	F
2172_1082_S159_	2011	D	02.05.2011	S159	15.1	26	Fam-23_2007_S159	159	146	23	2007	F
2172_1082_S160_	2011	D	02.05.2011	S160	13.5	20.5	Fam-23_2007_S160	160	147	23	2007	F
2173_1082_S161_	2011	D	02.05.2011	S161	14.4	22.5	Fam-28_2007_S161	161	148	28	2007	H
2173_1082_S162_	2011	D	02.05.2011	S162	14	22	Fam-19_2007_S162	162	149	19	2007	F
2173_1082_S163_	2011	D	02.05.2011	S163	14.3	23.5	Fam-13_2007_S163	163	150	13	2007	F
2173_1082_S164_	2011	D	02.05.2011	S164	15.2	27	Fam-11_2007_S164	164	151	11	2007	F
2173_1082_S165_	2011	D	02.05.2011	S165	14.2	22	Fam-03_2007_S165	165	152	3	2007	W
2173_1082_S166_	2011	D	02.05.2011	S166	16	34	Fam-28_2007_S166	166	153	28	2007	H

2173_1082_S167_	2011	D	02.05.2011	S167	14.5	23.5	Fam-21_2007_	S167	167	154	21	2007	F
2173_1082_S168_	2011	D	02.05.2011	S168	16.5	35.5	Fam-03_2007_	S168	168	155	3	2007	W
2173_1082_S170_	2011	D	02.05.2011	S170	15.8	30	Fam-23_2007_	S170	170	156	23	2007	F
2173_1082_S171_	2011	D	02.05.2011	S171	14.8	26.5	Fam-27_2007_	S171	171	157	27	2007	F
2173_1082_S172_	2011	D	02.05.2011	S172	14	20.5	Fam-23_2007_	S172	172	158	23	2007	F
2173_1082_S173_	2011	D	02.05.2011	S173	14.6	24	Fam-28_2007_	S173	173	159	28	2007	H
2173_1082_S174_	2011	D	02.05.2011	S174	14	23	Fam-04_2007_	S174	174	160	4	2007	W
2173_1082_S175_	2011	D	02.05.2011	S175	17	37.5	Fam-23_2007_	S175	175	161	23	2007	F
2173_1082_S176_	2011	D	02.05.2011	S176	13.8	20	Fam-04_2007_	S176	176	162	4	2007	W
2173_1082_S177_	2011	D	02.05.2011	S177	15	27	Fam-17_2007_	S177	177	163	17	2007	F
2173_1082_S178_	2011	D	02.05.2011	S178	14.5	26	Fam-11_2007_	S178	178	164	11	2007	F
2173_1082_S179_	2011	D	02.05.2011	S179	13.3	19.5	Fam-09_2007_	S179	179	165	9	2007	W
2173_1082_S180_	2011	D	02.05.2011	S180	15	28	Fam-28_2007_	S180	180	166	28	2007	H
2173_1082_S181_	2011	D	02.05.2011	S181	15.5	30.5	Fam-13_2007_	S181	181	167	13	2007	F

2173_1082_S183_	2011	D	02.05.2011	S183	14.6	26	Fam-19_2007_	S183	183	168	19	2007 F
2173_1082_S184_	2011	D	02.05.2011	S184	15.5	27	Fam-19_2007_	S184	184	169	19	2007 F
2173_1082_S185_	2011	D	02.05.2011	S185	14	24	Fam-19_2007_	S185	185	170	19	2007 F
2173_1082_S186_	2011	D	02.05.2011	S186	14	21	Fam-23_2007_	S186	186	171	23	2007 F
2173_1082_S187_	2011	D	02.05.2011	S187	13.6	21.5	Fam-04_2007_	S187	187	172	4	2007 W
2173_1082_S188_	2011	D	02.05.2011	S188	15.7	30.5	Fam-04_2007_	S188	188	173	4	2007 W
2173_1083_S189_	2011	D	02.05.2011	S189	14.1	21.5	Fam-09_2007_	S189	189	174	9	2007 W
2173_1083_S190_	2011	D	02.05.2011	S190	14.2	22.5	Fam-13_2007_	S190	190	175	13	2007 F
2173_1083_S191_	2011	D	02.05.2011	S191	15.4	28	Fam-27_2007_	S191	191	176	27	2007 F
2173_1083_S192_	2011	D	02.05.2011	S192	14.5	24.5	Fam-23_2007_	S192	192	177	23	2007 F
2173_1083_S193_	2011	D	02.05.2011	S193	16.8	37.5	Fam-17_2007_	S193	193	178	17	2007 F
2173_1083_S194_	2011	D	02.05.2011	S194	14	20.5	Fam-28_2007_	S194	194	179	28	2007 H
2173_1083_S196_	2011	D	02.05.2011	S196	15	26	Fam-04_2007_	S196	196	180	4	2007 W
2173_1083_S197_	2011	D	02.05.2011	S197	14.3	22.5	Fam-27_2007_	S197	197	181	27	2007 F

2173_1083_S198_	2011	D	02.05.2011	S198	14.5	24.5	Fam-28_2007_	S198	198	182	28	2007 H
2173_1083_S199_	2011	D	02.05.2011	S199	14	23	Fam-17_2007_	S199	199	183	17	2007 F
2173_1083_S200_	2011	D	02.05.2011	S200	14.2	22	Fam-27_2007_	S200	200	184	27	2007 F
2173_1083_S201_	2011	T	02.05.2011	S201	15.4	28	Family-27_200	S201	201	185	27	2007 F
2173_1083_S202_	2011	D	02.05.2011	S202	16.3	33.5	Fam-11_2007_	S202	202	186	11	2007 F
2173_1083_S203_	2011	D	02.05.2011	S203	14.4	23	Fam-23_2007_	S203	203	187	23	2007 F
2173_1083_S204_	2011	D	02.05.2011	S204	14.5	24.5	Fam-27_2007_	S204	204	188	27	2007 F
2173_1083_S205_	2011	D	02.05.2011	S205	14.5	23	Fam-28_2007_	S205	205	189	28	2007 H
2173_1083_S206_	2011	D	02.05.2011	S206	15.5	30.5	Fam-27_2007_	S206	206	190	27	2007 F
2173_1083_S207_	2011	D	02.05.2011	S207	13.4	20.5	Fam-09_2007_	S207	207	191	9	2007 W
2173_1083_S208_	2011	D	02.05.2011	S208	15	28.5	Fam-28_2007_	S208	208	192	28	2007 H
2173_1083_S209_	2011	D	02.05.2011	S209	16	32.5	Fam-19_2007_	S209	209	193	19	2007 F
2173_1083_S210_	2011	D	02.05.2011	S210	14.4	23.5	Fam-27_2007_	S210	210	194	27	2007 F
2173_1083_S212_	2011	D	02.05.2011	S212	14.6	27.5	Fam-27_2007_	S212	212	195	27	2007 F

2173_1083_S213_	2011 D	02.05.2011	S213	16	34.5	Fam-21_2007_S213	213	196	21	2007 F
2173_1083_S214_	2011 D	02.05.2011	S214	15.3	28	Fam-23_2007_S214	214	197	23	2007 F
2173_1083_S215_	2011 D	02.05.2011	S215	14.5	22.5	Fam-11_2007_S215	215	198	11	2007 F
2173_1083_S216_	2011 D	03.05.2011	S216	14.1	22	Fam-23_2007_S216	216	199	23	2007 F
2173_1083_S217_	2011 D	03.05.2011	S217	14.5	24	Fam-13_2007_S217	217	200	13	2007 F
2173_1083_S219_	2011 D	03.05.2011	S219	14.4	22.5	Fam-17_2007_S219	219	201	17	2007 F
2173_1083_S220_	2011 D	03.05.2011	S220	14.4	24	Fam-19_2007_S220	220	202	19	2007 F
2173_1083_S221_	2011 D	03.05.2011	S221	14.9	25.5	Fam-23_2007_S221	221	203	23	2007 F
2173_1083_S222_	2011 D	03.05.2011	S222	16	31.5	Fam-28_2007_S222	222	204	28	2007 H
2173_1083_S223_	2011 D	03.05.2011	S223	14.5	24	Fam-11_2007_S223	223	205	11	2007 F
2173_1083_S225_	2011 D	03.05.2011	S225	14.8	26	Fam-05_2007_S225	225	206	5	2007 W
2173_1083_S226_	2011 D	03.05.2011	S226	14.9	26.5	Fam-21_2007_S226	226	207	21	2007 F
2173_1083_S227_	2011 D	03.05.2011	S227	13.9	21	Fam-11_2007_S227	227	208	11	2007 F
2173_1083_S228_	2011 D	03.05.2011	S228	14.7	24.5	Fam-11_2007_S228	228	209	11	2007 F

2173_1083_S229_	2011	D	03.05.2011	S229	15.3	28.5	Fam-17_2007_S229	229	210	17	2007	F
2173_1083_S230_	2011	D	03.05.2011	S230	14.3	24	Fam-19_2007_S230	230	211	19	2007	F
2173_1083_S231_	2011	D	03.05.2011	S231	14.3	24.5	Fam-09_2007_S231	231	212	9	2007	W
2173_1083_S232_	2011	D	03.05.2011	S232	13.9	20.5	Fam-19_2007_S232	232	213	19	2007	F
2173_1083_S233_	2011	D	03.05.2011	S233	14.9	27	Fam-28_2007_S233	233	214	28	2007	H
2173_1083_S234_	2011	D	03.05.2011	S234	13.9	21.5	Fam-09_2007_S234	234	215	9	2007	W
2173_1083_S235_	2011	D	03.05.2011	S235	14.5	24	Fam-17_2007_S235	235	216	17	2007	F
2173_1083_S236_	2011	D	04.05.2011	S236	15	24.5	Fam-09_2007_S236	236	217	9	2007	W
2173_1083_S237_	2011	D	04.05.2011	S237	15.2	28.5	Fam-23_2007_S237	237	218	23	2007	F
2173_1083_S238_	2011	D	04.05.2011	S238	14.9	26.5	Fam-19_2007_S238	238	219	19	2007	F
2173_1083_S239_	2011	D	04.05.2011	S239	14.5	22	Fam-04_2007_S239	239	220	4	2007	W
2173_1083_S240_	2011	D	04.05.2011	S240	16	32	Fam-27_2007_S240	240	221	27	2007	F
2174_1083_S241_	2011	D	04.05.2011	S241	17.3	41	Fam-27_2007_S241	241	222	27	2007	F
2174_1083_S242_	2011	D	04.05.2011	S242	16.7	35.5	Fam-21_2007_S242	242	223	21	2007	F

2174_1083_S243_	2011	D	04.05.2011	S243	14.2	23	Fam-09_2007_	S243	243	224	9	2007	W
2174_1083_S244_	2011	D	04.05.2011	S244	12.6	16.5	Fam-27_2007_	S244	244	225	27	2007	F
2174_1083_S245_	2011	D	05.05.2011	S245	14.2	22	Fam-04_2007_	S245	245	226	4	2007	W
2174_1083_S246_	2011	D	05.05.2011	S246	14	22	Fam-28_2007_	S246	246	227	28	2007	H
2174_1083_S247_	2011	D	05.05.2011	S247	15.6	32	Fam-19_2007_	S247	247	228	19	2007	F
2174_1083_S248_	2011	D	05.05.2011	S248	15.9	31	Fam-11_2007_	S248	248	229	11	2007	F
2174_1083_S249_	2011	D	05.05.2011	S249	14.6	25	Fam-19_2007_	S249	249	230	19	2007	F
2174_1083_S250_	2011	D	05.05.2011	S250	15.3	30	Fam-13_2007_	S250	250	231	13	2007	F
2174_1083_S251_	2011	D	05.05.2011	S251	13.5	18	Fam-09_2007_	S251	251	232	9	2007	W
2174_1083_S252_	2011	D	06.05.2011	S252	16.9	38	Fam-19_2007_	S252	252	233	19	2007	F
2174_1083_S253_	2011	D	06.05.2011	S253	14.4	24	Fam-27_2007_	S253	253	234	27	2007	F
2174_1083_S254_	2011	D	06.05.2011	S254	15.7	31.5	Fam-27_2007_	S254	254	235	27	2007	F
2174_1083_S255_	2011	D	06.05.2011	S255	15.6	30	Fam-09_2007_	S255	255	236	9	2007	W
2174_1083_S256_	2011	D	06.05.2011	S256	14.1	22.5	Fam-09_2007_	S256	256	237	9	2007	W

2174_1083_S257_	2011	D	06.05.2011	S257	13.9	21	Fam-10_2007_ S257	257	238	10	2007	W
2174_1083_S258_	2011	D	06.05.2011	S258	15.5	32	Fam-04_2007_ S258	258	239	4	2007	W
2174_1083_S259_	2011	D	06.05.2011	S259	13.6	20.5	Fam-27_2007_ S259	259	240	27	2007	F
2174_1083_S260_	2011	D	06.05.2011	S260	13.7	22	Fam-19_2007_ S260	260	241	19	2007	F
2174_1083_S261_	2011	D	06.05.2011	S261	15.4	31	Fam-11_2007_ S261	261	242	11	2007	F
2174_1083_S262_	2011	D	06.05.2011	S262	15.5	29.5	Fam-17_2007_ S262	262	243	17	2007	F
2174_1083_S263_	2011	D	06.05.2011	S263	14.5	24	Fam-19_2007_ S263	263	244	19	2007	F
2174_1083_S264_	2011	D	06.05.2011	S264	14.4	24	Fam-09_2007_ S264	264	245	9	2007	W
2174_1083_S265_	2011	D	06.05.2011	S265	17.2	39	Fam-23_2007_ S265	265	246	23	2007	F
2174_1083_S266_	2011	D	06.05.2011	S266	15	27.5	Fam-19_2007_ S266	266	247	19	2007	F
2174_1083_S267_	2011	D	06.05.2011	S267	15.3	29	Fam-09_2007_ S267	267	248	9	2007	W
2174_1083_S269_	2011	D	06.05.2011	S269	14.2	24	Fam-11_2007_ S269	269	249	11	2007	F
2174_1083_S270_	2011	D	06.05.2011	S270	15.1	28	Fam-19_2007_ S270	270	250	19	2007	F
2174_1083_S272_	2011	D	06.05.2011	S272	16.2	35	Fam-09_2007_ S272	272	251	9	2007	W

2174_1083_S273_	2011	D	06.05.2011	S273	13	19	Fam-23_2007_	S273	273	252	23	2007	F
2174_1083_S274_	2011	D	06.05.2011	S274	15.5	30	Fam-09_2007_	S274	274	253	9	2007	W
2174_1083_S275_	2011	D	06.05.2011	S275	15.3	29	Fam-30_2007_	S275	275	254	30	2007	H
2174_1083_S276_	2011	D	06.05.2011	S276	16.5	34	Fam-19_2007_	S276	276	255	19	2007	F
2174_1083_S277_	2011	D	06.05.2011	S277	15.5	31	Fam-30_2007_	S277	277	256	30	2007	H
2174_1083_S278_	2011	D	06.05.2011	S278	15.1	29	Fam-17_2007_	S278	278	257	17	2007	F
2174_1083_S279_	2011	D	06.05.2011	S279	14.6	25	Fam-03_2007_	S279	279	258	3	2007	W
2174_1083_S280_	2011	D	09.05.2011	S280	16.2	32.5	Fam-28_2007_	S280	280	259	28	2007	H
2174_1083_S281_	2011	D	09.05.2011	S281	14.5	23.5	Fam-21_2007_	S281	281	260	21	2007	F
2174_1083_S282_	2011	D	09.05.2011	S282	16	31.5	Fam-23_2007_	S282	282	261	23	2007	F
2174_1084_S283_	2011	D	09.05.2011	S283	14.5	25	Fam-11_2007_	S283	283	262	11	2007	F
2174_1084_S285_	2011	D	09.05.2011	S285	14.7	25.5	Fam-11_2007_	S285	285	263	11	2007	F
2174_1084_S286_	2011	D	09.05.2011	S286	12.8	16.5	Fam-28_2007_	S286	286	264	28	2007	H
2174_1084_S287_	2011	D	09.05.2011	S287	14.5	23.5	Fam-19_2007_	S287	287	265	19	2007	F

2174_1084_S288_	2011	D	09.05.2011	S288	15.7	31	Fam-13_2007_	S288	288	266	13	2007	F
2174_1084_S289_	2011	D	09.05.2011	S289	14.7	25.5	Fam-11_2007_	S289	289	267	11	2007	F
2174_1084_S290_	2011	D	09.05.2011	S290	13.6	21	Fam-03_2007_	S290	290	268	3	2007	W
2174_1084_S291_	2011	D	09.05.2011	S291	13	16.5	Fam-19_2007_	S291	291	269	19	2007	F
2174_1084_S292_	2011	D	09.05.2011	S292	16.9	40.5	Fam-10_2007_	S292	292	270	10	2007	W
2174_1084_S293_	2011	D	09.05.2011	S293	14.5	23.5	Fam-27_2007_	S293	293	271	27	2007	F
2174_1084_S294_	2011	D	09.05.2011	S294	18.2	49	Fam-19_2007_	S294	294	272	19	2007	F
2174_1084_S295_	2011	D	09.05.2011	S295	17.7	40.5	Fam-23_2007_	S295	295	273	23	2007	F
2174_1084_S296_	2011	D	09.05.2011	S296	15.5	27.5	Fam-28_2007_	S296	296	274	28	2007	H
2174_1084_S297_	2011	D	09.05.2011	S297	15.6	31	Fam-13_2007_	S297	297	275	13	2007	F
2174_1084_S298_	2011	D	09.05.2011	S298	14.5	24.5	Fam-23_2007_	S298	298	276	23	2007	F
2174_1084_S299_	2011	D	09.05.2011	S299	13.6	20	Fam-30_2007_	S299	299	277	30	2007	H
2174_1084_S300_	2011	D	09.05.2011	S300	14.2	22.5	Fam-23_2007_	S300	300	278	23	2007	F
2174_1084_S301_	2011	D	09.05.2011	S301	14.5	26	Fam-19_2007_	S301	301	279	19	2007	F

2174_1084_S302_	2011 D	09.05.2011	S302	14.7	25	Fam-11_2007_S302	302	280	11	2007	F
2174_1084_S303_	2011 D	09.05.2011	S303	13.3	19	Fam-09_2007_S303	303	281	9	2007	W
2174_1084_S304_	2011 D	09.05.2011	S304	14.3	22	Fam-13_2007_S304	304	282	13	2007	F
2174_1084_S305_	2011 D	09.05.2011	S305	14.2	23	Fam-23_2007_S305	305	283	23	2007	F
2174_1084_S306_	2011 D	09.05.2011	S306	15	26	Fam-09_2007_S306	306	284	9	2007	W
2174_1084_S307_	2011 D	09.05.2011	S307	17	40	Fam-10_2007_S307	307	285	10	2007	W
2174_1084_S308_	2011 D	09.05.2011	S308	14.7	24	Fam-17_2007_S308	308	286	17	2007	F
2174_1084_S309_	2011 D	09.05.2011	S309	14.2	24	Fam-09_2007_S309	309	287	9	2007	W
2174_1084_S310_	2011 D	09.05.2011	S310	15.1	27	Fam-23_2007_S310	310	288	23	2007	F
2174_1084_S311_	2011 D	09.05.2011	S311	15.6	30.5	Fam-17_2007_S311	311	289	17	2007	F
2174_1084_S312_	2011 D	09.05.2011	S312	14.6	26	Fam-28_2007_S312	312	290	28	2007	H
2174_1084_S313_	2011 D	09.05.2011	S313	14.5	25	Fam-11_2007_S313	313	291	11	2007	F
2174_1084_S314_	2011 D	09.05.2011	S314	15.2	28	Fam-09_2007_S314	314	292	9	2007	W
2174_1084_S315_	2011 D	09.05.2011	S315	15.2	27.5	Fam-17_2007_S315	315	293	17	2007	F

2174_1084_S316_	2011	D	09.05.2011	S316	14	21.5	Fam-09_2007_	S316	316	294	9	2007	W
2174_1084_S317_	2011	D	09.05.2011	S317	14.2	23	Fam-09_2007_	S317	317	295	9	2007	W
2174_1084_S318_	2011	D	09.05.2011	S318	16	33.5	Fam-28_2007_	S318	318	296	28	2007	H
2174_1084_S319_	2011	D	09.05.2011	S319	13.9	20	Fam-03_2007_	S319	319	297	3	2007	W
2174_1084_S320_	2011	D	09.05.2011	S320	13.5	19.5	Fam-27_2007_	S320	320	298	27	2007	F
2175_1084_S321_	2011	D	09.05.2011	S321	13.2	18.5	Fam-09_2007_	S321	321	299	9	2007	W
2175_1084_S322_	2011	D	09.05.2011	S322	14	22	Fam-28_2007_	S322	322	300	28	2007	H
2175_1084_S324_	2011	D	09.05.2011	S324	16.5	37	Fam-25_2007_	S324	324	301	25	2007	F
2175_1084_S325_	2011	D	09.05.2011	S325	15.3	30	Fam-21_2007_	S325	325	302	21	2007	F
2175_1084_S326_	2011	D	09.05.2011	S326	14.5	24.5	Fam-11_2007_	S326	326	303	11	2007	F
2175_1084_S327_	2011	D	09.05.2011	S327	14	21	Fam-23_2007_	S327	327	304	23	2007	F
2175_1084_S328_	2011	D	09.05.2011	S328	17	38.5	Fam-19_2007_	S328	328	305	19	2007	F
2175_1084_S329_	2011	D	09.05.2011	S329	16	31	Fam-17_2007_	S329	329	306	17	2007	F
2175_1084_S330_	2011	D	09.05.2011	S330	16.3	34	Fam-03_2007_	S330	330	307	3	2007	W

2175_1084_S332_	2011	D	09.05.2011	S332	14.6	26	Fam-11_2007_	S332	332	308	11	2007	F
2175_1084_S333_	2011	D	09.05.2011	S333	14.5	24.5	Fam-13_2007_	S333	333	309	13	2007	F
2175_1084_S334_	2011	D	09.05.2011	S334	14.2	22.5	Fam-17_2007_	S334	334	310	17	2007	F
2175_1084_S335_	2011	D	09.05.2011	S335	14	21.5	Fam-27_2007_	S335	335	311	27	2007	F
2175_1084_S336_	2011	D	09.05.2011	S336	15.5	31	Fam-23_2007_	S336	336	312	23	2007	F
2175_1084_S337_	2011	D	09.05.2011	S337	15	25	Fam-19_2007_	S337	337	313	19	2007	F
2175_1084_S338_	2011	D	09.05.2011	S338	14.5	24	Fam-23_2007_	S338	338	314	23	2007	F
2175_1084_S339_	2011	D	09.05.2011	S339	15	27	Fam-28_2007_	S339	339	315	28	2007	H
2175_1084_S340_	2011	D	09.05.2011	S340	14.1	22	Fam-04_2007_	S340	340	316	4	2007	W
2175_1084_S341_	2011	D	09.05.2011	S341	14.8	26.5	Fam-09_2007_	S341	341	317	9	2007	W
2175_1084_S342_	2011	D	09.05.2011	S342	14.4	24	Fam-28_2007_	S342	342	318	28	2007	H
2175_1084_S343_	2011	D	09.05.2011	S343	14	22	Fam-13_2007_	S343	343	319	13	2007	F
2175_1084_S344_	2011	D	09.05.2011	S344	15.5	32	Fam-11_2007_	S344	344	320	11	2007	F
2175_1084_S345_	2011	D	09.05.2011	S345	15	26	Fam-23_2007_	S345	345	321	23	2007	F

2175_1084_S346_	2011	D	09.05.2011	S346	15.5	31	Fam-03_2007_	S346	346	322	3	2007	W
2175_1084_S347_	2011	D	09.05.2011	S347	13	17.5	Fam-03_2007_	S347	347	323	3	2007	W
2175_1084_S348_	2011	D	09.05.2011	S348	15	24.5	Fam-17_2007_	S348	348	324	17	2007	F
2175_1084_S349_	2011	D	09.05.2011	S349	13.9	22	Fam-04_2007_	S349	349	325	4	2007	W
2175_1084_S350_	2011	D	09.05.2011	S350	14.4	23	Fam-28_2007_	S350	350	326	28	2007	H
2175_1084_S351_	2011	D	09.05.2011	S351	15.1	28.5	Fam-28_2007_	S351	351	327	28	2007	H
2175_1084_S352_	2011	D	09.05.2011	S352	15.3	28.5	Fam-17_2007_	S352	352	328	17	2007	F
2175_1084_S353_	2011	D	09.05.2011	S353	14.5	23	Fam-11_2007_	S353	353	329	11	2007	F
2175_1084_S354_	2011	D	09.05.2011	S354	16.2	35	Fam-09_2007_	S354	354	330	9	2007	W
2175_1084_S355_	2011	D	09.05.2011	S355	15.3	29	Fam-23_2007_	S355	355	331	23	2007	F
2175_1084_S356_	2011	D	09.05.2011	S356	15	27.5	Fam-09_2007_	S356	356	332	9	2007	W
2175_1084_S359_	2011	D	09.05.2011	S359	13.5	19.5	Fam-28_2007_	S359	359	333	28	2007	H
2175_1084_S360_	2011	D	09.05.2011	S360	13.5	20	Fam-28_2007_	S360	360	334	28	2007	H
2175_1084_S361_	2011	D	09.05.2011	S361	14.1	22	Fam-19_2007_	S361	361	335	19	2007	F

2175_1084_S362_	2011	D	09.05.2011	S362	15	26	Fam-03_2007_ S362	362	336	3	2007	W
2175_1084_S363_	2011	D	09.05.2011	S363	17.2	37.5	Fam-21_2007_ S363	363	337	21	2007	F
2175_1084_S364_	2011	D	09.05.2011	S364	14	21.5	Fam-04_2007_ S364	364	338	4	2007	W
2175_1084_S365_	2011	D	09.05.2011	S365	14.1	23.5	Fam-27_2007_ S365	365	339	27	2007	F
2175_1084_S367_	2011	D	09.05.2011	S367	15.5	28.5	Fam-13_2007_ S367	367	340	13	2007	F
2175_1084_S368_	2011	D	09.05.2011	S368	14.4	22.5	Fam-28_2007_ S368	368	341	28	2007	H
2175_1084_S369_	2011	D	09.05.2011	S369	14.5	23.5	Fam-11_2007_ S369	369	342	11	2007	F
2175_1084_S370_	2011	D	09.05.2011	S370	14.8	24.5	Fam-09_2007_ S370	370	343	9	2007	W
2175_1084_S371_	2011	D	09.05.2011	S371	14.1	22	Fam-28_2007_ S371	371	344	28	2007	H
2175_1084_S372_	2011	D	09.05.2011	S372	15.5	28.5	Fam-17_2007_ S372	372	345	17	2007	F
2175_1084_S373_	2011	D	09.05.2011	S373	15	25	Fam-23_2007_ S373	373	346	23	2007	F
2175_1084_S374_	2011	D	09.05.2011	S374	14.6	26.5	Fam-23_2007_ S374	374	347	23	2007	F
2175_1084_S375_	2011	D	09.05.2011	S375	14.3	23.5	Fam-23_2007_ S375	375	348	23	2007	F
2175_1084_S376_	2011	D	09.05.2011	S376	15.1	27	Fam-23_2007_ S376	376	349	23	2007	F

2175_1085_S377_	2011	D	09.05.2011	S377	14.8	27	Fam-17_2007_S377	377	350	17	2007	F
2175_1085_S378_	2011	D	09.05.2011	S378	15.1	28.5	Fam-09_2007_S378	378	351	9	2007	W
2175_1085_S379_	2011	D	09.05.2011	S379	15.2	28	Fam-11_2007_S379	379	352	11	2007	F
2175_1085_S380_	2011	D	09.05.2011	S380	13.4	18.5	Fam-23_2007_S380	380	353	23	2007	F
2175_1085_S381_	2011	D	09.05.2011	S381	15	25	Fam-17_2007_S381	381	354	17	2007	F
2175_1085_S382_	2011	D	09.05.2011	S382	14.2	23.5	Fam-28_2007_S382	382	355	28	2007	H
2175_1085_S383_	2011	D	09.05.2011	S383	14.3	23	Fam-11_2007_S383	383	356	11	2007	F
2175_1085_S384_	2011	D	09.05.2011	S384	14.5	23	Fam-09_2007_S384	384	357	9	2007	W
2175_1085_S385_	2011	D	09.05.2011	S385	14.2	21.5	Fam-17_2007_S385	385	358	17	2007	F
2175_1085_S386_	2011	D	09.05.2011	S386	16.3	36	Fam-09_2007_S386	386	359	9	2007	W
2175_1085_S387_	2011	D	09.05.2011	S387	14.1	22.5	Fam-13_2007_S387	387	360	13	2007	F
2175_1085_S388_	2011	D	09.05.2011	S388	15	25.5	Fam-27_2007_S388	388	361	27	2007	F
2175_1085_S390_	2011	D	09.05.2011	S390	15	25.5	Fam-09_2007_S390	390	362	9	2007	W
2175_1085_S391_	2011	D	09.05.2011	S391	15	25	Fam-23_2007_S391	391	363	23	2007	F

2175_1085_S393_	2011	D	09.05.2011	S393	15.2	27	Fam-28_2007_	S393	393	364	28	2007	H
2175_1085_S394_	2011	D	09.05.2011	S394	15.5	27.5	Fam-09_2007_	S394	394	365	9	2007	W
2175_1085_S395_	2011	D	09.05.2011	S395	14.7	23	Fam-23_2007_	S395	395	366	23	2007	F
2175_1085_S396_	2011	D	09.05.2011	S396	16.1	31.5	Fam-13_2007_	S396	396	367	13	2007	F
2175_1085_S397_	2011	D	09.05.2011	S397	15.2	26	Fam-23_2007_	S397	397	368	23	2007	F
2175_1085_S398_	2011	D	09.05.2011	S398	13.6	20.5	Fam-21_2007_	S398	398	369	21	2007	F
2175_1085_S399_	2011	D	09.05.2011	S399	13.5	20	Fam-27_2007_	S399	399	370	27	2007	F
2176_1085_S401_	2011	D	09.05.2011	S401	14.1	21.5	Fam-21_2007_	S401	401	371	21	2007	F
2176_1085_S402_	2011	D	09.05.2011	S402	16.1	34	Fam-21_2007_	S402	402	372	21	2007	F
2176_1085_S403_	2011	D	09.05.2011	S403	14.1	21.5	Fam-28_2007_	S403	403	373	28	2007	H
2176_1085_S404_	2011	D	09.05.2011	S404	15	25.5	Fam-17_2007_	S404	404	374	17	2007	F
2176_1085_S405_	2011	D	09.05.2011	S405	13.8	20	Fam-09_2007_	S405	405	375	9	2007	W
2176_1085_S406_	2011	D	09.05.2011	S406	16.5	34	Fam-09_2007_	S406	406	376	9	2007	W
2176_1085_S407_	2011	D	09.05.2011	S407	15	26.5	Fam-28_2007_	S407	407	377	28	2007	H

2176_1085_S408_	2011	D	09.05.2011	S408	16	32.5	Fam-11_2007_	S408	408	378	11	2007	F
2176_1085_S409_	2011	D	09.05.2011	S409	13.2	17.5	Fam-11_2007_	S409	409	379	11	2007	F
2176_1085_S410_	2011	D	09.05.2011	S410	14.2	21.5	Fam-17_2007_	S410	410	380	17	2007	F
2176_1085_S411_	2011	D	09.05.2011	S411	17.2	38.5	Fam-03_2007_	S411	411	381	3	2007	W
2176_1085_S412_	2011	D	09.05.2011	S412	14.2	23	Fam-28_2007_	S412	412	382	28	2007	H
2176_1085_S413_	2011	D	09.05.2011	S413	15.2	26	Fam-13_2007_	S413	413	383	13	2007	F
2176_1085_S414_	2011	D	09.05.2011	S414	15.2	25	Fam-03_2007_	S414	414	384	3	2007	W
2176_1085_S415_	2011	D	09.05.2011	S415	14.2	21	Fam-17_2007_	S415	415	385	17	2007	F
2176_1085_S417_	2011	D	09.05.2011	S417	15.2	27.5	Fam-21_2007_	S417	417	386	21	2007	F
2176_1085_S418_	2011	D	09.05.2011	S418	14.2	21	Fam-03_2007_	S418	418	387	3	2007	W
2176_1085_S419_	2011	D	09.05.2011	S419	14.9	27.5	Fam-13_2007_	S419	419	388	13	2007	F
2176_1085_S420_	2011	D	09.05.2011	S420	15.2	27	Fam-03_2007_	S420	420	389	3	2007	W
2176_1085_S421_	2011	D	09.05.2011	S421	15.6	31.5	Fam-05_2007_	S421	421	390	5	2007	W
2176_1085_S422_	2011	D	09.05.2011	S422	14.8	25.5	Fam-19_2007_	S422	422	391	19	2007	F

2176_1085_S423_	2011	D	09.05.2011	S423	17.1	40.5	Fam-09_2007_S423	423	392	9	2007	W
2176_1085_S424_	2011	D	09.05.2011	S424	13.6	19	Fam-06_2007_S424	424	393	6	2007	W
2176_1085_S425_	2011	D	09.05.2011	S425	15.3	28.5	Fam-04_2007_S425	425	394	4	2007	W
2176_1085_S426_	2011	D	09.05.2011	S426	14.8	24.5	Fam-27_2007_S426	426	395	27	2007	F
2176_1085_S427_	2011	D	09.05.2011	S427	13.5	20.5	Fam-10_2007_S427	427	396	10	2007	W
2176_1085_S428_	2011	D	09.05.2011	S428	16	31.5	Fam-03_2007_S428	428	397	3	2007	W
2176_1085_S429_	2011	D	09.05.2011	S429	14.8	24.5	Fam-23_2007_S429	429	398	23	2007	F
2176_1085_S430_	2011	D	09.05.2011	S430	15.3	29.5	Fam-10_2007_S430	430	399	10	2007	W
2176_1085_S431_	2011	D	09.05.2011	S431	14.4	23.5	Fam-21_2007_S431	431	400	21	2007	F
2176_1085_S432_	2011	D	09.05.2011	S432	14.5	24.5	Fam-11_2007_S432	432	401	11	2007	F
2176_1085_S433_	2011	D	09.05.2011	S433	14.9	25	Fam-17_2007_S433	433	402	17	2007	F
2176_1085_S434_	2011	D	09.05.2011	S434	15.3	29.5	Fam-09_2007_S434	434	403	9	2007	W
2176_1085_S435_	2011	D	09.05.2011	S435	14.9	27	Fam-28_2007_S435	435	404	28	2007	H
2176_1085_S436_	2011	D	09.05.2011	S436	15.1	30.5	Fam-27_2007_S436	436	405	27	2007	F

2176_1085_S439_	2011	D	09.05.2011	S439	14.3	24.5	Fam-27_2007_S439	439	406	27	2007	F
2176_1085_S440_	2011	D	09.05.2011	S440	16.3	35	Fam-25_2007_S440	440	407	25	2007	F
2176_1085_S441_	2011	D	09.05.2011	S441	15.8	31.5	Fam-11_2007_S441	441	408	11	2007	F
2176_1085_S442_	2011	D	09.05.2011	S442	14.6	24.5	Fam-09_2007_S442	442	409	9	2007	W
2176_1085_S443_	2011	D	09.05.2011	S443	13.9	21.5	Fam-19_2007_S443	443	410	19	2007	F
2176_1085_S444_	2011	D	09.05.2011	S444	14.5	25	Fam-28_2007_S444	444	411	28	2007	H
2176_1085_S445_	2011	D	09.05.2011	S445	14.5	23	Fam-23_2007_S445	445	412	23	2007	F
2176_1085_S446_	2011	D	09.05.2011	S446	14	22	Fam-09_2007_S446	446	413	9	2007	W
2176_1085_S447_	2011	D	09.05.2011	S447	13.4	19.5	Fam-06_2007_S447	447	414	6	2007	W
2176_1085_S448_	2011	D	09.05.2011	S448	14.4	24	Fam-17_2007_S448	448	415	17	2007	F
2176_1085_S450_	2011	D	09.05.2011	S450	13.5	20	Fam-23_2007_S450	450	416	23	2007	F
2176_1085_S451_	2011	D	09.05.2011	S451	14.9	25	Fam-19_2007_S451	451	417	19	2007	F
2176_1085_S452_	2011	D	09.05.2011	S452	14	21.5	Fam-19_2007_S452	452	418	19	2007	F
2176_1085_S453_	2011	D	09.05.2011	S453	16.9	36.5	Fam-03_2007_S453	453	419	3	2007	W

2176_1085_S455_	2011	D	09.05.2011	S455	14	21	Fam-17_2007_	S455	455	420	17	2007 F
2176_1085_S456_	2011	D	09.05.2011	S456	14.8	24.5	Fam-23_2007_	S456	456	421	23	2007 F
2176_1085_S457_	2011	D	09.05.2011	S457	14.9	27	Fam-03_2007_	S457	457	422	3	2007 W
2176_1085_S459_	2011	D	09.05.2011	S459	13.4	20	Fam-28_2007_	S459	459	423	28	2007 H
2176_1085_S460_	2011	D	09.05.2011	S460	16.4	36.5	Fam-25_2007_	S460	460	424	25	2007 F
2176_1085_S461_	2011	D	09.05.2011	S461	14.9	26	Fam-10_2007_	S461	461	425	10	2007 W
2176_1085_S462_	2011	D	09.05.2011	S462	14	23	Fam-19_2007_	S462	462	426	19	2007 F
2176_1085_S464_	2011	D	09.05.2011	S464	15.1	26	Fam-10_2007_	S464	464	427	10	2007 W
2176_1085_S465_	2011	D	09.05.2011	S465	14.5	22.5	Fam-27_2007_	S465	465	428	27	2007 F
2176_1085_S466_	2011	D	09.05.2011	S466	15.2	27.5	Fam-23_2007_	S466	466	429	23	2007 F
2176_1085_S467_	2011	D	09.05.2011	S467	14.7	26	Fam-13_2007_	S467	467	430	13	2007 F
2176_1085_S468_	2011	D	09.05.2011	S468	15.7	30	Fam-03_2007_	S468	468	431	3	2007 W
2176_1085_S470_	2011	D	09.05.2011	S470	14.5	23	Fam-19_2007_	S470	470	432	19	2007 F
2176_1086_S471_	2011	D	09.05.2011	S471	13.9	19.5	Fam-28_2007_	S471	471	433	28	2007 H

2176_1086_S472_	2011	D	09.05.2011	S472	14.5	24	Fam-03_2007_	S472	472	434	3	2007	W
2176_1086_S473_	2011	D	09.05.2011	S473	13.5	21	Fam-28_2007_	S473	473	435	28	2007	H
2176_1086_S474_	2011	D	09.05.2011	S474	15	26.5	Fam-10_2007_	S474	474	436	10	2007	W
2176_1086_S475_	2011	D	09.05.2011	S475	14	24	Fam-13_2007_	S475	475	437	13	2007	F
2176_1086_S476_	2011	D	09.05.2011	S476	15.1	28	Fam-17_2007_	S476	476	438	17	2007	F
2176_1086_S477_	2011	D	09.05.2011	S477	14	21	Fam-19_2007_	S477	477	439	19	2007	F
2176_1086_S478_	2011	D	09.05.2011	S478	14	21.5	Fam-03_2007_	S478	478	440	3	2007	W
2176_1086_S479_	2011	D	09.05.2011	S479	16	34	Fam-03_2007_	S479	479	441	3	2007	W
2176_1086_S480_	2011	D	09.05.2011	S480	15.1	27	Fam-09_2007_	S480	480	442	9	2007	W
2177_1086_S481_	2011	D	09.05.2011	S481	16.1	32.5	Fam-21_2007_	S481	481	443	21	2007	F
2177_1086_S482_	2011	D	09.05.2011	S482	15.2	28	Fam-30_2007_	S482	482	444	30	2007	H
2177_1086_S483_	2011	D	09.05.2011	S483	13	17.5	Fam-10_2007_	S483	483	445	10	2007	W
2177_1086_S484_	2011	D	09.05.2011	S484	15	27.5	Fam-19_2007_	S484	484	446	19	2007	F
2177_1086_S485_	2011	D	09.05.2011	S485	16.5	36.5	Fam-09_2007_	S485	485	447	9	2007	W

2177_1086_S486_	2011	D	09.05.2011	S486	14.1	21.5	Fam-28_2007_S486	486	448	28	2007	H
2177_1086_S487_	2011	D	09.05.2011	S487	15.5	27.5	Fam-23_2007_S487	487	449	23	2007	F
2177_1086_S488_	2011	D	09.05.2011	S488	15.3	28	Fam-23_2007_S488	488	450	23	2007	F
2177_1086_S489_	2011	D	09.05.2011	S489	14.2	23	Fam-23_2007_S489	489	451	23	2007	F
2177_1086_S490_	2011	D	09.05.2011	S490	15.2	28.5	Fam-23_2007_S490	490	452	23	2007	F
2177_1086_S491_	2011	D	09.05.2011	S491	14.7	24.5	Fam-03_2007_S491	491	453	3	2007	W
2177_1086_S492_	2011	D	09.05.2011	S492	13.9	21	Fam-10_2007_S492	492	454	10	2007	W
2177_1086_S495_	2011	D	09.05.2011	S495	15.4	28	Fam-09_2007_S495	495	455	9	2007	W
2177_1086_S496_	2011	D	09.05.2011	S496	13.8	21.5	Fam-27_2007_S496	496	456	27	2007	F
2177_1086_S497_	2011	D	09.05.2011	S497	16.1	32.5	Fam-09_2007_S497	497	457	9	2007	W
2177_1086_S498_	2011	D	09.05.2011	S498	14	22	Fam-09_2007_S498	498	458	9	2007	W
2177_1086_S499_	2011	D	09.05.2011	S499	14	22.5	Fam-13_2007_S499	499	459	13	2007	F
2177_1086_S500_	2011	D	09.05.2011	S500	14.5	23.5	Fam-03_2007_S500	500	460	3	2007	W
2177_1086_S501_	2011	D	09.05.2011	S501	17.5	43	Fam-28_2007_S501	501	461	28	2007	H

2177_1086_S503_	2011 D	09.05.2011 S503	16	30.5	Fam-09_2007_S503	503	462	9	2007 W
2177_1086_S504_	2011 D	09.05.2011 S504	14.5	23.5	Fam-27_2007_S504	504	463	27	2007 F
2177_1086_S505_	2011 D	09.05.2011 S505	15	25.5	Fam-17_2007_S505	505	464	17	2007 F
2177_1086_S506_	2011 D	09.05.2011 S506	13.8	22.5	Fam-27_2007_S506	506	465	27	2007 F
2177_1086_S507_	2011 D	09.05.2011 S507	14.2	21	Fam-17_2007_S507	507	466	17	2007 F
2177_1086_S508_	2011 D	09.05.2011 S508	15	27	Fam-09_2007_S508	508	467	9	2007 W
2177_1086_S509_	2011 D	09.05.2011 S509	14.1	23	Fam-04_2007_S509	509	468	4	2007 W
2177_1086_S510_	2011 D	09.05.2011 S510	17.1	38.5	Fam-03_2007_S510	510	469	3	2007 W
2177_1086_S511_	2011 D	09.05.2011 S511	15	27	Fam-19_2007_S511	511	470	19	2007 F
2177_1086_S512_	2011 D	09.05.2011 S512	14.5	26	Fam-28_2007_S512	512	471	28	2007 H
2177_1086_S513_	2011 D	09.05.2011 S513	14.3	23	Fam-23_2007_S513	513	472	23	2007 F
2177_1086_S514_	2011 D	09.05.2011 S514	14.5	23.5	Fam-17_2007_S514	514	473	17	2007 F
2177_1086_S515_	2011 D	09.05.2011 S515	15.4	28	Fam-03_2007_S515	515	474	3	2007 W
2177_1086_S516_	2011 D	09.05.2011 S516	18	47	Fam-09_2007_S516	516	475	9	2007 W

2177_1086_S517_	2011	D	09.05.2011	S517	13.4	20	Fam-09_2007_S517	517	476	9	2007	W
2177_1086_S518_	2011	D	09.05.2011	S518	15.3	29.5	Fam-25_2007_S518	518	477	25	2007	F
2177_1086_S519_	2011	D	09.05.2011	S519	14.8	27.5	Fam-04_2007_S519	519	478	4	2007	W
2177_1086_S520_	2011	D	09.05.2011	S520	15.5	29	Fam-19_2007_S520	520	479	19	2007	F
2177_1086_S521_	2011	D	09.05.2011	S521	14.5	25	Fam-27_2007_S521	521	480	27	2007	F
2177_1086_S522_	2011	D	09.05.2011	S522	15.1	30	Fam-27_2007_S522	522	481	27	2007	F
2177_1086_S523_	2011	D	09.05.2011	S523	14.6	26.5	Fam-03_2007_S523	523	482	3	2007	W
2177_1086_S524_	2011	D	09.05.2011	S524	15.2	27.5	Fam-04_2007_S524	524	483	4	2007	W
2177_1086_S525_	2011	D	09.05.2011	S525	13.8	21	Fam-09_2007_S525	525	484	9	2007	W
2177_1086_S526_	2011	D	10.05.2011	S526	14.2	23.5	Fam-17_2007_S526	526	485	17	2007	F
2177_1086_S527_	2011	D	10.05.2011	S527	14.9	26.5	Fam-09_2007_S527	527	486	9	2007	W
2177_1086_S529_	2011	D	10.05.2011	S529	14	23	Fam-19_2007_S529	529	487	19	2007	F
2177_1086_S530_	2011	D	10.05.2011	S530	15	28.5	Fam-09_2007_S530	530	488	9	2007	W
2177_1086_S531_	2011	D	10.05.2011	S531	13.8	20	Fam-23_2007_S531	531	489	23	2007	F

2177_1086_S532_	2011	D	10.05.2011	S532	14	20	Fam-04_2007_S532	532	490	4	2007	W
2177_1086_S533_	2011	D	10.05.2011	S533	13.2	18	Fam-23_2007_S533	533	491	23	2007	F
2177_1086_S534_	2011	D	10.05.2011	S534	13.9	22.5	Fam-04_2007_S534	534	492	4	2007	W
2177_1086_S535_	2011	D	10.05.2011	S535	15.8	31.8	Fam-09_2007_S535	535	493	9	2007	W
2177_1086_S536_	2011	D	10.05.2011	S536	15.7	31	Fam-23_2007_S536	536	494	23	2007	F
2177_1086_S537_	2011	D	10.05.2011	S537	15.9	31.5	Fam-27_2007_S537	537	495	27	2007	F
2177_1086_S538_	2011	D	10.05.2011	S538	15.6	31	Fam-11_2007_S538	538	496	11	2007	F
2177_1086_S539_	2011	D	10.05.2011	S539	14.2	24	Fam-04_2007_S539	539	497	4	2007	W
2177_1086_S540_	2011	D	10.05.2011	S540	13.3	19.5	Fam-04_2007_S540	540	498	4	2007	W
2177_1086_S541_	2011	D	10.05.2011	S541	14.1	22.5	Fam-09_2007_S541	541	499	9	2007	W
2177_1086_S542_	2011	D	10.05.2011	S542	13.9	21	Fam-09_2007_S542	542	500	9	2007	W
2177_1086_S543_	2011	D	10.05.2011	S543	15.8	31.5	Fam-11_2007_S543	543	501	11	2007	F
2177_1086_S544_	2011	D	10.05.2011	S544	13.8	22	Fam-23_2007_S544	544	502	23	2007	F
2177_1086_S545_	2011	D	10.05.2011	S545	15.1	28	Fam-25_2007_S545	545	503	25	2007	F

2177_1086_S546_	2011	D	10.05.2011	S546	15.6	30	Fam-27_2007_	S546	546	504	27	2007	F
2177_1086_S547_	2011	D	10.05.2011	S547	14.6	27	Fam-04_2007_	S547	547	505	4	2007	W
2177_1086_S548_	2011	D	10.05.2011	S548	14.4	23	Fam-03_2007_	S548	548	506	3	2007	W
2177_1086_S549_	2011	D	10.05.2011	S549	14.8	25	Fam-03_2007_	S549	549	507	3	2007	W
2177_1086_S550_	2011	D	10.05.2011	S550	14.1	22.5	Fam-23_2007_	S550	550	508	23	2007	F
2177_1086_S551_	2011	D	10.05.2011	S551	15.3	29.5	Fam-10_2007_	S551	551	509	10	2007	W
2177_1086_S552_	2011	D	10.05.2011	S552	13.9	21.5	Fam-03_2007_	S552	552	510	3	2007	W
2177_1086_S553_	2011	D	10.05.2011	S553	14.1	23	Fam-09_2007_	S553	553	511	9	2007	W
2177_1086_S554_	2011	D	10.05.2011	S554	13.9	23.5	Fam-28_2007_	S554	554	512	28	2007	H
2177_1086_S555_	2011	D	10.05.2011	S555	14.1	22	Fam-27_2007_	S555	555	513	27	2007	F
2177_1086_S556_	2011	D	10.05.2011	S556	14.8	25	Fam-09_2007_	S556	556	514	9	2007	W
2177_1086_S557_	2011	D	10.05.2011	S557	14.8	27	Fam-09_2007_	S557	557	515	9	2007	W
2177_1086_S558_	2011	D	10.05.2011	S558	14.4	22	Fam-09_2007_	S558	558	516	9	2007	W
2177_1086_S559_	2011	D	10.05.2011	S559	15.3	31	Fam-25_2007_	S559	559	517	25	2007	F

2177_1086_S560_	2011	D	10.05.2011	S560	15.3	29	Fam-09_2007_S560	560	518	9	2007	W
2178_1086_S561_	2011	D	10.05.2011	S561	14.8	26.5	Fam-27_2007_S561	561	519	27	2007	F
2178_1086_S563_	2011	D	10.05.2011	S563	13.9	21	Fam-09_2007_S563	563	520	9	2007	W
2178_1086_S564_	2011	D	10.05.2011	S564	15.2	28.5	Fam-17_2007_S564	564	521	17	2007	F
2178_1087_S565_	2011	D	10.05.2011	S565	13.5	20.5	Fam-17_2007_S565	565	522	17	2007	F
2178_1087_S566_	2011	D	10.05.2011	S566	15	27.5	Fam-19_2007_S566	566	523	19	2007	F
2178_1087_S567_	2011	D	10.05.2011	S567	14.1	21.5	Fam-06_2007_S567	567	524	6	2007	W
2178_1087_S569_	2011	D	10.05.2011	S569	15	28.5	Fam-10_2007_S569	569	525	10	2007	W
2178_1087_S570_	2011	D	10.05.2011	S570	14.3	22	Fam-17_2007_S570	570	526	17	2007	F
2178_1087_S571_	2011	D	10.05.2011	S571	13.9	21	Fam-19_2007_S571	571	527	19	2007	F
2178_1087_S572_	2011	D	10.05.2011	S572	14.7	26	Fam-09_2007_S572	572	528	9	2007	W
2178_1087_S573_	2011	D	10.05.2011	S573	14.2	23.5	Fam-03_2007_S573	573	529	3	2007	W
2178_1087_S574_	2011	D	10.05.2011	S574	14.9	26	Fam-09_2007_S574	574	530	9	2007	W
2178_1087_S575_	2011	D	10.05.2011	S575	13.3	19	Fam-06_2007_S575	575	531	6	2007	W

2178_1087_S576_	2011	D	10.05.2011	S576	13.8	19.5	Fam-17_2007_	S576	576	532	17	2007	F
2178_1087_S577_	2011	D	10.05.2011	S577	13.3	19	Fam-10_2007_	S577	577	533	10	2007	W
2178_1087_S578_	2011	D	10.05.2011	S578	14.2	24	Fam-04_2007_	S578	578	534	4	2007	W
2178_1087_S579_	2011	D	10.05.2011	S579	14.6	26.5	Fam-04_2007_	S579	579	535	4	2007	W
2178_1087_S581_	2011	D	10.05.2011	S581	15.5	31	Fam-09_2007_	S581	581	536	9	2007	W
2178_1087_S582_	2011	D	10.05.2011	S582	14.6	25.5	Fam-21_2007_	S582	582	537	21	2007	F
2178_1087_S585_	2011	D	10.05.2011	S585	13.8	21	Fam-09_2007_	S585	585	538	9	2007	W
2178_1087_S586_	2011	D	10.05.2011	S586	13.3	18.5	Fam-30_2007_	S586	586	539	30	2007	H
2178_1087_S587_	2011	D	10.05.2011	S587	14.3	24	Fam-23_2007_	S587	587	540	23	2007	F
2178_1087_S589_	2011	D	10.05.2011	S589	15.8	31	Fam-23_2007_	S589	589	541	23	2007	F
2178_1087_S590_	2011	T	10.05.2011	S590	13.5	18.5	Family-17_200	S590	590	542	17	2007	F
2178_1087_S591_	2011	D	10.05.2011	S591	14.5	25.5	Fam-03_2007_	S591	591	543	3	2007	W
2178_1087_S592_	2011	D	10.05.2011	S592	15.3	28.5	Fam-09_2007_	S592	592	544	9	2007	W
2178_1087_S593_	2011	D	10.05.2011	S593	15.5	28	Fam-17_2007_	S593	593	545	17	2007	F

2178_1087_S594_	2011	D	10.05.2011	S594	13.2	18.5	Fam-17_2007_	S594	594	546	17	2007	F
2178_1087_S595_	2011	D	10.05.2011	S595	13.9	22	Fam-19_2007_	S595	595	547	19	2007	F
2178_1087_S596_	2011	D	10.05.2011	S596	15.3	28.5	Fam-03_2007_	S596	596	548	3	2007	W
2178_1087_S597_	2011	D	10.05.2011	S597	14.3	22	Fam-06_2007_	S597	597	549	6	2007	W
2178_1087_S598_	2011	D	10.05.2011	S598	14.2	23.5	Fam-04_2007_	S598	598	550	4	2007	W
2178_1087_S599_	2011	D	10.05.2011	S599	15.5	29.5	Fam-09_2007_	S599	599	551	9	2007	W
2178_1087_S600_	2011	D	10.05.2011	S600	14.2	24	Fam-09_2007_	S600	600	552	9	2007	W
2178_1087_S602_	2011	D	10.05.2011	S602	14.5	24.5	Fam-09_2007_	S602	602	553	9	2007	W
2178_1087_S603_	2011	D	10.05.2011	S603	14.2	25	Fam-19_2007_	S603	603	554	19	2007	F
2178_1087_S604_	2011	D	10.05.2011	S604	14.2	23.5	Fam-21_2007_	S604	604	555	21	2007	F
2178_1087_S605_	2011	D	10.05.2011	S605	18.1	47.5	Fam-09_2007_	S605	605	556	9	2007	W
2178_1087_S607_	2011	D	10.05.2011	S607	13.4	19.5	Fam-13_2007_	S607	607	557	13	2007	F
2178_1087_S608_	2011	D	10.05.2011	S608	16.5	36	Fam-10_2007_	S608	608	558	10	2007	W
2178_1087_S609_	2011	D	10.05.2011	S609	15.1	26	Fam-04_2007_	S609	609	559	4	2007	W

2178_1087_S610_	2011	D	10.05.2011	S610	14.7	25	Fam-28_2007_	S610	610	560	28	2007	H
2178_1087_S611_	2011	D	10.05.2011	S611	13.6	21.5	Fam-04_2007_	S611	611	561	4	2007	W
2178_1087_S612_	2011	D	10.05.2011	S612	14	21	Fam-21_2007_	S612	612	562	21	2007	F
2178_1087_S613_	2011	D	10.05.2011	S613	14.2	23.5	Fam-09_2007_	S613	613	563	9	2007	W
2178_1087_S614_	2011	D	10.05.2011	S614	15	26.5	Fam-23_2007_	S614	614	564	23	2007	F
2178_1087_S615_	2011	D	10.05.2011	S615	16.7	35	Fam-21_2007_	S615	615	565	21	2007	F
2178_1087_S616_	2011	D	10.05.2011	S616	14.5	24.5	Fam-27_2007_	S616	616	566	27	2007	F
2178_1087_S618_	2011	D	10.05.2011	S618	13.3	19.5	Fam-09_2007_	S618	618	567	9	2007	W
2178_1087_S619_	2011	D	10.05.2011	S619	14.9	25.5	Fam-28_2007_	S619	619	568	28	2007	H
2178_1087_S620_	2011	D	10.05.2011	S620	15.2	28.5	Fam-09_2007_	S620	620	569	9	2007	W
2178_1087_S621_	2011	D	10.05.2011	S621	13.8	22	Fam-09_2007_	S621	621	570	9	2007	W
2178_1087_S622_	2011	D	10.05.2011	S622	14.8	26	Fam-09_2007_	S622	622	571	9	2007	W
2178_1087_S623_	2011	D	10.05.2011	S623	17.7	46	Fam-13_2007_	S623	623	572	13	2007	F
2178_1087_S624_	2011	D	10.05.2011	S624	15.4	30.5	Fam-28_2007_	S624	624	573	28	2007	H

2178_1087_S625_	2011	D	10.05.2011	S625	15	25	Fam-23_2007_S625	625	574	23	2007	F
2178_1087_S626_	2011	D	10.05.2011	S626	15	26	Fam-28_2007_S626	626	575	28	2007	H
2178_1087_S627_	2011	D	10.05.2011	S627	13.3	19.5	Fam-23_2007_S627	627	576	23	2007	F
2178_1087_S628_	2011	D	10.05.2011	S628	14.1	23.5	Fam-09_2007_S628	628	577	9	2007	W
2178_1087_S629_	2011	D	10.05.2011	S629	16.2	34	Fam-04_2007_S629	629	578	4	2007	W
2178_1087_S630_	2011	D	10.05.2011	S630	14.7	25.5	Fam-09_2007_S630	630	579	9	2007	W
2178_1087_S631_	2011	D	10.05.2011	S631	14.8	25	Fam-03_2007_S631	631	580	3	2007	W
2178_1087_S632_	2011	D	10.05.2011	S632	14.5	26.5	Fam-11_2007_S632	632	581	11	2007	F
2178_1087_S633_	2011	D	10.05.2011	S633	15.6	29.5	Fam-03_2007_S633	633	582	3	2007	W
2178_1087_S634_	2011	D	10.05.2011	S634	15	28.5	Fam-09_2007_S634	634	583	9	2007	W
2178_1087_S635_	2011	D	10.05.2011	S635	15.5	29.5	Fam-09_2007_S635	635	584	9	2007	W
2178_1087_S636_	2011	D	10.05.2011	S636	13.4	20.5	Fam-21_2007_S636	636	585	21	2007	F
2178_1087_S637_	2011	D	10.05.2011	S637	14	23	Fam-11_2007_S637	637	586	11	2007	F
2178_1087_S638_	2011	D	10.05.2011	S638	15	27.5	Fam-28_2007_S638	638	587	28	2007	H

2178_1087_S639_	2011	D	10.05.2011	S639	14.6	25	Fam-28_2007_	S639	639	588	28	2007	H
2178_1087_S640_	2011	D	10.05.2011	S640	14.5	24	Fam-09_2007_	S640	640	589	9	2007	W
2179_1087_S641_	2011	D	10.05.2011	S641	13.5	19.5	Fam-03_2007_	S641	641	590	3	2007	W
2179_1087_S642_	2011	D	10.05.2011	S642	14.6	24	Fam-10_2007_	S642	642	591	10	2007	W
2179_1087_S643_	2011	D	10.05.2011	S643	14.5	25.5	Fam-28_2007_	S643	643	592	28	2007	H
2179_1087_S644_	2011	D	10.05.2011	S644	13.9	21	Fam-04_2007_	S644	644	593	4	2007	W
2179_1087_S645_	2011	D	10.05.2011	S645	14.5	24	Fam-09_2007_	S645	645	594	9	2007	W
2179_1087_S646_	2011	D	10.05.2011	S646	14.2	25	Fam-10_2007_	S646	646	595	10	2007	W
2179_1087_S647_	2011	D	10.05.2011	S647	14.6	25	Fam-13_2007_	S647	647	596	13	2007	F
2179_1087_S648_	2011	D	10.05.2011	S648	17.9	44	Fam-09_2007_	S648	648	597	9	2007	W
2179_1087_S649_	2011	D	10.05.2011	S649	15.9	29	Fam-23_2007_	S649	649	598	23	2007	F
2179_1087_S650_	2011	D	10.05.2011	S650	13.5	21	Fam-13_2007_	S650	650	599	13	2007	F
2179_1087_S651_	2011	D	10.05.2011	S651	14.5	24.5	Fam-09_2007_	S651	651	600	9	2007	W
2179_1087_S652_	2011	D	10.05.2011	S652	14.9	27	Fam-10_2007_	S652	652	601	10	2007	W

2179_1087_S653_	2011	D	10.05.2011	S653	16.2	32	Fam-04_2007_S653	653	602	4	2007	W
2179_1087_S654_	2011	D	10.05.2011	S654	14.4	24	Fam-17_2007_S654	654	603	17	2007	F
2179_1087_S655_	2011	D	10.05.2011	S655	14.5	25.5	Fam-17_2007_S655	655	604	17	2007	F
2179_1087_S656_	2011	D	10.05.2011	S656	16.5	26.5	Fam-09_2007_S656	656	605	9	2007	W
2179_1087_S657_	2011	D	10.05.2011	S657	14.5	24	Fam-17_2007_S657	657	606	17	2007	F
2179_1087_S658_	2011	D	10.05.2011	S658	16.2	27.5	Fam-28_2007_S658	658	607	28	2007	H
2179_1088_S659_	2011	D	10.05.2011	S659	14.5	26	Fam-04_2007_S659	659	608	4	2007	W
2179_1088_S660_	2011	D	10.05.2011	S660	16.4	26	Fam-23_2007_S660	660	609	23	2007	F
2179_1088_S661_	2011	D	10.05.2011	S661	16.4	34	Fam-19_2007_S661	661	610	19	2007	F
2179_1088_S663_	2011	D	10.05.2011	S663	18	45.5	Fam-09_2007_S663	663	611	9	2007	W
2179_1088_S664_	2011	D	10.05.2011	S664	14.5	22.5	Fam-09_2007_S664	664	612	9	2007	W
2179_1088_S665_	2011	D	10.05.2011	S665	14.6	25	Fam-19_2007_S665	665	613	19	2007	F
2179_1088_S666_	2011	D	10.05.2011	S666	14	22.5	Fam-03_2007_S666	666	614	3	2007	W
2179_1088_S667_	2011	D	10.05.2011	S667	14	21.5	Fam-09_2007_S667	667	615	9	2007	W

2179_1088_S668_	2011	D	10.05.2011	S668	14.7	30	Fam-09_2007_	S668	668	616	9	2007 W
2179_1088_S669_	2011	D	11.05.2011	S669	14.7	24.5	Fam-19_2007_	S669	669	617	19	2007 F
2179_1088_S670_	2011	D	11.05.2011	S670	14.6	26.5	Fam-04_2007_	S670	670	618	4	2007 W
2179_1088_S671_	2011	D	11.05.2011	S671	14.7	25	Fam-28_2007_	S671	671	619	28	2007 H
2179_1088_S672_	2011	D	11.05.2011	S672	16.6	37	Fam-09_2007_	S672	672	620	9	2007 W
2179_1088_S673_	2011	D	11.05.2011	S673	15.8	33	Fam-05_2007_	S673	673	621	5	2007 W
2179_1088_S674_	2011	D	11.05.2011	S674	15.3	29.5	Fam-04_2007_	S674	674	622	4	2007 W
2179_1088_S675_	2011	D	11.05.2011	S675	16.7	42.5	Fam-09_2007_	S675	675	623	9	2007 W
2179_1088_S676_	2011	D	11.05.2011	S676	15.8	30.5	Fam-23_2007_	S676	676	624	23	2007 F
2179_1088_S677_	2011	D	11.05.2011	S677	15.3	28.5	Fam-09_2007_	S677	677	625	9	2007 W
2179_1088_S678_	2011	D	11.05.2011	S678	14	25.5	Fam-09_2007_	S678	678	626	9	2007 W
2179_1088_S679_	2011	D	11.05.2011	S679	14.5	24.5	Fam-06_2007_	S679	679	627	6	2007 W
2179_1088_S680_	2011	T	11.05.2011	S680	13.6	18.5	Family-17_200	S680	680	628	17	2007 F
2179_1088_S682_	2011	D	11.05.2011	S682	16	33.5	Fam-09_2007_	S682	682	629	9	2007 W

2179_1088_S684_	2011	D	11.05.2011	S684	14.3	23	Fam-17_2007_S684	684	630	17	2007 F
2179_1088_S685_	2011	D	11.05.2011	S685	13.2	18	Fam-17_2007_S685	685	631	17	2007 F
2179_1088_S686_	2011	D	11.05.2011	S686	16.5	35.5	Fam-04_2007_S686	686	632	4	2007 W
2179_1088_S687_	2011	D	11.05.2011	S687	14.2	23	Fam-04_2007_S687	687	633	4	2007 W
2179_1088_S688_	2011	D	11.05.2011	S688	14.5	26	Fam-09_2007_S688	688	634	9	2007 W
2179_1088_S689_	2011	D	11.05.2011	S689	13.4	21	Fam-04_2007_S689	689	635	4	2007 W
2179_1088_S690_	2011	D	11.05.2011	S690	13.9	21.5	Fam-28_2007_S690	690	636	28	2007 H
2179_1088_S691_	2011	D	11.05.2011	S691	15.2	29	Fam-23_2007_S691	691	637	23	2007 F
2179_1088_S693_	2011	D	11.05.2011	S693	15.4	30.5	Fam-09_2007_S693	693	638	9	2007 W
2179_1088_S694_	2011	D	11.05.2011	S694	14.5	26	Fam-03_2007_S694	694	639	3	2007 W
2179_1088_S695_	2011	D	11.05.2011	S695	15.5	30	Fam-17_2007_S695	695	640	17	2007 F
2179_1088_S697_	2011	D	11.05.2011	S697	13.2	18.5	Fam-23_2007_S697	697	641	23	2007 F
2179_1088_S698_	2011	D	11.05.2011	S698	16.2	33.5	Fam-17_2007_S698	698	642	17	2007 F
2179_1088_S699_	2011	D	11.05.2011	S699	14.9	28.5	Fam-09_2007_S699	699	643	9	2007 W

2179_1088_S700_	2011	D	11.05.2011	S700	13.8	22	Fam-25_2007_S700	700	644	25	2007	F
2179_1088_S701_	2011	D	11.05.2011	S701	13.7	21.5	Fam-09_2007_S701	701	645	9	2007	W
2179_1088_S702_	2011	D	11.05.2011	S702	14.2	23	Fam-09_2007_S702	702	646	9	2007	W
2179_1088_S703_	2011	D	11.05.2011	S703	13	17.5	Fam-06_2007_S703	703	647	6	2007	W
2179_1088_S704_	2011	D	11.05.2011	S704	16.3	35	Fam-10_2007_S704	704	648	10	2007	W
2179_1088_S705_	2011	D	11.05.2011	S705	14.2	22.5	Fam-09_2007_S705	705	649	9	2007	W
2179_1088_S706_	2011	D	11.05.2011	S706	14.3	23	Fam-04_2007_S706	706	650	4	2007	W
2179_1088_S707_	2011	D	11.05.2011	S707	14.2	22.5	Fam-27_2007_S707	707	651	27	2007	F
2179_1088_S708_	2011	D	11.05.2011	S708	14.4	25	Fam-25_2007_S708	708	652	25	2007	F
2179_1088_S709_	2011	D	11.05.2011	S709	17.5	43.5	Fam-03_2007_S709	709	653	3	2007	W
2179_1088_S710_	2011	D	11.05.2011	S710	16.1	33.5	Fam-23_2007_S710	710	654	23	2007	F
2179_1088_S711_	2011	D	11.05.2011	S711	15	26.5	Fam-23_2007_S711	711	655	23	2007	F
2179_1088_S712_	2011	D	11.05.2011	S712	15.2	30	Fam-19_2007_S712	712	656	19	2007	F
2179_1088_S713_	2011	D	11.05.2011	S713	14	24	Fam-11_2007_S713	713	657	11	2007	F

2179_1088_S714_	2011	D	11.05.2011	S714	14.2	22.5	Fam-28_2007_S714	714	658	28	2007	H
2179_1088_S715_	2011	D	11.05.2011	S715	14.9	19.5	Fam-03_2007_S715	715	659	3	2007	W
2179_1088_S716_	2011	D	11.05.2011	S716	13.6	21	Fam-27_2007_S716	716	660	27	2007	F
2179_1088_S717_	2011	D	11.05.2011	S717	13.2	19.5	Fam-09_2007_S717	717	661	9	2007	W
2179_1088_S718_	2011	D	11.05.2011	S718	16.6	37	Fam-09_2007_S718	718	662	9	2007	W
2179_1088_S719_	2011	D	11.05.2011	S719	14.5	24.5	Fam-28_2007_S719	719	663	28	2007	H
2179_1088_S720_	2011	D	11.05.2011	S720	14	22.5	Fam-10_2007_S720	720	664	10	2007	W
2180_1088_S722_	2011	D	11.05.2011	S722	15.2	28	Fam-04_2007_S722	722	665	4	2007	W
2180_1088_S723_	2011	D	11.05.2011	S723	14.4	24	Fam-19_2007_S723	723	666	19	2007	F
2180_1088_S725_	2011	D	11.05.2011	S725	13	18.5	Fam-06_2007_S725	725	667	6	2007	W
2180_1088_S726_	2011	D	11.05.2011	S726	15.5	29.5	Fam-11_2007_S726	726	668	11	2007	F
2180_1088_S727_	2011	D	11.05.2011	S727	14.5	24.5	Fam-09_2007_S727	727	669	9	2007	W
2180_1088_S728_	2011	D	11.05.2011	S728	14.6	25	Fam-09_2007_S728	728	670	9	2007	W
2180_1088_S729_	2011	D	11.05.2011	S729	14.5	25	Fam-09_2007_S729	729	671	9	2007	W

2180_1088_S730_	2011	D	11.05.2011	S730	14.5	21	Fam-19_2007_	S730	730	672	19	2007	F
2180_1088_S731_	2011	D	11.05.2011	S731	13.9	21	Fam-28_2007_	S731	731	673	28	2007	H
2180_1088_S732_	2011	D	11.05.2011	S732	16.8	36	Fam-03_2007_	S732	732	674	3	2007	W
2180_1088_S734_	2011	D	11.05.2011	S734	15.6	29.5	Fam-17_2007_	S734	734	675	17	2007	F
2180_1088_S735_	2011	D	11.05.2011	S735	14.9	27.5	Fam-09_2007_	S735	735	676	9	2007	W
2180_1088_S736_	2011	D	11.05.2011	S736	15.4	29	Fam-23_2007_	S736	736	677	23	2007	F
2180_1088_S737_	2011	D	11.05.2011	S737	14.5	25.5	Fam-09_2007_	S737	737	678	9	2007	W
2180_1088_S738_	2011	D	11.05.2011	S738	14.4	23.5	Fam-09_2007_	S738	738	679	9	2007	W
2180_1088_S739_	2011	D	11.05.2011	S739	14.5	25	Fam-03_2007_	S739	739	680	3	2007	W
2180_1088_S740_	2011	D	11.05.2011	S740	15.7	31.5	Fam-04_2007_	S740	740	681	4	2007	W
2180_1088_S741_	2011	D	11.05.2011	S741	13.1	18	Fam-03_2007_	S741	741	682	3	2007	W
2180_1088_S742_	2011	D	11.05.2011	S742	16	29.5	Fam-04_2007_	S742	742	683	4	2007	W
2180_1088_S743_	2011	D	11.05.2011	S743	15	26.5	Fam-17_2007_	S743	743	684	17	2007	F
2180_1088_S744_	2011	D	11.05.2011	S744	16.5	32.5	Fam-21_2007_	S744	744	685	21	2007	F

2180_1088_S745_	2011	D	11.05.2011	S745	14.5	26	Fam-28_2007_	S745	745	686	28	2007	H
2180_1088_S747_	2011	D	11.05.2011	S747	14.6	24.5	Fam-17_2007_	S747	747	687	17	2007	F
2180_1088_S748_	2011	D	11.05.2011	S748	13.8	21.5	Fam-04_2007_	S748	748	688	4	2007	W
2180_1088_S750_	2011	D	11.05.2011	S750	15.6	29.5	Fam-09_2007_	S750	750	689	9	2007	W
2180_1088_S751_	2011	D	11.05.2011	S751	15.3	29	Fam-09_2007_	S751	751	690	9	2007	W
2180_1088_S752_	2011	D	11.05.2011	S752	15.1	27	Fam-23_2007_	S752	752	691	23	2007	F
2180_1089_S753_	2011	D	11.05.2011	S753	14.1	22.5	Fam-10_2007_	S753	753	692	10	2007	W
2180_1089_S754_	2011	D	11.05.2011	S754	14	19	Fam-28_2007_	S754	754	693	28	2007	H
2180_1089_S755_	2011	D	11.05.2011	S755	14.3	23.5	Fam-27_2007_	S755	755	694	27	2007	F
2180_1089_S756_	2011	D	11.05.2011	S756	13	17.5	Fam-19_2007_	S756	756	695	19	2007	F
2180_1089_S757_	2011	D	11.05.2011	S757	14.1	22	Fam-21_2007_	S757	757	696	21	2007	F
2180_1089_S758_	2011	D	11.05.2011	S758	14.9	26	Fam-21_2007_	S758	758	697	21	2007	F
2180_1089_S759_	2011	D	11.05.2011	S759	15.8	32	Fam-05_2007_	S759	759	698	5	2007	W
2180_1089_S760_	2011	D	11.05.2011	S760	14.5	25	Fam-04_2007_	S760	760	699	4	2007	W

2180_1089_S761_	2011	D	11.05.2011	S761	14	23	Fam-04_2007_ S761	761	700	4	2007	W
2180_1089_S763_	2011	D	11.05.2011	S763	14.5	24	Fam-09_2007_ S763	763	701	9	2007	W
2180_1089_S764_	2011	D	11.05.2011	S764	14.1	22	Fam-19_2007_ S764	764	702	19	2007	F
2180_1089_S765_	2011	D	11.05.2011	S765	14.8	26	Fam-03_2007_ S765	765	703	3	2007	W
2180_1089_S766_	2011	D	11.05.2011	S766	13.2	19	Fam-28_2007_ S766	766	704	28	2007	H
2180_1089_S767_	2011	D	11.05.2011	S767	15.3	28.5	Fam-10_2007_ S767	767	705	10	2007	W
2180_1089_S768_	2011	D	11.05.2011	S768	15	28	Fam-09_2007_ S768	768	706	9	2007	W
2180_1089_S769_	2011	D	11.05.2011	S769	14.6	25	Fam-11_2007_ S769	769	707	11	2007	F
2180_1089_S770_	2011	D	11.05.2011	S770	16.3	32.5	Fam-03_2007_ S770	770	708	3	2007	W
2180_1089_S771_	2011	D	11.05.2011	S771	14.3	23.5	Fam-03_2007_ S771	771	709	3	2007	W
2180_1089_S772_	2011	D	11.05.2011	S772	14.5	24.5	Fam-23_2007_ S772	772	710	23	2007	F
2180_1089_S773_	2011	D	11.05.2011	S773	14	22	Fam-09_2007_ S773	773	711	9	2007	W
2180_1089_S774_	2011	D	11.05.2011	S774	14.6	24	Fam-09_2007_ S774	774	712	9	2007	W
2180_1089_S775_	2011	D	11.05.2011	S775	15.1	25	Fam-06_2007_ S775	775	713	6	2007	W

2180_1089_S776_	2011	D	11.05.2011	S776	13.8	24	Fam-11_2007_S776	776	714	11	2007	F
2180_1089_S777_	2011	D	11.05.2011	S777	14.3	25	Fam-04_2007_S777	777	715	4	2007	W
2180_1089_S778_	2011	D	11.05.2011	S778	15.7	30.5	Fam-23_2007_S778	778	716	23	2007	F
2180_1089_S779_	2011	D	11.05.2011	S779	15.3	29.5	Fam-11_2007_S779	779	717	11	2007	F
2180_1089_S780_	2011	D	11.05.2011	S780	14.6	23.5	Fam-27_2007_S780	780	718	27	2007	F
2180_1089_S781_	2011	D	11.05.2011	S781	14.1	24	Fam-10_2007_S781	781	719	10	2007	W
2180_1089_S782_	2011	D	11.05.2011	S782	13.7	19.5	Fam-11_2007_S782	782	720	11	2007	F
2180_1089_S783_	2011	D	11.05.2011	S783	14	22	Fam-11_2007_S783	783	721	11	2007	F
2180_1089_S784_	2011	D	11.05.2011	S784	15.5	33	Fam-05_2007_S784	784	722	5	2007	W
2180_1089_S785_	2011	D	11.05.2011	S785	14.5	25	Fam-25_2007_S785	785	723	25	2007	F
2180_1089_S786_	2011	D	11.05.2011	S786	13.3	19.5	Fam-11_2007_S786	786	724	11	2007	F
2180_1089_S787_	2011	D	11.05.2011	S787	13.8	21	Fam-09_2007_S787	787	725	9	2007	W
2180_1089_S788_	2011	D	11.05.2011	S788	14.3	24.5	Fam-28_2007_S788	788	726	28	2007	H
2180_1089_S789_	2011	D	11.05.2011	S789	14.9	27	Fam-04_2007_S789	789	727	4	2007	W

2180_1089_S790_	2011	D	11.05.2011	S790	14.8	26.5	Fam-04_2007_	S790	790	728	4	2007	W
2180_1089_S791_	2011	D	11.05.2011	S791	14.7	26.5	Fam-09_2007_	S791	791	729	9	2007	W
2180_1089_S793_	2011	D	11.05.2011	S793	13.9	22.5	Fam-09_2007_	S793	793	730	9	2007	W
2180_1089_S794_	2011	D	11.05.2011	S794	14.5	25.5	Fam-04_2007_	S794	794	731	4	2007	W
2180_1089_S795_	2011	D	11.05.2011	S795	15.8	32	Fam-11_2007_	S795	795	732	11	2007	F
2180_1089_S796_	2011	T	11.05.2011	S796	14	19.5	Family-17_200	S796	796	733	17	2007	F
2180_1089_S799_	2011	D	11.05.2011	S799	14.2	23.5	Fam-09_2007_	S799	799	734	9	2007	W
2180_1089_S800_	2011	D	11.05.2011	S800	14.4	23.5	Fam-28_2007_	S800	800	735	28	2007	H
2181_1089_S801_	2011	D	11.05.2011	S801	14.5	23.5	Fam-28_2007_	S801	801	736	28	2007	H
2181_1089_S802_	2011	D	11.05.2011	S802	15.4	28.5	Fam-09_2007_	S802	802	737	9	2007	W
2181_1089_S803_	2011	D	11.05.2011	S803	13.8	19.5	Fam-06_2007_	S803	803	738	6	2007	W
2181_1089_S804_	2011	D	11.05.2011	S804	15.9	33	Fam-04_2007_	S804	804	739	4	2007	W
2181_1089_S805_	2011	D	11.05.2011	S805	14.2	23.5	Fam-30_2007_	S805	805	740	30	2007	H
2181_1089_S806_	2011	D	11.05.2011	S806	15.1	28	Fam-09_2007_	S806	806	741	9	2007	W

2181_1089_S807_	2011	D	11.05.2011	S807	14.6	26	Fam-09_2007_S807	807	742	9	2007	W
2181_1089_S808_	2011	D	11.05.2011	S808	13.5	19.5	Fam-28_2007_S808	808	743	28	2007	H
2181_1089_S809_	2011	D	11.05.2011	S809	14.2	22.5	Fam-11_2007_S809	809	744	11	2007	F
2181_1089_S810_	2011	D	11.05.2011	S810	13.9	21.5	Fam-09_2007_S810	810	745	9	2007	W
2181_1089_S811_	2011	D	11.05.2011	S811	13.4	19	Fam-17_2007_S811	811	746	17	2007	F
2181_1089_S812_	2011	D	11.05.2011	S812	14.9	26	Fam-04_2007_S812	812	747	4	2007	W
2181_1089_S813_	2011	D	11.05.2011	S813	14.2	23.5	Fam-10_2007_S813	813	748	10	2007	W
2181_1089_S814_	2011	D	11.05.2011	S814	14.1	23	Fam-09_2007_S814	814	749	9	2007	W
2181_1089_S815_	2011	D	11.05.2011	S815	15	29	Fam-13_2007_S815	815	750	13	2007	F
2181_1089_S816_	2011	D	11.05.2011	S816	13.4	21	Fam-09_2007_S816	816	751	9	2007	W
2181_1089_S817_	2011	D	11.05.2011	S817	16.2	32.5	Fam-03_2007_S817	817	752	3	2007	W
2181_1089_S818_	2011	D	11.05.2011	S818	15.2	28	Fam-03_2007_S818	818	753	3	2007	W
2181_1089_S819_	2011	D	11.05.2011	S819	15	26	Fam-17_2007_S819	819	754	17	2007	F
2181_1089_S820_	2011	D	11.05.2011	S820	15.1	28	Fam-09_2007_S820	820	755	9	2007	W

2181_1089_S821_	2011	D	11.05.2011	S821	15.5	28.5	Fam-09_2007_	S821	821	756	9	2007	W
2181_1089_S822_	2011	D	11.05.2011	S822	16	33	Fam-10_2007_	S822	822	757	10	2007	W
2181_1089_S823_	2011	D	11.05.2011	S823	14.9	26	Fam-10_2007_	S823	823	758	10	2007	W
2181_1089_S825_	2011	D	11.05.2011	S825	14	22.5	Fam-27_2007_	S825	825	759	27	2007	F
2181_1089_S826_	2011	D	11.05.2011	S826	16	32.5	Fam-10_2007_	S826	826	760	10	2007	W
2181_1089_S827_	2011	D	11.05.2011	S827	14.8	26.5	Fam-17_2007_	S827	827	761	17	2007	F
2181_1089_S828_	2011	D	11.05.2011	S828	14.1	23	Fam-04_2007_	S828	828	762	4	2007	W
2181_1089_S829_	2011	D	11.05.2011	S829	15	26.5	Fam-23_2007_	S829	829	763	23	2007	F
2181_1089_S830_	2011	D	11.05.2011	S830	15	26	Fam-09_2007_	S830	830	764	9	2007	W
2181_1089_S831_	2011	D	11.05.2011	S831	14.5	24	Fam-28_2007_	S831	831	765	28	2007	H
2181_1089_S832_	2011	D	11.05.2011	S832	14.7	24.5	Fam-10_2007_	S832	832	766	10	2007	W
2181_1089_S834_	2011	D	11.05.2011	S834	17.8	47	Fam-09_2007_	S834	834	767	9	2007	W
2181_1089_S835_	2011	D	11.05.2011	S835	15.5	31.5	Fam-27_2007_	S835	835	768	27	2007	F
2181_1089_S836_	2011	D	11.05.2011	S836	14	21.5	Fam-03_2007_	S836	836	769	3	2007	W

2181_1089_S837_	2011	D	11.05.2011	S837	16.4	37.5	Fam-09_2007_ S837	837	770	9	2007	W
2181_1089_S838_	2011	D	11.05.2011	S838	14.6	24	Fam-11_2007_ S838	838	771	11	2007	F
2181_1089_S839_	2011	D	11.05.2011	S839	14.5	24	Fam-09_2007_ S839	839	772	9	2007	W
2181_1089_S840_	2011	D	11.05.2011	S840	14	24	Fam-19_2007_ S840	840	773	19	2007	F
2181_1089_S841_	2011	D	11.05.2011	S841	15.3	27	Fam-11_2007_ S841	841	774	11	2007	F
2181_1089_S842_	2011	D	11.05.2011	S842	13.5	20	Fam-21_2007_ S842	842	775	21	2007	F
2181_1089_S843_	2011	D	11.05.2011	S843	14.7	26	Fam-09_2007_ S843	843	776	9	2007	W
2181_1089_S844_	2011	D	11.05.2011	S844	14.2	21.5	Fam-28_2007_ S844	844	777	28	2007	H
2181_1089_S845_	2011	D	11.05.2011	S845	16.8	39.5	Fam-28_2007_ S845	845	778	28	2007	H
2181_1089_S846_	2011	D	11.05.2011	S846	15	26	Fam-09_2007_ S846	846	779	9	2007	W
2181_1090_S847_	2011	D	11.05.2011	S847	14.5	24	Fam-10_2007_ S847	847	780	10	2007	W
2181_1090_S848_	2011	D	11.05.2011	S848	14.3	24.5	Fam-03_2007_ S848	848	781	3	2007	W
2181_1090_S849_	2011	D	11.05.2011	S849	15.2	77	Fam-09_2007_ S849	849	782	9	2007	W
2181_1090_S850_	2011	D	11.05.2011	S850	15	27	Fam-09_2007_ S850	850	783	9	2007	W

2181_1090_S851_	2011	D	11.05.2011	S851	14.3	24.5	Fam-10_2007_S851	851	784	10	2007 W
2181_1090_S852_	2011	D	11.05.2011	S852	15	27.5	Fam-09_2007_S852	852	785	9	2007 W
2181_1090_S853_	2011	D	11.05.2011	S853	13.2	19.5	Fam-09_2007_S853	853	786	9	2007 W
2181_1090_S854_	2011	D	12.05.2011	S854	15.1	26.5	Fam-09_2007_S854	854	787	9	2007 W
2181_1090_S855_	2011	D	12.05.2011	S855	14.7	25.5	Fam-04_2007_S855	855	788	4	2007 W
2181_1090_S856_	2011	D	12.05.2011	S856	15.1	27.5	Fam-23_2007_S856	856	789	23	2007 F
2181_1090_S857_	2011	D	12.05.2011	S857	13.6	21	Fam-03_2007_S857	857	790	3	2007 W
2181_1090_S858_	2011	D	12.05.2011	S858	14.8	28	Fam-04_2007_S858	858	791	4	2007 W
2181_1090_S859_	2011	D	12.05.2011	S859	14.6	24.5	Fam-09_2007_S859	859	792	9	2007 W
2181_1090_S860_	2011	D	12.05.2011	S860	14.4	23	Fam-28_2007_S860	860	793	28	2007 H
2181_1090_S861_	2011	D	12.05.2011	S861	13.6	21	Fam-28_2007_S861	861	794	28	2007 H
2181_1090_S862_	2011	D	12.05.2011	S862	15.7	31.5	Fam-05_2007_S862	862	795	5	2007 W
2181_1090_S863_	2011	D	12.05.2011	S863	15.1	27.5	Fam-28_2007_S863	863	796	28	2007 H
2181_1090_S864_	2011	D	12.05.2011	S864	13.8	22	Fam-28_2007_S864	864	797	28	2007 H

2181_1090_S865_	2011	D	12.05.2011	S865	15	26.5	Fam-03_2007_	S865	865	798	3	2007	W
2181_1090_S866_	2011	D	12.05.2011	S866	13.3	18	Fam-17_2007_	S866	866	799	17	2007	F
2181_1090_S867_	2011	D	12.05.2011	S867	16.9	40	Fam-09_2007_	S867	867	800	9	2007	W
2181_1090_S868_	2011	D	12.05.2011	S868	15.3	27.5	Fam-23_2007_	S868	868	801	23	2007	F
2181_1090_S869_	2011	D	12.05.2011	S869	13.5	20	Fam-09_2007_	S869	869	802	9	2007	W
2181_1090_S870_	2011	T	12.05.2011	S870	13.1	16	Family-27_200	S870	870	803	27	2007	F
2181_1090_S871_	2011	D	12.05.2011	S871	14.1	24.5	Fam-23_2007_	S871	871	804	23	2007	F
2181_1090_S872_	2011	D	12.05.2011	S872	14.4	25	Fam-17_2007_	S872	872	805	17	2007	F
2181_1090_S875_	2011	D	12.05.2011	S875	15.7	30.5	Fam-03_2007_	S875	875	806	3	2007	W
2181_1090_S876_	2011	D	12.05.2011	S876	13.9	20.5	Fam-11_2007_	S876	876	807	11	2007	F
2181_1090_S877_	2011	D	12.05.2011	S877	14.8	26.5	Fam-09_2007_	S877	877	808	9	2007	W
2181_1090_S878_	2011	D	12.05.2011	S878	14.9	25	Fam-17_2007_	S878	878	809	17	2007	F
2181_1090_S879_	2011	D	12.05.2011	S879	15.5	28.5	Fam-09_2007_	S879	879	810	9	2007	W
2181_1090_S880_	2011	D	12.05.2011	S880	14.2	24.5	Fam-09_2007_	S880	880	811	9	2007	W

2182_1090_S881_	2011	D	12.05.2011	S881	14.1	22	Fam-09_2007_ S881	881	812	9	2007	W
2182_1090_S882_	2011	D	12.05.2011	S882	16.4	37	Fam-04_2007_ S882	882	813	4	2007	W
2182_1090_S883_	2011	D	12.05.2011	S883	15.2	29.5	Fam-04_2007_ S883	883	814	4	2007	W
2182_1090_S884_	2011	D	12.05.2011	S884	14.7	25.5	Fam-23_2007_ S884	884	815	23	2007	F
2182_1090_S885_	2011	D	12.05.2011	S885	15.7	31.5	Fam-19_2007_ S885	885	816	19	2007	F
2182_1090_S886_	2011	D	12.05.2011	S886	15.9	32	Fam-09_2007_ S886	886	817	9	2007	W
2182_1090_S887_	2011	D	12.05.2011	S887	14.5	24	Fam-21_2007_ S887	887	818	21	2007	F
2182_1090_S888_	2011	D	12.05.2011	S888	18	48.5	Fam-10_2007_ S888	888	819	10	2007	W
2182_1090_S889_	2011	D	12.05.2011	S889	15.7	31.5	Fam-10_2007_ S889	889	820	10	2007	W
2182_1090_S890_	2011	D	12.05.2011	S890	14	21.5	Fam-03_2007_ S890	890	821	3	2007	W
2182_1090_S891_	2011	D	12.05.2011	S891	16.5	35	Fam-23_2007_ S891	891	822	23	2007	F
2182_1090_S892_	2011	D	12.05.2011	S892	13.4	19.5	Fam-17_2007_ S892	892	823	17	2007	F
2182_1090_S893_	2011	D	12.05.2011	S893	14.6	25.5	Fam-11_2007_ S893	893	824	11	2007	F
2182_1090_S894_	2011	D	12.05.2011	S894	14.8	26	Fam-03_2007_ S894	894	825	3	2007	W

2182_1090_S897_	2011	D	12.05.2011	S897	15	26.5	Fam-28_2007_S897	897	826	28	2007	H
2182_1090_S898_	2011	D	12.05.2011	S898	14	20.5	Fam-09_2007_S898	898	827	9	2007	W
2182_1090_S899_	2011	D	12.05.2011	S899	14.5	26.5	Fam-04_2007_S899	899	828	4	2007	W
2182_1090_S901_	2011	D	12.05.2011	S901	15.5	31.5	Fam-17_2007_S901	901	829	17	2007	F
2182_1090_S902_	2011	D	12.05.2011	S902	14	23	Fam-04_2007_S902	902	830	4	2007	W
2182_1090_S903_	2011	D	12.05.2011	S903	14.5	26.5	Fam-19_2007_S903	903	831	19	2007	F
2182_1090_S904_	2011	D	12.05.2011	S904	14.5	25.5	Fam-05_2007_S904	904	832	5	2007	W
2182_1090_S905_	2011	D	12.05.2011	S905	14	22.5	Fam-04_2007_S905	905	833	4	2007	W
2182_1090_S906_	2011	D	12.05.2011	S906	15.5	32	Fam-27_2007_S906	906	834	27	2007	F
2182_1090_S907_	2011	D	12.05.2011	S907	15	28.5	Fam-28_2007_S907	907	835	28	2007	H
2182_1090_S908_	2011	D	12.05.2011	S908	14.7	24.5	Fam-03_2007_S908	908	836	3	2007	W
2182_1090_S909_	2011	D	12.05.2011	S909	13.9	22	Fam-11_2007_S909	909	837	11	2007	F
2182_1090_S910_	2011	D	12.05.2011	S910	17.9	43.5	Fam-07_2007_S910	910	838	7	2007	W
2182_1090_S911_	2011	D	12.05.2011	S911	13.9	21.5	Fam-09_2007_S911	911	839	9	2007	W

2182_1090_S912_	2011	D	12.05.2011	S912	13.5	20	Fam-03_2007_S912	912	840	3	2007	W
2182_1090_S913_	2011	D	12.05.2011	S913	14.9	27	Fam-09_2007_S913	913	841	9	2007	W
2182_1090_S914_	2011	D	12.05.2011	S914	13.4	20	Fam-09_2007_S914	914	842	9	2007	W
2182_1090_S915_	2011	D	12.05.2011	S915	14.6	24.5	Fam-03_2007_S915	915	843	3	2007	W
2182_1090_S916_	2011	D	12.05.2011	S916	15.1	36	Fam-04_2007_S916	916	844	4	2007	W
2182_1090_S917_	2011	D	13.05.2011	S917	13.1	18.5	Fam-09_2007_S917	917	845	9	2007	W
2182_1090_S918_	2011	D	13.05.2011	S918	15	25.5	Fam-28_2007_S918	918	846	28	2007	H
2182_1090_S919_	2011	D	13.05.2011	S919	14.8	26	Fam-25_2007_S919	919	847	25	2007	F
2182_1090_S920_	2011	D	13.05.2011	S920	15.1	25.5	Fam-17_2007_S920	920	848	17	2007	F
2182_1090_S921_	2011	D	13.05.2011	S921	14.3	23.5	Fam-17_2007_S921	921	849	17	2007	F
2182_1090_S922_	2011	D	13.05.2011	S922	14.5	23.5	Fam-17_2007_S922	922	850	17	2007	F
2182_1090_S923_	2011	D	13.05.2011	S923	15.1	27.5	Fam-09_2007_S923	923	851	9	2007	W
2182_1090_S924_	2011	D	13.05.2011	S924	14.4	23	Fam-11_2007_S924	924	852	11	2007	F
2182_1090_S925_	2011	D	13.05.2011	S925	14.1	23.5	Fam-03_2007_S925	925	853	3	2007	W

2182_1090_S926_	2011	D	13.05.2011	S926	13.7	20	Fam-23_2007_S926	926	854	23	2007	F
2182_1090_S927_	2011	D	13.05.2011	S927	16	31	Fam-04_2007_S927	927	855	4	2007	W
2182_1090_S928_	2011	D	13.05.2011	S928	14.5	26	Fam-17_2007_S928	928	856	17	2007	F
2182_1090_S929_	2011	D	13.05.2011	S929	13.5	19	Fam-04_2007_S929	929	857	4	2007	W
2182_1090_S930_	2011	D	13.05.2011	S930	14.3	22	Fam-13_2007_S930	930	858	13	2007	F
2182_1090_S931_	2011	D	13.05.2011	S931	13.4	19	Fam-11_2007_S931	931	859	11	2007	F
2182_1090_S932_	2011	D	13.05.2011	S932	15.1	27.5	Fam-21_2007_S932	932	860	21	2007	F
2182_1090_S933_	2011	D	13.05.2011	S933	15	25	Fam-17_2007_S933	933	861	17	2007	F
2182_1090_S934_	2011	D	13.05.2011	S934	14.5	24	Fam-17_2007_S934	934	862	17	2007	F
2182_1090_S935_	2011	D	13.05.2011	S935	14.8	25	Fam-09_2007_S935	935	863	9	2007	W
2182_1090_S936_	2011	D	13.05.2011	S936	14.5	24	Fam-07_2007_S936	936	864	7	2007	W
2182_1090_S937_	2011	D	13.05.2011	S937	14.8	25	Fam-21_2007_S937	937	865	21	2007	F
2182_1090_S938_	2011	D	13.05.2011	S938	15.1	26.5	Fam-17_2007_S938	938	866	17	2007	F
2182_1090_S939_	2011	D	13.05.2011	S939	14.5	25	Fam-10_2007_S939	939	867	10	2007	W

2182_1090_S940_	2011	D	13.05.2011	S940	14.5	24	Fam-09_2007_	S940	940	868	9	2007	W
2182_1091_S941_	2011	D	13.05.2011	S941	13.2	17.5	Fam-19_2007_	S941	941	869	19	2007	F
2182_1091_S943_	2011	D	13.05.2011	S943	14.5	24	Fam-13_2007_	S943	943	870	13	2007	F
2182_1091_S944_	2011	D	13.05.2011	S944	16	31.5	Fam-03_2007_	S944	944	871	3	2007	W
2182_1091_S945_	2011	D	13.05.2011	S945	14.5	23.5	Fam-03_2007_	S945	945	872	3	2007	W
2182_1091_S946_	2011	D	13.05.2011	S946	15.7	30.5	Fam-23_2007_	S946	946	873	23	2007	F
2182_1091_S947_	2011	D	13.05.2011	S947	14.4	24.5	Fam-23_2007_	S947	947	874	23	2007	F
2182_1091_S948_	2011	D	13.05.2011	S948	13.8	21.5	Fam-25_2007_	S948	948	875	25	2007	F
2182_1091_S949_	2011	D	13.05.2011	S949	15	27	Fam-09_2007_	S949	949	876	9	2007	W
2182_1091_S950_	2011	D	13.05.2011	S950	16.3	35	Fam-23_2007_	S950	950	877	23	2007	F
2182_1091_S951_	2011	D	13.05.2011	S951	14.1	22	Fam-23_2007_	S951	951	878	23	2007	F
2182_1091_S954_	2011	D	13.05.2011	S954	14.9	27	Fam-19_2007_	S954	954	879	19	2007	F
2182_1091_S955_	2011	D	13.05.2011	S955	15	27	Fam-09_2007_	S955	955	880	9	2007	W
2182_1091_S956_	2011	D	13.05.2011	S956	13.8	21.5	Fam-10_2007_	S956	956	881	10	2007	W

2182_1091_S957_	2011	D	13.05.2011	S957	16	34	Fam-09_2007_	S957	957	882	9	2007	W
2182_1091_S958_	2011	D	13.05.2011	S958	15	25.5	Fam-28_2007_	S958	958	883	28	2007	H
2182_1091_S959_	2011	D	13.05.2011	S959	16	32.5	Fam-17_2007_	S959	959	884	17	2007	F
2182_1091_S960_	2011	D	13.05.2011	S960	14	22	Fam-09_2007_	S960	960	885	9	2007	W
2183_1091_S961_	2011	D	13.05.2011	S961	15	26.5	Fam-03_2007_	S961	961	886	3	2007	W
2183_1091_S962_	2011	D	13.05.2011	S962	14	22.5	Fam-03_2007_	S962	962	887	3	2007	W
2183_1091_S963_	2011	D	13.05.2011	S963	15.7	30	Fam-17_2007_	S963	963	888	17	2007	F
2183_1091_S964_	2011	D	16.05.2011	S964	15.4	28	Fam-23_2007_	S964	964	889	23	2007	F
2183_1091_S965_	2011	D	16.05.2011	S965	15.3	27	Fam-21_2007_	S965	965	890	21	2007	F
2183_1091_S966_	2011	D	16.05.2011	S966	14.9	26	Fam-09_2007_	S966	966	891	9	2007	W
2183_1091_S967_	2011	D	16.05.2011	S967	14.8	27.5	Fam-28_2007_	S967	967	892	28	2007	H
2183_1091_S968_	2011	D	16.05.2011	S968	14.8	27.5	Fam-11_2007_	S968	968	893	11	2007	F
2183_1091_S969_	2011	D	16.05.2011	S969	15	26.5	Fam-03_2007_	S969	969	894	3	2007	W
2183_1091_S970_	2011	D	16.05.2011	S970	16.6	35.5	Fam-03_2007_	S970	970	895	3	2007	W

2183_1091_S971_	2011	D	16.05.2011	S971	14.7	23	Fam-28_2007_	S971	971	896	28	2007	H
2183_1091_S972_	2011	D	16.05.2011	S972	15.2	28	Fam-11_2007_	S972	972	897	11	2007	F
2183_1091_S974_	2011	D	16.05.2011	S974	15.7	32	Fam-19_2007_	S974	974	898	19	2007	F
2183_1091_S975_	2011	D	16.05.2011	S975	16	32	Fam-03_2007_	S975	975	899	3	2007	W
2183_1091_S976_	2011	D	16.05.2011	S976	14.5	25	Fam-23_2007_	S976	976	900	23	2007	F
2183_1091_S977_	2011	D	16.05.2011	S977	16.5	35.5	Fam-09_2007_	S977	977	901	9	2007	W
2183_1091_S978_	2011	D	16.05.2011	S978	14	19	Fam-03_2007_	S978	978	902	3	2007	W
2183_1091_S979_	2011	D	16.05.2011	S979	15.4	30.5	Fam-11_2007_	S979	979	903	11	2007	F
2183_1091_S981_	2011	D	16.05.2011	S981	13.8	21	Fam-09_2007_	S981	981	904	9	2007	W
2183_1091_S982_	2011	D	16.05.2011	S982	15	26.5	Fam-10_2007_	S982	982	905	10	2007	W
2183_1091_S983_	2011	D	16.05.2011	S983	13.2	19	Fam-27_2007_	S983	983	906	27	2007	F
2183_1091_S984_	2011	D	16.05.2011	S984	16.1	36	Fam-25_2007_	S984	984	907	25	2007	F
2183_1091_S985_	2011	D	16.05.2011	S985	14.5	23.5	Fam-09_2007_	S985	985	908	9	2007	W
2183_1091_S986_	2011	D	16.05.2011	S986	14.3	23.5	Fam-19_2007_	S986	986	909	19	2007	F

2183_1091_S987_	2011	D	16.05.2011	S987	15.2	28	Fam-11_2007_S987	987	910	11	2007	F
2183_1091_S988_	2011	D	16.05.2011	S988	13.4	19	Fam-03_2007_S988	988	911	3	2007	W
2183_1091_S989_	2011	D	16.05.2011	S989	14.6	24.5	Fam-09_2007_S989	989	912	9	2007	W
2183_1091_S990_	2011	D	16.05.2011	S990	15.2	27.5	Fam-11_2007_S990	990	913	11	2007	F
2183_1091_S991_	2011	D	16.05.2011	S991	15.6	30.5	Fam-19_2007_S991	991	914	19	2007	F
2183_1091_S994_	2011	D	16.05.2011	S994	14.5	24.5	Fam-09_2007_S994	994	915	9	2007	W
2183_1091_S995_	2011	D	16.05.2011	S995	14.5	24.5	Fam-28_2007_S995	995	916	28	2007	H
2183_1091_S996_	2011	D	16.05.2011	S996	13.6	21	Fam-19_2007_S996	996	917	19	2007	F
2183_1091_S997_	2011	D	16.05.2011	S997	13.6	19.5	Fam-28_2007_S997	997	918	28	2007	H
2183_1091_S998_	2011	D	16.05.2011	S998	14.5	22.5	Fam-03_2007_S998	998	919	3	2007	W
2183_1091_S999_	2011	D	16.05.2011	S999	14.4	24.5	Fam-09_2007_S999	999	920	9	2007	W
2183_1091_S1000	2011	D	16.05.2011	S1000	15.5	31	Fam-09_2007_S1000	1000	921	9	2007	W
2183_1091_S1001	2011	D	16.05.2011	S1001	14.8	26.5	Fam-28_2007_S1001	1001	922	28	2007	H
2183_1091_S1002	2011	D	16.05.2011	S1002	14.8	28	Fam-25_2007_S1002	1002	923	25	2007	F

2183_1091_S1003	2011	D	16.05.2011	S1003	14	20.5	Fam-10_2007_S1003	1003	924	10	2007	W
2183_1091_S1004	2011	D	16.05.2011	S1004	14.7	25	Fam-11_2007_S1004	1004	925	11	2007	F
2183_1091_S1005	2011	D	16.05.2011	S1005	17.2	37.5	Fam-09_2007_S1005	1005	926	9	2007	W
2183_1091_S1006	2011	D	16.05.2011	S1006	15.7	31.5	Fam-28_2007_S1006	1006	927	28	2007	H
2183_1091_S1007	2011	D	16.05.2011	S1007	14.8	26	Fam-09_2007_S1007	1007	928	9	2007	W
2183_1091_S1008	2011	D	16.05.2011	S1008	17.7	25	Fam-23_2007_S1008	1008	929	23	2007	F
2183_1091_S1009	2011	D	16.05.2011	S1009	15	27	Fam-03_2007_S1009	1009	930	3	2007	W
2183_1091_S1010	2011	D	16.05.2011	S1010	14.5	25	Fam-28_2007_S1010	1010	931	28	2007	H
2183_1091_S1011	2011	D	16.05.2011	S1011	14.4	22	Fam-09_2007_S1011	1011	932	9	2007	W
2183_1091_S1012	2011	D	16.05.2011	S1012	15.2	27.5	Fam-28_2007_S1012	1012	933	28	2007	H
2183_1091_S1013	2011	D	16.05.2011	S1013	17.2	40.5	Fam-09_2007_S1013	1013	934	9	2007	W
2183_1091_S1014	2011	D	16.05.2011	S1014	14.5	24.5	Fam-28_2007_S1014	1014	935	28	2007	H
2183_1091_S1015	2011	D	16.05.2011	S1015	14.7	24	Fam-10_2007_S1015	1015	936	10	2007	W
2183_1091_S1016	2011	D	16.05.2011	S1016	15	28	Fam-04_2007_S1016	1016	937	4	2007	W

2183_1091_S1017	2011	D	16.05.2011	S1017	16.9	38	Fam-04_2007_	S1017	1017	938	4	2007	W
2183_1091_S1018	2011	D	16.05.2011	S1018	13.9	21.5	Fam-09_2007_	S1018	1018	939	9	2007	W
2183_1091_S1019	2011	D	16.05.2011	S1019	15	26	Fam-03_2007_	S1019	1019	940	3	2007	W
2183_1091_S1020	2011	D	16.05.2011	S1020	14.2	23.5	Fam-09_2007_	S1020	1020	941	9	2007	W
2183_1091_S1021	2011	D	16.05.2011	S1021	15.5	32	Fam-28_2007_	S1021	1021	942	28	2007	H
2183_1091_S1022	2011	D	16.05.2011	S1022	13.5	18.5	Fam-17_2007_	S1022	1022	943	17	2007	F
2183_1091_S1023	2011	D	16.05.2011	S1023	14.4	24.5	Fam-10_2007_	S1023	1023	944	10	2007	W
2183_1091_S1024	2011	D	16.05.2011	S1024	13.9	23	Fam-09_2007_	S1024	1024	945	9	2007	W
2183_1091_S1025	2011	D	16.05.2011	S1025	13.4	19.5	Fam-17_2007_	S1025	1025	946	17	2007	F
2183_1091_S1026	2011	D	16.05.2011	S1026	14	21.5	Fam-03_2007_	S1026	1026	947	3	2007	W
2183_1091_S1027	2011	D	16.05.2011	S1027	15.9	31	Fam-28_2007_	S1027	1027	948	28	2007	H
2183_1091_S1028	2011	D	16.05.2011	S1028	13.5	25	Fam-17_2007_	S1028	1028	949	17	2007	F
2183_1091_S1029	2011	D	16.05.2011	S1029	13.5	19	Fam-28_2007_	S1029	1029	950	28	2007	H
2183_1091_S1030	2011	D	16.05.2011	S1030	13.9	22.5	Fam-27_2007_	S1030	1030	951	27	2007	F

2183_1091_S1031	2011	D	16.05.2011	S1031	15.1	28	Fam-03_2007_S1031	1031	952	3	2007	W
2183_1091_S1032	2011	D	16.05.2011	S1032	15	27	Fam-09_2007_S1032	1032	953	9	2007	W
2183_1091_S1033	2011	D	16.05.2011	S1033	15.4	29	Fam-23_2007_S1033	1033	954	23	2007	F
2183_1091_S1034	2011	D	16.05.2011	S1034	14.9	28	Fam-10_2007_S1034	1034	955	10	2007	W
2183_1092_S1035	2011	D	16.05.2011	S1035	13.8	22	Fam-04_2007_S1035	1035	956	4	2007	W
2183_1092_S1036	2011	D	16.05.2011	S1036	13.5	21	Fam-27_2007_S1036	1036	957	27	2007	F
2183_1092_S1037	2011	D	16.05.2011	S1037	14.5	26	Fam-10_2007_S1037	1037	958	10	2007	W
2183_1092_S1038	2011	D	16.05.2011	S1038	14	22.5	Fam-27_2007_S1038	1038	959	27	2007	F
2183_1092_S1039	2011	D	16.05.2011	S1039	14.8	25.5	Fam-09_2007_S1039	1039	960	9	2007	W
2184_1092_S1041	2011	D	16.05.2011	S1041	16.1	33	Fam-17_2007_S1041	1041	961	17	2007	F
2184_1092_S1042	2011	D	16.05.2011	S1042	15.5	31	Fam-11_2007_S1042	1042	962	11	2007	F
2184_1092_S1043	2011	D	16.05.2011	S1043	14.2	25	Fam-09_2007_S1043	1043	963	9	2007	W
2184_1092_S1045	2011	D	16.05.2011	S1045	15	27	Fam-04_2007_S1045	1045	964	4	2007	W
2184_1092_S1046	2011	D	16.05.2011	S1046	14.3	22.5	Fam-17_2007_S1046	1046	965	17	2007	F

2184_1092_S1047	2011	D	16.05.2011	S1047	14.5	24.5	Fam-03_2007_	S1047	1047	966	3	2007	W
2184_1092_S1048	2011	D	16.05.2011	S1048	13.8	21	Fam-09_2007_	S1048	1048	967	9	2007	W
2184_1092_S1049	2011	D	16.05.2011	S1049	13.7	22	Fam-04_2007_	S1049	1049	968	4	2007	W
2184_1092_S1050	2011	D	16.05.2011	S1050	13.4	20.5	Fam-13_2007_	S1050	1050	969	13	2007	F
2184_1092_S1051	2011	D	16.05.2011	S1051	14.5	25.5	Fam-13_2007_	S1051	1051	970	13	2007	F
2184_1092_S1052	2011	D	16.05.2011	S1052	15.5	30.5	Fam-04_2007_	S1052	1052	971	4	2007	W
2184_1092_S1054	2011	D	16.05.2011	S1054	14.6	25.5	Fam-09_2007_	S1054	1054	972	9	2007	W
2184_1092_S1055	2011	D	16.05.2011	S1055	14.4	24	Fam-27_2007_	S1055	1055	973	27	2007	F
2184_1092_S1056	2011	D	16.05.2011	S1056	14.5	24.5	Fam-03_2007_	S1056	1056	974	3	2007	W
2184_1092_S1057	2011	D	18.05.2011	S1057	14.5	23.5	Fam-23_2007_	S1057	1057	975	23	2007	F
2184_1092_S1058	2011	D	18.05.2011	S1058	17.6	26.5	Fam-04_2007_	S1058	1058	976	4	2007	W
2184_1092_S1059	2011	D	18.05.2011	S1059	15.4	27.5	Fam-28_2007_	S1059	1059	977	28	2007	H
2184_1092_S1060	2011	D	18.05.2011	S1060	15.5	31	Fam-09_2007_	S1060	1060	978	9	2007	W
2184_1092_S1061	2011	D	18.05.2011	S1061	15.2	28	Fam-03_2007_	S1061	1061	979	3	2007	W

2184_1092_S1062	2011	D	18.05.2011	S1062	15.1	28	Fam-13_2007_	S1062	1062	980	13	2007	F
2184_1092_S1063	2011	D	18.05.2011	S1063	15.5	30.5	Fam-11_2007_	S1063	1063	981	11	2007	F
2184_1092_S1064	2011	D	18.05.2011	S1064	16.3	34	Fam-09_2007_	S1064	1064	982	9	2007	W
2184_1092_S1066	2011	D	18.05.2011	S1066	14.2	22.5	Fam-03_2007_	S1066	1066	983	3	2007	W
2184_1092_S1067	2011	D	18.05.2011	S1067	15.9	32.5	Fam-23_2007_	S1067	1067	984	23	2007	F
2184_1092_S1068	2011	D	18.05.2011	S1068	15.4	29.5	Fam-19_2007_	S1068	1068	985	19	2007	F
2184_1092_S1069	2011	D	18.05.2011	S1069	15	26	Fam-11_2007_	S1069	1069	986	11	2007	F
2184_1092_S1070	2011	D	18.05.2011	S1070	14.8	26.5	Fam-13_2007_	S1070	1070	987	13	2007	F
2184_1092_S1072	2011	D	18.05.2011	S1072	13.7	20.5	Fam-17_2007_	S1072	1072	988	17	2007	F
2184_1092_S1073	2011	D	18.05.2011	S1073	14.4	24	Fam-17_2007_	S1073	1073	989	17	2007	F
2184_1092_S1074	2011	D	18.05.2011	S1074	13.3	19	Fam-17_2007_	S1074	1074	990	17	2007	F
2184_1092_S1075	2011	D	18.05.2011	S1075	14.5	25	Fam-09_2007_	S1075	1075	991	9	2007	W
2184_1092_S1076	2011	D	18.05.2011	S1076	14.2	24	Fam-27_2007_	S1076	1076	992	27	2007	F
2184_1092_S1077	2011	D	18.05.2011	S1077	14	21.5	Fam-03_2007_	S1077	1077	993	3	2007	W

2184_1092_S1078	2011	D	18.05.2011	S1078	15.3	30	Fam-04_2007_	S1078	1078	994	4	2007	W
2184_1092_S1079	2011	D	18.05.2011	S1079	14.1	23	Fam-28_2007_	S1079	1079	995	28	2007	H
2184_1092_S1080	2011	D	18.05.2011	S1080	15.6	31.5	Fam-11_2007_	S1080	1080	996	11	2007	F
2184_1092_S1081	2011	D	18.05.2011	S1081	14.4	24.5	Fam-04_2007_	S1081	1081	997	4	2007	W
2184_1092_S1082	2011	D	18.05.2011	S1082	13.9	22	Fam-28_2007_	S1082	1082	998	28	2007	H
2184_1092_S1083	2011	D	18.05.2011	S1083	14.3	24	Fam-11_2007_	S1083	1083	999	11	2007	F
2184_1092_S1084	2011	D	18.05.2011	S1084	14.1	23.5	Fam-17_2007_	S1084	1084	1000	17	2007	F
2184_1092_S1085	2011	D	18.05.2011	S1085	15	26.5	Fam-17_2007_	S1085	1085	1001	17	2007	F
2184_1092_S1086	2011	D	18.05.2011	S1086	14.4	25	Fam-09_2007_	S1086	1086	1002	9	2007	W
2184_1092_S1087	2011	D	18.05.2011	S1087	14.5	23	Fam-17_2007_	S1087	1087	1003	17	2007	F
2184_1092_S1088	2011	D	18.05.2011	S1088	15.6	28.5	Fam-09_2007_	S1088	1088	1004	9	2007	W
2184_1092_S1089	2011	D	18.05.2011	S1089	15.7	29.5	Fam-09_2007_	S1089	1089	1005	9	2007	W
2184_1092_S1090	2011	D	18.05.2011	S1090	15.2	27.5	Fam-03_2007_	S1090	1090	1006	3	2007	W
2184_1092_S1091	2011	D	18.05.2011	S1091	14.4	24	Fam-30_2007_	S1091	1091	1007	30	2007	H

2184_1092_S1092	2011	D	19.05.2011	S1092	15.1	29	Fam-03_2007_	S1092	1092	1008	3	2007	W
2184_1092_S1093	2011	D	19.05.2011	S1093	15.3	28.5	Fam-17_2007_	S1093	1093	1009	17	2007	F
2184_1092_S1094	2011	D	19.05.2011	S1094	16.1	33.5	Fam-09_2007_	S1094	1094	1010	9	2007	W
2184_1092_S1095	2011	D	19.05.2011	S1095	14.9	26.5	Fam-17_2007_	S1095	1095	1011	17	2007	F
2184_1092_S1096	2011	D	19.05.2011	S1096	14.7	25.5	Fam-30_2007_	S1096	1096	1012	30	2007	H
2184_1092_S1097	2011	D	19.05.2011	S1097	14.3	24.5	Fam-10_2007_	S1097	1097	1013	10	2007	W
2184_1092_S1098	2011	D	19.05.2011	S1098	15.7	33.5	Fam-09_2007_	S1098	1098	1014	9	2007	W
2184_1092_S1099	2011	D	19.05.2011	S1099	15.1	28.5	Fam-04_2007_	S1099	1099	1015	4	2007	W
2184_1092_S1100	2011	D	19.05.2011	S1100	15.2	27	Fam-28_2007_	S1100	1100	1016	28	2007	H
2184_1092_S1101	2011	D	19.05.2011	S1101	15.5	30.5	Fam-09_2007_	S1101	1101	1017	9	2007	W
2184_1092_S1102	2011	D	19.05.2011	S1102	15.5	30	Fam-21_2007_	S1102	1102	1018	21	2007	F
2184_1092_S1103	2011	D	19.05.2011	S1103	14.8	27.5	Fam-28_2007_	S1103	1103	1019	28	2007	H
2184_1092_S1104	2011	D	19.05.2011	S1104	14.5	23	Fam-03_2007_	S1104	1104	1020	3	2007	W
2184_1092_S1105	2011	D	19.05.2011	S1105	13.5	20	Fam-19_2007_	S1105	1105	1021	19	2007	F

2184_1092_S1106	2011	D	19.05.2011	S1106	15.3	29	Fam-28_2007_ S1106	1106	1022	28	2007	H
2184_1092_S1107	2011	D	19.05.2011	S1107	13.5	20	Fam-04_2007_ S1107	1107	1023	4	2007	W
2184_1092_S1110	2011	D	19.05.2011	S1110	14.4	25.5	Fam-09_2007_ S1110	1110	1024	9	2007	W
2184_1092_S1111	2011	D	19.05.2011	S1111	12.7	17	Fam-03_2007_ S1111	1111	1025	3	2007	W
2184_1092_S1112	2011	D	19.05.2011	S1112	15.1	27.5	Fam-21_2007_ S1112	1112	1026	21	2007	F
2184_1092_S1113	2011	D	19.05.2011	S1113	14.2	21.5	Fam-21_2007_ S1113	1113	1027	21	2007	F
2184_1092_S1114	2011	D	19.05.2011	S1114	14.2	24	Fam-05_2007_ S1114	1114	1028	5	2007	W
2184_1092_S1115	2011	D	19.05.2011	S1115	15.2	27.5	Fam-06_2007_ S1115	1115	1029	6	2007	W
2184_1092_S1116	2011	D	19.05.2011	S1116	16.2	37.5	Fam-04_2007_ S1116	1116	1030	4	2007	W
2184_1092_S1117	2011	D	19.05.2011	S1117	16.8	37.5	Fam-09_2007_ S1117	1117	1031	9	2007	W
2184_1092_S1118	2011	D	19.05.2011	S1118	16.6	38.5	Fam-09_2007_ S1118	1118	1032	9	2007	W
2184_1092_S1119	2011	D	19.05.2011	S1119	15.5	30.5	Fam-09_2007_ S1119	1119	1033	9	2007	W
2184_1092_S1120	2011	D	19.05.2011	S1120	15.3	30	Fam-09_2007_ S1120	1120	1034	9	2007	W
2184_1092_S1122	2011	D	19.05.2011	S1122	14	23.5	Fam-04_2007_ S1122	1122	1035	4	2007	W

2184_1092_S1123	2011	D	19.05.2011	S1123	14.6	26	Fam-09_2007_	S1123	1123	1036	9	2007	W
2184_1092_S1124	2011	D	19.05.2011	S1124	15.8	31.5	Fam-11_2007_	S1124	1124	1037	11	2007	F
2184_1092_S1125	2011	D	19.05.2011	S1125	14.9	28.5	Fam-09_2007_	S1125	1125	1038	9	2007	W
2184_1092_S1126	2011	D	19.05.2011	S1126	15.5	31.5	Fam-04_2007_	S1126	1126	1039	4	2007	W
2184_1092_S1127	2011	D	19.05.2011	S1127	14.7	25.5	Fam-09_2007_	S1127	1127	1040	9	2007	W
2184_1092_S1128	2011	D	19.05.2011	S1128	14.8	26.5	Fam-04_2007_	S1128	1128	1041	4	2007	W
2185_1093_S1129	2011	D	19.05.2011	S1129	15.2	28.5	Fam-03_2007_	S1129	1129	1042	3	2007	W
2185_1093_S1130	2011	D	19.05.2011	S1130	14.5	24.5	Fam-09_2007_	S1130	1130	1043	9	2007	W
2185_1093_S1131	2011	D	19.05.2011	S1131	13.9	22	Fam-04_2007_	S1131	1131	1044	4	2007	W
2185_1093_S1132	2011	D	19.05.2011	S1132	15.6	29	Fam-09_2007_	S1132	1132	1045	9	2007	W
2185_1093_S1133	2011	D	19.05.2011	S1133	14.2	23	Fam-17_2007_	S1133	1133	1046	17	2007	F
2185_1093_S1135	2011	D	19.05.2011	S1135	14.4	24.5	Fam-04_2007_	S1135	1135	1047	4	2007	W
2185_1093_S1136	2011	D	19.05.2011	S1136	14	22	Fam-17_2007_	S1136	1136	1048	17	2007	F
2185_1093_S1137	2011	D	19.05.2011	S1137	16.4	36.5	Fam-28_2007_	S1137	1137	1049	28	2007	H

2185_1093_S1138	2011	D	19.05.2011	S1138	14.8	27	Fam-09_2007_	S1138	1138	1050	9	2007	W
2185_1093_S1139	2011	D	19.05.2011	S1139	15.7	32	Fam-09_2007_	S1139	1139	1051	9	2007	W
2185_1093_S1140	2011	D	19.05.2011	S1140	15.2	27.5	Fam-10_2007_	S1140	1140	1052	10	2007	W
2185_1093_S1141	2011	D	19.05.2011	S1141	15	27	Fam-03_2007_	S1141	1141	1053	3	2007	W
2185_1093_S1142	2011	D	19.05.2011	S1142	14.5	24	Fam-28_2007_	S1142	1142	1054	28	2007	H
2185_1093_S1143	2011	D	19.05.2011	S1143	15	26	Fam-21_2007_	S1143	1143	1055	21	2007	F
2185_1093_S1144	2011	D	19.05.2011	S1144	14.4	23	Fam-04_2007_	S1144	1144	1056	4	2007	W
2185_1093_S1145	2011	D	20.05.2011	S1145	15.2	29.5	Fam-17_2007_	S1145	1145	1057	17	2007	F
2185_1093_S1146	2011	D	20.05.2011	S1146	17.9	45.5	Fam-04_2007_	S1146	1146	1058	4	2007	W
2185_1093_S1147	2011	D	20.05.2011	S1147	15.3	28	Fam-28_2007_	S1147	1147	1059	28	2007	H
2185_1093_S1148	2011	D	20.05.2011	S1148	14.5	25	Fam-17_2007_	S1148	1148	1060	17	2007	F
2185_1093_S1149	2011	D	20.05.2011	S1149	13.6	20.5	Fam-10_2007_	S1149	1149	1061	10	2007	W
2185_1093_S1150	2011	D	20.05.2011	S1150	14.7	25	Fam-09_2007_	S1150	1150	1062	9	2007	W
2185_1093_S1151	2011	D	20.05.2011	S1151	14.9	26.5	Fam-09_2007_	S1151	1151	1063	9	2007	W

2185_1093_S1153	2011	D	20.05.2011	S1153	15.6	30	Fam-11_2007_	S1153	1153	1064	11	2007	F
2185_1093_S1154	2011	D	20.05.2011	S1154	14.6	25	Fam-28_2007_	S1154	1154	1065	28	2007	H
2185_1093_S1156	2011	D	20.05.2011	S1156	14.5	27	Fam-04_2007_	S1156	1156	1066	4	2007	W
2185_1093_S1157	2011	D	20.05.2011	S1157	14.5	26.5	Fam-04_2007_	S1157	1157	1067	4	2007	W
2185_1093_S1158	2011	D	20.05.2011	S1158	15.9	32.5	Fam-05_2007_	S1158	1158	1068	5	2007	W
2185_1093_S1159	2011	D	20.05.2011	S1159	14.5	25.5	Fam-04_2007_	S1159	1159	1069	4	2007	W
2185_1093_S1160	2011	D	20.05.2011	S1160	14.7	25.5	Fam-17_2007_	S1160	1160	1070	17	2007	F
2185_1093_S1161	2011	D	20.05.2011	S1161	13.5	20.5	Fam-09_2007_	S1161	1161	1071	9	2007	W
2185_1093_S1162	2011	D	23.05.2011	S1162	14.8	26	Fam-25_2007_	S1162	1162	1072	25	2007	F
2185_1093_S1163	2011	D	23.05.2011	S1163	14.9	25.5	Fam-21_2007_	S1163	1163	1073	21	2007	F
2185_1093_S1164	2011	D	23.05.2011	S1164	15.5	31	Fam-25_2007_	S1164	1164	1074	25	2007	F
2185_1093_S1165	2011	D	23.05.2011	S1165	15	27.5	Fam-03_2007_	S1165	1165	1075	3	2007	W
2185_1093_S1166	2011	D	23.05.2011	S1166	15	26.5	Fam-07_2007_	S1166	1166	1076	7	2007	W
2185_1093_S1167	2011	D	23.05.2011	S1167	16.5	31	Fam-17_2007_	S1167	1167	1077	17	2007	F

2185_1093_S116[illegible]	2011	D	23.05.2011	S1168	14.8	26.5	Fam-09_2007_S1168	1168	1078	9	2007 W
2185_1093_S116[illegible]	2011	D	23.05.2011	S1169	14.5	23.5	Fam-03_2007_S1169	1169	1079	3	2007 W
2185_1093_S117[illegible]	2011	D	23.05.2011	S1170	15.3	29	Fam-04_2007_S1170	1170	1080	4	2007 W
2185_1093_S1171	2011	D	23.05.2011	S1171	14.5	23	Fam-30_2007_S1171	1171	1081	30	2007 H
2185_1093_S117[illegible]	2011	D	23.05.2011	S1172	14.2	23	Fam-10_2007_S1172	1172	1082	10	2007 W
2185_1093_S117[illegible]	2011	D	23.05.2011	S1173	14	24	Fam-09_2007_S1173	1173	1083	9	2007 W
2185_1093_S117[illegible]	2011	D	23.05.2011	S1174	14.5	25	Fam-05_2007_S1174	1174	1084	5	2007 W
2185_1093_S117[illegible]	2011	D	23.05.2011	S1175	14.5	26	Fam-25_2007_S1175	1175	1085	25	2007 F
2185_1093_S117[illegible]	2011	D	23.05.2011	S1176	14.5	23	Fam-28_2007_S1176	1176	1086	28	2007 H
2185_1093_S117[illegible]	2011	D	23.05.2011	S1177	15.5	29	Fam-11_2007_S1177	1177	1087	11	2007 F
2185_1093_S117[illegible]	2011	D	23.05.2011	S1178	15.6	32	Fam-05_2007_S1178	1178	1088	5	2007 W
2185_1093_S117[illegible]	2011	D	23.05.2011	S1179	15.3	25.5	Fam-17_2007_S1179	1179	1089	17	2007 F
2185_1093_S118[illegible]	2011	D	23.05.2011	S1180	14.7	26	Fam-04_2007_S1180	1180	1090	4	2007 W
2185_1093_S1181	2011	D	23.05.2011	S1181	14.3	24.5	Fam-03_2007_S1181	1181	1091	3	2007 W

2185_1093_S1182	2011	D	23.05.2011	S1182	16.8	38.5	Fam-21_2007_	S1182	1182	1092	21	2007	F
2185_1093_S1183	2011	D	23.05.2011	S1183	14.2	23	Fam-03_2007_	S1183	1183	1093	3	2007	W
2185_1093_S1184	2011	D	23.05.2011	S1184	16.9	40.5	Fam-07_2007_	S1184	1184	1094	7	2007	W
2185_1093_S1185	2011	D	23.05.2011	S1185	13.4	19	Fam-03_2007_	S1185	1185	1095	3	2007	W
2185_1093_S1187	2011	D	23.05.2011	S1187	14	23.5	Fam-05_2007_	S1187	1187	1096	5	2007	W
2185_1093_S1188	2011	D	23.05.2011	S1188	14	23.5	Fam-04_2007_	S1188	1188	1097	4	2007	W
2185_1093_S1189	2011	D	23.05.2011	S1189	14.7	25.5	Fam-04_2007_	S1189	1189	1098	4	2007	W
2185_1093_S1190	2011	D	23.05.2011	S1190	16.3	34	Fam-28_2007_	S1190	1190	1099	28	2007	H
2185_1093_S1191	2011	D	23.05.2011	S1191	13.5	21	Fam-25_2007_	S1191	1191	1100	25	2007	F
2185_1093_S1192	2011	D	23.05.2011	S1192	15	28	Fam-09_2007_	S1192	1192	1101	9	2007	W
2185_1093_S1193	2011	D	23.05.2011	S1193	15.2	27	Fam-25_2007_	S1193	1193	1102	25	2007	F
2185_1093_S1194	2011	D	23.05.2011	S1194	14.1	21.5	Fam-09_2007_	S1194	1194	1103	9	2007	W
2185_1093_S1195	2011	D	23.05.2011	S1195	14.4	24	Fam-09_2007_	S1195	1195	1104	9	2007	W
2185_1093_S1196	2011	D	23.05.2011	S1196	15.7	34	Fam-11_2007_	S1196	1196	1105	11	2007	F

2185_1093_S1197	2011	D	23.05.2011	S1197	14.3	23	Fam-09_2007_	S1197	1197	1106	9	2007	W
2185_1093_S1198	2011	D	23.05.2011	S1198	13.7	19.5	Fam-17_2007_	S1198	1198	1107	17	2007	F
2185_1093_S1200	2011	D	23.05.2011	S1200	13.7	22.5	Fam-10_2007_	S1200	1200	1108	10	2007	W
2186_1093_S1201	2011	D	23.05.2011	S1201	15.1	30	Fam-10_2007_	S1201	1201	1109	10	2007	W
2186_1093_S1202	2011	D	23.05.2011	S1202	15.7	29.5	Fam-09_2007_	S1202	1202	1110	9	2007	W
2186_1093_S1203	2011	D	23.05.2011	S1203	15.1	27	Fam-10_2007_	S1203	1203	1111	10	2007	W
2186_1093_S1204	2011	D	23.05.2011	S1204	14.9	26.5	Fam-03_2007_	S1204	1204	1112	3	2007	W
2186_1093_S1205	2011	D	23.05.2011	S1205	14.5	25.5	Fam-05_2007_	S1205	1205	1113	5	2007	W
2186_1093_S1206	2011	D	23.05.2011	S1206	14.7	25.5	Fam-11_2007_	S1206	1206	1114	11	2007	F
2186_1093_S1207	2011	D	23.05.2011	S1207	15	26.5	Fam-30_2007_	S1207	1207	1115	30	2007	H
2186_1093_S1208	2011	D	23.05.2011	S1208	15.2	29	Fam-25_2007_	S1208	1208	1116	25	2007	F
2186_1093_S1209	2011	D	23.05.2011	S1209	15.4	29.5	Fam-17_2007_	S1209	1209	1117	17	2007	F
2186_1093_S1210	2011	D	23.05.2011	S1210	15	26.5	Fam-25_2007_	S1210	1210	1118	25	2007	F
2186_1093_S1211	2011	D	23.05.2011	S1211	13.5	20	Fam-25_2007_	S1211	1211	1119	25	2007	F

2186_1093_S1212	2011	D	23.05.2011	S1212	15.5	30.5	Fam-25_2007_S1212	1212	1120	25	2007	F
2186_1093_S1213	2011	D	23.05.2011	S1213	17.9	44	Fam-30_2007_S1213	1213	1121	30	2007	H
2186_1093_S1214	2011	D	23.05.2011	S1214	15.1	28	Fam-03_2007_S1214	1214	1122	3	2007	W
2186_1093_S1215	2011	D	23.05.2011	S1215	15.5	28.5	Fam-17_2007_S1215	1215	1123	17	2007	F
2186_1093_S1216	2011	D	23.05.2011	S1216	13.7	20.5	Fam-23_2007_S1216	1216	1124	23	2007	F
2186_1093_S1217	2011	D	23.05.2011	S1217	14	21.5	Fam-19_2007_S1217	1217	1125	19	2007	F
2186_1093_S1218	2011	D	23.05.2011	S1218	15.7	29.5	Fam-03_2007_S1218	1218	1126	3	2007	W
2186_1093_S1219	2011	D	23.05.2011	S1219	14.3	22.5	Fam-09_2007_S1219	1219	1127	9	2007	W
2186_1093_S1220	2011	D	23.05.2011	S1220	14.8	26	Fam-11_2007_S1220	1220	1128	11	2007	F
2186_1093_S1221	2011	D	23.05.2011	S1221	13.1	18.5	Fam-05_2007_S1221	1221	1129	5	2007	W
2186_1093_S1222	2011	D	23.05.2011	S1222	13.7	21.5	Fam-25_2007_S1222	1222	1130	25	2007	F
2186_1094_S1223	2011	D	23.05.2011	S1223	15.2	28	Fam-27_2007_S1223	1223	1131	27	2007	F
2186_1094_S1224	2011	D	23.05.2011	S1224	16	33.5	Fam-04_2007_S1224	1224	1132	4	2007	W
2186_1094_S1225	2011	D	23.05.2011	S1225	14.9	26.5	Fam-09_2007_S1225	1225	1133	9	2007	W

2186_1094_S1226	2011	D	23.05.2011	S1226	14.7	25	Fam-09_2007_	S1226	1226	1134	9	2007	W
2186_1094_S1227	2011	D	23.05.2011	S1227	13.4	18	Fam-09_2007_	S1227	1227	1135	9	2007	W
2186_1094_S1228	2011	D	23.05.2011	S1228	14.6	23.5	Fam-23_2007_	S1228	1228	1136	23	2007	F
2186_1094_S1229	2011	D	23.05.2011	S1229	15.5	29.5	Fam-23_2007_	S1229	1229	1137	23	2007	F
2186_1094_S1230	2011	D	23.05.2011	S1230	14.1	23.5	Fam-05_2007_	S1230	1230	1138	5	2007	W
2186_1094_S1231	2011	D	23.05.2011	S1231	14.9	26.5	Fam-23_2007_	S1231	1231	1139	23	2007	F
2186_1094_S1232	2011	D	23.05.2011	S1232	16	31.5	Fam-09_2007_	S1232	1232	1140	9	2007	W
2186_1094_S1233	2011	D	23.05.2011	S1233	15	27	Fam-09_2007_	S1233	1233	1141	9	2007	W
2186_1094_S1234	2011	D	23.05.2011	S1234	12.2	16	Fam-17_2007_	S1234	1234	1142	17	2007	F
2186_1094_S1235	2011	D	23.05.2011	S1235	14.5	25	Fam-25_2007_	S1235	1235	1143	25	2007	F
2186_1094_S1236	2011	D	23.05.2011	S1236	15	24.5	Fam-03_2007_	S1236	1236	1144	3	2007	W
2186_1094_S1237	2011	D	23.05.2011	S1237	15.1	28.5	Fam-11_2007_	S1237	1237	1145	11	2007	F
2186_1094_S1238	2011	D	23.05.2011	S1238	14.6	24	Fam-09_2007_	S1238	1238	1146	9	2007	W
2186_1094_S1239	2011	D	23.05.2011	S1239	15.2	27.5	Fam-21_2007_	S1239	1239	1147	21	2007	F

2186_1094_S1241	2011	D	23.05.2011	S1241	13.6	20	Fam-09_2007_S1241	1241	1148	9	2007	W
2186_1094_S1242	2011	D	23.05.2011	S1242	14.9	26.5	Fam-11_2007_S1242	1242	1149	11	2007	F
2186_1094_S1243	2011	D	23.05.2011	S1243	15.2	28.5	Fam-04_2007_S1243	1243	1150	4	2007	W
2186_1094_S1244	2011	D	23.05.2011	S1244	13.9	22	Fam-17_2007_S1244	1244	1151	17	2007	F
2186_1094_S1245	2011	D	23.05.2011	S1245	14.6	25	Fam-25_2007_S1245	1245	1152	25	2007	F
2186_1094_S1246	2011	D	23.05.2011	S1246	14	23.5	Fam-27_2007_S1246	1246	1153	27	2007	F
2186_1094_S1247	2011	D	23.05.2011	S1247	15.7	28.5	Fam-03_2007_S1247	1247	1154	3	2007	W
2186_1094_S1248	2011	D	23.05.2011	S1248	14.5	24	Fam-07_2007_S1248	1248	1155	7	2007	W
2186_1094_S1249	2011	D	23.05.2011	S1249	14	22	Fam-05_2007_S1249	1249	1156	5	2007	W
2186_1094_S1250	2011	D	23.05.2011	S1250	15.8	29.5	Fam-17_2007_S1250	1250	1157	17	2007	F
2186_1094_S1251	2011	D	23.05.2011	S1251	13.7	20.5	Fam-19_2007_S1251	1251	1158	19	2007	F
2186_1094_S1252	2011	D	23.05.2011	S1252	16.2	36	Fam-05_2007_S1252	1252	1159	5	2007	W
2186_1094_S1253	2011	D	23.05.2011	S1253	15.1	26.5	Fam-17_2007_S1253	1253	1160	17	2007	F
2186_1094_S1254	2011	D	23.05.2011	S1254	16	34	Fam-25_2007_S1254	1254	1161	25	2007	F

2186_1094_S1255	2011	D	23.05.2011	S1255	14.5	23	Fam-03_2007_	S1255	1255	1162	3	2007	W
2186_1094_S1256	2011	D	23.05.2011	S1256	14.2	24	Fam-10_2007_	S1256	1256	1163	10	2007	W
2186_1094_S1257	2011	D	23.05.2011	S1257	15.5	29.5	Fam-03_2007_	S1257	1257	1164	3	2007	W
2186_1094_S1258	2011	T	23.05.2011	S1258	14.2	21	Family-17_200	S1258	1258	1165	17	2007	F
2186_1094_S1260	2011	D	23.05.2011	S1260	17.5	44	Fam-06_2007_	S1260	1260	1166	6	2007	W
2186_1094_S1261	2011	D	23.05.2011	S1261	13	17.5	Fam-06_2007_	S1261	1261	1167	6	2007	W
2186_1094_S1262	2011	D	23.05.2011	S1262	14.5	24.5	Fam-03_2007_	S1262	1262	1168	3	2007	W
2186_1094_S1263	2011	D	23.05.2011	S1263	15.3	28	Fam-10_2007_	S1263	1263	1169	10	2007	W
2186_1094_S1264	2011	D	23.05.2011	S1264	13.7	21	Fam-04_2007_	S1264	1264	1170	4	2007	W
2186_1094_S1265	2011	D	23.05.2011	S1265	14	23	Fam-05_2007_	S1265	1265	1171	5	2007	W
2186_1094_S1267	2011	D	23.05.2011	S1267	13.5	21	Fam-09_2007_	S1267	1267	1172	9	2007	W
2186_1094_S1268	2011	D	23.05.2011	S1268	15.1	27	Fam-23_2007_	S1268	1268	1173	23	2007	F
2186_1094_S1269	2011	T	23.05.2011	S1269	13.6	18.5	Family-17_200	S1269	1269	1174	17	2007	F
2186_1094_S1270	2011	D	23.05.2011	S1270	14.5	25	Fam-09_2007_	S1270	1270	1175	9	2007	W

2186_1094_S1271	2011	D	23.05.2011	S1271	14	21.5	Fam-03_2007_ S1271	1271	1176	3	2007	W
2186_1094_S1272	2011	D	23.05.2011	S1272	14	23.5	Fam-17_2007_ S1272	1272	1177	17	2007	F
2186_1094_S1273	2011	D	23.05.2011	S1273	15	26.5	Fam-03_2007_ S1273	1273	1178	3	2007	W
2186_1094_S1274	2011	D	23.05.2011	S1274	14	21.5	Fam-09_2007_ S1274	1274	1179	9	2007	W
2186_1094_S1275	2011	D	23.05.2011	S1275	14.7	27	Fam-04_2007_ S1275	1275	1180	4	2007	W
2186_1094_S1276	2011	D	23.05.2011	S1276	14.2	22.5	Fam-04_2007_ S1276	1276	1181	4	2007	W
2186_1094_S1278	2011	D	23.05.2011	S1278	14.1	21.5	Fam-03_2007_ S1278	1278	1182	3	2007	W
2186_1094_S1280	2011	D	23.05.2011	S1280	14.6	24.5	Fam-28_2007_ S1280	1280	1183	28	2007	H
2187_1094_S1282	2011	D	23.05.2011	S1282	17.7	42	Fam-21_2007_ S1282	1282	1184	21	2007	F
2187_1094_S1283	2011	D	23.05.2011	S1283	17.7	43.5	Fam-03_2007_ S1283	1283	1185	3	2007	W
2187_1094_S1284	2011	D	23.05.2011	S1284	14.5	21.5	Fam-09_2007_ S1284	1284	1186	9	2007	W
2187_1094_S1285	2011	D	23.05.2011	S1285	14.4	23	Fam-09_2007_ S1285	1285	1187	9	2007	W
2187_1094_S1286	2011	D	23.05.2011	S1286	17.2	38.5	Fam-06_2007_ S1286	1286	1188	6	2007	W
2187_1094_S1287	2011	D	23.05.2011	S1287	15.7	30	Fam-25_2007_ S1287	1287	1189	25	2007	F

2187_1094_S1289	2011	D	23.05.2011	S1289	15.1	27.5	Fam-09_2007_	S1289	1289	1190	9	2007	W
2187_1094_S1291	2011	D	23.05.2011	S1291	14.1	24	Fam-25_2007_	S1291	1291	1191	25	2007	F
2187_1094_S1292	2011	D	23.05.2011	S1292	14.7	26.5	Fam-09_2007_	S1292	1292	1192	9	2007	W
2187_1094_S1293	2011	D	23.05.2011	S1293	15.3	31	Fam-05_2007_	S1293	1293	1193	5	2007	W
2187_1094_S1296	2011	D	23.05.2011	S1296	15.5	29.5	Fam-03_2007_	S1296	1296	1194	3	2007	W
2187_1094_S1297	2011	D	23.05.2011	S1297	15.5	29	Fam-09_2007_	S1297	1297	1195	9	2007	W
2187_1094_S1298	2011	D	23.05.2011	S1298	16.2	31.5	Fam-28_2007_	S1298	1298	1196	28	2007	H
2187_1094_S1299	2011	D	23.05.2011	S1299	14.2	21.5	Fam-09_2007_	S1299	1299	1197	9	2007	W
2187_1094_S1300	2011	D	23.05.2011	S1300	16	31	Fam-04_2007_	S1300	1300	1198	4	2007	W
2187_1094_S1301	2011	D	23.05.2011	S1301	13.8	21	Fam-09_2007_	S1301	1301	1199	9	2007	W
2187_1094_S1302	2011	D	23.05.2011	S1302	15	26	Fam-28_2007_	S1302	1302	1200	28	2007	H
2187_1094_S1303	2011	D	23.05.2011	S1303	14.5	24	Fam-03_2007_	S1303	1303	1201	3	2007	W
2187_1094_S1304	2011	D	23.05.2011	S1304	13.5	19	Fam-28_2007_	S1304	1304	1202	28	2007	H
2187_1094_S1305	2011	D	23.05.2011	S1305	15.4	29.5	Fam-17_2007_	S1305	1305	1203	17	2007	F

2187_1094_S1306	2011	D	23.05.2011	S1306	13.3	18.5	Fam-27_2007_	S1306	1306	1204	27	2007	F
2187_1094_S1307	2011	D	23.05.2011	S1307	15.5	30.5	Fam-04_2007_	S1307	1307	1205	4	2007	W
2187_1094_S1308	2011	D	23.05.2011	S1308	15.2	28.5	Fam-03_2007_	S1308	1308	1206	3	2007	W
2187_1094_S1309	2011	D	23.05.2011	S1309	15.6	30.5	Fam-04_2007_	S1309	1309	1207	4	2007	W
2187_1094_S1310	2011	D	23.05.2011	S1310	15.9	31.5	Fam-03_2007_	S1310	1310	1208	3	2007	W
2187_1094_S1311	2011	D	23.05.2011	S1311	13.5	21.5	Fam-10_2007_	S1311	1311	1209	10	2007	W
2187_1094_S1312	2011	D	23.05.2011	S1312	15.7	30.5	Fam-17_2007_	S1312	1312	1210	17	2007	F
2187_1094_S1313	2011	D	23.05.2011	S1313	14	22.5	Fam-05_2007_	S1313	1313	1211	5	2007	W
2187_1094_S1314	2011	D	23.05.2011	S1314	13.9	19.5	Fam-03_2007_	S1314	1314	1212	3	2007	W
2187_1094_S1315	2011	D	23.05.2011	S1315	14.7	25	Fam-10_2007_	S1315	1315	1213	10	2007	W
2187_1094_S1316	2011	D	23.05.2011	S1316	16.1	34	Fam-09_2007_	S1316	1316	1214	9	2007	W
2187_1096_S1317	2011	D	23.05.2011	S1317	14.7	24	Fam-09_2007_	S1317	1317	1215	9	2007	W
2187_1096_S1318	2011	D	23.05.2011	S1318	14.7	28	Fam-09_2007_	S1318	1318	1216	9	2007	W
2187_1096_S1319	2011	D	23.05.2011	S1319	13.9	21.5	Fam-03_2007_	S1319	1319	1217	3	2007	W

2187_1096_S1320	2011	D	23.05.2011	S1320	14.8	25.5	Fam-03_2007_	S1320	1320	1218	3	2007	W
2187_1096_S1321	2011	D	23.05.2011	S1321	14.3	22	Fam-06_2007_	S1321	1321	1219	6	2007	W
2187_1096_S1322	2011	D	23.05.2011	S1322	14.5	25.5	Fam-05_2007_	S1322	1322	1220	5	2007	W
2187_1096_S1323	2011	D	23.05.2011	S1323	14.1	24	Fam-05_2007_	S1323	1323	1221	5	2007	W
2187_1096_S1324	2011	D	23.05.2011	S1324	14.7	27	Fam-09_2007_	S1324	1324	1222	9	2007	W
2187_1096_S1325	2011	D	23.05.2011	S1325	14.6	24.5	Fam-17_2007_	S1325	1325	1223	17	2007	F
2187_1096_S1326	2011	D	23.05.2011	S1326	15.6	29.5	Fam-06_2007_	S1326	1326	1224	6	2007	W
2187_1096_S1327	2011	D	23.05.2011	S1327	13.7	21.5	Fam-05_2007_	S1327	1327	1225	5	2007	W
2187_1096_S1328	2011	D	23.05.2011	S1328	16	30.5	Fam-03_2007_	S1328	1328	1226	3	2007	W
2187_1096_S1330	2011	D	23.05.2011	S1330	16.5	37.5	Fam-25_2007_	S1330	1330	1227	25	2007	F
2187_1096_S1331	2011	D	23.05.2011	S1331	13.2	19	Fam-03_2007_	S1331	1331	1228	3	2007	W
2187_1096_S1332	2011	D	23.05.2011	S1332	15	27	Fam-25_2007_	S1332	1332	1229	25	2007	F
2187_1096_S1333	2011	D	23.05.2011	S1333	14.9	25	Fam-03_2007_	S1333	1333	1230	3	2007	W
2187_1096_S1334	2011	D	23.05.2011	S1334	15.4	30.5	Fam-04_2007_	S1334	1334	1231	4	2007	W

2187_1096_S1335	2011	D	23.05.2011	S1335	15	27.5	Fam-25_2007_	S1335	1335	1232	25	2007	F
2187_1096_S1336	2011	D	23.05.2011	S1336	14.7	26.5	Fam-25_2007_	S1336	1336	1233	25	2007	F
2187_1096_S1337	2011	D	23.05.2011	S1337	14.5	22	Fam-03_2007_	S1337	1337	1234	3	2007	W
2187_1096_S1338	2011	D	23.05.2011	S1338	15	27.5	Fam-07_2007_	S1338	1338	1235	7	2007	W
2187_1096_S1339	2011	D	23.05.2011	S1339	13.6	20.5	Fam-09_2007_	S1339	1339	1236	9	2007	W
2187_1096_S1340	2011	D	23.05.2011	S1340	14.5	23.5	Fam-09_2007_	S1340	1340	1237	9	2007	W
2187_1096_S1341	2011	D	23.05.2011	S1341	14.5	25.5	Fam-11_2007_	S1341	1341	1238	11	2007	F
2187_1096_S1342	2011	D	23.05.2011	S1342	14.1	23.5	Fam-10_2007_	S1342	1342	1239	10	2007	W
2187_1096_S1343	2011	D	23.05.2011	S1343	14.5	24.5	Fam-05_2007_	S1343	1343	1240	5	2007	W
2187_1096_S1344	2011	D	23.05.2011	S1344	15.9	32	Fam-09_2007_	S1344	1344	1241	9	2007	W
2187_1096_S1345	2011	D	23.05.2011	S1345	16.8	40.5	Fam-25_2007_	S1345	1345	1242	25	2007	F
2187_1096_S1346	2011	D	23.05.2011	S1346	14.4	25	Fam-11_2007_	S1346	1346	1243	11	2007	F
2187_1096_S1347	2011	D	23.05.2011	S1347	15.2	29	Fam-09_2007_	S1347	1347	1244	9	2007	W
2187_1096_S1348	2011	D	23.05.2011	S1348	14.2	23.5	Fam-25_2007_	S1348	1348	1245	25	2007	F

2187_1096_S1349	2011	D	23.05.2011	S1349	15.9	34	Fam-25_2007_ S1349	1349	1246	25	2007	F
2187_1096_S1350	2011	D	23.05.2011	S1350	13	18.5	Fam-03_2007_ S1350	1350	1247	3	2007	W
2187_1096_S1351	2011	D	23.05.2011	S1351	14	22	Fam-09_2007_ S1351	1351	1248	9	2007	W
2187_1096_S1352	2011	D	23.05.2011	S1352	14.5	24	Fam-13_2007_ S1352	1352	1249	13	2007	F
2187_1096_S1353	2011	D	23.05.2011	S1353	14.3	23	Fam-09_2007_ S1353	1353	1250	9	2007	W
2187_1096_S1354	2011	D	23.05.2011	S1354	14.5	25.5	Fam-25_2007_ S1354	1354	1251	25	2007	F
2187_1096_S1355	2011	D	23.05.2011	S1355	14.9	25.5	Fam-10_2007_ S1355	1355	1252	10	2007	W
2187_1096_S1356	2011	D	23.05.2011	S1356	14.5	23.5	Fam-03_2007_ S1356	1356	1253	3	2007	W
2187_1096_S1357	2011	D	23.05.2011	S1357	15.3	29.5	Fam-09_2007_ S1357	1357	1254	9	2007	W
2187_1096_S1360	2011	D	23.05.2011	S1360	13.5	18.5	Fam-09_2007_ S1360	1360	1255	9	2007	W
2188_1096_S1361	2011	D	23.05.2011	S1361	15	27	Fam-25_2007_ S1361	1361	1256	25	2007	F
2188_1096_S1362	2011	D	23.05.2011	S1362	15.3	27	Fam-03_2007_ S1362	1362	1257	3	2007	W
2188_1096_S1363	2011	D	23.05.2011	S1363	14.2	22.5	Fam-03_2007_ S1363	1363	1258	3	2007	W
2188_1096_S1364	2011	D	23.05.2011	S1364	13.5	21.5	Fam-19_2007_ S1364	1364	1259	19	2007	F

2188_1096_S1365	2011	D	23.05.2011	S1365	14	22	Fam-03_2007_	S1365	1365	1260	3	2007	W
2188_1096_S1366	2011	D	23.05.2011	S1366	14	21	Fam-06_2007_	S1366	1366	1261	6	2007	W
2188_1096_S1367	2011	D	23.05.2011	S1367	13.9	21.5	Fam-09_2007_	S1367	1367	1262	9	2007	W
2188_1096_S1368	2011	D	23.05.2011	S1368	12.9	17.5	Fam-05_2007_	S1368	1368	1263	5	2007	W
2188_1096_S1369	2011	D	23.05.2011	S1369	16.8	36	Fam-23_2007_	S1369	1369	1264	23	2007	F
2188_1096_S1370	2011	D	23.05.2011	S1370	15.3	28.5	Fam-21_2007_	S1370	1370	1265	21	2007	F
2188_1096_S1371	2011	D	23.05.2011	S1371	14.4	22.5	Fam-28_2007_	S1371	1371	1266	28	2007	H
2188_1096_S1372	2011	D	23.05.2011	S1372	15.7	30	Fam-28_2007_	S1372	1372	1267	28	2007	H
2188_1096_S1373	2011	D	23.05.2011	S1373	14.7	24	Fam-09_2007_	S1373	1373	1268	9	2007	W
2188_1096_S1374	2011	D	23.05.2011	S1374	15.1	26	Fam-23_2007_	S1374	1374	1269	23	2007	F
2188_1096_S1375	2011	D	23.05.2011	S1375	13.6	20.5	Fam-11_2007_	S1375	1375	1270	11	2007	F
2188_1096_S1376	2011	D	23.05.2011	S1376	15	26.5	Fam-11_2007_	S1376	1376	1271	11	2007	F
2188_1096_S1377	2011	D	23.05.2011	S1377	15.5	31	Fam-25_2007_	S1377	1377	1272	25	2007	F
2188_1096_S1378	2011	D	23.05.2011	S1378	14.3	23.5	Fam-10_2007_	S1378	1378	1273	10	2007	W

2188_1096_S1379	2011	D	23.05.2011	S1379	13.9	22.5	Fam-10_2007_	S1379	1379	1274	10	2007	W
2188_1096_S1380	2011	D	23.05.2011	S1380	14.5	22.5	Fam-09_2007_	S1380	1380	1275	9	2007	W
2188_1096_S1381	2011	D	23.05.2011	S1381	14.5	24.5	Fam-21_2007_	S1381	1381	1276	21	2007	F
2188_1096_S1382	2011	D	23.05.2011	S1382	17	35.5	Fam-28_2007_	S1382	1382	1277	28	2007	H
2188_1096_S1383	2011	D	23.05.2011	S1383	14.1	23	Fam-27_2007_	S1383	1383	1278	27	2007	F
2188_1096_S1384	2011	D	23.05.2011	S1384	14.6	24.5	Fam-23_2007_	S1384	1384	1279	23	2007	F
2188_1096_S1385	2011	D	23.05.2011	S1385	14.5	25.5	Fam-05_2007_	S1385	1385	1280	5	2007	W
2188_1096_S1386	2011	D	24.05.2011	S1386	14	23.5	Fam-04_2007_	S1386	1386	1281	4	2007	W
2188_1096_S1387	2011	D	24.05.2011	S1387	14.6	26.5	Fam-04_2007_	S1387	1387	1282	4	2007	W
2188_1096_S1388	2011	D	24.05.2011	S1388	14.2	23	Fam-07_2007_	S1388	1388	1283	7	2007	W
2188_1096_S1389	2011	D	24.05.2011	S1389	14.5	22.5	Fam-07_2007_	S1389	1389	1284	7	2007	W
2188_1096_S1390	2011	D	24.05.2011	S1390	14.1	22	Fam-03_2007_	S1390	1390	1285	3	2007	W
2188_1096_S1391	2011	D	24.05.2011	S1391	14.7	26.5	Fam-04_2007_	S1391	1391	1286	4	2007	W
2188_1096_S1392	2011	D	24.05.2011	S1392	14.3	22	Fam-17_2007_	S1392	1392	1287	17	2007	F

2188_1096_S1393	2011	D	24.05.2011	S1393	15.5	28.5	Fam-09_2007_	S1393	1393	1288	9	2007	W
2188_1096_S1395	2011	D	24.05.2011	S1395	18.1	44	Fam-10_2007_	S1395	1395	1289	10	2007	W
2188_1096_S1397	2011	D	24.05.2011	S1397	15	25.5	Fam-09_2007_	S1397	1397	1290	9	2007	W
2188_1096_S1398	2011	D	24.05.2011	S1398	14.1	21.5	Fam-17_2007_	S1398	1398	1291	17	2007	F
2188_1096_S1399	2011	D	24.05.2011	S1399	15.1	27.5	Fam-09_2007_	S1399	1399	1292	9	2007	W
2188_1096_S1400	2011	D	24.05.2011	S1400	14.7	24.5	Fam-03_2007_	S1400	1400	1293	3	2007	W
2188_1096_S1401	2011	D	24.05.2011	S1401	15.4	28.5	Fam-10_2007_	S1401	1401	1294	10	2007	W
2188_1096_S1402	2011	D	24.05.2011	S1402	14.5	22.5	Fam-03_2007_	S1402	1402	1295	3	2007	W
2188_1096_S1403	2011	D	24.05.2011	S1403	14.4	23	Fam-17_2007_	S1403	1403	1296	17	2007	F
2188_1096_S1404	2011	D	24.05.2011	S1404	13.2	19.5	Fam-25_2007_	S1404	1404	1297	25	2007	F
2188_1096_S1405	2011	D	24.05.2011	S1405	15	25	Fam-03_2007_	S1405	1405	1298	3	2007	W
2188_1096_S1406	2011	D	24.05.2011	S1406	15	25	Fam-03_2007_	S1406	1406	1299	3	2007	W
2188_1096_S1407	2011	D	24.05.2011	S1407	14.1	22.5	Fam-09_2007_	S1407	1407	1300	9	2007	W
2188_1096_S1408	2011	D	24.05.2011	S1408	14.3	23	Fam-23_2007_	S1408	1408	1301	23	2007	F

2188_1096_S1409	2011	D	24.05.2011	S1409	14.2	22.5	Fam-03_2007_	S1409	1409	1302	3	2007	W
2188_1096_S1410	2011	D	24.05.2011	S1410	15	26	Fam-25_2007_	S1410	1410	1303	25	2007	F
2188_1097_S1411	2011	D	24.05.2011	S1411	14	22	Fam-28_2007_	S1411	1411	1304	28	2007	H
2188_1097_S1412	2011	D	24.05.2011	S1412	16.7	37	Fam-07_2007_	S1412	1412	1305	7	2007	W
2188_1097_S1413	2011	D	24.05.2011	S1413	13.4	18	Fam-09_2007_	S1413	1413	1306	9	2007	W
2188_1097_S1414	2011	D	24.05.2011	S1414	14.5	25	Fam-05_2007_	S1414	1414	1307	5	2007	W
2188_1097_S1415	2011	D	24.05.2011	S1415	14.9	27	Fam-05_2007_	S1415	1415	1308	5	2007	W
2188_1097_S1416	2011	D	24.05.2011	S1416	12.2	14.5	Fam-09_2007_	S1416	1416	1309	9	2007	W
2188_1097_S1417	2011	D	24.05.2011	S1417	12.5	15	Fam-06_2007_	S1417	1417	1310	6	2007	W
2188_1097_S1418	2011	D	24.05.2011	S1418	14.1	22	Fam-25_2007_	S1418	1418	1311	25	2007	F
2188_1097_S1420	2011	D	24.05.2011	S1420	13.8	21	Fam-04_2007_	S1420	1420	1312	4	2007	W
2188_1097_S1421	2011	D	24.05.2011	S1421	12.1	15	Fam-05_2007_	S1421	1421	1313	5	2007	W
2188_1097_S1422	2011	D	26.05.2011	S1422	15	27	Fam-04_2007_	S1422	1422	1314	4	2007	W
2188_1097_S1423	2011	D	31.05.2011	S1423	15.9	36	Fam-03_2007_	S1423	1423	1315	3	2007	W

2188_1097_S1424	2011	D	31.05.2011	S1424	15.6	32	Fam-05_2007_S1424	1424	1316	5	2007	W
2188_1097_S1425	2011	D	31.05.2011	S1425	14.8	26	Fam-04_2007_S1425	1425	1317	4	2007	W
2188_1097_S1426	2011	D	31.05.2011	S1426	14.8	27	Fam-10_2007_S1426	1426	1318	10	2007	W
2188_1097_S1427	2011	D	31.05.2011	S1427	14.5	24	Fam-25_2007_S1427	1427	1319	25	2007	F
2188_1097_S1428	2011	D	31.05.2011	S1428	14.5	25	Fam-25_2007_S1428	1428	1320	25	2007	F
2188_1097_S1429	2011	D	31.05.2011	S1429	14	21.5	Fam-03_2007_S1429	1429	1321	3	2007	W
2188_1097_S1430	2011	D	31.05.2011	S1430	13.7	20.5	Fam-03_2007_S1430	1430	1322	3	2007	W
2188_1097_S1431	2011	D	31.05.2011	S1431	13.6	21	Fam-05_2007_S1431	1431	1323	5	2007	W
2188_1097_S1432	2011	D	31.05.2011	S1432	14.1	22	Fam-04_2007_S1432	1432	1324	4	2007	W
2188_1097_S1433	2011	D	31.05.2011	S1433	15	26	Fam-04_2007_S1433	1433	1325	4	2007	W
2188_1097_S1434	2011	D	31.05.2011	S1434	13.8	21	Fam-05_2007_S1434	1434	1326	5	2007	W
2188_1097_S1435	2011	D	31.05.2011	S1435	14	21.5	Fam-07_2007_S1435	1435	1327	7	2007	W
2188_1097_S1436	2011	D	31.05.2011	S1436	13.8	21.5	Fam-05_2007_S1436	1436	1328	5	2007	W
2188_1097_S1437	2011	D	31.05.2011	S1437	16.2	34	Fam-09_2007_S1437	1437	1329	9	2007	W

2188_1097_S1438	2011	D	31.05.2011	S1438	14.7	25.5	Fam-05_2007_S1438	1438	1330	5	2007	W
2188_1097_S1439	2011	D	31.05.2011	S1439	16	33	Fam-07_2007_S1439	1439	1331	7	2007	W
2188_1097_S1440	2011	D	31.05.2011	S1440	15.5	27.5	Fam-07_2007_S1440	1440	1332	7	2007	W
2189_1097_S1441	2011	D	31.05.2011	S1441	14.8	25	Fam-03_2007_S1441	1441	1333	3	2007	W
2189_1097_S1442	2011	D	31.05.2011	S1442	13.5	20	Fam-05_2007_S1442	1442	1334	5	2007	W
2189_1097_S1443	2011	D	31.05.2011	S1443	15.4	29	Fam-07_2007_S1443	1443	1335	7	2007	W
2189_1097_S1444	2011	D	31.05.2011	S1444	14.4	23.5	Fam-05_2007_S1444	1444	1336	5	2007	W
2189_1097_S1445	2011	D	31.05.2011	S1445	15.2	27	Fam-07_2007_S1445	1445	1337	7	2007	W
2189_1097_S1446	2011	D	31.05.2011	S1446	15.1	28	Fam-05_2007_S1446	1446	1338	5	2007	W
2189_1097_S1447	2011	D	31.05.2011	S1447	14.7	26.5	Fam-05_2007_S1447	1447	1339	5	2007	W
2189_1097_S1448	2011	D	31.05.2011	S1448	14.1	22	Fam-07_2007_S1448	1448	1340	7	2007	W
2189_1097_S1449	2011	D	31.05.2011	S1449	14.7	25	Fam-05_2007_S1449	1449	1341	5	2007	W
2189_1097_S1450	2011	D	31.05.2011	S1450	14.2	23.5	Fam-04_2007_S1450	1450	1342	4	2007	W
2189_1097_S1451	2011	D	31.05.2011	S1451	17	40	Fam-05_2007_S1451	1451	1343	5	2007	W

2189_1097_S1452	2011	D	31.05.2011	S1452	15.7	28.5	Fam-03_2007_	S1452	1452	1344	3	2007	W
2189_1097_S1453	2011	D	31.05.2011	S1453	16.2	33	Fam-04_2007_	S1453	1453	1345	4	2007	W
2189_1097_S1454	2011	D	31.05.2011	S1454	14.4	22.5	Fam-06_2007_	S1454	1454	1346	6	2007	W
2189_1097_S1455	2011	D	31.05.2011	S1455	15.4	29	Fam-03_2007_	S1455	1455	1347	3	2007	W
2189_1097_S1456	2011	D	31.05.2011	S1456	14.5	25	Fam-05_2007_	S1456	1456	1348	5	2007	W
2189_1097_S1457	2011	D	31.05.2011	S1457	15.5	29.5	Fam-03_2007_	S1457	1457	1349	3	2007	W
2189_1097_S1458	2011	D	31.05.2011	S1458	14.7	24	Fam-10_2007_	S1458	1458	1350	10	2007	W
2189_1097_S1459	2011	D	31.05.2011	S1459	15.3	29	Fam-04_2007_	S1459	1459	1351	4	2007	W
2189_1097_S1460	2011	D	31.05.2011	S1460	14	21.5	Fam-30_2007_	S1460	1460	1352	30	2007	H
2189_1097_S1461	2011	D	31.05.2011	S1461	14	22	Fam-10_2007_	S1461	1461	1353	10	2007	W
2189_1097_S1462	2011	D	31.05.2011	S1462	14.3	24	Fam-17_2007_	S1462	1462	1354	17	2007	F
2189_1097_S1463	2011	D	31.05.2011	S1463	15.2	28.5	Fam-04_2007_	S1463	1463	1355	4	2007	W
2189_1097_S1464	2011	D	31.05.2011	S1464	15	20	Fam-06_2007_	S1464	1464	1356	6	2007	W
2189_1097_S1465	2011	D	31.05.2011	S1465	14.9	26	Fam-05_2007_	S1465	1465	1357	5	2007	W

2189_1097_S1466	2011	D	31.05.2011	S1466	16	32.5	Fam-05_2007_	S1466	1466	1358	5	2007	W
2189_1097_S1467	2011	D	31.05.2011	S1467	13.8	21.5	Fam-05_2007_	S1467	1467	1359	5	2007	W
2189_1097_S1468	2011	D	31.05.2011	S1468	13.8	22	Fam-30_2007_	S1468	1468	1360	30	2007	H
2189_1097_S1469	2011	D	03.06.2011	S1469	13.4	18	Fam-06_2007_	S1469	1469	1361	6	2007	W
2189_1097_S1470	2011	D	03.06.2011	S1470	15.5	28.5	Fam-04_2007_	S1470	1470	1362	4	2007	W
2189_1097_S1471	2011	D	03.06.2011	S1471	14	21	Fam-05_2007_	S1471	1471	1363	5	2007	W
2189_1097_S1474	2011	D	03.06.2011	S1474	16.2	33.5	Fam-07_2007_	S1474	1474	1364	7	2007	W
2189_1097_S1475	2011	D	03.06.2011	S1475	16.3	35	Fam-07_2007_	S1475	1475	1365	7	2007	W
2189_1097_S1476	2011	D	03.06.2011	S1476	15.9	30.5	Fam-07_2007_	S1476	1476	1366	7	2007	W
2189_1097_S1477	2011	D	03.06.2011	S1477	14	21	Fam-03_2007_	S1477	1477	1367	3	2007	W
2189_1097_S1478	2011	D	03.06.2011	S1478	14.3	22.5	Fam-06_2007_	S1478	1478	1368	6	2007	W
2189_1097_S1479	2011	D	03.06.2011	S1479	14.5	25.5	Fam-05_2007_	S1479	1479	1369	5	2007	W
2189_1097_S1480	2011	D	03.06.2011	S1480	15.3	27.5	Fam-04_2007_	S1480	1480	1370	4	2007	W
2189_1097_S1481	2011	D	03.06.2011	S1481	14.8	26	Fam-04_2007_	S1481	1481	1371	4	2007	W

2189_1097_S1482	2011	D	03.06.2011	S1482	14.6	25	Fam-05_2007_	S1482	1482	1372	5	2007	W
2189_1097_S1483	2011	D	03.06.2011	S1483	13.8	21	Fam-11_2007_	S1483	1483	1373	11	2007	F
2189_1097_S1484	2011	D	03.06.2011	S1484	13.9	21	Fam-04_2007_	S1484	1484	1374	4	2007	W
2189_1097_S1485	2011	D	03.06.2011	S1485	15	28	Fam-06_2007_	S1485	1485	1375	6	2007	W
2189_1097_S1486	2011	D	06.06.2011	S1486	14.5	26	Fam-04_2007_	S1486	1486	1376	4	2007	W
2189_1097_S1487	2011	D	06.06.2011	S1487	15	28	Fam-04_2007_	S1487	1487	1377	4	2007	W
2189_1097_S1488	2011	D	06.06.2011	S1488	15.4	28.5	Fam-04_2007_	S1488	1488	1378	4	2007	W
2189_1097_S1490	2011	D	07.06.2011	S1490	16	33	Fam-07_2007_	S1490	1490	1379	7	2007	W
2189_1097_S1491	2011	D	07.06.2011	S1491	14.5	23	Fam-03_2007_	S1491	1491	1380	3	2007	W
2189_1097_S1492	2011	D	07.06.2011	S1492	14.4	23	Fam-09_2007_	S1492	1492	1381	9	2007	W
2189_1097_S1493	2011	D	07.06.2011	S1493	15.5	31	Fam-07_2007_	S1493	1493	1382	7	2007	W
2189_1097_S1494	2011	D	07.06.2011	S1494	15.4	29	Fam-07_2007_	S1494	1494	1383	7	2007	W
2189_1097_S1495	2011	D	07.06.2011	S1495	16.1	32	Fam-07_2007_	S1495	1495	1384	7	2007	W
2189_1097_S1496	2011	D	07.06.2011	S1496	15.3	29	Fam-04_2007_	S1496	1496	1385	4	2007	W

2189_1097_S1497	2011	D	08.06.2011	S1497	14.3	23.5	Fam-04_2007_S1497	1497	1386	4	2007 W
2189_1097_S1498	2011	D	10.06.2011	S1498	15.5	29	Fam-04_2007_S1498	1498	1387	4	2007 W
2189_1097_S1499	2011	D	10.06.2011	S1499	15.1	29	Fam-25_2007_S1499	1499	1388	25	2007 F
2189_1097_S1500	2011	D	10.06.2011	S1500	17	41	Fam-05_2007_S1500	1500	1389	5	2007 W
2189_1097_S1501	2011	D	14.06.2011	S1501	13.7	20.5	Fam-07_2007_S1501	1501	1390	7	2007 W
2189_1097_S1502	2011	D	14.06.2011	S1502	15.9	31.5	Fam-05_2007_S1502	1502	1391	5	2007 W
2189_1097_S1503	2011	D	14.06.2011	S1503	16	33	Fam-07_2007_S1503	1503	1392	7	2007 W
2189_1097_S1504	2011	D	14.06.2011	S1504	16.5	34	Fam-07_2007_S1504	1504	1393	7	2007 W
2189_1108_S1505	2011	D	14.06.2011	S1505	15.2	29	Fam-05_2007_S1505	1505	1394	5	2007 W
2189_1108_S1506	2011	D	14.06.2011	S1506	14.5	22.5	Fam-03_2007_S1506	1506	1395	3	2007 W
2189_1108_S1507	2011	D	14.06.2011	S1507	14	20.5	Fam-05_2007_S1507	1507	1396	5	2007 W
2189_1108_S1508	2011	D	14.06.2011	S1508	14.3	23.5	Fam-05_2007_S1508	1508	1397	5	2007 W
2189_1108_S1509	2011	D	17.06.2011	S1509	15.4	29	Fam-05_2007_S1509	1509	1398	5	2007 W
2189_1108_S1510	2011	D	17.06.2011	S1510	15.2	27	Fam-07_2007_S1510	1510	1399	7	2007 W

2189_1108_S1511	2011	D	17.06.2011	S1511	14.9	24.5	Fam-07_2007_	S1511	1511	1400	7	2007	W
2189_1108_S1512	2011	D	17.06.2011	S1512	15.7	29.5	Fam-05_2007_	S1512	1512	1401	5	2007	W
2189_1108_S1513	2011	D	17.06.2011	S1513	14.8	22.5	Fam-05_2007_	S1513	1513	1402	5	2007	W
2189_1108_S1514	2011	D	17.06.2011	S1514	15	26.5	Fam-07_2007_	S1514	1514	1403	7	2007	W
2189_1108_S1515	2011	D	17.06.2011	S1515	14	22.5	Fam-05_2007_	S1515	1515	1404	5	2007	W
2189_1108_S1516	2011	D	17.06.2011	S1516	15.1	27	Fam-05_2007_	S1516	1516	1405	5	2007	W
2189_1108_S1517	2011	D	17.06.2011	S1517	15.1	26	Fam-09_2007_	S1517	1517	1406	9	2007	W
2189_1108_S1519	2011	D	17.06.2011	S1519	16.2	33	Fam-04_2007_	S1519	1519	1407	4	2007	W
2189_1108_S1520	2011	D	17.06.2011	S1520	16	29.5	Fam-03_2007_	S1520	1520	1408	3	2007	W
2189_1108_S1521	2011	D	17.06.2011	S1521	15.6	29.5	Fam-05_2007_	S1521	1521	1409	5	2007	W
2189_1108_S1522	2011	D	17.06.2011	S1522	16.4	34.5	Fam-07_2007_	S1522	1522	1410	7	2007	W
2189_1108_S1523	2011	D	17.06.2011	S1523	14.3	22	Fam-04_2007_	S1523	1523	1411	4	2007	W
2189_1108_S1524	2011	D	17.06.2011	S1524	14.6	24.5	Fam-05_2007_	S1524	1524	1412	5	2007	W
2189_1108_S1525	2011	D	17.06.2011	S1525	15.3	28	Fam-05_2007_	S1525	1525	1413	5	2007	W

2189_1108_S152(	2011	D	17.06.2011	S1526	16	32	Fam-30_2007_S1526	1526	1414	30	2007	H
2189_1108_S152;	2011	D	17.06.2011	S1527	14	21.5	Fam-05_2007_S1527	1527	1415	5	2007	W
2189_1108_S152{	2011	D	17.06.2011	S1528	14.5	24	Fam-05_2007_S1528	1528	1416	5	2007	W
2189_1108_S152(	2011	D	17.06.2011	S1529	14	21	Fam-06_2007_S1529	1529	1417	6	2007	W
2189_1108_S153(	2011	D	17.06.2011	S1530	14	23	Fam-05_2007_S1530	1530	1418	5	2007	W
2698_1510_S1_A(	2012	D	12.04.2012	S1	13.2	18	Fam-13_2007_S1	1	1419	13	2007	F
2698_1510_S2_B(	2012	D	12.04.2012	S2	16.2	32	Fam-21_2007_S2	2	1420	21	2007	F
2698_1510_S3_C(	2012	D	24.04.2012	S3	14.9	26	Fam-37_2009_S3	3	1421	37	2009	F
2698_1510_S5_E(	2012	D	27.04.2012	S5	14.3	24	Fam-27_2007_S5	5	1423	27	2007	F
2698_1510_S6_F(	2012	D	27.04.2012	S6	14.4	24	Fam-21_2007_S6	6	1424	21	2007	F
2698_1510_S7_G(	2012	D	27.04.2012	S7	15.8	34	Fam-19_2007_S7	7	1425	19	2007	F
2698_1510_S8_H(	2012	D	27.04.2012	S8	15.2	28	Fam-11_2007_S8	8	1426	11	2007	F
2698_1510_S9_A(	2012	D	30.04.2012	S9	15.2	30	Fam-09_2007_S9	9	1427	9	2007	W
2698_1510_S10_E	2012	D	30.04.2012	S10	17.5	40	Fam-23_2007_S10	10	1428	23	2007	F

2698_1510_S11_C	2012	D	30.04.2012	S11	14.5	24.5	Fam-25_2007_	S11	11	1429	25	2007	F
2698_1510_S12_D	2012	D	30.04.2012	S12	14.6	25	Fam-28_2007_	S12	12	1430	28	2007	H
2698_1510_S13_E	2012	D	30.04.2012	S13	14.8	24.5	Fam-23_2007_	S13	13	1431	23	2007	F
2698_1510_S14_F	2012	D	30.04.2012	S14	15.7	30	Fam-11_2007_	S14	14	1432	11	2007	F
2698_1510_S15_G	2012	D	30.04.2012	S15	15.1	26.5	Fam-19_2007_	S15	15	1433	19	2007	F
2698_1510_S16_H	2012	D	30.04.2012	S16	14	22	Fam-51_2009_	S16	16	1434	51	2009	W
2698_1510_S17_A	2012	D	30.04.2012	S17	15	25	Fam-21_2007_	S17	17	1435	21	2007	F
2698_1510_S18_B	2012	D	30.04.2012	S18	15.3	30	Fam-09_2007_	S18	18	1436	9	2007	W
2698_1510_S19_C	2012	D	30.04.2012	S19	15.9	32	Fam-43_2009_	S19	19	1437	43	2009	F
2698_1510_S20_D	2012	D	30.04.2012	S20	15	28	Fam-19_2007_	S20	20	1438	19	2007	F
2698_1510_S21_E	2012	D	30.04.2012	S21	15.8	30	Fam-17_2007_	S21	21	1439	17	2007	F
2698_1510_S22_F	2012	D	30.04.2012	S22	15.5	28	Fam-10_2007_	S22	22	1440	10	2007	W
2698_1510_S23_G	2012	D	30.04.2012	S23	13.6	15.5	Fam-56_2009_	S23	23	1441	56	2009	W
2698_1510_S24_H	2012	D	30.04.2012	S24	15.1	27.5	Fam-11_2007_	S24	24	1442	11	2007	F

2698_1510_S25_A	2012	D	30.04.2012	S25	17.3	44	Fam-13_2007_	S25	25	1443	13	2007	F
2698_1510_S26_B	2012	D	03.05.2012	S26	14.6	27	Fam-13_2007_	S26	26	1444	13	2007	F
2698_1510_S27_C	2012	D	03.05.2012	S27	15.4	28	Fam-21_2007_	S27	27	1445	21	2007	F
2698_1510_S28_D	2012	D	03.05.2012	S28	15.1	27	Fam-27_2007_	S28	28	1446	27	2007	F
2698_1510_S29_E	2012	T	03.05.2012	S29	13.8	18	Family-27_200	S29	29	1447	27	2007	F
2698_1510_S30_F	2012	D	03.05.2012	S30	13.5	19.5	Fam-03_2007_	S30	30	1448	3	2007	W
2698_1510_S31_G	2012	D	03.05.2012	S31	16.5	35	Fam-04_2007_	S31	31	1449	4	2007	W
2698_1510_S32_H	2012	D	03.05.2012	S32	15.5	28	Fam-09_2007_	S32	32	1450	9	2007	W
2698_1510_S33_A	2012	D	03.05.2012	S33	13.2	18	Fam-19_2007_	S33	33	1451	19	2007	F
2698_1510_S34_B	2012	D	03.05.2012	S34	13.9	19	Fam-17_2007_	S34	34	1452	17	2007	F
2698_1510_S35_C	2012	D	03.05.2012	S35	16	34	Fam-21_2007_	S35	35	1453	21	2007	F
2698_1510_S36_D	2012	D	03.05.2012	S36	15.9	32.5	Fam-04_2007_	S36	36	1454	4	2007	W
2698_1510_S37_E	2012	D	03.05.2012	S37	14.9	27.5	Fam-13_2007_	S37	37	1455	13	2007	F
2698_1510_S38_F	2012	D	03.05.2012	S38	14.7	26	Fam-13_2007_	S38	38	1456	13	2007	F

2698_1510_S39_(	2012	D	03.05.2012	S39	15.2	27.5	Fam-09_2007_	S39	39	1457	9	2007	W
2698_1510_S40_I	2012	D	03.05.2012	S40	15.4	30	Fam-17_2007_	S40	40	1458	17	2007	F
2698_1510_S41_/	2012	D	03.05.2012	S41	15.4	28.5	Fam-03_2007_	S41	41	1459	3	2007	W
2698_1510_S42_E	2012	D	03.05.2012	S42	12.8	18	Fam-44_2009_	S42	42	1460	44	2009	H
2698_1510_S43_(	2012	D	03.05.2012	S43	15.2	27.5	Fam-28_2007_	S43	43	1461	28	2007	H
2698_1510_S44_[	2012	T	03.05.2012	S44	14.2	22	Family-28_200	S44	44	1462	28	2007	H
2698_1510_S45_E	2012	D	03.05.2012	S45	15.1	27	Fam-11_2007_	S45	45	1463	11	2007	F
2698_1510_S46_F	2012	D	03.05.2012	S46	14.4	24	Fam-28_2007_	S46	46	1464	28	2007	H
2698_1510_S47_(	2012	D	03.05.2012	S47	15.5	30.5	Fam-27_2007_	S47	47	1465	27	2007	F
2698_1510_S48_I	2012	D	03.05.2012	S48	16.2	34	Fam-17_2007_	S48	48	1466	17	2007	F
2698_1510_S49_/	2012	D	03.05.2012	S49	15.3	29.5	Fam-19_2007_	S49	49	1467	19	2007	F
2698_1510_S50_E	2012	D	03.05.2012	S50	14	22.5	Fam-09_2007_	S50	50	1468	9	2007	W
2698_1510_S51_(	2012	D	03.05.2012	S51	13.6	21.5	Fam-44_2009_	S51	51	1469	44	2009	H
2698_1510_S53_E	2012	D	08.05.2012	S53	15	32	Fam-28_2007_	S53	53	1470	28	2007	H

2698_1510_S54_F	2012	D	08.05.2012	S54	15.5	29.5	Fam-04_2007_	S54	54	1471	4	2007	W
2698_1510_S55_G	2012	D	08.05.2012	S55	14.5	24	Fam-13_2007_	S55	55	1472	13	2007	F
2698_1510_S56_H	2012	D	08.05.2012	S56	17.2	40	Fam-09_2007_	S56	56	1473	9	2007	W
2698_1510_S57_A	2012	D	08.05.2012	S57	16.7	37	Fam-04_2007_	S57	57	1474	4	2007	W
2698_1510_S58_B	2012	D	08.05.2012	S58	15.5	29.5	Fam-09_2007_	S58	58	1475	9	2007	W
2698_1510_S59_C	2012	D	08.05.2012	S59	13.8	21	Fam-39_2009_	S59	59	1476	39	2009	F
2698_1510_S60_D	2012	D	08.05.2012	S60	16.2	32.5	Fam-19_2007_	S60	60	1477	19	2007	F
2698_1510_S61_E	2012	D	08.05.2012	S61	14.9	24.5	Fam-28_2007_	S61	61	1478	28	2007	H
2698_1510_S62_F	2012	D	08.05.2012	S62	15.5	30.5	Fam-09_2007_	S62	62	1479	9	2007	W
2698_1510_S63_G	2012	D	08.05.2012	S63	15.5	29.5	Fam-13_2007_	S63	63	1480	13	2007	F
2698_1510_S64_H	2012	D	08.05.2012	S64	14.2	24	Fam-19_2007_	S64	64	1481	19	2007	F
2698_1510_S65_A	2012	D	08.05.2012	S65	15.4	29	Fam-09_2007_	S65	65	1482	9	2007	W
2698_1510_S66_B	2012	D	08.05.2012	S66	14.4	23.5	Fam-09_2007_	S66	66	1483	9	2007	W
2698_1510_S67_C	2012	D	09.05.2012	S67	15.1	28	Fam-19_2007_	S67	67	1484	19	2007	F

2698_1510_S69_[illegible]	2012	D	11.05.2012	S69	15.5	29.5	Fam-09_2007_S69	69	1485	9	2007	W
2698_1510_S70_[illegible]	2012	D	11.05.2012	S70	16.9	39	Fam-19_2007_S70	70	1486	19	2007	F
2698_1510_S71_[illegible]	2012	D	11.05.2012	S71	17.1	39	Fam-11_2007_S71	71	1487	11	2007	F
2698_1510_S72_[illegible]	2012	D	11.05.2012	S72	15.2	28	Fam-13_2007_S72	72	1488	13	2007	F
2698_1510_S73_[illegible]	2012	D	11.05.2012	S73	15.3	27	Fam-11_2007_S73	73	1489	11	2007	F
2698_1510_S74_[illegible]	2012	D	11.05.2012	S74	14.5	24	Fam-21_2007_S74	74	1490	21	2007	F
2698_1510_S75_[illegible]	2012	D	11.05.2012	S75	16.1	33	Fam-03_2007_S75	75	1491	3	2007	W
2698_1510_S76_[illegible]	2012	D	11.05.2012	S76	18	46.5	Fam-09_2007_S76	76	1492	9	2007	W
2698_1510_S77_[illegible]	2012	D	11.05.2012	S77	15	29	Fam-13_2007_S77	77	1493	13	2007	F
2698_1510_S78_[illegible]	2012	D	11.05.2012	S78	14.1	22	Fam-11_2007_S78	78	1494	11	2007	F
2698_1510_S79_[illegible]	2012	D	11.05.2012	S79	14.6	25	Fam-09_2007_S79	79	1495	9	2007	W
2698_1510_S80_[illegible]	2012	D	11.05.2012	S80	14.4	24	Fam-03_2007_S80	80	1496	3	2007	W
2699_1510_S81_[illegible]	2012	D	11.05.2012	S81	16.4	32.5	Fam-03_2007_S81	81	1497	3	2007	W
2699_1510_S82_[illegible]	2012	D	14.05.2012	S82	15.3	26	Fam-13_2007_S82	82	1498	13	2007	F

2699_1510_S83_D	2012	D	14.05.2012	S83	15.1	26.5	Fam-03_2007_	S83	83	1499	3	2007	W
2699_1510_S84_E	2012	D	14.05.2012	S84	15.4	28.5	Fam-09_2007_	S84	84	1500	9	2007	W
2699_1510_S85_F	2012	D	14.05.2012	S85	15.8	32	Fam-19_2007_	S85	85	1501	19	2007	F
2699_1510_S86_G	2012	D	14.05.2012	S86	15.2	26	Fam-03_2007_	S86	86	1502	3	2007	W
2699_1510_S87_H	2012	D	14.05.2012	S87	15.1	25.5	Fam-50_2009_	S87	87	1503	50	2009	H
2699_1510_S88_A	2012	D	14.05.2012	S88	13.8	19.5	Fam-06_2007_	S88	88	1504	6	2007	W
2699_1510_S89_E	2012	D	14.05.2012	S89	14.5	25	Fam-05_2007_	S89	89	1505	5	2007	W
2699_1510_S90_D	2012	D	14.05.2012	S90	15.5	29	Fam-10_2007_	S90	90	1506	10	2007	W
2699_1510_S91_E	2012	D	14.05.2012	S91	14.5	23.5	Fam-10_2007_	S91	91	1507	10	2007	W
2699_1510_S92_F	2012	D	14.05.2012	S92	13.7	20	Fam-04_2007_	S92	92	1508	4	2007	W
2699_1510_S93_G	2012	D	14.05.2012	S93	16.2	32	Fam-19_2007_	S93	93	1509	19	2007	F
2699_1510_S94_H	2012	D	14.05.2012	S94	13.5	20	Fam-09_2007_	S94	94	1510	9	2007	W
2699_1511_S95_A	2012	D	14.05.2012	S95	15	28	Fam-10_2007_	S95	95	1511	10	2007	W
2699_1511_S96_E	2012	D	14.05.2012	S96	14.3	24	Fam-13_2007_	S96	96	1512	13	2007	F

2699_1511_S97_C	2012	D	14.05.2012	S97	14.2	21.5	Fam-10_2007_	S97	97	1513	10	2007	W
2699_1511_S98_D	2012	D	14.05.2012	S98	14.6	24	Fam-50_2009_	S98	98	1514	50	2009	H
2699_1511_S99_E	2012	D	14.05.2012	S99	14.7	25	Fam-44_2009_	S99	99	1515	44	2009	H
2699_1511_S100_	2012	D	14.05.2012	S100	14.5	25	Fam-09_2007_	S100	100	1516	9	2007	W
2699_1511_S101_	2012	D	14.05.2012	S101	14.5	24.5	Fam-17_2007_	S101	101	1517	17	2007	F
2699_1511_S102_	2012	D	14.05.2012	S102	15.2	28.5	Fam-04_2007_	S102	102	1518	4	2007	W
2699_1511_S103_	2012	D	14.05.2012	S103	16.1	35.5	Fam-04_2007_	S103	103	1519	4	2007	W
2699_1511_S104_	2012	D	14.05.2012	S104	15.5	29	Fam-39_2009_	S104	104	1520	39	2009	F
2699_1511_S105_	2012	D	14.05.2012	S105	15	27	Fam-28_2007_	S105	105	1521	28	2007	H
2699_1511_S106_	2012	D	14.05.2012	S106	16	32.5	Fam-10_2007_	S106	106	1522	10	2007	W
2699_1511_S107_	2012	D	14.05.2012	S107	15.2	28.5	Fam-03_2007_	S107	107	1523	3	2007	W
2699_1511_S108_	2012	D	14.05.2012	S108	14.4	23.5	Fam-17_2007_	S108	108	1524	17	2007	F
2699_1511_S109_	2012	D	14.05.2012	S109	13.9	20.5	Fam-03_2007_	S109	109	1525	3	2007	W
2699_1511_S110_	2012	D	14.05.2012	S110	16.1	35	Fam-09_2007_	S110	110	1526	9	2007	W

2699_1511_S111_	2012	D	14.05.2012	S111	14.9	24.5	Fam-03_2007_	S111	111	1527	3	2007	W
2699_1511_S112_	2012	D	14.05.2012	S112	16.7	39	Fam-03_2007_	S112	112	1528	3	2007	W
2699_1511_S113_	2012	D	14.05.2012	S113	16.1	33.5	Fam-09_2007_	S113	113	1529	9	2007	W
2699_1511_S114_	2012	D	14.05.2012	S114	15.8	32	Fam-04_2007_	S114	114	1530	4	2007	W
2699_1511_S115_	2012	D	14.05.2012	S115	14.9	27.5	Fam-04_2007_	S115	115	1531	4	2007	W
2699_1511_S116_	2012	D	14.05.2012	S116	14.8	26.5	Fam-04_2007_	S116	116	1532	4	2007	W
2699_1511_S117_	2012	D	14.05.2012	S117	13.8	21	Fam-10_2007_	S117	117	1533	10	2007	W
2699_1511_S118_	2012	D	14.05.2012	S118	14.9	26.5	Fam-10_2007_	S118	118	1534	10	2007	W
2699_1511_S119_	2012	D	14.05.2012	S119	16.1	32.5	Fam-27_2007_	S119	119	1535	27	2007	F
2699_1511_S120_	2012	D	14.05.2012	S120	15.9	31	Fam-09_2007_	S120	120	1536	9	2007	W
2699_1511_S121_	2012	D	14.05.2012	S121	15	27.5	Fam-09_2007_	S121	121	1537	9	2007	W
2699_1511_S122_	2012	D	14.05.2012	S122	13.5	18.5	Fam-17_2007_	S122	122	1538	17	2007	F
2699_1511_S123_	2012	D	14.05.2012	S123	14.6	25	Fam-10_2007_	S123	123	1539	10	2007	W
2699_1511_S124_	2012	D	14.05.2012	S124	14.7	24.5	Fam-17_2007_	S124	124	1540	17	2007	F

2699_1511_S125_	2012	D	14.05.2012	S125	14.8	25.5	Fam-09_2007_	S125	125	1541	9	2007	W
2699_1511_S126_	2012	D	14.05.2012	S126	14.6	25.5	Fam-44_2009_	S126	126	1542	44	2009	H
2699_1511_S127_	2012	D	14.05.2012	S127	14.7	24	Fam-03_2007_	S127	127	1543	3	2007	W
2699_1511_S128_	2012	D	14.05.2012	S128	15.1	27.5	Fam-09_2007_	S128	128	1544	9	2007	W
2699_1511_S129_	2012	D	14.05.2012	S129	16.3	35.5	Fam-06_2007_	S129	129	1545	6	2007	W
2699_1511_S130_	2012	D	14.05.2012	S130	15.2	28.5	Fam-09_2007_	S130	130	1546	9	2007	W
2699_1511_S131_	2012	D	14.05.2012	S131	14.6	25.5	Fam-21_2007_	S131	131	1547	21	2007	F
2699_1511_S132_	2012	D	14.05.2012	S132	15.4	28	Fam-21_2007_	S132	132	1548	21	2007	F
2699_1511_S133_	2012	D	14.05.2012	S133	14.8	25	Fam-04_2007_	S133	133	1549	4	2007	W
2699_1511_S134_	2012	D	14.05.2012	S134	15.5	28.5	Fam-09_2007_	S134	134	1550	9	2007	W
2699_1511_S135_	2012	D	14.05.2012	S135	15.1	28.5	Fam-03_2007_	S135	135	1551	3	2007	W
2699_1511_S136_	2012	D	14.05.2012	S136	14.7	25	Fam-21_2007_	S136	136	1552	21	2007	F
2699_1511_S137_	2012	D	14.05.2012	S137	15.5	27	Fam-17_2007_	S137	137	1553	17	2007	F
2699_1511_S138_	2012	D	14.05.2012	S138	16.3	35.5	Fam-09_2007_	S138	138	1554	9	2007	W

2699_1511_S139_	2012	D	14.05.2012	S139	14.2	22.5	Fam-09_2007_S139	139	1555	9	2007	W
2699_1511_S140_	2012	D	14.05.2012	S140	15.2	29.5	Fam-04_2007_S140	140	1556	4	2007	W
2699_1511_S141_	2012	D	14.05.2012	S141	14.1	21.5	Fam-17_2007_S141	141	1557	17	2007	F
2699_1511_S142_	2012	D	14.05.2012	S142	15.4	29	Fam-10_2007_S142	142	1558	10	2007	W
2699_1511_S143_	2012	D	14.05.2012	S143	14.7	25	Fam-09_2007_S143	143	1559	9	2007	W
2699_1511_S144_	2012	D	14.05.2012	S144	13.9	22	Fam-27_2007_S144	144	1560	27	2007	F
2699_1511_S145_	2012	D	14.05.2012	S145	15.1	28.5	Fam-04_2007_S145	145	1561	4	2007	W
2699_1511_S146_	2012	D	14.05.2012	S146	14.3	24	Fam-09_2007_S146	146	1562	9	2007	W
2699_1511_S147_	2012	D	14.05.2012	S147	13.9	22	Fam-03_2007_S147	147	1563	3	2007	W
2699_1511_S148_	2012	D	14.05.2012	S148	17.3	41.5	Fam-04_2007_S148	148	1564	4	2007	W
2699_1511_S149_	2012	D	14.05.2012	S149	15.6	29	Fam-03_2007_S149	149	1565	3	2007	W
2699_1511_S150_	2012	D	14.05.2012	S150	13.2	17.5	Fam-39_2009_S150	150	1566	39	2009	F
2699_1511_S151_	2012	D	14.05.2012	S151	13.8	20.5	Fam-10_2007_S151	151	1567	10	2007	W
2699_1511_S152_	2012	D	14.05.2012	S152	13.9	21.5	Fam-10_2007_S152	152	1568	10	2007	W

2699_1511_S153_	2012	D	14.05.2012	S153	14.7	24.5	Fam-03_2007_	S153	153	1569	3	2007 W
2699_1511_S154_	2012	D	14.05.2012	S154	13.6	18	Fam-10_2007_	S154	154	1570	10	2007 W
2699_1511_S155_	2012	D	14.05.2012	S155	15.6	30.5	Fam-10_2007_	S155	155	1571	10	2007 W
2699_1511_S156_	2012	D	14.05.2012	S156	15	25.5	Fam-03_2007_	S156	156	1572	3	2007 W
2699_1511_S157_	2012	D	14.05.2012	S157	12.4	14	Fam-10_2007_	S157	157	1573	10	2007 W
2699_1511_S158_	2012	D	14.05.2012	S158	14.9	26.5	Fam-25_2007_	S158	158	1574	25	2007 F
2699_1511_S159_	2012	D	14.05.2012	S159	15.8	30.5	Fam-09_2007_	S159	159	1575	9	2007 W
2699_1511_S160_	2012	D	14.05.2012	S160	13.9	21	Fam-27_2007_	S160	160	1576	27	2007 F
2700_1511_S162_	2012	D	14.05.2012	S162	14.4	24	Fam-10_2007_	S162	162	1577	10	2007 W
2700_1511_S163_	2012	D	14.05.2012	S163	13.8	18.5	Fam-21_2007_	S163	163	1578	21	2007 F
2700_1511_S164_	2012	T	14.05.2012	S164	13.3	16.5	Family-23_200	S164	164	1579	23	2007 F
2700_1511_S165_	2012	D	14.05.2012	S165	14.5	24	Fam-06_2007_	S165	165	1580	6	2007 W
2700_1511_S166_	2012	D	14.05.2012	S166	15.2	27	Fam-03_2007_	S166	166	1581	3	2007 W
2700_1511_S167_	2012	D	14.05.2012	S167	15.9	32	Fam-10_2007_	S167	167	1582	10	2007 W

2700_1511_S168_	2012	D	14.05.2012	S168	13.7	21.5	Fam-09_2007_	S168	168	1583	9	2007	W
2700_1511_S169_	2012	D	14.05.2012	S169	15	26.5	Fam-03_2007_	S169	169	1584	3	2007	W
2700_1511_S170_	2012	D	14.05.2012	S170	14.2	23	Fam-27_2007_	S170	170	1585	27	2007	F
2700_1511_S172_	2012	D	14.05.2012	S172	14.6	24.5	Fam-10_2007_	S172	172	1586	10	2007	W
2700_1511_S173_	2012	D	14.05.2012	S173	15.7	30.5	Fam-03_2007_	S173	173	1587	3	2007	W
2700_1511_S174_	2012	D	14.05.2012	S174	15.1	28	Fam-28_2007_	S174	174	1588	28	2007	H
2700_1511_S175_	2012	D	14.05.2012	S175	15.1	27.5	Fam-17_2007_	S175	175	1589	17	2007	F
2700_1511_S176_	2012	D	14.05.2012	S176	14.9	26.5	Fam-28_2007_	S176	176	1590	28	2007	H
2700_1511_S177_	2012	D	14.05.2012	S177	15.7	31.5	Fam-04_2007_	S177	177	1591	4	2007	W
2700_1511_S178_	2012	D	14.05.2012	S178	13.5	20	Fam-25_2007_	S178	178	1592	25	2007	F
2700_1511_S179_	2012	D	14.05.2012	S179	15.5	29	Fam-28_2007_	S179	179	1593	28	2007	H
2700_1511_S180_	2012	D	14.05.2012	S180	13.8	21	Fam-05_2007_	S180	180	1594	5	2007	W
2700_1511_S181_	2012	D	14.05.2012	S181	14.8	23.5	Fam-05_2007_	S181	181	1595	5	2007	W
2700_1511_S182_	2012	D	14.05.2012	S182	13.9	20.5	Fam-03_2007_	S182	182	1596	3	2007	W

2700_1511_S183_	2012	D	14.05.2012	S183	14.3	24.5	Fam-10_2007_S183	183	1597	10	2007	W
2700_1511_S184_	2012	D	14.05.2012	S184	15.3	28.5	Fam-09_2007_S184	184	1598	9	2007	W
2700_1511_S185_	2012	D	14.05.2012	S185	15.2	27.5	Fam-23_2007_S185	185	1599	23	2007	F
2700_1511_S186_	2012	D	14.05.2012	S186	15.4	29.5	Fam-11_2007_S186	186	1600	11	2007	F
2700_1511_S187_	2012	D	14.05.2012	S187	15.8	31.5	Fam-04_2007_S187	187	1601	4	2007	W
2700_1511_S188_	2012	D	14.05.2012	S188	14.8	26	Fam-58_2009_S188	188	1602	58	2009	W
2700_1512_S189_	2012	D	14.05.2012	S189	15.2	29	Fam-10_2007_S189	189	1603	10	2007	W
2700_1512_S191_	2012	D	14.05.2012	S191	14.7	25	Fam-09_2007_S191	191	1604	9	2007	W
2700_1512_S192_	2012	D	14.05.2012	S192	13.2	17	Fam-17_2007_S192	192	1605	17	2007	F
2700_1512_S193_	2012	D	14.05.2012	S193	13.6	19.5	Fam-09_2007_S193	193	1606	9	2007	W
2700_1512_S194_	2012	D	14.05.2012	S194	15.1	28	Fam-09_2007_S194	194	1607	9	2007	W
2700_1512_S195_	2012	D	14.05.2012	S195	15.2	27.5	Fam-09_2007_S195	195	1608	9	2007	W
2700_1512_S196_	2012	D	14.05.2012	S196	14.5	23.5	Fam-03_2007_S196	196	1609	3	2007	W
2700_1512_S197_	2012	D	14.05.2012	S197	16.1	32.5	Fam-13_2007_S197	197	1610	13	2007	F

2700_1512_S198_	2012	D	14.05.2012	S198	14.1	23.5	Fam-09_2007_	S198	198	1611	9	2007 W
2700_1512_S199_	2012	D	14.05.2012	S199	15.4	29.5	Fam-11_2007_	S199	199	1612	11	2007 F
2700_1512_S200_	2012	D	14.05.2012	S200	14.7	25.5	Fam-10_2007_	S200	200	1613	10	2007 W
2700_1512_S201_	2012	D	14.05.2012	S201	15.5	31.5	Fam-13_2007_	S201	201	1614	13	2007 F
2700_1512_S202_	2012	D	14.05.2012	S202	15	27.5	Fam-11_2007_	S202	202	1615	11	2007 F
2700_1512_S203_	2012	D	14.05.2012	S203	15.4	28.5	Fam-10_2007_	S203	203	1616	10	2007 W
2700_1512_S204_	2012	D	14.05.2012	S204	14.5	24.5	Fam-03_2007_	S204	204	1617	3	2007 W
2700_1512_S205_	2012	T	14.05.2012	S205	14.1	20	Family-28_200	S205	205	1618	28	2007 H
2700_1512_S206_	2012	D	14.05.2012	S206	14.1	23	Fam-04_2007_	S206	206	1619	4	2007 W
2700_1512_S207_	2012	D	14.05.2012	S207	14.9	26	Fam-03_2007_	S207	207	1620	3	2007 W
2700_1512_S208_	2012	D	14.05.2012	S208	15.9	31.5	Fam-03_2007_	S208	208	1621	3	2007 W
2700_1512_S210_	2012	D	14.05.2012	S210	13.9	22.5	Fam-51_2009_	S210	210	1622	51	2009 W
2700_1512_S211_	2012	D	14.05.2012	S211	15.8	30.5	Fam-09_2007_	S211	211	1623	9	2007 W
2700_1512_S212_	2012	D	14.05.2012	S212	15.3	28.5	Fam-11_2007_	S212	212	1624	11	2007 F

2700_1512_S213_	2012	D	14.05.2012	S213	16.9	35.5	Fam-28_2007_	S213	213	1625	28	2007	H
2700_1512_S214_	2012	D	14.05.2012	S214	15.5	29	Fam-10_2007_	S214	214	1626	10	2007	W
2700_1512_S215_	2012	D	14.05.2012	S215	14.2	23	Fam-17_2007_	S215	215	1627	17	2007	F
2700_1512_S216_	2012	D	14.05.2012	S216	15.3	27.5	Fam-10_2007_	S216	216	1628	10	2007	W
2700_1512_S217_	2012	D	14.05.2012	S217	13.7	22.5	Fam-10_2007_	S217	217	1629	10	2007	W
2700_1512_S218_	2012	D	14.05.2012	S218	14.8	24.5	Fam-17_2007_	S218	218	1630	17	2007	F
2700_1512_S219_	2012	D	14.05.2012	S219	16.6	38	Fam-04_2007_	S219	219	1631	4	2007	W
2700_1512_S221_	2012	D	14.05.2012	S221	15.4	29.5	Fam-04_2007_	S221	221	1632	4	2007	W
2700_1512_S222_	2012	D	14.05.2012	S222	15.2	29.5	Fam-04_2007_	S222	222	1633	4	2007	W
2700_1512_S223_	2012	D	14.05.2012	S223	13.9	21.5	Fam-23_2007_	S223	223	1634	23	2007	F
2700_1512_S224_	2012	D	14.05.2012	S224	15.8	34	Fam-09_2007_	S224	224	1635	9	2007	W
2700_1512_S225_	2012	D	14.05.2012	S225	14.7	25	Fam-09_2007_	S225	225	1636	9	2007	W
2700_1512_S226_	2012	D	14.05.2012	S226	16.2	34.5	Fam-09_2007_	S226	226	1637	9	2007	W
2700_1512_S227_	2012	D	14.05.2012	S227	15.4	28.5	Fam-10_2007_	S227	227	1638	10	2007	W

2700_1512_S228_	2012	D	14.05.2012	S228	15	27.5	Fam-13_2007_S228	228	1639	13	2007	F
2700_1512_S229_	2012	D	14.05.2012	S229	15.5	30.5	Fam-03_2007_S229	229	1640	3	2007	W
2700_1512_S230_	2012	D	14.05.2012	S230	14.5	24	Fam-03_2007_S230	230	1641	3	2007	W
2700_1512_S231_	2012	D	14.05.2012	S231	15.1	28	Fam-09_2007_S231	231	1642	9	2007	W
2700_1512_S232_	2012	D	14.05.2012	S232	15.4	31.5	Fam-10_2007_S232	232	1643	10	2007	W
2700_1512_S233_	2012	D	14.05.2012	S233	14	23	Fam-25_2007_S233	233	1644	25	2007	F
2700_1512_S234_	2012	D	14.05.2012	S234	14.1	20.5	Fam-51_2009_S234	234	1645	51	2009	W
2700_1512_S235_	2012	D	14.05.2012	S235	14.3	25	Fam-03_2007_S235	235	1646	3	2007	W
2700_1512_S236_	2012	D	14.05.2012	S236	15.3	29	Fam-03_2007_S236	236	1647	3	2007	W
2700_1512_S237_	2012	D	14.05.2012	S237	15.6	33	Fam-05_2007_S237	237	1648	5	2007	W
2700_1512_S238_	2012	D	14.05.2012	S238	16.1	32.5	Fam-03_2007_S238	238	1649	3	2007	W
2700_1512_S239_	2012	D	14.05.2012	S239	14.7	25	Fam-06_2007_S239	239	1650	6	2007	W
2700_1512_S240_	2012	D	14.05.2012	S240	16.9	39	Fam-03_2007_S240	240	1651	3	2007	W
2701_1512_S241_	2012	D	14.05.2012	S241	15.9	33.5	Fam-04_2007_S241	241	1652	4	2007	W

2701_1512_S242_	2012	D	14.05.2012	S242	15.3	28.5	Fam-10_2007_ S242	242	1653	10	2007	W
2701_1512_S243_	2012	D	14.05.2012	S243	15.1	27.5	Fam-03_2007_ S243	243	1654	3	2007	W
2701_1512_S244_	2012	D	14.05.2012	S244	15.1	27.5	Fam-03_2007_ S244	244	1655	3	2007	W
2701_1512_S245_	2012	D	14.05.2012	S245	15.6	30	Fam-09_2007_ S245	245	1656	9	2007	W
2701_1512_S246_	2012	D	14.05.2012	S246	13.8	23	Fam-05_2007_ S246	246	1657	5	2007	W
2701_1512_S247_	2012	D	14.05.2012	S247	13.5	19.5	Fam-13_2007_ S247	247	1658	13	2007	F
2701_1512_S248_	2012	D	14.05.2012	S248	14	22.5	Fam-10_2007_ S248	248	1659	10	2007	W
2701_1512_S249_	2012	D	14.05.2012	S249	14.7	28.5	Fam-05_2007_ S249	249	1660	5	2007	W
2701_1512_S251_	2012	D	14.05.2012	S251	16.2	35	Fam-13_2007_ S251	251	1661	13	2007	F
2701_1512_S252_	2012	D	14.05.2012	S252	14.5	24	Fam-04_2007_ S252	252	1662	4	2007	W
2701_1512_S253_	2012	D	14.05.2012	S253	14.5	24.5	Fam-10_2007_ S253	253	1663	10	2007	W
2701_1512_S254_	2012	D	14.05.2012	S254	14.1	24	Fam-28_2007_ S254	254	1664	28	2007	H
2701_1512_S256_	2012	D	14.05.2012	S256	13.1	19	Fam-03_2007_ S256	256	1665	3	2007	W
2701_1512_S257_	2012	D	14.05.2012	S257	14.4	30	Fam-09_2007_ S257	257	1666	9	2007	W

2701_1512_S258_	2012	D	14.05.2012	S258	15.1	28.5	Fam-44_2009_S258	258	1667	44	2009	H
2701_1512_S259_	2012	D	14.05.2012	S259	16.1	34	Fam-04_2007_S259	259	1668	4	2007	W
2701_1512_S260_	2012	D	14.05.2012	S260	14.3	23.5	Fam-04_2007_S260	260	1669	4	2007	W
2701_1512_S261_	2012	D	14.05.2012	S261	17.5	42	Fam-04_2007_S261	261	1670	4	2007	W
2701_1512_S262_	2012	D	14.05.2012	S262	16.1	32	Fam-03_2007_S262	262	1671	3	2007	W
2701_1512_S263_	2012	D	14.05.2012	S263	13.8	20.5	Fam-03_2007_S263	263	1672	3	2007	W
2701_1512_S264_	2012	D	14.05.2012	S264	15.9	32	Fam-04_2007_S264	264	1673	4	2007	W
2701_1512_S265_	2012	D	14.05.2012	S265	15.2	31	Fam-04_2007_S265	265	1674	4	2007	W
2701_1512_S266_	2012	D	14.05.2012	S266	15.2	28	Fam-04_2007_S266	266	1675	4	2007	W
2701_1512_S267_	2012	D	14.05.2012	S267	13.6	19	Fam-03_2007_S267	267	1676	3	2007	W
2701_1512_S268_	2012	D	14.05.2012	S268	14.5	23	Fam-03_2007_S268	268	1677	3	2007	W
2701_1512_S269_	2012	D	14.05.2012	S269	14.8	23	Fam-03_2007_S269	269	1678	3	2007	W
2701_1512_S270_	2012	D	14.05.2012	S270	15.1	27	Fam-13_2007_S270	270	1679	13	2007	F
2701_1512_S271_	2012	D	14.05.2012	S271	16.1	35	Fam-28_2007_S271	271	1680	28	2007	H

2701_1512_S272_	2012	D	14.05.2012	S272	14	21.5	Fam-03_2007_	S272	272	1681	3	2007	W
2701_1512_S273_	2012	D	14.05.2012	S273	15.1	27.5	Fam-09_2007_	S273	273	1682	9	2007	W
2701_1512_S274_	2012	D	14.05.2012	S274	15	25.5	Fam-19_2007_	S274	274	1683	19	2007	F
2701_1512_S275_	2012	D	14.05.2012	S275	16.6	35.5	Fam-04_2007_	S275	275	1684	4	2007	W
2701_1512_S276_	2012	D	14.05.2012	S276	16	31.5	Fam-09_2007_	S276	276	1685	9	2007	W
2701_1512_S277_	2012	D	14.05.2012	S277	13.9	20.5	Fam-10_2007_	S277	277	1686	10	2007	W
2701_1512_S278_	2012	D	14.05.2012	S278	14.5	23	Fam-09_2007_	S278	278	1687	9	2007	W
2701_1512_S279_	2012	D	14.05.2012	S279	14.1	23.5	Fam-04_2007_	S279	279	1688	4	2007	W
2701_1512_S280_	2012	D	14.05.2012	S280	15	28	Fam-10_2007_	S280	280	1689	10	2007	W
2701_1512_S281_	2012	D	14.05.2012	S281	16	34	Fam-21_2007_	S281	281	1690	21	2007	F
2701_1512_S282_	2012	D	14.05.2012	S282	14.5	25.5	Fam-04_2007_	S282	282	1691	4	2007	W
2701_1513_S284_	2012	D	14.05.2012	S284	14.7	25	Fam-09_2007_	S284	284	1692	9	2007	W
2701_1513_S285_	2012	D	14.05.2012	S285	14.6	25.5	Fam-10_2007_	S285	285	1693	10	2007	W
2701_1513_S286_	2012	D	14.05.2012	S286	14.7	25.5	Fam-09_2007_	S286	286	1694	9	2007	W

2701_1513_S287_	2012	D	15.05.2012	S287	13.4	25.5	Fam-03_2007_S287	287	1695	3	2007	W
2701_1513_S288_	2012	D	15.05.2012	S288	11.8	20.5	Fam-06_2007_S288	288	1696	6	2007	W
2701_1513_S289_	2012	D	15.05.2012	S289	14.3	29.5	Fam-03_2007_S289	289	1697	3	2007	W
2701_1513_S290_	2012	D	15.05.2012	S290	13.7	31.5	Fam-03_2007_S290	290	1698	3	2007	W
2701_1513_S291_	2012	D	15.05.2012	S291	12.3	18.5	Fam-52_2009_S291	291	1699	52	2009	W
2701_1513_S292_	2012	D	15.05.2012	S292	13.6	24.5	Fam-04_2007_S292	292	1700	4	2007	W
2701_1513_S293_	2012	D	15.05.2012	S293	13.8	31	Fam-05_2007_S293	293	1701	5	2007	W
2701_1513_S294_	2012	D	15.05.2012	S294	14.5	29	Fam-04_2007_S294	294	1702	4	2007	W
2701_1513_S295_	2012	D	15.05.2012	S295	12.4	17.5	Fam-06_2007_S295	295	1703	6	2007	W
2701_1513_S296_	2012	D	15.05.2012	S296	15.3	34.5	Fam-09_2007_S296	296	1704	9	2007	W
2701_1513_S297_	2012	D	15.05.2012	S297	12.9	23.5	Fam-04_2007_S297	297	1705	4	2007	W
2701_1513_S298_	2012	D	15.05.2012	S298	12.7	22.5	Fam-10_2007_S298	298	1706	10	2007	W
2701_1513_S299_	2012	D	15.05.2012	S299	13.6	27	Fam-04_2007_S299	299	1707	4	2007	W
2701_1513_S300_	2012	D	15.05.2012	S300	14.2	29.5	Fam-13_2007_S300	300	1708	13	2007	F

2701_1513_S301_	2012	D	15.05.2012	S301	12.7	19	Fam-52_2009_	S301	301	1709	52	2009	W
2701_1513_S302_	2012	D	15.05.2012	S302	13.9	27	Fam-04_2007_	S302	302	1710	4	2007	W
2701_1513_S303_	2012	D	15.05.2012	S303	12.9	26	Fam-10_2007_	S303	303	1711	10	2007	W
2701_1513_S304_	2012	D	15.05.2012	S304	13.2	24.5	Fam-09_2007_	S304	304	1712	9	2007	W
2701_1513_S305_	2012	D	15.05.2012	S305	14.3	31	Fam-10_2007_	S305	305	1713	10	2007	W
2701_1513_S306_	2012	D	15.05.2012	S306	13.6	26.5	Fam-03_2007_	S306	306	1714	3	2007	W
2701_1513_S307_	2012	D	15.05.2012	S307	16.9	49	Fam-03_2007_	S307	307	1715	3	2007	W
2701_1513_S308_	2012	D	15.05.2012	S308	12.3	19	Fam-05_2007_	S308	308	1716	5	2007	W
2701_1513_S309_	2012	D	15.05.2012	S309	13.8	28	Fam-05_2007_	S309	309	1717	5	2007	W
2701_1513_S311_	2012	D	15.05.2012	S311	12.3	22.5	Fam-10_2007_	S311	311	1718	10	2007	W
2701_1513_S312_	2012	D	15.05.2012	S312	14.4	31	Fam-04_2007_	S312	312	1719	4	2007	W
2701_1513_S313_	2012	D	15.05.2012	S313	14.7	32.5	Fam-10_2007_	S313	313	1720	10	2007	W
2701_1513_S314_	2012	D	15.05.2012	S314	14.6	31	Fam-03_2007_	S314	314	1721	3	2007	W
2701_1513_S315_	2012	D	15.05.2012	S315	12.6	26.5	Fam-03_2007_	S315	315	1722	3	2007	W

2701_1513_S316_	2012	D	15.05.2012	S316	16.6	45	Fam-03_2007_S316	316	1723	3	2007	W
2701_1513_S317_	2012	D	15.05.2012	S317	14.6	31.5	Fam-03_2007_S317	317	1724	3	2007	W
2701_1513_S318_	2012	D	15.05.2012	S318	13.8	26	Fam-28_2007_S318	318	1725	28	2007	H
2701_1513_S319_	2012	D	15.05.2012	S319	15.5	38	Fam-09_2007_S319	319	1726	9	2007	W
2702_1513_S321_	2012	D	15.05.2012	S321	14.6	33.5	Fam-10_2007_S321	321	1728	10	2007	W
2702_1513_S322_	2012	D	15.05.2012	S322	14.1	29	Fam-11_2007_S322	322	1729	11	2007	F
2702_1513_S323_	2012	D	15.05.2012	S323	12.9	23.5	Fam-10_2007_S323	323	1730	10	2007	W
2702_1513_S324_	2012	D	15.05.2012	S324	14.2	29	Fam-09_2007_S324	324	1731	9	2007	W
2702_1513_S325_	2012	D	15.05.2012	S325	13.2	23.5	Fam-28_2007_S325	325	1732	28	2007	H
2702_1513_S326_	2012	D	15.05.2012	S326	14.2	32	Fam-04_2007_S326	326	1733	4	2007	W
2702_1513_S327_	2012	D	15.05.2012	S327	13.7	29	Fam-04_2007_S327	327	1734	4	2007	W
2702_1513_S328_	2012	D	15.05.2012	S328	14.1	28.5	Fam-13_2007_S328	328	1735	13	2007	F
2702_1513_S329_	2012	D	15.05.2012	S329	15.6	41	Fam-09_2007_S329	329	1736	9	2007	W
2702_1513_S330_	2012	D	15.05.2012	S330	13.3	24.5	Fam-03_2007_S330	330	1737	3	2007	W

2702_1513_S331_	2012	D	15.05.2012	S331	14.6	30.5	Fam-21_2007_	S331	331	1738	21	2007	F
2702_1513_S332_	2012	D	15.05.2012	S332	12.9	21.5	Fam-10_2007_	S332	332	1739	10	2007	W
2702_1513_S333_	2012	D	15.05.2012	S333	16.8	45	Fam-03_2007_	S333	333	1740	3	2007	W
2702_1513_S334_	2012	D	15.05.2012	S334	14.7	31	Fam-13_2007_	S334	334	1741	13	2007	F
2702_1513_S335_	2012	D	15.05.2012	S335	13.4	23	Fam-10_2007_	S335	335	1742	10	2007	W
2702_1513_S336_	2012	D	15.05.2012	S336	13.6	29	Fam-05_2007_	S336	336	1743	5	2007	W
2702_1513_S337_	2012	D	15.05.2012	S337	14.8	33	Fam-09_2007_	S337	337	1744	9	2007	W
2702_1513_S338_	2012	D	15.05.2012	S338	10.7	14.5	Fam-62_1_201	S338	338	1745	62	2010	H
2702_1513_S339_	2012	D	15.05.2012	S339	13.7	27.5	Fam-09_2007_	S339	339	1746	9	2007	W
2702_1513_S340_	2012	D	15.05.2012	S340	13.8	29.5	Fam-25_2007_	S340	340	1747	25	2007	F
2702_1513_S341_	2012	D	15.05.2012	S341	13.9	29.5	Fam-10_2007_	S341	341	1748	10	2007	W
2702_1513_S342_	2012	D	15.05.2012	S342	14.7	33.5	Fam-28_2007_	S342	342	1749	28	2007	H
2702_1513_S343_	2012	D	15.05.2012	S343	12.8	22	Fam-10_2007_	S343	343	1750	10	2007	W
2702_1513_S344_	2012	D	15.05.2012	S344	12.3	19	Fam-19_2007_	S344	344	1751	19	2007	F

2702_1513_S345_	2012	D	15.05.2012	S345	10.7	13	Fam-44_2009_	S345	345	1752	44	2009	H
2702_1513_S346_	2012	D	15.05.2012	S346	15.6	39	Fam-04_2007_	S346	346	1753	4	2007	W
2702_1513_S347_	2012	D	15.05.2012	S347	13.3	23	Fam-17_2007_	S347	347	1754	17	2007	F
2702_1513_S348_	2012	D	15.05.2012	S348	12.6	19.5	Fam-09_2007_	S348	348	1755	9	2007	W
2702_1513_S349_	2012	D	15.05.2012	S349	13.8	27.5	Fam-09_2007_	S349	349	1756	9	2007	W
2702_1513_S350_	2012	D	15.05.2012	S350	13.7	27	Fam-28_2007_	S350	350	1757	28	2007	H
2702_1513_S351_	2012	D	15.05.2012	S351	12.7	21.5	Fam-03_2007_	S351	351	1758	3	2007	W
2702_1513_S352_	2012	D	15.05.2012	S352	13.3	24	Fam-51_2009_	S352	352	1759	51	2009	W
2702_1513_S353_	2012	D	15.05.2012	S353	14.3	31.5	Fam-09_2007_	S353	353	1760	9	2007	W
2702_1513_S354_	2012	D	15.05.2012	S354	15.3	37	Fam-13_2007_	S354	354	1761	13	2007	F
2702_1513_S355_	2012	D	15.05.2012	S355	13.8	27	Fam-09_2007_	S355	355	1762	9	2007	W
2702_1513_S356_	2012	D	15.05.2012	S356	15.3	35.5	Fam-04_2007_	S356	356	1763	4	2007	W
2702_1513_S357_	2012	D	15.05.2012	S357	14.3	31.5	Fam-03_2007_	S357	357	1764	3	2007	W
2702_1513_S358_	2012	D	15.05.2012	S358	14.1	29	Fam-19_2007_	S358	358	1765	19	2007	F

2702_1513_S359_	2012	D	15.05.2012	S359	13.4	25	Fam-04_2007_S359	359	1766	4	2007	W
2702_1513_S360_	2012	D	15.05.2012	S360	14.2	29	Fam-03_2007_S360	360	1767	3	2007	W
2702_1513_S362_	2012	D	15.05.2012	S362	13.7	27	Fam-03_2007_S362	362	1768	3	2007	W
2702_1513_S363_	2012	D	15.05.2012	S363	13.8	26	Fam-03_2007_S363	363	1769	3	2007	W
2702_1513_S364_	2012	D	15.05.2012	S364	14.2	30	Fam-10_2007_S364	364	1770	10	2007	W
2702_1513_S365_	2012	D	21.05.2012	S365	14.9	24.5	Fam-03_2007_S365	365	1771	3	2007	W
2702_1513_S366_	2012	D	21.05.2012	S366	15.6	28.5	Fam-10_2007_S366	366	1772	10	2007	W
2702_1513_S367_	2012	D	21.05.2012	S367	15.7	29	Fam-03_2007_S367	367	1773	3	2007	W
2702_1513_S368_	2012	D	21.05.2012	S368	14.5	23.5	Fam-09_2007_S368	368	1774	9	2007	W
2702_1513_S370_	2012	D	21.05.2012	S370	15.8	32	Fam-09_2007_S370	370	1776	9	2007	W
2702_1513_S371_	2012	D	21.05.2012	S371	15.3	28.5	Fam-04_2007_S371	371	1777	4	2007	W
2702_1513_S372_	2012	D	21.05.2012	S372	15.9	32.5	Fam-03_2007_S372	372	1778	3	2007	W
2702_1513_S373_	2012	D	21.05.2012	S373	15.1	28	Fam-13_2007_S373	373	1779	13	2007	F
2702_1513_S374_	2012	D	21.05.2012	S374	13.9	20.5	Fam-52_2009_S374	374	1780	52	2009	W

2702_1513_S375_	2012	D	21.05.2012	S375	15.1	27	Fam-03_2007_S375	375	1781	3	2007	W
2702_1513_S376_	2012	D	21.05.2012	S376	15.9	31	Fam-03_2007_S376	376	1782	3	2007	W
2702_1515_S377_	2012	D	21.05.2012	S377	15	24	Fam-21_2007_S377	377	1783	21	2007	F
2702_1515_S378_	2012	D	21.05.2012	S378	16.2	33	Fam-03_2007_S378	378	1784	3	2007	W
2702_1515_S379_	2012	D	21.05.2012	S379	15.4	29.5	Fam-09_2007_S379	379	1785	9	2007	W
2702_1515_S380_	2012	D	21.05.2012	S380	14.5	24	Fam-09_2007_S380	380	1786	9	2007	W
2702_1515_S381_	2012	D	21.05.2012	S381	15.3	27	Fam-03_2007_S381	381	1787	3	2007	W
2702_1515_S382_	2012	D	21.05.2012	S382	15.6	31	Fam-28_2007_S382	382	1788	28	2007	H
2702_1515_S383_	2012	D	21.05.2012	S383	13.9	20	Fam-50_2009_S383	383	1789	50	2009	H
2702_1515_S384_	2012	D	21.05.2012	S384	16	30	Fam-09_2007_S384	384	1790	9	2007	W
2702_1515_S385_	2012	D	21.05.2012	S385	15.5	28	Fam-11_2007_S385	385	1791	11	2007	F
2702_1515_S386_	2012	D	21.05.2012	S386	15.9	31.5	Fam-04_2007_S386	386	1792	4	2007	W
2702_1515_S387_	2012	D	21.05.2012	S387	13.8	21.5	Fam-05_2007_S387	387	1793	5	2007	W
2702_1515_S388_	2012	D	21.05.2012	S388	14.6	25.5	Fam-10_2007_S388	388	1794	10	2007	W

2702_1515_S389_	2012	D	21.05.2012	S389	15.4	28.5	Fam-03_2007_	S389	389	1795	3	2007 W
2702_1515_S390_	2012	D	21.05.2012	S390	15.5	29.5	Fam-04_2007_	S390	390	1796	4	2007 W
2702_1515_S391_	2012	D	21.05.2012	S391	14.1	23	Fam-10_2007_	S391	391	1797	10	2007 W
2702_1515_S392_	2012	T	21.05.2012	S392	12.6	14	Family-13_200	S392	392	1798	13	2007 F
2702_1515_S393_	2012	D	21.05.2012	S393	14.7	23.5	Fam-03_2007_	S393	393	1799	3	2007 W
2702_1515_S395_	2012	D	21.05.2012	S395	15.3	28.5	Fam-09_2007_	S395	395	1801	9	2007 W
2702_1515_S396_	2012	D	21.05.2012	S396	14.6	23	Fam-03_2007_	S396	396	1802	3	2007 W
2702_1515_S397_	2012	D	21.05.2012	S397	13.5	18	Fam-04_2007_	S397	397	1803	4	2007 W
2702_1515_S398_	2012	D	21.05.2012	S398	14.4	23.5	Fam-05_2007_	S398	398	1804	5	2007 W
2702_1515_S399_	2012	D	21.05.2012	S399	12.9	17.5	Fam-05_2007_	S399	399	1805	5	2007 W
2702_1515_S400_	2012	D	21.05.2012	S400	16	34	Fam-03_2007_	S400	400	1806	3	2007 W
2703_1515_S401_	2012	D	21.05.2012	S401	17.5	43	Fam-13_2007_	S401	401	1807	13	2007 F
2703_1515_S402_	2012	D	21.05.2012	S402	15	27	Fam-04_2007_	S402	402	1808	4	2007 W
2703_1515_S403_	2012	D	21.05.2012	S403	15.4	27.5	Fam-30_2007_	S403	403	1809	30	2007 H

2703_1515_S405_	2012	D	21.05.2012	S405	13.5	20	Fam-05_2007_	S405	405	1810	5	2007 W
2703_1515_S406_	2012	D	21.05.2012	S406	15.4	28	Fam-03_2007_	S406	406	1811	3	2007 W
2703_1515_S407_	2012	D	21.05.2012	S407	14.6	24.5	Fam-03_2007_	S407	407	1812	3	2007 W
2703_1515_S408_	2012	D	21.05.2012	S408	15	27.5	Fam-10_2007_	S408	408	1813	10	2007 W
2703_1515_S409_	2012	D	21.05.2012	S409	14.2	23	Fam-03_2007_	S409	409	1814	3	2007 W
2703_1515_S410_	2012	D	21.05.2012	S410	14.5	23	Fam-06_2007_	S410	410	1815	6	2007 W
2703_1515_S411_	2012	D	21.05.2012	S411	15	28.5	Fam-09_2007_	S411	411	1816	9	2007 W
2703_1515_S412_	2012	D	21.05.2012	S412	13.8	21.5	Fam-10_2007_	S412	412	1817	10	2007 W
2703_1515_S413_	2012	D	21.05.2012	S413	15.9	33	Fam-25_2007_	S413	413	1818	25	2007 F
2703_1515_S414_	2012	D	21.05.2012	S414	15.2	28	Fam-04_2007_	S414	414	1819	4	2007 W
2703_1515_S415_	2012	D	21.05.2012	S415	15.3	28.5	Fam-09_2007_	S415	415	1820	9	2007 W
2703_1515_S416_	2012	D	21.05.2012	S416	15.4	35.5	Fam-34_2009_	S416	416	1821	34	2009 H
2703_1515_S417_	2012	D	21.05.2012	S417	15.1	28.5	Fam-09_2007_	S417	417	1822	9	2007 W
2703_1515_S418_	2012	D	21.05.2012	S418	16.4	36	Fam-25_2007_	S418	418	1823	25	2007 F

2703_1515_S419_	2012	D	21.05.2012	S419	14.9	27.5	Fam-04_2007_S419	419	1824	4	2007 W
2703_1515_S420_	2012	D	21.05.2012	S420	15.4	27	Fam-17_2007_S420	420	1825	17	2007 F
2703_1515_S421_	2012	D	21.05.2012	S421	15.5	27	Fam-03_2007_S421	421	1826	3	2007 W
2703_1515_S422_	2012	D	21.05.2012	S422	15.9	32	Fam-10_2007_S422	422	1827	10	2007 W
2703_1515_S423_	2012	D	21.05.2012	S423	15	26.5	Fam-10_2007_S423	423	1828	10	2007 W
2703_1515_S424_	2012	D	21.05.2012	S424	15.6	30	Fam-03_2007_S424	424	1829	3	2007 W
2703_1515_S426_	2012	D	21.05.2012	S426	14.2	23	Fam-09_2007_S426	426	1830	9	2007 W
2703_1515_S427_	2012	D	21.05.2012	S427	14.8	27.5	Fam-09_2007_S427	427	1831	9	2007 W
2703_1515_S428_	2012	D	21.05.2012	S428	14.2	21.5	Fam-04_2007_S428	428	1832	4	2007 W
2703_1515_S429_	2012	D	21.05.2012	S429	13.6	20.5	Fam-05_2007_S429	429	1833	5	2007 W
2703_1515_S430_	2012	D	21.05.2012	S430	15.2	29	Fam-09_2007_S430	430	1834	9	2007 W
2703_1515_S432_	2012	D	21.05.2012	S432	13.9	21	Fam-10_2007_S432	432	1835	10	2007 W
2703_1515_S434_	2012	D	21.05.2012	S434	14.9	24.5	Fam-03_2007_S434	434	1836	3	2007 W
2703_1515_S435_	2012	D	21.05.2012	S435	16.4	38	Fam-04_2007_S435	435	1837	4	2007 W

2703_1515_S436_	2012	D	21.05.2012	S436	15.3	29	Fam-10_2007_S436	436	1838	10	2007	W
2703_1515_S437_	2012	D	21.05.2012	S437	15.3	31.5	Fam-04_2007_S437	437	1839	4	2007	W
2703_1515_S438_	2012	D	21.05.2012	S438	15.3	26	Fam-03_2007_S438	438	1840	3	2007	W
2703_1515_S439_	2012	D	21.05.2012	S439	14.1	21	Fam-03_2007_S439	439	1841	3	2007	W
2703_1515_S440_	2012	D	21.05.2012	S440	16	31.5	Fam-51_2009_S440	440	1842	51	2009	W
2703_1515_S441_	2012	D	21.05.2012	S441	16.2	35.5	Fam-10_2007_S441	441	1843	10	2007	W
2703_1515_S442_	2012	D	21.05.2012	S442	15.2	28.5	Fam-03_2007_S442	442	1844	3	2007	W
2703_1515_S443_	2012	D	21.05.2012	S443	14.9	25.5	Fam-03_2007_S443	443	1845	3	2007	W
2703_1515_S444_	2012	D	21.05.2012	S444	13.8	22.5	Fam-10_2007_S444	444	1846	10	2007	W
2703_1515_S445_	2012	D	21.05.2012	S445	15.1	27.5	Fam-04_2007_S445	445	1847	4	2007	W
2703_1515_S446_	2012	D	21.05.2012	S446	14.5	25	Fam-25_2007_S446	446	1848	25	2007	F
2703_1515_S447_	2012	D	21.05.2012	S447	14.4	24	Fam-09_2007_S447	447	1849	9	2007	W
2703_1515_S448_	2012	D	21.05.2012	S448	14.6	25.5	Fam-10_2007_S448	448	1850	10	2007	W
2703_1515_S449_	2012	D	21.05.2012	S449	15.1	28	Fam-04_2007_S449	449	1851	4	2007	W

2703_1515_S450_	2012	D	21.05.2012	S450	15	27.5	Fam-05_2007_S450	450	1852	5	2007	W
2703_1515_S451_	2012	D	21.05.2012	S451	15.9	30	Fam-17_2007_S451	451	1853	17	2007	F
2703_1515_S453_	2012	D	21.05.2012	S453	15.4	30	Fam-10_2007_S453	453	1855	10	2007	W
2703_1515_S454_	2012	D	21.05.2012	S454	14.2	24	Fam-04_2007_S454	454	1856	4	2007	W
2703_1515_S455_	2012	D	21.05.2012	S455	14.7	25.5	Fam-03_2007_S455	455	1857	3	2007	W
2703_1515_S456_	2012	D	21.05.2012	S456	15.7	33.5	Fam-04_2007_S456	456	1858	4	2007	W
2703_1515_S457_	2012	D	21.05.2012	S457	13.9	23.5	Fam-10_2007_S457	457	1859	10	2007	W
2703_1515_S458_	2012	D	21.05.2012	S458	15.9	31.5	Fam-25_2007_S458	458	1860	25	2007	F
2703_1515_S459_	2012	D	21.05.2012	S459	14.2	23.5	Fam-09_2007_S459	459	1861	9	2007	W
2703_1515_S460_	2012	D	21.05.2012	S460	14.2	24	Fam-10_2007_S460	460	1862	10	2007	W
2703_1515_S461_	2012	D	21.05.2012	S461	14	20	Fam-03_2007_S461	461	1863	3	2007	W
2703_1515_S462_	2012	D	21.05.2012	S462	15.5	31.5	Fam-03_2007_S462	462	1864	3	2007	W
2703_1515_S463_	2012	D	21.05.2012	S463	14.9	26	Fam-04_2007_S463	463	1865	4	2007	W
2703_1515_S465_	2012	D	21.05.2012	S465	13.8	20.5	Fam-09_2007_S465	465	1866	9	2007	W

2703_1515_S466_	2012	D	21.05.2012	S466	15.4	28.5	Fam-19_2007_S466	466	1867	19	2007	F
2703_1515_S467_	2012	D	21.05.2012	S467	14.7	24	Fam-09_2007_S467	467	1868	9	2007	W
2703_1515_S468_	2012	D	21.05.2012	S468	15.1	27.5	Fam-10_2007_S468	468	1869	10	2007	W
2703_1515_S469_	2012	D	22.05.2012	S469	15.1	26	Fam-03_2007_S469	469	1870	3	2007	W
2703_1515_S470_	2012	D	22.05.2012	S470	13.2	17.5	Fam-03_2007_S470	470	1871	3	2007	W
2703_1515_S471_	2012	D	22.05.2012	S471	13.9	20.5	Fam-05_2007_S471	471	1872	5	2007	W
2703_1515_S472_	2012	D	22.05.2012	S472	14.8	27	Fam-10_2007_S472	472	1873	10	2007	W
2703_1515_S473_	2012	D	22.05.2012	S473	15.2	29	Fam-04_2007_S473	473	1874	4	2007	W
2703_1515_S474_	2012	D	22.05.2012	S474	13.6	21.5	Fam-05_2007_S474	474	1875	5	2007	W
2703_1515_S475_	2012	D	22.05.2012	S475	14.4	23	Fam-04_2007_S475	475	1876	4	2007	W
2703_1515_S476_	2012	D	22.05.2012	S476	14.8	26	Fam-04_2007_S476	476	1877	4	2007	W
2703_1515_S478_	2012	D	22.05.2012	S478	14.5	23	Fam-03_2007_S478	478	1878	3	2007	W
2703_1515_S479_	2012	D	22.05.2012	S479	13.9	25	Fam-05_2007_S479	479	1879	5	2007	W
2703_1515_S480_	2012	D	22.05.2012	S480	15.1	27.5	Fam-04_2007_S480	480	1880	4	2007	W

2704_1515_S481_	2012	D	22.05.2012	S481	15.5	31.5	Fam-30_2007_S481	481	1881	30	2007	H
2704_1515_S483_	2012	D	22.05.2012	S483	14.5	25.5	Fam-05_2007_S483	483	1882	5	2007	W
2704_1515_S484_	2012	D	22.05.2012	S484	14.5	24	Fam-03_2007_S484	484	1883	3	2007	W
2704_1515_S485_	2012	D	22.05.2012	S485	14.6	25.5	Fam-10_2007_S485	485	1884	10	2007	W
2704_1515_S487_	2012	D	22.05.2012	S487	13.6	19.5	Fam-06_2007_S487	487	1885	6	2007	W
2704_1515_S488_	2012	D	22.05.2012	S488	14.5	24.5	Fam-10_2007_S488	488	1886	10	2007	W
2704_1515_S489_	2012	D	22.05.2012	S489	14.9	26.5	Fam-05_2007_S489	489	1887	5	2007	W
2704_1515_S490_	2012	D	22.05.2012	S490	15.1	27	Fam-03_2007_S490	490	1888	3	2007	W
2704_1515_S491_	2012	D	22.05.2012	S491	14.4	26	Fam-05_2007_S491	491	1889	5	2007	W
2704_1515_S492_	2012	D	22.05.2012	S492	14	22	Fam-05_2007_S492	492	1890	5	2007	W
2704_1515_S493_	2012	D	23.05.2012	S493	15.3	29.5	Fam-09_2007_S493	493	1891	9	2007	W
2704_1515_S494_	2012	D	23.05.2012	S494	16	33	Fam-04_2007_S494	494	1892	4	2007	W
2704_1515_S495_	2012	D	23.05.2012	S495	13.7	20	Fam-03_2007_S495	495	1893	3	2007	W
2704_1515_S496_	2012	D	23.05.2012	S496	15.2	28	Fam-03_2007_S496	496	1894	3	2007	W

2704_1515_S497_	2012	D	23.05.2012	S497	14	22	Fam-04_2007_S497	497	1895	4	2007	W
2704_1515_S498_	2012	D	23.05.2012	S498	14.8	26	Fam-05_2007_S498	498	1896	5	2007	W
2704_1515_S499_	2012	D	23.05.2012	S499	15.7	28	Fam-30_2007_S499	499	1897	30	2007	H
2704_1515_S500_	2012	D	23.05.2012	S500	14.5	25.5	Fam-05_2007_S500	500	1898	5	2007	W
2704_1515_S501_	2012	D	23.05.2012	S501	13.7	20	Fam-05_2007_S501	501	1899	5	2007	W
2704_1515_S503_	2012	D	23.05.2012	S503	15.1	28	Fam-04_2007_S503	503	1901	4	2007	W
2704_1515_S504_	2012	D	23.05.2012	S504	16.5	38	Fam-03_2007_S504	504	1902	3	2007	W
2704_1515_S505_	2012	D	23.05.2012	S505	14.5	24.5	Fam-10_2007_S505	505	1903	10	2007	W
2704_1515_S506_	2012	D	23.05.2012	S506	13.8	21.5	Fam-05_2007_S506	506	1904	5	2007	W
2704_1515_S507_	2012	D	23.05.2012	S507	15.3	30.5	Fam-10_2007_S507	507	1905	10	2007	W
2704_1515_S508_	2012	D	23.05.2012	S508	16.1	31	Fam-10_2007_S508	508	1906	10	2007	W
2704_1515_S509_	2012	D	23.05.2012	S509	14.4	25.5	Fam-04_2007_S509	509	1907	4	2007	W
2704_1515_S510_	2012	D	23.05.2012	S510	14.7	28.5	Fam-04_2007_S510	510	1908	4	2007	W
2704_1515_S511_	2012	D	23.05.2012	S511	14.4	24	Fam-05_2007_S511	511	1909	5	2007	W

2704_1515_S512_	2012	D	23.05.2012	S512	14.6	26	Fam-10_2007_	S512	512	1910	10	2007	W
2704_1515_S513_	2012	D	23.05.2012	S513	14.4	26	Fam-09_2007_	S513	513	1911	9	2007	W
2704_1515_S514_	2012	D	23.05.2012	S514	14.6	26	Fam-04_2007_	S514	514	1912	4	2007	W
2704_1515_S515_	2012	D	23.05.2012	S515	14.4	24.5	Fam-04_2007_	S515	515	1913	4	2007	W
2704_1515_S516_	2012	D	23.05.2012	S516	13	17	Fam-06_2007_	S516	516	1914	6	2007	W
2704_1515_S517_	2012	D	23.05.2012	S517	14	20	Fam-06_2007_	S517	517	1915	6	2007	W
2704_1515_S518_	2012	D	24.05.2012	S518	15	29	Fam-10_2007_	S518	518	1916	10	2007	W
2704_1515_S519_	2012	D	24.05.2012	S519	14.6	23.5	Fam-06_2007_	S519	519	1917	6	2007	W
2704_1515_S520_	2012	D	24.05.2012	S520	14.6	27	Fam-04_2007_	S520	520	1918	4	2007	W
2704_1515_S521_	2012	D	24.05.2012	S521	15.6	30.5	Fam-10_2007_	S521	521	1919	10	2007	W
2704_1515_S522_	2012	D	24.05.2012	S522	15.4	28.5	Fam-10_2007_	S522	522	1920	10	2007	W
2704_1515_S523_	2012	D	24.05.2012	S523	14.2	23.5	Fam-05_2007_	S523	523	1921	5	2007	W
2704_1515_S524_	2012	D	24.05.2012	S524	15.1	28.5	Fam-05_2007_	S524	524	1922	5	2007	W
2704_1515_S525_	2012	D	24.05.2012	S525	13.5	20.5	Fam-05_2007_	S525	525	1923	5	2007	W

2704_1515_S526_	2012	D	24.05.2012	S526	15.9	32	Fam-10_2007_	S526	526	1924	10	2007	W
2704_1515_S527_	2012	D	24.05.2012	S527	13.9	22	Fam-10_2007_	S527	527	1925	10	2007	W
2704_1515_S528_	2012	D	24.05.2012	S528	15.5	27.5	Fam-03_2007_	S528	528	1926	3	2007	W
2704_1515_S529_	2012	D	24.05.2012	S529	19.2	61.5	Fam-07_2007_	S529	529	1927	7	2007	W
2704_1515_S530_	2012	D	24.05.2012	S530	16.1	35	Fam-05_2007_	S530	530	1928	5	2007	W
2704_1515_S531_	2012	D	24.05.2012	S531	14.3	24.5	Fam-10_2007_	S531	531	1929	10	2007	W
2704_1515_S532_	2012	D	24.05.2012	S532	15	28.5	Fam-30_2007_	S532	532	1930	30	2007	H
2704_1515_S533_	2012	D	24.05.2012	S533	13.6	21.5	Fam-05_2007_	S533	533	1931	5	2007	W
2704_1515_S534_	2012	D	24.05.2012	S534	14.9	26.5	Fam-05_2007_	S534	534	1932	5	2007	W
2704_1515_S535_	2012	D	24.05.2012	S535	15.5	31.5	Fam-04_2007_	S535	535	1933	4	2007	W
2704_1515_S536_	2012	D	24.05.2012	S536	14	25.5	Fam-04_2007_	S536	536	1934	4	2007	W
2704_1515_S537_	2012	D	24.05.2012	S537	14.2	24	Fam-10_2007_	S537	537	1935	10	2007	W
2704_1515_S538_	2012	D	24.05.2012	S538	13.9	23	Fam-04_2007_	S538	538	1936	4	2007	W
2704_1515_S539_	2012	D	24.05.2012	S539	14.5	23.5	Fam-03_2007_	S539	539	1937	3	2007	W

2704_1515_S540_	2012	D	24.05.2012	S540	15.5	31	Fam-04_2007_S540	540	1938	4	2007	W
2704_1515_S541_	2012	D	24.05.2012	S541	13.2	20	Fam-04_2007_S541	541	1939	4	2007	W
2704_1515_S542_	2012	D	24.05.2012	S542	15.2	28.5	Fam-04_2007_S542	542	1940	4	2007	W
2704_1515_S543_	2012	D	24.05.2012	S543	14.6	25	Fam-10_2007_S543	543	1941	10	2007	W
2704_1515_S544_	2012	D	24.05.2012	S544	16	33.5	Fam-09_2007_S544	544	1942	9	2007	W
2704_1515_S545_	2012	D	24.05.2012	S545	15.2	31	Fam-05_2007_S545	545	1943	5	2007	W
2704_1515_S546_	2012	D	24.05.2012	S546	14.8	26	Fam-09_2007_S546	546	1944	9	2007	W
2704_1515_S547_	2012	D	24.05.2012	S547	15.7	32.5	Fam-04_2007_S547	547	1945	4	2007	W
2704_1515_S548_	2012	D	24.05.2012	S548	14	22.5	Fam-05_2007_S548	548	1946	5	2007	W
2704_1515_S549_	2012	D	24.05.2012	S549	14	24	Fam-05_2007_S549	549	1947	5	2007	W
2704_1515_S550_	2012	D	24.05.2012	S550	14.3	24	Fam-05_2007_S550	550	1948	5	2007	W
2704_1515_S551_	2012	D	24.05.2012	S551	14.6	26	Fam-05_2007_S551	551	1949	5	2007	W
2704_1515_S552_	2012	D	24.05.2012	S552	14.5	25.5	Fam-05_2007_S552	552	1950	5	2007	W
2704_1515_S553_	2012	D	24.05.2012	S553	14.5	25	Fam-04_2007_S553	553	1951	4	2007	W

2704_1515_S554_	2012	D	24.05.2012	S554	16.1	33.5	Fam-04_2007_	S554	554	1952	4	2007	W
2704_1515_S555_	2012	D	24.05.2012	S555	15.2	27	Fam-03_2007_	S555	555	1953	3	2007	W
2704_1515_S556_	2012	D	24.05.2012	S556	14.2	22	Fam-03_2007_	S556	556	1954	3	2007	W
2704_1515_S557_	2012	D	24.05.2012	S557	14.7	26	Fam-05_2007_	S557	557	1955	5	2007	W
2704_1515_S558_	2012	D	25.05.2012	S558	16.1	33.5	Fam-03_2007_	S558	558	1956	3	2007	W
2704_1515_S559_	2012	D	25.05.2012	S559	14.7	26	Fam-04_2007_	S559	559	1957	4	2007	W
2704_1515_S560_	2012	D	25.05.2012	S560	14.8	25.5	Fam-06_2007_	S560	560	1958	6	2007	W
2705_1515_S561_	2012	D	25.05.2012	S561	15.4	30	Fam-05_2007_	S561	561	1959	5	2007	W
2705_1515_S562_	2012	D	25.05.2012	S562	14.3	23.5	Fam-05_2007_	S562	562	1960	5	2007	W
2705_1515_S563_	2012	D	25.05.2012	S563	13.7	21	Fam-05_2007_	S563	563	1961	5	2007	W
2705_1515_S564_	2012	D	25.05.2012	S564	13.6	21	Fam-06_2007_	S564	564	1962	6	2007	W
2705_1516_S565_	2012	D	25.05.2012	S565	14.8	27	Fam-04_2007_	S565	565	1963	4	2007	W
2705_1516_S566_	2012	D	25.05.2012	S566	14.3	26	Fam-04_2007_	S566	566	1964	4	2007	W
2705_1516_S567_	2012	D	25.05.2012	S567	15.9	33.5	Fam-05_2007_	S567	567	1965	5	2007	W

2705_1516_S568_	2012	D	25.05.2012	S568	13.9	22.5	Fam-05_2007_S568	568	1966	5	2007	W
2705_1516_S569_	2012	D	25.05.2012	S569	14.7	25.5	Fam-04_2007_S569	569	1967	4	2007	W
2705_1516_S570_	2012	D	25.05.2012	S570	14.6	24.5	Fam-04_2007_S570	570	1968	4	2007	W
2705_1516_S571_	2012	D	25.05.2012	S571	13.1	18.5	Fam-04_2007_S571	571	1969	4	2007	W
2705_1516_S572_	2012	D	25.05.2012	S572	15.4	30	Fam-10_2007_S572	572	1970	10	2007	W
2705_1516_S573_	2012	D	25.05.2012	S573	13.8	22	Fam-04_2007_S573	573	1971	4	2007	W
2705_1516_S574_	2012	D	25.05.2012	S574	15.7	32	Fam-09_2007_S574	574	1972	9	2007	W
2705_1516_S575_	2012	D	25.05.2012	S575	15.2	28.5	Fam-05_2007_S575	575	1973	5	2007	W
2705_1516_S576_	2012	D	25.05.2012	S576	16.4	32.5	Fam-03_2007_S576	576	1974	3	2007	W
2705_1516_S577_	2012	D	25.05.2012	S577	15.6	32.5	Fam-03_2007_S577	577	1975	3	2007	W
2705_1516_S578_	2012	D	25.05.2012	S578	15.1	28	Fam-05_2007_S578	578	1976	5	2007	W
2705_1516_S579_	2012	D	25.05.2012	S579	15	28	Fam-04_2007_S579	579	1977	4	2007	W
2705_1516_S580_	2012	D	25.05.2012	S580	13.9	21.5	Fam-06_2007_S580	580	1978	6	2007	W
2705_1516_S581_	2012	D	25.05.2012	S581	16	34.5	Fam-05_2007_S581	581	1979	5	2007	W

2705_1516_S582_	2012	D	25.05.2012	S582	16.1	35.5	Fam-09_2007_	S582	582	1980	9	2007	W
2705_1516_S583_	2012	D	25.05.2012	S583	15.7	32	Fam-10_2007_	S583	583	1981	10	2007	W
2705_1516_S584_	2012	D	25.05.2012	S584	15.2	26	Fam-03_2007_	S584	584	1982	3	2007	W
2705_1516_S585_	2012	D	25.05.2012	S585	14.7	26	Fam-04_2007_	S585	585	1983	4	2007	W
2705_1516_S586_	2012	D	25.05.2012	S586	14.8	25	Fam-10_2007_	S586	586	1984	10	2007	W
2705_1516_S587_	2012	D	25.05.2012	S587	14.7	26.5	Fam-05_2007_	S587	587	1985	5	2007	W
2705_1516_S588_	2012	D	25.05.2012	S588	14	22	Fam-07_2007_	S588	588	1986	7	2007	W
2705_1516_S589_	2012	D	25.05.2012	S589	14	22	Fam-05_2007_	S589	589	1987	5	2007	W
2705_1516_S590_	2012	D	25.05.2012	S590	13.3	19.5	Fam-05_2007_	S590	590	1988	5	2007	W
2705_1516_S591_	2012	D	25.05.2012	S591	15.3	30	Fam-05_2007_	S591	591	1989	5	2007	W
2705_1516_S592_	2012	D	29.05.2012	S592	14.6	24.5	Fam-04_2007_	S592	592	1990	4	2007	W
2705_1516_S593_	2012	D	29.05.2012	S593	16.2	31.5	Fam-03_2007_	S593	593	1991	3	2007	W
2705_1516_S594_	2012	D	29.05.2012	S594	15.5	29.5	Fam-05_2007_	S594	594	1992	5	2007	W
2705_1516_S595_	2012	D	29.05.2012	S595	16.1	31.5	Fam-07_2007_	S595	595	1993	7	2007	W

2705_1516_S596_	2012	D	29.05.2012	S596	15.1	27.5	Fam-05_2007_S596	596	1994	5	2007	W
2705_1516_S597_	2012	D	29.05.2012	S597	13.7	20	Fam-06_2007_S597	597	1995	6	2007	W
2705_1516_S598_	2012	D	29.05.2012	S598	17.3	42	Fam-09_2007_S598	598	1996	9	2007	W
2705_1516_S599_	2012	D	29.05.2012	S599	14	22.5	Fam-05_2007_S599	599	1997	5	2007	W
2705_1516_S600_	2012	D	29.05.2012	S600	15.5	28	Fam-03_2007_S600	600	1998	3	2007	W
2705_1516_S601_	2012	D	29.05.2012	S601	14.4	22	Fam-03_2007_S601	601	1999	3	2007	W
2705_1516_S602_	2012	D	29.05.2012	S602	14.7	25	Fam-04_2007_S602	602	2000	4	2007	W
2705_1516_S603_	2012	D	29.05.2012	S603	14.4	24.5	Fam-05_2007_S603	603	2001	5	2007	W
2705_1516_S604_	2012	D	29.05.2012	S604	15.2	26.5	Fam-07_2007_S604	604	2002	7	2007	W
2705_1516_S605_	2012	D	29.05.2012	S605	14.6	25.5	Fam-05_2007_S605	605	2003	5	2007	W
2705_1516_S606_	2012	D	29.05.2012	S606	14.4	26	Fam-05_2007_S606	606	2004	5	2007	W
2705_1516_S607_	2012	D	29.05.2012	S607	13.6	20.5	Fam-30_2007_S607	607	2005	30	2007	H
2705_1516_S608_	2012	D	29.05.2012	S608	13.3	19.5	Fam-06_2007_S608	608	2006	6	2007	W
2705_1516_S609_	2012	D	29.05.2012	S609	14.3	24.5	Fam-05_2007_S609	609	2007	5	2007	W

2705_1516_S610_	2012	D	29.05.2012	S610	15.6	30	Fam-03_2007_S610	610	2008	3	2007	W
2705_1516_S611_	2012	D	29.05.2012	S611	12.3	14.5	Fam-06_2007_S611	611	2009	6	2007	W
2705_1516_S612_	2012	D	29.05.2012	S612	12.7	17	Fam-05_2007_S612	612	2010	5	2007	W
2705_1516_S613_	2012	D	29.05.2012	S613	14.6	27.5	Fam-04_2007_S613	613	2011	4	2007	W
2705_1516_S614_	2012	D	01.06.2012	S614	15.8	31	Fam-04_2007_S614	614	2012	4	2007	W
2705_1516_S615_	2012	D	01.06.2012	S615	14.4	24.5	Fam-05_2007_S615	615	2013	5	2007	W
2705_1516_S616_	2012	D	01.06.2012	S616	14.8	25.5	Fam-05_2007_S616	616	2014	5	2007	W
2705_1516_S617_	2012	D	01.06.2012	S617	14.5	24	Fam-09_2007_S617	617	2015	9	2007	W
2705_1516_S618_	2012	D	01.06.2012	S618	14	21	Fam-05_2007_S618	618	2016	5	2007	W
2705_1516_S619_	2012	D	13.06.2012	S619	16.5	43	Fam-10_2007_S619	619	2017	10	2007	W
2705_1516_S620_	2012	D	13.06.2012	S620	15.3	27	Fam-05_2007_S620	620	2018	5	2007	W
2705_1516_S621_	2012	D	13.06.2012	S621	15.2	27.5	Fam-04_2007_S621	621	2019	4	2007	W
2705_1516_S622_	2012	D	13.06.2012	S622	15.1	29	Fam-04_2007_S622	622	2020	4	2007	W
2705_1516_S623_	2012	D	13.06.2012	S623	16.4	34.5	Fam-03_2007_S623	623	2021	3	2007	W

2705_1516_S624_	2012	D	15.06.2012	S624	15.8	30.5	Fam-03_2007_S624	624	2022	3	2007	W
2705_1516_S625_	2012	D	15.06.2012	S625	16.2	31	Fam-04_2007_S625	625	2023	4	2007	W
2705_1516_S626_	2012	D	18.06.2012	S626	15	29	Fam-05_2007_S626	626	2024	5	2007	W
2705_1516_S627_	2012	D	18.06.2012	S627	15.5	28.5	Fam-05_2007_S627	627	2025	5	2007	W
2705_1516_S628_	2012	D	18.06.2012	S628	15	28.5	Fam-05_2007_S628	628	2026	5	2007	W
2705_1516_S629_	2012	D	18.06.2012	S629	14.7	26.5	Fam-05_2007_S629	629	2027	5	2007	W
2705_1516_S630_	2012	D	18.06.2012	S630	15.1	27.5	Fam-05_2007_S630	630	2028	5	2007	W
2705_1516_S631_	2012	D	18.06.2012	S631	15	26	Fam-06_2007_S631	631	2029	6	2007	W
2705_1516_S632_	2012	D	18.06.2012	S632	16.5	35.5	Fam-07_2007_S632	632	2030	7	2007	W
2705_1516_S633_	2012	D	18.06.2012	S633	14.6	24	Fam-06_2007_S633	633	2031	6	2007	W
2705_1516_S634_	2012	D	18.06.2012	S634	16.4	35	Fam-05_2007_S634	634	2032	5	2007	W
2705_1516_S635_	2012	D	18.06.2012	S635	14.8	26	Fam-05_2007_S635	635	2033	5	2007	W
2705_1516_S636_	2012	D	18.06.2012	S636	15.1	27.5	Fam-05_2007_S636	636	2034	5	2007	W
2705_1516_S637_	2012	D	18.06.2012	S637	15.3	27	Fam-05_2007_S637	637	2035	5	2007	W

2705_1516_S638_	2012	D	18.06.2012	S638	15.5	30	Fam-05_2007_ S638	638	2036	5	2007	W
2705_1516_S639_	2012	D	18.06.2012	S639	15.3	28	Fam-05_2007_ S639	639	2037	5	2007	W
2705_1516_S640_	2012	D	18.06.2012	S640	15.3	29.5	Fam-05_2007_ S640	640	2038	5	2007	W
2706_1516_S641_	2012	D	18.06.2012	S641	15	27.5	Fam-05_2007_ S641	641	2039	5	2007	W
2706_1516_S642_	2012	D	18.06.2012	S642	14.2	23	Fam-05_2007_ S642	642	2040	5	2007	W
2706_1516_S643_	2012	D	18.06.2012	S643	14.5	25.5	Fam-05_2007_ S643	643	2041	5	2007	W
2706_1516_S644_	2012	D	18.06.2012	S644	16.6	36.5	Fam-05_2007_ S644	644	2042	5	2007	W
2706_1516_S645_	2012	D	18.06.2012	S645	14.1	21.5	Fam-52_2009_ S645	645	2043	52	2009	W
2706_1516_S646_	2012	D	18.06.2012	S646	15.6	30.5	Fam-05_2007_ S646	646	2044	5	2007	W
2706_1516_S647_	2012	D	18.06.2012	S647	15.8	32.5	Fam-04_2007_ S647	647	2045	4	2007	W
2706_1516_S648_	2012	D	18.06.2012	S648	15.5	29	Fam-05_2007_ S648	648	2046	5	2007	W
2706_1516_S649_	2012	D	18.06.2012	S649	15.6	30	Fam-04_2007_ S649	649	2047	4	2007	W
2706_1516_S650_	2012	D	19.06.2012	S650	14.9	27	Fam-05_2007_ S650	650	2048	5	2007	W
2706_1516_S651_	2012	D	20.06.2012	S651	15.3	28	Fam-05_2007_ S651	651	2049	5	2007	W

2706_1516_S652_	2012	D	20.06.2012	S652	14.5	24	Fam-05_2007_S652	652	2050	5	2007	W
2706_1516_S653_	2012	D	22.06.2012	S653	14.5	23.5	Fam-05_2007_S653	653	2051	5	2007	W
2706_1516_S654_	2012	D	22.06.2012	S654	15.5	30	Fam-05_2007_S654	654	2052	5	2007	W
2707_1517_S1_A(	2013	D	24.04.2013	S1	13.2	18	Fam-35_2009_S1	1	2053	35	2009	F
2707_1517_S3_C(	2013	D	29.04.2013	S3	14.5	24	Fam-37_2009_S3	3	2055	37	2009	F
2707_1517_S4_D(	2013	D	29.04.2013	S4	14	23.5	Fam-47_2009_S4	4	2056	47	2009	F
2707_1517_S6_F(	2013	D	29.04.2013	S6	14.5	24.5	Fam-37_2009_S6	6	2057	37	2009	F
2707_1517_S7_G(	2013	D	29.04.2013	S7	14	22	Fam-37_2009_S7	7	2058	37	2009	F
2707_1517_S8_H(	2013	D	10.05.2013	S8	13.6	19.5	Fam-35_2009_S8	8	2059	35	2009	F
2707_1517_S9_A(	2013	D	10.05.2013	S9	14.5	25.5	Fam-11_2007_S9	9	2060	11	2007	F
2707_1517_S10_E	2013	D	10.05.2013	S10	14.6	24	Fam-35_2009_S10	10	2061	35	2009	F
2707_1517_S11_(	2013	D	10.05.2013	S11	14.7	25	Fam-39_2009_S11	11	2062	39	2009	F
2707_1517_S13_E	2013	D	10.05.2013	S13	13.5	19.5	Fam-31_2009_S13	13	2064	31	2009	F
2707_1517_S14_F	2013	D	10.05.2013	S14	13.4	19	Fam-39_2009_S14	14	2065	39	2009	F

2707_1517_S15_(	2013	D	10.05.2013	S15	13	18	Fam-48_2009_	S15	15	2066	48	2009	H
2707_1517_S16_H	2013	D	10.05.2013	S16	13.1	19.5	Fam-47_2009_	S16	16	2067	47	2009	F
2707_1517_S18_E	2013	D	10.05.2013	S18	12.5	16	Fam-58_2009_	S18	18	2068	58	2009	W
2707_1517_S19_(	2013	D	10.05.2013	S19	14.4	24.4	Fam-48_2009_	S19	19	2069	48	2009	H
2707_1517_S20_[	2013	D	10.05.2013	S20	13.3	20.5	Fam-31_2009_	S20	20	2070	31	2009	F
2707_1517_S22_F	2013	D	10.05.2013	S22	11.9	14.5	Fam-44_2009_	S22	22	2072	44	2009	H
2707_1517_S25_A	2013	D	10.05.2013	S25	13.2	19	Fam-51_2009_	S25	25	2075	51	2009	W
2707_1517_S26_E	2013	D	10.05.2013	S26	14	23	Fam-31_2009_	S26	26	2076	31	2009	F
2707_1517_S27_(	2013	D	10.05.2013	S27	13.8	21.5	Fam-43_2009_	S27	27	2077	43	2009	F
2707_1517_S28_[	2013	D	10.05.2013	S28	14	22	Fam-57_2009_	S28	28	2078	57	2009	W
2707_1517_S30_F	2013	D	10.05.2013	S30	14.2	25.5	Fam-31_2009_	S30	30	2080	31	2009	F
2707_1517_S31_(	2013	D	10.05.2013	S31	14.6	28	Fam-38_2009_	S31	31	2081	38	2009	H
2707_1517_S32_H	2013	D	10.05.2013	S32	14	22	Fam-37_2009_	S32	32	2082	37	2009	F
2707_1517_S33_A	2013	D	10.05.2013	S33	14.1	23	Fam-11_2007_	S33	33	2083	11	2007	F

2707_1517_S34_E	2013	D	10.05.2013	S34	14.1	24	Fam-38_2009_	S34	34	2084	38	2009 H
2707_1517_S35_C	2013	D	10.05.2013	S35	13.4	18.5	Fam-36_2009_	S35	35	2085	36	2009 H
2707_1517_S36_D	2013	D	10.05.2013	S36	13.4	19.5	Fam-62_1_201	S36	36	2086	62	2010 H
2707_1517_S37_E	2013	D	10.05.2013	S37	14.7	25.5	Fam-33_2009_	S37	37	2087	33	2009 F
2707_1517_S38_F	2013	D	10.05.2013	S38	13.9	21.5	Fam-76_2010_	S38	38	2088	76	2010 H
2707_1517_S39_G	2013	D	10.05.2013	S39	14	22	Fam-47_2009_	S39	39	2089	47	2009 F
2707_1517_S40_H	2013	D	10.05.2013	S40	15.1	28	Fam-33_2009_	S40	40	2090	33	2009 F
2707_1517_S41_A	2013	D	10.05.2013	S41	13.3	19	Fam-37_2009_	S41	41	2091	37	2009 F
2707_1517_S43_C	2013	D	10.05.2013	S43	15.2	28.5	Fam-28_2007_	S43	43	2092	28	2007 H
2707_1517_S44_D	2013	D	10.05.2013	S44	14.1	22	Fam-48_2009_	S44	44	2093	48	2009 H
2707_1517_S45_E	2013	D	10.05.2013	S45	13.2	20	Fam-62_1_201	S45	45	2094	62	2010 H
2707_1517_S46_F	2013	D	10.05.2013	S46	16.2	37.5	Fam-44_2009_	S46	46	2095	44	2009 H
2707_1517_S47_G	2013	D	10.05.2013	S47	14.2	23.5	Fam-48_2009_	S47	47	2096	48	2009 H
2707_1517_S48_H	2013	D	10.05.2013	S48	13.6	22	Fam-38_2009_	S48	48	2097	38	2009 H

2707_1517_S49_A	2013	D	10.05.2013	S49	15.2	29	Fam-39_2009_	S49	49	2098	39	2009	F
2707_1517_S50_E	2013	D	10.05.2013	S50	13.2	19.5	Fam-37_2009_	S50	50	2099	37	2009	F
2707_1517_S51_D	2013	D	10.05.2013	S51	12.2	15.5	Fam-59_2009_	S51	51	2100	59	2009	W
2707_1517_S52_E	2013	D	10.05.2013	S52	12.7	16.5	Fam-44_2009_	S52	52	2101	44	2009	H
2707_1517_S53_F	2013	D	10.05.2013	S53	14.4	23	Fam-37_2009_	S53	53	2102	37	2009	F
2707_1517_S54_G	2013	D	10.05.2013	S54	14.3	23.5	Fam-37_2009_	S54	54	2103	37	2009	F
2707_1517_S55_H	2013	D	10.05.2013	S55	13	17.5	Fam-56_2009_	S55	55	2104	56	2009	W
2707_1517_S56_A	2013	D	10.05.2013	S56	14.9	26.5	Fam-47_2009_	S56	56	2105	47	2009	F
2707_1517_S57_E	2013	D	10.05.2013	S57	13.4	20	Fam-31_2009_	S57	57	2106	31	2009	F
2707_1517_S58_C	2013	D	10.05.2013	S58	14	22	Fam-37_2009_	S58	58	2107	37	2009	F
2707_1517_S59_D	2013	D	10.05.2013	S59	13.9	23	Fam-45_2009_	S59	59	2108	45	2009	F
2707_1517_S60_E	2013	D	10.05.2013	S60	12.2	16	Fam-44_2009_	S60	60	2109	44	2009	H
2707_1517_S61_F	2013	D	10.05.2013	S61	14.9	28.5	Fam-48_2009_	S61	61	2110	48	2009	H
2707_1517_S62_G	2013	D	10.05.2013	S62	14.1	25.5	Fam-45_2009_	S62	62	2111	45	2009	F

2707_1517_S63_H	2013	D	10.05.2013	S63	13	17.5	Fam-58_2009_	S63	63	2112	58	2009 W
2707_1517_S64_A	2013	D	10.05.2013	S64	12.5	15.5	Fam-47_2009_	S64	64	2113	47	2009 F
2707_1517_S65_B	2013	D	10.05.2013	S65	12.8	17.5	Fam-36_2009_	S65	65	2114	36	2009 H
2707_1517_S66_C	2013	D	10.05.2013	S66	12.8	17	Fam-58_2009_	S66	66	2115	58	2009 W
2707_1517_S67_D	2013	D	10.05.2013	S67	12.8	18.5	Fam-45_2009_	S67	67	2116	45	2009 F
2707_1517_S68_E	2013	D	10.05.2013	S68	13.4	20	Fam-54_2009_	S68	68	2117	54	2009 W
2707_1517_S69_F	2013	D	10.05.2013	S69	13.9	22	Fam-37_2009_	S69	69	2118	37	2009 F
2707_1517_S70_G	2013	D	10.05.2013	S70	13.3	21	Fam-42_2009_	S70	70	2119	42	2009 H
2707_1517_S71_H	2013	D	10.05.2013	S71	14.3	23.5	Fam-36_2009_	S71	71	2120	36	2009 H
2707_1517_S72_A	2013	D	10.05.2013	S72	14	23.5	Fam-27_2007_	S72	72	2121	27	2007 F
2707_1517_S73_B	2013	D	10.05.2013	S73	13.4	20	Fam-31_2009_	S73	73	2122	31	2009 F
2707_1517_S74_C	2013	D	10.05.2013	S74	14.5	25	Fam-38_2009_	S74	74	2123	38	2009 H
2707_1517_S75_D	2013	D	10.05.2013	S75	13.4	21.5	Fam-45_2009_	S75	75	2124	45	2009 F
2707_1517_S76_E	2013	D	10.05.2013	S76	13.2	18.5	Fam-39_2009_	S76	76	2125	39	2009 F

2707_1517_S77_F	2013	D	10.05.2013	S77	12.9	18.5	Fam-33_2009_	S77	77	2126	33	2009	F
2707_1517_S78_G	2013	D	10.05.2013	S78	13.7	21	Fam-35_2009_	S78	78	2127	35	2009	F
2707_1517_S79_H	2013	D	10.05.2013	S79	13	17.5	Fam-51_2009_	S79	79	2128	51	2009	W
2707_1517_S80_A	2013	D	10.05.2013	S80	13.3	20	Fam-45_2009_	S80	80	2129	45	2009	F
2708_1517_S81_C	2013	D	10.05.2013	S81	14	21.5	Fam-35_2009_	S81	81	2130	35	2009	F
2708_1517_S82_D	2013	D	10.05.2013	S82	14.2	23	Fam-47_2009_	S82	82	2131	47	2009	F
2708_1517_S84_F	2013	D	10.05.2013	S84	13.3	21	Fam-47_2009_	S84	84	2132	47	2009	F
2708_1517_S85_G	2013	D	10.05.2013	S85	11.7	13	Fam-56_2009_	S85	85	2133	56	2009	W
2708_1517_S86_H	2013	D	10.05.2013	S86	13.3	18.5	Fam-57_2009_	S86	86	2134	57	2009	W
2708_1517_S87_A	2013	D	10.05.2013	S87	13.5	21.5	Fam-54_2009_	S87	87	2135	54	2009	W
2708_1517_S88_B	2013	D	10.05.2013	S88	13.3	20	Fam-31_2009_	S88	88	2136	31	2009	F
2708_1517_S89_C	2013	D	10.05.2013	S89	13.7	19.5	Fam-36_2009_	S89	89	2137	36	2009	H
2708_1517_S90_D	2013	D	10.05.2013	S90	12.9	18	Fam-38_2009_	S90	90	2138	38	2009	H
2708_1517_S91_E	2013	D	10.05.2013	S91	13	20	Fam-36_2009_	S91	91	2139	36	2009	H

2708_1517_S92_F	2013	D	10.05.2013	S92	14.5	25	Fam-48_2009_	S92	92	2140	48	2009	H
2708_1517_S93_G	2013	D	10.05.2013	S93	13.7	22.5	Fam-48_2009_	S93	93	2141	48	2009	H
2708_1517_S94_H	2013	D	10.05.2013	S94	12.6	17	Fam-39_2009_	S94	94	2142	39	2009	F
2708_1518_S95_A	2013	D	10.05.2013	S95	12.7	23	Fam-54_2009_	S95	95	2143	54	2009	W
2708_1518_S96_B	2013	D	10.05.2013	S96	14.7	23	Fam-45_2009_	S96	96	2144	45	2009	F
2708_1518_S97_C	2013	D	10.05.2013	S97	14	23	Fam-31_2009_	S97	97	2145	31	2009	F
2708_1518_S98_D	2013	D	10.05.2013	S98	13.5	21.5	Fam-56_2009_	S98	98	2146	56	2009	W
2708_1518_S99_E	2013	D	10.05.2013	S99	15.4	29	Fam-33_2009_	S99	99	2147	33	2009	F
2708_1518_S100_	2013	D	10.05.2013	S100	12.7	17	Fam-37_2009_	S100	100	2148	37	2009	F
2708_1518_S101_	2013	D	10.05.2013	S101	12.7	17	Fam-56_2009_	S101	101	2149	56	2009	W
2708_1518_S102_	2013	D	10.05.2013	S102	14.1	24	Fam-31_2009_	S102	102	2150	31	2009	F
2708_1518_S103_	2013	D	10.05.2013	S103	14.1	22	Fam-38_2009_	S103	103	2151	38	2009	H
2708_1518_S104_	2013	D	10.05.2013	S104	11.8	13.5	Fam-58_2009_	S104	104	2152	58	2009	W
2708_1518_S105_	2013	D	10.05.2013	S105	13	18.5	Fam-47_2009_	S105	105	2153	47	2009	F

2708_1518_S106_	2013	D	10.05.2013	S106	13.9	22	Fam-43_2009_	S106	106	2154	43	2009	F
2708_1518_S107_	2013	D	10.05.2013	S107	13.5	20	Fam-39_2009_	S107	107	2155	39	2009	F
2708_1518_S108_	2013	D	10.05.2013	S108	13.9	23.5	Fam-31_2009_	S108	108	2156	31	2009	F
2708_1518_S109_	2013	D	10.05.2013	S109	14.7	24.5	Fam-36_2009_	S109	109	2157	36	2009	H
2708_1518_S110_	2013	D	10.05.2013	S110	16.3	34	Fam-50_2009_	S110	110	2158	50	2009	H
2708_1518_S111_	2013	D	10.05.2013	S111	13.3	19.5	Fam-31_2009_	S111	111	2159	31	2009	F
2708_1518_S112_	2013	D	10.05.2013	S112	15.4	31.5	Fam-45_2009_	S112	112	2160	45	2009	F
2708_1518_S113_	2013	D	10.05.2013	S113	12.7	17	Fam-39_2009_	S113	113	2161	39	2009	F
2708_1518_S114_	2013	D	10.05.2013	S114	15.1	26	Fam-33_2009_	S114	114	2162	33	2009	F
2708_1518_S115_	2013	D	10.05.2013	S115	13.7	20	Fam-38_2009_	S115	115	2163	38	2009	H
2708_1518_S116_	2013	D	10.05.2013	S116	13.4	20.5	Fam-35_2009_	S116	116	2164	35	2009	F
2708_1518_S117_	2013	D	10.05.2013	S117	14	22.5	Fam-48_2009_	S117	117	2165	48	2009	H
2708_1518_S118_	2013	D	10.05.2013	S118	13.8	21.5	Fam-48_2009_	S118	118	2166	48	2009	H
2708_1518_S119_	2013	D	10.05.2013	S119	13	17	Fam-51_2009_	S119	119	2167	51	2009	W

2708_1518_S120_	2013	D	10.05.2013	S120	13.3	18.5	Fam-38_2009_S120	120	2168	38	2009	H
2708_1518_S121_	2013	D	10.05.2013	S121	13.2	19	Fam-39_2009_S121	121	2169	39	2009	F
2708_1518_S122_	2013	D	10.05.2013	S122	12.4	15	Fam-51_2009_S122	122	2170	51	2009	W
2708_1518_S123_	2013	D	10.05.2013	S123	13.2	18.5	Fam-37_2009_S123	123	2171	37	2009	F
2708_1518_S124_	2013	D	10.05.2013	S124	12.9	17	Fam-58_2009_S124	124	2172	58	2009	W
2708_1518_S125_	2013	D	10.05.2013	S125	13.9	22	Fam-45_2009_S125	125	2173	45	2009	F
2708_1518_S126_	2013	D	10.05.2013	S126	12.8	18	Fam-36_2009_S126	126	2174	36	2009	H
2708_1518_S127_	2013	D	10.05.2013	S127	13	18	Fam-50_2009_S127	127	2175	50	2009	H
2708_1518_S128_	2013	D	10.05.2013	S128	13.9	21.5	Fam-38_2009_S128	128	2176	38	2009	H
2708_1518_S129_	2013	D	10.05.2013	S129	13.6	21.5	Fam-35_2009_S129	129	2177	35	2009	F
2708_1518_S130_	2013	D	10.05.2013	S130	14.4	24	Fam-37_2009_S130	130	2178	37	2009	F
2708_1518_S131_	2013	D	10.05.2013	S131	14.1	22.5	Fam-51_2009_S131	131	2179	51	2009	W
2708_1518_S132_	2013	D	10.05.2013	S132	13.8	21.5	Fam-51_2009_S132	132	2180	51	2009	W
2708_1518_S133_	2013	D	10.05.2013	S133	13.9	22	Fam-48_2009_S133	133	2181	48	2009	H

2708_1518_S134_	2013	D	10.05.2013	S134	13.2	20	Fam-39_2009_	S134	134	2182	39	2009 F
2708_1518_S135_	2013	D	10.05.2013	S135	13.7	21	Fam-51_2009_	S135	135	2183	51	2009 W
2708_1518_S136_	2013	D	10.05.2013	S136	13.8	22	Fam-47_2009_	S136	136	2184	47	2009 F
2708_1518_S137_	2013	D	10.05.2013	S137	12.3	15	Fam-44_2009_	S137	137	2185	44	2009 H
2708_1518_S138_	2013	D	10.05.2013	S138	14.4	25	Fam-48_2009_	S138	138	2186	48	2009 H
2708_1518_S139_	2013	D	10.05.2013	S139	13.2	17.5	Fam-39_2009_	S139	139	2187	39	2009 F
2708_1518_S140_	2013	D	10.05.2013	S140	13.4	20.5	Fam-35_2009_	S140	140	2188	35	2009 F
2708_1518_S141_	2013	D	10.05.2013	S141	13.2	18	Fam-58_2009_	S141	141	2189	58	2009 W
2708_1518_S142_	2013	D	10.05.2013	S142	12.9	16.5	Fam-51_2009_	S142	142	2190	51	2009 W
2708_1518_S143_	2013	D	10.05.2013	S143	14	21	Fam-38_2009_	S143	143	2191	38	2009 H
2708_1518_S144_	2013	D	10.05.2013	S144	12.8	19.5	Fam-44_2009_	S144	144	2192	44	2009 H
2708_1518_S145_	2013	D	10.05.2013	S145	12.6	16.5	Fam-58_2009_	S145	145	2193	58	2009 W
2708_1518_S146_	2013	D	10.05.2013	S146	12.7	18	Fam-54_2009_	S146	146	2194	54	2009 W
2708_1518_S147_	2013	D	10.05.2013	S147	13.4	21.5	Fam-31_2009_	S147	147	2195	31	2009 F

2708_1518_S148_	2013	D	10.05.2013	S148	11.9	15	Fam-36_2009_	S148	148	2196	36	2009	H
2708_1518_S149_	2013	D	10.05.2013	S149	14	23	Fam-31_2009_	S149	149	2197	31	2009	F
2708_1518_S150_	2013	D	10.05.2013	S150	13.9	22.5	Fam-47_2009_	S150	150	2198	47	2009	F
2708_1518_S151_	2013	D	10.05.2013	S151	14.3	24.5	Fam-47_2009_	S151	151	2199	47	2009	F
2708_1518_S152_	2013	D	10.05.2013	S152	13.9	21.5	Fam-89_2010_	S152	152	2200	89	2010	W
2708_1518_S153_	2013	D	10.05.2013	S153	13.2	19.5	Fam-42_2009_	S153	153	2201	42	2009	H
2708_1518_S154_	2013	D	10.05.2013	S154	14.1	23	Fam-50_2009_	S154	154	2202	50	2009	H
2708_1518_S155_	2013	D	10.05.2013	S155	14.4	24	Fam-37_2009_	S155	155	2203	37	2009	F
2708_1518_S156_	2013	D	10.05.2013	S156	11.8	14.5	Fam-44_2009_	S156	156	2204	44	2009	H
2708_1518_S157_	2013	D	10.05.2013	S157	12	15	Fam-44_2009_	S157	157	2205	44	2009	H
2708_1518_S158_	2013	D	10.05.2013	S158	15.9	31.5	Fam-50_2009_	S158	158	2206	50	2009	H
2708_1518_S159_	2013	D	10.05.2013	S159	13.1	18	Fam-51_2009_	S159	159	2207	51	2009	W
2708_1518_S160_	2013	D	10.05.2013	S160	13.4	20	Fam-31_2009_	S160	160	2208	31	2009	F
2709_1518_S161_	2013	D	10.05.2013	S161	13	17	Fam-35_2009_	S161	161	2209	35	2009	F

2709_1518_S162_	2013	D	10.05.2013	S162	13.9	23	Fam-38_2009_	S162	162	2210	38	2009	H
2709_1518_S163_	2013	D	10.05.2013	S163	14.6	25.5	Fam-56_2009_	S163	163	2211	56	2009	W
2709_1518_S164_	2013	D	10.05.2013	S164	13.7	20.5	Fam-51_2009_	S164	164	2212	51	2009	W
2709_1518_S165_	2013	D	10.05.2013	S165	13.6	22	Fam-38_2009_	S165	165	2213	38	2009	H
2709_1518_S166_	2013	D	10.05.2013	S166	13.5	20.5	Fam-85_2010_	S166	166	2214	85	2010	W
2709_1518_S167_	2013	D	10.05.2013	S167	13	18.5	Fam-37_2009_	S167	167	2215	37	2009	F
2709_1518_S168_	2013	D	10.05.2013	S168	12.8	16.5	Fam-35_2009_	S168	168	2216	35	2009	F
2709_1518_S169_	2013	D	10.05.2013	S169	15.5	31.5	Fam-33_2009_	S169	169	2217	33	2009	F
2709_1518_S170_	2013	D	10.05.2013	S170	13.6	21	Fam-47_2009_	S170	170	2218	47	2009	F
2709_1518_S171_	2013	D	10.05.2013	S171	12.3	16	Fam-41_2009_	S171	171	2219	41	2009	F
2709_1518_S172_	2013	D	10.05.2013	S172	13.8	20	Fam-39_2009_	S172	172	2220	39	2009	F
2709_1518_S173_	2013	D	10.05.2013	S173	14.2	24	Fam-43_2009_	S173	173	2221	43	2009	F
2709_1518_S174_	2013	D	10.05.2013	S174	14.5	25.5	Fam-48_2009_	S174	174	2222	48	2009	H
2709_1518_S175_	2013	D	10.05.2013	S175	14	23	Fam-37_2009_	S175	175	2223	37	2009	F

2709_1518_S176_	2013	D	10.05.2013	S176	13.1	17	Fam-48_2009_	S176	176	2224	48	2009	H
2709_1518_S177_	2013	D	10.05.2013	S177	14.2	22.5	Fam-58_2009_	S177	177	2225	58	2009	W
2709_1518_S178_	2013	D	10.05.2013	S178	13.2	18.5	Fam-58_2009_	S178	178	2226	58	2009	W
2709_1518_S179_	2013	D	10.05.2013	S179	14.5	25.5	Fam-31_2009_	S179	179	2227	31	2009	F
2709_1518_S180_	2013	D	10.05.2013	S180	13	18	Fam-36_2009_	S180	180	2228	36	2009	H
2709_1518_S181_	2013	D	10.05.2013	S181	13.7	20.5	Fam-48_2009_	S181	181	2229	48	2009	H
2709_1518_S182_	2013	D	10.05.2013	S182	15.4	31.5	Fam-47_2009_	S182	182	2230	47	2009	F
2709_1518_S183_	2013	D	10.05.2013	S183	12.9	16.5	Fam-48_2009_	S183	183	2231	48	2009	H
2709_1518_S184_	2013	D	10.05.2013	S184	13.8	21	Fam-48_2009_	S184	184	2232	48	2009	H
2709_1518_S185_	2013	D	10.05.2013	S185	13.9	20.5	Fam-31_2009_	S185	185	2233	31	2009	F
2709_1518_S186_	2013	D	10.05.2013	S186	13.2	18.5	Fam-47_2009_	S186	186	2234	47	2009	F
2709_1518_S187_	2013	D	10.05.2013	S187	13.5	20.5	Fam-36_2009_	S187	187	2235	36	2009	H
2709_1518_S188_	2013	D	10.05.2013	S188	13.4	20.5	Fam-38_2009_	S188	188	2236	38	2009	H
2709_1519_S189_	2013	D	10.05.2013	S189	13.5	21.5	Fam-35_2009_	S189	189	2237	35	2009	F

2709_1519_S190_	2013	D	10.05.2013	S190	14.9	29	Fam-39_2009_	S190	190	2238	39	2009	F
2709_1519_S191_	2013	D	10.05.2013	S191	14.7	26.5	Fam-31_2009_	S191	191	2239	31	2009	F
2709_1519_S192_	2013	D	10.05.2013	S192	14.4	24.5	Fam-51_2009_	S192	192	2240	51	2009	W
2709_1519_S193_	2013	D	10.05.2013	S193	13.6	20	Fam-38_2009_	S193	193	2241	38	2009	H
2709_1519_S194_	2013	D	10.05.2013	S194	13.6	21.5	Fam-41_2009_	S194	194	2242	41	2009	F
2709_1519_S195_	2013	D	10.05.2013	S195	12.2	16	Fam-57_2009_	S195	195	2243	57	2009	W
2709_1519_S196_	2013	D	10.05.2013	S196	11.7	12.5	Fam-58_2009_	S196	196	2244	58	2009	W
2709_1519_S197_	2013	D	10.05.2013	S197	13.1	23.5	Fam-62_1_201	S197	197	2245	62	2010	H
2709_1519_S198_	2013	D	10.05.2013	S198	14.4	24	Fam-57_2009_	S198	198	2246	57	2009	W
2709_1519_S199_	2013	D	10.05.2013	S199	14.6	25.5	Fam-47_2009_	S199	199	2247	47	2009	F
2709_1519_S200_	2013	D	10.05.2013	S200	13.4	19	Fam-40_2009_	S200	200	2248	40	2009	H
2709_1519_S201_	2013	D	10.05.2013	S201	13.5	19.5	Fam-50_2009_	S201	201	2249	50	2009	H
2709_1519_S202_	2013	D	10.05.2013	S202	13.7	21.5	Fam-37_2009_	S202	202	2250	37	2009	F
2709_1519_S203_	2013	D	10.05.2013	S203	13.1	18.5	Fam-48_2009_	S203	203	2251	48	2009	H

2709_1519_S204_	2013	D	10.05.2013	S204	14.7	26.5	Fam-45_2009_S204	204	2252	45	2009	F
2709_1519_S205_	2013	D	10.05.2013	S205	14.2	23	Fam-33_2009_S205	205	2253	33	2009	F
2709_1519_S206_	2013	D	10.05.2013	S206	13.8	20.5	Fam-47_2009_S206	206	2254	47	2009	F
2709_1519_S207_	2013	D	10.05.2013	S207	13	19	Fam-45_2009_S207	207	2255	45	2009	F
2709_1519_S208_	2013	D	10.05.2013	S208	14.7	26.5	Fam-35_2009_S208	208	2256	35	2009	F
2709_1519_S209_	2013	D	10.05.2013	S209	14.8	24.5	Fam-39_2009_S209	209	2257	39	2009	F
2709_1519_S210_	2013	D	10.05.2013	S210	14.6	25	Fam-45_2009_S210	210	2258	45	2009	F
2709_1519_S211_	2013	D	10.05.2013	S211	13.4	20.5	Fam-50_2009_S211	211	2259	50	2009	H
2709_1519_S212_	2013	D	10.05.2013	S212	14.6	25	Fam-51_2009_S212	212	2260	51	2009	W
2709_1519_S213_	2013	D	10.05.2013	S213	14.4	25.5	Fam-37_2009_S213	213	2261	37	2009	F
2709_1519_S214_	2013	D	10.05.2013	S214	13.3	19.5	Fam-31_2009_S214	214	2262	31	2009	F
2709_1519_S215_	2013	D	10.05.2013	S215	13.2	19.5	Fam-39_2009_S215	215	2263	39	2009	F
2709_1519_S216_	2013	D	10.05.2013	S216	14	23	Fam-43_2009_S216	216	2264	43	2009	F
2709_1519_S217_	2013	D	10.05.2013	S217	13.8	21	Fam-39_2009_S217	217	2265	39	2009	F

2709_1519_S218_	2013	D	10.05.2013	S218	15.3	28.5	Fam-28_2007_	S218	218	2266	28	2007	H
2709_1519_S219_	2013	D	10.05.2013	S219	15.5	28.5	Fam-52_2009_	S219	219	2267	52	2009	W
2709_1519_S220_	2013	D	10.05.2013	S220	12.1	15	Fam-47_2009_	S220	220	2268	47	2009	F
2709_1519_S221_	2013	D	10.05.2013	S221	12.2	15.5	Fam-44_2009_	S221	221	2269	44	2009	H
2709_1519_S222_	2013	D	10.05.2013	S222	12.2	15	Fam-56_2009_	S222	222	2270	56	2009	W
2709_1519_S223_	2013	D	10.05.2013	S223	15.1	28.5	Fam-38_2009_	S223	223	2271	38	2009	H
2709_1519_S224_	2013	D	10.05.2013	S224	13.9	23	Fam-35_2009_	S224	224	2272	35	2009	F
2709_1519_S225_	2013	D	10.05.2013	S225	12.1	15.5	Fam-35_2009_	S225	225	2273	35	2009	F
2709_1519_S226_	2013	D	10.05.2013	S226	15.1	30	Fam-47_2009_	S226	226	2274	47	2009	F
2709_1519_S227_	2013	D	10.05.2013	S227	13.5	22	Fam-47_2009_	S227	227	2275	47	2009	F
2709_1519_S228_	2013	D	10.05.2013	S228	13.5	20.5	Fam-40_2009_	S228	228	2276	40	2009	H
2709_1519_S229_	2013	D	10.05.2013	S229	14.8	25.5	Fam-47_2009_	S229	229	2277	47	2009	F
2709_1519_S230_	2013	D	10.05.2013	S230	13.4	18	Fam-43_2009_	S230	230	2278	43	2009	F
2709_1519_S231_	2013	D	10.05.2013	S231	12.5	16	Fam-36_2009_	S231	231	2279	36	2009	H

2709_1519_S232_	2013	D	10.05.2013	S232	13.6	20	Fam-47_2009_	S232	232	2280	47	2009	F
2709_1519_S233_	2013	D	10.05.2013	S233	15.3	29.5	Fam-28_2007_	S233	233	2281	28	2007	H
2709_1519_S234_	2013	D	10.05.2013	S234	14.8	28.5	Fam-43_2009_	S234	234	2282	43	2009	F
2709_1519_S235_	2013	D	10.05.2013	S235	14	22	Fam-40_2009_	S235	235	2283	40	2009	H
2709_1519_S236_	2013	D	10.05.2013	S236	13.6	17.5	Fam-44_2009_	S236	236	2284	44	2009	H
2709_1519_S237_	2013	D	10.05.2013	S237	14.2	25	Fam-71_2010_	S237	237	2285	71	2010	F
2709_1519_S238_	2013	D	10.05.2013	S238	15.3	29.5	Fam-47_2009_	S238	238	2286	47	2009	F
2709_1519_S239_	2013	D	10.05.2013	S239	14.4	25.5	Fam-31_2009_	S239	239	2287	31	2009	F
2709_1519_S240_	2013	D	10.05.2013	S240	13.6	20	Fam-36_2009_	S240	240	2288	36	2009	H
2710_1519_S241_	2013	D	10.05.2013	S241	13.3	19	Fam-36_2009_	S241	241	2289	36	2009	H
2710_1519_S242_	2013	D	10.05.2013	S242	14.2	23.5	Fam-45_2009_	S242	242	2290	45	2009	F
2710_1519_S243_	2013	D	10.05.2013	S243	13.3	20	Fam-47_2009_	S243	243	2291	47	2009	F
2710_1519_S244_	2013	D	10.05.2013	S244	13.1	18	Fam-39_2009_	S244	244	2292	39	2009	F
2710_1519_S245_	2013	D	10.05.2013	S245	13.7	21.5	Fam-36_2009_	S245	245	2293	36	2009	H

2710_1519_S246_	2013	D	10.05.2013	S246	13.9	21	Fam-38_2009_	S246	246	2294	38	2009	H
2710_1519_S247_	2013	D	10.05.2013	S247	12.7	18	Fam-54_2009_	S247	247	2295	54	2009	W
2710_1519_S248_	2013	D	10.05.2013	S248	13.5	20.5	Fam-80_2010_	S248	248	2296	80	2010	H
2710_1519_S249_	2013	D	10.05.2013	S249	14	23.5	Fam-45_2009_	S249	249	2297	45	2009	F
2710_1519_S250_	2013	D	10.05.2013	S250	13.2	18	Fam-37_2009_	S250	250	2298	37	2009	F
2710_1519_S251_	2013	D	10.05.2013	S251	13.2	21	Fam-47_2009_	S251	251	2299	47	2009	F
2710_1519_S252_	2013	D	10.05.2013	S252	15	27	Fam-04_2007_	S252	252	2300	4	2007	W
2710_1519_S253_	2013	D	10.05.2013	S253	13.8	21.5	Fam-31_2009_	S253	253	2301	31	2009	F
2710_1519_S254_	2013	D	10.05.2013	S254	13.8	21	Fam-37_2009_	S254	254	2302	37	2009	F
2710_1519_S255_	2013	D	10.05.2013	S255	12.9	18	Fam-44_2009_	S255	255	2303	44	2009	H
2710_1519_S256_	2013	D	10.05.2013	S256	14.9	28	Fam-39_2009_	S256	256	2304	39	2009	F
2710_1519_S257_	2013	D	10.05.2013	S257	14.5	25	Fam-36_2009_	S257	257	2305	36	2009	H
2710_1519_S258_	2013	D	10.05.2013	S258	12.5	16	Fam-43_2009_	S258	258	2306	43	2009	F
2710_1519_S259_	2013	D	10.05.2013	S259	13.5	21	Fam-31_2009_	S259	259	2307	31	2009	F

2710_1519_S260_	2013	D	10.05.2013	S260	13	18	Fam-50_2009_	S260	260	2308	50	2009 H
2710_1519_S261_	2013	D	10.05.2013	S261	12.4	15	Fam-38_2009_	S261	261	2309	38	2009 H
2710_1519_S262_	2013	D	10.05.2013	S262	14.3	23	Fam-49_2009_	S262	262	2310	49	2009 F
2710_1519_S263_	2013	D	10.05.2013	S263	12.8	19	Fam-47_2009_	S263	263	2311	47	2009 F
2710_1519_S264_	2013	D	10.05.2013	S264	13.5	20	Fam-49_2009_	S264	264	2312	49	2009 F
2710_1519_S265_	2013	D	10.05.2013	S265	14.4	24.5	Fam-39_2009_	S265	265	2313	39	2009 F
2710_1519_S267_	2013	D	10.05.2013	S267	13.8	21	Fam-39_2009_	S267	267	2314	39	2009 F
2710_1519_S268_	2013	D	10.05.2013	S268	13.8	21	Fam-49_2009_	S268	268	2315	49	2009 F
2710_1519_S269_	2013	D	10.05.2013	S269	14.6	24.5	Fam-35_2009_	S269	269	2316	35	2009 F
2710_1519_S270_	2013	D	10.05.2013	S270	13.6	18.5	Fam-43_2009_	S270	270	2317	43	2009 F
2710_1519_S271_	2013	D	10.05.2013	S271	13.2	19.5	Fam-33_2009_	S271	271	2318	33	2009 F
2710_1519_S272_	2013	D	10.05.2013	S272	13.8	20.5	Fam-39_2009_	S272	272	2319	39	2009 F
2710_1519_S273_	2013	D	10.05.2013	S273	12.6	16.5	Fam-37_2009_	S273	273	2320	37	2009 F
2710_1519_S274_	2013	D	10.05.2013	S274	13.2	19	Fam-50_2009_	S274	274	2321	50	2009 H

2710_1519_S275_	2013	D	10.05.2013	S275	13.9	22	Fam-51_2009_	S275	275	2322	51	2009	W
2710_1519_S276_	2013	D	10.05.2013	S276	15	27.5	Fam-48_2009_	S276	276	2323	48	2009	H
2710_1519_S277_	2013	D	10.05.2013	S277	12.3	17	Fam-44_2009_	S277	277	2324	44	2009	H
2710_1519_S278_	2013	D	10.05.2013	S278	13.5	19.5	Fam-35_2009_	S278	278	2325	35	2009	F
2710_1519_S279_	2013	D	10.05.2013	S279	13.2	18.5	Fam-38_2009_	S279	279	2326	38	2009	H
2710_1519_S280_	2013	D	10.05.2013	S280	14	23.5	Fam-33_2009_	S280	280	2327	33	2009	F
2710_1519_S281_	2013	D	10.05.2013	S281	13.5	19.5	Fam-35_2009_	S281	281	2328	35	2009	F
2710_1519_S282_	2013	D	10.05.2013	S282	13	17	Fam-38_2009_	S282	282	2329	38	2009	H
2710_1520_S283_	2013	D	10.05.2013	S283	12.2	18	Fam-38_2009_	S283	283	2330	38	2009	H
2710_1520_S284_	2013	D	10.05.2013	S284	15.2	26	Fam-39_2009_	S284	284	2331	39	2009	F
2710_1520_S285_	2013	D	10.05.2013	S285	13.6	16.5	Fam-44_2009_	S285	285	2332	44	2009	H
2710_1520_S286_	2013	D	10.05.2013	S286	13.8	22	Fam-47_2009_	S286	286	2333	47	2009	F
2710_1520_S287_	2013	D	10.05.2013	S287	14	22.5	Fam-03_2007_	S287	287	2334	3	2007	W
2710_1520_S289_	2013	D	10.05.2013	S289	13.2	19.5	Fam-49_2009_	S289	289	2335	49	2009	F

2710_1520_S290_	2013	D	10.05.2013	S290	13.1	17.5	Fam-51_2009_S290	290	2336	51	2009	W
2710_1520_S291_	2013	D	10.05.2013	S291	13.6	21	Fam-31_2009_S291	291	2337	31	2009	F
2710_1520_S292_	2013	D	10.05.2013	S292	13.3	19.5	Fam-80_2010_S292	292	2338	80	2010	H
2710_1520_S293_	2013	D	10.05.2013	S293	13	18.5	Fam-58_2009_S293	293	2339	58	2009	W
2710_1520_S294_	2013	D	10.05.2013	S294	13.4	20.5	Fam-45_2009_S294	294	2340	45	2009	F
2710_1520_S295_	2013	D	10.05.2013	S295	13.2	18	Fam-35_2009_S295	295	2341	35	2009	F
2710_1520_S296_	2013	D	10.05.2013	S296	13.7	21.5	Fam-47_2009_S296	296	2342	47	2009	F
2710_1520_S297_	2013	D	10.05.2013	S297	14.5	23.5	Fam-39_2009_S297	297	2343	39	2009	F
2710_1520_S298_	2013	D	10.05.2013	S298	15.2	28.5	Fam-51_2009_S298	298	2344	51	2009	W
2710_1520_S299_	2013	D	10.05.2013	S299	15.5	30	Fam-47_2009_S299	299	2345	47	2009	F
2710_1520_S300_	2013	D	10.05.2013	S300	13.5	21	Fam-49_2009_S300	300	2346	49	2009	F
2710_1520_S301_	2013	D	10.05.2013	S301	13.7	21.5	Fam-33_2009_S301	301	2347	33	2009	F
2710_1520_S302_	2013	D	10.05.2013	S302	13.6	20.5	Fam-35_2009_S302	302	2348	35	2009	F
2710_1520_S303_	2013	D	10.05.2013	S303	13.1	18.5	Fam-43_2009_S303	303	2349	43	2009	F

2710_1520_S304_	2013	D	10.05.2013	S304	13	17.5	Fam-47_2009_S304	304	2350	47	2009	F
2710_1520_S305_	2013	D	10.05.2013	S305	12.6	19	Fam-79_2010_S305	305	2351	79	2010	F
2710_1520_S306_	2013	D	10.05.2013	S306	13.1	18	Fam-35_2009_S306	306	2352	35	2009	F
2710_1520_S307_	2013	D	10.05.2013	S307	13.7	22	Fam-47_2009_S307	307	2353	47	2009	F
2710_1520_S308_	2013	D	10.05.2013	S308	12.7	17	Fam-58_2009_S308	308	2354	58	2009	W
2710_1520_S309_	2013	D	10.05.2013	S309	15.2	27.5	Fam-37_2009_S309	309	2355	37	2009	F
2710_1520_S310_	2013	D	10.05.2013	S310	15.2	27.5	Fam-27_2007_S310	310	2356	27	2007	F
2710_1520_S312_	2013	D	10.05.2013	S312	14.3	24	Fam-47_2009_S312	312	2357	47	2009	F
2710_1520_S313_	2013	D	10.05.2013	S313	16	35.5	Fam-11_2007_S313	313	2358	11	2007	F
2710_1520_S314_	2013	D	10.05.2013	S314	13.2	17.5	Fam-43_2009_S314	314	2359	43	2009	F
2710_1520_S315_	2013	D	10.05.2013	S315	12.4	16	Fam-38_2009_S315	315	2360	38	2009	H
2710_1520_S316_	2013	D	10.05.2013	S316	13.2	20	Fam-47_2009_S316	316	2361	47	2009	F
2710_1520_S317_	2013	D	10.05.2013	S317	14.4	22.5	Fam-43_2009_S317	317	2362	43	2009	F
2710_1520_S318_	2013	D	10.05.2013	S318	12.5	15.5	Fam-51_2009_S318	318	2363	51	2009	W

2710_1520_S319_	2013	D	10.05.2013	S319	13.7	20.5	Fam-47_2009_S319	319	2364	47	2009	F
2710_1520_S320_	2013	D	10.05.2013	S320	14.7	25	Fam-57_2009_S320	320	2365	57	2009	W
2711_1520_S321_	2013	D	10.05.2013	S321	13.5	19.5	Fam-40_2009_S321	321	2366	40	2009	H
2711_1520_S322_	2013	D	10.05.2013	S322	12.1	15	Fam-39_2009_S322	322	2367	39	2009	F
2711_1520_S323_	2013	D	10.05.2013	S323	13.1	17.5	Fam-45_2009_S323	323	2368	45	2009	F
2711_1520_S324_	2013	D	10.05.2013	S324	13.4	20.5	Fam-41_2009_S324	324	2369	41	2009	F
2711_1520_S325_	2013	D	10.05.2013	S325	15.2	30	Fam-28_2007_S325	325	2370	28	2007	H
2711_1520_S326_	2013	D	10.05.2013	S326	13.2	20	Fam-58_2009_S326	326	2371	58	2009	W
2711_1520_S327_	2013	D	10.05.2013	S327	11.3	12	Fam-44_2009_S327	327	2372	44	2009	H
2711_1520_S328_	2013	D	10.05.2013	S328	13.4	20	Fam-31_2009_S328	328	2373	31	2009	F
2711_1520_S329_	2013	D	10.05.2013	S329	14.1	24	Fam-77_2010_S329	329	2374	77	2010	F
2711_1520_S330_	2013	D	10.05.2013	S330	13.4	19	Fam-40_2009_S330	330	2375	40	2009	H
2711_1520_S331_	2013	D	10.05.2013	S331	12.9	17.5	Fam-58_2009_S331	331	2376	58	2009	W
2711_1520_S332_	2013	D	16.05.2013	S332	13.1	19.5	Fam-48_2009_S332	332	2377	48	2009	H

2711_1520_S333_	2013	D	16.05.2013	S333	15.4	35	Fam-51_2009_S333	333	2378	51	2009	W
2711_1520_S334_	2013	D	16.05.2013	S334	14.8	31.5	Fam-03_2007_S334	334	2379	3	2007	W
2711_1520_S335_	2013	D	16.05.2013	S335	12.1	17.5	Fam-70_2010_S335	335	2380	70	2010	H
2711_1520_S336_	2013	D	16.05.2013	S336	12.4	17.5	Fam-75_2010_S336	336	2381	75	2010	F
2711_1520_S337_	2013	D	16.05.2013	S337	13.6	18	Fam-51_2009_S337	337	2382	51	2009	W
2711_1520_S338_	2013	D	16.05.2013	S338	13.4	19	Fam-50_2009_S338	338	2383	50	2009	H
2711_1520_S339_	2013	D	16.05.2013	S339	14.5	25	Fam-74_2010_S339	339	2384	74	2010	H
2711_1520_S340_	2013	D	16.05.2013	S340	13.2	18	Fam-58_2009_S340	340	2385	58	2009	W
2711_1520_S341_	2013	D	16.05.2013	S341	13.8	20.5	Fam-45_2009_S341	341	2386	45	2009	F
2711_1520_S342_	2013	D	16.05.2013	S342	13	17	Fam-51_2009_S342	342	2387	51	2009	W
2711_1520_S343_	2013	D	16.05.2013	S343	13	16.5	Fam-38_2009_S343	343	2388	38	2009	H
2711_1520_S344_	2013	D	16.05.2013	S344	13.5	20	Fam-47_2009_S344	344	2389	47	2009	F
2711_1520_S345_	2013	D	16.05.2013	S345	12.2	15	Fam-44_2009_S345	345	2390	44	2009	H
2711_1520_S346_	2013	D	16.05.2013	S346	12.7	16.5	Fam-58_2009_S346	346	2391	58	2009	W

2711_1520_S347_	2013	D	16.05.2013	S347	13.5	19	Fam-43_2009_	S347	347	2392	43	2009	F
2711_1520_S348_	2013	D	16.05.2013	S348	13.8	20	Fam-38_2009_	S348	348	2393	38	2009	H
2711_1520_S349_	2013	D	20.05.2013	S349	14.3	23.5	Fam-39_2009_	S349	349	2394	39	2009	F
2711_1520_S350_	2013	D	20.05.2013	S350	12.7	16	Fam-51_2009_	S350	350	2395	51	2009	W
2711_1520_S351_	2013	D	20.05.2013	S351	13.7	20.5	Fam-51_2009_	S351	351	2396	51	2009	W
2711_1520_S352_	2013	D	20.05.2013	S352	14.7	24	Fam-52_2009_	S352	352	2397	52	2009	W
2711_1520_S353_	2013	D	20.05.2013	S353	13.5	21	Fam-44_2009_	S353	353	2398	44	2009	H
2711_1520_S354_	2013	D	20.05.2013	S354	14.5	24.5	Fam-43_2009_	S354	354	2399	43	2009	F
2711_1520_S355_	2013	D	20.05.2013	S355	14.2	23.5	Fam-03_2007_	S355	355	2400	3	2007	W
2711_1520_S356_	2013	D	20.05.2013	S356	12.8	19	Fam-42_2009_	S356	356	2401	42	2009	H
2711_1520_S357_	2013	D	20.05.2013	S357	14.7	24	Fam-43_2009_	S357	357	2402	43	2009	F
2711_1520_S358_	2013	D	20.05.2013	S358	13.1	16.5	Fam-57_2009_	S358	358	2403	57	2009	W
2711_1520_S359_	2013	D	20.05.2013	S359	13.3	14.5	Fam-38_2009_	S359	359	2404	38	2009	H
2711_1520_S360_	2013	D	20.05.2013	S360	13.4	18.5	Fam-69_2010_	S360	360	2405	69	2010	F

2711_1520_S361_	2013	D	20.05.2013	S361	13.5	19.5	Fam-54_2009_	S361	361	2406	54	2009	W
2711_1520_S362_	2013	D	20.05.2013	S362	13.8	19.5	Fam-89_2010_	S362	362	2407	89	2010	W
2711_1520_S363_	2013	D	20.05.2013	S363	15	28.5	Fam-03_2007_	S363	363	2408	3	2007	W
2711_1520_S364_	2013	D	20.05.2013	S364	14.2	21.5	Fam-43_2009_	S364	364	2409	43	2009	F
2711_1520_S365_	2013	D	20.05.2013	S365	13.9	19.5	Fam-49_2009_	S365	365	2410	49	2009	F
2711_1520_S366_	2013	D	20.05.2013	S366	12.7	16	Fam-39_2009_	S366	366	2411	39	2009	F
2711_1520_S367_	2013	D	20.05.2013	S367	13.5	18.5	Fam-58_2009_	S367	367	2412	58	2009	W
2711_1520_S368_	2013	D	20.05.2013	S368	12.2	15.5	Fam-45_2009_	S368	368	2413	45	2009	F
2711_1520_S369_	2013	D	20.05.2013	S369	13.5	19	Fam-27_2007_	S369	369	2414	27	2007	F
2711_1520_S370_	2013	D	20.05.2013	S370	13	17	Fam-55_2009_	S370	370	2415	55	2009	W
2711_1520_S371_	2013	D	20.05.2013	S371	13.8	22	Fam-50_2009_	S371	371	2416	50	2009	H
2711_1520_S372_	2013	D	20.05.2013	S372	12.5	16	Fam-43_2009_	S372	372	2417	43	2009	F
2711_1520_S373_	2013	D	20.05.2013	S373	13.5	18	Fam-53_2009_	S373	373	2418	53	2009	W
2711_1520_S374_	2013	D	20.05.2013	S374	12.9	18	Fam-49_2009_	S374	374	2419	49	2009	F

2711_1520_S375_	2013	D	20.05.2013	S375	12.6	15.5	Fam-59_2009_	S375	375	2420	59	2009 W
2711_1520_S376_	2013	D	20.05.2013	S376	15	27	Fam-40_2009_	S376	376	2421	40	2009 H
2711_1521_S377_	2013	D	20.05.2013	S377	13.6	20	Fam-39_2009_	S377	377	2422	39	2009 F
2711_1521_S378_	2013	D	20.05.2013	S378	13.5	21	Fam-41_2009_	S378	378	2423	41	2009 F
2711_1521_S379_	2013	D	20.05.2013	S379	14	20	Fam-59_2009_	S379	379	2424	59	2009 W
2711_1521_S380_	2013	D	20.05.2013	S380	13	18	Fam-44_2009_	S380	380	2425	44	2009 H
2711_1521_S381_	2013	D	20.05.2013	S381	16.6	37.5	Fam-10_2007_	S381	381	2426	10	2007 W
2711_1521_S382_	2013	D	20.05.2013	S382	15	29	Fam-09_2007_	S382	382	2427	9	2007 W
2711_1521_S383_	2013	D	20.05.2013	S383	14.4	23.5	Fam-31_2009_	S383	383	2428	31	2009 F
2711_1521_S384_	2013	D	20.05.2013	S384	13.5	19	Fam-32_2009_	S384	384	2429	32	2009 H
2711_1521_S385_	2013	D	20.05.2013	S385	12.6	16.5	Fam-39_2009_	S385	385	2430	39	2009 F
2711_1521_S386_	2013	D	20.05.2013	S386	13	17.5	Fam-44_2009_	S386	386	2431	44	2009 H
2711_1521_S387_	2013	D	20.05.2013	S387	13	17.5	Fam-52_2009_	S387	387	2432	52	2009 W
2711_1521_S388_	2013	D	20.05.2013	S388	15.1	25.5	Fam-49_2009_	S388	388	2433	49	2009 F

2711_1521_S389_	2013	D	20.05.2013	S389	13	16.5	Fam-43_2009_	S389	389	2434	43	2009	F
2711_1521_S390_	2013	D	20.05.2013	S390	12.4	16	Fam-42_2009_	S390	390	2435	42	2009	H
2711_1521_S391_	2013	D	20.05.2013	S391	17	20.5	Fam-50_2009_	S391	391	2436	50	2009	H
2711_1521_S393_	2013	D	20.05.2013	S393	12.6	16	Fam-62_1_201	S393	393	2437	62	2010	H
2711_1521_S394_	2013	D	20.05.2013	S394	14.8	25	Fam-53_2009_	S394	394	2438	53	2009	W
2711_1521_S395_	2013	D	20.05.2013	S395	14.2	22.5	Fam-49_2009_	S395	395	2439	49	2009	F
2711_1521_S396_	2013	D	20.05.2013	S396	14	22.5	Fam-42_2009_	S396	396	2440	42	2009	H
2711_1521_S397_	2013	D	20.05.2013	S397	13	19	Fam-31_2009_	S397	397	2441	31	2009	F
2711_1521_S398_	2013	D	20.05.2013	S398	15.1	27	Fam-27_2007_	S398	398	2442	27	2007	F
2711_1521_S399_	2013	D	20.05.2013	S399	15.2	29.5	Fam-03_2007_	S399	399	2443	3	2007	W
2711_1521_S400_	2013	D	20.05.2013	S400	13	17	Fam-51_2009_	S400	400	2444	51	2009	W
2712_1521_S401_	2013	D	20.05.2013	S401	13.5	19	Fam-52_2009_	S401	401	2445	52	2009	W
2712_1521_S402_	2013	D	20.05.2013	S402	15	26	Fam-50_2009_	S402	402	2446	50	2009	H
2712_1521_S403_	2013	D	20.05.2013	S403	13.1	18	Fam-57_2009_	S403	403	2447	57	2009	W

2712_1521_S404_	2013	D	20.05.2013	S404	14.2	22.5	Fam-45_2009_S404	404	2448	45	2009	F
2712_1521_S405_	2013	D	20.05.2013	S405	14.9	25.5	Fam-03_2007_S405	405	2449	3	2007	W
2712_1521_S406_	2013	D	20.05.2013	S406	15.3	31.5	Fam-03_2007_S406	406	2450	3	2007	W
2712_1521_S407_	2013	D	20.05.2013	S407	14	22.5	Fam-32_2009_S407	407	2451	32	2009	H
2712_1521_S408_	2013	D	20.05.2013	S408	12.3	14	Fam-55_2009_S408	408	2452	55	2009	W
2712_1521_S409_	2013	D	20.05.2013	S409	13.7	19.5	Fam-52_2009_S409	409	2453	52	2009	W
2712_1521_S410_	2013	D	20.05.2013	S410	14.5	23.5	Fam-43_2009_S410	410	2454	43	2009	F
2712_1521_S411_	2013	D	20.05.2013	S411	13.4	18.5	Fam-38_2009_S411	411	2455	38	2009	H
2712_1521_S412_	2013	D	20.05.2013	S412	14.5	24	Fam-51_2009_S412	412	2456	51	2009	W
2712_1521_S413_	2013	D	20.05.2013	S413	16.8	37.5	Fam-30_2007_S413	413	2457	30	2007	H
2712_1521_S414_	2013	D	20.05.2013	S414	14.2	21.5	Fam-45_2009_S414	414	2458	45	2009	F
2712_1521_S415_	2013	D	20.05.2013	S415	13	17.5	Fam-59_2009_S415	415	2459	59	2009	W
2712_1521_S416_	2013	D	20.05.2013	S416	12.9	18	Fam-47_2009_S416	416	2460	47	2009	F
2712_1521_S417_	2013	D	20.05.2013	S417	13.6	19.5	Fam-42_2009_S417	417	2461	42	2009	H

2712_1521_S418_	2013	D	20.05.2013	S418	13.9	21	Fam-09_2007_	S418	418	2462	9	2007	W
2712_1521_S419_	2013	D	20.05.2013	S419	13.6	18.5	Fam-52_2009_	S419	419	2463	52	2009	W
2712_1521_S420_	2013	D	20.05.2013	S420	14.5	23.5	Fam-39_2009_	S420	420	2464	39	2009	F
2712_1521_S421_	2013	D	20.05.2013	S421	13.2	17.5	Fam-51_2009_	S421	421	2465	51	2009	W
2712_1521_S422_	2013	D	20.05.2013	S422	12.5	17	Fam-59_2009_	S422	422	2466	59	2009	W
2712_1521_S423_	2013	D	20.05.2013	S423	13.6	20.5	Fam-50_2009_	S423	423	2467	50	2009	H
2712_1521_S424_	2013	D	20.05.2013	S424	13.4	18	Fam-51_2009_	S424	424	2468	51	2009	W
2712_1521_S425_	2013	D	20.05.2013	S425	13.4	18.5	Fam-40_2009_	S425	425	2469	40	2009	H
2712_1521_S426_	2013	D	20.05.2013	S426	13.7	20.5	Fam-42_2009_	S426	426	2470	42	2009	H
2712_1521_S427_	2013	D	20.05.2013	S427	13	17	Fam-58_2009_	S427	427	2471	58	2009	W
2712_1521_S428_	2013	D	20.05.2013	S428	13	17	Fam-54_2009_	S428	428	2472	54	2009	W
2712_1521_S429_	2013	D	20.05.2013	S429	14.3	23.5	Fam-51_2009_	S429	429	2473	51	2009	W
2712_1521_S430_	2013	D	20.05.2013	S430	14	20	Fam-51_2009_	S430	430	2474	51	2009	W
2712_1521_S431_	2013	D	20.05.2013	S431	12.8	16	Fam-51_2009_	S431	431	2475	51	2009	W

2712_1521_S432_	2013	D	20.05.2013	S432	14.4	23	Fam-38_2009_S432	432	2476	38	2009	H
2712_1521_S433_	2013	D	20.05.2013	S433	15	25.5	Fam-32_2009_S433	433	2477	32	2009	H
2712_1521_S434_	2013	D	20.05.2013	S434	13	16	Fam-58_2009_S434	434	2478	58	2009	W
2712_1521_S435_	2013	D	20.05.2013	S435	13	17	Fam-57_2009_S435	435	2479	57	2009	W
2712_1521_S436_	2013	D	20.05.2013	S436	13.5	18.5	Fam-57_2009_S436	436	2480	57	2009	W
2712_1521_S437_	2013	D	20.05.2013	S437	14.9	25	Fam-39_2009_S437	437	2481	39	2009	F
2712_1521_S438_	2013	D	20.05.2013	S438	13.3	18.5	Fam-39_2009_S438	438	2482	39	2009	F
2712_1521_S439_	2013	D	20.05.2013	S439	14.5	24.5	Fam-73_2010_S439	439	2483	73	2010	F
2712_1521_S440_	2013	D	20.05.2013	S440	14.3	23	Fam-49_2009_S440	440	2484	49	2009	F
2712_1521_S441_	2013	D	20.05.2013	S441	14.4	23	Fam-47_2009_S441	441	2485	47	2009	F
2712_1521_S442_	2013	D	20.05.2013	S442	14.3	21	Fam-44_2009_S442	442	2486	44	2009	H
2712_1521_S443_	2013	D	20.05.2013	S443	15.5	28	Fam-09_2007_S443	443	2487	9	2007	W
2712_1521_S444_	2013	D	20.05.2013	S444	13.5	20	Fam-38_2009_S444	444	2488	38	2009	H
2712_1521_S445_	2013	D	20.05.2013	S445	15.2	28.5	Fam-51_2009_S445	445	2489	51	2009	W

2712_1521_S446_	2013	D	20.05.2013	S446	14.2	22	Fam-39_2009_	S446	446	2490	39	2009	F
2712_1521_S447_	2013	D	20.05.2013	S447	12.9	16	Fam-50_2009_	S447	447	2491	50	2009	H
2712_1521_S448_	2013	D	20.05.2013	S448	14	23.5	Fam-63_2010_	S448	448	2492	63	2010	F
2712_1521_S449_	2013	D	20.05.2013	S449	15	27.5	Fam-39_2009_	S449	449	2493	39	2009	F
2712_1521_S450_	2013	D	20.05.2013	S450	14	24	Fam-10_2007_	S450	450	2494	10	2007	W
2712_1521_S451_	2013	D	20.05.2013	S451	13.9	20	Fam-40_2009_	S451	451	2495	40	2009	H
2712_1521_S452_	2013	D	20.05.2013	S452	13.5	19	Fam-48_2009_	S452	452	2496	48	2009	H
2712_1521_S453_	2013	D	20.05.2013	S453	13	17	Fam-47_2009_	S453	453	2497	47	2009	F
2712_1521_S454_	2013	D	20.05.2013	S454	15.5	27.5	Fam-34_2009_	S454	454	2498	34	2009	H
2712_1521_S455_	2013	D	20.05.2013	S455	12.5	14	Fam-58_2009_	S455	455	2499	58	2009	W
2712_1521_S456_	2013	D	20.05.2013	S456	13.8	18.5	Fam-51_2009_	S456	456	2500	51	2009	W
2712_1521_S457_	2013	D	20.05.2013	S457	13.5	21	Fam-35_2009_	S457	457	2501	35	2009	F
2712_1521_S458_	2013	D	20.05.2013	S458	12.4	15	Fam-40_2009_	S458	458	2502	40	2009	H
2712_1521_S459_	2013	D	20.05.2013	S459	15	23.5	Fam-59_2009_	S459	459	2503	59	2009	W

2712_1521_S460_	2013	D	20.05.2013	S460	13.8	19.5	Fam-45_2009_	S460	460	2504	45	2009 F
2712_1521_S461_	2013	D	20.05.2013	S461	13.8	22	Fam-52_2009_	S461	461	2505	52	2009 W
2712_1521_S462_	2013	D	20.05.2013	S462	13.6	20.5	Fam-58_2009_	S462	462	2506	58	2009 W
2712_1521_S463_	2013	D	20.05.2013	S463	14.5	22	Fam-59_2009_	S463	463	2507	59	2009 W
2712_1521_S464_	2013	D	20.05.2013	S464	14.1	22.5	Fam-58_2009_	S464	464	2508	58	2009 W
2712_1521_S465_	2013	D	20.05.2013	S465	16.9	35	Fam-40_2009_	S465	465	2509	40	2009 H
2712_1521_S466_	2013	D	20.05.2013	S466	14.4	22.5	Fam-50_2009_	S466	466	2510	50	2009 H
2712_1521_S467_	2013	D	20.05.2013	S467	14.5	22.5	Fam-32_2009_	S467	467	2511	32	2009 H
2712_1521_S468_	2013	D	20.05.2013	S468	11.9	13	Fam-38_2009_	S468	468	2512	38	2009 H
2712_1521_S469_	2013	D	20.05.2013	S469	14	24	Fam-32_2009_	S469	469	2513	32	2009 H
2712_1521_S470_	2013	D	20.05.2013	S470	14	21	Fam-43_2009_	S470	470	2514	43	2009 F
2712_1522_S471_	2013	D	20.05.2013	S471	15.2	28	Fam-03_2007_	S471	471	2515	3	2007 W
2712_1522_S472_	2013	D	20.05.2013	S472	12.6	15	Fam-59_2009_	S472	472	2516	59	2009 W
2712_1522_S473_	2013	D	20.05.2013	S473	13.2	19.5	Fam-44_2009_	S473	473	2517	44	2009 H

2712_1522_S474_	2013	D	20.05.2013	S474	13	16.5	Fam-49_2009_	S474	474	2518	49	2009	F
2712_1522_S475_	2013	D	20.05.2013	S475	14.3	23	Fam-49_2009_	S475	475	2519	49	2009	F
2712_1522_S476_	2013	D	20.05.2013	S476	13.2	19	Fam-34_2009_	S476	476	2520	34	2009	H
2712_1522_S477_	2013	D	20.05.2013	S477	14	22.5	Fam-05_2007_	S477	477	2521	5	2007	W
2712_1522_S478_	2013	D	20.05.2013	S478	14	21.5	Fam-75_2010_	S478	478	2522	75	2010	F
2712_1522_S479_	2013	D	20.05.2013	S479	15	18.5	Fam-56_2009_	S479	479	2523	56	2009	W
2712_1522_S480_	2013	D	20.05.2013	S480	14	20	Fam-39_2009_	S480	480	2524	39	2009	F
2713_1522_S481_	2013	D	20.05.2013	S481	15	24	Fam-50_2009_	S481	481	2525	50	2009	H
2713_1522_S482_	2013	D	20.05.2013	S482	14.2	22	Fam-50_2009_	S482	482	2526	50	2009	H
2713_1522_S483_	2013	D	20.05.2013	S483	14.2	23.5	Fam-41_2009_	S483	483	2527	41	2009	F
2713_1522_S484_	2013	D	20.05.2013	S484	15.4	31.5	Fam-35_2009_	S484	484	2528	35	2009	F
2713_1522_S486_	2013	D	20.05.2013	S486	14.5	24	Fam-79_2010_	S486	486	2529	79	2010	F
2713_1522_S487_	2013	D	20.05.2013	S487	13.2	18.5	Fam-33_2009_	S487	487	2530	33	2009	F
2713_1522_S488_	2013	D	20.05.2013	S488	14	20.5	Fam-39_2009_	S488	488	2531	39	2009	F

2713_1522_S489_	2013	D	20.05.2013	S489	14.8	24.5	Fam-59_2009_S489	489	2532	59	2009 W
2713_1522_S490_	2013	D	20.05.2013	S490	12.9	16	Fam-52_2009_S490	490	2533	52	2009 W
2713_1522_S491_	2013	D	20.05.2013	S491	13.2	17.5	Fam-57_2009_S491	491	2534	57	2009 W
2713_1522_S492_	2013	D	20.05.2013	S492	13	16.5	Fam-44_2009_S492	492	2535	44	2009 H
2713_1522_S493_	2013	D	20.05.2013	S493	13.6	19	Fam-38_2009_S493	493	2536	38	2009 H
2713_1522_S494_	2013	D	20.05.2013	S494	0	0	Fam-44_2009_S494	494	2537	44	2009 H
2713_1522_S495_	2013	D	20.05.2013	S495	12.7	17	Fam-57_2009_S495	495	2538	57	2009 W
2713_1522_S496_	2013	D	20.05.2013	S496	13.5	19.5	Fam-06_2007_S496	496	2539	6	2007 W
2713_1522_S497_	2013	D	20.05.2013	S497	12.6	16.5	Fam-44_2009_S497	497	2540	44	2009 H
2713_1522_S498_	2013	D	20.05.2013	S498	12.5	15.5	Fam-59_2009_S498	498	2541	59	2009 W
2713_1522_S500_	2013	D	20.05.2013	S500	13.2	19.5	Fam-58_2009_S500	500	2542	58	2009 W
2713_1522_S501_	2013	D	20.05.2013	S501	13.6	20	Fam-39_2009_S501	501	2543	39	2009 F
2713_1522_S502_	2013	D	20.05.2013	S502	13	17.5	Fam-54_2009_S502	502	2544	54	2009 W
2713_1522_S503_	2013	D	20.05.2013	S503	13.3	19	Fam-54_2009_S503	503	2545	54	2009 W

2713_1522_S504_	2013	D	20.05.2013	S504	11.5	12.5	Fam-52_2009_	S504	504	2546	52	2009	W
2713_1522_S505_	2013	D	20.05.2013	S505	13.7	19	Fam-39_2009_	S505	505	2547	39	2009	F
2713_1522_S506_	2013	D	20.05.2013	S506	12.9	17	Fam-54_2009_	S506	506	2548	54	2009	W
2713_1522_S507_	2013	D	20.05.2013	S507	12.4	14	Fam-51_2009_	S507	507	2549	51	2009	W
2713_1522_S508_	2013	D	20.05.2013	S508	12.4	14.5	Fam-58_2009_	S508	508	2550	58	2009	W
2713_1522_S509_	2013	D	20.05.2013	S509	14.9	26	Fam-03_2007_	S509	509	2551	3	2007	W
2713_1522_S510_	2013	D	20.05.2013	S510	15	28	Fam-09_2007_	S510	510	2552	9	2007	W
2713_1522_S511_	2013	D	20.05.2013	S511	12.6	17	Fam-60_2009_	S511	511	2553	60	2009	W
2713_1522_S512_	2013	D	20.05.2013	S512	13.6	19.5	Fam-58_2009_	S512	512	2554	58	2009	W
2713_1522_S514_	2013	D	20.05.2013	S514	12.2	14	Fam-36_2009_	S514	514	2555	36	2009	H
2713_1522_S515_	2013	D	20.05.2013	S515	13.2	19	Fam-58_2009_	S515	515	2556	58	2009	W
2713_1522_S516_	2013	D	20.05.2013	S516	14	22	Fam-34_2009_	S516	516	2557	34	2009	H
2713_1522_S517_	2013	D	20.05.2013	S517	11.6	13.5	Fam-56_2009_	S517	517	2558	56	2009	W
2713_1522_S518_	2013	D	20.05.2013	S518	14.9	27.5	Fam-19_2007_	S518	518	2559	19	2007	F

2713_1522_S519_	2013	D	20.05.2013	S519	12.5	15	Fam-59_2009_S519	519	2560	59	2009	W
2713_1522_S520_	2013	D	20.05.2013	S520	12.5	17	Fam-42_2009_S520	520	2561	42	2009	H
2713_1522_S521_	2013	D	20.05.2013	S521	11.1	13	Fam-76_2010_S521	521	2562	76	2010	H
2713_1522_S522_	2013	D	20.05.2013	S522	12.6	18.5	Fam-68_2010_S522	522	2563	68	2010	H
2713_1522_S523_	2013	D	20.05.2013	S523	12.9	18	Fam-59_2009_S523	523	2564	59	2009	W
2713_1522_S524_	2013	D	20.05.2013	S524	14.4	22.5	Fam-40_2009_S524	524	2565	40	2009	H
2713_1522_S525_	2013	D	20.05.2013	S525	16.2	32	Fam-51_2009_S525	525	2566	51	2009	W
2713_1522_S526_	2013	D	20.05.2013	S526	13	18.5	Fam-44_2009_S526	526	2567	44	2009	H
2713_1522_S527_	2013	D	20.05.2013	S527	13.9	20	Fam-49_2009_S527	527	2568	49	2009	F
2713_1522_S528_	2013	D	20.05.2013	S528	13.3	19	Fam-59_2009_S528	528	2569	59	2009	W
2713_1522_S529_	2013	D	20.05.2013	S529	12.5	17	Fam-54_2009_S529	529	2570	54	2009	W
2713_1522_S530_	2013	D	20.05.2013	S530	12.5	16	Fam-54_2009_S530	530	2571	54	2009	W
2713_1522_S531_	2013	D	20.05.2013	S531	16.8	37	Fam-51_2009_S531	531	2572	51	2009	W
2713_1522_S532_	2013	D	20.05.2013	S532	12.6	17	Fam-41_2009_S532	532	2573	41	2009	F

2713_1522_S533_	2013	D	20.05.2013	S533	14.6	26	Fam-04_2007_S533	533	2574	4	2007 W
2713_1522_S534_	2013	D	20.05.2013	S534	13.2	17.5	Fam-58_2009_S534	534	2575	58	2009 W
2713_1522_S535_	2013	D	20.05.2013	S535	13.8	21.5	Fam-34_2009_S535	535	2576	34	2009 H
2713_1522_S536_	2013	D	20.05.2013	S536	12	13.5	Fam-51_2009_S536	536	2577	51	2009 W
2713_1522_S537_	2013	D	20.05.2013	S537	14.4	25	Fam-09_2007_S537	537	2578	9	2007 W
2713_1522_S538_	2013	D	20.05.2013	S538	13.6	22.5	Fam-56_2009_S538	538	2579	56	2009 W
2713_1522_S539_	2013	D	22.05.2013	S539	13	18.5	Fam-58_2009_S539	539	2580	58	2009 W
2713_1522_S540_	2013	D	22.05.2013	S540	15.5	30	Fam-09_2007_S540	540	2581	9	2007 W
2713_1522_S541_	2013	D	22.05.2013	S541	12.6	17	Fam-73_2010_S541	541	2582	73	2010 F
2713_1522_S542_	2013	D	22.05.2013	S542	14.5	25	Fam-51_2009_S542	542	2583	51	2009 W
2713_1522_S543_	2013	D	22.05.2013	S543	13.5	21	Fam-39_2009_S543	543	2584	39	2009 F
2713_1522_S544_	2013	D	22.05.2013	S544	13	17	Fam-57_2009_S544	544	2585	57	2009 W
2713_1522_S545_	2013	D	22.05.2013	S545	12.4	16.5	Fam-42_2009_S545	545	2586	42	2009 H
2713_1522_S546_	2013	D	22.05.2013	S546	14.5	22.5	Fam-43_2009_S546	546	2587	43	2009 F

2713_1522_S547_	2013	D	22.05.2013	S547	12.6	16	Fam-59_2009_	S547	547	2588	59	2009 W
2713_1522_S548_	2013	D	22.05.2013	S548	11.9	15	Fam-61_1_201	S548	548	2589	61	2010 F
2713_1522_S549_	2013	D	22.05.2013	S549	13.5	20	Fam-04_2007_	S549	549	2590	4	2007 W
2713_1522_S550_	2013	D	22.05.2013	S550	12.2	16	Fam-44_2009_	S550	550	2591	44	2009 H
2713_1522_S551_	2013	D	22.05.2013	S551	14.2	24	Fam-52_2009_	S551	551	2592	52	2009 W
2713_1522_S552_	2013	D	22.05.2013	S552	15.1	26	Fam-69_2010_	S552	552	2593	69	2010 F
2713_1522_S553_	2013	D	22.05.2013	S553	14.5	23.5	Fam-50_2009_	S553	553	2594	50	2009 H
2713_1522_S554_	2013	D	22.05.2013	S554	13.2	19	Fam-55_2009_	S554	554	2595	55	2009 W
2713_1522_S555_	2013	D	22.05.2013	S555	14.6	26	Fam-03_2007_	S555	555	2596	3	2007 W
2713_1522_S556_	2013	D	22.05.2013	S556	14.4	24	Fam-74_2010_	S556	556	2597	74	2010 H
2713_1522_S557_	2013	D	22.05.2013	S557	14.5	24.5	Fam-34_2009_	S557	557	2598	34	2009 H
2713_1522_S558_	2013	D	22.05.2013	S558	14	21.5	Fam-40_2009_	S558	558	2599	40	2009 H
2713_1522_S559_	2013	D	22.05.2013	S559	12.2	16	Fam-56_2009_	S559	559	2600	56	2009 W
2713_1522_S560_	2013	D	22.05.2013	S560	12.5	17	Fam-44_2009_	S560	560	2601	44	2009 H

2714_1522_S561_	2013	D	22.05.2013	S561	13.4	20.5	Fam-42_2009_	S561	561	2602	42	2009 H
2714_1522_S562_	2013	D	22.05.2013	S562	15.5	30.5	Fam-09_2007_	S562	562	2603	9	2007 W
2714_1522_S563_	2013	D	22.05.2013	S563	14	23	Fam-45_2009_	S563	563	2604	45	2009 F
2714_1522_S564_	2013	D	22.05.2013	S564	13.2	18	Fam-52_2009_	S564	564	2605	52	2009 W
2714_1523_S565_	2013	D	22.05.2013	S565	13.2	18.5	Fam-44_2009_	S565	565	2606	44	2009 H
2714_1523_S566_	2013	D	22.05.2013	S566	13	17.5	Fam-67_2010_	S566	566	2607	67	2010 F
2714_1523_S567_	2013	D	22.05.2013	S567	15.7	19.5	Fam-53_2009_	S567	567	2608	53	2009 W
2714_1523_S568_	2013	D	22.05.2013	S568	13.1	16.5	Fam-54_2009_	S568	568	2609	54	2009 W
2714_1523_S569_	2013	D	22.05.2013	S569	13.1	18	Fam-37_2009_	S569	569	2610	37	2009 F
2714_1523_S570_	2013	D	22.05.2013	S570	13.3	20	Fam-39_2009_	S570	570	2611	39	2009 F
2714_1523_S571_	2013	D	22.05.2013	S571	13.8	22	Fam-44_2009_	S571	571	2612	44	2009 H
2714_1523_S572_	2013	D	22.05.2013	S572	15.2	28	Fam-03_2007_	S572	572	2613	3	2007 W
2714_1523_S573_	2013	D	22.05.2013	S573	12.6	16.5	Fam-41_2009_	S573	573	2614	41	2009 F
2714_1523_S574_	2013	D	22.05.2013	S574	15.5	30	Fam-30_2007_	S574	574	2615	30	2007 H

2714_1523_S575_	2013	D	22.05.2013	S575	15.5	29	Fam-60_2009_S575	575	2616	60	2009	W
2714_1523_S576_	2013	D	22.05.2013	S576	13	18.5	Fam-44_2009_S576	576	2617	44	2009	H
2714_1523_S577_	2013	D	22.05.2013	S577	12	13.5	Fam-59_2009_S577	577	2618	59	2009	W
2714_1523_S578_	2013	D	22.05.2013	S578	13.7	19.5	Fam-42_2009_S578	578	2619	42	2009	H
2714_1523_S579_	2013	D	22.05.2013	S579	13.8	21	Fam-58_2009_S579	579	2620	58	2009	W
2714_1523_S580_	2013	D	22.05.2013	S580	14	21	Fam-55_2009_S580	580	2621	55	2009	W
2714_1523_S581_	2013	D	22.05.2013	S581	13.1	18	Fam-45_2009_S581	581	2622	45	2009	F
2714_1523_S582_	2013	D	22.05.2013	S582	14	22	Fam-40_2009_S582	582	2623	40	2009	H
2714_1523_S583_	2013	D	22.05.2013	S583	12.9	18	Fam-54_2009_S583	583	2624	54	2009	W
2714_1523_S584_	2013	D	22.05.2013	S584	12.9	15.5	Fam-51_2009_S584	584	2625	51	2009	W
2714_1523_S585_	2013	D	22.05.2013	S585	13	17.5	Fam-40_2009_S585	585	2626	40	2009	H
2714_1523_S587_	2013	D	22.05.2013	S587	13	17.5	Fam-60_2009_S587	587	2627	60	2009	W
2714_1523_S588_	2013	D	22.05.2013	S588	12.4	16	Fam-42_2009_S588	588	2628	42	2009	H
2714_1523_S589_	2013	D	22.05.2013	S589	13.5	19	Fam-52_2009_S589	589	2629	52	2009	W

2714_1523_S590_	2013	D	22.05.2013	S590	13.6	20.5	Fam-41_2009_	S590	590	2630	41	2009 F
2714_1523_S591_	2013	D	22.05.2013	S591	14.5	24	Fam-03_2007_	S591	591	2631	3	2007 W
2714_1523_S592_	2013	D	22.05.2013	S592	12.8	16.5	Fam-52_2009_	S592	592	2632	52	2009 W
2714_1523_S593_	2013	D	22.05.2013	S593	12.4	15.3	Fam-44_2009_	S593	593	2633	44	2009 H
2714_1523_S594_	2013	D	22.05.2013	S594	12.9	17	Fam-60_2009_	S594	594	2634	60	2009 W
2714_1523_S595_	2013	D	22.05.2013	S595	13.1	19	Fam-42_2009_	S595	595	2635	42	2009 H
2714_1523_S596_	2013	D	22.05.2013	S596	15.1	27	Fam-51_2009_	S596	596	2636	51	2009 W
2714_1523_S597_	2013	D	22.05.2013	S597	14	23	Fam-79_2010_	S597	597	2637	79	2010 F
2714_1523_S598_	2013	D	22.05.2013	S598	12.8	15.5	Fam-59_2009_	S598	598	2638	59	2009 W
2714_1523_S599_	2013	D	22.05.2013	S599	13.2	20	Fam-42_2009_	S599	599	2639	42	2009 H
2714_1523_S600_	2013	D	22.05.2013	S600	14.8	25	Fam-32_2009_	S600	600	2640	32	2009 H
2714_1523_S601_	2013	D	22.05.2013	S601	12.5	17.5	Fam-44_2009_	S601	601	2641	44	2009 H
2714_1523_S602_	2013	D	22.05.2013	S602	11.8	13	Fam-85_2010_	S602	602	2642	85	2010 W
2714_1523_S603_	2013	D	22.05.2013	S603	13.2	19.5	Fam-61_1_201	S603	603	2643	61	2010 F

2714_1523_S604_	2013	D	22.05.2013	S604	13.5	20.5	Fam-42_2009_S604	604	2644	42	2009	H
2714_1523_S605_	2013	D	22.05.2013	S605	13	18	Fam-38_2009_S605	605	2645	38	2009	H
2714_1523_S606_	2013	D	22.05.2013	S606	15.7	17.5	Fam-44_2009_S606	606	2646	44	2009	H
2714_1523_S607_	2013	D	22.05.2013	S607	11.8	12	Fam-49_2009_S607	607	2647	49	2009	F
2714_1523_S608_	2013	D	22.05.2013	S608	13	16.5	Fam-52_2009_S608	608	2648	52	2009	W
2714_1523_S609_	2013	D	22.05.2013	S609	12	14	Fam-38_2009_S609	609	2649	38	2009	H
2714_1523_S610_	2013	D	22.05.2013	S610	13.8	20.5	Fam-04_2007_S610	610	2650	4	2007	W
2714_1523_S611_	2013	D	22.05.2013	S611	14.9	24	Fam-53_2009_S611	611	2651	53	2009	W
2714_1523_S612_	2013	D	22.05.2013	S612	14	22.5	Fam-52_2009_S612	612	2652	52	2009	W
2714_1523_S613_	2013	D	22.05.2013	S613	13.9	21.5	Fam-45_2009_S613	613	2653	45	2009	F
2714_1523_S614_	2013	D	22.05.2013	S614	13	16.5	Fam-44_2009_S614	614	2654	44	2009	H
2714_1523_S615_	2013	D	22.05.2013	S615	14.1	22	Fam-42_2009_S615	615	2655	42	2009	H
2714_1523_S616_	2013	D	22.05.2013	S616	14.5	24	Fam-34_2009_S616	616	2656	34	2009	H
2714_1523_S617_	2013	D	22.05.2013	S617	14.4	23.5	Fam-56_2009_S617	617	2657	56	2009	W

2714_1523_S618_	2013	D	22.05.2013	S618	13.5	20.5	Fam-41_2009_	S618	618	2658	41	2009	F
2714_1523_S619_	2013	D	22.05.2013	S619	13	18	Fam-59_2009_	S619	619	2659	59	2009	W
2714_1523_S620_	2013	D	22.05.2013	S620	13	17.5	Fam-52_2009_	S620	620	2660	52	2009	W
2714_1523_S621_	2013	D	22.05.2013	S621	14.9	25.5	Fam-03_2007_	S621	621	2661	3	2007	W
2714_1523_S622_	2013	D	22.05.2013	S622	13.5	19	Fam-55_2009_	S622	622	2662	55	2009	W
2714_1523_S623_	2013	D	22.05.2013	S623	13.9	21.5	Fam-55_2009_	S623	623	2663	55	2009	W
2714_1523_S624_	2013	D	22.05.2013	S624	16.5	38	Fam-04_2007_	S624	624	2664	4	2007	W
2714_1523_S625_	2013	D	22.05.2013	S625	12.5	15.5	Fam-57_2009_	S625	625	2665	57	2009	W
2714_1523_S626_	2013	D	22.05.2013	S626	15	28	Fam-34_2009_	S626	626	2666	34	2009	H
2714_1523_S627_	2013	D	22.05.2013	S627	12.9	17.5	Fam-40_2009_	S627	627	2667	40	2009	H
2714_1523_S628_	2013	D	22.05.2013	S628	13.7	21	Fam-06_2007_	S628	628	2668	6	2007	W
2714_1523_S629_	2013	D	22.05.2013	S629	13.9	20	Fam-50_2009_	S629	629	2669	50	2009	H
2714_1523_S630_	2013	D	22.05.2013	S630	14.5	23.5	Fam-49_2009_	S630	630	2670	49	2009	F
2714_1523_S631_	2013	D	22.05.2013	S631	13.5	19	Fam-59_2009_	S631	631	2671	59	2009	W

2714_1523_S632_	2013	D	22.05.2013	S632	14	21	Fam-52_2009_	S632	632	2672	52	2009	W
2714_1523_S633_	2013	D	22.05.2013	S633	13.4	18	Fam-34_2009_	S633	633	2673	34	2009	H
2714_1523_S634_	2013	D	22.05.2013	S634	14.9	24	Fam-40_2009_	S634	634	2674	40	2009	H
2714_1523_S635_	2013	D	22.05.2013	S635	12.7	15.5	Fam-50_2009_	S635	635	2675	50	2009	H
2714_1523_S636_	2013	D	22.05.2013	S636	13.4	19	Fam-52_2009_	S636	636	2676	52	2009	W
2714_1523_S637_	2013	D	22.05.2013	S637	14.2	23.5	Fam-54_2009_	S637	637	2677	54	2009	W
2714_1523_S638_	2013	D	22.05.2013	S638	14.3	22.5	Fam-34_2009_	S638	638	2678	34	2009	H
2714_1523_S639_	2013	D	22.05.2013	S639	14.5	23	Fam-34_2009_	S639	639	2679	34	2009	H
2714_1523_S640_	2013	D	22.05.2013	S640	12	14	Fam-38_2009_	S640	640	2680	38	2009	H
2715_1523_S641_	2013	D	22.05.2013	S641	14.4	23.5	Fam-34_2009_	S641	641	2681	34	2009	H
2715_1523_S642_	2013	D	22.05.2013	S642	13.5	19	Fam-58_2009_	S642	642	2682	58	2009	W
2715_1523_S643_	2013	D	22.05.2013	S643	13.5	19	Fam-32_2009_	S643	643	2683	32	2009	H
2715_1523_S644_	2013	D	22.05.2013	S644	15.8	30	Fam-34_2009_	S644	644	2684	34	2009	H
2715_1523_S645_	2013	D	22.05.2013	S645	13.5	19	Fam-54_2009_	S645	645	2685	54	2009	W

2715_1523_S646_	2013	D	22.05.2013	S646	15.4	25.5	Fam-10_2007_ S646	646	2686	10	2007	W
2715_1523_S647_	2013	D	22.05.2013	S647	15.5	29	Fam-54_2009_ S647	647	2687	54	2009	W
2715_1523_S648_	2013	D	22.05.2013	S648	14.5	25	Fam-56_2009_ S648	648	2688	56	2009	W
2715_1523_S649_	2013	D	22.05.2013	S649	13.8	21.5	Fam-56_2009_ S649	649	2689	56	2009	W
2715_1523_S650_	2013	D	22.05.2013	S650	14.4	24	Fam-10_2007_ S650	650	2690	10	2007	W
2715_1523_S651_	2013	D	22.05.2013	S651	13	17	Fam-51_2009_ S651	651	2691	51	2009	W
2715_1523_S652_	2013	D	22.05.2013	S652	13.7	19.5	Fam-57_2009_ S652	652	2692	57	2009	W
2715_1523_S653_	2013	D	22.05.2013	S653	13.3	19	Fam-34_2009_ S653	653	2693	34	2009	H
2715_1523_S654_	2013	D	22.05.2013	S654	13.2	19	Fam-60_2009_ S654	654	2694	60	2009	W
2715_1523_S655_	2013	D	22.05.2013	S655	12.5	16	Fam-59_2009_ S655	655	2695	59	2009	W
2715_1523_S656_	2013	D	22.05.2013	S656	13.2	19	Fam-52_2009_ S656	656	2696	52	2009	W
2715_1523_S657_	2013	D	22.05.2013	S657	13.2	19	Fam-58_2009_ S657	657	2697	58	2009	W
2715_1523_S658_	2013	D	22.05.2013	S658	15	24.5	Fam-34_2009_ S658	658	2698	34	2009	H
2715_1524_S659_	2013	D	22.05.2013	S659	13	18.5	Fam-60_2009_ S659	659	2699	60	2009	W

2715_1524_S660_	2013	D	22.05.2013	S660	12.5	15.5	Fam-52_2009_S660	660	2700	52	2009	W
2715_1524_S661_	2013	D	22.05.2013	S661	13.9	21	Fam-58_2009_S661	661	2701	58	2009	W
2715_1524_S662_	2013	D	22.05.2013	S662	13.2	18	Fam-59_2009_S662	662	2702	59	2009	W
2715_1524_S663_	2013	D	22.05.2013	S663	13.4	19.5	Fam-49_2009_S663	663	2703	49	2009	F
2715_1524_S664_	2013	D	22.05.2013	S664	14.5	25.5	Fam-49_2009_S664	664	2704	49	2009	F
2715_1524_S665_	2013	D	22.05.2013	S665	12.6	14	Fam-50_2009_S665	665	2705	50	2009	H
2715_1524_S667_	2013	D	22.05.2013	S667	13.6	19	Fam-34_2009_S667	667	2706	34	2009	H
2715_1524_S668_	2013	D	22.05.2013	S668	14.9	27	Fam-09_2007_S668	668	2707	9	2007	W
2715_1524_S669_	2013	D	22.05.2013	S669	12.8	16.5	Fam-59_2009_S669	669	2708	59	2009	W
2715_1524_S670_	2013	D	22.05.2013	S670	15.5	32	Fam-09_2007_S670	670	2709	9	2007	W
2715_1524_S671_	2013	D	22.05.2013	S671	13.4	21	Fam-31_2009_S671	671	2710	31	2009	F
2715_1524_S672_	2013	D	22.05.2013	S672	13.2	18.5	Fam-43_2009_S672	672	2711	43	2009	F
2715_1524_S673_	2013	D	22.05.2013	S673	14.6	25.5	Fam-42_2009_S673	673	2712	42	2009	H
2715_1524_S674_	2013	D	22.05.2013	S674	14.5	23.5	Fam-76_2010_S674	674	2713	76	2010	H

2715_1524_S675_	2013	D	22.05.2013	S675	14.4	25	Fam-43_2009_S675	675	2714	43	2009	F
2715_1524_S676_	2013	D	22.05.2013	S676	14.3	24	Fam-45_2009_S676	676	2715	45	2009	F
2715_1524_S677_	2013	D	22.05.2013	S677	12.5	16	Fam-59_2009_S677	677	2716	59	2009	W
2715_1524_S678_	2013	D	22.05.2013	S678	13.5	20	Fam-48_2009_S678	678	2717	48	2009	H
2715_1524_S679_	2013	D	22.05.2013	S679	13.6	19.5	Fam-49_2009_S679	679	2718	49	2009	F
2715_1524_S680_	2013	D	22.05.2013	S680	14	21.5	Fam-39_2009_S680	680	2719	39	2009	F
2715_1524_S681_	2013	D	22.05.2013	S681	14.5	23.5	Fam-52_2009_S681	681	2720	52	2009	W
2715_1524_S682_	2013	D	22.05.2013	S682	12.6	18	Fam-58_2009_S682	682	2721	58	2009	W
2715_1524_S683_	2013	D	22.05.2013	S683	12.6	17	Fam-44_2009_S683	683	2722	44	2009	H
2715_1524_S684_	2013	D	22.05.2013	S684	13.2	21	Fam-41_2009_S684	684	2723	41	2009	F
2715_1524_S685_	2013	D	22.05.2013	S685	13.5	22	Fam-47_2009_S685	685	2724	47	2009	F
2715_1524_S686_	2013	D	22.05.2013	S686	15	28.5	Fam-05_2007_S686	686	2725	5	2007	W
2715_1524_S687_	2013	D	22.05.2013	S687	14	25	Fam-42_2009_S687	687	2726	42	2009	H
2715_1524_S688_	2013	D	22.05.2013	S688	12.6	17.5	Fam-43_2009_S688	688	2727	43	2009	F

2715_1524_S689_	2013	D	22.05.2013	S689	12.6	16	Fam-49_2009_S689	689	2728	49	2009	F
2715_1524_S690_	2013	D	22.05.2013	S690	13.8	22.5	Fam-44_2009_S690	690	2729	44	2009	H
2715_1524_S691_	2013	D	22.05.2013	S691	13.5	20	Fam-50_2009_S691	691	2730	50	2009	H
2715_1524_S692_	2013	D	22.05.2013	S692	12.9	18	Fam-56_2009_S692	692	2731	56	2009	W
2715_1524_S693_	2013	D	22.05.2013	S693	13.4	19.5	Fam-48_2009_S693	693	2732	48	2009	H
2715_1524_S694_	2013	D	22.05.2013	S694	15	29	Fam-55_2009_S694	694	2733	55	2009	W
2715_1524_S695_	2013	D	22.05.2013	S695	11.8	14.5	Fam-44_2009_S695	695	2734	44	2009	H
2715_1524_S696_	2013	D	22.05.2013	S696	12.6	16.5	Fam-59_2009_S696	696	2735	59	2009	W
2715_1524_S697_	2013	D	22.05.2013	S697	12.4	18	Fam-45_2009_S697	697	2736	45	2009	F
2715_1524_S698_	2013	D	22.05.2013	S698	15.6	32	Fam-09_2007_S698	698	2737	9	2007	W
2715_1524_S699_	2013	D	22.05.2013	S699	13.4	18.5	Fam-51_2009_S699	699	2738	51	2009	W
2715_1524_S700_	2013	D	22.05.2013	S700	14.7	26.5	Fam-52_2009_S700	700	2739	52	2009	W
2715_1524_S701_	2013	D	22.05.2013	S701	13.2	22.5	Fam-55_2009_S701	701	2740	55	2009	W
2715_1524_S702_	2013	D	22.05.2013	S702	13.8	27	Fam-40_2009_S702	702	2741	40	2009	H

2715_1524_S704_	2013	D	22.05.2013	S704	12.9	19	Fam-42_2009_ S704	704	2742	42	2009	H
2715_1524_S705_	2013	D	22.05.2013	S705	13.2	19	Fam-51_2009_ S705	705	2743	51	2009	W
2715_1524_S706_	2013	D	22.05.2013	S706	13.6	20.5	Fam-31_2009_ S706	706	2744	31	2009	F
2715_1524_S707_	2013	D	22.05.2013	S707	13.2	20	Fam-42_2009_ S707	707	2745	42	2009	H
2715_1524_S708_	2013	D	22.05.2013	S708	12.2	14	Fam-51_2009_ S708	708	2746	51	2009	W
2715_1524_S709_	2013	D	22.05.2013	S709	13.8	21	Fam-50_2009_ S709	709	2747	50	2009	H
2715_1524_S710_	2013	D	22.05.2013	S710	13.5	20.5	Fam-40_2009_ S710	710	2748	40	2009	H
2715_1524_S711_	2013	D	22.05.2013	S711	11.3	12	Fam-44_2009_ S711	711	2749	44	2009	H
2715_1524_S712_	2013	D	22.05.2013	S712	13	19.5	Fam-47_2009_ S712	712	2750	47	2009	F
2715_1524_S713_	2013	D	22.05.2013	S713	14.8	25.5	Fam-59_2009_ S713	713	2751	59	2009	W
2715_1524_S714_	2013	D	22.05.2013	S714	14.5	26.5	Fam-56_2009_ S714	714	2752	56	2009	W
2715_1524_S715_	2013	D	22.05.2013	S715	14.3	24	Fam-55_2009_ S715	715	2753	55	2009	W
2715_1524_S716_	2013	D	22.05.2013	S716	13	18	Fam-34_2009_ S716	716	2754	34	2009	H
2715_1524_S717_	2013	D	22.05.2013	S717	12.2	15.5	Fam-56_2009_ S717	717	2755	56	2009	W

2715_1524_S718_	2013	D	22.05.2013	S718	15.1	29.5	Fam-03_2007_S718	718	2756	3	2007	W
2715_1524_S719_	2013	D	22.05.2013	S719	13.2	19	Fam-55_2009_S719	719	2757	55	2009	W
2716_1524_S721_	2013	D	22.05.2013	S721	13.2	19.5	Fam-38_2009_S721	721	2758	38	2009	H
2716_1524_S722_	2013	D	22.05.2013	S722	14	20.5	Fam-53_2009_S722	722	2759	53	2009	W
2716_1524_S723_	2013	D	22.05.2013	S723	12.2	14	Fam-51_2009_S723	723	2760	51	2009	W
2716_1524_S725_	2013	D	22.05.2013	S725	13.5	19	Fam-52_2009_S725	725	2761	52	2009	W
2716_1524_S726_	2013	D	22.05.2013	S726	11.9	13.5	Fam-59_2009_S726	726	2762	59	2009	W
2716_1524_S727_	2013	D	22.05.2013	S727	13.2	18	Fam-59_2009_S727	727	2763	59	2009	W
2716_1524_S728_	2013	D	22.05.2013	S728	13.2	19	Fam-56_2009_S728	728	2764	56	2009	W
2716_1524_S729_	2013	D	22.05.2013	S729	12.3	16	Fam-56_2009_S729	729	2765	56	2009	W
2716_1524_S730_	2013	D	22.05.2013	S730	12.3	16	Fam-54_2009_S730	730	2766	54	2009	W
2716_1524_S731_	2013	D	22.05.2013	S731	13.2	19	Fam-41_2009_S731	731	2767	41	2009	F
2716_1524_S732_	2013	D	22.05.2013	S732	13.5	22	Fam-45_2009_S732	732	2768	45	2009	F
2716_1524_S733_	2013	D	22.05.2013	S733	12.7	16	Fam-36_2009_S733	733	2769	36	2009	H

2716_1524_S734_	2013	D	22.05.2013	S734	13	18	Fam-51_2009_S734	734	2770	51	2009	W
2716_1524_S735_	2013	D	22.05.2013	S735	13.5	21.5	Fam-44_2009_S735	735	2771	44	2009	H
2716_1524_S736_	2013	D	22.05.2013	S736	16.2	35	Fam-03_2007_S736	736	2772	3	2007	W
2716_1524_S737_	2013	D	22.05.2013	S737	12.5	16.5	Fam-52_2009_S737	737	2773	52	2009	W
2716_1524_S739_	2013	D	22.05.2013	S739	12.5	16	Fam-44_2009_S739	739	2774	44	2009	H
2716_1524_S740_	2013	D	22.05.2013	S740	11.6	13.5	Fam-85_2010_S740	740	2775	85	2010	W
2716_1524_S741_	2013	D	22.05.2013	S741	13.5	18.5	Fam-40_2009_S741	741	2776	40	2009	H
2716_1524_S742_	2013	D	22.05.2013	S742	14.5	25	Fam-41_2009_S742	742	2777	41	2009	F
2716_1524_S743_	2013	D	22.05.2013	S743	15.1	28	Fam-03_2007_S743	743	2778	3	2007	W
2716_1524_S744_	2013	D	22.05.2013	S744	11.8	14	Fam-53_2009_S744	744	2779	53	2009	W
2716_1524_S745_	2013	D	22.05.2013	S745	12.6	16	Fam-54_2009_S745	745	2780	54	2009	W
2716_1524_S746_	2013	D	22.05.2013	S746	12.5	15.5	Fam-59_2009_S746	746	2781	59	2009	W
2716_1524_S747_	2013	D	22.05.2013	S747	12.7	16.5	Fam-58_2009_S747	747	2782	58	2009	W
2716_1524_S748_	2013	D	22.05.2013	S748	13	19.5	Fam-42_2009_S748	748	2783	42	2009	H

2716_1524_S749_	2013	D	22.05.2013	S749	14	20	Fam-39_2009_	S749	749	2784	39	2009	F
2716_1524_S750_	2013	D	22.05.2013	S750	13.8	20	Fam-34_2009_	S750	750	2785	34	2009	H
2716_1524_S751_	2013	D	22.05.2013	S751	14	22	Fam-39_2009_	S751	751	2786	39	2009	F
2716_1524_S752_	2013	D	22.05.2013	S752	14.2	23	Fam-59_2009_	S752	752	2787	59	2009	W
2716_1525_S753_	2013	D	22.05.2013	S753	13.4	19	Fam-51_2009_	S753	753	2788	51	2009	W
2716_1525_S754_	2013	D	22.05.2013	S754	12.7	16.5	Fam-60_2009_	S754	754	2789	60	2009	W
2716_1525_S755_	2013	D	22.05.2013	S755	13.2	18.5	Fam-44_2009_	S755	755	2790	44	2009	H
2716_1525_S756_	2013	D	22.05.2013	S756	15	26.5	Fam-43_2009_	S756	756	2791	43	2009	F
2716_1525_S757_	2013	D	22.05.2013	S757	15.2	29.5	Fam-05_2007_	S757	757	2792	5	2007	W
2716_1525_S758_	2013	D	22.05.2013	S758	14.6	25	Fam-34_2009_	S758	758	2793	34	2009	H
2716_1525_S759_	2013	D	22.05.2013	S759	13	17.5	Fam-54_2009_	S759	759	2794	54	2009	W
2716_1525_S760_	2013	D	22.05.2013	S760	13.4	18.5	Fam-75_2010_	S760	760	2795	75	2010	F
2716_1525_S761_	2013	D	22.05.2013	S761	12.4	15.5	Fam-52_2009_	S761	761	2796	52	2009	W
2716_1525_S762_	2013	D	22.05.2013	S762	13.6	19.5	Fam-57_2009_	S762	762	2797	57	2009	W

2716_1525_S763_	2013	D	22.05.2013	S763	13.7	20	Fam-52_2009_	S763	763	2798	52	2009	W
2716_1525_S764_	2013	D	22.05.2013	S764	11.8	13	Fam-59_2009_	S764	764	2799	59	2009	W
2716_1525_S765_	2013	D	22.05.2013	S765	13	18	Fam-55_2009_	S765	765	2800	55	2009	W
2716_1525_S766_	2013	D	22.05.2013	S766	13	19	Fam-42_2009_	S766	766	2801	42	2009	H
2716_1525_S767_	2013	D	22.05.2013	S767	12.6	16.5	Fam-31_2009_	S767	767	2802	31	2009	F
2716_1525_S768_	2013	D	22.05.2013	S768	12.8	15	Fam-59_2009_	S768	768	2803	59	2009	W
2716_1525_S769_	2013	D	22.05.2013	S769	14.8	25.5	Fam-34_2009_	S769	769	2804	34	2009	H
2716_1525_S770_	2013	D	22.05.2013	S770	16.2	32	Fam-28_2007_	S770	770	2805	28	2007	H
2716_1525_S771_	2013	D	22.05.2013	S771	13.7	21	Fam-56_2009_	S771	771	2806	56	2009	W
2716_1525_S772_	2013	D	22.05.2013	S772	13.5	19	Fam-43_2009_	S772	772	2807	43	2009	F
2716_1525_S773_	2013	D	22.05.2013	S773	12.6	16	Fam-59_2009_	S773	773	2808	59	2009	W
2716_1525_S774_	2013	D	22.05.2013	S774	13.1	18	Fam-06_2007_	S774	774	2809	6	2007	W
2716_1525_S775_	2013	D	22.05.2013	S775	12.5	14.5	Fam-53_2009_	S775	775	2810	53	2009	W
2716_1525_S776_	2013	D	22.05.2013	S776	11.4	12.5	Fam-44_2009_	S776	776	2811	44	2009	H

2716_1525_S777_	2013	D	22.05.2013	S777	13	18	Fam-55_2009_	S777	777	2812	55	2009	W
2716_1525_S778_	2013	D	22.05.2013	S778	13.1	19	Fam-32_2009_	S778	778	2813	32	2009	H
2716_1525_S779_	2013	D	22.05.2013	S779	14	22	Fam-56_2009_	S779	779	2814	56	2009	W
2716_1525_S780_	2013	D	22.05.2013	S780	14.5	22.5	Fam-44_2009_	S780	780	2815	44	2009	H
2716_1525_S781_	2013	D	22.05.2013	S781	13.1	18	Fam-56_2009_	S781	781	2816	56	2009	W
2716_1525_S782_	2013	D	22.05.2013	S782	12.6	17	Fam-59_2009_	S782	782	2817	59	2009	W
2716_1525_S783_	2013	D	22.05.2013	S783	13.1	17	Fam-34_2009_	S783	783	2818	34	2009	H
2716_1525_S784_	2013	D	27.05.2013	S784	14.5	24.5	Fam-34_2009_	S784	784	2819	34	2009	H
2716_1525_S785_	2013	D	27.05.2013	S785	12.1	17.5	Fam-42_2009_	S785	785	2820	42	2009	H
2716_1525_S786_	2013	D	27.05.2013	S786	13.9	20.5	Fam-60_2009_	S786	786	2821	60	2009	W
2716_1525_S787_	2013	D	27.05.2013	S787	12.6	16.5	Fam-56_2009_	S787	787	2822	56	2009	W
2716_1525_S788_	2013	D	27.05.2013	S788	12.1	14	Fam-59_2009_	S788	788	2823	59	2009	W
2716_1525_S789_	2013	D	27.05.2013	S789	13.6	21	Fam-34_2009_	S789	789	2824	34	2009	H
2716_1525_S790_	2013	D	27.05.2013	S790	13.2	19.5	Fam-42_2009_	S790	790	2825	42	2009	H

2716_1525_S791_	2013	D	27.05.2013	S791	15.8	33	Fam-03_2007_	S791	791	2826	3	2007	W
2716_1525_S792_	2013	D	27.05.2013	S792	12.3	15	Fam-59_2009_	S792	792	2827	59	2009	W
2716_1525_S793_	2013	D	27.05.2013	S793	14.6	26	Fam-34_2009_	S793	793	2828	34	2009	H
2716_1525_S794_	2013	D	27.05.2013	S794	12.7	16.5	Fam-59_2009_	S794	794	2829	59	2009	W
2716_1525_S795_	2013	D	27.05.2013	S795	13.6	21	Fam-60_2009_	S795	795	2830	60	2009	W
2716_1525_S796_	2013	D	27.05.2013	S796	13.8	22	Fam-54_2009_	S796	796	2831	54	2009	W
2716_1525_S797_	2013	D	27.05.2013	S797	12.6	17	Fam-60_2009_	S797	797	2832	60	2009	W
2716_1525_S798_	2013	D	27.05.2013	S798	13.4	20.5	Fam-05_2007_	S798	798	2833	5	2007	W
2716_1525_S799_	2013	D	27.05.2013	S799	13.8	21	Fam-59_2009_	S799	799	2834	59	2009	W
2716_1525_S800_	2013	D	27.05.2013	S800	13	17	Fam-68_2010_	S800	800	2835	68	2010	H
2717_1525_S801_	2013	D	27.05.2013	S801	15.2	28	Fam-57_2009_	S801	801	2836	57	2009	W
2717_1525_S802_	2013	D	27.05.2013	S802	12.4	18.5	Fam-44_2009_	S802	802	2837	44	2009	H
2717_1525_S803_	2013	D	27.05.2013	S803	13	18	Fam-05_2007_	S803	803	2838	5	2007	W
2717_1525_S804_	2013	D	27.05.2013	S804	12.6	17	Fam-52_2009_	S804	804	2839	52	2009	W

2717_1525_S805_	2013	D	27.05.2013	S805	13.4	19	Fam-57_2009_	S805	805	2840	57	2009	W
2717_1525_S806_	2013	D	27.05.2013	S806	12.2	15.5	Fam-59_2009_	S806	806	2841	59	2009	W
2717_1525_S807_	2013	D	27.05.2013	S807	14.6	24	Fam-32_2009_	S807	807	2842	32	2009	H
2717_1525_S808_	2013	D	27.05.2013	S808	13.1	19.5	Fam-42_2009_	S808	808	2843	42	2009	H
2717_1525_S809_	2013	D	27.05.2013	S809	14.3	24.5	Fam-34_2009_	S809	809	2844	34	2009	H
2717_1525_S810_	2013	D	27.05.2013	S810	12.9	17.5	Fam-56_2009_	S810	810	2845	56	2009	W
2717_1525_S811_	2013	D	27.05.2013	S811	13.5	19.5	Fam-60_2009_	S811	811	2846	60	2009	W
2717_1525_S812_	2013	D	27.05.2013	S812	14.1	21	Fam-52_2009_	S812	812	2847	52	2009	W
2717_1525_S813_	2013	D	27.05.2013	S813	15.6	31	Fam-05_2007_	S813	813	2848	5	2007	W
2717_1525_S814_	2013	D	27.05.2013	S814	13.6	21	Fam-04_2007_	S814	814	2849	4	2007	W
2717_1525_S815_	2013	D	27.05.2013	S815	14.5	26	Fam-42_2009_	S815	815	2850	42	2009	H
2717_1525_S816_	2013	D	27.05.2013	S816	13	17.5	Fam-59_2009_	S816	816	2851	59	2009	W
2717_1525_S817_	2013	D	27.05.2013	S817	13.2	19	Fam-90_2010_	S817	817	2852	90	2010	W
2717_1525_S818_	2013	D	27.05.2013	S818	13	17.5	Fam-04_2007_	S818	818	2853	4	2007	W

2717_1525_S819_	2013	D	27.05.2013	S819	12.5	15	Fam-59_2009_ S819	819	2854	59	2009 W
2717_1525_S820_	2013	D	27.05.2013	S820	13.4	20.5	Fam-59_2009_ S820	820	2855	59	2009 W
2717_1525_S821_	2013	D	27.05.2013	S821	14.3	24.5	Fam-34_2009_ S821	821	2856	34	2009 H
2717_1525_S822_	2013	D	27.05.2013	S822	15	27.5	Fam-05_2007_ S822	822	2857	5	2007 W
2717_1525_S823_	2013	D	27.05.2013	S823	13.4	19.5	Fam-54_2009_ S823	823	2858	54	2009 W
2717_1525_S824_	2013	D	27.05.2013	S824	14.5	24.5	Fam-51_2009_ S824	824	2859	51	2009 W
2717_1525_S825_	2013	D	27.05.2013	S825	14.3	24.5	Fam-42_2009_ S825	825	2860	42	2009 H
2717_1525_S826_	2013	D	27.05.2013	S826	13.3	19	Fam-60_2009_ S826	826	2861	60	2009 W
2717_1525_S827_	2013	D	27.05.2013	S827	12.7	17	Fam-52_2009_ S827	827	2862	52	2009 W
2717_1525_S828_	2013	D	27.05.2013	S828	14.1	21.5	Fam-09_2007_ S828	828	2863	9	2007 W
2717_1525_S829_	2013	D	27.05.2013	S829	13.7	20.5	Fam-89_2010_ S829	829	2864	89	2010 W
2717_1525_S830_	2013	D	27.05.2013	S830	13.9	20.5	Fam-34_2009_ S830	830	2865	34	2009 H
2717_1525_S831_	2013	D	27.05.2013	S831	14.7	28	Fam-13_2007_ S831	831	2866	13	2007 F
2717_1525_S832_	2013	D	27.05.2013	S832	13.9	21.5	Fam-48_2009_ S832	832	2867	48	2009 H

2717_1525_S833_	2013	D	27.05.2013	S833	11.9	13	Fam-59_2009_	S833	833	2868	59	2009	W
2717_1525_S834_	2013	D	27.05.2013	S834	13.3	19	Fam-59_2009_	S834	834	2869	59	2009	W
2717_1525_S835_	2013	D	31.05.2013	S835	12.5	15.5	Fam-59_2009_	S835	835	2870	59	2009	W
2717_1525_S836_	2013	D	31.05.2013	S836	14.8	24	Fam-34_2009_	S836	836	2871	34	2009	H
2717_1525_S837_	2013	D	31.05.2013	S837	14.5	24	Fam-03_2007_	S837	837	2872	3	2007	W
2717_1525_S838_	2013	D	31.05.2013	S838	13.8	20.5	Fam-60_2009_	S838	838	2873	60	2009	W
2717_1525_S840_	2013	D	31.05.2013	S840	14.3	23.5	Fam-60_2009_	S840	840	2874	60	2009	W
2717_1525_S841_	2013	D	31.05.2013	S841	13.5	19	Fam-32_2009_	S841	841	2875	32	2009	H
2717_1525_S842_	2013	D	31.05.2013	S842	12.7	16.5	Fam-60_2009_	S842	842	2876	60	2009	W
2717_1525_S843_	2013	D	31.05.2013	S843	13.8	20.5	Fam-32_2009_	S843	843	2877	32	2009	H
2717_1525_S844_	2013	D	31.05.2013	S844	12.9	17	Fam-54_2009_	S844	844	2878	54	2009	W
2717_1525_S845_	2013	D	31.05.2013	S845	15.7	30.5	Fam-32_2009_	S845	845	2879	32	2009	H
2717_1525_S846_	2013	D	31.05.2013	S846	14	22	Fam-60_2009_	S846	846	2880	60	2009	W
2717_1526_S847_	2013	D	31.05.2013	S847	13.7	21	Fam-25_2007_	S847	847	2881	25	2007	F

2717_1526_S848_	2013	D	31.05.2013	S848	13.9	21	Fam-44_2009_	S848	848	2882	44	2009	H
2717_1526_S849_	2013	D	31.05.2013	S849	14.6	23.5	Fam-05_2007_	S849	849	2883	5	2007	W
2717_1526_S850_	2013	D	31.05.2013	S850	14.7	25	Fam-59_2009_	S850	850	2884	59	2009	W
2717_1526_S851_	2013	D	31.05.2013	S851	13.7	20	Fam-60_2009_	S851	851	2885	60	2009	W
2717_1526_S852_	2013	D	31.05.2013	S852	14.2	22	Fam-55_2009_	S852	852	2886	55	2009	W
2717_1526_S854_	2013	D	31.05.2013	S854	13	17.5	Fam-42_2009_	S854	854	2887	42	2009	H
2717_1526_S855_	2013	D	31.05.2013	S855	14.1	23	Fam-32_2009_	S855	855	2888	32	2009	H
2717_1526_S856_	2013	D	03.06.2013	S856	14	22	Fam-34_2009_	S856	856	2889	34	2009	H
2717_1526_S857_	2013	D	03.06.2013	S857	14.3	23	Fam-52_2009_	S857	857	2890	52	2009	W
2717_1526_S858_	2013	D	03.06.2013	S858	14.5	25	Fam-10_2007_	S858	858	2891	10	2007	W
2717_1526_S859_	2013	D	03.06.2013	S859	12.3	15	Fam-56_2009_	S859	859	2892	56	2009	W
2717_1526_S860_	2013	D	03.06.2013	S860	14.2	24	Fam-34_2009_	S860	860	2893	34	2009	H
2717_1526_S861_	2013	D	03.06.2013	S861	14.6	25	Fam-51_2009_	S861	861	2894	51	2009	W
2717_1526_S862_	2013	D	03.06.2013	S862	14.2	22	Fam-60_2009_	S862	862	2895	60	2009	W

2717_1526_S863_	2013	D	03.06.2013	S863	13.9	21	Fam-34_2009_S863	863	2896	34	2009	H
2717_1526_S864_	2013	D	03.06.2013	S864	15.9	31	Fam-03_2007_S864	864	2897	3	2007	W
2717_1526_S865_	2013	D	03.06.2013	S865	16.1	32.5	Fam-55_2009_S865	865	2898	55	2009	W
2717_1526_S866_	2013	D	03.06.2013	S866	14.1	23	Fam-32_2009_S866	866	2899	32	2009	H
2717_1526_S867_	2013	D	03.06.2013	S867	14.2	22.5	Fam-34_2009_S867	867	2900	34	2009	H
2717_1526_S868_	2013	D	03.06.2013	S868	14.5	26.5	Fam-07_2007_S868	868	2901	7	2007	W
2717_1526_S869_	2013	D	03.06.2013	S869	12.9	17	Fam-60_2009_S869	869	2902	60	2009	W
2717_1526_S870_	2013	D	03.06.2013	S870	14	23.5	Fam-42_2009_S870	870	2903	42	2009	H
2717_1526_S871_	2013	D	03.06.2013	S871	13.2	18	Fam-60_2009_S871	871	2904	60	2009	W
2717_1526_S872_	2013	D	03.06.2013	S872	13.1	18.5	Fam-44_2009_S872	872	2905	44	2009	H
2717_1526_S873_	2013	D	03.06.2013	S873	14.1	23.5	Fam-05_2007_S873	873	2906	5	2007	W
2717_1526_S874_	2013	D	03.06.2013	S874	14.3	22.5	Fam-40_2009_S874	874	2907	40	2009	H
2717_1526_S875_	2013	D	03.06.2013	S875	13.2	18	Fam-60_2009_S875	875	2908	60	2009	W
2717_1526_S876_	2013	D	03.06.2013	S876	15.3	30	Fam-21_2007_S876	876	2909	21	2007	F

2717_1526_S877_	2013	D	03.06.2013	S877	14.3	23	Fam-06_2007_S877	877	2910	6	2007	W
2717_1526_S878_	2013	D	03.06.2013	S878	12.5	16.5	Fam-59_2009_S878	878	2911	59	2009	W
2717_1526_S879_	2013	D	03.06.2013	S879	13.5	20	Fam-52_2009_S879	879	2912	52	2009	W
2717_1526_S880_	2013	D	03.06.2013	S880	14.9	25	Fam-32_2009_S880	880	2913	32	2009	H
2718_1526_S881_	2013	D	03.06.2013	S881	14.5	25	Fam-40_2009_S881	881	2914	40	2009	H
2718_1526_S882_	2013	D	03.06.2013	S882	12.5	16	Fam-59_2009_S882	882	2915	59	2009	W
2718_1526_S883_	2013	D	03.06.2013	S883	13	17	Fam-34_2009_S883	883	2916	34	2009	H
2718_1526_S884_	2013	D	03.06.2013	S884	14.9	26.5	Fam-04_2007_S884	884	2917	4	2007	W
2718_1526_S885_	2013	D	03.06.2013	S885	15.9	30	Fam-32_2009_S885	885	2918	32	2009	H
2718_1526_S886_	2013	D	03.06.2013	S886	13.5	20	Fam-44_2009_S886	886	2919	44	2009	H
2718_1526_S887_	2013	D	03.06.2013	S887	13.2	19.5	Fam-42_2009_S887	887	2920	42	2009	H
2718_1526_S888_	2013	D	03.06.2013	S888	16.1	36	Fam-04_2007_S888	888	2921	4	2007	W
2718_1526_S889_	2013	D	03.06.2013	S889	12.9	18	Fam-41_2009_S889	889	2922	41	2009	F
2718_1526_S890_	2013	D	03.06.2013	S890	14.6	26	Fam-30_2007_S890	890	2923	30	2007	H

2718_1526_S891_	2013	D	03.06.2013	S891	12.8	16	Fam-52_2009_ S891	891	2924	52	2009	W
2718_1526_S892_	2013	D	03.06.2013	S892	13.3	18	Fam-59_2009_ S892	892	2925	59	2009	W
2718_1526_S893_	2013	D	03.06.2013	S893	14	22	Fam-55_2009_ S893	893	2926	55	2009	W
2718_1526_S894_	2013	D	03.06.2013	S894	13	18	Fam-32_2009_ S894	894	2927	32	2009	H
2718_1526_S895_	2013	D	03.06.2013	S895	12.9	17	Fam-54_2009_ S895	895	2928	54	2009	W
2718_1526_S896_	2013	D	03.06.2013	S896	13.5	20	Fam-34_2009_ S896	896	2929	34	2009	H
2718_1526_S897_	2013	D	03.06.2013	S897	13.6	21	Fam-40_2009_ S897	897	2930	40	2009	H
2718_1526_S898_	2013	D	03.06.2013	S898	16.4	35	Fam-04_2007_ S898	898	2931	4	2007	W
2718_1526_S899_	2013	D	03.06.2013	S899	14.9	26.5	Fam-03_2007_ S899	899	2932	3	2007	W
2718_1526_S900_	2013	D	03.06.2013	S900	14	22	Fam-34_2009_ S900	900	2933	34	2009	H
2718_1526_S901_	2013	D	03.06.2013	S901	15	30	Fam-42_2009_ S901	901	2934	42	2009	H
2718_1526_S902_	2013	D	03.06.2013	S902	13.8	22	Fam-32_2009_ S902	902	2935	32	2009	H
2718_1526_S903_	2013	D	03.06.2013	S903	14	22	Fam-55_2009_ S903	903	2936	55	2009	W
2718_1526_S904_	2013	D	03.06.2013	S904	15	27	Fam-06_2007_ S904	904	2937	6	2007	W

2718_1526_S905_	2013	D	03.06.2013	S905	14.6	26.5	Fam-05_2007_S905	905	2938	5	2007	W
2718_1526_S907_	2013	D	03.06.2013	S907	15	28	Fam-55_2009_S907	907	2939	55	2009	W
2718_1526_S908_	2013	D	03.06.2013	S908	12.6	16	Fam-34_2009_S908	908	2940	34	2009	H
2718_1526_S909_	2013	D	03.06.2013	S909	13.6	20.5	Fam-55_2009_S909	909	2941	55	2009	W
2718_1526_S910_	2013	D	03.06.2013	S910	14.5	24.5	Fam-03_2007_S910	910	2942	3	2007	W
2718_1526_S911_	2013	D	03.06.2013	S911	13	17	Fam-03_2007_S911	911	2943	3	2007	W
2718_1526_S912_	2013	D	03.06.2013	S912	13.6	20	Fam-60_2009_S912	912	2944	60	2009	W
2718_1526_S913_	2013	D	03.06.2013	S913	14	21.5	Fam-34_2009_S913	913	2945	34	2009	H
2718_1526_S914_	2013	D	03.06.2013	S914	15.9	32	Fam-03_2007_S914	914	2946	3	2007	W
2718_1526_S915_	2013	D	11.06.2013	S915	14.4	22.5	Fam-52_2009_S915	915	2947	52	2009	W
2718_1526_S916_	2013	D	11.06.2013	S916	13.8	20	Fam-32_2009_S916	916	2948	32	2009	H
2718_1526_S917_	2013	D	11.06.2013	S917	14.6	24	Fam-50_2009_S917	917	2949	50	2009	H
2718_1526_S918_	2013	D	18.06.2013	S918	16.2	13.5	Fam-34_2009_S918	918	2950	34	2009	H
2718_1526_S919_	2013	D	18.06.2013	S919	15.9	30	Fam-60_2009_S919	919	2951	60	2009	W

2718_1526_S920_	2013	D	24.06.2013	S920	15	24.5	Fam-55_2009_S920	920	2952	55	2009 W
2718_1526_S921_	2013	D	24.06.2013	S921	14.8	27.5	Fam-45_2009_S921	921	2953	45	2009 F
2718_1526_S922_	2013	D	24.06.2013	S922	14.6	23	Fam-52_2009_S922	922	2954	52	2009 W
2718_1526_S923_	2013	D	24.06.2013	S923	12.5	15.5	Fam-59_2009_S923	923	2955	59	2009 W
2718_1526_S924_	2013	D	24.06.2013	S924	13.7	20	Fam-60_2009_S924	924	2956	60	2009 W
2718_1526_S925_	2013	D	24.06.2013	S925	17	38.5	Fam-55_2009_S925	925	2957	55	2009 W
2718_1526_S926_	2013	D	24.06.2013	S926	13.5	19	Fam-59_2009_S926	926	2958	59	2009 W
2718_1526_S927_	2013	D	24.06.2013	S927	14.5	23	Fam-34_2009_S927	927	2959	34	2009 H
2718_1526_S928_	2013	D	24.06.2013	S928	15.5	25.5	Fam-55_2009_S928	928	2960	55	2009 W
2718_1526_S929_	2013	D	24.06.2013	S929	13.8	20.5	Fam-60_2009_S929	929	2961	60	2009 W
2718_1526_S930_	2013	D	24.06.2013	S930	14	22	Fam-59_2009_S930	930	2962	59	2009 W
2718_1526_S931_	2013	D	24.06.2013	S931	15.8	30	Fam-55_2009_S931	931	2963	55	2009 W
2718_1526_S932_	2013	D	24.06.2013	S932	14.6	24.5	Fam-55_2009_S932	932	2964	55	2009 W
2718_1526_S933_	2013	D	24.06.2013	S933	15.6	26.5	Fam-32_2009_S933	933	2965	32	2009 H

2900_1695_S1_A(	2014	D	31.03.2014	S1	15.8	30	Fam-35_2009_	S1	1	2966	35	2009	F
2900_1695_S2_B(	2014	D	31.03.2014	S2	13.4	20.5	Fam-36_2009_	S2	2	2967	36	2009	H
2900_1695_S3_C(	2014	D	31.03.2014	S3	12.5	15.5	Fam-35_2009_	S3	3	2968	35	2009	F
2900_1695_S4_D(	2014	D	31.03.2014	S4	13.4	18.5	Fam-51_2009_	S4	4	2969	51	2009	W
2900_1695_S5_E(	2014	D	31.03.2014	S5	12.5	17	Fam-85_2010_	S5	5	2970	85	2010	W
2900_1695_S6_F(	2014	D	31.03.2014	S6	14	21.5	Fam-83_2010_	S6	6	2971	83	2010	W
2900_1695_S7_G(	2014	D	01.04.2014	S7	13.5	20.5	Fam-61_1_201	S7	7	2972	61	2010	F
2900_1695_S8_H(	2014	D	01.04.2014	S8	12.2	14	Fam-36_2009_	S8	8	2973	36	2009	H
2900_1695_S9_A(	2014	D	01.04.2014	S9	13.7	20	Fam-31_2009_	S9	9	2974	31	2009	F
2900_1695_S10_E	2014	D	01.04.2014	S10	12.6	15.5	Fam-70_2010_	S10	10	2975	70	2010	H
2900_1695_S11_(	2014	D	01.04.2014	S11	15	28.5	Fam-31_2009_	S11	11	2976	31	2009	F
2900_1695_S12_D	2014	D	01.04.2014	S12	13.2	19	Fam-58_2009_	S12	12	2977	58	2009	W
2900_1695_S13_E	2014	D	04.04.2014	S13	13	18.5	Fam-31_2009_	S13	13	2978	31	2009	F
2900_1695_S14_F	2014	D	04.04.2014	S14	15.5	30	Fam-37_2009_	S14	14	2979	37	2009	F

2900_1695_S15_G	2014	D	04.04.2014	S15	13.5	22	Fam-80_2010_	S15	15	2980	80	2010 H
2900_1695_S16_H	2014	D	04.04.2014	S16	14	25.5	Fam-38_2009_	S16	16	2981	38	2009 H
2900_1695_S17_A	2014	T	04.04.2014	S17	13.4	17.5	Family-34_200	S17	17	2982	34	2009 H
2900_1695_S18_B	2014	D	04.04.2014	S18	14.5	23.5	Fam-45_2009_	S18	18	2983	45	2009 F
2900_1695_S19_C	2014	D	04.04.2014	S19	12.6	16.5	Fam-35_2009_	S19	19	2984	35	2009 F
2900_1695_S20_E	2014	D	04.04.2014	S20	14.8	24.5	Fam-37_2009_	S20	20	2985	37	2009 F
2900_1695_S21_F	2014	D	04.04.2014	S21	12.6	16	Fam-72_2010_	S21	21	2986	72	2010 H
2900_1695_S22_G	2014	D	04.04.2014	S22	13.7	21	Fam-31_2009_	S22	22	2987	31	2009 F
2900_1695_S23_H	2014	D	07.04.2014	S23	13.5	16.5	Fam-36_2009_	S23	23	2988	36	2009 H
2900_1695_S24_A	2014	D	07.04.2014	S24	13.5	21	Fam-35_2009_	S24	24	2989	35	2009 F
2900_1695_S25_B	2014	D	07.04.2014	S25	14.6	26	Fam-33_2009_	S25	25	2990	33	2009 F
2900_1695_S26_C	2014	D	07.04.2014	S26	14.3	23.5	Fam-71_2010_	S26	26	2991	71	2010 F
2900_1695_S27_D	2014	D	07.04.2014	S27	15.6	30	Fam-61_1_201	S27	27	2992	61	2010 F
2900_1695_S28_E	2014	D	07.04.2014	S28	14	25	Fam-58_2009_	S28	28	2993	58	2009 W

2900_1695_S29_F	2014	D	07.04.2014	S29	15.5	30.5	Fam-33_2009_	S29	29	2994	33	2009	F
2900_1695_S30_G	2014	D	07.04.2014	S30	14	21.5	Fam-72_2010_	S30	30	2995	72	2010	H
2900_1695_S31_H	2014	D	07.04.2014	S31	14	21.5	Fam-38_2009_	S31	31	2996	38	2009	H
2900_1695_S32_A	2014	T	07.04.2014	S32	13.6	19.5	Family-47_200	S32	32	2997	47	2009	F
2900_1695_S33_B	2014	D	07.04.2014	S33	13.6	20	Fam-36_2009_	S33	33	2998	36	2009	H
2900_1695_S34_C	2014	D	07.04.2014	S34	14.7	26	Fam-31_2009_	S34	34	2999	31	2009	F
2900_1695_S35_D	2014	D	07.04.2014	S35	14.4	23.5	Fam-37_2009_	S35	35	3000	37	2009	F
2900_1695_S36_E	2014	D	07.04.2014	S36	14.9	24.5	Fam-50_2009_	S36	36	3001	50	2009	H
2900_1695_S37_F	2014	D	07.04.2014	S37	14.4	24.5	Fam-61_1_201	S37	37	3002	61	2010	F
2900_1695_S38_G	2014	D	07.04.2014	S38	14.1	24	Fam-31_2009_	S38	38	3003	31	2009	F
2900_1695_S39_H	2014	D	07.04.2014	S39	12.9	15.5	Fam-43_2009_	S39	39	3004	43	2009	F
2900_1695_S40_A	2014	D	07.04.2014	S40	13.5	20.5	Fam-61_1_201	S40	40	3005	61	2010	F
2900_1695_S41_B	2014	D	07.04.2014	S41	13.3	22	Fam-31_2009_	S41	41	3006	31	2009	F
2900_1695_S42_C	2014	D	07.04.2014	S42	12.4	15.5	Fam-44_2009_	S42	42	3007	44	2009	H

2900_1695_S43_[illegible]	2014	D	07.04.2014	S43	14.6	25.5	Fam-43_2009_	S43	43	3008	43	2009	F
2900_1695_S44_[illegible]	2014	D	07.04.2014	S44	14.7	26	Fam-58_2009_	S44	44	3009	58	2009	W
2900_1695_S45_[illegible]	2014	D	07.04.2014	S45	14.9	27	Fam-33_2009_	S45	45	3010	33	2009	F
2900_1695_S46_[illegible]	2014	D	07.04.2014	S46	17	35	Fam-71_2010_	S46	46	3011	71	2010	F
2900_1695_S48_[illegible]	2014	D	07.04.2014	S48	12.6	16	Fam-50_2009_	S48	48	3012	50	2009	H
2900_1695_S49_[illegible]	2014	D	07.04.2014	S49	14	23	Fam-58_2009_	S49	49	3013	58	2009	W
2900_1695_S50_[illegible]	2014	D	07.04.2014	S50	12.6	16	Fam-59_2009_	S50	50	3014	59	2009	W
2900_1695_S51_[illegible]	2014	D	07.04.2014	S51	12.2	17	Fam-56_2009_	S51	51	3015	56	2009	W
2900_1695_S52_[illegible]	2014	D	07.04.2014	S52	13	18	Fam-54_2009_	S52	52	3016	54	2009	W
2900_1695_S53_[illegible]	2014	D	07.04.2014	S53	12.7	17.5	Fam-85_2010_	S53	53	3017	85	2010	W
2900_1695_S54_[illegible]	2014	D	07.04.2014	S54	13.9	22.5	Fam-36_2009_	S54	54	3018	36	2009	H
2900_1695_S55_[illegible]	2014	D	07.04.2014	S55	13.5	22	Fam-37_2009_	S55	55	3019	37	2009	F
2900_1695_S56_[illegible]	2014	D	07.04.2014	S56	12.2	15	Fam-44_2009_	S56	56	3020	44	2009	H
2900_1695_S58_[illegible]	2014	D	08.04.2014	S58	14.4	27	Fam-31_2009_	S58	58	3021	31	2009	F

2900_1695_S59_D	2014	D	08.04.2014	S59	13.2	20	Fam-45_2009_	S59	59	3022	45	2009	F
2900_1695_S60_E	2014	D	08.04.2014	S60	14.1	22	Fam-31_2009_	S60	60	3023	31	2009	F
2900_1695_S61_F	2014	D	08.04.2014	S61	13.8	21.5	Fam-31_2009_	S61	61	3024	31	2009	F
2900_1695_S62_G	2014	D	08.04.2014	S62	13.7	20.5	Fam-35_2009_	S62	62	3025	35	2009	F
2900_1695_S63_H	2014	D	08.04.2014	S63	12.5	16	Fam-36_2009_	S63	63	3026	36	2009	H
2900_1695_S64_A	2014	D	08.04.2014	S64	16	32	Fam-37_2009_	S64	64	3027	37	2009	F
2900_1695_S65_B	2014	D	08.04.2014	S65	15.5	30	Fam-48_2009_	S65	65	3028	48	2009	H
2900_1695_S66_C	2014	D	08.04.2014	S66	13.3	19	Fam-43_2009_	S66	66	3029	43	2009	F
2900_1695_S67_D	2014	D	08.04.2014	S67	12.4	14.5	Fam-36_2009_	S67	67	3030	36	2009	H
2900_1695_S68_E	2014	D	08.04.2014	S68	14.6	25.5	Fam-31_2009_	S68	68	3031	31	2009	F
2900_1695_S69_F	2014	D	08.04.2014	S69	12.9	19	Fam-45_2009_	S69	69	3032	45	2009	F
2900_1695_S70_G	2014	D	08.04.2014	S70	14.9	25.5	Fam-48_2009_	S70	70	3033	48	2009	H
2900_1695_S71_H	2014	D	08.04.2014	S71	13.5	21	Fam-35_2009_	S71	71	3034	35	2009	F
2900_1695_S72_A	2014	D	08.04.2014	S72	13.9	22	Fam-59_2009_	S72	72	3035	59	2009	W

2900_1695_S73_(	2014	D	08.04.2014	S73	15	30	Fam-79_2010_S73	73	3036	79	2010 F
2900_1695_S74_[	2014	D	08.04.2014	S74	15.2	31	Fam-37_2009_S74	74	3037	37	2009 F
2900_1695_S75_E	2014	D	08.04.2014	S75	14.3	24	Fam-47_2009_S75	75	3038	47	2009 F
2900_1695_S76_F	2014	D	08.04.2014	S76	14	22.5	Fam-35_2009_S76	76	3039	35	2009 F
2900_1695_S77_(	2014	D	08.04.2014	S77	13	18.5	Fam-36_2009_S77	77	3040	36	2009 H
2900_1695_S78_H	2014	D	08.04.2014	S78	13.5	20.5	Fam-79_2010_S78	78	3041	79	2010 F
2900_1695_S79_A	2014	D	08.04.2014	S79	13.8	21.5	Fam-48_2009_S79	79	3042	48	2009 H
2900_1695_S80_E	2014	D	08.04.2014	S80	12.4	15.5	Fam-59_2009_S80	80	3043	59	2009 W
2901_1695_S81_(	2014	D	08.04.2014	S81	15	28	Fam-36_2009_S81	81	3044	36	2009 H
2901_1695_S82_[	2014	D	08.04.2014	S82	14.5	24.5	Fam-38_2009_S82	82	3045	38	2009 H
2901_1695_S83_E	2014	D	08.04.2014	S83	13.6	20	Fam-47_2009_S83	83	3046	47	2009 F
2901_1695_S84_F	2014	D	08.04.2014	S84	14.2	25	Fam-31_2009_S84	84	3047	31	2009 F
2901_1695_S85_(	2014	D	08.04.2014	S85	13.8	21	Fam-38_2009_S85	85	3048	38	2009 H
2901_1695_S86_H	2014	D	08.04.2014	S86	13.3	20	Fam-31_2009_S86	86	3049	31	2009 F

2901_1695_S87_A	2014	D	08.04.2014	S87	13.6	20	Fam-51_2009_	S87	87	3050	51	2009	W
2901_1695_S88_E	2014	D	08.04.2014	S88	15.5	30	Fam-72_2010_	S88	88	3051	72	2010	H
2901_1695_S90_D	2014	D	08.04.2014	S90	12.4	16	Fam-63_2010_	S90	90	3052	63	2010	F
2901_1695_S91_E	2014	D	08.04.2014	S91	13.5	20.5	Fam-72_2010_	S91	91	3053	72	2010	H
2901_1695_S92_F	2014	D	08.04.2014	S92	14.6	27	Fam-31_2009_	S92	92	3054	31	2009	F
2901_1695_S93_C	2014	D	08.04.2014	S93	14.2	23.5	Fam-31_2009_	S93	93	3055	31	2009	F
2901_1695_S94_H	2014	D	08.04.2014	S94	13.6	21	Fam-36_2009_	S94	94	3056	36	2009	H
2901_1696_S95_A	2014	D	08.04.2014	S95	12.3	15.5	Fam-57_2009_	S95	95	3057	57	2009	W
2901_1696_S96_E	2014	D	08.04.2014	S96	14.8	24.5	Fam-71_2010_	S96	96	3058	71	2010	F
2901_1696_S97_C	2014	D	08.04.2014	S97	12	14	Fam-80_2010_	S97	97	3059	80	2010	H
2901_1696_S98_D	2014	D	08.04.2014	S98	15.2	28	Fam-37_2009_	S98	98	3060	37	2009	F
2901_1696_S99_E	2014	D	08.04.2014	S99	12.8	17	Fam-36_2009_	S99	99	3061	36	2009	H
2901_1696_S100_	2014	T	08.04.2014	S100	13.3	16.5	Family-35_200	S100	100	3062	35	2009	F
2901_1696_S101_	2014	T	08.04.2014	S101	14.1	19.5	Family-35_200	S101	101	3063	35	2009	F

2901_1696_S102_	2014	D	08.04.2014	S102	14.7	27.5	Fam-47_2009_	S102	102	3064	47	2009	F
2901_1696_S103_	2014	T	08.04.2014	S103	14.4	23	Family-50_200	S103	103	3065	50	2009	H
2901_1696_S104_	2014	D	08.04.2014	S104	14	23	Fam-58_2009_	S104	104	3066	58	2009	W
2901_1696_S106_	2014	D	08.04.2014	S106	15.3	28.5	Fam-38_2009_	S106	106	3067	38	2009	H
2901_1696_S107_	2014	D	08.04.2014	S107	13	18	Fam-51_2009_	S107	107	3068	51	2009	W
2901_1696_S108_	2014	D	08.04.2014	S108	13.8	23	Fam-45_2009_	S108	108	3069	45	2009	F
2901_1696_S109_	2014	D	08.04.2014	S109	13.4	19	Fam-31_2009_	S109	109	3070	31	2009	F
2901_1696_S110_	2014	D	08.04.2014	S110	14.4	23.5	Fam-36_2009_	S110	110	3071	36	2009	H
2901_1696_S111_	2014	D	08.04.2014	S111	13.7	20.5	Fam-36_2009_	S111	111	3072	36	2009	H
2901_1696_S112_	2014	D	08.04.2014	S112	14.2	23	Fam-47_2009_	S112	112	3073	47	2009	F
2901_1696_S113_	2014	D	08.04.2014	S113	13.5	21	Fam-35_2009_	S113	113	3074	35	2009	F
2901_1696_S114_	2014	D	08.04.2014	S114	12.4	17	Fam-41_2009_	S114	114	3075	41	2009	F
2901_1696_S115_	2014	D	08.04.2014	S115	13.4	19	Fam-51_2009_	S115	115	3076	51	2009	W
2901_1696_S116_	2014	D	08.04.2014	S116	14	22	Fam-47_2009_	S116	116	3077	47	2009	F

2901_1696_S117_	2014	D	08.04.2014	S117	12.4	17	Fam-80_2010_	S117	117	3078	80	2010 H
2901_1696_S119_	2014	D	08.04.2014	S119	12.5	18	Fam-85_2010_	S119	119	3079	85	2010 W
2901_1696_S120_	2014	D	08.04.2014	S120	14.7	27.5	Fam-39_2009_	S120	120	3080	39	2009 F
2901_1696_S121_	2014	D	08.04.2014	S121	14.9	25	Fam-35_2009_	S121	121	3081	35	2009 F
2901_1696_S122_	2014	D	08.04.2014	S122	14	23.5	Fam-31_2009_	S122	122	3082	31	2009 F
2901_1696_S123_	2014	D	08.04.2014	S123	13.5	21	Fam-51_2009_	S123	123	3083	51	2009 W
2901_1696_S124_	2014	D	08.04.2014	S124	12.8	17.5	Fam-31_2009_	S124	124	3084	31	2009 F
2901_1696_S125_	2014	D	08.04.2014	S125	13.6	21	Fam-54_2009_	S125	125	3085	54	2009 W
2901_1696_S126_	2014	D	08.04.2014	S126	14	22.5	Fam-31_2009_	S126	126	3086	31	2009 F
2901_1696_S127_	2014	D	08.04.2014	S127	14.5	24	Fam-37_2009_	S127	127	3087	37	2009 F
2901_1696_S128_	2014	D	08.04.2014	S128	13.5	20	Fam-31_2009_	S128	128	3088	31	2009 F
2901_1696_S129_	2014	D	08.04.2014	S129	14.3	23	Fam-35_2009_	S129	129	3089	35	2009 F
2901_1696_S130_	2014	D	08.04.2014	S130	13.4	19.5	Fam-51_2009_	S130	130	3090	51	2009 W
2901_1696_S132_	2014	D	10.04.2014	S132	12.4	16	Fam-85_2010_	S132	132	3091	85	2010 W

2901_1696_S134_	2014	D	10.04.2014	S134	14.8	26	Fam-47_2009_	S134	134	3092	47	2009 F
2901_1696_S135_	2014	D	10.04.2014	S135	13.5	29.5	Fam-61_1_201	S135	135	3093	61	2010 F
2901_1696_S136_	2014	D	10.04.2014	S136	13.8	21.5	Fam-38_2009_	S136	136	3094	38	2009 H
2901_1696_S137_	2014	D	10.04.2014	S137	14.2	21	Fam-48_2009_	S137	137	3095	48	2009 H
2901_1696_S138_	2014	D	10.04.2014	S138	14.1	22.5	Fam-35_2009_	S138	138	3096	35	2009 F
2901_1696_S139_	2014	D	10.04.2014	S139	14.9	26	Fam-37_2009_	S139	139	3097	37	2009 F
2901_1696_S140_	2014	D	10.04.2014	S140	11.2	11.5	Fam-72_2010_	S140	140	3098	72	2010 H
2901_1696_S141_	2014	D	10.04.2014	S141	13.3	19	Fam-54_2009_	S141	141	3099	54	2009 W
2901_1696_S142_	2014	D	10.04.2014	S142	13	17	Fam-35_2009_	S142	142	3100	35	2009 F
2901_1696_S143_	2014	D	10.04.2014	S143	13.6	22	Fam-40_2009_	S143	143	3101	40	2009 H
2901_1696_S144_	2014	D	10.04.2014	S144	12.6	15.5	Fam-82_2010_	S144	144	3102	82	2010 W
2901_1696_S145_	2014	D	10.04.2014	S145	12.5	15	Fam-51_2009_	S145	145	3103	51	2009 W
2901_1696_S146_	2014	D	10.04.2014	S146	13.8	20.5	Fam-51_2009_	S146	146	3104	51	2009 W
2901_1696_S147_	2014	D	10.04.2014	S147	12.9	17	Fam-58_2009_	S147	147	3105	58	2009 W

2901_1696_S148_	2014	D	10.04.2014	S148	13.3	19	Fam-37_2009_	S148	148	3106	37	2009 F
2901_1696_S149_	2014	D	10.04.2014	S149	14.1	21	Fam-40_2009_	S149	149	3107	40	2009 H
2901_1696_S150_	2014	D	10.04.2014	S150	12.5	17.5	Fam-56_2009_	S150	150	3108	56	2009 W
2901_1696_S151_	2014	D	10.04.2014	S151	13.6	21	Fam-54_2009_	S151	151	3109	54	2009 W
2901_1696_S152_	2014	D	10.04.2014	S152	13.4	18.5	Fam-35_2009_	S152	152	3110	35	2009 F
2901_1696_S153_	2014	D	10.04.2014	S153	14.6	22	Fam-50_2009_	S153	153	3111	50	2009 H
2901_1696_S154_	2014	D	10.04.2014	S154	12.2	14.5	Fam-72_2010_	S154	154	3112	72	2010 H
2901_1696_S155_	2014	D	10.04.2014	S155	13.4	18	Fam-58_2009_	S155	155	3113	58	2009 W
2901_1696_S156_	2014	D	10.04.2014	S156	13.5	19.5	Fam-57_2009_	S156	156	3114	57	2009 W
2901_1696_S158_	2014	D	10.04.2014	S158	14.9	26	Fam-48_2009_	S158	158	3115	48	2009 H
2901_1696_S160_	2014	D	11.04.2014	S160	14.2	21.5	Fam-38_2009_	S160	160	3116	38	2009 H
2902_1696_S161_	2014	D	11.04.2014	S161	14.6	27	Fam-31_2009_	S161	161	3117	31	2009 F
2902_1696_S163_	2014	D	11.04.2014	S163	13.1	17	Fam-36_2009_	S163	163	3118	36	2009 H
2902_1696_S165_	2014	D	11.04.2014	S165	12.6	17	Fam-72_2010_	S165	165	3119	72	2010 H

2902_1696_S166_	2014	D	11.04.2014	S166	13.6	20.5	Fam-51_2009_S166	166	3120	51	2009	W
2902_1696_S167_	2014	D	11.04.2014	S167	13.9	22	Fam-75_2010_S167	167	3121	75	2010	F
2902_1696_S168_	2014	D	11.04.2014	S168	13.5	22	Fam-31_2009_S168	168	3122	31	2009	F
2902_1696_S169_	2014	D	11.04.2014	S169	13.5	19	Fam-50_2009_S169	169	3123	50	2009	H
2902_1696_S170_	2014	D	11.04.2014	S170	15.4	30	Fam-36_2009_S170	170	3124	36	2009	H
2902_1696_S171_	2014	D	11.04.2014	S171	14.2	22	Fam-38_2009_S171	171	3125	38	2009	H
2902_1696_S172_	2014	D	14.04.2014	S172	15.5	29	Fam-37_2009_S172	172	3126	37	2009	F
2902_1696_S173_	2014	D	14.04.2014	S173	13.6	19	Fam-51_2009_S173	173	3127	51	2009	W
2902_1696_S174_	2014	D	14.04.2014	S174	12.7	15.5	Fam-57_2009_S174	174	3128	57	2009	W
2902_1696_S175_	2014	D	14.04.2014	S175	15.4	29	Fam-31_2009_S175	175	3129	31	2009	F
2902_1696_S176_	2014	D	14.04.2014	S176	13.1	17	Fam-84_2010_S176	176	3130	84	2010	W
2902_1696_S177_	2014	D	14.04.2014	S177	13.9	22	Fam-36_2009_S177	177	3131	36	2009	H
2902_1696_S178_	2014	D	14.04.2014	S178	16.4	34	Fam-37_2009_S178	178	3132	37	2009	F
2902_1696_S179_	2014	D	14.04.2014	S179	15.3	31	Fam-49_2009_S179	179	3133	49	2009	F

2902_1696_S180_	2014	D	14.04.2014	S180	16.2	32	Fam-35_2009_	S180	180	3134	35	2009	F
2902_1696_S181_	2014	D	14.04.2014	S181	15.2	29.5	Fam-79_2010_	S181	181	3135	79	2010	F
2902_1696_S182_	2014	D	14.04.2014	S182	13	18	Fam-61_1_201	S182	182	3136	61	2010	F
2902_1696_S183_	2014	D	14.04.2014	S183	15.2	28	Fam-61_1_201	S183	183	3137	61	2010	F
2902_1696_S184_	2014	D	14.04.2014	S184	15.2	28	Fam-48_2009_	S184	184	3138	48	2009	H
2902_1696_S185_	2014	D	14.04.2014	S185	12	13.5	Fam-85_2010_	S185	185	3139	85	2010	W
2902_1696_S186_	2014	D	14.04.2014	S186	14.5	24	Fam-35_2009_	S186	186	3140	35	2009	F
2902_1696_S187_	2014	D	14.04.2014	S187	13.8	21	Fam-31_2009_	S187	187	3141	31	2009	F
2902_1696_S188_	2014	D	14.04.2014	S188	15	29	Fam-36_2009_	S188	188	3142	36	2009	H
2902_1697_S189_	2014	D	14.04.2014	S189	13.2	18.5	Fam-61_1_201	S189	189	3143	61	2010	F
2902_1697_S190_	2014	D	14.04.2014	S190	13.5	19.5	Fam-58_2009_	S190	190	3144	58	2009	W
2902_1697_S191_	2014	D	14.04.2014	S191	13.8	20.5	Fam-72_2010_	S191	191	3145	72	2010	H
2902_1697_S192_	2014	T	14.04.2014	S192	12.6	15	Family-36_200	S192	192	3146	36	2009	H
2902_1697_S193_	2014	D	14.04.2014	S193	14.2	23	Fam-37_2009_	S193	193	3147	37	2009	F

2902_1697_S195_	2014	D	14.04.2014	S195	14	24	Fam-72_2010_	S195	195	3148	72	2010	H
2902_1697_S196_	2014	D	14.04.2014	S196	14.7	27	Fam-47_2009_	S196	196	3149	47	2009	F
2902_1697_S197_	2014	D	14.04.2014	S197	13.9	21.5	Fam-58_2009_	S197	197	3150	58	2009	W
2902_1697_S198_	2014	D	14.04.2014	S198	15.1	29	Fam-80_2010_	S198	198	3151	80	2010	H
2902_1697_S199_	2014	D	14.04.2014	S199	14	24	Fam-31_2009_	S199	199	3152	31	2009	F
2902_1697_S200_	2014	D	14.04.2014	S200	13.1	18.5	Fam-44_2009_	S200	200	3153	44	2009	H
2902_1697_S201_	2014	D	14.04.2014	S201	12.3	15.5	Fam-72_2010_	S201	201	3154	72	2010	H
2902_1697_S202_	2014	D	14.04.2014	S202	15	27	Fam-47_2009_	S202	202	3155	47	2009	F
2902_1697_S203_	2014	D	14.04.2014	S203	15.7	29.5	Fam-43_2009_	S203	203	3156	43	2009	F
2902_1697_S204_	2014	D	14.04.2014	S204	13.1	18.5	Fam-89_2010_	S204	204	3157	89	2010	W
2902_1697_S205_	2014	D	14.04.2014	S205	16.2	34	Fam-37_2009_	S205	205	3158	37	2009	F
2902_1697_S206_	2014	D	14.04.2014	S206	14	22	Fam-58_2009_	S206	206	3159	58	2009	W
2902_1697_S207_	2014	D	14.04.2014	S207	15.3	28.5	Fam-50_2009_	S207	207	3160	50	2009	H
2902_1697_S208_	2014	D	14.04.2014	S208	14.8	26	Fam-31_2009_	S208	208	3161	31	2009	F

2902_1697_S209_	2014	D	14.04.2014	S209	14.8	27	Fam-47_2009_S209	209	3162	47	2009 F
2902_1697_S210_	2014	D	14.04.2014	S210	13.8	21	Fam-71_2010_S210	210	3163	71	2010 F
2902_1697_S211_	2014	D	14.04.2014	S211	14.1	23	Fam-35_2009_S211	211	3164	35	2009 F
2902_1697_S212_	2014	D	14.04.2014	S212	11.4	11	Fam-35_2009_S212	212	3165	35	2009 F
2902_1697_S213_	2014	D	14.04.2014	S213	12.9	18	Fam-56_2009_S213	213	3166	56	2009 W
2902_1697_S214_	2014	D	14.04.2014	S214	13.3	20.5	Fam-37_2009_S214	214	3167	37	2009 F
2902_1697_S215_	2014	D	14.04.2014	S215	13.3	19	Fam-51_2009_S215	215	3168	51	2009 W
2902_1697_S216_	2014	D	14.04.2014	S216	12.9	17	Fam-58_2009_S216	216	3169	58	2009 W
2902_1697_S217_	2014	D	14.04.2014	S217	13.2	18.5	Fam-51_2009_S217	217	3170	51	2009 W
2902_1697_S218_	2014	D	14.04.2014	S218	13.5	20.5	Fam-85_2010_S218	218	3171	85	2010 W
2902_1697_S219_	2014	D	14.04.2014	S219	15.4	29	Fam-48_2009_S219	219	3172	48	2009 H
2902_1697_S220_	2014	D	14.04.2014	S220	15.4	31	Fam-47_2009_S220	220	3173	47	2009 F
2902_1697_S221_	2014	D	14.04.2014	S221	13.2	19	Fam-76_2010_S221	221	3174	76	2010 H
2902_1697_S222_	2014	D	14.04.2014	S222	15.5	29.5	Fam-35_2009_S222	222	3175	35	2009 F

2902_1697_S223_	2014	D	14.04.2014	S223	13.5	19	Fam-57_2009_	S223	223	3176	57	2009	W
2902_1697_S224_	2014	D	16.04.2014	S224	15.2	28	Fam-43_2009_	S224	224	3177	43	2009	F
2902_1697_S225_	2014	D	16.04.2014	S225	13	17.5	Fam-36_2009_	S225	225	3178	36	2009	H
2902_1697_S226_	2014	D	16.04.2014	S226	15	27.5	Fam-31_2009_	S226	226	3179	31	2009	F
2902_1697_S227_	2014	D	16.04.2014	S227	12	16	Fam-51_2009_	S227	227	3180	51	2009	W
2902_1697_S228_	2014	D	16.04.2014	S228	14.5	25	Fam-57_2009_	S228	228	3181	57	2009	W
2902_1697_S229_	2014	D	16.04.2014	S229	13.8	22.5	Fam-62_1_201	S229	229	3182	62	2010	H
2902_1697_S230_	2014	D	16.04.2014	S230	16.1	35	Fam-31_2009_	S230	230	3183	31	2009	F
2902_1697_S231_	2014	D	16.04.2014	S231	16.2	34.5	Fam-71_2010_	S231	231	3184	71	2010	F
2902_1697_S232_	2014	D	16.04.2014	S232	13.5	20	Fam-58_2009_	S232	232	3185	58	2009	W
2902_1697_S233_	2014	D	16.04.2014	S233	13.5	19	Fam-51_2009_	S233	233	3186	51	2009	W
2902_1697_S234_	2014	D	16.04.2014	S234	14.8	28	Fam-45_2009_	S234	234	3187	45	2009	F
2902_1697_S235_	2014	D	16.04.2014	S235	14	22	Fam-36_2009_	S235	235	3188	36	2009	H
2902_1697_S236_	2014	D	16.04.2014	S236	15.7	31	Fam-31_2009_	S236	236	3189	31	2009	F

2902_1697_S237_	2014	D	16.04.2014	S237	13.3	19.5	Fam-59_2009_	S237	237	3190	59	2009 W
2902_1697_S238_	2014	D	16.04.2014	S238	14	22	Fam-61_1_201	S238	238	3191	61	2010 F
2902_1697_S240_	2014	D	16.04.2014	S240	14.5	25.5	Fam-36_2009_	S240	240	3192	36	2009 H
2903_1697_S241_	2014	D	16.04.2014	S241	14.3	24	Fam-43_2009_	S241	241	3193	43	2009 F
2903_1697_S242_	2014	D	16.04.2014	S242	13.1	18	Fam-36_2009_	S242	242	3194	36	2009 H
2903_1697_S244_	2014	D	16.04.2014	S244	14.1	22	Fam-89_2010_	S244	244	3195	89	2010 W
2903_1697_S245_	2014	D	16.04.2014	S245	13.7	19	Fam-35_2009_	S245	245	3196	35	2009 F
2903_1697_S246_	2014	D	16.04.2014	S246	14	23	Fam-48_2009_	S246	246	3197	48	2009 H
2903_1697_S247_	2014	D	16.04.2014	S247	12	14.4	Fam-58_2009_	S247	247	3198	58	2009 W
2903_1697_S248_	2014	D	16.04.2014	S248	13.6	20	Fam-50_2009_	S248	248	3199	50	2009 H
2903_1697_S249_	2014	D	22.04.2014	S249	14.5	24	Fam-35_2009_	S249	249	3200	35	2009 F
2903_1697_S250_	2014	D	22.04.2014	S250	13.9	21.5	Fam-61_1_201	S250	250	3201	61	2010 F
2903_1697_S251_	2014	D	22.04.2014	S251	13.9	22	Fam-35_2009_	S251	251	3202	35	2009 F
2903_1697_S252_	2014	D	22.04.2014	S252	13	20	Fam-38_2009_	S252	252	3203	38	2009 H

2903_1697_S253_	2014	D	22.04.2014	S253	13.2	20	Fam-65_2010_	S253	253	3204	65	2010 F
2903_1697_S254_	2014	D	22.04.2014	S254	14.4	23.5	Fam-69_2010_	S254	254	3205	69	2010 F
2903_1697_S255_	2014	D	22.04.2014	S255	14.2	24.5	Fam-72_2010_	S255	255	3206	72	2010 H
2903_1697_S256_	2014	D	22.04.2014	S256	12.4	15	Fam-59_2009_	S256	256	3207	59	2009 W
2903_1697_S257_	2014	D	22.04.2014	S257	13.2	19	Fam-89_2010_	S257	257	3208	89	2010 W
2903_1697_S258_	2014	D	22.04.2014	S258	16.2	34	Fam-31_2009_	S258	258	3209	31	2009 F
2903_1697_S259_	2014	D	22.04.2014	S259	13.8	21	Fam-53_2009_	S259	259	3210	53	2009 W
2903_1697_S260_	2014	D	22.04.2014	S260	15.5	27.5	Fam-64_2010_	S260	260	3211	64	2010 H
2903_1697_S261_	2014	D	22.04.2014	S261	14.5	21.5	Fam-43_2009_	S261	261	3212	43	2009 F
2903_1697_S262_	2014	D	22.04.2014	S262	15.4	28	Fam-38_2009_	S262	262	3213	38	2009 H
2903_1697_S263_	2014	D	22.04.2014	S263	14.5	25.5	Fam-31_2009_	S263	263	3214	31	2009 F
2903_1697_S264_	2014	D	22.04.2014	S264	12.4	15.5	Fam-61_1_201	S264	264	3215	61	2010 F
2903_1697_S265_	2014	D	22.04.2014	S265	14.3	23	Fam-51_2009_	S265	265	3216	51	2009 W
2903_1697_S266_	2014	D	22.04.2014	S266	15	29	Fam-31_2009_	S266	266	3217	31	2009 F

2903_1697_S268_	2014	D	22.04.2014	S268	12.5	16	Fam-56_2009_S268	268	3218	56	2009	W
2903_1697_S269_	2014	D	22.04.2014	S269	13.9	21.5	Fam-85_2010_S269	269	3219	85	2010	W
2903_1697_S270_	2014	D	22.04.2014	S270	16.4	34	Fam-37_2009_S270	270	3220	37	2009	F
2903_1697_S271_	2014	D	22.04.2014	S271	14.7	27	Fam-47_2009_S271	271	3221	47	2009	F
2903_1697_S273_	2014	D	22.04.2014	S273	14.2	25.5	Fam-35_2009_S273	273	3222	35	2009	F
2903_1697_S274_	2014	D	22.04.2014	S274	13.6	20	Fam-51_2009_S274	274	3223	51	2009	W
2903_1697_S275_	2014	D	22.04.2014	S275	15.3	30	Fam-38_2009_S275	275	3224	38	2009	H
2903_1697_S276_	2014	D	22.04.2014	S276	11.9	14	Fam-56_2009_S276	276	3225	56	2009	W
2903_1697_S277_	2014	D	22.04.2014	S277	12.5	15.5	Fam-73_2010_S277	277	3226	73	2010	F
2903_1697_S279_	2014	D	22.04.2014	S279	12.8	17	Fam-58_2009_S279	279	3227	58	2009	W
2903_1697_S280_	2014	D	22.04.2014	S280	14	23.5	Fam-47_2009_S280	280	3228	47	2009	F
2903_1697_S281_	2014	D	22.04.2014	S281	13.9	22	Fam-31_2009_S281	281	3229	31	2009	F
2903_1697_S282_	2014	D	22.04.2014	S282	13.8	22	Fam-35_2009_S282	282	3230	35	2009	F
2903_1698_S283_	2014	D	22.04.2014	S283	12.6	17	Fam-90_2010_S283	283	3231	90	2010	W

2903_1698_S284_	2014	D	22.04.2014	S284	14.5	24	Fam-50_2009_	S284	284	3232	50	2009	H
2903_1698_S285_	2014	D	22.04.2014	S285	14	22	Fam-36_2009_	S285	285	3233	36	2009	H
2903_1698_S287_	2014	D	22.04.2014	S287	12.7	16.5	Fam-42_2009_	S287	287	3234	42	2009	H
2903_1698_S288_	2014	D	22.04.2014	S288	13	17.5	Fam-72_2010_	S288	288	3235	72	2010	H
2903_1698_S289_	2014	D	22.04.2014	S289	15.2	28.5	Fam-61_1_201	S289	289	3236	61	2010	F
2903_1698_S290_	2014	D	22.04.2014	S290	14.7	24	Fam-40_2009_	S290	290	3237	40	2009	H
2903_1698_S291_	2014	D	22.04.2014	S291	13.6	21	Fam-37_2009_	S291	291	3238	37	2009	F
2903_1698_S292_	2014	D	22.04.2014	S292	13.8	21	Fam-63_2010_	S292	292	3239	63	2010	F
2903_1698_S293_	2014	D	22.04.2014	S293	13.9	21.5	Fam-45_2009_	S293	293	3240	45	2009	F
2903_1698_S294_	2014	D	22.04.2014	S294	13.2	18.5	Fam-73_2010_	S294	294	3241	73	2010	F
2903_1698_S295_	2014	D	22.04.2014	S295	14	24	Fam-80_2010_	S295	295	3242	80	2010	H
2903_1698_S296_	2014	D	22.04.2014	S296	12.7	15.5	Fam-72_2010_	S296	296	3243	72	2010	H
2903_1698_S297_	2014	D	22.04.2014	S297	13.3	19.5	Fam-35_2009_	S297	297	3244	35	2009	F
2903_1698_S298_	2014	D	22.04.2014	S298	14	21.5	Fam-31_2009_	S298	298	3245	31	2009	F

2903_1698_S299_	2014	D	22.04.2014	S299	15.9	31.5	Fam-79_2010_	S299	299	3246	79	2010 F
2903_1698_S300_	2014	D	22.04.2014	S300	16	33	Fam-47_2009_	S300	300	3247	47	2009 F
2903_1698_S301_	2014	D	22.04.2014	S301	12.5	16	Fam-71_2010_	S301	301	3248	71	2010 F
2903_1698_S302_	2014	D	22.04.2014	S302	13.4	17.5	Fam-43_2009_	S302	302	3249	43	2009 F
2903_1698_S303_	2014	D	25.04.2014	S303	12	14	Fam-61_1_201	S303	303	3250	61	2010 F
2903_1698_S304_	2014	D	25.04.2014	S304	13.9	21	Fam-59_2009_	S304	304	3251	59	2009 W
2903_1698_S305_	2014	D	25.04.2014	S305	15.2	27	Fam-48_2009_	S305	305	3252	48	2009 H
2903_1698_S306_	2014	D	25.04.2014	S306	13.2	19.5	Fam-36_2009_	S306	306	3253	36	2009 H
2903_1698_S307_	2014	D	25.04.2014	S307	14.7	26.5	Fam-61_1_201	S307	307	3254	61	2010 F
2903_1698_S308_	2014	D	25.04.2014	S308	15.5	31	Fam-31_2009_	S308	308	3255	31	2009 F
2903_1698_S309_	2014	D	25.04.2014	S309	13.6	19.5	Fam-38_2009_	S309	309	3256	38	2009 H
2903_1698_S311_	2014	D	25.04.2014	S311	12.7	16.5	Fam-75_2010_	S311	311	3257	75	2010 F
2903_1698_S312_	2014	D	25.04.2014	S312	12.4	14	Fam-35_2009_	S312	312	3258	35	2009 F
2903_1698_S313_	2014	D	25.04.2014	S313	14.2	23.5	Fam-61_1_201	S313	313	3259	61	2010 F

2903_1698_S314_	2014	D	25.04.2014	S314	14	21.5	Fam-61_1_201	S314	314	3260	61	2010	F
2903_1698_S315_	2014	D	25.04.2014	S315	15	29	Fam-49_2009_	S315	315	3261	49	2009	F
2903_1698_S316_	2014	D	25.04.2014	S316	13.7	21	Fam-35_2009_	S316	316	3262	35	2009	F
2903_1698_S317_	2014	D	25.04.2014	S317	14.7	26.5	Fam-77_2010_	S317	317	3263	77	2010	F
2903_1698_S318_	2014	D	25.04.2014	S318	14.7	24	Fam-35_2009_	S318	318	3264	35	2009	F
2903_1698_S319_	2014	T	25.04.2014	S319	14.8	24	Family-70_201	S319	319	3265	70	2010	H
2903_1698_S320_	2014	D	25.04.2014	S320	13.7	21	Fam-58_2009_	S320	320	3266	58	2009	W
2904_1698_S321_	2014	D	25.04.2014	S321	12.6	17	Fam-58_2009_	S321	321	3267	58	2009	W
2904_1698_S323_	2014	D	25.04.2014	S323	15.2	30.5	Fam-66_2010_	S323	323	3268	66	2010	H
2904_1698_S324_	2014	D	25.04.2014	S324	12.5	16	Fam-61_1_201	S324	324	3269	61	2010	F
2904_1698_S325_	2014	D	25.04.2014	S325	14.7	28	Fam-42_2009_	S325	325	3270	42	2009	H
2904_1698_S326_	2014	D	25.04.2014	S326	12.9	17.5	Fam-42_2009_	S326	326	3271	42	2009	H
2904_1698_S327_	2014	D	25.04.2014	S327	14	22	Fam-38_2009_	S327	327	3272	38	2009	H
2904_1698_S328_	2014	D	25.04.2014	S328	15.2	28	Fam-39_2009_	S328	328	3273	39	2009	F

2904_1698_S329_	2014	D	25.04.2014	S329	15	29.5	Fam-70_2010_	S329	329	3274	70	2010	H
2904_1698_S330_	2014	D	25.04.2014	S330	14	23.5	Fam-42_2009_	S330	330	3275	42	2009	H
2904_1698_S331_	2014	D	25.04.2014	S331	14.8	26.5	Fam-33_2009_	S331	331	3276	33	2009	F
2904_1698_S332_	2014	D	25.04.2014	S332	12.6	16.5	Fam-59_2009_	S332	332	3277	59	2009	W
2904_1698_S333_	2014	D	25.04.2014	S333	13.3	19.5	Fam-49_2009_	S333	333	3278	49	2009	F
2904_1698_S334_	2014	D	25.04.2014	S334	13.7	21	Fam-64_2010_	S334	334	3279	64	2010	H
2904_1698_S335_	2014	D	25.04.2014	S335	15.4	28	Fam-50_2009_	S335	335	3280	50	2009	H
2904_1698_S336_	2014	D	25.04.2014	S336	13.1	18	Fam-38_2009_	S336	336	3281	38	2009	H
2904_1698_S337_	2014	D	25.04.2014	S337	14.6	24.5	Fam-39_2009_	S337	337	3282	39	2009	F
2904_1698_S338_	2014	D	25.04.2014	S338	12.9	18	Fam-59_2009_	S338	338	3283	59	2009	W
2904_1698_S340_	2014	D	25.04.2014	S340	13.1	18	Fam-50_2009_	S340	340	3284	50	2009	H
2904_1698_S341_	2014	D	25.04.2014	S341	12.6	16.5	Fam-57_2009_	S341	341	3285	57	2009	W
2904_1698_S342_	2014	D	25.04.2014	S342	15.5	30.5	Fam-33_2009_	S342	342	3286	33	2009	F
2904_1698_S343_	2014	D	25.04.2014	S343	12.9	16.5	Fam-36_2009_	S343	343	3287	36	2009	H

2904_1698_S344_	2014	D	25.04.2014	S344	14.1	20.5	Fam-57_2009_	S344	344	3288	57	2009	W
2904_1698_S346_	2014	D	25.04.2014	S346	14.3	22.5	Fam-36_2009_	S346	346	3289	36	2009	H
2904_1698_S347_	2014	D	25.04.2014	S347	13.5	21	Fam-38_2009_	S347	347	3290	38	2009	H
2904_1698_S348_	2014	D	25.04.2014	S348	14.7	26.5	Fam-35_2009_	S348	348	3291	35	2009	F
2904_1698_S349_	2014	T	25.04.2014	S349	13.3	17	Family-36_200	S349	349	3292	36	2009	H
2904_1698_S352_	2014	D	25.04.2014	S352	13.8	22	Fam-42_2009_	S352	352	3293	42	2009	H
2904_1698_S353_	2014	D	25.04.2014	S353	13.4	20.5	Fam-56_2009_	S353	353	3294	56	2009	W
2904_1698_S354_	2014	D	25.04.2014	S354	12.5	17.5	Fam-58_2009_	S354	354	3295	58	2009	W
2904_1698_S355_	2014	D	25.04.2014	S355	13	17	Fam-58_2009_	S355	355	3296	58	2009	W
2904_1698_S356_	2014	D	25.04.2014	S356	14.3	25	Fam-66_2010_	S356	356	3297	66	2010	H
2904_1698_S357_	2014	D	25.04.2014	S357	16.7	33	Fam-71_2010_	S357	357	3298	71	2010	F
2904_1698_S358_	2014	D	25.04.2014	S358	15	29.5	Fam-31_2009_	S358	358	3299	31	2009	F
2904_1698_S359_	2014	D	25.04.2014	S359	15	27	Fam-84_2010_	S359	359	3300	84	2010	W
2904_1698_S360_	2014	D	25.04.2014	S360	13.2	19.5	Fam-58_2009_	S360	360	3301	58	2009	W

2904_1698_S361_	2014	D	25.04.2014	S361	14.4	26	Fam-80_2010_	S361	361	3302	80	2010 H
2904_1698_S362_	2014	D	25.04.2014	S362	14	24	Fam-58_2009_	S362	362	3303	58	2009 W
2904_1698_S363_	2014	D	25.04.2014	S363	13	19	Fam-80_2010_	S363	363	3304	80	2010 H
2904_1698_S364_	2014	D	25.04.2014	S364	14.8	27	Fam-50_2009_	S364	364	3305	50	2009 H
2904_1698_S365_	2014	D	25.04.2014	S365	13.2	20	Fam-31_2009_	S365	365	3306	31	2009 F
2904_1698_S366_	2014	D	25.04.2014	S366	14.9	26.5	Fam-47_2009_	S366	366	3307	47	2009 F
2904_1698_S367_	2014	T	25.04.2014	S367	13	17	Family-45_200	S367	367	3308	45	2009 F
2904_1698_S368_	2014	D	25.04.2014	S368	14.3	23	Fam-57_2009_	S368	368	3309	57	2009 W
2904_1698_S369_	2014	D	25.04.2014	S369	14.5	25	Fam-31_2009_	S369	369	3310	31	2009 F
2904_1698_S370_	2014	D	25.04.2014	S370	12.9	16.5	Fam-64_2010_	S370	370	3311	64	2010 H
2904_1698_S371_	2014	D	25.04.2014	S371	13	19.5	Fam-72_2010_	S371	371	3312	72	2010 H
2904_1698_S372_	2014	D	25.04.2014	S372	13.9	22.5	Fam-38_2009_	S372	372	3313	38	2009 H
2904_1698_S373_	2014	D	25.04.2014	S373	13.8	20	Fam-36_2009_	S373	373	3314	36	2009 H
2904_1698_S374_	2014	D	25.04.2014	S374	14.1	24	Fam-42_2009_	S374	374	3315	42	2009 H

2904_1698_S375_	2014	D	25.04.2014	S375	14.5	26.5	Fam-80_2010_	S375	375	3316	80	2010	H
2904_1698_S376_	2014	D	25.04.2014	S376	13.2	18.5	Fam-36_2009_	S376	376	3317	36	2009	H
2904_1699_S377_	2014	D	25.04.2014	S377	15.2	29	Fam-35_2009_	S377	377	3318	35	2009	F
2904_1699_S379_	2014	D	25.04.2014	S379	13	17	Fam-70_2010_	S379	379	3319	70	2010	H
2904_1699_S381_	2014	D	25.04.2014	S381	13.5	25.5	Fam-63_2010_	S381	381	3320	63	2010	F
2904_1699_S382_	2014	D	25.04.2014	S382	12.4	16	Fam-44_2009_	S382	382	3321	44	2009	H
2904_1699_S383_	2014	D	25.04.2014	S383	13.3	19	Fam-57_2009_	S383	383	3322	57	2009	W
2904_1699_S384_	2014	D	25.04.2014	S384	13.5	20.5	Fam-38_2009_	S384	384	3323	38	2009	H
2904_1699_S385_	2014	D	25.04.2014	S385	12.6	17	Fam-63_2010_	S385	385	3324	63	2010	F
2904_1699_S386_	2014	D	25.04.2014	S386	15.9	31	Fam-48_2009_	S386	386	3325	48	2009	H
2904_1699_S387_	2014	D	25.04.2014	S387	12.6	17.5	Fam-41_2009_	S387	387	3326	41	2009	F
2904_1699_S388_	2014	D	25.04.2014	S388	13.2	19.5	Fam-80_2010_	S388	388	3327	80	2010	H
2904_1699_S389_	2014	D	25.04.2014	S389	15.2	28	Fam-38_2009_	S389	389	3328	38	2009	H
2904_1699_S390_	2014	D	25.04.2014	S390	14.6	25.5	Fam-64_2010_	S390	390	3329	64	2010	H

2904_1699_S391_	2014	D	25.04.2014	S391	14.2	22.5	Fam-49_2009_	S391	391	3330	49	2009	F
2904_1699_S392_	2014	D	25.04.2014	S392	13.5	20.5	Fam-38_2009_	S392	392	3331	38	2009	H
2904_1699_S393_	2014	D	25.04.2014	S393	14.6	26.5	Fam-47_2009_	S393	393	3332	47	2009	F
2904_1699_S395_	2014	D	25.04.2014	S395	15.5	30	Fam-31_2009_	S395	395	3333	31	2009	F
2904_1699_S396_	2014	D	25.04.2014	S396	14.5	24.5	Fam-35_2009_	S396	396	3334	35	2009	F
2904_1699_S397_	2014	D	25.04.2014	S397	12.7	17	Fam-38_2009_	S397	397	3335	38	2009	H
2904_1699_S398_	2014	D	25.04.2014	S398	14.5	25	Fam-44_2009_	S398	398	3336	44	2009	H
2904_1699_S399_	2014	D	25.04.2014	S399	13.3	19.5	Fam-50_2009_	S399	399	3337	50	2009	H
2904_1699_S400_	2014	D	25.04.2014	S400	14	22	Fam-50_2009_	S400	400	3338	50	2009	H
2905_1699_S401_	2014	D	25.04.2014	S401	14.1	23	Fam-43_2009_	S401	401	3339	43	2009	F
2905_1699_S402_	2014	D	25.04.2014	S402	14.8	25	Fam-31_2009_	S402	402	3340	31	2009	F
2905_1699_S404_	2014	D	25.04.2014	S404	14.6	26	Fam-48_2009_	S404	404	3341	48	2009	H
2905_1699_S405_	2014	D	25.04.2014	S405	15	28	Fam-51_2009_	S405	405	3342	51	2009	W
2905_1699_S406_	2014	D	25.04.2014	S406	14.5	25.5	Fam-50_2009_	S406	406	3343	50	2009	H

2905_1699_S407_	2014	D	25.04.2014	S407	12	18	Fam-59_2009_	S407	407	3344	59	2009	W
2905_1699_S408_	2014	D	25.04.2014	S408	15	25.5	Fam-57_2009_	S408	408	3345	57	2009	W
2905_1699_S409_	2014	D	25.04.2014	S409	13.6	21.5	Fam-38_2009_	S409	409	3346	38	2009	H
2905_1699_S410_	2014	D	25.04.2014	S410	15.3	29	Fam-38_2009_	S410	410	3347	38	2009	H
2905_1699_S411_	2014	D	25.04.2014	S411	13.5	20.5	Fam-47_2009_	S411	411	3348	47	2009	F
2905_1699_S412_	2014	D	25.04.2014	S412	14.5	23	Fam-39_2009_	S412	412	3349	39	2009	F
2905_1699_S413_	2014	D	25.04.2014	S413	13.9	23	Fam-79_2010_	S413	413	3350	79	2010	F
2905_1699_S414_	2014	D	25.04.2014	S414	13.2	19	Fam-90_2010_	S414	414	3351	90	2010	W
2905_1699_S415_	2014	D	25.04.2014	S415	14.7	24.5	Fam-50_2009_	S415	415	3352	50	2009	H
2905_1699_S416_	2014	D	25.04.2014	S416	16.9	37	Fam-38_2009_	S416	416	3353	38	2009	H
2905_1699_S417_	2014	D	25.04.2014	S417	13	18.5	Fam-36_2009_	S417	417	3354	36	2009	H
2905_1699_S418_	2014	D	25.04.2014	S418	14.8	26	Fam-36_2009_	S418	418	3355	36	2009	H
2905_1699_S419_	2014	D	25.04.2014	S419	14.7	24.5	Fam-57_2009_	S419	419	3356	57	2009	W
2905_1699_S420_	2014	D	25.04.2014	S420	15	25	Fam-50_2009_	S420	420	3357	50	2009	H

2905_1699_S421_	2014	D	25.04.2014	S421	14	23	Fam-31_2009_	S421	421	3358	31	2009 F
2905_1699_S422_	2014	D	25.04.2014	S422	15	27.5	Fam-31_2009_	S422	422	3359	31	2009 F
2905_1699_S423_	2014	D	25.04.2014	S423	14.5	25	Fam-38_2009_	S423	423	3360	38	2009 H
2905_1699_S424_	2014	D	25.04.2014	S424	15	28.5	Fam-38_2009_	S424	424	3361	38	2009 H
2905_1699_S425_	2014	D	25.04.2014	S425	14	22.5	Fam-38_2009_	S425	425	3362	38	2009 H
2905_1699_S426_	2014	D	25.04.2014	S426	15	25	Fam-48_2009_	S426	426	3363	48	2009 H
2905_1699_S427_	2014	D	25.04.2014	S427	14.2	23.5	Fam-36_2009_	S427	427	3364	36	2009 H
2905_1699_S428_	2014	D	25.04.2014	S428	14.2	23.5	Fam-58_2009_	S428	428	3365	58	2009 W
2905_1699_S429_	2014	D	25.04.2014	S429	14	22.5	Fam-36_2009_	S429	429	3366	36	2009 H
2905_1699_S430_	2014	D	25.04.2014	S430	15.5	27.5	Fam-80_2010_	S430	430	3367	80	2010 H
2905_1699_S431_	2014	D	25.04.2014	S431	16	34.5	Fam-66_2010_	S431	431	3368	66	2010 H
2905_1699_S432_	2014	D	25.04.2014	S432	16.3	33	Fam-69_2010_	S432	432	3369	69	2010 F
2905_1699_S433_	2014	D	25.04.2014	S433	13.8	21.5	Fam-35_2009_	S433	433	3370	35	2009 F
2905_1699_S434_	2014	D	25.04.2014	S434	14	24	Fam-33_2009_	S434	434	3371	33	2009 F

2905_1699_S435_	2014	D	25.04.2014	S435	15	26	Fam-83_2010_	S435	435	3372	83	2010	W
2905_1699_S436_	2014	T	25.04.2014	S436	13.4	18.5	Family-31_200	S436	436	3373	31	2009	F
2905_1699_S437_	2014	D	25.04.2014	S437	14	22.5	Fam-38_2009_	S437	437	3374	38	2009	H
2905_1699_S439_	2014	D	25.04.2014	S439	14	22	Fam-39_2009_	S439	439	3375	39	2009	F
2905_1699_S440_	2014	D	25.04.2014	S440	14	22.5	Fam-51_2009_	S440	440	3376	51	2009	W
2905_1699_S441_	2014	D	25.04.2014	S441	13.8	20.5	Fam-38_2009_	S441	441	3377	38	2009	H
2905_1699_S442_	2014	D	25.04.2014	S442	14.2	23	Fam-39_2009_	S442	442	3378	39	2009	F
2905_1699_S443_	2014	D	25.04.2014	S443	13.5	19	Fam-54_2009_	S443	443	3379	54	2009	W
2905_1699_S444_	2014	D	25.04.2014	S444	13.2	20.5	Fam-85_2010_	S444	444	3380	85	2010	W
2905_1699_S445_	2014	D	25.04.2014	S445	14.5	24	Fam-72_2010_	S445	445	3381	72	2010	H
2905_1699_S446_	2014	D	25.04.2014	S446	14.7	23.5	Fam-58_2009_	S446	446	3382	58	2009	W
2905_1699_S447_	2014	D	25.04.2014	S447	15	27.5	Fam-58_2009_	S447	447	3383	58	2009	W
2905_1699_S448_	2014	D	25.04.2014	S448	14.3	24	Fam-38_2009_	S448	448	3384	38	2009	H
2905_1699_S449_	2014	D	25.04.2014	S449	12.6	17	Fam-56_2009_	S449	449	3385	56	2009	W

2905_1699_S450_	2014	D	25.04.2014	S450	13.8	22.5	Fam-36_2009_	S450	450	3386	36	2009	H
2905_1699_S451_	2014	D	25.04.2014	S451	12.6	16.5	Fam-80_2010_	S451	451	3387	80	2010	H
2905_1699_S452_	2014	D	25.04.2014	S452	14.9	26.5	Fam-38_2009_	S452	452	3388	38	2009	H
2905_1699_S453_	2014	D	25.04.2014	S453	14.1	22.5	Fam-36_2009_	S453	453	3389	36	2009	H
2905_1699_S454_	2014	D	25.04.2014	S454	13.1	17.5	Fam-43_2009_	S454	454	3390	43	2009	F
2905_1699_S455_	2014	D	25.04.2014	S455	14	22	Fam-35_2009_	S455	455	3391	35	2009	F
2905_1699_S456_	2014	D	25.04.2014	S456	14.9	28.5	Fam-47_2009_	S456	456	3392	47	2009	F
2905_1699_S457_	2014	D	25.04.2014	S457	13.2	19	Fam-54_2009_	S457	457	3393	54	2009	W
2905_1699_S458_	2014	D	25.04.2014	S458	14.5	25.5	Fam-54_2009_	S458	458	3394	54	2009	W
2905_1699_S459_	2014	D	25.04.2014	S459	14.8	26	Fam-41_2009_	S459	459	3395	41	2009	F
2905_1699_S460_	2014	D	25.04.2014	S460	16	31	Fam-51_2009_	S460	460	3396	51	2009	W
2905_1699_S461_	2014	D	25.04.2014	S461	15.7	32.5	Fam-32_2009_	S461	461	3397	32	2009	H
2905_1699_S462_	2014	D	25.04.2014	S462	13.4	18	Fam-69_2010_	S462	462	3398	69	2010	F
2905_1699_S463_	2014	D	25.04.2014	S463	14.1	22	Fam-85_2010_	S463	463	3399	85	2010	W

2905_1699_S464_	2014	D	25.04.2014	S464	13	19.5	Fam-77_2010_S464	464	3400	77	2010	F
2905_1699_S465_	2014	D	25.04.2014	S465	14.4	24.5	Fam-39_2009_S465	465	3401	39	2009	F
2905_1699_S466_	2014	D	25.04.2014	S466	14	22	Fam-58_2009_S466	466	3402	58	2009	W
2905_1699_S467_	2014	D	25.04.2014	S467	15.5	30.5	Fam-41_2009_S467	467	3403	41	2009	F
2905_1699_S468_	2014	D	25.04.2014	S468	13.1	18	Fam-35_2009_S468	468	3404	35	2009	F
2905_1699_S469_	2014	D	25.04.2014	S469	13	17.5	Fam-54_2009_S469	469	3405	54	2009	W
2905_1699_S470_	2014	D	25.04.2014	S470	13	17.5	Fam-84_2010_S470	470	3406	84	2010	W
2905_1700_S471_	2014	D	25.04.2014	S471	12.9	18.5	Fam-36_2009_S471	471	3407	36	2009	H
2905_1700_S472_	2014	D	25.04.2014	S472	13.6	18.5	Fam-53_2009_S472	472	3408	53	2009	W
2905_1700_S473_	2014	D	25.04.2014	S473	15	29	Fam-58_2009_S473	473	3409	58	2009	W
2905_1700_S474_	2014	D	25.04.2014	S474	12.8	17.5	Fam-36_2009_S474	474	3410	36	2009	H
2905_1700_S475_	2014	D	25.04.2014	S475	14.4	25.5	Fam-84_2010_S475	475	3411	84	2010	W
2905_1700_S476_	2014	D	25.04.2014	S476	13.4	19.5	Fam-44_2009_S476	476	3412	44	2009	H
2905_1700_S477_	2014	D	25.04.2014	S477	12.3	16	Fam-38_2009_S477	477	3413	38	2009	H

2905_1700_S478_	2014	D	25.04.2014	S478	12.8	16.5	Fam-53_2009_S478	478	3414	53	2009	W
2905_1700_S479_	2014	D	25.04.2014	S479	13.9	21.5	Fam-53_2009_S479	479	3415	53	2009	W
2905_1700_S480_	2014	D	25.04.2014	S480	13.8	21.5	Fam-51_2009_S480	480	3416	51	2009	W
2906_1700_S481_	2014	D	25.04.2014	S481	14.8	25.5	Fam-40_2009_S481	481	3417	40	2009	H
2906_1700_S482_	2014	D	25.04.2014	S482	13.4	20	Fam-56_2009_S482	482	3418	56	2009	W
2906_1700_S483_	2014	D	25.04.2014	S483	15.5	27	Fam-38_2009_S483	483	3419	38	2009	H
2906_1700_S484_	2014	D	25.04.2014	S484	13.5	19.5	Fam-58_2009_S484	484	3420	58	2009	W
2906_1700_S485_	2014	D	25.04.2014	S485	13.1	17.5	Fam-50_2009_S485	485	3421	50	2009	H
2906_1700_S486_	2014	D	25.04.2014	S486	14.8	26	Fam-48_2009_S486	486	3422	48	2009	H
2906_1700_S487_	2014	D	25.04.2014	S487	12.8	18	Fam-42_2009_S487	487	3423	42	2009	H
2906_1700_S488_	2014	D	25.04.2014	S488	14.1	23.5	Fam-31_2009_S488	488	3424	31	2009	F
2906_1700_S489_	2014	D	25.04.2014	S489	15	28.5	Fam-58_2009_S489	489	3425	58	2009	W
2906_1700_S490_	2014	D	25.04.2014	S490	15	28	Fam-45_2009_S490	490	3426	45	2009	F
2906_1700_S491_	2014	D	25.04.2014	S491	14	23.5	Fam-53_2009_S491	491	3427	53	2009	W

2906_1700_S492_	2014	D	25.04.2014	S492	14.1	23.5	Fam-51_2009_	S492	492	3428	51	2009	W
2906_1700_S493_	2014	D	25.04.2014	S493	13.7	20.5	Fam-52_2009_	S493	493	3429	52	2009	W
2906_1700_S494_	2014	D	25.04.2014	S494	14	23.5	Fam-67_2010_	S494	494	3430	67	2010	F
2906_1700_S495_	2014	D	25.04.2014	S495	13.5	20	Fam-58_2009_	S495	495	3431	58	2009	W
2906_1700_S496_	2014	D	25.04.2014	S496	14.1	22	Fam-38_2009_	S496	496	3432	38	2009	H
2906_1700_S497_	2014	D	25.04.2014	S497	13.1	19	Fam-35_2009_	S497	497	3433	35	2009	F
2906_1700_S498_	2014	D	25.04.2014	S498	13.4	20	Fam-51_2009_	S498	498	3434	51	2009	W
2906_1700_S499_	2014	D	25.04.2014	S499	15.9	29.5	Fam-47_2009_	S499	499	3435	47	2009	F
2906_1700_S500_	2014	D	25.04.2014	S500	14.9	27	Fam-31_2009_	S500	500	3436	31	2009	F
2906_1700_S501_	2014	D	25.04.2014	S501	15.5	28.5	Fam-50_2009_	S501	501	3437	50	2009	H
2906_1700_S502_	2014	D	25.04.2014	S502	13	18.5	Fam-51_2009_	S502	502	3438	51	2009	W
2906_1700_S503_	2014	D	25.04.2014	S503	14.5	25.5	Fam-58_2009_	S503	503	3439	58	2009	W
2906_1700_S504_	2014	D	25.04.2014	S504	13.8	23.5	Fam-47_2009_	S504	504	3440	47	2009	F
2906_1700_S505_	2014	D	25.04.2014	S505	13.8	20.5	Fam-50_2009_	S505	505	3441	50	2009	H

2906_1700_S506_	2014	D	25.04.2014	S506	13.8	23.5	Fam-37_2009_	S506	506	3442	37	2009	F
2906_1700_S507_	2014	D	25.04.2014	S507	15.3	31.5	Fam-38_2009_	S507	507	3443	38	2009	H
2906_1700_S508_	2014	D	25.04.2014	S508	13	19	Fam-44_2009_	S508	508	3444	44	2009	H
2906_1700_S509_	2014	D	25.04.2014	S509	14.4	23	Fam-50_2009_	S509	509	3445	50	2009	H
2906_1700_S510_	2014	D	25.04.2014	S510	13	18	Fam-62_1_201	S510	510	3446	62	2010	H
2906_1700_S511_	2014	D	25.04.2014	S511	14.9	27.5	Fam-75_2010_	S511	511	3447	75	2010	F
2906_1700_S512_	2014	D	25.04.2014	S512	13.9	24.5	Fam-80_2010_	S512	512	3448	80	2010	H
2906_1700_S513_	2014	D	25.04.2014	S513	13.7	23	Fam-47_2009_	S513	513	3449	47	2009	F
2906_1700_S514_	2014	D	25.04.2014	S514	13.8	21	Fam-53_2009_	S514	514	3450	53	2009	W
2906_1700_S515_	2014	D	25.04.2014	S515	15.5	30.5	Fam-33_2009_	S515	515	3451	33	2009	F
2906_1700_S516_	2014	D	25.04.2014	S516	14.8	26.5	Fam-47_2009_	S516	516	3452	47	2009	F
2906_1700_S517_	2014	D	25.04.2014	S517	14.8	28	Fam-39_2009_	S517	517	3453	39	2009	F
2906_1700_S518_	2014	D	25.04.2014	S518	14	22	Fam-51_2009_	S518	518	3454	51	2009	W
2906_1700_S519_	2014	D	25.04.2014	S519	13.4	20.5	Fam-61_1_201	S519	519	3455	61	2010	F

2906_1700_S520_	2014	D	25.04.2014	S520	14.2	22.5	Fam-48_2009_	S520	520	3456	48	2009	H
2906_1700_S521_	2014	D	25.04.2014	S521	14.4	25.5	Fam-58_2009_	S521	521	3457	58	2009	W
2906_1700_S522_	2014	D	25.04.2014	S522	12.9	16.5	Fam-57_2009_	S522	522	3458	57	2009	W
2906_1700_S523_	2014	D	25.04.2014	S523	12.6	16.5	Fam-42_2009_	S523	523	3459	42	2009	H
2906_1700_S524_	2014	D	28.04.2014	S524	14.6	24	Fam-43_2009_	S524	524	3460	43	2009	F
2906_1700_S525_	2014	D	28.04.2014	S525	14.5	26	Fam-45_2009_	S525	525	3461	45	2009	F
2906_1700_S526_	2014	D	28.04.2014	S526	15	25	Fam-50_2009_	S526	526	3462	50	2009	H
2906_1700_S527_	2014	D	28.04.2014	S527	14.2	25	Fam-45_2009_	S527	527	3463	45	2009	F
2906_1700_S528_	2014	D	28.04.2014	S528	14.5	26	Fam-57_2009_	S528	528	3464	57	2009	W
2906_1700_S529_	2014	D	28.04.2014	S529	14.2	22	Fam-45_2009_	S529	529	3465	45	2009	F
2906_1700_S530_	2014	D	28.04.2014	S530	14.5	23.5	Fam-44_2009_	S530	530	3466	44	2009	H
2906_1700_S531_	2014	D	28.04.2014	S531	15.2	28	Fam-31_2009_	S531	531	3467	31	2009	F
2906_1700_S532_	2014	D	28.04.2014	S532	14	23	Fam-79_2010_	S532	532	3468	79	2010	F
2906_1700_S533_	2014	D	28.04.2014	S533	14.4	25.5	Fam-45_2009_	S533	533	3469	45	2009	F

2906_1700_S534_	2014	D	28.04.2014	S534	13.2	19	Fam-38_2009_	S534	534	3470	38	2009	H
2906_1700_S535_	2014	D	28.04.2014	S535	13.8	20	Fam-50_2009_	S535	535	3471	50	2009	H
2906_1700_S536_	2014	D	28.04.2014	S536	16	34.5	Fam-36_2009_	S536	536	3472	36	2009	H
2906_1700_S537_	2014	D	28.04.2014	S537	17.5	44	Fam-31_2009_	S537	537	3473	31	2009	F
2906_1700_S538_	2014	D	28.04.2014	S538	13.2	19	Fam-73_2010_	S538	538	3474	73	2010	F
2906_1700_S539_	2014	D	28.04.2014	S539	12.2	15	Fam-59_2009_	S539	539	3475	59	2009	W
2906_1700_S540_	2014	D	28.04.2014	S540	15	27	Fam-48_2009_	S540	540	3476	48	2009	H
2906_1700_S541_	2014	D	28.04.2014	S541	15	27.5	Fam-50_2009_	S541	541	3477	50	2009	H
2906_1700_S542_	2014	D	28.04.2014	S542	15.6	30.5	Fam-63_2010_	S542	542	3478	63	2010	F
2906_1700_S543_	2014	D	28.04.2014	S543	14.1	23	Fam-32_2009_	S543	543	3479	32	2009	H
2906_1700_S544_	2014	D	28.04.2014	S544	13.8	22.5	Fam-69_2010_	S544	544	3480	69	2010	F
2906_1700_S545_	2014	D	28.04.2014	S545	14	21	Fam-52_2009_	S545	545	3481	52	2009	W
2906_1700_S546_	2014	D	28.04.2014	S546	14.5	23.5	Fam-73_2010_	S546	546	3482	73	2010	F
2906_1700_S547_	2014	D	28.04.2014	S547	13.8	20	Fam-31_2009_	S547	547	3483	31	2009	F

2906_1700_S548_	2014	D	28.04.2014	S548	15.3	28.5	Fam-47_2009_S548	548	3484	47	2009	F
2906_1700_S549_	2014	D	28.04.2014	S549	13	17	Fam-59_2009_S549	549	3485	59	2009	W
2906_1700_S550_	2014	D	28.04.2014	S550	14.6	25.5	Fam-42_2009_S550	550	3486	42	2009	H
2906_1700_S551_	2014	D	28.04.2014	S551	15.5	33	Fam-31_2009_S551	551	3487	31	2009	F
2906_1700_S552_	2014	D	28.04.2014	S552	14.8	27.5	Fam-31_2009_S552	552	3488	31	2009	F
2906_1700_S553_	2014	D	28.04.2014	S553	14.3	24	Fam-35_2009_S553	553	3489	35	2009	F
2906_1700_S554_	2014	D	28.04.2014	S554	13	17.5	Fam-34_2009_S554	554	3490	34	2009	H
2906_1700_S555_	2014	D	28.04.2014	S555	13	18.5	Fam-42_2009_S555	555	3491	42	2009	H
2906_1700_S556_	2014	D	28.04.2014	S556	14.3	24	Fam-43_2009_S556	556	3492	43	2009	F
2906_1700_S557_	2014	D	28.04.2014	S557	14.9	27	Fam-69_2010_S557	557	3493	69	2010	F
2906_1700_S558_	2014	D	28.04.2014	S558	13.1	18	Fam-47_2009_S558	558	3494	47	2009	F
2906_1700_S559_	2014	D	28.04.2014	S559	12.3	14.5	Fam-59_2009_S559	559	3495	59	2009	W
2906_1700_S560_	2014	D	28.04.2014	S560	14.5	26	Fam-47_2009_S560	560	3496	47	2009	F
2907_1700_S561_	2014	D	28.04.2014	S561	14	20	Fam-59_2009_S561	561	3497	59	2009	W

2907_1700_S562_	2014	D	28.04.2014	S562	14.9	26	Fam-69_2010_	S562	562	3498	69	2010	F
2907_1700_S563_	2014	D	28.04.2014	S563	13.9	22	Fam-57_2009_	S563	563	3499	57	2009	W
2907_1700_S564_	2014	D	28.04.2014	S564	14.7	25.5	Fam-58_2009_	S564	564	3500	58	2009	W
2907_1701_S565_	2014	D	28.04.2014	S565	13.8	22	Fam-52_2009_	S565	565	3501	52	2009	W
2907_1701_S566_	2014	D	28.04.2014	S566	15	27	Fam-36_2009_	S566	566	3502	36	2009	H
2907_1701_S567_	2014	D	28.04.2014	S567	12.9	18	Fam-58_2009_	S567	567	3503	58	2009	W
2907_1701_S568_	2014	D	28.04.2014	S568	14.4	23	Fam-51_2009_	S568	568	3504	51	2009	W
2907_1701_S569_	2014	D	28.04.2014	S569	13.8	21	Fam-54_2009_	S569	569	3505	54	2009	W
2907_1701_S571_	2014	D	28.04.2014	S571	14.7	25.5	Fam-40_2009_	S571	571	3506	40	2009	H
2907_1701_S572_	2014	D	28.04.2014	S572	12.8	18	Fam-36_2009_	S572	572	3507	36	2009	H
2907_1701_S573_	2014	D	28.04.2014	S573	15	26.5	Fam-40_2009_	S573	573	3508	40	2009	H
2907_1701_S575_	2014	D	28.04.2014	S575	14.5	21.5	Fam-68_2010_	S575	575	3509	68	2010	H
2907_1701_S576_	2014	D	28.04.2014	S576	14	22	Fam-44_2009_	S576	576	3510	44	2009	H
2907_1701_S577_	2014	D	28.04.2014	S577	15.4	31	Fam-47_2009_	S577	577	3511	47	2009	F

2907_1701_S578_	2014	D	28.04.2014	S578	14	21	Fam-51_2009_	S578	578	3512	51	2009	W
2907_1701_S579_	2014	D	28.04.2014	S579	14.2	23.5	Fam-85_2010_	S579	579	3513	85	2010	W
2907_1701_S581_	2014	D	28.04.2014	S581	15.5	31	Fam-79_2010_	S581	581	3514	79	2010	F
2907_1701_S582_	2014	D	28.04.2014	S582	13.2	18.5	Fam-36_2009_	S582	582	3515	36	2009	H
2907_1701_S583_	2014	D	28.04.2014	S583	12.6	17	Fam-38_2009_	S583	583	3516	38	2009	H
2907_1701_S584_	2014	D	28.04.2014	S584	16.5	39	Fam-65_2010_	S584	584	3517	65	2010	F
2907_1701_S585_	2014	D	28.04.2014	S585	13.8	19.5	Fam-50_2009_	S585	585	3518	50	2009	H
2907_1701_S586_	2014	D	28.04.2014	S586	13.3	18	Fam-51_2009_	S586	586	3519	51	2009	W
2907_1701_S587_	2014	D	28.04.2014	S587	15.1	28	Fam-45_2009_	S587	587	3520	45	2009	F
2907_1701_S588_	2014	D	28.04.2014	S588	13.2	19	Fam-57_2009_	S588	588	3521	57	2009	W
2907_1701_S589_	2014	D	28.04.2014	S589	15.4	28	Fam-43_2009_	S589	589	3522	43	2009	F
2907_1701_S590_	2014	D	28.04.2014	S590	13.5	18.5	Fam-35_2009_	S590	590	3523	35	2009	F
2907_1701_S591_	2014	D	28.04.2014	S591	13.6	20.5	Fam-51_2009_	S591	591	3524	51	2009	W
2907_1701_S592_	2014	D	28.04.2014	S592	12.7	16.5	Fam-68_2010_	S592	592	3525	68	2010	H

2907_1701_S593_	2014	D	28.04.2014	S593	14	22.5	Fam-76_2010_	S593	593	3526	76	2010	H
2907_1701_S594_	2014	D	28.04.2014	S594	14.8	27	Fam-61_1_201	S594	594	3527	61	2010	F
2907_1701_S595_	2014	D	28.04.2014	S595	14.2	22	Fam-52_2009_	S595	595	3528	52	2009	W
2907_1701_S596_	2014	D	28.04.2014	S596	13.5	20.5	Fam-38_2009_	S596	596	3529	38	2009	H
2907_1701_S597_	2014	D	28.04.2014	S597	14.2	22	Fam-35_2009_	S597	597	3530	35	2009	F
2907_1701_S598_	2014	D	28.04.2014	S598	14	22	Fam-71_2010_	S598	598	3531	71	2010	F
2907_1701_S599_	2014	D	28.04.2014	S599	12.9	16	Fam-58_2009_	S599	599	3532	58	2009	W
2907_1701_S600_	2014	D	28.04.2014	S600	14	21.5	Fam-59_2009_	S600	600	3533	59	2009	W
2907_1701_S601_	2014	D	28.04.2014	S601	13.9	24	Fam-41_2009_	S601	601	3534	41	2009	F
2907_1701_S602_	2014	D	28.04.2014	S602	15	27.5	Fam-40_2009_	S602	602	3535	40	2009	H
2907_1701_S603_	2014	D	28.04.2014	S603	14	21.5	Fam-58_2009_	S603	603	3536	58	2009	W
2907_1701_S604_	2014	D	28.04.2014	S604	13	17.5	Fam-85_2010_	S604	604	3537	85	2010	W
2907_1701_S605_	2014	D	28.04.2014	S605	14.2	22	Fam-57_2009_	S605	605	3538	57	2009	W
2907_1701_S606_	2014	D	28.04.2014	S606	14.1	23	Fam-85_2010_	S606	606	3539	85	2010	W

2907_1701_S607_	2014	D	28.04.2014	S607	13	18	Fam-38_2009_	S607	607	3540	38	2009	H
2907_1701_S608_	2014	D	28.04.2014	S608	14.1	23	Fam-38_2009_	S608	608	3541	38	2009	H
2907_1701_S609_	2014	D	28.04.2014	S609	14.7	27	Fam-33_2009_	S609	609	3542	33	2009	F
2907_1701_S610_	2014	D	28.04.2014	S610	15	26.5	Fam-36_2009_	S610	610	3543	36	2009	H
2907_1701_S611_	2014	D	28.04.2014	S611	14.4	25.5	Fam-39_2009_	S611	611	3544	39	2009	F
2907_1701_S612_	2014	D	28.04.2014	S612	14.9	24.5	Fam-58_2009_	S612	612	3545	58	2009	W
2907_1701_S613_	2014	D	28.04.2014	S613	13.5	20	Fam-35_2009_	S613	613	3546	35	2009	F
2907_1701_S614_	2014	D	28.04.2014	S614	14.6	27	Fam-31_2009_	S614	614	3547	31	2009	F
2907_1701_S615_	2014	D	28.04.2014	S615	14.8	25	Fam-40_2009_	S615	615	3548	40	2009	H
2907_1701_S616_	2014	D	28.04.2014	S616	13.7	19.5	Fam-35_2009_	S616	616	3549	35	2009	F
2907_1701_S617_	2014	D	28.04.2014	S617	16.5	36.5	Fam-57_2009_	S617	617	3550	57	2009	W
2907_1701_S618_	2014	D	28.04.2014	S618	15	28.5	Fam-36_2009_	S618	618	3551	36	2009	H
2907_1701_S619_	2014	D	28.04.2014	S619	13.5	20	Fam-38_2009_	S619	619	3552	38	2009	H
2907_1701_S620_	2014	D	28.04.2014	S620	12.9	18.5	Fam-90_2010_	S620	620	3553	90	2010	W

2907_1701_S621_	2014	D	28.04.2014	S621	16	30.5	Fam-71_2010_S621	621	3554	71	2010 F
2907_1701_S622_	2014	D	28.04.2014	S622	13.2	19	Fam-54_2009_S622	622	3555	54	2009 W
2907_1701_S623_	2014	D	28.04.2014	S623	12.5	15.5	Fam-85_2010_S623	623	3556	85	2010 W
2907_1701_S624_	2014	D	28.04.2014	S624	16	34.5	Fam-66_2010_S624	624	3557	66	2010 H
2907_1701_S625_	2014	D	28.04.2014	S625	14	22.5	Fam-58_2009_S625	625	3558	58	2009 W
2907_1701_S626_	2014	D	28.04.2014	S626	13	16	Fam-50_2009_S626	626	3559	50	2009 H
2907_1701_S627_	2014	D	28.04.2014	S627	14.7	26	Fam-39_2009_S627	627	3560	39	2009 F
2907_1701_S628_	2014	D	28.04.2014	S628	13.5	19.5	Fam-36_2009_S628	628	3561	36	2009 H
2907_1701_S629_	2014	D	28.04.2014	S629	12.5	15	Fam-35_2009_S629	629	3562	35	2009 F
2907_1701_S630_	2014	D	28.04.2014	S630	16.9	40	Fam-77_2010_S630	630	3563	77	2010 F
2907_1701_S631_	2014	D	28.04.2014	S631	15.3	27.5	Fam-57_2009_S631	631	3564	57	2009 W
2907_1701_S632_	2014	D	28.04.2014	S632	14.5	26	Fam-36_2009_S632	632	3565	36	2009 H
2907_1701_S633_	2014	D	28.04.2014	S633	14.3	25.5	Fam-43_2009_S633	633	3566	43	2009 F
2907_1701_S634_	2014	D	28.04.2014	S634	15.8	30.5	Fam-38_2009_S634	634	3567	38	2009 H

2907_1701_S635_	2014	D	28.04.2014	S635	14	21	Fam-40_2009_S635	635	3568	40	2009	H
2907_1701_S636_	2014	D	28.04.2014	S636	15.5	31	Fam-38_2009_S636	636	3569	38	2009	H
2907_1701_S638_	2014	D	28.04.2014	S638	15.1	25.5	Fam-51_2009_S638	638	3570	51	2009	W
2907_1701_S639_	2014	D	28.04.2014	S639	13.9	22	Fam-45_2009_S639	639	3571	45	2009	F
2907_1701_S640_	2014	D	28.04.2014	S640	14.2	23	Fam-38_2009_S640	640	3572	38	2009	H
2908_1701_S641_	2014	D	28.04.2014	S641	13.7	21	Fam-50_2009_S641	641	3573	50	2009	H
2908_1701_S642_	2014	D	28.04.2014	S642	12.4	17	Fam-58_2009_S642	642	3574	58	2009	W
2908_1701_S643_	2014	D	28.04.2014	S643	13.2	18	Fam-76_2010_S643	643	3575	76	2010	H
2908_1701_S644_	2014	D	28.04.2014	S644	14.1	23	Fam-50_2009_S644	644	3576	50	2009	H
2908_1701_S645_	2014	D	28.04.2014	S645	15	27	Fam-51_2009_S645	645	3577	51	2009	W
2908_1701_S646_	2014	D	28.04.2014	S646	12.9	17	Fam-51_2009_S646	646	3578	51	2009	W
2908_1701_S647_	2014	D	28.04.2014	S647	14.7	24.5	Fam-40_2009_S647	647	3579	40	2009	H
2908_1701_S648_	2014	D	28.04.2014	S648	13.5	19.5	Fam-40_2009_S648	648	3580	40	2009	H
2908_1701_S649_	2014	D	28.04.2014	S649	14.3	24	Fam-65_2010_S649	649	3581	65	2010	F

2908_1701_S650_	2014	D	28.04.2014	S650	13.2	18.5	Fam-49_2009_	S650	650	3582	49	2009 F
2908_1701_S651_	2014	D	28.04.2014	S651	14.3	23	Fam-58_2009_	S651	651	3583	58	2009 W
2908_1701_S652_	2014	T	28.04.2014	S652	15	25.5	Family-50_200	S652	652	3584	50	2009 H
2908_1701_S653_	2014	D	28.04.2014	S653	14.5	25.5	Fam-89_2010_	S653	653	3585	89	2010 W
2908_1701_S654_	2014	D	28.04.2014	S654	14.5	23.5	Fam-57_2009_	S654	654	3586	57	2009 W
2908_1701_S655_	2014	D	28.04.2014	S655	14	23	Fam-58_2009_	S655	655	3587	58	2009 W
2908_1701_S656_	2014	D	28.04.2014	S656	13.2	19	Fam-51_2009_	S656	656	3588	51	2009 W
2908_1701_S657_	2014	D	28.04.2014	S657	13.3	20	Fam-83_2010_	S657	657	3589	83	2010 W
2908_1703_S658_	2014	D	28.04.2014	S658	14	21.5	Fam-57_2009_	S658	658	3590	57	2009 W
2908_1703_S659_	2014	D	28.04.2014	S659	13.5	20	Fam-50_2009_	S659	659	3591	50	2009 H
2908_1703_S660_	2014	D	28.04.2014	S660	15.2	28	Fam-40_2009_	S660	660	3592	40	2009 H
2908_1703_S661_	2014	D	28.04.2014	S661	13.5	21	Fam-31_2009_	S661	661	3593	31	2009 F
2908_1703_S662_	2014	D	28.04.2014	S662	13.5	21	Fam-31_2009_	S662	662	3594	31	2009 F
2908_1703_S663_	2014	D	28.04.2014	S663	12.7	16	Fam-59_2009_	S663	663	3595	59	2009 W

2908_1703_S664_	2014	D	28.04.2014	S664	14.9	28	Fam-74_2010_	S664	664	3596	74	2010	H
2908_1703_S665_	2014	D	28.04.2014	S665	13.7	20.5	Fam-62_1_201	S665	665	3597	62	2010	H
2908_1703_S666_	2014	D	28.04.2014	S666	14.6	25.5	Fam-58_2009_	S666	666	3598	58	2009	W
2908_1703_S667_	2014	D	28.04.2014	S667	15.1	27	Fam-31_2009_	S667	667	3599	31	2009	F
2908_1703_S668_	2014	D	28.04.2014	S668	15.9	31	Fam-47_2009_	S668	668	3600	47	2009	F
2908_1703_S669_	2014	D	28.04.2014	S669	13.6	21	Fam-41_2009_	S669	669	3601	41	2009	F
2908_1703_S670_	2014	D	28.04.2014	S670	14	22	Fam-76_2010_	S670	670	3602	76	2010	H
2908_1703_S671_	2014	D	28.04.2014	S671	12.8	17.5	Fam-85_2010_	S671	671	3603	85	2010	W
2908_1703_S672_	2014	D	28.04.2014	S672	14.6	23.5	Fam-50_2009_	S672	672	3604	50	2009	H
2908_1703_S673_	2014	D	28.04.2014	S673	14.7	27	Fam-69_2010_	S673	673	3605	69	2010	F
2908_1703_S674_	2014	D	28.04.2014	S674	15.5	28.5	Fam-50_2009_	S674	674	3606	50	2009	H
2908_1703_S675_	2014	D	28.04.2014	S675	16.7	37	Fam-47_2009_	S675	675	3607	47	2009	F
2908_1703_S676_	2014	D	28.04.2014	S676	13.3	20	Fam-37_2009_	S676	676	3608	37	2009	F
2908_1703_S677_	2014	D	28.04.2014	S677	13.9	20.5	Fam-35_2009_	S677	677	3609	35	2009	F

2908_1703_S678_	2014	D	28.04.2014	S678	15.7	29.5	Fam-51_2009_	S678	678	3610	51	2009	W
2908_1703_S679_	2014	D	28.04.2014	S679	13.2	17	Fam-51_2009_	S679	679	3611	51	2009	W
2908_1703_S680_	2014	D	28.04.2014	S680	14.7	27.5	Fam-31_2009_	S680	680	3612	31	2009	F
2908_1703_S681_	2014	D	28.04.2014	S681	15.1	26.5	Fam-55_2009_	S681	681	3613	55	2009	W
2908_1703_S682_	2014	D	28.04.2014	S682	14.5	24.5	Fam-59_2009_	S682	682	3614	59	2009	W
2908_1703_S683_	2014	D	28.04.2014	S683	12.8	17.5	Fam-58_2009_	S683	683	3615	58	2009	W
2908_1703_S684_	2014	D	28.04.2014	S684	12.8	17	Fam-76_2010_	S684	684	3616	76	2010	H
2908_1703_S685_	2014	D	28.04.2014	S685	15.6	28	Fam-40_2009_	S685	685	3617	40	2009	H
2908_1703_S686_	2014	D	28.04.2014	S686	13.6	20.5	Fam-55_2009_	S686	686	3618	55	2009	W
2908_1703_S687_	2014	D	28.04.2014	S687	13.6	20.5	Fam-54_2009_	S687	687	3619	54	2009	W
2908_1703_S688_	2014	D	28.04.2014	S688	15.1	31	Fam-13_2007_	S688	688	3620	13	2007	F
2908_1703_S691_	2014	D	28.04.2014	S691	14	23	Fam-64_2010_	S691	691	3621	64	2010	H
2908_1703_S692_	2014	D	28.04.2014	S692	14.4	22	Fam-43_2009_	S692	692	3622	43	2009	F
2908_1703_S693_	2014	D	28.04.2014	S693	14.6	27	Fam-72_2010_	S693	693	3623	72	2010	H

2908_1703_S694_	2014	D	28.04.2014	S694	13.5	20.5	Fam-72_2010_	S694	694	3624	72	2010	H
2908_1703_S695_	2014	D	28.04.2014	S695	15.5	29	Fam-54_2009_	S695	695	3625	54	2009	W
2908_1703_S696_	2014	D	28.04.2014	S696	12.2	15.5	Fam-44_2009_	S696	696	3626	44	2009	H
2908_1703_S697_	2014	D	28.04.2014	S697	13.2	19	Fam-58_2009_	S697	697	3627	58	2009	W
2908_1703_S698_	2014	D	28.04.2014	S698	14	21.5	Fam-51_2009_	S698	698	3628	51	2009	W
2908_1703_S699_	2014	D	28.04.2014	S699	12.6	16.5	Fam-76_2010_	S699	699	3629	76	2010	H
2908_1703_S700_	2014	D	30.04.2014	S700	13.9	22	Fam-84_2010_	S700	700	3630	84	2010	W
2908_1703_S701_	2014	D	30.04.2014	S701	14.7	25	Fam-45_2009_	S701	701	3631	45	2009	F
2908_1703_S702_	2014	D	30.04.2014	S702	14.2	14.5	Fam-87_2010_	S702	702	3632	87	2010	W
2908_1703_S703_	2014	D	30.04.2014	S703	14.2	25	Fam-37_2009_	S703	703	3633	37	2009	F
2908_1703_S704_	2014	D	30.04.2014	S704	15.4	28	Fam-52_2009_	S704	704	3634	52	2009	W
2908_1703_S705_	2014	T	30.04.2014	S705	13.7	19	Family-39_200	S705	705	3635	39	2009	F
2908_1703_S706_	2014	D	30.04.2014	S706	15.2	28	Fam-36_2009_	S706	706	3636	36	2009	H
2908_1703_S707_	2014	D	30.04.2014	S707	14	22.5	Fam-58_2009_	S707	707	3637	58	2009	W

2908_1703_S708_	2014	D	30.04.2014	S708	15	25.5	Fam-50_2009_	S708	708	3638	50	2009	H
2908_1703_S709_	2014	D	30.04.2014	S709	15.6	30	Fam-39_2009_	S709	709	3639	39	2009	F
2908_1703_S710_	2014	D	30.04.2014	S710	13.2	21	Fam-65_2010_	S710	710	3640	65	2010	F
2908_1703_S711_	2014	D	30.04.2014	S711	13.9	21	Fam-65_2010_	S711	711	3641	65	2010	F
2908_1703_S712_	2014	D	30.04.2014	S712	12.8	19	Fam-41_2009_	S712	712	3642	41	2009	F
2908_1703_S713_	2014	D	30.04.2014	S713	14.4	24	Fam-65_2010_	S713	713	3643	65	2010	F
2908_1703_S714_	2014	D	30.04.2014	S714	13.9	22	Fam-59_2009_	S714	714	3644	59	2009	W
2908_1703_S715_	2014	D	30.04.2014	S715	15.9	32.5	Fam-64_2010_	S715	715	3645	64	2010	H
2908_1703_S716_	2014	T	30.04.2014	S716	14.7	23	Family-39_200	S716	716	3646	39	2009	F
2908_1703_S717_	2014	D	30.04.2014	S717	13.8	19.5	Fam-52_2009_	S717	717	3647	52	2009	W
2908_1703_S718_	2014	D	30.04.2014	S718	13	17.5	Fam-62_1_201	S718	718	3648	62	2010	H
2908_1703_S719_	2014	D	30.04.2014	S719	15	27	Fam-40_2009_	S719	719	3649	40	2009	H
2908_1703_S720_	2014	D	30.04.2014	S720	15.6	33	Fam-35_2009_	S720	720	3650	35	2009	F
2909_1703_S721_	2014	D	30.04.2014	S721	12.9	17.5	Fam-52_2009_	S721	721	3651	52	2009	W

2909_1703_S722_	2014	D	30.04.2014	S722	14.5	26.5	Fam-45_2009_S722	722	3652	45	2009	F
2909_1703_S723_	2014	D	30.04.2014	S723	13.9	21	Fam-83_2010_S723	723	3653	83	2010	W
2909_1703_S724_	2014	D	30.04.2014	S724	14	23.5	Fam-60_2009_S724	724	3654	60	2009	W
2909_1703_S725_	2014	D	30.04.2014	S725	14.6	27	Fam-42_2009_S725	725	3655	42	2009	H
2909_1703_S727_	2014	D	30.04.2014	S727	13.7	21	Fam-54_2009_S727	727	3656	54	2009	W
2909_1703_S728_	2014	D	30.04.2014	S728	13	18.5	Fam-80_2010_S728	728	3657	80	2010	H
2909_1703_S729_	2014	D	30.04.2014	S729	13.4	19.5	Fam-89_2010_S729	729	3658	89	2010	W
2909_1703_S730_	2014	D	30.04.2014	S730	14.5	24	Fam-58_2009_S730	730	3659	58	2009	W
2909_1703_S731_	2014	D	30.04.2014	S731	15.4	28	Fam-75_2010_S731	731	3660	75	2010	F
2909_1703_S732_	2014	D	30.04.2014	S732	13.8	20.5	Fam-54_2009_S732	732	3661	54	2009	W
2909_1703_S733_	2014	D	30.04.2014	S733	13.3	20	Fam-85_2010_S733	733	3662	85	2010	W
2909_1703_S734_	2014	D	30.04.2014	S734	13.7	22	Fam-38_2009_S734	734	3663	38	2009	H
2909_1703_S735_	2014	D	30.04.2014	S735	15.2	33.5	Fam-33_2009_S735	735	3664	33	2009	F
2909_1703_S737_	2014	D	30.04.2014	S737	14.2	23.5	Fam-51_2009_S737	737	3665	51	2009	W

2909_1703_S739_	2014	D	30.04.2014	S739	13.9	21.5	Fam-57_2009_	S739	739	3666	57	2009	W
2909_1703_S740_	2014	D	30.04.2014	S740	13.9	23	Fam-41_2009_	S740	740	3667	41	2009	F
2909_1703_S741_	2014	D	30.04.2014	S741	14.6	25	Fam-39_2009_	S741	741	3668	39	2009	F
2909_1703_S742_	2014	D	30.04.2014	S742	14.5	25.5	Fam-48_2009_	S742	742	3669	48	2009	H
2909_1703_S743_	2014	D	30.04.2014	S743	13.6	20.5	Fam-62_1_201	S743	743	3670	62	2010	H
2909_1703_S744_	2014	D	30.04.2014	S744	13.6	19.5	Fam-54_2009_	S744	744	3671	54	2009	W
2909_1703_S745_	2014	D	30.04.2014	S745	14	21.5	Fam-57_2009_	S745	745	3672	57	2009	W
2909_1703_S746_	2014	D	30.04.2014	S746	14.3	24.5	Fam-58_2009_	S746	746	3673	58	2009	W
2909_1703_S747_	2014	D	30.04.2014	S747	13.6	21	Fam-47_2009_	S747	747	3674	47	2009	F
2909_1703_S748_	2014	D	02.05.2014	S748	13.4	19	Fam-71_2010_	S748	748	3675	71	2010	F
2909_1703_S749_	2014	D	02.05.2014	S749	15	27.5	Fam-45_2009_	S749	749	3676	45	2009	F
2909_1703_S750_	2014	D	02.05.2014	S750	13.5	21	Fam-42_2009_	S750	750	3677	42	2009	H
2909_1703_S751_	2014	T	02.05.2014	S751	14	19	Family-50_200	S751	751	3678	50	2009	H
2909_1704_S752_	2014	D	02.05.2014	S752	15	28	Fam-43_2009_	S752	752	3679	43	2009	F

2909_1704_S753_	2014	D	02.05.2014	S753	14	21.5	Fam-39_2009_	S753	753	3680	39	2009	F
2909_1704_S754_	2014	D	02.05.2014	S754	14.4	23.5	Fam-43_2009_	S754	754	3681	43	2009	F
2909_1704_S755_	2014	D	02.05.2014	S755	16.2	30.5	Fam-65_2010_	S755	755	3682	65	2010	F
2909_1704_S756_	2014	D	02.05.2014	S756	13.5	20	Fam-52_2009_	S756	756	3683	52	2009	W
2909_1704_S757_	2014	D	02.05.2014	S757	14.9	25.5	Fam-38_2009_	S757	757	3684	38	2009	H
2909_1704_S758_	2014	D	02.05.2014	S758	13.8	21	Fam-62_1_201	S758	758	3685	62	2010	H
2909_1704_S759_	2014	D	02.05.2014	S759	12.6	15.5	Fam-32_2009_	S759	759	3686	32	2009	H
2909_1704_S760_	2014	D	02.05.2014	S760	13.6	20.5	Fam-58_2009_	S760	760	3687	58	2009	W
2909_1704_S761_	2014	D	02.05.2014	S761	13.3	18.5	Fam-76_2010_	S761	761	3688	76	2010	H
2909_1704_S762_	2014	D	02.05.2014	S762	13.2	17	Fam-35_2009_	S762	762	3689	35	2009	F
2909_1704_S764_	2014	D	02.05.2014	S764	13.8	25.5	Fam-51_2009_	S764	764	3690	51	2009	W
2909_1704_S765_	2014	D	02.05.2014	S765	14.9	29	Fam-50_2009_	S765	765	3691	50	2009	H
2909_1704_S766_	2014	D	02.05.2014	S766	12.6	16.5	Fam-76_2010_	S766	766	3692	76	2010	H
2909_1704_S767_	2014	D	02.05.2014	S767	12.7	19	Fam-70_2010_	S767	767	3693	70	2010	H

2909_1704_S769_	2014	D	02.05.2014	S769	14.3	23	Fam-57_2009_	S769	769	3694	57	2009	W
2909_1704_S770_	2014	D	02.05.2014	S770	13.8	21	Fam-38_2009_	S770	770	3695	38	2009	H
2909_1704_S771_	2014	D	02.05.2014	S771	13.9	20.5	Fam-40_2009_	S771	771	3696	40	2009	H
2909_1704_S772_	2014	D	02.05.2014	S772	17.6	38	Fam-77_2010_	S772	772	3697	77	2010	F
2909_1704_S773_	2014	D	02.05.2014	S773	12.6	16	Fam-32_2009_	S773	773	3698	32	2009	H
2909_1704_S774_	2014	D	02.05.2014	S774	14.3	23	Fam-52_2009_	S774	774	3699	52	2009	W
2909_1704_S775_	2014	D	02.05.2014	S775	13.6	19	Fam-52_2009_	S775	775	3700	52	2009	W
2909_1704_S776_	2014	D	02.05.2014	S776	14	20	Fam-51_2009_	S776	776	3701	51	2009	W
2909_1704_S777_	2014	D	02.05.2014	S777	13	18	Fam-59_2009_	S777	777	3702	59	2009	W
2909_1704_S778_	2014	D	02.05.2014	S778	13.8	24.5	Fam-42_2009_	S778	778	3703	42	2009	H
2909_1704_S779_	2014	D	02.05.2014	S779	14.4	23	Fam-47_2009_	S779	779	3704	47	2009	F
2909_1704_S780_	2014	D	02.05.2014	S780	12.5	18	Fam-54_2009_	S780	780	3705	54	2009	W
2909_1704_S782_	2014	D	05.05.2014	S782	13.4	18	Fam-53_2009_	S782	782	3706	53	2009	W
2909_1704_S783_	2014	D	05.05.2014	S783	13.6	19.5	Fam-35_2009_	S783	783	3707	35	2009	F

2909_1704_S784_	2014	D	05.05.2014	S784	14	23	Fam-36_2009_	S784	784	3708	36	2009	H
2909_1704_S786_	2014	D	05.05.2014	S786	13.5	21	Fam-38_2009_	S786	786	3709	38	2009	H
2909_1704_S787_	2014	D	05.05.2014	S787	14.5	24.5	Fam-62_1_201	S787	787	3710	62	2010	H
2909_1704_S788_	2014	D	05.05.2014	S788	14.4	24.5	Fam-87_2010_	S788	788	3711	87	2010	W
2909_1704_S789_	2014	D	05.05.2014	S789	13.7	20.5	Fam-38_2009_	S789	789	3712	38	2009	H
2909_1704_S790_	2014	D	05.05.2014	S790	15	26.5	Fam-31_2009_	S790	790	3713	31	2009	F
2909_1704_S792_	2014	D	05.05.2014	S792	14.2	23.5	Fam-38_2009_	S792	792	3714	38	2009	H
2909_1704_S793_	2014	D	05.05.2014	S793	13.4	19	Fam-48_2009_	S793	793	3715	48	2009	H
2909_1704_S794_	2014	D	05.05.2014	S794	12.2	14.5	Fam-59_2009_	S794	794	3716	59	2009	W
2909_1704_S795_	2014	D	05.05.2014	S795	14.2	23.5	Fam-58_2009_	S795	795	3717	58	2009	W
2909_1704_S796_	2014	D	05.05.2014	S796	13	17	Fam-38_2009_	S796	796	3718	38	2009	H
2909_1704_S797_	2014	D	05.05.2014	S797	12.2	14	Fam-71_2010_	S797	797	3719	71	2010	F
2909_1704_S798_	2014	D	05.05.2014	S798	14.8	24.5	Fam-89_2010_	S798	798	3720	89	2010	W
2909_1704_S799_	2014	D	05.05.2014	S799	12.5	16	Fam-38_2009_	S799	799	3721	38	2009	H

2909_1704_S800_	2014	D	05.05.2014	S800	13	17	Fam-51_2009_	S800	800	3722	51	2009	W
2911_1704_S802_	2014	D	05.05.2014	S802	14.4	24.5	Fam-38_2009_	S802	802	3723	38	2009	H
2911_1704_S803_	2014	D	05.05.2014	S803	14	21.5	Fam-51_2009_	S803	803	3724	51	2009	W
2911_1704_S804_	2014	D	05.05.2014	S804	14.5	24	Fam-42_2009_	S804	804	3725	42	2009	H
2911_1704_S806_	2014	D	05.05.2014	S806	14.2	24	Fam-62_1_201	S806	806	3726	62	2010	H
2911_1704_S807_	2014	D	05.05.2014	S807	14.2	22	Fam-40_2009_	S807	807	3727	40	2009	H
2911_1704_S808_	2014	D	05.05.2014	S808	13.5	19.5	Fam-54_2009_	S808	808	3728	54	2009	W
2911_1704_S809_	2014	D	05.05.2014	S809	14.5	24.5	Fam-40_2009_	S809	809	3729	40	2009	H
2911_1704_S810_	2014	D	05.05.2014	S810	12.3	15	Fam-59_2009_	S810	810	3730	59	2009	W
2911_1704_S811_	2014	D	05.05.2014	S811	14.2	21.5	Fam-57_2009_	S811	811	3731	57	2009	W
2911_1704_S812_	2014	D	05.05.2014	S812	14	20.5	Fam-83_2010_	S812	812	3732	83	2010	W
2911_1704_S813_	2014	D	05.05.2014	S813	14.4	24	Fam-79_2010_	S813	813	3733	79	2010	F
2911_1704_S814_	2014	D	05.05.2014	S814	14	22.5	Fam-52_2009_	S814	814	3734	52	2009	W
2911_1704_S815_	2014	D	05.05.2014	S815	14.3	23	Fam-62_1_201	S815	815	3735	62	2010	H

2911_1704_S816_	2014	D	05.05.2014	S816	13.5	18.5	Fam-54_2009_	S816	816	3736	54	2009	W
2911_1704_S817_	2014	D	05.05.2014	S817	14.5	26	Fam-45_2009_	S817	817	3737	45	2009	F
2911_1704_S818_	2014	D	05.05.2014	S818	14.7	25.5	Fam-68_2010_	S818	818	3738	68	2010	H
2911_1704_S819_	2014	D	05.05.2014	S819	13.8	20.5	Fam-62_1_201	S819	819	3739	62	2010	H
2911_1704_S820_	2014	D	05.05.2014	S820	12.8	17.5	Fam-57_2009_	S820	820	3740	57	2009	W
2911_1704_S821_	2014	D	05.05.2014	S821	15.3	27.5	Fam-40_2009_	S821	821	3741	40	2009	H
2911_1704_S822_	2014	D	05.05.2014	S822	14.5	24	Fam-40_2009_	S822	822	3742	40	2009	H
2911_1704_S823_	2014	D	05.05.2014	S823	13.9	20.5	Fam-52_2009_	S823	823	3743	52	2009	W
2911_1704_S824_	2014	D	05.05.2014	S824	14.6	26	Fam-50_2009_	S824	824	3744	50	2009	H
2911_1704_S825_	2014	D	05.05.2014	S825	14.3	23.5	Fam-39_2009_	S825	825	3745	39	2009	F
2911_1704_S827_	2014	D	06.05.2014	S827	13	18.5	Fam-42_2009_	S827	827	3746	42	2009	H
2911_1704_S828_	2014	D	06.05.2014	S828	14.6	26	Fam-45_2009_	S828	828	3747	45	2009	F
2911_1704_S830_	2014	D	06.05.2014	S830	14	22	Fam-36_2009_	S830	830	3748	36	2009	H
2911_1704_S832_	2014	D	06.05.2014	S832	15.8	31	Fam-85_2010_	S832	832	3749	85	2010	W

2911_1704_S833_	2014	D	06.05.2014	S833	12.8	16.5	Fam-59_2009_	S833	833	3750	59	2009	W
2911_1704_S834_	2014	D	06.05.2014	S834	15.8	31.5	Fam-34_2009_	S834	834	3751	34	2009	H
2911_1704_S835_	2014	D	06.05.2014	S835	13.6	20	Fam-59_2009_	S835	835	3752	59	2009	W
2911_1704_S836_	2014	D	06.05.2014	S836	14.1	25	Fam-77_2010_	S836	836	3753	77	2010	F
2911_1704_S837_	2014	T	06.05.2014	S837	14.9	25.5	Family-72_201	S837	837	3754	72	2010	H
2911_1704_S838_	2014	D	06.05.2014	S838	15	27	Fam-77_2010_	S838	838	3755	77	2010	F
2911_1704_S839_	2014	D	06.05.2014	S839	12.1	14	Fam-59_2009_	S839	839	3756	59	2009	W
2911_1704_S840_	2014	D	06.05.2014	S840	14.3	24.5	Fam-36_2009_	S840	840	3757	36	2009	H
2911_1704_S841_	2014	D	06.05.2014	S841	15.3	29.5	Fam-48_2009_	S841	841	3758	48	2009	H
2911_1704_S842_	2014	D	06.05.2014	S842	14.2	23.5	Fam-52_2009_	S842	842	3759	52	2009	W
2911_1704_S843_	2014	D	06.05.2014	S843	12.3	15.5	Fam-36_2009_	S843	843	3760	36	2009	H
2911_1704_S844_	2014	D	06.05.2014	S844	13.3	20	Fam-62_1_201	S844	844	3761	62	2010	H
2911_1704_S845_	2014	D	06.05.2014	S845	15.6	29.5	Fam-32_2009_	S845	845	3762	32	2009	H
2911_1705_S847_	2014	D	06.05.2014	S847	13	17	Fam-59_2009_	S847	847	3763	59	2009	W

2911_1705_S848_	2014	D	06.05.2014	S848	14.6	26	Fam-38_2009_S848	848	3764	38	2009	H
2911_1705_S849_	2014	D	06.05.2014	S849	15.1	28.5	Fam-49_2009_S849	849	3765	49	2009	F
2911_1705_S850_	2014	D	06.05.2014	S850	14.4	24.5	Fam-58_2009_S850	850	3766	58	2009	W
2911_1705_S851_	2014	D	06.05.2014	S851	14.2	23.5	Fam-57_2009_S851	851	3767	57	2009	W
2911_1705_S852_	2014	D	08.05.2014	S852	15.6	31.5	Fam-38_2009_S852	852	3768	38	2009	H
2911_1705_S853_	2014	D	08.05.2014	S853	14.1	21.5	Fam-71_2010_S853	853	3769	71	2010	F
2911_1705_S854_	2014	D	08.05.2014	S854	13.5	20	Fam-72_2010_S854	854	3770	72	2010	H
2911_1705_S855_	2014	D	08.05.2014	S855	14.8	26	Fam-40_2009_S855	855	3771	40	2009	H
2911_1705_S856_	2014	D	08.05.2014	S856	13.3	19	Fam-80_2010_S856	856	3772	80	2010	H
2911_1705_S857_	2014	D	08.05.2014	S857	14	23	Fam-42_2009_S857	857	3773	42	2009	H
2911_1705_S858_	2014	D	08.05.2014	S858	14.9	25	Fam-70_2010_S858	858	3774	70	2010	H
2911_1705_S859_	2014	D	08.05.2014	S859	13.8	21.5	Fam-85_2010_S859	859	3775	85	2010	W
2911_1705_S860_	2014	D	08.05.2014	S860	13	17	Fam-44_2009_S860	860	3776	44	2009	H
2911_1705_S861_	2014	D	08.05.2014	S861	14.5	26.5	Fam-45_2009_S861	861	3777	45	2009	F

2911_1705_S862_	2014	D	08.05.2014	S862	14.5	25.5	Fam-79_2010_S862	862	3778	79	2010 F
2911_1705_S863_	2014	D	08.05.2014	S863	14.8	26.5	Fam-50_2009_S863	863	3779	50	2009 H
2911_1705_S864_	2014	D	08.05.2014	S864	13.7	21	Fam-58_2009_S864	864	3780	58	2009 W
2911_1705_S865_	2014	D	08.05.2014	S865	16.6	35.5	Fam-79_2010_S865	865	3781	79	2010 F
2911_1705_S866_	2014	D	08.05.2014	S866	13.7	21	Fam-85_2010_S866	866	3782	85	2010 W
2911_1705_S867_	2014	D	08.05.2014	S867	14.4	25	Fam-42_2009_S867	867	3783	42	2009 H
2911_1705_S868_	2014	D	08.05.2014	S868	13.5	19.5	Fam-36_2009_S868	868	3784	36	2009 H
2911_1705_S869_	2014	D	08.05.2014	S869	13.7	22	Fam-58_2009_S869	869	3785	58	2009 W
2911_1705_S870_	2014	D	08.05.2014	S870	14.4	25.5	Fam-42_2009_S870	870	3786	42	2009 H
2911_1705_S871_	2014	D	08.05.2014	S871	14	22	Fam-43_2009_S871	871	3787	43	2009 F
2911_1705_S872_	2014	D	08.05.2014	S872	13.6	21.5	Fam-32_2009_S872	872	3788	32	2009 H
2911_1705_S873_	2014	D	08.05.2014	S873	13.9	22	Fam-32_2009_S873	873	3789	32	2009 H
2911_1705_S874_	2014	D	08.05.2014	S874	13	18.5	Fam-38_2009_S874	874	3790	38	2009 H
2911_1705_S875_	2014	D	08.05.2014	S875	14.8	25.5	Fam-64_2010_S875	875	3791	64	2010 H

2911_1705_S876_	2014	D	08.05.2014	S876	14.7	26	Fam-40_2009_	S876	876	3792	40	2009	H
2911_1705_S877_	2014	T	08.05.2014	S877	12.9	17	Family-42_200	S877	877	3793	42	2009	H
2911_1705_S878_	2014	T	08.05.2014	S878	13.7	19.5	Family-76_201	S878	878	3794	76	2010	H
2911_1705_S879_	2014	D	08.05.2014	S879	13.8	21.5	Fam-44_2009_	S879	879	3795	44	2009	H
2911_1705_S880_	2014	D	08.05.2014	S880	14.1	21.5	Fam-57_2009_	S880	880	3796	57	2009	W
2912_1705_S881_	2014	D	08.05.2014	S881	13.9	22.5	Fam-47_2009_	S881	881	3797	47	2009	F
2912_1705_S882_	2014	D	08.05.2014	S882	13.1	17.5	Fam-36_2009_	S882	882	3798	36	2009	H
2912_1705_S883_	2014	D	08.05.2014	S883	13.4	18	Fam-59_2009_	S883	883	3799	59	2009	W
2912_1705_S884_	2014	D	08.05.2014	S884	16	34.5	Fam-62_1_201	S884	884	3800	62	2010	H
2912_1705_S885_	2014	D	08.05.2014	S885	15.1	28.5	Fam-51_2009_	S885	885	3801	51	2009	W
2912_1705_S886_	2014	D	08.05.2014	S886	14.5	27.5	Fam-42_2009_	S886	886	3802	42	2009	H
2912_1705_S887_	2014	D	08.05.2014	S887	12.6	16.5	Fam-57_2009_	S887	887	3803	57	2009	W
2912_1705_S888_	2014	D	08.05.2014	S888	14.9	27	Fam-31_2009_	S888	888	3804	31	2009	F
2912_1705_S889_	2014	D	08.05.2014	S889	12.5	16.5	Fam-69_2010_	S889	889	3805	69	2010	F

2912_1705_S890_	2014	D	08.05.2014	S890	13	19.5	Fam-42_2009_S890	890	3806	42	2009	H
2912_1705_S891_	2014	D	08.05.2014	S891	13.5	20	Fam-31_2009_S891	891	3807	31	2009	F
2912_1705_S892_	2014	D	08.05.2014	S892	13	17.5	Fam-58_2009_S892	892	3808	58	2009	W
2912_1705_S894_	2014	D	08.05.2014	S894	13.5	19	Fam-39_2009_S894	894	3809	39	2009	F
2912_1705_S895_	2014	D	08.05.2014	S895	14.1	23	Fam-78_2010_S895	895	3810	78	2010	H
2912_1705_S896_	2014	D	08.05.2014	S896	13.9	21	Fam-83_2010_S896	896	3811	83	2010	W
2912_1705_S897_	2014	D	08.05.2014	S897	14.5	24.5	Fam-65_2010_S897	897	3812	65	2010	F
2912_1705_S898_	2014	D	08.05.2014	S898	14.5	27.5	Fam-45_2009_S898	898	3813	45	2009	F
2912_1705_S899_	2014	D	08.05.2014	S899	14.2	22.5	Fam-74_2010_S899	899	3814	74	2010	H
2912_1705_S900_	2014	D	08.05.2014	S900	14.5	25.5	Fam-70_2010_S900	900	3815	70	2010	H
2912_1705_S901_	2014	D	08.05.2014	S901	14.5	25	Fam-83_2010_S901	901	3816	83	2010	W
2912_1705_S902_	2014	D	08.05.2014	S902	15	26.5	Fam-39_2009_S902	902	3817	39	2009	F
2912_1705_S903_	2014	D	08.05.2014	S903	17.5	41.5	Fam-70_2010_S903	903	3818	70	2010	H
2912_1705_S904_	2014	D	08.05.2014	S904	13.1	19	Fam-78_2010_S904	904	3819	78	2010	H

2912_1705_S905_	2014	D	08.05.2014	S905	13.4	21	Fam-42_2009_	S905	905	3820	42	2009 H
2912_1705_S906_	2014	D	08.05.2014	S906	14.5	25.5	Fam-38_2009_	S906	906	3821	38	2009 H
2912_1705_S907_	2014	D	08.05.2014	S907	14	21.5	Fam-60_2009_	S907	907	3822	60	2009 W
2912_1705_S908_	2014	T	08.05.2014	S908	12.6	15	Family-34_200	S908	908	3823	34	2009 H
2912_1705_S909_	2014	D	08.05.2014	S909	13.5	18	Fam-64_2010_	S909	909	3824	64	2010 H
2912_1705_S910_	2014	D	08.05.2014	S910	12.4	15	Fam-54_2009_	S910	910	3825	54	2009 W
2912_1705_S912_	2014	D	08.05.2014	S912	14	24	Fam-90_2010_	S912	912	3826	90	2010 W
2912_1705_S913_	2014	D	08.05.2014	S913	14.1	23	Fam-54_2009_	S913	913	3827	54	2009 W
2912_1705_S914_	2014	D	08.05.2014	S914	14.6	26	Fam-42_2009_	S914	914	3828	42	2009 H
2912_1705_S915_	2014	D	08.05.2014	S915	13.5	21	Fam-38_2009_	S915	915	3829	38	2009 H
2912_1705_S916_	2014	D	08.05.2014	S916	15.7	31	Fam-73_2010_	S916	916	3830	73	2010 F
2912_1705_S917_	2014	D	08.05.2014	S917	14.5	26	Fam-65_2010_	S917	917	3831	65	2010 F
2912_1705_S918_	2014	D	08.05.2014	S918	13	19.5	Fam-63_2010_	S918	918	3832	63	2010 F
2912_1705_S920_	2014	D	08.05.2014	S920	13	19	Fam-38_2009_	S920	920	3833	38	2009 H

2912_1705_S921_	2014	D	08.05.2014	S921	13.8	22	Fam-60_2009_	S921	921	3834	60	2009	W
2912_1705_S922_	2014	D	08.05.2014	S922	13.5	22	Fam-87_2010_	S922	922	3835	87	2010	W
2912_1705_S923_	2014	D	08.05.2014	S923	11.5	14	Fam-59_2009_	S923	923	3836	59	2009	W
2912_1705_S924_	2014	D	08.05.2014	S924	14.5	24.5	Fam-31_2009_	S924	924	3837	31	2009	F
2912_1705_S925_	2014	D	08.05.2014	S925	15.5	29	Fam-52_2009_	S925	925	3838	52	2009	W
2912_1705_S926_	2014	D	08.05.2014	S926	12.5	16	Fam-69_2010_	S926	926	3839	69	2010	F
2912_1705_S927_	2014	D	08.05.2014	S927	13.8	20	Fam-49_2009_	S927	927	3840	49	2009	F
2912_1705_S928_	2014	D	08.05.2014	S928	14.5	24	Fam-52_2009_	S928	928	3841	52	2009	W
2912_1705_S929_	2014	D	08.05.2014	S929	13.1	18	Fam-62_1_201	S929	929	3842	62	2010	H
2912_1705_S930_	2014	D	08.05.2014	S930	15.1	30	Fam-80_2010_	S930	930	3843	80	2010	H
2912_1705_S931_	2014	D	08.05.2014	S931	13.2	18.5	Fam-57_2009_	S931	931	3844	57	2009	W
2912_1705_S932_	2014	D	08.05.2014	S932	12.5	15.5	Fam-59_2009_	S932	932	3845	59	2009	W
2912_1705_S933_	2014	D	08.05.2014	S933	15.1	26.5	Fam-40_2009_	S933	933	3846	40	2009	H
2912_1705_S934_	2014	D	08.05.2014	S934	12.7	15.5	Fam-51_2009_	S934	934	3847	51	2009	W

2912_1705_S935_	2014	D	08.05.2014	S935	16.2	35	Fam-83_2010_	S935	935	3848	83	2010	W
2912_1705_S936_	2014	D	08.05.2014	S936	14	21.5	Fam-40_2009_	S936	936	3849	40	2009	H
2912_1705_S937_	2014	D	08.05.2014	S937	13.8	20.5	Fam-40_2009_	S937	937	3850	40	2009	H
2912_1705_S938_	2014	D	08.05.2014	S938	13.6	20	Fam-62_1_201	S938	938	3851	62	2010	H
2912_1705_S939_	2014	D	08.05.2014	S939	14	23	Fam-38_2009_	S939	939	3852	38	2009	H
2912_1706_S940_	2014	D	08.05.2014	S940	14.5	25	Fam-87_2010_	S940	940	3853	87	2010	W
2912_1706_S941_	2014	D	08.05.2014	S941	13.1	19	Fam-54_2009_	S941	941	3854	54	2009	W
2912_1706_S942_	2014	D	08.05.2014	S942	13.4	18.5	Fam-57_2009_	S942	942	3855	57	2009	W
2912_1706_S943_	2014	D	09.05.2014	S943	12	14	Fam-59_2009_	S943	943	3856	59	2009	W
2912_1706_S944_	2014	D	09.05.2014	S944	16.5	37	Fam-66_2010_	S944	944	3857	66	2010	H
2912_1706_S945_	2014	D	09.05.2014	S945	14.2	24	Fam-42_2009_	S945	945	3858	42	2009	H
2912_1706_S946_	2014	D	09.05.2014	S946	14.5	26.5	Fam-63_2010_	S946	946	3859	63	2010	F
2912_1706_S947_	2014	D	09.05.2014	S947	12.9	18	Fam-56_2009_	S947	947	3860	56	2009	W
2912_1706_S948_	2014	D	09.05.2014	S948	13	19.5	Fam-42_2009_	S948	948	3861	42	2009	H

2912_1706_S949_	2014	D	09.05.2014	S949	14	21	Fam-69_2010_S949	949	3862	69	2010	F
2912_1706_S950_	2014	D	09.05.2014	S950	14.9	25	Fam-34_2009_S950	950	3863	34	2009	H
2912_1706_S951_	2014	D	09.05.2014	S951	13.8	20.5	Fam-56_2009_S951	951	3864	56	2009	W
2912_1706_S953_	2014	D	09.05.2014	S953	14.2	24	Fam-83_2010_S953	953	3865	83	2010	W
2912_1706_S954_	2014	D	09.05.2014	S954	12.7	17	Fam-85_2010_S954	954	3866	85	2010	W
2912_1706_S955_	2014	D	09.05.2014	S955	14.1	22	Fam-57_2009_S955	955	3867	57	2009	W
2912_1706_S956_	2014	D	09.05.2014	S956	13.5	20.5	Fam-54_2009_S956	956	3868	54	2009	W
2912_1706_S957_	2014	D	13.05.2014	S957	13.5	22	Fam-42_2009_S957	957	3869	42	2009	H
2912_1706_S958_	2014	D	13.05.2014	S958	15.1	26	Fam-66_2010_S958	958	3870	66	2010	H
2912_1706_S959_	2014	D	13.05.2014	S959	14.3	25	Fam-59_2009_S959	959	3871	59	2009	W
2912_1706_S960_	2014	D	13.05.2014	S960	12.9	19.5	Fam-47_2009_S960	960	3872	47	2009	F
2913_1706_S961_	2014	D	13.05.2014	S961	15	24.5	Fam-37_2009_S961	961	3873	37	2009	F
2913_1706_S962_	2014	D	13.05.2014	S962	12.9	17.5	Fam-38_2009_S962	962	3874	38	2009	H
2913_1706_S963_	2014	D	13.05.2014	S963	13.2	17	Fam-72_2010_S963	963	3875	72	2010	H

2913_1706_S964_	2014	D	13.05.2014	S964	12.5	14.5	Fam-57_2009_S964	964	3876	57	2009	W
2913_1706_S965_	2014	D	13.05.2014	S965	14.3	24	Fam-54_2009_S965	965	3877	54	2009	W
2913_1706_S966_	2014	D	13.05.2014	S966	14.2	23	Fam-59_2009_S966	966	3878	59	2009	W
2913_1706_S967_	2014	D	13.05.2014	S967	13.9	21.5	Fam-59_2009_S967	967	3879	59	2009	W
2913_1706_S968_	2014	D	13.05.2014	S968	16.4	36	Fam-50_2009_S968	968	3880	50	2009	H
2913_1706_S969_	2014	D	13.05.2014	S969	13.6	21	Fam-36_2009_S969	969	3881	36	2009	H
2913_1706_S970_	2014	D	13.05.2014	S970	14.4	23.5	Fam-57_2009_S970	970	3882	57	2009	W
2913_1706_S971_	2014	D	13.05.2014	S971	13	17.5	Fam-59_2009_S971	971	3883	59	2009	W
2913_1706_S972_	2014	D	13.05.2014	S972	13.5	21	Fam-80_2010_S972	972	3884	80	2010	H
2913_1706_S973_	2014	D	13.05.2014	S973	14.1	22	Fam-70_2010_S973	973	3885	70	2010	H
2913_1706_S974_	2014	D	13.05.2014	S974	13.6	20.5	Fam-59_2009_S974	974	3886	59	2009	W
2913_1706_S975_	2014	D	13.05.2014	S975	12.6	16	Fam-59_2009_S975	975	3887	59	2009	W
2913_1706_S976_	2014	D	13.05.2014	S976	14	23	Fam-59_2009_S976	976	3888	59	2009	W
2913_1706_S977_	2014	D	13.05.2014	S977	15.2	30	Fam-64_2010_S977	977	3889	64	2010	H

2913_1706_S978_	2014	D	13.05.2014	S978	15.1	28.5	Fam-59_2009_S978	978	3890	59	2009	W
2913_1706_S979_	2014	D	13.05.2014	S979	14	22	Fam-56_2009_S979	979	3891	56	2009	W
2913_1706_S981_	2014	D	13.05.2014	S981	14.5	24	Fam-36_2009_S981	981	3892	36	2009	H
2913_1706_S983_	2014	D	13.05.2014	S983	13.3	21.5	Fam-66_2010_S983	983	3893	66	2010	H
2913_1706_S984_	2014	D	13.05.2014	S984	14.7	28	Fam-31_2009_S984	984	3894	31	2009	F
2913_1706_S985_	2014	D	13.05.2014	S985	13.1	19	Fam-73_2010_S985	985	3895	73	2010	F
2913_1706_S986_	2014	D	13.05.2014	S986	14.2	26.5	Fam-87_2010_S986	986	3896	87	2010	W
2913_1706_S988_	2014	D	13.05.2014	S988	15.7	31.5	Fam-63_2010_S988	988	3897	63	2010	F
2913_1706_S989_	2014	D	13.05.2014	S989	14	24.5	Fam-42_2009_S989	989	3898	42	2009	H
2913_1706_S990_	2014	D	13.05.2014	S990	15.6	30	Fam-89_2010_S990	990	3899	89	2010	W
2913_1706_S991_	2014	D	13.05.2014	S991	12.5	16.5	Fam-59_2009_S991	991	3900	59	2009	W
2913_1706_S993_	2014	D	13.05.2014	S993	13	19.5	Fam-42_2009_S993	993	3901	42	2009	H
2913_1706_S994_	2014	D	13.05.2014	S994	14.4	26.5	Fam-41_2009_S994	994	3902	41	2009	F
2913_1706_S995_	2014	D	13.05.2014	S995	13.8	22.5	Fam-78_2010_S995	995	3903	78	2010	H

2913_1706_S997_	2014	D	13.05.2014	S997	13	18.5	Fam-59_2009_	S997	997	3904	59	2009	W
2913_1706_S998_	2014	D	13.05.2014	S998	13.5	19	Fam-38_2009_	S998	998	3905	38	2009	H
2913_1706_S100(	2014	D	13.05.2014	S1000	13.3	19.5	Fam-38_2009_	S1000	1000	3906	38	2009	H
2913_1706_S1001	2014	D	13.05.2014	S1001	14.2	23	Fam-68_2010_	S1001	1001	3907	68	2010	H
2913_1706_S1002	2014	D	13.05.2014	S1002	14.3	22	Fam-89_2010_	S1002	1002	3908	89	2010	W
2913_1706_S1003	2014	D	13.05.2014	S1003	14.5	27.5	Fam-64_2010_	S1003	1003	3909	64	2010	H
2913_1706_S1004	2014	D	13.05.2014	S1004	14.2	24	Fam-42_2009_	S1004	1004	3910	42	2009	H
2913_1706_S1005	2014	D	13.05.2014	S1005	14.2	22	Fam-54_2009_	S1005	1005	3911	54	2009	W
2913_1706_S1006	2014	D	13.05.2014	S1006	13.9	21.5	Fam-69_2010_	S1006	1006	3912	69	2010	F
2913_1706_S1007	2014	D	13.05.2014	S1007	13.3	19.5	Fam-57_2009_	S1007	1007	3913	57	2009	W
2913_1706_S1008	2014	D	13.05.2014	S1008	14	22	Fam-57_2009_	S1008	1008	3914	57	2009	W
2913_1706_S1009	2014	D	13.05.2014	S1009	12.3	14	Fam-59_2009_	S1009	1009	3915	59	2009	W
2913_1706_S101(	2014	D	13.05.2014	S1010	13	17.5	Fam-36_2009_	S1010	1010	3916	36	2009	H
2913_1706_S1011	2014	D	13.05.2014	S1011	14.5	24	Fam-32_2009_	S1011	1011	3917	32	2009	H

2913_1706_S1012	2014	D	13.05.2014	S1012	14	23.5	Fam-41_2009_	S1012	1012	3918	41	2009	F
2913_1706_S1013	2014	D	13.05.2014	S1013	14.6	25	Fam-52_2009_	S1013	1013	3919	52	2009	W
2913_1706_S1014	2014	D	13.05.2014	S1014	14.8	27	Fam-50_2009_	S1014	1014	3920	50	2009	H
2913_1706_S1015	2014	D	13.05.2014	S1015	14	22	Fam-32_2009_	S1015	1015	3921	32	2009	H
2913_1706_S1016	2014	D	13.05.2014	S1016	15	26	Fam-32_2009_	S1016	1016	3922	32	2009	H
2913_1706_S1018	2014	D	13.05.2014	S1018	13.5	19.5	Fam-42_2009_	S1018	1018	3923	42	2009	H
2913_1706_S1020	2014	D	13.05.2014	S1020	13.6	20.5	Fam-36_2009_	S1020	1020	3924	36	2009	H
2913_1706_S1021	2014	D	13.05.2014	S1021	15	28	Fam-58_2009_	S1021	1021	3925	58	2009	W
2913_1706_S1022	2014	D	13.05.2014	S1022	16.5	35	Fam-40_2009_	S1022	1022	3926	40	2009	H
2913_1706_S1023	2014	D	13.05.2014	S1023	14	20.5	Fam-82_2010_	S1023	1023	3927	82	2010	W
2913_1706_S1024	2014	D	13.05.2014	S1024	15	26	Fam-43_2009_	S1024	1024	3928	43	2009	F
2913_1706_S1025	2014	D	13.05.2014	S1025	15.6	30	Fam-53_2009_	S1025	1025	3929	53	2009	W
2913_1706_S1026	2014	D	13.05.2014	S1026	14	20	Fam-76_2010_	S1026	1026	3930	76	2010	H
2913_1706_S1027	2014	D	13.05.2014	S1027	13.2	18	Fam-57_2009_	S1027	1027	3931	57	2009	W

2913_1706_S1028	2014	D	13.05.2014	S1028	13.8	20.5	Fam-36_2009_	S1028	1028	3932	36	2009	H
2913_1706_S1029	2014	D	13.05.2014	S1029	15.2	30	Fam-45_2009_	S1029	1029	3933	45	2009	F
2913_1706_S1030	2014	D	13.05.2014	S1030	13.8	21.5	Fam-34_2009_	S1030	1030	3934	34	2009	H
2913_1706_S1031	2014	D	13.05.2014	S1031	15.8	22.5	Fam-42_2009_	S1031	1031	3935	42	2009	H
2913_1706_S1032	2014	D	13.05.2014	S1032	12.6	16	Fam-78_2010_	S1032	1032	3936	78	2010	H
2913_1706_S1033	2014	D	13.05.2014	S1033	13.4	19.5	Fam-83_2010_	S1033	1033	3937	83	2010	W
2913_1707_S1034	2014	D	13.05.2014	S1034	13.3	20	Fam-90_2010_	S1034	1034	3938	90	2010	W
2913_1707_S1035	2014	D	13.05.2014	S1035	13.4	20.5	Fam-36_2009_	S1035	1035	3939	36	2009	H
2913_1707_S1036	2014	D	13.05.2014	S1036	12.6	16.5	Fam-49_2009_	S1036	1036	3940	49	2009	F
2913_1707_S1037	2014	D	13.05.2014	S1037	11.4	12	Fam-59_2009_	S1037	1037	3941	59	2009	W
2913_1707_S1038	2014	D	13.05.2014	S1038	14.6	25	Fam-38_2009_	S1038	1038	3942	38	2009	H
2913_1707_S1039	2014	D	13.05.2014	S1039	13.7	20.5	Fam-59_2009_	S1039	1039	3943	59	2009	W
2913_1707_S1040	2014	D	13.05.2014	S1040	13.5	20	Fam-83_2010_	S1040	1040	3944	83	2010	W
2914_1707_S1041	2014	D	13.05.2014	S1041	12.8	17	Fam-62_1_201	S1041	1041	3945	62	2010	H

2914_1707_S1042	2014	D	13.05.2014	S1042	15.4	28	Fam-48_2009_	S1042	1042	3946	48	2009	H
2914_1707_S1043	2014	D	13.05.2014	S1043	13	18.5	Fam-58_2009_	S1043	1043	3947	58	2009	W
2914_1707_S1044	2014	D	13.05.2014	S1044	14.5	25.5	Fam-39_2009_	S1044	1044	3948	39	2009	F
2914_1707_S1045	2014	D	13.05.2014	S1045	13.7	20	Fam-32_2009_	S1045	1045	3949	32	2009	H
2914_1707_S1046	2014	D	13.05.2014	S1046	15.3	32	Fam-80_2010_	S1046	1046	3950	80	2010	H
2914_1707_S1047	2014	D	13.05.2014	S1047	12.6	16	Fam-50_2009_	S1047	1047	3951	50	2009	H
2914_1707_S1048	2014	D	13.05.2014	S1048	12.1	14	Fam-54_2009_	S1048	1048	3952	54	2009	W
2914_1707_S1049	2014	D	13.05.2014	S1049	13	17.5	Fam-90_2010_	S1049	1049	3953	90	2010	W
2914_1707_S1050	2014	D	13.05.2014	S1050	12.9	17.5	Fam-38_2009_	S1050	1050	3954	38	2009	H
2914_1707_S1051	2014	D	13.05.2014	S1051	14.7	27	Fam-61_1_201	S1051	1051	3955	61	2010	F
2914_1707_S1052	2014	D	13.05.2014	S1052	14.7	27	Fam-39_2009_	S1052	1052	3956	39	2009	F
2914_1707_S1053	2014	D	13.05.2014	S1053	14.8	25.5	Fam-51_2009_	S1053	1053	3957	51	2009	W
2914_1707_S1055	2014	D	13.05.2014	S1055	13	16.5	Fam-62_1_201	S1055	1055	3958	62	2010	H
2914_1707_S1056	2014	D	13.05.2014	S1056	15.4	31	Fam-80_2010_	S1056	1056	3959	80	2010	H

2914_1707_S1057	2014	D	13.05.2014	S1057	15	27	Fam-58_2009_	S1057	1057	3960	58	2009	W
2914_1707_S1058	2014	D	13.05.2014	S1058	13.5	19.5	Fam-59_2009_	S1058	1058	3961	59	2009	W
2914_1707_S1059	2014	D	13.05.2014	S1059	12.7	17	Fam-58_2009_	S1059	1059	3962	58	2009	W
2914_1707_S1060	2014	D	13.05.2014	S1060	13	18	Fam-44_2009_	S1060	1060	3963	44	2009	H
2914_1707_S1061	2014	D	13.05.2014	S1061	14.6	25	Fam-32_2009_	S1061	1061	3964	32	2009	H
2914_1707_S1062	2014	D	13.05.2014	S1062	14.6	26	Fam-39_2009_	S1062	1062	3965	39	2009	F
2914_1707_S1063	2014	D	13.05.2014	S1063	12.3	16	Fam-85_2010_	S1063	1063	3966	85	2010	W
2914_1707_S1064	2014	D	13.05.2014	S1064	14.5	25.5	Fam-59_2009_	S1064	1064	3967	59	2009	W
2914_1707_S1065	2014	D	13.05.2014	S1065	13.5	19.5	Fam-59_2009_	S1065	1065	3968	59	2009	W
2914_1707_S1066	2014	D	15.05.2014	S1066	14	22.5	Fam-45_2009_	S1066	1066	3969	45	2009	F
2914_1707_S1067	2014	D	15.05.2014	S1067	14	20	Fam-51_2009_	S1067	1067	3970	51	2009	W
2914_1707_S1068	2014	D	15.05.2014	S1068	13	20.5	Fam-86_2010_	S1068	1068	3971	86	2010	W
2914_1707_S1070	2014	T	15.05.2014	S1070	16.5	33	Family-72_201	S1070	1070	3972	72	2010	H
2914_1707_S1071	2014	D	15.05.2014	S1071	13.2	19	Fam-52_2009_	S1071	1071	3973	52	2009	W

2914_1707_S1073	2014	D	15.05.2014	S1073	15.5	32.5	Fam-80_2010_	S1073	1073	3974	80	2010	H
2914_1707_S1074	2014	D	15.05.2014	S1074	14.9	25.5	Fam-65_2010_	S1074	1074	3975	65	2010	F
2914_1707_S1075	2014	D	15.05.2014	S1075	14.6	23.5	Fam-40_2009_	S1075	1075	3976	40	2009	H
2914_1707_S1076	2014	D	15.05.2014	S1076	13	17	Fam-57_2009_	S1076	1076	3977	57	2009	W
2914_1707_S1077	2014	D	15.05.2014	S1077	13.9	22.5	Fam-45_2009_	S1077	1077	3978	45	2009	F
2914_1707_S1078	2014	D	15.05.2014	S1078	14.5	23	Fam-59_2009_	S1078	1078	3979	59	2009	W
2914_1707_S1079	2014	D	15.05.2014	S1079	14.1	23.5	Fam-57_2009_	S1079	1079	3980	57	2009	W
2914_1707_S1080	2014	D	15.05.2014	S1080	13.4	18.5	Fam-57_2009_	S1080	1080	3981	57	2009	W
2914_1707_S1081	2014	D	15.05.2014	S1081	13.5	19.5	Fam-54_2009_	S1081	1081	3982	54	2009	W
2914_1707_S1082	2014	D	15.05.2014	S1082	13.2	19.5	Fam-38_2009_	S1082	1082	3983	38	2009	H
2914_1707_S1083	2014	D	15.05.2014	S1083	13.2	18	Fam-59_2009_	S1083	1083	3984	59	2009	W
2914_1707_S1084	2014	D	15.05.2014	S1084	12.5	14.5	Fam-76_2010_	S1084	1084	3985	76	2010	H
2914_1707_S1085	2014	D	15.05.2014	S1085	14	21	Fam-44_2009_	S1085	1085	3986	44	2009	H
2914_1707_S1086	2014	D	15.05.2014	S1086	13.6	20.5	Fam-32_2009_	S1086	1086	3987	32	2009	H

2914_1707_S1087	2014	D	15.05.2014	S1087	16	30.5	Fam-73_2010_	S1087	1087	3988	73	2010 F
2914_1707_S1088	2014	D	15.05.2014	S1088	13.5	19	Fam-54_2009_	S1088	1088	3989	54	2009 W
2914_1707_S1089	2014	D	15.05.2014	S1089	13.5	19	Fam-54_2009_	S1089	1089	3990	54	2009 W
2914_1707_S1090	2014	D	15.05.2014	S1090	13.2	18	Fam-68_2010_	S1090	1090	3991	68	2010 H
2914_1707_S1091	2014	D	15.05.2014	S1091	13.5	19	Fam-85_2010_	S1091	1091	3992	85	2010 W
2914_1707_S1092	2014	D	15.05.2014	S1092	14.1	23.5	Fam-62_1_201	S1092	1092	3993	62	2010 H
2914_1707_S1093	2014	D	15.05.2014	S1093	14	21.5	Fam-55_2009_	S1093	1093	3994	55	2009 W
2914_1707_S1094	2014	D	15.05.2014	S1094	14.2	22.5	Fam-54_2009_	S1094	1094	3995	54	2009 W
2914_1707_S1095	2014	D	15.05.2014	S1095	14.3	22	Fam-51_2009_	S1095	1095	3996	51	2009 W
2914_1707_S1096	2014	D	15.05.2014	S1096	13.5	20	Fam-44_2009_	S1096	1096	3997	44	2009 H
2914_1707_S1097	2014	D	15.05.2014	S1097	13.2	20.5	Fam-38_2009_	S1097	1097	3998	38	2009 H
2914_1707_S1098	2014	D	15.05.2014	S1098	14.5	24.5	Fam-68_2010_	S1098	1098	3999	68	2010 H
2914_1707_S1100	2014	D	15.05.2014	S1100	13.4	18.5	Fam-76_2010_	S1100	1100	4000	76	2010 H
2914_1707_S1101	2014	D	15.05.2014	S1101	13.5	20.5	Fam-38_2009_	S1101	1101	4001	38	2009 H

2914_1707_S1102	2014	D	15.05.2014	S1102	14.5	22	Fam-68_2010_	S1102	1102	4002	68	2010	H
2914_1707_S1103	2014	D	15.05.2014	S1103	14.5	23.5	Fam-57_2009_	S1103	1103	4003	57	2009	W
2914_1707_S1104	2014	D	15.05.2014	S1104	14.9	27	Fam-71_2010_	S1104	1104	4004	71	2010	F
2914_1707_S1105	2014	D	15.05.2014	S1105	14.9	24.5	Fam-39_2009_	S1105	1105	4005	39	2009	F
2914_1707_S1106	2014	D	15.05.2014	S1106	15.8	30	Fam-83_2010_	S1106	1106	4006	83	2010	W
2914_1707_S1107	2014	D	15.05.2014	S1107	13.5	20.5	Fam-59_2009_	S1107	1107	4007	59	2009	W
2914_1707_S1108	2014	D	15.05.2014	S1108	14.6	26	Fam-50_2009_	S1108	1108	4008	50	2009	H
2914_1707_S1109	2014	D	15.05.2014	S1109	15	26	Fam-59_2009_	S1109	1109	4009	59	2009	W
2914_1707_S1110	2014	D	15.05.2014	S1110	13.8	21	Fam-38_2009_	S1110	1110	4010	38	2009	H
2914_1707_S1111	2014	D	15.05.2014	S1111	13.6	18.5	Fam-50_2009_	S1111	1111	4011	50	2009	H
2914_1707_S1112	2014	D	15.05.2014	S1112	15.6	32.5	Fam-47_2009_	S1112	1112	4012	47	2009	F
2914_1707_S1113	2014	D	15.05.2014	S1113	15.5	28.5	Fam-43_2009_	S1113	1113	4013	43	2009	F
2914_1707_S1114	2014	D	15.05.2014	S1114	14.5	25	Fam-54_2009_	S1114	1114	4014	54	2009	W
2914_1707_S1115	2014	D	15.05.2014	S1115	14	22	Fam-59_2009_	S1115	1115	4015	59	2009	W

2914_1707_S1116	2014	D	15.05.2014	S1116	15.1	27	Fam-54_2009_	S1116	1116	4016	54	2009	W
2914_1707_S1117	2014	D	15.05.2014	S1117	14.3	23	Fam-53_2009_	S1117	1117	4017	53	2009	W
2914_1707_S1118	2014	D	15.05.2014	S1118	14	21.5	Fam-59_2009_	S1118	1118	4018	59	2009	W
2914_1707_S1119	2014	D	15.05.2014	S1119	13.8	21	Fam-54_2009_	S1119	1119	4019	54	2009	W
2914_1707_S1120	2014	D	15.05.2014	S1120	16	32.5	Fam-87_2010_	S1120	1120	4020	87	2010	W
2915_1707_S1121	2014	D	15.05.2014	S1121	15	26.5	Fam-42_2009_	S1121	1121	4021	42	2009	H
2915_1707_S1122	2014	D	15.05.2014	S1122	14.5	23	Fam-42_2009_	S1122	1122	4022	42	2009	H
2915_1707_S1123	2014	D	15.05.2014	S1123	15.8	31	Fam-47_2009_	S1123	1123	4023	47	2009	F
2915_1707_S1124	2014	D	15.05.2014	S1124	13.8	21	Fam-62_1_201	S1124	1124	4024	62	2010	H
2915_1707_S1125	2014	D	15.05.2014	S1125	14	22	Fam-43_2009_	S1125	1125	4025	43	2009	F
2915_1707_S1126	2014	D	15.05.2014	S1126	14	23	Fam-41_2009_	S1126	1126	4026	41	2009	F
2915_1707_S1127	2014	D	15.05.2014	S1127	16.2	35.5	Fam-47_2009_	S1127	1127	4027	47	2009	F
2915_1708_S1128	2014	D	15.05.2014	S1128	13.8	21	Fam-32_2009_	S1128	1128	4028	32	2009	H
2915_1708_S1129	2014	D	15.05.2014	S1129	14.7	25	Fam-31_2009_	S1129	1129	4029	31	2009	F

2915_1708_S113(	2014	D	15.05.2014	S1130	14.8	25	Fam-68_2010_	S1130	1130	4030	68	2010 H
2915_1708_S1131	2014	D	15.05.2014	S1131	13.1	17	Fam-51_2009_	S1131	1131	4031	51	2009 W
2915_1708_S1132	2014	D	15.05.2014	S1132	12	14	Fam-68_2010_	S1132	1132	4032	68	2010 H
2915_1708_S1133	2014	D	15.05.2014	S1133	13.7	20.5	Fam-54_2009_	S1133	1133	4033	54	2009 W
2915_1708_S1134	2014	D	15.05.2014	S1134	13.5	19	Fam-51_2009_	S1134	1134	4034	51	2009 W
2915_1708_S1135	2014	D	15.05.2014	S1135	14	21	Fam-32_2009_	S1135	1135	4035	32	2009 H
2915_1708_S1136	2014	D	15.05.2014	S1136	13.5	22	Fam-58_2009_	S1136	1136	4036	58	2009 W
2915_1708_S1137	2014	D	15.05.2014	S1137	13.4	19.5	Fam-32_2009_	S1137	1137	4037	32	2009 H
2915_1708_S1138	2014	D	15.05.2014	S1138	12.7	17	Fam-59_2009_	S1138	1138	4038	59	2009 W
2915_1708_S1139	2014	D	15.05.2014	S1139	16	31.5	Fam-51_2009_	S1139	1139	4039	51	2009 W
2915_1708_S114(	2014	D	15.05.2014	S1140	14.2	24	Fam-50_2009_	S1140	1140	4040	50	2009 H
2915_1708_S1141	2014	T	15.05.2014	S1141	13.2	18	Family-76_201	S1141	1141	4041	76	2010 H
2915_1708_S1142	2014	D	15.05.2014	S1142	13.5	20	Fam-74_2010_	S1142	1142	4042	74	2010 H
2915_1708_S1143	2014	D	15.05.2014	S1143	14	21	Fam-66_2010_	S1143	1143	4043	66	2010 H

2915_1708_S1144	2014	D	15.05.2014	S1144	13.4	19	Fam-32_2009_	S1144	1144	4044	32	2009	H
2915_1708_S1145	2014	D	16.05.2014	S1145	13.4	18.5	Fam-60_2009_	S1145	1145	4045	60	2009	W
2915_1708_S1146	2014	D	16.05.2014	S1146	14.5	24.5	Fam-59_2009_	S1146	1146	4046	59	2009	W
2915_1708_S1147	2014	D	16.05.2014	S1147	14	23	Fam-36_2009_	S1147	1147	4047	36	2009	H
2915_1708_S1148	2014	D	16.05.2014	S1148	14.2	23	Fam-68_2010_	S1148	1148	4048	68	2010	H
2915_1708_S1149	2014	D	16.05.2014	S1149	16.1	31	Fam-57_2009_	S1149	1149	4049	57	2009	W
2915_1708_S1150	2014	D	16.05.2014	S1150	16.7	38.5	Fam-45_2009_	S1150	1150	4050	45	2009	F
2915_1708_S1151	2014	D	16.05.2014	S1151	14.8	28	Fam-87_2010_	S1151	1151	4051	87	2010	W
2915_1708_S1152	2014	D	16.05.2014	S1152	15.2	26.5	Fam-50_2009_	S1152	1152	4052	50	2009	H
2915_1708_S1153	2014	D	16.05.2014	S1153	14.9	25.5	Fam-51_2009_	S1153	1153	4053	51	2009	W
2915_1708_S1154	2014	D	16.05.2014	S1154	15.4	29	Fam-57_2009_	S1154	1154	4054	57	2009	W
2915_1708_S1155	2014	D	16.05.2014	S1155	14	21	Fam-89_2010_	S1155	1155	4055	89	2010	W
2915_1708_S1156	2014	D	16.05.2014	S1156	15.2	30	Fam-42_2009_	S1156	1156	4056	42	2009	H
2915_1708_S1157	2014	D	16.05.2014	S1157	15.5	31	Fam-58_2009_	S1157	1157	4057	58	2009	W

2915_1708_S1158	2014	D	16.05.2014	S1158	14.7	24	Fam-40_2009_	S1158	1158	4058	40	2009	H
2915_1708_S1159	2014	D	16.05.2014	S1159	14.1	22	Fam-59_2009_	S1159	1159	4059	59	2009	W
2915_1708_S1160	2014	D	16.05.2014	S1160	14.2	26	Fam-68_2010_	S1160	1160	4060	68	2010	H
2915_1708_S1161	2014	D	16.05.2014	S1161	15.5	30	Fam-42_2009_	S1161	1161	4061	42	2009	H
2915_1708_S1162	2014	D	16.05.2014	S1162	13.2	19.5	Fam-85_2010_	S1162	1162	4062	85	2010	W
2915_1708_S1163	2014	D	16.05.2014	S1163	13.2	18.5	Fam-55_2009_	S1163	1163	4063	55	2009	W
2915_1708_S1164	2014	D	16.05.2014	S1164	14	22	Fam-35_2009_	S1164	1164	4064	35	2009	F
2915_1708_S1165	2014	D	16.05.2014	S1165	14	22	Fam-82_2010_	S1165	1165	4065	82	2010	W
2915_1708_S1166	2014	D	16.05.2014	S1166	14	23.5	Fam-39_2009_	S1166	1166	4066	39	2009	F
2915_1708_S1167	2014	D	16.05.2014	S1167	14	21.5	Fam-67_2010_	S1167	1167	4067	67	2010	F
2915_1708_S1168	2014	D	16.05.2014	S1168	13.2	20	Fam-40_2009_	S1168	1168	4068	40	2009	H
2915_1708_S1170	2014	D	16.05.2014	S1170	13.4	20	Fam-38_2009_	S1170	1170	4069	38	2009	H
2915_1708_S1171	2014	D	16.05.2014	S1171	13.8	21	Fam-55_2009_	S1171	1171	4070	55	2009	W
2915_1708_S1172	2014	D	16.05.2014	S1172	13.8	20	Fam-51_2009_	S1172	1172	4071	51	2009	W

2915_1708_S1173	2014	D	16.05.2014	S1173	14	21.5	Fam-51_2009_	S1173	1173	4072	51	2009	W
2915_1708_S1174	2014	D	16.05.2014	S1174	13.7	20	Fam-52_2009_	S1174	1174	4073	52	2009	W
2915_1708_S1175	2014	D	16.05.2014	S1175	15.5	28	Fam-43_2009_	S1175	1175	4074	43	2009	F
2915_1708_S1176	2014	D	16.05.2014	S1176	12.8	16	Fam-73_2010_	S1176	1176	4075	73	2010	F
2915_1708_S1178	2014	D	16.05.2014	S1178	14.5	24.5	Fam-52_2009_	S1178	1178	4076	52	2009	W
2915_1708_S1179	2014	D	16.05.2014	S1179	12.6	15.5	Fam-59_2009_	S1179	1179	4077	59	2009	W
2915_1708_S1180	2014	D	16.05.2014	S1180	15.9	32	Fam-34_2009_	S1180	1180	4078	34	2009	H
2915_1708_S1181	2014	D	16.05.2014	S1181	13.7	20.5	Fam-59_2009_	S1181	1181	4079	59	2009	W
2915_1708_S1182	2014	D	16.05.2014	S1182	12.5	15	Fam-51_2009_	S1182	1182	4080	51	2009	W
2915_1708_S1183	2014	D	16.05.2014	S1183	13.8	21	Fam-80_2010_	S1183	1183	4081	80	2010	H
2915_1708_S1184	2014	D	16.05.2014	S1184	14.7	25	Fam-57_2009_	S1184	1184	4082	57	2009	W
2915_1708_S1185	2014	D	16.05.2014	S1185	14.3	23	Fam-74_2010_	S1185	1185	4083	74	2010	H
2915_1708_S1186	2014	D	16.05.2014	S1186	12.4	15.5	Fam-59_2009_	S1186	1186	4084	59	2009	W
2915_1708_S1187	2014	D	16.05.2014	S1187	13.5	20	Fam-54_2009_	S1187	1187	4085	54	2009	W

2915_1708_S1188	2014	D	16.05.2014	S1188	15.3	28	Fam-57_2009_	S1188	1188	4086	57	2009	W
2915_1708_S1189	2014	D	16.05.2014	S1189	15.1	27	Fam-57_2009_	S1189	1189	4087	57	2009	W
2915_1708_S1190	2014	D	16.05.2014	S1190	13.5	21	Fam-54_2009_	S1190	1190	4088	54	2009	W
2915_1708_S1191	2014	D	16.05.2014	S1191	14.2	22	Fam-57_2009_	S1191	1191	4089	57	2009	W
2915_1708_S1192	2014	D	16.05.2014	S1192	16	33.5	Fam-69_2010_	S1192	1192	4090	69	2010	F
2915_1708_S1193	2014	D	16.05.2014	S1193	13.5	20	Fam-40_2009_	S1193	1193	4091	40	2009	H
2915_1708_S1194	2014	D	16.05.2014	S1194	15.8	31	Fam-50_2009_	S1194	1194	4092	50	2009	H
2915_1708_S1195	2014	D	16.05.2014	S1195	14.6	23	Fam-89_2010_	S1195	1195	4093	89	2010	W
2915_1708_S1196	2014	D	16.05.2014	S1196	14.5	25	Fam-44_2009_	S1196	1196	4094	44	2009	H
2915_1708_S1197	2014	D	16.05.2014	S1197	15	27.5	Fam-52_2009_	S1197	1197	4095	52	2009	W
2915_1708_S1198	2014	D	16.05.2014	S1198	12.4	15.5	Fam-63_2010_	S1198	1198	4096	63	2010	F
2915_1708_S1199	2014	D	16.05.2014	S1199	13.1	18	Fam-76_2010_	S1199	1199	4097	76	2010	H
2915_1708_S1200	2014	D	16.05.2014	S1200	12	15	Fam-42_2009_	S1200	1200	4098	42	2009	H
2916_1708_S1201	2014	D	16.05.2014	S1201	13.7	24	Fam-35_2009_	S1201	1201	4099	35	2009	F

2916_1708_S1202	2014	D	16.05.2014	S1202	15.3	27.5	Fam-54_2009_	S1202	1202	4100	54	2009	W
2916_1708_S1203	2014	D	16.05.2014	S1203	13.9	22	Fam-90_2010_	S1203	1203	4101	90	2010	W
2916_1708_S1205	2014	D	16.05.2014	S1205	13.8	23	Fam-47_2009_	S1205	1205	4102	47	2009	F
2916_1708_S1207	2014	D	16.05.2014	S1207	14.1	22	Fam-32_2009_	S1207	1207	4103	32	2009	H
2916_1708_S1208	2014	D	16.05.2014	S1208	14.2	23.5	Fam-89_2010_	S1208	1208	4104	89	2010	W
2916_1708_S1209	2014	D	16.05.2014	S1209	13.9	22.5	Fam-45_2009_	S1209	1209	4105	45	2009	F
2916_1708_S1210	2014	D	16.05.2014	S1210	14.8	26	Fam-57_2009_	S1210	1210	4106	57	2009	W
2916_1708_S1211	2014	D	16.05.2014	S1211	15.1	27	Fam-48_2009_	S1211	1211	4107	48	2009	H
2916_1708_S1212	2014	D	16.05.2014	S1212	14	24.5	Fam-47_2009_	S1212	1212	4108	47	2009	F
2916_1708_S1213	2014	D	16.05.2014	S1213	12.6	16	Fam-55_2009_	S1213	1213	4109	55	2009	W
2916_1708_S1214	2014	D	16.05.2014	S1214	13.6	20.5	Fam-54_2009_	S1214	1214	4110	54	2009	W
2916_1708_S1215	2014	D	16.05.2014	S1215	12.8	18	Fam-69_2010_	S1215	1215	4111	69	2010	F
2916_1708_S1216	2014	D	16.05.2014	S1216	14.1	21	Fam-34_2009_	S1216	1216	4112	34	2009	H
2916_1708_S1217	2014	D	16.05.2014	S1217	15.3	28	Fam-51_2009_	S1217	1217	4113	51	2009	W

2916_1708_S1218	2014	D	16.05.2014	S1218	15.5	35	Fam-55_2009_	S1218	1218	4114	55	2009	W
2916_1708_S1219	2014	D	16.05.2014	S1219	14.8	27	Fam-52_2009_	S1219	1219	4115	52	2009	W
2916_1708_S1220	2014	D	16.05.2014	S1220	13.2	25	Fam-80_2010_	S1220	1220	4116	80	2010	H
2916_1708_S1221	2014	D	16.05.2014	S1221	16.4	38.5	Fam-31_2009_	S1221	1221	4117	31	2009	F
2916_1709_S1222	2014	D	16.05.2014	S1222	16.1	35	Fam-38_2009_	S1222	1222	4118	38	2009	H
2916_1709_S1223	2014	D	16.05.2014	S1223	14.1	23	Fam-52_2009_	S1223	1223	4119	52	2009	W
2916_1709_S1224	2014	D	16.05.2014	S1224	15.2	28.5	Fam-40_2009_	S1224	1224	4120	40	2009	H
2916_1709_S1225	2014	D	16.05.2014	S1225	15.2	28	Fam-42_2009_	S1225	1225	4121	42	2009	H
2916_1709_S1226	2014	D	16.05.2014	S1226	15	27	Fam-89_2010_	S1226	1226	4122	89	2010	W
2916_1709_S1227	2014	D	16.05.2014	S1227	14.2	23	Fam-52_2009_	S1227	1227	4123	52	2009	W
2916_1709_S1228	2014	D	16.05.2014	S1228	15	28	Fam-51_2009_	S1228	1228	4124	51	2009	W
2916_1709_S1229	2014	D	16.05.2014	S1229	13.6	19	Fam-59_2009_	S1229	1229	4125	59	2009	W
2916_1709_S1230	2014	D	16.05.2014	S1230	14.5	27	Fam-58_2009_	S1230	1230	4126	58	2009	W
2916_1709_S1231	2014	D	16.05.2014	S1231	14.3	25	Fam-58_2009_	S1231	1231	4127	58	2009	W

2916_1709_S1232	2014	D	16.05.2014	S1232	12.7	17	Fam-54_2009_	S1232	1232	4128	54	2009	W
2916_1709_S1233	2014	D	16.05.2014	S1233	13.9	22	Fam-75_2010_	S1233	1233	4129	75	2010	F
2916_1709_S1234	2014	D	16.05.2014	S1234	13.8	22	Fam-52_2009_	S1234	1234	4130	52	2009	W
2916_1709_S1235	2014	D	16.05.2014	S1235	14.9	30	Fam-54_2009_	S1235	1235	4131	54	2009	W
2916_1709_S1236	2014	D	16.05.2014	S1236	15.5	30	Fam-57_2009_	S1236	1236	4132	57	2009	W
2916_1709_S1237	2014	D	16.05.2014	S1237	15.2	29	Fam-57_2009_	S1237	1237	4133	57	2009	W
2916_1709_S1238	2014	D	16.05.2014	S1238	14.5	24	Fam-57_2009_	S1238	1238	4134	57	2009	W
2916_1709_S1239	2014	D	16.05.2014	S1239	15.1	28	Fam-34_2009_	S1239	1239	4135	34	2009	H
2916_1709_S1240	2014	D	16.05.2014	S1240	14.3	25	Fam-89_2010_	S1240	1240	4136	89	2010	W
2916_1709_S1242	2014	D	16.05.2014	S1242	13.8	21.5	Fam-83_2010_	S1242	1242	4137	83	2010	W
2916_1709_S1245	2014	D	16.05.2014	S1245	15.9	30	Fam-40_2009_	S1245	1245	4138	40	2009	H
2916_1709_S1246	2014	D	16.05.2014	S1246	13.2	20	Fam-60_2009_	S1246	1246	4139	60	2009	W
2916_1709_S1247	2014	D	16.05.2014	S1247	14.1	22.5	Fam-59_2009_	S1247	1247	4140	59	2009	W
2916_1709_S1248	2014	D	16.05.2014	S1248	14	22	Fam-32_2009_	S1248	1248	4141	32	2009	H

2916_1709_S1249	2014	D	16.05.2014	S1249	12	14	Fam-62_1_201	S1249	1249	4142	62	2010	H
2916_1709_S1250	2014	D	16.05.2014	S1250	13.7	21.5	Fam-54_2009_	S1250	1250	4143	54	2009	W
2916_1709_S1251	2014	D	16.05.2014	S1251	13.2	19	Fam-39_2009_	S1251	1251	4144	39	2009	F
2916_1709_S1252	2014	D	16.05.2014	S1252	15	31	Fam-54_2009_	S1252	1252	4145	54	2009	W
2916_1709_S1253	2014	D	16.05.2014	S1253	12.7	17	Fam-32_2009_	S1253	1253	4146	32	2009	H
2916_1709_S1254	2014	D	16.05.2014	S1254	13.7	21	Fam-82_2010_	S1254	1254	4147	82	2010	W
2916_1709_S1255	2014	D	16.05.2014	S1255	13.5	20.5	Fam-90_2010_	S1255	1255	4148	90	2010	W
2916_1709_S1256	2014	D	16.05.2014	S1256	15.5	29	Fam-40_2009_	S1256	1256	4149	40	2009	H
2916_1709_S1257	2014	D	16.05.2014	S1257	15.5	30	Fam-31_2009_	S1257	1257	4150	31	2009	F
2916_1709_S1258	2014	D	16.05.2014	S1258	15.3	26	Fam-82_2010_	S1258	1258	4151	82	2010	W
2916_1709_S1259	2014	D	16.05.2014	S1259	14.6	22.5	Fam-59_2009_	S1259	1259	4152	59	2009	W
2916_1709_S1260	2014	D	16.05.2014	S1260	15.1	27	Fam-60_2009_	S1260	1260	4153	60	2009	W
2916_1709_S1261	2014	D	16.05.2014	S1261	14.2	24	Fam-31_2009_	S1261	1261	4154	31	2009	F
2916_1709_S1262	2014	D	16.05.2014	S1262	41.3	22.5	Fam-53_2009_	S1262	1262	4155	53	2009	W

2916_1709_S1263	2014	D	16.05.2014	S1263	14	22	Fam-34_2009_	S1263	1263	4156	34	2009	H
2916_1709_S1265	2014	D	16.05.2014	S1265	13.2	19	Fam-54_2009_	S1265	1265	4157	54	2009	W
2916_1709_S1266	2014	D	16.05.2014	S1266	12.8	17	Fam-55_2009_	S1266	1266	4158	55	2009	W
2916_1709_S1269	2014	D	16.05.2014	S1269	14.2	22	Fam-34_2009_	S1269	1269	4159	34	2009	H
2916_1709_S1270	2014	D	16.05.2014	S1270	14	23	Fam-56_2009_	S1270	1270	4160	56	2009	W
2916_1709_S1271	2014	D	16.05.2014	S1271	12.1	14	Fam-59_2009_	S1271	1271	4161	59	2009	W
2916_1709_S1272	2014	D	16.05.2014	S1272	15	27.5	Fam-60_2009_	S1272	1272	4162	60	2009	W
2916_1709_S1273	2014	D	16.05.2014	S1273	14	23	Fam-42_2009_	S1273	1273	4163	42	2009	H
2916_1709_S1274	2014	D	16.05.2014	S1274	15.3	28	Fam-32_2009_	S1274	1274	4164	32	2009	H
2916_1709_S1275	2014	D	16.05.2014	S1275	13.9	20	Fam-64_2010_	S1275	1275	4165	64	2010	H
2916_1709_S1276	2014	D	16.05.2014	S1276	14.3	26	Fam-58_2009_	S1276	1276	4166	58	2009	W
2916_1709_S1277	2014	D	16.05.2014	S1277	15.2	29	Fam-51_2009_	S1277	1277	4167	51	2009	W
2916_1709_S1278	2014	D	16.05.2014	S1278	14.5	25	Fam-51_2009_	S1278	1278	4168	51	2009	W
2916_1709_S1279	2014	D	16.05.2014	S1279	14	21.5	Fam-51_2009_	S1279	1279	4169	51	2009	W

2916_1709_S1280	2014	D	16.05.2014	S1280	14.9	27	Fam-40_2009_	S1280	1280	4170	40	2009	H
2917_1709_S1281	2014	D	16.05.2014	S1281	14.1	24	Fam-32_2009_	S1281	1281	4171	32	2009	H
2917_1709_S1282	2014	D	16.05.2014	S1282	13.6	20.5	Fam-52_2009_	S1282	1282	4172	52	2009	W
2917_1709_S1283	2014	D	16.05.2014	S1283	12.5	17	Fam-59_2009_	S1283	1283	4173	59	2009	W
2917_1709_S1284	2014	D	16.05.2014	S1284	14	24.5	Fam-58_2009_	S1284	1284	4174	58	2009	W
2917_1709_S1285	2014	D	16.05.2014	S1285	14.9	26.5	Fam-51_2009_	S1285	1285	4175	51	2009	W
2917_1709_S1286	2014	D	16.05.2014	S1286	16.7	37.5	Fam-57_2009_	S1286	1286	4176	57	2009	W
2917_1709_S1287	2014	D	16.05.2014	S1287	13.2	19	Fam-40_2009_	S1287	1287	4177	40	2009	H
2917_1709_S1288	2014	D	16.05.2014	S1288	13.7	21	Fam-50_2009_	S1288	1288	4178	50	2009	H
2917_1709_S1289	2014	D	16.05.2014	S1289	13.6	22	Fam-58_2009_	S1289	1289	4179	58	2009	W
2917_1709_S1290	2014	D	16.05.2014	S1290	15	27	Fam-75_2010_	S1290	1290	4180	75	2010	F
2917_1709_S1291	2014	D	16.05.2014	S1291	14.9	29	Fam-42_2009_	S1291	1291	4181	42	2009	H
2917_1709_S1292	2014	D	16.05.2014	S1292	13.2	18.5	Fam-57_2009_	S1292	1292	4182	57	2009	W
2917_1709_S1293	2014	D	16.05.2014	S1293	12.4	18.5	Fam-65_2010_	S1293	1293	4183	65	2010	F

2917_1709_S1294	2014	D	16.05.2014	S1294	14.4	23.5	Fam-32_2009_	S1294	1294	4184	32	2009	H
2917_1709_S1295	2014	D	16.05.2014	S1295	14.6	27	Fam-54_2009_	S1295	1295	4185	54	2009	W
2917_1709_S1296	2014	D	16.05.2014	S1296	12.2	16	Fam-34_2009_	S1296	1296	4186	34	2009	H
2917_1709_S1297	2014	D	16.05.2014	S1297	12.6	16.5	Fam-52_2009_	S1297	1297	4187	52	2009	W
2917_1709_S1298	2014	D	16.05.2014	S1298	15.6	27.5	Fam-39_2009_	S1298	1298	4188	39	2009	F
2917_1709_S1299	2014	D	16.05.2014	S1299	13.7	22.5	Fam-50_2009_	S1299	1299	4189	50	2009	H
2917_1709_S1300	2014	D	16.05.2014	S1300	14.1	22.5	Fam-51_2009_	S1300	1300	4190	51	2009	W
2917_1709_S1302	2014	D	16.05.2014	S1302	14.3	24	Fam-51_2009_	S1302	1302	4191	51	2009	W
2917_1709_S1303	2014	D	16.05.2014	S1303	12.7	17.5	Fam-59_2009_	S1303	1303	4192	59	2009	W
2917_1709_S1304	2014	D	16.05.2014	S1304	14.3	24	Fam-58_2009_	S1304	1304	4193	58	2009	W
2917_1709_S1305	2014	D	16.05.2014	S1305	15	26.5	Fam-51_2009_	S1305	1305	4194	51	2009	W
2917_1709_S1307	2014	D	16.05.2014	S1307	13.5	21	Fam-59_2009_	S1307	1307	4195	59	2009	W
2917_1709_S1308	2014	D	16.05.2014	S1308	14.9	28	Fam-48_2009_	S1308	1308	4196	48	2009	H
2917_1709_S1309	2014	D	16.05.2014	S1309	14.7	27	Fam-59_2009_	S1309	1309	4197	59	2009	W

2917_1709_S1310	2014	D	16.05.2014	S1310	14.2	23.5	Fam-59_2009_	S1310	1310	4198	59	2009	W
2917_1709_S1311	2014	D	16.05.2014	S1311	15	28	Fam-55_2009_	S1311	1311	4199	55	2009	W
2917_1709_S1312	2014	D	16.05.2014	S1312	16.2	35.5	Fam-60_2009_	S1312	1312	4200	60	2009	W
2917_1709_S1313	2014	D	16.05.2014	S1313	14.3	26	Fam-54_2009_	S1313	1313	4201	54	2009	W
2917_1709_S1314	2014	D	16.05.2014	S1314	15.9	32	Fam-66_2010_	S1314	1314	4202	66	2010	H
2917_1709_S1315	2014	D	16.05.2014	S1315	14.9	29	Fam-42_2009_	S1315	1315	4203	42	2009	H
2917_1710_S1316	2014	D	16.05.2014	S1316	14.4	25	Fam-48_2009_	S1316	1316	4204	48	2009	H
2917_1710_S1317	2014	D	16.05.2014	S1317	12.6	16.5	Fam-64_2010_	S1317	1317	4205	64	2010	H
2917_1710_S1318	2014	D	16.05.2014	S1318	13.7	22	Fam-44_2009_	S1318	1318	4206	44	2009	H
2917_1710_S1319	2014	D	16.05.2014	S1319	13	17	Fam-54_2009_	S1319	1319	4207	54	2009	W
2917_1710_S1320	2014	D	16.05.2014	S1320	12.9	18.5	Fam-34_2009_	S1320	1320	4208	34	2009	H
2917_1710_S1321	2014	D	16.05.2014	S1321	13.9	20.5	Fam-54_2009_	S1321	1321	4209	54	2009	W
2917_1710_S1322	2014	D	16.05.2014	S1322	13.5	19.5	Fam-72_2010_	S1322	1322	4210	72	2010	H
2917_1710_S1323	2014	D	16.05.2014	S1323	13.4	19.5	Fam-53_2009_	S1323	1323	4211	53	2009	W

2917_1710_S1324	2014	D	16.05.2014	S1324	13.5	19.5	Fam-54_2009_	S1324	1324	4212	54	2009	W
2917_1710_S1325	2014	D	16.05.2014	S1325	12.6	16	Fam-62_1_201	S1325	1325	4213	62	2010	H
2917_1710_S1326	2014	D	16.05.2014	S1326	14.5	26.5	Fam-34_2009_	S1326	1326	4214	34	2009	H
2917_1710_S1327	2014	D	16.05.2014	S1327	13.9	23	Fam-42_2009_	S1327	1327	4215	42	2009	H
2917_1710_S1328	2014	D	16.05.2014	S1328	15	29	Fam-85_2010_	S1328	1328	4216	85	2010	W
2917_1710_S1329	2014	D	16.05.2014	S1329	13.2	19	Fam-59_2009_	S1329	1329	4217	59	2009	W
2917_1710_S1330	2014	D	16.05.2014	S1330	15.1	28	Fam-67_2010_	S1330	1330	4218	67	2010	F
2917_1710_S1331	2014	D	16.05.2014	S1331	14	20	Fam-32_2009_	S1331	1331	4219	32	2009	H
2917_1710_S1332	2014	D	16.05.2014	S1332	14.7	25.5	Fam-35_2009_	S1332	1332	4220	35	2009	F
2917_1710_S1333	2014	D	16.05.2014	S1333	14	22.5	Fam-32_2009_	S1333	1333	4221	32	2009	H
2917_1710_S1334	2014	D	16.05.2014	S1334	13.5	19.5	Fam-85_2010_	S1334	1334	4222	85	2010	W
2917_1710_S1335	2014	D	16.05.2014	S1335	14	22	Fam-78_2010_	S1335	1335	4223	78	2010	H
2917_1710_S1336	2014	D	16.05.2014	S1336	13.7	20.5	Fam-81_2010_	S1336	1336	4224	81	2010	W
2917_1710_S1337	2014	D	16.05.2014	S1337	15	26	Fam-51_2009_	S1337	1337	4225	51	2009	W

2917_1710_S1338	2014	D	16.05.2014	S1338	11.2	11.5	Fam-54_2009_	S1338	1338	4226	54	2009	W
2917_1710_S1339	2014	D	16.05.2014	S1339	13.2	18.5	Fam-59_2009_	S1339	1339	4227	59	2009	W
2917_1710_S1340	2014	D	16.05.2014	S1340	15.8	31	Fam-57_2009_	S1340	1340	4228	57	2009	W
2917_1710_S1341	2014	D	16.05.2014	S1341	15	25	Fam-57_2009_	S1341	1341	4229	57	2009	W
2917_1710_S1342	2014	D	16.05.2014	S1342	14	22	Fam-57_2009_	S1342	1342	4230	57	2009	W
2917_1710_S1343	2014	D	16.05.2014	S1343	16.5	37.5	Fam-49_2009_	S1343	1343	4231	49	2009	F
2917_1710_S1344	2014	D	16.05.2014	S1344	12.8	17	Fam-54_2009_	S1344	1344	4232	54	2009	W
2917_1710_S1345	2014	D	16.05.2014	S1345	14	24.5	Fam-53_2009_	S1345	1345	4233	53	2009	W
2917_1710_S1346	2014	D	16.05.2014	S1346	14.5	25.5	Fam-54_2009_	S1346	1346	4234	54	2009	W
2917_1710_S1347	2014	D	16.05.2014	S1347	13.5	20.5	Fam-54_2009_	S1347	1347	4235	54	2009	W
2917_1710_S1348	2014	D	16.05.2014	S1348	12.5	16.5	Fam-52_2009_	S1348	1348	4236	52	2009	W
2917_1710_S1349	2014	D	16.05.2014	S1349	14.7	26.5	Fam-78_2010_	S1349	1349	4237	78	2010	H
2917_1710_S1350	2014	D	16.05.2014	S1350	13.2	18	Fam-59_2009_	S1350	1350	4238	59	2009	W
2917_1710_S1351	2014	D	16.05.2014	S1351	14.7	24	Fam-28_2007_	S1351	1351	4239	28	2007	H

2917_1710_S1352	2014	D	16.05.2014	S1352	12.8	16.5	Fam-59_2009_S1352	1352	4240	59	2009	W
2917_1710_S1353	2014	D	16.05.2014	S1353	14	22.5	Fam-54_2009_S1353	1353	4241	54	2009	W
2917_1710_S1354	2014	D	16.05.2014	S1354	15.5	31.5	Fam-55_2009_S1354	1354	4242	55	2009	W
2917_1710_S1355	2014	D	16.05.2014	S1355	13.5	18.5	Fam-52_2009_S1355	1355	4243	52	2009	W
2917_1710_S1356	2014	D	16.05.2014	S1356	14.3	22.5	Fam-85_2010_S1356	1356	4244	85	2010	W
2917_1710_S1358	2014	D	16.05.2014	S1358	15.5	28.5	Fam-60_2009_S1358	1358	4245	60	2009	W
2917_1710_S1360	2014	D	16.05.2014	S1360	15.5	28	Fam-52_2009_S1360	1360	4246	52	2009	W
2918_1710_S1361	2014	D	16.05.2014	S1361	14.5	24	Fam-58_2009_S1361	1361	4247	58	2009	W
2918_1710_S1362	2014	D	16.05.2014	S1362	15.5	30	Fam-51_2009_S1362	1362	4248	51	2009	W
2918_1710_S1363	2014	D	16.05.2014	S1363	13.8	22	Fam-64_2010_S1363	1363	4249	64	2010	H
2918_1710_S1364	2014	D	16.05.2014	S1364	12.7	16.5	Fam-80_2010_S1364	1364	4250	80	2010	H
2918_1710_S1366	2014	D	16.05.2014	S1366	14.2	23.5	Fam-85_2010_S1366	1366	4251	85	2010	W
2918_1710_S1367	2014	D	16.05.2014	S1367	15	27.5	Fam-57_2009_S1367	1367	4252	57	2009	W
2918_1710_S1368	2014	D	16.05.2014	S1368	13.4	21	Fam-59_2009_S1368	1368	4253	59	2009	W

2918_1710_S1369	2014	T	16.05.2014	S1369	14.1	22	Family-49_200	S1369	1369	4254	49	2009	F
2918_1710_S1370	2014	D	16.05.2014	S1370	14.1	25	Fam-42_2009_	S1370	1370	4255	42	2009	H
2918_1710_S1371	2014	D	16.05.2014	S1371	14	23	Fam-54_2009_	S1371	1371	4256	54	2009	W
2918_1710_S1372	2014	D	16.05.2014	S1372	14.3	24	Fam-57_2009_	S1372	1372	4257	57	2009	W
2918_1710_S1373	2014	D	16.05.2014	S1373	13.3	19.5	Fam-85_2010_	S1373	1373	4258	85	2010	W
2918_1710_S1374	2014	D	16.05.2014	S1374	13.1	20.5	Fam-58_2009_	S1374	1374	4259	58	2009	W
2918_1710_S1375	2014	D	16.05.2014	S1375	13	20	Fam-56_2009_	S1375	1375	4260	56	2009	W
2918_1710_S1377	2014	D	16.05.2014	S1377	14.8	26.5	Fam-58_2009_	S1377	1377	4261	58	2009	W
2918_1710_S1378	2014	D	16.05.2014	S1378	14.1	24.5	Fam-51_2009_	S1378	1378	4262	51	2009	W
2918_1710_S1379	2014	D	16.05.2014	S1379	15.2	29	Fam-58_2009_	S1379	1379	4263	58	2009	W
2918_1710_S1380	2014	D	16.05.2014	S1380	12.4	17	Fam-59_2009_	S1380	1380	4264	59	2009	W
2918_1710_S1381	2014	D	16.05.2014	S1381	15	28.5	Fam-53_2009_	S1381	1381	4265	53	2009	W
2918_1710_S1382	2014	D	16.05.2014	S1382	13.6	25	Fam-45_2009_	S1382	1382	4266	45	2009	F
2918_1710_S1383	2014	D	16.05.2014	S1383	12.7	17	Fam-58_2009_	S1383	1383	4267	58	2009	W

2918_1710_S1384	2014	D	16.05.2014	S1384	15.9	33.5	Fam-40_2009_	S1384	1384	4268	40	2009	H
2918_1710_S1386	2014	D	16.05.2014	S1386	13	18.5	Fam-58_2009_	S1386	1386	4269	58	2009	W
2918_1710_S1387	2014	D	16.05.2014	S1387	13.2	19.5	Fam-31_2009_	S1387	1387	4270	31	2009	F
2918_1710_S1388	2014	D	16.05.2014	S1388	14.1	24	Fam-57_2009_	S1388	1388	4271	57	2009	W
2918_1710_S1389	2014	D	16.05.2014	S1389	16.3	37	Fam-10_2007_	S1389	1389	4272	10	2007	W
2918_1710_S1390	2014	D	16.05.2014	S1390	14.9	27	Fam-87_2010_	S1390	1390	4273	87	2010	W
2918_1710_S1391	2014	D	16.05.2014	S1391	14.1	26.5	Fam-90_2010_	S1391	1391	4274	90	2010	W
2918_1710_S1392	2014	D	16.05.2014	S1392	16.6	38	Fam-66_2010_	S1392	1392	4275	66	2010	H
2918_1710_S1393	2014	D	16.05.2014	S1393	14.1	23.5	Fam-55_2009_	S1393	1393	4276	55	2009	W
2918_1710_S1394	2014	D	16.05.2014	S1394	13.7	23	Fam-58_2009_	S1394	1394	4277	58	2009	W
2918_1710_S1395	2014	D	16.05.2014	S1395	14.4	24.5	Fam-59_2009_	S1395	1395	4278	59	2009	W
2918_1710_S1396	2014	D	16.05.2014	S1396	13	19	Fam-59_2009_	S1396	1396	4279	59	2009	W
2918_1710_S1397	2014	D	16.05.2014	S1397	13.2	21.5	Fam-87_2010_	S1397	1397	4280	87	2010	W
2918_1710_S1398	2014	D	16.05.2014	S1398	14	22.5	Fam-57_2009_	S1398	1398	4281	57	2009	W

2918_1710_S1399	2014	D	16.05.2014	S1399	12.9	18.5	Fam-52_2009_	S1399	1399	4282	52	2009	W
2918_1710_S1400	2014	D	16.05.2014	S1400	14.3	24	Fam-40_2009_	S1400	1400	4283	40	2009	H
2918_1710_S1401	2014	D	16.05.2014	S1401	14.1	24	Fam-62_1_201	S1401	1401	4284	62	2010	H
2918_1710_S1403	2014	D	16.05.2014	S1403	12.6	16.5	Fam-59_2009_	S1403	1403	4285	59	2009	W
2918_1710_S1405	2014	D	16.05.2014	S1405	13.7	21.5	Fam-58_2009_	S1405	1405	4286	58	2009	W
2918_1710_S1407	2014	D	16.05.2014	S1407	12.8	18	Fam-89_2010_	S1407	1407	4287	89	2010	W
2918_1710_S1408	2014	D	16.05.2014	S1408	13.1	20	Fam-52_2009_	S1408	1408	4288	52	2009	W
2918_1710_S1409	2014	D	18.05.2014	S1409	14.6	26	Fam-48_2009_	S1409	1409	4289	48	2009	H
2918_1711_S1410	2014	D	18.05.2014	S1410	14.5	24	Fam-53_2009_	S1410	1410	4290	53	2009	W
2918_1711_S1411	2014	D	18.05.2014	S1411	15	27.5	Fam-40_2009_	S1411	1411	4291	40	2009	H
2918_1711_S1412	2014	D	18.05.2014	S1412	13.6	21	Fam-42_2009_	S1412	1412	4292	42	2009	H
2918_1711_S1413	2014	D	18.05.2014	S1413	13.7	21	Fam-59_2009_	S1413	1413	4293	59	2009	W
2918_1711_S1415	2014	D	18.05.2014	S1415	12.5	17.5	Fam-42_2009_	S1415	1415	4294	42	2009	H
2918_1711_S1416	2014	D	18.05.2014	S1416	15.2	27	Fam-51_2009_	S1416	1416	4295	51	2009	W

2918_1711_S1417	2014	D	18.05.2014	S1417	14.3	24.5	Fam-75_2010_	S1417	1417	4296	75	2010	F
2918_1711_S1418	2014	D	18.05.2014	S1418	14.8	24	Fam-50_2009_	S1418	1418	4297	50	2009	H
2918_1711_S1419	2014	D	18.05.2014	S1419	14.4	24	Fam-54_2009_	S1419	1419	4298	54	2009	W
2918_1711_S1420	2014	D	18.05.2014	S1420	13.5	20	Fam-36_2009_	S1420	1420	4299	36	2009	H
2918_1711_S1421	2014	D	18.05.2014	S1421	14.5	23.5	Fam-57_2009_	S1421	1421	4300	57	2009	W
2918_1711_S1422	2014	D	18.05.2014	S1422	15.5	31	Fam-57_2009_	S1422	1422	4301	57	2009	W
2918_1711_S1423	2014	D	18.05.2014	S1423	13.6	19.5	Fam-52_2009_	S1423	1423	4302	52	2009	W
2918_1711_S1424	2014	D	18.05.2014	S1424	13.8	21	Fam-76_2010_	S1424	1424	4303	76	2010	H
2918_1711_S1425	2014	D	18.05.2014	S1425	15.1	29	Fam-60_2009_	S1425	1425	4304	60	2009	W
2918_1711_S1426	2014	D	18.05.2014	S1426	14.3	26	Fam-54_2009_	S1426	1426	4305	54	2009	W
2918_1711_S1427	2014	D	18.05.2014	S1427	13.6	19.5	Fam-32_2009_	S1427	1427	4306	32	2009	H
2918_1711_S1428	2014	D	18.05.2014	S1428	15.3	29.5	Fam-57_2009_	S1428	1428	4307	57	2009	W
2918_1711_S1429	2014	D	18.05.2014	S1429	13.7	20	Fam-54_2009_	S1429	1429	4308	54	2009	W
2918_1711_S1430	2014	D	18.05.2014	S1430	14.3	25	Fam-42_2009_	S1430	1430	4309	42	2009	H

2918_1711_S1431	2014	D	18.05.2014	S1431	14.4	24	Fam-52_2009_	S1431	1431	4310	52	2009	W
2918_1711_S1433	2014	D	18.05.2014	S1433	15.9	34.5	Fam-42_2009_	S1433	1433	4311	42	2009	H
2918_1711_S1434	2014	D	18.05.2014	S1434	14.5	25.5	Fam-51_2009_	S1434	1434	4312	51	2009	W
2918_1711_S1435	2014	D	18.05.2014	S1435	13	18	Fam-57_2009_	S1435	1435	4313	57	2009	W
2918_1711_S1436	2014	D	18.05.2014	S1436	13.2	19	Fam-54_2009_	S1436	1436	4314	54	2009	W
2918_1711_S1437	2014	D	18.05.2014	S1437	14.7	25.5	Fam-54_2009_	S1437	1437	4315	54	2009	W
2918_1711_S1438	2014	D	18.05.2014	S1438	15	26	Fam-57_2009_	S1438	1438	4316	57	2009	W
2918_1711_S1439	2014	D	18.05.2014	S1439	14.6	26	Fam-55_2009_	S1439	1439	4317	55	2009	W
2918_1711_S1440	2014	D	18.05.2014	S1440	15	27.5	Fam-57_2009_	S1440	1440	4318	57	2009	W
2919_1711_S1441	2014	D	18.05.2014	S1441	15.4	28.5	Fam-57_2009_	S1441	1441	4319	57	2009	W
2919_1711_S1443	2014	D	18.05.2014	S1443	14.4	25	Fam-44_2009_	S1443	1443	4320	44	2009	H
2919_1711_S1444	2014	D	18.05.2014	S1444	12.5	16.5	Fam-59_2009_	S1444	1444	4321	59	2009	W
2919_1711_S1445	2014	D	18.05.2014	S1445	13	18	Fam-87_2010_	S1445	1445	4322	87	2010	W
2919_1711_S1446	2014	D	18.05.2014	S1446	14	23	Fam-73_2010_	S1446	1446	4323	73	2010	F

2919_1711_S1447	2014	D	18.05.2014	S1447	14.2	24	Fam-58_2009_	S1447	1447	4324	58	2009	W
2919_1711_S1448	2014	D	18.05.2014	S1448	14.8	28.5	Fam-58_2009_	S1448	1448	4325	58	2009	W
2919_1711_S1449	2014	D	18.05.2014	S1449	12.3	15.5	Fam-58_2009_	S1449	1449	4326	58	2009	W
2919_1711_S1450	2014	D	18.05.2014	S1450	12.2	15.5	Fam-44_2009_	S1450	1450	4327	44	2009	H
2919_1711_S1451	2014	D	18.05.2014	S1451	14	21.5	Fam-72_2010_	S1451	1451	4328	72	2010	H
2919_1711_S1452	2014	D	18.05.2014	S1452	15.2	29	Fam-54_2009_	S1452	1452	4329	54	2009	W
2919_1711_S1454	2014	D	18.05.2014	S1454	15.2	28	Fam-57_2009_	S1454	1454	4330	57	2009	W
2919_1711_S1455	2014	D	18.05.2014	S1455	13.3	19.5	Fam-58_2009_	S1455	1455	4331	58	2009	W
2919_1711_S1456	2014	D	18.05.2014	S1456	16.4	37.5	Fam-54_2009_	S1456	1456	4332	54	2009	W
2919_1711_S1457	2014	D	18.05.2014	S1457	12.9	18	Fam-90_2010_	S1457	1457	4333	90	2010	W
2919_1711_S1458	2014	D	18.05.2014	S1458	13.2	20	Fam-56_2009_	S1458	1458	4334	56	2009	W
2919_1711_S1459	2014	D	18.05.2014	S1459	12.5	16.5	Fam-54_2009_	S1459	1459	4335	54	2009	W
2919_1711_S1460	2014	D	18.05.2014	S1460	13.3	18.5	Fam-52_2009_	S1460	1460	4336	52	2009	W
2919_1711_S1461	2014	D	18.05.2014	S1461	13.7	21	Fam-34_2009_	S1461	1461	4337	34	2009	H

2919_1711_S1462	2014	D	18.05.2014	S1462	14	21.5	Fam-57_2009_	S1462	1462	4338	57	2009 W
2919_1711_S1463	2014	D	18.05.2014	S1463	14.5	26	Fam-43_2009_	S1463	1463	4339	43	2009 F
2919_1711_S1464	2014	D	18.05.2014	S1464	12.8	17.5	Fam-59_2009_	S1464	1464	4340	59	2009 W
2919_1711_S1465	2014	D	18.05.2014	S1465	13.7	21.5	Fam-60_2009_	S1465	1465	4341	60	2009 W
2919_1711_S1466	2014	D	18.05.2014	S1466	15.1	28.5	Fam-40_2009_	S1466	1466	4342	40	2009 H
2919_1711_S1467	2014	D	18.05.2014	S1467	15.2	29.5	Fam-42_2009_	S1467	1467	4343	42	2009 H
2919_1711_S1468	2014	D	18.05.2014	S1468	14.8	24.5	Fam-70_2010_	S1468	1468	4344	70	2010 H
2919_1711_S1469	2014	D	18.05.2014	S1469	14.5	26	Fam-06_2007_	S1469	1469	4345	6	2007 W
2919_1711_S1470	2014	D	18.05.2014	S1470	13.8	21	Fam-84_2010_	S1470	1470	4346	84	2010 W
2919_1711_S1471	2014	D	18.05.2014	S1471	13.8	20.5	Fam-32_2009_	S1471	1471	4347	32	2009 H
2919_1711_S1472	2014	D	18.05.2014	S1472	12.1	15	Fam-85_2010_	S1472	1472	4348	85	2010 W
2919_1711_S1473	2014	D	18.05.2014	S1473	14.1	23.5	Fam-60_2009_	S1473	1473	4349	60	2009 W
2919_1711_S1474	2014	D	18.05.2014	S1474	14.3	24.5	Fam-58_2009_	S1474	1474	4350	58	2009 W
2919_1711_S1476	2014	D	18.05.2014	S1476	14.5	26	Fam-56_2009_	S1476	1476	4351	56	2009 W

2919_1711_S1477	2014	D	18.05.2014	S1477	13.9	22.5	Fam-54_2009_	S1477	1477	4352	54	2009	W
2919_1711_S1478	2014	D	18.05.2014	S1478	16	32	Fam-50_2009_	S1478	1478	4353	50	2009	H
2919_1711_S1479	2014	D	18.05.2014	S1479	14.8	26.5	Fam-45_2009_	S1479	1479	4354	45	2009	F
2919_1711_S1480	2014	D	18.05.2014	S1480	13.1	18	Fam-59_2009_	S1480	1480	4355	59	2009	W
2919_1711_S1481	2014	D	18.05.2014	S1481	15.8	28.5	Fam-58_2009_	S1481	1481	4356	58	2009	W
2919_1711_S1482	2014	D	18.05.2014	S1482	17.7	45.5	Fam-76_2010_	S1482	1482	4357	76	2010	H
2919_1711_S1483	2014	D	18.05.2014	S1483	13.1	18	Fam-59_2009_	S1483	1483	4358	59	2009	W
2919_1711_S1484	2014	D	18.05.2014	S1484	13.5	20	Fam-59_2009_	S1484	1484	4359	59	2009	W
2919_1711_S1485	2014	D	18.05.2014	S1485	15.5	29	Fam-51_2009_	S1485	1485	4360	51	2009	W
2919_1711_S1486	2014	D	18.05.2014	S1486	13.1	18	Fam-55_2009_	S1486	1486	4361	55	2009	W
2919_1711_S1487	2014	D	18.05.2014	S1487	15	26	Fam-40_2009_	S1487	1487	4362	40	2009	H
2919_1711_S1488	2014	D	18.05.2014	S1488	14.6	27	Fam-67_2010_	S1488	1488	4363	67	2010	F
2919_1711_S1489	2014	D	18.05.2014	S1489	14.5	25	Fam-57_2009_	S1489	1489	4364	57	2009	W
2919_1711_S1490	2014	D	18.05.2014	S1490	13.6	21	Fam-59_2009_	S1490	1490	4365	59	2009	W

2919_1711_S1491	2014	D	18.05.2014	S1491	14	23	Fam-58_2009_	S1491	1491	4366	58	2009 W
2919_1711_S1492	2014	D	18.05.2014	S1492	15.1	28	Fam-47_2009_	S1492	1492	4367	47	2009 F
2919_1711_S1493	2014	D	18.05.2014	S1493	15.7	31.5	Fam-83_2010_	S1493	1493	4368	83	2010 W
2919_1711_S1494	2014	D	18.05.2014	S1494	14.7	26	Fam-56_2009_	S1494	1494	4369	56	2009 W
2919_1711_S1495	2014	D	18.05.2014	S1495	14.5	26.5	Fam-42_2009_	S1495	1495	4370	42	2009 H
2919_1711_S1496	2014	D	18.05.2014	S1496	14.8	24.5	Fam-55_2009_	S1496	1496	4371	55	2009 W
2919_1711_S1497	2014	D	18.05.2014	S1497	15.1	29.5	Fam-58_2009_	S1497	1497	4372	58	2009 W
2919_1711_S1498	2014	D	18.05.2014	S1498	13.5	19	Fam-59_2009_	S1498	1498	4373	59	2009 W
2919_1711_S1499	2014	D	18.05.2014	S1499	15.6	28.5	Fam-40_2009_	S1499	1499	4374	40	2009 H
2919_1711_S1500	2014	D	18.05.2014	S1500	16.2	33	Fam-57_2009_	S1500	1500	4375	57	2009 W
2919_1711_S1501	2014	D	18.05.2014	S1501	14.8	29	Fam-54_2009_	S1501	1501	4376	54	2009 W
2919_1711_S1502	2014	D	18.05.2014	S1502	14.8	26.5	Fam-59_2009_	S1502	1502	4377	59	2009 W
2919_1711_S1503	2014	D	18.05.2014	S1503	13.2	20	Fam-54_2009_	S1503	1503	4378	54	2009 W
2919_1722_S1504	2014	D	18.05.2014	S1504	15	22	Fam-59_2009_	S1504	1504	4379	59	2009 W

2919_1722_S1505	2014	D	18.05.2014	S1505	13	19	Fam-60_2009_	S1505	1505	4380	60	2009	W
2919_1722_S1506	2014	D	18.05.2014	S1506	12.2	15.5	Fam-54_2009_	S1506	1506	4381	54	2009	W
2919_1722_S1507	2014	D	18.05.2014	S1507	12.3	15.5	Fam-54_2009_	S1507	1507	4382	54	2009	W
2919_1722_S1508	2014	D	18.05.2014	S1508	14.5	24.5	Fam-55_2009_	S1508	1508	4383	55	2009	W
2919_1722_S1509	2014	D	18.05.2014	S1509	13.2	19	Fam-59_2009_	S1509	1509	4384	59	2009	W
2919_1722_S1511	2014	D	18.05.2014	S1511	15.5	30.5	Fam-55_2009_	S1511	1511	4385	55	2009	W
2919_1722_S1512	2014	D	18.05.2014	S1512	13.6	20.5	Fam-90_2010_	S1512	1512	4386	90	2010	W
2919_1722_S1513	2014	D	18.05.2014	S1513	14.5	25	Fam-54_2009_	S1513	1513	4387	54	2009	W
2919_1722_S1514	2014	D	18.05.2014	S1514	13	17.5	Fam-54_2009_	S1514	1514	4388	54	2009	W
2919_1722_S1515	2014	D	18.05.2014	S1515	12.7	17	Fam-44_2009_	S1515	1515	4389	44	2009	H
2919_1722_S1516	2014	D	18.05.2014	S1516	13.8	22	Fam-54_2009_	S1516	1516	4390	54	2009	W
2919_1722_S1517	2014	D	18.05.2014	S1517	14	23	Fam-58_2009_	S1517	1517	4391	58	2009	W
2919_1722_S1518	2014	D	18.05.2014	S1518	15.3	26.5	Fam-53_2009_	S1518	1518	4392	53	2009	W
2919_1722_S1519	2014	D	18.05.2014	S1519	15.6	31	Fam-58_2009_	S1519	1519	4393	58	2009	W

2919_1722_S1520	2014	D	18.05.2014	S1520	13.3	19	Fam-59_2009_ S1520	1520	4394	59	2009	W
2920_1713_S1521	2014	D	18.05.2014	S1521	14	23	Fam-55_2009_ S1521	1521	4395	55	2009	W
2920_1713_S1522	2014	D	18.05.2014	S1522	14.7	24.5	Fam-59_2009_ S1522	1522	4396	59	2009	W
2920_1713_S1523	2014	D	18.05.2014	S1523	14.8	26	Fam-52_2009_ S1523	1523	4397	52	2009	W
2920_1713_S1524	2014	D	18.05.2014	S1524	13.2	20	Fam-90_2010_ S1524	1524	4398	90	2010	W
2920_1713_S1525	2014	D	18.05.2014	S1525	14.3	25	Fam-70_2010_ S1525	1525	4399	70	2010	H
2920_1713_S1526	2014	D	18.05.2014	S1526	13	17.5	Fam-59_2009_ S1526	1526	4400	59	2009	W
2920_1713_S1527	2014	D	18.05.2014	S1527	14	22	Fam-55_2009_ S1527	1527	4401	55	2009	W
2920_1713_S1528	2014	D	18.05.2014	S1528	12.4	16	Fam-42_2009_ S1528	1528	4402	42	2009	H
2920_1713_S1529	2014	D	18.05.2014	S1529	12.5	15.5	Fam-56_2009_ S1529	1529	4403	56	2009	W
2920_1713_S1530	2014	D	18.05.2014	S1530	13.2	19.5	Fam-59_2009_ S1530	1530	4404	59	2009	W
2920_1713_S1531	2014	D	18.05.2014	S1531	14.3	24	Fam-50_2009_ S1531	1531	4405	50	2009	H
2920_1713_S1532	2014	D	18.05.2014	S1532	13	18	Fam-59_2009_ S1532	1532	4406	59	2009	W
2920_1713_S1533	2014	D	18.05.2014	S1533	13.3	19.5	Fam-32_2009_ S1533	1533	4407	32	2009	H

2920_1713_S1534	2014	D	19.05.2014	S1534	14.5	24.5	Fam-52_2009_	S1534	1534	4408	52	2009	W
2920_1713_S1535	2014	D	19.05.2014	S1535	14.5	23.5	Fam-69_2010_	S1535	1535	4409	69	2010	F
2920_1713_S1536	2014	D	19.05.2014	S1536	14.1	24	Fam-56_2009_	S1536	1536	4410	56	2009	W
2920_1713_S1537	2014	D	19.05.2014	S1537	13.7	22.5	Fam-31_2009_	S1537	1537	4411	31	2009	F
2920_1713_S1538	2014	D	19.05.2014	S1538	14.3	24.5	Fam-55_2009_	S1538	1538	4412	55	2009	W
2920_1713_S1539	2014	D	19.05.2014	S1539	14.6	28.5	Fam-67_2010_	S1539	1539	4413	67	2010	F
2920_1713_S1540	2014	D	19.05.2014	S1540	14.1	23	Fam-42_2009_	S1540	1540	4414	42	2009	H
2920_1713_S1541	2014	D	19.05.2014	S1541	14.9	26.5	Fam-84_2010_	S1541	1541	4415	84	2010	W
2920_1713_S1542	2014	D	19.05.2014	S1542	14.6	26	Fam-38_2009_	S1542	1542	4416	38	2009	H
2920_1713_S1543	2014	D	19.05.2014	S1543	15.1	30	Fam-42_2009_	S1543	1543	4417	42	2009	H
2920_1713_S1544	2014	D	19.05.2014	S1544	14.2	24	Fam-53_2009_	S1544	1544	4418	53	2009	W
2920_1713_S1545	2014	D	19.05.2014	S1545	15.1	30.5	Fam-42_2009_	S1545	1545	4419	42	2009	H
2920_1713_S1546	2014	D	19.05.2014	S1546	14.1	25.5	Fam-60_2009_	S1546	1546	4420	60	2009	W
2920_1713_S1548	2014	D	19.05.2014	S1548	14.4	25.5	Fam-58_2009_	S1548	1548	4421	58	2009	W

2920_1713_S1549	2014	D	19.05.2014	S1549	13.7	21.5	Fam-55_2009_	S1549	1549	4422	55	2009	W
2920_1713_S1550	2014	D	19.05.2014	S1550	13.9	22.5	Fam-52_2009_	S1550	1550	4423	52	2009	W
2920_1713_S1551	2014	D	19.05.2014	S1551	14.5	26	Fam-32_2009_	S1551	1551	4424	32	2009	H
2920_1713_S1552	2014	D	19.05.2014	S1552	15.3	28	Fam-59_2009_	S1552	1552	4425	59	2009	W
2920_1713_S1553	2014	D	19.05.2014	S1553	14.6	26	Fam-52_2009_	S1553	1553	4426	52	2009	W
2920_1713_S1554	2014	D	19.05.2014	S1554	14	22	Fam-52_2009_	S1554	1554	4427	52	2009	W
2920_1713_S1555	2014	D	19.05.2014	S1555	13.2	19	Fam-55_2009_	S1555	1555	4428	55	2009	W
2920_1713_S1556	2014	D	19.05.2014	S1556	16.7	35	Fam-57_2009_	S1556	1556	4429	57	2009	W
2920_1713_S1557	2014	D	19.05.2014	S1557	15.9	33	Fam-34_2009_	S1557	1557	4430	34	2009	H
2920_1713_S1558	2014	D	19.05.2014	S1558	14.1	25	Fam-54_2009_	S1558	1558	4431	54	2009	W
2920_1713_S1559	2014	D	19.05.2014	S1559	13.3	19	Fam-58_2009_	S1559	1559	4432	58	2009	W
2920_1713_S1560	2014	D	19.05.2014	S1560	14.4	23.5	Fam-34_2009_	S1560	1560	4433	34	2009	H
2920_1713_S1561	2014	D	19.05.2014	S1561	15.7	31.5	Fam-51_2009_	S1561	1561	4434	51	2009	W
2920_1713_S1562	2014	D	19.05.2014	S1562	15	28	Fam-32_2009_	S1562	1562	4435	32	2009	H

2920_1713_S1563	2014	D	19.05.2014	S1563	13.3	20	Fam-59_2009_	S1563	1563	4436	59	2009	W
2920_1713_S1564	2014	D	19.05.2014	S1564	14	23	Fam-34_2009_	S1564	1564	4437	34	2009	H
2920_1713_S1565	2014	D	19.05.2014	S1565	14.5	26	Fam-60_2009_	S1565	1565	4438	60	2009	W
2920_1713_S1566	2014	D	19.05.2014	S1566	12.8	18.5	Fam-38_2009_	S1566	1566	4439	38	2009	H
2920_1713_S1567	2014	D	19.05.2014	S1567	13.1	19	Fam-89_2010_	S1567	1567	4440	89	2010	W
2920_1713_S1568	2014	D	19.05.2014	S1568	14.1	26	Fam-52_2009_	S1568	1568	4441	52	2009	W
2920_1713_S1569	2014	D	19.05.2014	S1569	12.5	17.5	Fam-87_2010_	S1569	1569	4442	87	2010	W
2920_1713_S1570	2014	D	19.05.2014	S1570	13.6	20.5	Fam-52_2009_	S1570	1570	4443	52	2009	W
2920_1713_S1571	2014	D	19.05.2014	S1571	13.9	22.5	Fam-57_2009_	S1571	1571	4444	57	2009	W
2920_1713_S1572	2014	D	19.05.2014	S1572	14.5	27	Fam-60_2009_	S1572	1572	4445	60	2009	W
2920_1713_S1573	2014	D	19.05.2014	S1573	14	23.5	Fam-54_2009_	S1573	1573	4446	54	2009	W
2920_1713_S1574	2014	D	19.05.2014	S1574	14.9	27.5	Fam-58_2009_	S1574	1574	4447	58	2009	W
2920_1713_S1575	2014	D	19.05.2014	S1575	13.1	18.5	Fam-55_2009_	S1575	1575	4448	55	2009	W
2920_1713_S1576	2014	D	19.05.2014	S1576	15.7	30	Fam-32_2009_	S1576	1576	4449	32	2009	H

2920_1713_S1577	2014	D	19.05.2014	S1577	14.5	25.5	Fam-47_2009_S1577	1577	4450	47	2009	F
2920_1713_S1578	2014	D	19.05.2014	S1578	13.2	19	Fam-51_2009_S1578	1578	4451	51	2009	W
2920_1713_S1579	2014	D	19.05.2014	S1579	12.5	20.5	Fam-85_2010_S1579	1579	4452	85	2010	W
2920_1713_S1580	2014	D	19.05.2014	S1580	13.2	19	Fam-54_2009_S1580	1580	4453	54	2009	W
2920_1713_S1581	2014	D	19.05.2014	S1581	13.4	20	Fam-67_2010_S1581	1581	4454	67	2010	F
2920_1713_S1582	2014	D	19.05.2014	S1582	14.5	26	Fam-58_2009_S1582	1582	4455	58	2009	W
2920_1713_S1583	2014	D	19.05.2014	S1583	12.1	15	Fam-85_2010_S1583	1583	4456	85	2010	W
2920_1713_S1584	2014	D	19.05.2014	S1584	13.8	22.5	Fam-56_2009_S1584	1584	4457	56	2009	W
2920_1713_S1585	2014	D	19.05.2014	S1585	14.1	23	Fam-54_2009_S1585	1585	4458	54	2009	W
2920_1713_S1586	2014	D	19.05.2014	S1586	13.7	20	Fam-57_2009_S1586	1586	4459	57	2009	W
2920_1713_S1587	2014	D	19.05.2014	S1587	13.7	21	Fam-84_2010_S1587	1587	4460	84	2010	W
2920_1713_S1588	2014	D	19.05.2014	S1588	14.1	23	Fam-57_2009_S1588	1588	4461	57	2009	W
2920_1713_S1589	2014	D	19.05.2014	S1589	12.2	15	Fam-60_2009_S1589	1589	4462	60	2009	W
2920_1713_S1590	2014	D	19.05.2014	S1590	15.3	29.5	Fam-56_2009_S1590	1590	4463	56	2009	W

2920_1713_S1591	2014	D	19.05.2014	S1591	12.6	16.5	Fam-58_2009_	S1591	1591	4464	58	2009	W
2920_1713_S1592	2014	D	19.05.2014	S1592	13.8	24	Fam-56_2009_	S1592	1592	4465	56	2009	W
2920_1713_S1593	2014	D	19.05.2014	S1593	14.7	25	Fam-83_2010_	S1593	1593	4466	83	2010	W
2920_1713_S1594	2014	D	19.05.2014	S1594	13.3	22	Fam-42_2009_	S1594	1594	4467	42	2009	H
2920_1713_S1595	2014	D	19.05.2014	S1595	14.9	26.5	Fam-34_2009_	S1595	1595	4468	34	2009	H
2920_1713_S1596	2014	D	19.05.2014	S1596	15	28.5	Fam-54_2009_	S1596	1596	4469	54	2009	W
2920_1713_S1597	2014	D	19.05.2014	S1597	13.9	22.5	Fam-54_2009_	S1597	1597	4470	54	2009	W
2920_1713_S1598	2014	D	19.05.2014	S1598	14.6	26	Fam-52_2009_	S1598	1598	4471	52	2009	W
2920_1713_S1599	2014	D	19.05.2014	S1599	12.5	16.5	Fam-56_2009_	S1599	1599	4472	56	2009	W
2920_1713_S1600	2014	D	19.05.2014	S1600	13.7	22.5	Fam-58_2009_	S1600	1600	4473	58	2009	W
2921_1713_S1602	2014	D	19.05.2014	S1602	14.4	25	Fam-55_2009_	S1602	1602	4474	55	2009	W
2921_1713_S1603	2014	D	19.05.2014	S1603	14.2	22.5	Fam-57_2009_	S1603	1603	4475	57	2009	W
2921_1713_S1604	2014	D	19.05.2014	S1604	13.8	22	Fam-57_2009_	S1604	1604	4476	57	2009	W
2921_1713_S1605	2014	D	19.05.2014	S1605	14.2	24	Fam-60_2009_	S1605	1605	4477	60	2009	W

2921_1713_S1606	2014	D	19.05.2014	S1606	14.3	28.5	Fam-54_2009_	S1606	1606	4478	54	2009	W
2921_1713_S1607	2014	D	19.05.2014	S1607	14.6	27.5	Fam-42_2009_	S1607	1607	4479	42	2009	H
2921_1713_S1608	2014	D	19.05.2014	S1608	14	23	Fam-52_2009_	S1608	1608	4480	52	2009	W
2921_1713_S1609	2014	D	19.05.2014	S1609	14.6	28	Fam-58_2009_	S1609	1609	4481	58	2009	W
2921_1713_S1610	2014	D	19.05.2014	S1610	13.9	23	Fam-58_2009_	S1610	1610	4482	58	2009	W
2921_1713_S1611	2014	D	19.05.2014	S1611	14.6	24.5	Fam-54_2009_	S1611	1611	4483	54	2009	W
2921_1713_S1612	2014	D	19.05.2014	S1612	14.2	24	Fam-04_2007_	S1612	1612	4484	4	2007	W
2921_1713_S1613	2014	D	19.05.2014	S1613	13.7	21	Fam-52_2009_	S1613	1613	4485	52	2009	W
2921_1713_S1614	2014	D	19.05.2014	S1614	13.3	20.5	Fam-74_2010_	S1614	1614	4486	74	2010	H
2921_1714_S1615	2014	D	19.05.2014	S1615	15.2	27.5	Fam-57_2009_	S1615	1615	4487	57	2009	W
2921_1714_S1616	2014	D	19.05.2014	S1616	13.8	21.5	Fam-54_2009_	S1616	1616	4488	54	2009	W
2921_1714_S1618	2014	D	19.05.2014	S1618	13.5	21	Fam-56_2009_	S1618	1618	4489	56	2009	W
2921_1714_S1619	2014	D	19.05.2014	S1619	14.7	24.5	Fam-51_2009_	S1619	1619	4490	51	2009	W
2921_1714_S1620	2014	D	19.05.2014	S1620	15	29	Fam-60_2009_	S1620	1620	4491	60	2009	W

2921_1714_S1621	2014	D	19.05.2014	S1621	14.6	26.5	Fam-60_2009_	S1621	1621	4492	60	2009	W
2921_1714_S1622	2014	D	19.05.2014	S1622	15.4	27.5	Fam-51_2009_	S1622	1622	4493	51	2009	W
2921_1714_S1623	2014	D	19.05.2014	S1623	14.2	23.5	Fam-52_2009_	S1623	1623	4494	52	2009	W
2921_1714_S1624	2014	D	19.05.2014	S1624	15.5	30	Fam-57_2009_	S1624	1624	4495	57	2009	W
2921_1714_S1625	2014	D	19.05.2014	S1625	15.2	30	Fam-57_2009_	S1625	1625	4496	57	2009	W
2921_1714_S1626	2014	D	19.05.2014	S1626	13.5	22	Fam-59_2009_	S1626	1626	4497	59	2009	W
2921_1714_S1627	2014	D	19.05.2014	S1627	14	21.5	Fam-36_2009_	S1627	1627	4498	36	2009	H
2921_1714_S1628	2014	D	19.05.2014	S1628	15.3	31	Fam-32_2009_	S1628	1628	4499	32	2009	H
2921_1714_S1629	2014	D	19.05.2014	S1629	13.9	22	Fam-52_2009_	S1629	1629	4500	52	2009	W
2921_1714_S1630	2014	D	19.05.2014	S1630	14	22	Fam-59_2009_	S1630	1630	4501	59	2009	W
2921_1714_S1631	2014	D	19.05.2014	S1631	15.7	32	Fam-45_2009_	S1631	1631	4502	45	2009	F
2921_1714_S1632	2014	D	19.05.2014	S1632	14.4	25	Fam-42_2009_	S1632	1632	4503	42	2009	H
2921_1714_S1633	2014	D	19.05.2014	S1633	14.8	25.5	Fam-52_2009_	S1633	1633	4504	52	2009	W
2921_1714_S1634	2014	D	19.05.2014	S1634	15.7	30.5	Fam-32_2009_	S1634	1634	4505	32	2009	H

2921_1714_S1635	2014	D	19.05.2014	S1635	14.1	23	Fam-62_1_201	S1635	1635	4506	62	2010	H
2921_1714_S1636	2014	D	19.05.2014	S1636	14.5	26	Fam-87_2010_	S1636	1636	4507	87	2010	W
2921_1714_S1637	2014	D	19.05.2014	S1637	15	29	Fam-58_2009_	S1637	1637	4508	58	2009	W
2921_1714_S1638	2014	D	19.05.2014	S1638	15.6	30	Fam-40_2009_	S1638	1638	4509	40	2009	H
2921_1714_S1639	2014	D	19.05.2014	S1639	16	33	Fam-57_2009_	S1639	1639	4510	57	2009	W
2921_1714_S1640	2014	D	19.05.2014	S1640	13.2	19	Fam-86_2010_	S1640	1640	4511	86	2010	W
2921_1714_S1641	2014	D	19.05.2014	S1641	13.6	20	Fam-68_2010_	S1641	1641	4512	68	2010	H
2921_1714_S1642	2014	D	19.05.2014	S1642	14.1	23.5	Fam-59_2009_	S1642	1642	4513	59	2009	W
2921_1714_S1643	2014	T	19.05.2014	S1643	14.8	24	Family-76_201	S1643	1643	4514	76	2010	H
2921_1714_S1644	2014	D	19.05.2014	S1644	15.2	28	Fam-42_2009_	S1644	1644	4515	42	2009	H
2921_1714_S1645	2014	D	19.05.2014	S1645	13	18.5	Fam-10_2007_	S1645	1645	4516	10	2007	W
2921_1714_S1646	2014	D	19.05.2014	S1646	14	22	Fam-40_2009_	S1646	1646	4517	40	2009	H
2921_1714_S1647	2014	D	19.05.2014	S1647	15.5	32.5	Fam-44_2009_	S1647	1647	4518	44	2009	H
2921_1714_S1648	2014	D	19.05.2014	S1648	14	23	Fam-38_2009_	S1648	1648	4519	38	2009	H

2921_1714_S1649	2014	D	19.05.2014	S1649	14.8	25.5	Fam-58_2009_	S1649	1649	4520	58	2009	W
2921_1714_S1650	2014	D	19.05.2014	S1650	14.4	24	Fam-50_2009_	S1650	1650	4521	50	2009	H
2921_1714_S1651	2014	D	19.05.2014	S1651	14.5	25	Fam-51_2009_	S1651	1651	4522	51	2009	W
2921_1714_S1652	2014	D	19.05.2014	S1652	14.9	26	Fam-74_2010_	S1652	1652	4523	74	2010	H
2921_1714_S1653	2014	D	19.05.2014	S1653	14.8	25.5	Fam-40_2009_	S1653	1653	4524	40	2009	H
2921_1714_S1654	2014	D	19.05.2014	S1654	13.2	18	Fam-59_2009_	S1654	1654	4525	59	2009	W
2921_1714_S1655	2014	D	19.05.2014	S1655	14.3	22	Fam-57_2009_	S1655	1655	4526	57	2009	W
2921_1714_S1656	2014	D	19.05.2014	S1656	15.5	30.5	Fam-43_2009_	S1656	1656	4527	43	2009	F
2921_1714_S1657	2014	D	19.05.2014	S1657	14.6	24.5	Fam-57_2009_	S1657	1657	4528	57	2009	W
2921_1714_S1658	2014	D	19.05.2014	S1658	13.5	19	Fam-59_2009_	S1658	1658	4529	59	2009	W
2921_1714_S1659	2014	D	19.05.2014	S1659	12.8	17	Fam-54_2009_	S1659	1659	4530	54	2009	W
2921_1714_S1660	2014	D	19.05.2014	S1660	15.2	29	Fam-54_2009_	S1660	1660	4531	54	2009	W
2921_1714_S1662	2014	D	19.05.2014	S1662	13.5	21	Fam-58_2009_	S1662	1662	4532	58	2009	W
2921_1714_S1664	2014	D	19.05.2014	S1664	14.5	24.5	Fam-55_2009_	S1664	1664	4533	55	2009	W

2921_1714_S1665	2014	D	19.05.2014	S1665	11.3	15.5	Fam-59_2009_	S1665	1665	4534	59	2009	W
2921_1714_S1666	2014	D	19.05.2014	S1666	13.5	20	Fam-58_2009_	S1666	1666	4535	58	2009	W
2921_1714_S1667	2014	D	19.05.2014	S1667	12.4	16	Fam-59_2009_	S1667	1667	4536	59	2009	W
2921_1714_S1668	2014	D	19.05.2014	S1668	13.3	20.5	Fam-42_2009_	S1668	1668	4537	42	2009	H
2921_1714_S1669	2014	D	19.05.2014	S1669	14.7	20	Fam-52_2009_	S1669	1669	4538	52	2009	W
2921_1714_S1670	2014	D	19.05.2014	S1670	14.9	27.5	Fam-57_2009_	S1670	1670	4539	57	2009	W
2921_1714_S1671	2014	D	19.05.2014	S1671	14.2	25.5	Fam-45_2009_	S1671	1671	4540	45	2009	F
2921_1714_S1672	2014	D	19.05.2014	S1672	13.7	22	Fam-57_2009_	S1672	1672	4541	57	2009	W
2921_1714_S1673	2014	D	19.05.2014	S1673	13.5	20.5	Fam-52_2009_	S1673	1673	4542	52	2009	W
2921_1714_S1674	2014	D	19.05.2014	S1674	14.5	24	Fam-54_2009_	S1674	1674	4543	54	2009	W
2921_1714_S1675	2014	D	19.05.2014	S1675	14.8	28	Fam-40_2009_	S1675	1675	4544	40	2009	H
2921_1714_S1676	2014	D	19.05.2014	S1676	12.8	19	Fam-57_2009_	S1676	1676	4545	57	2009	W
2921_1714_S1678	2014	D	19.05.2014	S1678	13.7	21.5	Fam-59_2009_	S1678	1678	4546	59	2009	W
2921_1714_S1679	2014	D	19.05.2014	S1679	15.1	29	Fam-83_2010_	S1679	1679	4547	83	2010	W

2921_1714_S1680	2014	D	19.05.2014	S1680	13.1	21.5	Fam-55_2009_	S1680	1680	4548	55	2009	W
2924_1714_S1681	2014	D	19.05.2014	S1681	13.9	22	Fam-54_2009_	S1681	1681	4549	54	2009	W
2924_1714_S1682	2014	D	19.05.2014	S1682	14.1	22.5	Fam-53_2009_	S1682	1682	4550	53	2009	W
2924_1714_S1683	2014	D	19.05.2014	S1683	13.4	20.5	Fam-57_2009_	S1683	1683	4551	57	2009	W
2924_1714_S1684	2014	D	19.05.2014	S1684	14.5	24.5	Fam-57_2009_	S1684	1684	4552	57	2009	W
2924_1714_S1685	2014	D	19.05.2014	S1685	15.3	31.5	Fam-58_2009_	S1685	1685	4553	58	2009	W
2924_1714_S1687	2014	D	19.05.2014	S1687	14.1	22	Fam-55_2009_	S1687	1687	4554	55	2009	W
2924_1714_S1688	2014	D	19.05.2014	S1688	13.5	20.5	Fam-54_2009_	S1688	1688	4555	54	2009	W
2924_1714_S1689	2014	D	19.05.2014	S1689	15.7	30	Fam-39_2009_	S1689	1689	4556	39	2009	F
2924_1714_S1690	2014	D	19.05.2014	S1690	13.9	23.5	Fam-58_2009_	S1690	1690	4557	58	2009	W
2924_1714_S1691	2014	D	19.05.2014	S1691	14.4	20.4	Fam-55_2009_	S1691	1691	4558	55	2009	W
2924_1714_S1692	2014	D	19.05.2014	S1692	14.5	26	Fam-85_2010_	S1692	1692	4559	85	2010	W
2924_1714_S1693	2014	D	19.05.2014	S1693	14.5	26	Fam-60_2009_	S1693	1693	4560	60	2009	W
2924_1714_S1694	2014	D	19.05.2014	S1694	16.7	37	Fam-34_2009_	S1694	1694	4561	34	2009	H

2924_1714_S1695	2014	D	19.05.2014	S1695	14.3	27.5	Fam-42_2009_	S1695	1695	4562	42	2009	H
2924_1714_S1696	2014	D	19.05.2014	S1696	13.3	21	Fam-51_2009_	S1696	1696	4563	51	2009	W
2924_1714_S1697	2014	D	19.05.2014	S1697	15.9	30.5	Fam-51_2009_	S1697	1697	4564	51	2009	W
2924_1714_S1698	2014	D	19.05.2014	S1698	15.7	31	Fam-60_2009_	S1698	1698	4565	60	2009	W
2924_1714_S1699	2014	D	19.05.2014	S1699	15	28	Fam-54_2009_	S1699	1699	4566	54	2009	W
2924_1714_S1700	2014	D	19.05.2014	S1700	14	22.5	Fam-76_2010_	S1700	1700	4567	76	2010	H
2924_1714_S1701	2014	D	19.05.2014	S1701	13.1	20	Fam-80_2010_	S1701	1701	4568	80	2010	H
2924_1714_S1703	2014	D	19.05.2014	S1703	12.5	18	Fam-42_2009_	S1703	1703	4569	42	2009	H
2924_1714_S1704	2014	D	19.05.2014	S1704	14.2	23.5	Fam-54_2009_	S1704	1704	4570	54	2009	W
2924_1714_S1705	2014	D	19.05.2014	S1705	14.1	23.5	Fam-40_2009_	S1705	1705	4571	40	2009	H
2924_1714_S1707	2014	D	19.05.2014	S1707	15.3	30.5	Fam-50_2009_	S1707	1707	4572	50	2009	H
2924_1714_S1708	2014	D	19.05.2014	S1708	15	27	Fam-89_2010_	S1708	1708	4573	89	2010	W
2924_1715_S1709	2014	D	19.05.2014	S1709	13.6	21.5	Fam-58_2009_	S1709	1709	4574	58	2009	W
2924_1715_S1710	2014	D	19.05.2014	S1710	13.8	23.5	Fam-58_2009_	S1710	1710	4575	58	2009	W

2924_1715_S1711	2014	D	19.05.2014	S1711	12.4	16	Fam-42_2009_	S1711	1711	4576	42	2009	H
2924_1715_S1712	2014	D	19.05.2014	S1712	14.5	26	Fam-54_2009_	S1712	1712	4577	54	2009	W
2924_1715_S1713	2014	D	19.05.2014	S1713	15	26	Fam-57_2009_	S1713	1713	4578	57	2009	W
2924_1715_S1714	2014	D	19.05.2014	S1714	14.1	23	Fam-55_2009_	S1714	1714	4579	55	2009	W
2924_1715_S1716	2014	D	19.05.2014	S1716	13.8	23	Fam-54_2009_	S1716	1716	4580	54	2009	W
2924_1715_S1717	2014	D	19.05.2014	S1717	13.2	20	Fam-90_2010_	S1717	1717	4581	90	2010	W
2924_1715_S1718	2014	D	19.05.2014	S1718	12.9	17	Fam-54_2009_	S1718	1718	4582	54	2009	W
2924_1715_S1719	2014	D	19.05.2014	S1719	14.3	25	Fam-59_2009_	S1719	1719	4583	59	2009	W
2924_1715_S1720	2014	D	19.05.2014	S1720	14.7	26	Fam-58_2009_	S1720	1720	4584	58	2009	W
2924_1715_S1721	2014	D	19.05.2014	S1721	13	19	Fam-58_2009_	S1721	1721	4585	58	2009	W
2924_1715_S1722	2014	D	19.05.2014	S1722	13.5	21	Fam-60_2009_	S1722	1722	4586	60	2009	W
2924_1715_S1723	2014	D	19.05.2014	S1723	15.2	27	Fam-32_2009_	S1723	1723	4587	32	2009	H
2924_1715_S1724	2014	D	19.05.2014	S1724	14.2	23.5	Fam-54_2009_	S1724	1724	4588	54	2009	W
2924_1715_S1725	2014	D	19.05.2014	S1725	13.7	21	Fam-34_2009_	S1725	1725	4589	34	2009	H

2924_1715_S1726	2014	D	19.05.2014	S1726	14.5	22.5	Fam-89_2010_	S1726	1726	4590	89	2010	W
2924_1715_S1727	2014	D	19.05.2014	S1727	13.8	21.5	Fam-64_2010_	S1727	1727	4591	64	2010	H
2924_1715_S1728	2014	D	19.05.2014	S1728	12	14.5	Fam-32_2009_	S1728	1728	4592	32	2009	H
2924_1715_S1729	2014	D	19.05.2014	S1729	15	26	Fam-57_2009_	S1729	1729	4593	57	2009	W
2924_1715_S1730	2014	D	19.05.2014	S1730	13.5	21	Fam-52_2009_	S1730	1730	4594	52	2009	W
2924_1715_S1731	2014	D	19.05.2014	S1731	15.2	27	Fam-32_2009_	S1731	1731	4595	32	2009	H
2924_1715_S1732	2014	D	19.05.2014	S1732	14.2	22.5	Fam-89_2010_	S1732	1732	4596	89	2010	W
2924_1715_S1733	2014	D	19.05.2014	S1733	13.8	22	Fam-57_2009_	S1733	1733	4597	57	2009	W
2924_1715_S1734	2014	D	19.05.2014	S1734	16.3	36	Fam-34_2009_	S1734	1734	4598	34	2009	H
2924_1715_S1735	2014	D	19.05.2014	S1735	13.7	20	Fam-57_2009_	S1735	1735	4599	57	2009	W
2924_1715_S1736	2014	D	19.05.2014	S1736	13.8	20.5	Fam-52_2009_	S1736	1736	4600	52	2009	W
2924_1715_S1737	2014	D	19.05.2014	S1737	14.6	25.5	Fam-44_2009_	S1737	1737	4601	44	2009	H
2924_1715_S1738	2014	D	19.05.2014	S1738	15.3	27	Fam-70_2010_	S1738	1738	4602	70	2010	H
2924_1715_S1740	2014	D	19.05.2014	S1740	13	18.5	Fam-58_2009_	S1740	1740	4603	58	2009	W

2924_1715_S1741	2014	D	19.05.2014	S1741	13.8	21	Fam-76_2010_S1741	1741	4604	76	2010	H
2924_1715_S1742	2014	D	19.05.2014	S1742	13.9	22	Fam-42_2009_S1742	1742	4605	42	2009	H
2924_1715_S1743	2014	D	19.05.2014	S1743	15	28	Fam-60_2009_S1743	1743	4606	60	2009	W
2924_1715_S1744	2014	D	19.05.2014	S1744	14.4	23	Fam-59_2009_S1744	1744	4607	59	2009	W
2924_1715_S1745	2014	D	19.05.2014	S1745	14.5	24.5	Fam-54_2009_S1745	1745	4608	54	2009	W
2924_1715_S1746	2014	D	19.05.2014	S1746	14.5	24	Fam-58_2009_S1746	1746	4609	58	2009	W
2924_1715_S1747	2014	D	19.05.2014	S1747	15.3	28	Fam-53_2009_S1747	1747	4610	53	2009	W
2924_1715_S1748	2014	D	19.05.2014	S1748	15.6	28	Fam-34_2009_S1748	1748	4611	34	2009	H
2924_1715_S1749	2014	D	19.05.2014	S1749	14.5	25.5	Fam-59_2009_S1749	1749	4612	59	2009	W
2924_1715_S1750	2014	D	19.05.2014	S1750	14	23	Fam-59_2009_S1750	1750	4613	59	2009	W
2924_1715_S1751	2014	D	19.05.2014	S1751	15.8	32	Fam-32_2009_S1751	1751	4614	32	2009	H
2924_1715_S1752	2014	D	19.05.2014	S1752	13.3	19	Fam-58_2009_S1752	1752	4615	58	2009	W
2924_1715_S1753	2014	D	19.05.2014	S1753	14	23.5	Fam-59_2009_S1753	1753	4616	59	2009	W
2924_1715_S1754	2014	D	19.05.2014	S1754	13.3	18	Fam-57_2009_S1754	1754	4617	57	2009	W

2924_1715_S1755	2014	D	19.05.2014	S1755	14.4	23	Fam-54_2009_	S1755	1755	4618	54	2009	W
2924_1715_S1756	2014	D	19.05.2014	S1756	14.8	27.5	Fam-45_2009_	S1756	1756	4619	45	2009	F
2924_1715_S1757	2014	D	19.05.2014	S1757	14.5	23	Fam-51_2009_	S1757	1757	4620	51	2009	W
2924_1715_S1758	2014	D	19.05.2014	S1758	13.3	17.5	Fam-53_2009_	S1758	1758	4621	53	2009	W
2924_1715_S1759	2014	D	19.05.2014	S1759	13.6	20.5	Fam-58_2009_	S1759	1759	4622	58	2009	W
2924_1715_S1760	2014	D	19.05.2014	S1760	14	23	Fam-42_2009_	S1760	1760	4623	42	2009	H
2925_1715_S1761	2014	D	19.05.2014	S1761	13.5	22	Fam-58_2009_	S1761	1761	4624	58	2009	W
2925_1715_S1762	2014	D	19.05.2014	S1762	14	24	Fam-59_2009_	S1762	1762	4625	59	2009	W
2925_1715_S1763	2014	D	19.05.2014	S1763	12.9	19.5	Fam-70_2010_	S1763	1763	4626	70	2010	H
2925_1715_S1764	2014	D	19.05.2014	S1764	15.4	30	Fam-51_2009_	S1764	1764	4627	51	2009	W
2925_1715_S1766	2014	D	19.05.2014	S1766	14.4	25.5	Fam-51_2009_	S1766	1766	4628	51	2009	W
2925_1715_S1767	2014	D	19.05.2014	S1767	15.5	29.5	Fam-57_2009_	S1767	1767	4629	57	2009	W
2925_1715_S1768	2014	D	19.05.2014	S1768	12.5	17	Fam-38_2009_	S1768	1768	4630	38	2009	H
2925_1715_S1770	2014	D	19.05.2014	S1770	13.3	20	Fam-54_2009_	S1770	1770	4631	54	2009	W

2925_1715_S1771	2014	D	19.05.2014	S1771	14.7	29	Fam-90_2010_	S1771	1771	4632	90	2010	W
2925_1715_S1772	2014	D	19.05.2014	S1772	15.2	27.5	Fam-54_2009_	S1772	1772	4633	54	2009	W
2925_1715_S1773	2014	D	19.05.2014	S1773	12.5	16.5	Fam-85_2010_	S1773	1773	4634	85	2010	W
2925_1715_S1774	2014	D	19.05.2014	S1774	13.6	22	Fam-58_2009_	S1774	1774	4635	58	2009	W
2925_1715_S1775	2014	D	19.05.2014	S1775	13.9	21	Fam-52_2009_	S1775	1775	4636	52	2009	W
2925_1715_S1777	2014	D	19.05.2014	S1777	13.7	21	Fam-57_2009_	S1777	1777	4637	57	2009	W
2925_1715_S1778	2014	D	19.05.2014	S1778	13.5	21	Fam-60_2009_	S1778	1778	4638	60	2009	W
2925_1715_S1779	2014	D	19.05.2014	S1779	15.6	31.5	Fam-54_2009_	S1779	1779	4639	54	2009	W
2925_1715_S1780	2014	D	19.05.2014	S1780	14.2	26.5	Fam-62_1_201	S1780	1780	4640	62	2010	H
2925_1715_S1781	2014	D	19.05.2014	S1781	15.2	28	Fam-40_2009_	S1781	1781	4641	40	2009	H
2925_1715_S1782	2014	D	19.05.2014	S1782	15.7	31	Fam-40_2009_	S1782	1782	4642	40	2009	H
2925_1715_S1783	2014	D	19.05.2014	S1783	14.7	26.5	Fam-55_2009_	S1783	1783	4643	55	2009	W
2925_1715_S1784	2014	D	19.05.2014	S1784	13.8	24.5	Fam-54_2009_	S1784	1784	4644	54	2009	W
2925_1715_S1785	2014	D	19.05.2014	S1785	13.2	20.5	Fam-54_2009_	S1785	1785	4645	54	2009	W

2925_1715_S1786	2014	D	19.05.2014	S1786	14.6	26	Fam-85_2010_	S1786	1786	4646	85	2010	W
2925_1715_S1787	2014	D	19.05.2014	S1787	14.6	26	Fam-54_2009_	S1787	1787	4647	54	2009	W
2925_1715_S1788	2014	D	19.05.2014	S1788	14.7	26.5	Fam-55_2009_	S1788	1788	4648	55	2009	W
2925_1715_S1789	2014	D	19.05.2014	S1789	14.4	24.5	Fam-83_2010_	S1789	1789	4649	83	2010	W
2925_1715_S1790	2014	D	19.05.2014	S1790	16.6	37	Fam-40_2009_	S1790	1790	4650	40	2009	H
2925_1715_S1791	2014	D	19.05.2014	S1791	14.9	27.5	Fam-54_2009_	S1791	1791	4651	54	2009	W
2925_1715_S1792	2014	D	19.05.2014	S1792	14.4	24	Fam-54_2009_	S1792	1792	4652	54	2009	W
2925_1715_S1793	2014	D	19.05.2014	S1793	14.5	26	Fam-63_2010_	S1793	1793	4653	63	2010	F
2925_1715_S1794	2014	D	19.05.2014	S1794	14.5	26	Fam-87_2010_	S1794	1794	4654	87	2010	W
2925_1715_S1795	2014	D	19.05.2014	S1795	14	21	Fam-57_2009_	S1795	1795	4655	57	2009	W
2925_1715_S1796	2014	D	19.05.2014	S1796	14.6	25	Fam-52_2009_	S1796	1796	4656	52	2009	W
2925_1715_S1798	2014	D	19.05.2014	S1798	13.5	19	Fam-54_2009_	S1798	1798	4657	54	2009	W
2925_1715_S1799	2014	D	19.05.2014	S1799	15	28.5	Fam-54_2009_	S1799	1799	4658	54	2009	W
2925_1715_S1800	2014	D	19.05.2014	S1800	13.2	16	Fam-40_2009_	S1800	1800	4659	40	2009	H

2925_1715_S1801	2014	D	19.05.2014	S1801	13.9	22.5	Fam-54_2009_	S1801	1801	4660	54	2009	W
2925_1715_S1802	2014	D	19.05.2014	S1802	13.4	20	Fam-59_2009_	S1802	1802	4661	59	2009	W
2925_1716_S1803	2014	D	19.05.2014	S1803	13.9	23	Fam-54_2009_	S1803	1803	4662	54	2009	W
2925_1716_S1804	2014	D	19.05.2014	S1804	12.7	17	Fam-54_2009_	S1804	1804	4663	54	2009	W
2925_1716_S1805	2014	D	19.05.2014	S1805	13.8	18.5	Fam-32_2009_	S1805	1805	4664	32	2009	H
2925_1716_S1806	2014	D	19.05.2014	S1806	13.7	20.5	Fam-66_2010_	S1806	1806	4665	66	2010	H
2925_1716_S1807	2014	D	19.05.2014	S1807	14.2	25	Fam-59_2009_	S1807	1807	4666	59	2009	W
2925_1716_S1808	2014	D	19.05.2014	S1808	13.1	19.5	Fam-44_2009_	S1808	1808	4667	44	2009	H
2925_1716_S1809	2014	D	19.05.2014	S1809	14.9	25.5	Fam-54_2009_	S1809	1809	4668	54	2009	W
2925_1716_S1810	2014	D	19.05.2014	S1810	14.1	24	Fam-52_2009_	S1810	1810	4669	52	2009	W
2925_1716_S1811	2014	D	19.05.2014	S1811	14.2	23.5	Fam-54_2009_	S1811	1811	4670	54	2009	W
2925_1716_S1812	2014	D	19.05.2014	S1812	13.8	20.5	Fam-54_2009_	S1812	1812	4671	54	2009	W
2925_1716_S1813	2014	D	19.05.2014	S1813	13.8	22	Fam-56_2009_	S1813	1813	4672	56	2009	W
2925_1716_S1814	2014	D	19.05.2014	S1814	14.5	24.5	Fam-52_2009_	S1814	1814	4673	52	2009	W

2925_1716_S1815	2014	D	19.05.2014	S1815	12	16	Fam-81_2010_ S1815	1815	4674	81	2010	W
2925_1716_S1816	2014	D	19.05.2014	S1816	13.6	21	Fam-60_2009_ S1816	1816	4675	60	2009	W
2925_1716_S1817	2014	D	19.05.2014	S1817	14.4	24	Fam-84_2010_ S1817	1817	4676	84	2010	W
2925_1716_S1818	2014	D	19.05.2014	S1818	13.6	22	Fam-60_2009_ S1818	1818	4677	60	2009	W
2925_1716_S1819	2014	D	19.05.2014	S1819	12.9	17.5	Fam-54_2009_ S1819	1819	4678	54	2009	W
2925_1716_S1820	2014	D	19.05.2014	S1820	13.8	21	Fam-53_2009_ S1820	1820	4679	53	2009	W
2925_1716_S1821	2014	D	19.05.2014	S1821	14.6	25	Fam-51_2009_ S1821	1821	4680	51	2009	W
2925_1716_S1822	2014	D	19.05.2014	S1822	13.6	21	Fam-54_2009_ S1822	1822	4681	54	2009	W
2925_1716_S1823	2014	D	19.05.2014	S1823	15.4	29.5	Fam-41_2009_ S1823	1823	4682	41	2009	F
2925_1716_S1824	2014	D	19.05.2014	S1824	14	20.5	Fam-57_2009_ S1824	1824	4683	57	2009	W
2925_1716_S1825	2014	D	19.05.2014	S1825	16.6	37.5	Fam-52_2009_ S1825	1825	4684	52	2009	W
2925_1716_S1826	2014	D	19.05.2014	S1826	14.9	26	Fam-54_2009_ S1826	1826	4685	54	2009	W
2925_1716_S1827	2014	D	19.05.2014	S1827	14.3	24.5	Fam-51_2009_ S1827	1827	4686	51	2009	W
2925_1716_S1828	2014	D	19.05.2014	S1828	14.6	26.5	Fam-51_2009_ S1828	1828	4687	51	2009	W

2925_1716_S1829	2014	D	19.05.2014	S1829	13.9	23.5	Fam-54_2009_	S1829	1829	4688	54	2009	W
2925_1716_S1830	2014	D	19.05.2014	S1830	13.5	21	Fam-54_2009_	S1830	1830	4689	54	2009	W
2925_1716_S1831	2014	D	19.05.2014	S1831	15.2	27	Fam-32_2009_	S1831	1831	4690	32	2009	H
2925_1716_S1832	2014	D	19.05.2014	S1832	15	28	Fam-51_2009_	S1832	1832	4691	51	2009	W
2925_1716_S1834	2014	D	19.05.2014	S1834	13.1	18	Fam-51_2009_	S1834	1834	4692	51	2009	W
2925_1716_S1835	2014	D	19.05.2014	S1835	13.8	22	Fam-52_2009_	S1835	1835	4693	52	2009	W
2925_1716_S1836	2014	D	19.05.2014	S1836	13.3	20	Fam-58_2009_	S1836	1836	4694	58	2009	W
2925_1716_S1837	2014	D	19.05.2014	S1837	13.8	22.5	Fam-90_2010_	S1837	1837	4695	90	2010	W
2925_1716_S1838	2014	D	19.05.2014	S1838	13.5	22	Fam-58_2009_	S1838	1838	4696	58	2009	W
2925_1716_S1839	2014	D	19.05.2014	S1839	15.5	31	Fam-53_2009_	S1839	1839	4697	53	2009	W
2925_1716_S1840	2014	D	19.05.2014	S1840	13.7	20.5	Fam-55_2009_	S1840	1840	4698	55	2009	W
2926_1716_S1841	2014	D	19.05.2014	S1841	13.6	16.5	Fam-59_2009_	S1841	1841	4699	59	2009	W
2926_1716_S1842	2014	D	19.05.2014	S1842	13.3	21.5	Fam-52_2009_	S1842	1842	4700	52	2009	W
2926_1716_S1843	2014	D	19.05.2014	S1843	13.7	22	Fam-55_2009_	S1843	1843	4701	55	2009	W

2926_1716_S1844	2014	D	19.05.2014	S1844	12.6	15.5	Fam-59_2009_	S1844	1844	4702	59	2009	W
2926_1716_S1845	2014	D	19.05.2014	S1845	13.8	22.5	Fam-58_2009_	S1845	1845	4703	58	2009	W
2926_1716_S1846	2014	D	19.05.2014	S1846	13.5	20.5	Fam-58_2009_	S1846	1846	4704	58	2009	W
2926_1716_S1847	2014	D	19.05.2014	S1847	12.1	15	Fam-59_2009_	S1847	1847	4705	59	2009	W
2926_1716_S1848	2014	D	19.05.2014	S1848	14.4	25	Fam-40_2009_	S1848	1848	4706	40	2009	H
2926_1716_S1849	2014	D	19.05.2014	S1849	13.9	22	Fam-56_2009_	S1849	1849	4707	56	2009	W
2926_1716_S1850	2014	D	19.05.2014	S1850	13.9	22	Fam-54_2009_	S1850	1850	4708	54	2009	W
2926_1716_S1851	2014	D	19.05.2014	S1851	14.5	25	Fam-57_2009_	S1851	1851	4709	57	2009	W
2926_1716_S1852	2014	D	19.05.2014	S1852	13	19	Fam-54_2009_	S1852	1852	4710	54	2009	W
2926_1716_S1853	2014	D	19.05.2014	S1853	13.2	20.5	Fam-60_2009_	S1853	1853	4711	60	2009	W
2926_1716_S1854	2014	D	19.05.2014	S1854	12.5	16	Fam-56_2009_	S1854	1854	4712	56	2009	W
2926_1716_S1855	2014	D	19.05.2014	S1855	13.1	19	Fam-54_2009_	S1855	1855	4713	54	2009	W
2926_1716_S1856	2014	D	19.05.2014	S1856	13.2	19	Fam-57_2009_	S1856	1856	4714	57	2009	W
2926_1716_S1857	2014	D	19.05.2014	S1857	15.3	28	Fam-57_2009_	S1857	1857	4715	57	2009	W

2926_1716_S1858	2014	D	19.05.2014	S1858	15	30	Fam-54_2009_	S1858	1858	4716	54	2009	W
2926_1716_S1859	2014	D	19.05.2014	S1859	13.7	21.5	Fam-54_2009_	S1859	1859	4717	54	2009	W
2926_1716_S1860	2014	D	19.05.2014	S1860	15.3	27.5	Fam-83_2010_	S1860	1860	4718	83	2010	W
2926_1716_S1861	2014	D	19.05.2014	S1861	13.3	20	Fam-59_2009_	S1861	1861	4719	59	2009	W
2926_1716_S1862	2014	D	19.05.2014	S1862	14.2	26	Fam-60_2009_	S1862	1862	4720	60	2009	W
2926_1716_S1863	2014	D	19.05.2014	S1863	15.3	31	Fam-48_2009_	S1863	1863	4721	48	2009	H
2926_1716_S1864	2014	D	19.05.2014	S1864	14.3	25	Fam-54_2009_	S1864	1864	4722	54	2009	W
2926_1716_S1865	2014	D	19.05.2014	S1865	12.8	16.5	Fam-70_2010_	S1865	1865	4723	70	2010	H
2926_1716_S1866	2014	D	19.05.2014	S1866	14.1	25	Fam-70_2010_	S1866	1866	4724	70	2010	H
2926_1716_S1867	2014	D	19.05.2014	S1867	15	26	Fam-51_2009_	S1867	1867	4725	51	2009	W
2926_1716_S1869	2014	D	19.05.2014	S1869	15.3	29.5	Fam-57_2009_	S1869	1869	4726	57	2009	W
2926_1716_S1870	2014	D	19.05.2014	S1870	15.3	28.5	Fam-40_2009_	S1870	1870	4727	40	2009	H
2926_1716_S1871	2014	D	19.05.2014	S1871	16	34	Fam-51_2009_	S1871	1871	4728	51	2009	W
2926_1716_S1872	2014	D	19.05.2014	S1872	14.3	24	Fam-34_2009_	S1872	1872	4729	34	2009	H

2926_1716_S1873	2014	D	19.05.2014	S1873	13.8	22.5	Fam-42_2009_	S1873	1873	4730	42	2009	H
2926_1716_S1874	2014	D	19.05.2014	S1874	14.5	26.5	Fam-45_2009_	S1874	1874	4731	45	2009	F
2926_1716_S1875	2014	D	19.05.2014	S1875	14.9	30	Fam-34_2009_	S1875	1875	4732	34	2009	H
2926_1716_S1876	2014	D	19.05.2014	S1876	12.8	19.5	Fam-90_2010_	S1876	1876	4733	90	2010	W
2926_1716_S1877	2014	D	19.05.2014	S1877	14.5	24	Fam-51_2009_	S1877	1877	4734	51	2009	W
2926_1716_S1878	2014	D	19.05.2014	S1878	15.8	33	Fam-60_2009_	S1878	1878	4735	60	2009	W
2926_1716_S1879	2014	D	19.05.2014	S1879	14	24	Fam-41_2009_	S1879	1879	4736	41	2009	F
2926_1716_S1880	2014	D	19.05.2014	S1880	14.3	24.5	Fam-57_2009_	S1880	1880	4737	57	2009	W
2926_1716_S1881	2014	D	19.05.2014	S1881	15	26.5	Fam-40_2009_	S1881	1881	4738	40	2009	H
2926_1716_S1882	2014	D	19.05.2014	S1882	13.9	22	Fam-38_2009_	S1882	1882	4739	38	2009	H
2926_1716_S1883	2014	D	19.05.2014	S1883	14.4	24.5	Fam-53_2009_	S1883	1883	4740	53	2009	W
2926_1716_S1884	2014	D	19.05.2014	S1884	14.6	26	Fam-85_2010_	S1884	1884	4741	85	2010	W
2926_1716_S1885	2014	D	19.05.2014	S1885	14.7	27	Fam-55_2009_	S1885	1885	4742	55	2009	W
2926_1716_S1886	2014	D	19.05.2014	S1886	14.9	26.5	Fam-52_2009_	S1886	1886	4743	52	2009	W

2926_1716_S1887	2014	D	19.05.2014	S1887	14.9	23.5	Fam-53_2009_	S1887	1887	4744	53	2009	W
2926_1716_S1888	2014	D	19.05.2014	S1888	14.3	22	Fam-59_2009_	S1888	1888	4745	59	2009	W
2926_1716_S1889	2014	D	19.05.2014	S1889	14.3	24.5	Fam-59_2009_	S1889	1889	4746	59	2009	W
2926_1716_S1890	2014	D	19.05.2014	S1890	13.2	18.5	Fam-59_2009_	S1890	1890	4747	59	2009	W
2926_1716_S1891	2014	D	19.05.2014	S1891	15.6	33.5	Fam-54_2009_	S1891	1891	4748	54	2009	W
2926_1716_S1892	2014	D	19.05.2014	S1892	14.7	25.5	Fam-53_2009_	S1892	1892	4749	53	2009	W
2926_1716_S1893	2014	D	19.05.2014	S1893	15.6	32	Fam-42_2009_	S1893	1893	4750	42	2009	H
2926_1716_S1894	2014	D	19.05.2014	S1894	13	19.5	Fam-81_2010_	S1894	1894	4751	81	2010	W
2926_1716_S1895	2014	D	19.05.2014	S1895	14.6	25	Fam-55_2009_	S1895	1895	4752	55	2009	W
2926_1716_S1896	2014	D	19.05.2014	S1896	15.6	29	Fam-57_2009_	S1896	1896	4753	57	2009	W
2926_1717_S1897	2014	D	19.05.2014	S1897	14.5	25.5	Fam-34_2009_	S1897	1897	4754	34	2009	H
2926_1717_S1898	2014	D	19.05.2014	S1898	14.3	25	Fam-55_2009_	S1898	1898	4755	55	2009	W
2926_1717_S1899	2014	D	19.05.2014	S1899	14.7	24.5	Fam-57_2009_	S1899	1899	4756	57	2009	W
2926_1717_S1900	2014	D	19.05.2014	S1900	13.7	21.5	Fam-85_2010_	S1900	1900	4757	85	2010	W

2926_1717_S1901	2014	D	19.05.2014	S1901	14.3	23	Fam-51_2009_	S1901	1901	4758	51	2009	W
2926_1717_S1902	2014	D	19.05.2014	S1902	14.1	24	Fam-68_2010_	S1902	1902	4759	68	2010	H
2926_1717_S1903	2014	D	19.05.2014	S1903	14.2	24.5	Fam-55_2009_	S1903	1903	4760	55	2009	W
2926_1717_S1904	2014	D	19.05.2014	S1904	15.1	27	Fam-76_2010_	S1904	1904	4761	76	2010	H
2926_1717_S1905	2014	D	19.05.2014	S1905	14.5	24	Fam-32_2009_	S1905	1905	4762	32	2009	H
2926_1717_S1906	2014	D	19.05.2014	S1906	14.9	26.5	Fam-40_2009_	S1906	1906	4763	40	2009	H
2926_1717_S1907	2014	D	19.05.2014	S1907	14.6	23.5	Fam-54_2009_	S1907	1907	4764	54	2009	W
2926_1717_S1908	2014	D	19.05.2014	S1908	12.7	16	Fam-59_2009_	S1908	1908	4765	59	2009	W
2926_1717_S1909	2014	D	19.05.2014	S1909	14.4	24.5	Fam-57_2009_	S1909	1909	4766	57	2009	W
2926_1717_S1910	2014	D	19.05.2014	S1910	13.6	21.5	Fam-68_2010_	S1910	1910	4767	68	2010	H
2926_1717_S1911	2014	D	19.05.2014	S1911	15.8	31.5	Fam-57_2009_	S1911	1911	4768	57	2009	W
2926_1717_S1912	2014	D	19.05.2014	S1912	13.9	24	Fam-83_2010_	S1912	1912	4769	83	2010	W
2926_1717_S1913	2014	D	19.05.2014	S1913	15	26.5	Fam-54_2009_	S1913	1913	4770	54	2009	W
2926_1717_S1914	2014	D	19.05.2014	S1914	13	19	Fam-52_2009_	S1914	1914	4771	52	2009	W

2926_1717_S1915	2014	D	19.05.2014	S1915	15.9	32.5	Fam-53_2009_	S1915	1915	4772	53	2009	W
2926_1717_S1916	2014	D	19.05.2014	S1916	13.7	21	Fam-52_2009_	S1916	1916	4773	52	2009	W
2926_1717_S1917	2014	D	19.05.2014	S1917	13.1	18	Fam-85_2010_	S1917	1917	4774	85	2010	W
2926_1717_S1918	2014	D	19.05.2014	S1918	13.5	18.5	Fam-84_2010_	S1918	1918	4775	84	2010	W
2926_1717_S1919	2014	D	19.05.2014	S1919	13.9	22.5	Fam-41_2009_	S1919	1919	4776	41	2009	F
2926_1717_S1920	2014	D	19.05.2014	S1920	15.8	33	Fam-40_2009_	S1920	1920	4777	40	2009	H
2927_1717_S1921	2014	D	19.05.2014	S1921	14.9	27.5	Fam-55_2009_	S1921	1921	4778	55	2009	W
2927_1717_S1922	2014	D	19.05.2014	S1922	13.3	19.5	Fam-53_2009_	S1922	1922	4779	53	2009	W
2927_1717_S1923	2014	D	19.05.2014	S1923	15	26.5	Fam-57_2009_	S1923	1923	4780	57	2009	W
2927_1717_S1924	2014	D	19.05.2014	S1924	14.3	25	Fam-51_2009_	S1924	1924	4781	51	2009	W
2927_1717_S1925	2014	D	19.05.2014	S1925	11.6	13	Fam-62_1_201	S1925	1925	4782	62	2010	H
2927_1717_S1926	2014	D	19.05.2014	S1926	15.2	27	Fam-54_2009_	S1926	1926	4783	54	2009	W
2927_1717_S1927	2014	D	19.05.2014	S1927	12.5	16	Fam-81_2010_	S1927	1927	4784	81	2010	W
2927_1717_S1928	2014	D	19.05.2014	S1928	14.4	27	Fam-60_2009_	S1928	1928	4785	60	2009	W

2927_1717_S1929	2014	D	20.05.2014	S1929	13.7	21.5	Fam-48_2009_	S1929	1929	4786	48	2009	H
2927_1717_S1930	2014	D	20.05.2014	S1930	13.5	19.5	Fam-82_2010_	S1930	1930	4787	82	2010	W
2927_1717_S1931	2014	D	20.05.2014	S1931	13.6	21.5	Fam-42_2009_	S1931	1931	4788	42	2009	H
2927_1717_S1932	2014	D	20.05.2014	S1932	14.8	27	Fam-55_2009_	S1932	1932	4789	55	2009	W
2927_1717_S1933	2014	D	20.05.2014	S1933	13.8	20.5	Fam-72_2010_	S1933	1933	4790	72	2010	H
2927_1717_S1934	2014	D	20.05.2014	S1934	15	28.5	Fam-48_2009_	S1934	1934	4791	48	2009	H
2927_1717_S1935	2014	D	20.05.2014	S1935	16	32.5	Fam-55_2009_	S1935	1935	4792	55	2009	W
2927_1717_S1936	2014	D	20.05.2014	S1936	15.1	30	Fam-45_2009_	S1936	1936	4793	45	2009	F
2927_1717_S1937	2014	D	20.05.2014	S1937	14.6	25.5	Fam-34_2009_	S1937	1937	4794	34	2009	H
2927_1717_S1938	2014	D	20.05.2014	S1938	15.7	33	Fam-54_2009_	S1938	1938	4795	54	2009	W
2927_1717_S1939	2014	D	20.05.2014	S1939	14.3	25.5	Fam-45_2009_	S1939	1939	4796	45	2009	F
2927_1717_S1940	2014	D	20.05.2014	S1940	13.9	23	Fam-54_2009_	S1940	1940	4797	54	2009	W
2927_1717_S1941	2014	D	20.05.2014	S1941	15.1	26.5	Fam-32_2009_	S1941	1941	4798	32	2009	H
2927_1717_S1942	2014	D	20.05.2014	S1942	14.6	27	Fam-31_2009_	S1942	1942	4799	31	2009	F

2927_1717_S1943	2014	D	20.05.2014	S1943	15.2	32.5	Fam-60_2009_	S1943	1943	4800	60	2009	W
2927_1717_S1944	2014	D	20.05.2014	S1944	13.7	21.5	Fam-62_1_201	S1944	1944	4801	62	2010	H
2927_1717_S1945	2014	D	20.05.2014	S1945	14.7	26.5	Fam-40_2009_	S1945	1945	4802	40	2009	H
2927_1717_S1946	2014	D	20.05.2014	S1946	13.2	20	Fam-90_2010_	S1946	1946	4803	90	2010	W
2927_1717_S1947	2014	D	20.05.2014	S1947	15.1	27.5	Fam-62_1_201	S1947	1947	4804	62	2010	H
2927_1717_S1948	2014	D	20.05.2014	S1948	16.2	31.5	Fam-51_2009_	S1948	1948	4805	51	2009	W
2927_1717_S1949	2014	D	20.05.2014	S1949	14.5	23	Fam-51_2009_	S1949	1949	4806	51	2009	W
2927_1717_S1950	2014	D	20.05.2014	S1950	13.7	20.5	Fam-55_2009_	S1950	1950	4807	55	2009	W
2927_1717_S1952	2014	D	20.05.2014	S1952	14.7	26.5	Fam-58_2009_	S1952	1952	4808	58	2009	W
2927_1717_S1953	2014	D	20.05.2014	S1953	14.9	27	Fam-55_2009_	S1953	1953	4809	55	2009	W
2927_1717_S1954	2014	D	20.05.2014	S1954	14.5	26.5	Fam-55_2009_	S1954	1954	4810	55	2009	W
2927_1717_S1955	2014	D	20.05.2014	S1955	14.8	24.5	Fam-55_2009_	S1955	1955	4811	55	2009	W
2927_1717_S1956	2014	D	20.05.2014	S1956	14.4	23	Fam-55_2009_	S1956	1956	4812	55	2009	W
2927_1717_S1957	2014	D	20.05.2014	S1957	12.7	17	Fam-59_2009_	S1957	1957	4813	59	2009	W

2927_1717_S1958	2014	D	20.05.2014	S1958	13.3	19.5	Fam-44_2009_	S1958	1958	4814	44	2009 H
2927_1717_S1959	2014	D	20.05.2014	S1959	13.4	20	Fam-59_2009_	S1959	1959	4815	59	2009 W
2927_1717_S1960	2014	D	20.05.2014	S1960	15.5	29	Fam-51_2009_	S1960	1960	4816	51	2009 W
2927_1717_S1961	2014	D	20.05.2014	S1961	15.7	34	Fam-42_2009_	S1961	1961	4817	42	2009 H
2927_1717_S1962	2014	T	20.05.2014	S1962	16	31	Family-32_200	S1962	1962	4818	32	2009 H
2927_1717_S1963	2014	D	20.05.2014	S1963	14.7	25.5	Fam-53_2009_	S1963	1963	4819	53	2009 W
2927_1717_S1964	2014	D	20.05.2014	S1964	14.7	29	Fam-60_2009_	S1964	1964	4820	60	2009 W
2927_1717_S1965	2014	D	20.05.2014	S1965	14	23	Fam-55_2009_	S1965	1965	4821	55	2009 W
2927_1717_S1966	2014	D	20.05.2014	S1966	14.5	25	Fam-54_2009_	S1966	1966	4822	54	2009 W
2927_1717_S1967	2014	D	20.05.2014	S1967	13.7	21.5	Fam-90_2010_	S1967	1967	4823	90	2010 W
2927_1717_S1968	2014	D	20.05.2014	S1968	15.7	31.5	Fam-40_2009_	S1968	1968	4824	40	2009 H
2927_1717_S1969	2014	D	20.05.2014	S1969	14.7	25.5	Fam-32_2009_	S1969	1969	4825	32	2009 H
2927_1717_S1970	2014	D	20.05.2014	S1970	15.6	32	Fam-54_2009_	S1970	1970	4826	54	2009 W
2927_1717_S1971	2014	D	20.05.2014	S1971	15.8	31.5	Fam-55_2009_	S1971	1971	4827	55	2009 W

2927_1717_S1972	2014	D	20.05.2014	S1972	14	23	Fam-36_2009_S1972	1972	4828	36	2009	H
2927_1717_S1973	2014	D	20.05.2014	S1973	15.1	28	Fam-34_2009_S1973	1973	4829	34	2009	H
2927_1717_S1974	2014	D	20.05.2014	S1974	14.5	25	Fam-32_2009_S1974	1974	4830	32	2009	H
2927_1717_S1975	2014	D	20.05.2014	S1975	14.2	23	Fam-55_2009_S1975	1975	4831	55	2009	W
2927_1717_S1976	2014	D	20.05.2014	S1976	15.8	32.5	Fam-83_2010_S1976	1976	4832	83	2010	W
2927_1717_S1977	2014	D	20.05.2014	S1977	13.2	18.5	Fam-59_2009_S1977	1977	4833	59	2009	W
2927_1717_S1978	2014	D	20.05.2014	S1978	14.8	27	Fam-40_2009_S1978	1978	4834	40	2009	H
2927_1717_S1979	2014	D	20.05.2014	S1979	14.7	26	Fam-87_2010_S1979	1979	4835	87	2010	W
2927_1717_S1980	2014	D	20.05.2014	S1980	14.6	24	Fam-55_2009_S1980	1980	4836	55	2009	W
2927_1717_S1981	2014	D	20.05.2014	S1981	15.3	30.5	Fam-32_2009_S1981	1981	4837	32	2009	H
2927_1717_S1982	2014	D	20.05.2014	S1982	12.1	15.5	Fam-56_2009_S1982	1982	4838	56	2009	W
2927_1717_S1983	2014	D	20.05.2014	S1983	14.6	26.5	Fam-58_2009_S1983	1983	4839	58	2009	W
2927_1717_S1984	2014	D	20.05.2014	S1984	14.2	23.5	Fam-57_2009_S1984	1984	4840	57	2009	W
2927_1717_S1985	2014	D	20.05.2014	S1985	14.6	24.5	Fam-53_2009_S1985	1985	4841	53	2009	W

2927_1717_S1986	2014	D	20.05.2014	S1986	14.7	26	Fam-54_2009_	S1986	1986	4842	54	2009	W
2927_1717_S1987	2014	D	20.05.2014	S1987	13.8	21.5	Fam-58_2009_	S1987	1987	4843	58	2009	W
2927_1717_S1988	2014	D	20.05.2014	S1988	14	22.5	Fam-54_2009_	S1988	1988	4844	54	2009	W
2927_1717_S1989	2014	D	20.05.2014	S1989	14.7	26	Fam-53_2009_	S1989	1989	4845	53	2009	W
2927_1717_S1990	2014	D	20.05.2014	S1990	14.7	24.5	Fam-51_2009_	S1990	1990	4846	51	2009	W
2927_1718_S1991	2014	D	20.05.2014	S1991	12.7	17.5	Fam-87_2010_	S1991	1991	4847	87	2010	W
2927_1718_S1992	2014	D	20.05.2014	S1992	13.6	21.5	Fam-54_2009_	S1992	1992	4848	54	2009	W
2927_1718_S1993	2014	D	20.05.2014	S1993	15.7	32.5	Fam-60_2009_	S1993	1993	4849	60	2009	W
2927_1718_S1994	2014	D	20.05.2014	S1994	14.3	25	Fam-55_2009_	S1994	1994	4850	55	2009	W
2927_1718_S1995	2014	D	20.05.2014	S1995	14.2	24.5	Fam-34_2009_	S1995	1995	4851	34	2009	H
2927_1718_S1996	2014	D	20.05.2014	S1996	13.7	21	Fam-53_2009_	S1996	1996	4852	53	2009	W
2927_1718_S1997	2014	D	20.05.2014	S1997	14.3	25.5	Fam-85_2010_	S1997	1997	4853	85	2010	W
2927_1718_S1998	2014	D	20.05.2014	S1998	14.2	24	Fam-60_2009_	S1998	1998	4854	60	2009	W
2927_1718_S2000	2014	D	20.05.2014	S2000	14.4	24.5	Fam-52_2009_	S2000	2000	4855	52	2009	W

2928_1718_S2001	2014	D	20.05.2014	S2001	12.6	16.5	Fam-85_2010_S2001	2001	4856	85	2010	W
2928_1718_S2002	2014	D	20.05.2014	S2002	11.2	11.5	Fam-70_2010_S2002	2002	4857	70	2010	H
2928_1718_S2003	2014	D	20.05.2014	S2003	13.9	22	Fam-50_2009_S2003	2003	4858	50	2009	H
2928_1718_S2004	2014	D	20.05.2014	S2004	12.8	19	Fam-89_2010_S2004	2004	4859	89	2010	W
2928_1718_S2005	2014	D	20.05.2014	S2005	16.8	38	Fam-34_2009_S2005	2005	4860	34	2009	H
2928_1718_S2006	2014	D	20.05.2014	S2006	12.7	16	Fam-51_2009_S2006	2006	4861	51	2009	W
2928_1718_S2007	2014	D	20.05.2014	S2007	13.8	23	Fam-54_2009_S2007	2007	4862	54	2009	W
2928_1718_S2008	2014	D	20.05.2014	S2008	14.8	27	Fam-56_2009_S2008	2008	4863	56	2009	W
2928_1718_S2009	2014	D	20.05.2014	S2009	14	22.5	Fam-59_2009_S2009	2009	4864	59	2009	W
2928_1718_S2010	2014	D	20.05.2014	S2010	15	25.5	Fam-53_2009_S2010	2010	4865	53	2009	W
2928_1718_S2011	2014	D	20.05.2014	S2011	14.4	24.5	Fam-54_2009_S2011	2011	4866	54	2009	W
2928_1718_S2012	2014	D	20.05.2014	S2012	14.6	24.5	Fam-40_2009_S2012	2012	4867	40	2009	H
2928_1718_S2013	2014	D	20.05.2014	S2013	13.8	21.5	Fam-58_2009_S2013	2013	4868	58	2009	W
2928_1718_S2014	2014	D	20.05.2014	S2014	16.2	34.5	Fam-60_2009_S2014	2014	4869	60	2009	W

2928_1718_S2015	2014	D	20.05.2014	S2015	15.1	30.5	Fam-58_2009_	S2015	2015	4870	58	2009	W
2928_1718_S2016	2014	D	20.05.2014	S2016	14.2	24.5	Fam-40_2009_	S2016	2016	4871	40	2009	H
2928_1718_S2017	2014	D	20.05.2014	S2017	12.5	18	Fam-68_2010_	S2017	2017	4872	68	2010	H
2928_1718_S2018	2014	D	20.05.2014	S2018	13.9	24	Fam-54_2009_	S2018	2018	4873	54	2009	W
2928_1718_S2019	2014	D	20.05.2014	S2019	14.8	24	Fam-57_2009_	S2019	2019	4874	57	2009	W
2928_1718_S2020	2014	D	20.05.2014	S2020	13.8	22.5	Fam-85_2010_	S2020	2020	4875	85	2010	W
2928_1718_S2021	2014	D	20.05.2014	S2021	13.6	21.5	Fam-32_2009_	S2021	2021	4876	32	2009	H
2928_1718_S2022	2014	D	20.05.2014	S2022	14	22.5	Fam-54_2009_	S2022	2022	4877	54	2009	W
2928_1718_S2023	2014	D	20.05.2014	S2023	13.2	19	Fam-59_2009_	S2023	2023	4878	59	2009	W
2928_1718_S2024	2014	D	20.05.2014	S2024	13.9	23.5	Fam-58_2009_	S2024	2024	4879	58	2009	W
2928_1718_S2025	2014	D	20.05.2014	S2025	14.1	23	Fam-59_2009_	S2025	2025	4880	59	2009	W
2928_1718_S2026	2014	D	20.05.2014	S2026	14.2	24.5	Fam-44_2009_	S2026	2026	4881	44	2009	H
2928_1718_S2027	2014	D	20.05.2014	S2027	16.6	35.5	Fam-53_2009_	S2027	2027	4882	53	2009	W
2928_1718_S2028	2014	D	20.05.2014	S2028	13.6	21.5	Fam-36_2009_	S2028	2028	4883	36	2009	H

2928_1718_S2029	2014	D	20.05.2014	S2029	14.3	24	Fam-55_2009_	S2029	2029	4884	55	2009	W
2928_1718_S2030	2014	D	20.05.2014	S2030	13.2	19	Fam-54_2009_	S2030	2030	4885	54	2009	W
2928_1718_S2031	2014	D	20.05.2014	S2031	16.6	38.5	Fam-57_2009_	S2031	2031	4886	57	2009	W
2928_1718_S2032	2014	D	20.05.2014	S2032	15	28.5	Fam-41_2009_	S2032	2032	4887	41	2009	F
2928_1718_S2033	2014	D	20.05.2014	S2033	14	22.5	Fam-54_2009_	S2033	2033	4888	54	2009	W
2928_1718_S2034	2014	D	20.05.2014	S2034	15.5	28.5	Fam-52_2009_	S2034	2034	4889	52	2009	W
2928_1718_S2035	2014	D	20.05.2014	S2035	14.7	26	Fam-55_2009_	S2035	2035	4890	55	2009	W
2928_1718_S2036	2014	D	20.05.2014	S2036	15.8	34.5	Fam-54_2009_	S2036	2036	4891	54	2009	W
2928_1718_S2037	2014	D	20.05.2014	S2037	17.1	44	Fam-73_2010_	S2037	2037	4892	73	2010	F
2928_1718_S2038	2014	D	20.05.2014	S2038	14.6	26	Fam-54_2009_	S2038	2038	4893	54	2009	W
2928_1718_S2039	2014	D	20.05.2014	S2039	14.4	25	Fam-51_2009_	S2039	2039	4894	51	2009	W
2928_1718_S2040	2014	D	20.05.2014	S2040	15.3	29	Fam-40_2009_	S2040	2040	4895	40	2009	H
2928_1718_S2041	2014	D	20.05.2014	S2041	13.7	22.5	Fam-57_2009_	S2041	2041	4896	57	2009	W
2928_1718_S2042	2014	D	20.05.2014	S2042	15.6	33.5	Fam-42_2009_	S2042	2042	4897	42	2009	H

2928_1718_S2043	2014	D	20.05.2014	S2043	14.4	25.5	Fam-42_2009_	S2043	2043	4898	42	2009	H
2928_1718_S2044	2014	D	20.05.2014	S2044	13.2	19	Fam-59_2009_	S2044	2044	4899	59	2009	W
2928_1718_S2045	2014	D	20.05.2014	S2045	14.4	26	Fam-59_2009_	S2045	2045	4900	59	2009	W
2928_1718_S2046	2014	D	20.05.2014	S2046	13.9	22	Fam-54_2009_	S2046	2046	4901	54	2009	W
2928_1718_S2047	2014	D	20.05.2014	S2047	15.6	31	Fam-40_2009_	S2047	2047	4902	40	2009	H
2928_1718_S2048	2014	D	20.05.2014	S2048	15	29	Fam-55_2009_	S2048	2048	4903	55	2009	W
2928_1718_S2049	2014	D	20.05.2014	S2049	13.7	22.5	Fam-59_2009_	S2049	2049	4904	59	2009	W
2928_1718_S2050	2014	D	20.05.2014	S2050	15.4	29.5	Fam-57_2009_	S2050	2050	4905	57	2009	W
2928_1718_S2051	2014	D	20.05.2014	S2051	15.3	31	Fam-66_2010_	S2051	2051	4906	66	2010	H
2928_1718_S2052	2014	D	20.05.2014	S2052	13	20	Fam-87_2010_	S2052	2052	4907	87	2010	W
2928_1718_S2053	2014	D	20.05.2014	S2053	13.7	21.5	Fam-82_2010_	S2053	2053	4908	82	2010	W
2928_1718_S2054	2014	D	20.05.2014	S2054	13.5	20.5	Fam-32_2009_	S2054	2054	4909	32	2009	H
2928_1718_S2055	2014	D	20.05.2014	S2055	13.5	21.5	Fam-39_2009_	S2055	2055	4910	39	2009	F
2928_1718_S2056	2014	D	20.05.2014	S2056	14.6	26	Fam-56_2009_	S2056	2056	4911	56	2009	W

2928_1718_S2057	2014	D	20.05.2014	S2057	14	24	Fam-56_2009_	S2057	2057	4912	56	2009	W
2928_1718_S2058	2014	D	20.05.2014	S2058	14.7	26.5	Fam-55_2009_	S2058	2058	4913	55	2009	W
2928_1718_S2059	2014	D	20.05.2014	S2059	15.7	29	Fam-50_2009_	S2059	2059	4914	50	2009	H
2928_1718_S2060	2014	D	20.05.2014	S2060	15.5	30.5	Fam-42_2009_	S2060	2060	4915	42	2009	H
2928_1718_S2061	2014	D	20.05.2014	S2061	13.8	23.5	Fam-54_2009_	S2061	2061	4916	54	2009	W
2928_1718_S2062	2014	D	20.05.2014	S2062	14.3	25	Fam-54_2009_	S2062	2062	4917	54	2009	W
2928_1718_S2063	2014	D	20.05.2014	S2063	14.1	24	Fam-59_2009_	S2063	2063	4918	59	2009	W
2928_1718_S2064	2014	D	20.05.2014	S2064	13.9	22.5	Fam-54_2009_	S2064	2064	4919	54	2009	W
2928_1718_S2065	2014	D	20.05.2014	S2065	15.7	33	Fam-81_2010_	S2065	2065	4920	81	2010	W
2928_1718_S2066	2014	D	20.05.2014	S2066	14.9	27.5	Fam-53_2009_	S2066	2066	4921	53	2009	W
2928_1718_S2067	2014	D	20.05.2014	S2067	15.8	32.5	Fam-35_2009_	S2067	2067	4922	35	2009	F
2928_1718_S2068	2014	D	20.05.2014	S2068	13.9	22	Fam-55_2009_	S2068	2068	4923	55	2009	W
2928_1718_S2069	2014	D	20.05.2014	S2069	12.4	16	Fam-38_2009_	S2069	2069	4924	38	2009	H
2928_1718_S2070	2014	D	20.05.2014	S2070	14.2	24	Fam-32_2009_	S2070	2070	4925	32	2009	H

2928_1718_S2071	2014	D	20.05.2014	S2071	14.1	22.5	Fam-55_2009_	S2071	2071	4926	55	2009	W
2928_1718_S2072	2014	D	20.05.2014	S2072	15.5	29	Fam-60_2009_	S2072	2072	4927	60	2009	W
2928_1718_S2073	2014	D	20.05.2014	S2073	15.2	26.5	Fam-57_2009_	S2073	2073	4928	57	2009	W
2928_1718_S2074	2014	D	20.05.2014	S2074	13	17.5	Fam-59_2009_	S2074	2074	4929	59	2009	W
2928_1718_S2075	2014	D	20.05.2014	S2075	14.8	25	Fam-60_2009_	S2075	2075	4930	60	2009	W
2928_1718_S2076	2014	D	20.05.2014	S2076	13.1	19	Fam-56_2009_	S2076	2076	4931	56	2009	W
2928_1718_S2077	2014	D	20.05.2014	S2077	13.8	19.5	Fam-82_2010_	S2077	2077	4932	82	2010	W
2928_1718_S2078	2014	D	20.05.2014	S2078	13.1	18	Fam-62_1_201	S2078	2078	4933	62	2010	H
2928_1718_S2079	2014	D	20.05.2014	S2079	13.2	18	Fam-55_2009_	S2079	2079	4934	55	2009	W
2928_1718_S2080	2014	D	20.05.2014	S2080	13.1	18	Fam-59_2009_	S2080	2080	4935	59	2009	W
2929_1718_S2081	2014	D	20.05.2014	S2081	13.8	21.5	Fam-60_2009_	S2081	2081	4936	60	2009	W
2929_1718_S2082	2014	D	20.05.2014	S2082	15.9	31.5	Fam-60_2009_	S2082	2082	4937	60	2009	W
2929_1718_S2083	2014	D	20.05.2014	S2083	14.6	24.5	Fam-58_2009_	S2083	2083	4938	58	2009	W
2929_1718_S2084	2014	T	20.05.2014	S2084	14	22	Family-42_200	S2084	2084	4939	42	2009	H

2929_1719_S2085	2014	D	20.05.2014	S2085	16	32	Fam-55_2009_	S2085	2085	4940	55	2009	W
2929_1719_S2086	2014	D	20.05.2014	S2086	15.2	26.5	Fam-51_2009_	S2086	2086	4941	51	2009	W
2929_1719_S2087	2014	D	20.05.2014	S2087	13.5	18	Fam-59_2009_	S2087	2087	4942	59	2009	W
2929_1719_S2088	2014	D	20.05.2014	S2088	14.7	26	Fam-87_2010_	S2088	2088	4943	87	2010	W
2929_1719_S2089	2014	D	20.05.2014	S2089	15.6	31	Fam-40_2009_	S2089	2089	4944	40	2009	H
2929_1719_S2090	2014	D	20.05.2014	S2090	13.5	20	Fam-44_2009_	S2090	2090	4945	44	2009	H
2929_1719_S2092	2014	D	20.05.2014	S2092	14.1	21.5	Fam-52_2009_	S2092	2092	4946	52	2009	W
2929_1719_S2093	2014	D	20.05.2014	S2093	14.3	23	Fam-60_2009_	S2093	2093	4947	60	2009	W
2929_1719_S2094	2014	D	20.05.2014	S2094	13.2	19	Fam-54_2009_	S2094	2094	4948	54	2009	W
2929_1719_S2095	2014	D	20.05.2014	S2095	14.9	26.5	Fam-60_2009_	S2095	2095	4949	60	2009	W
2929_1719_S2096	2014	D	20.05.2014	S2096	15.2	26.5	Fam-40_2009_	S2096	2096	4950	40	2009	H
2929_1719_S2097	2014	D	20.05.2014	S2097	12.2	14.5	Fam-58_2009_	S2097	2097	4951	58	2009	W
2929_1719_S2098	2014	D	20.05.2014	S2098	13	16.5	Fam-73_2010_	S2098	2098	4952	73	2010	F
2929_1719_S2099	2014	D	20.05.2014	S2099	15.3	28.5	Fam-32_2009_	S2099	2099	4953	32	2009	H

2929_1719_S210(	2014	D	20.05.2014	S2100	14.1	22.5	Fam-60_2009_	S2100	2100	4954	60	2009	W
2929_1719_S2101	2014	D	20.05.2014	S2101	13	17	Fam-57_2009_	S2101	2101	4955	57	2009	W
2929_1719_S2102	2014	D	20.05.2014	S2102	13.6	21	Fam-54_2009_	S2102	2102	4956	54	2009	W
2929_1719_S2103	2014	D	20.05.2014	S2103	14.3	24.5	Fam-42_2009_	S2103	2103	4957	42	2009	H
2929_1719_S2104	2014	D	20.05.2014	S2104	14	23.5	Fam-30_2007_	S2104	2104	4958	30	2007	H
2929_1719_S210€	2014	D	20.05.2014	S2106	13.8	21.5	Fam-58_2009_	S2106	2106	4959	58	2009	W
2929_1719_S2107	2014	D	20.05.2014	S2107	15.5	29.5	Fam-54_2009_	S2107	2107	4960	54	2009	W
2929_1719_S210S	2014	D	20.05.2014	S2109	14	21	Fam-59_2009_	S2109	2109	4961	59	2009	W
2929_1719_S211(	2014	D	20.05.2014	S2110	15	27	Fam-60_2009_	S2110	2110	4962	60	2009	W
2929_1719_S2111	2014	D	20.05.2014	S2111	15	26.5	Fam-51_2009_	S2111	2111	4963	51	2009	W
2929_1719_S2113	2014	D	20.05.2014	S2113	15.5	30	Fam-60_2009_	S2113	2113	4964	60	2009	W
2929_1719_S2114	2014	D	20.05.2014	S2114	14.5	23.5	Fam-55_2009_	S2114	2114	4965	55	2009	W
2929_1719_S2115	2014	D	20.05.2014	S2115	12.9	18	Fam-54_2009_	S2115	2115	4966	54	2009	W
2929_1719_S211€	2014	D	20.05.2014	S2116	14.5	24.5	Fam-57_2009_	S2116	2116	4967	57	2009	W

2929_1719_S2117	2014	D	20.05.2014	S2117	12.9	16.5	Fam-54_2009_S2117	2117	4968	54	2009	W
2929_1719_S2118	2014	D	20.05.2014	S2118	15.4	29.5	Fam-74_2010_S2118	2118	4969	74	2010	H
2929_1719_S2119	2014	D	20.05.2014	S2119	14.1	21.5	Fam-54_2009_S2119	2119	4970	54	2009	W
2929_1719_S2120	2014	D	20.05.2014	S2120	14.5	25	Fam-60_2009_S2120	2120	4971	60	2009	W
2929_1719_S2121	2014	D	20.05.2014	S2121	14.2	25	Fam-42_2009_S2121	2121	4972	42	2009	H
2929_1719_S2122	2014	D	20.05.2014	S2122	13.6	21	Fam-32_2009_S2122	2122	4973	32	2009	H
2929_1719_S2123	2014	D	20.05.2014	S2123	13.9	23	Fam-58_2009_S2123	2123	4974	58	2009	W
2929_1719_S2124	2014	D	20.05.2014	S2124	14	23	Fam-56_2009_S2124	2124	4975	56	2009	W
2929_1719_S2125	2014	D	20.05.2014	S2125	13.8	22.5	Fam-60_2009_S2125	2125	4976	60	2009	W
2929_1719_S2126	2014	D	20.05.2014	S2126	13.7	22	Fam-55_2009_S2126	2126	4977	55	2009	W
2929_1719_S2127	2014	D	20.05.2014	S2127	15.1	30	Fam-38_2009_S2127	2127	4978	38	2009	H
2929_1719_S2128	2014	D	20.05.2014	S2128	13.4	20	Fam-52_2009_S2128	2128	4979	52	2009	W
2929_1719_S2129	2014	D	20.05.2014	S2129	14.3	23.5	Fam-51_2009_S2129	2129	4980	51	2009	W
2929_1719_S2130	2014	D	20.05.2014	S2130	14.6	26.5	Fam-51_2009_S2130	2130	4981	51	2009	W

2929_1719_S2131	2014	D	20.05.2014	S2131	14.2	24	Fam-54_2009_	S2131	2131	4982	54	2009	W
2929_1719_S2132	2014	D	20.05.2014	S2132	14.3	24.5	Fam-54_2009_	S2132	2132	4983	54	2009	W
2929_1719_S2133	2014	D	20.05.2014	S2133	12.2	15.5	Fam-58_2009_	S2133	2133	4984	58	2009	W
2929_1719_S2134	2014	D	20.05.2014	S2134	13.6	21	Fam-55_2009_	S2134	2134	4985	55	2009	W
2929_1719_S2135	2014	D	20.05.2014	S2135	13.9	22	Fam-59_2009_	S2135	2135	4986	59	2009	W
2929_1719_S2136	2014	D	20.05.2014	S2136	13.8	22	Fam-81_2010_	S2136	2136	4987	81	2010	W
2929_1719_S2137	2014	D	20.05.2014	S2137	13.8	22	Fam-86_2010_	S2137	2137	4988	86	2010	W
2929_1719_S2138	2014	D	20.05.2014	S2138	13.7	20.5	Fam-42_2009_	S2138	2138	4989	42	2009	H
2929_1719_S2139	2014	D	20.05.2014	S2139	13.2	17.5	Fam-54_2009_	S2139	2139	4990	54	2009	W
2929_1719_S2140	2014	D	20.05.2014	S2140	13.6	22	Fam-50_2009_	S2140	2140	4991	50	2009	H
2929_1719_S2141	2014	D	21.05.2014	S2141	16.7	38.5	Fam-58_2009_	S2141	2141	4992	58	2009	W
2929_1719_S2142	2014	T	21.05.2014	S2142	13.8	20.5	Family-40_200	S2142	2142	4993	40	2009	H
2929_1719_S2143	2014	D	21.05.2014	S2143	13.2	19	Fam-59_2009_	S2143	2143	4994	59	2009	W
2929_1719_S2144	2014	D	21.05.2014	S2144	12.6	16.5	Fam-57_2009_	S2144	2144	4995	57	2009	W

2929_1719_S2145	2014	D	21.05.2014	S2145	15.6	31.5	Fam-55_2009_	S2145	2145	4996	55	2009	W
2929_1719_S2147	2014	D	21.05.2014	S2147	14.2	22.5	Fam-57_2009_	S2147	2147	4997	57	2009	W
2929_1719_S2148	2014	D	21.05.2014	S2148	14.4	25	Fam-40_2009_	S2148	2148	4998	40	2009	H
2929_1719_S2149	2014	D	21.05.2014	S2149	13.7	22.5	Fam-82_2010_	S2149	2149	4999	82	2010	W
2929_1719_S2150	2014	D	21.05.2014	S2150	14.7	26.5	Fam-34_2009_	S2150	2150	5000	34	2009	H
2929_1719_S2151	2014	D	21.05.2014	S2151	14.2	23.5	Fam-59_2009_	S2151	2151	5001	59	2009	W
2929_1719_S2152	2014	D	21.05.2014	S2152	13.2	19	Fam-57_2009_	S2152	2152	5002	57	2009	W
2929_1719_S2153	2014	D	21.05.2014	S2153	14.8	25	Fam-59_2009_	S2153	2153	5003	59	2009	W
2929_1719_S2154	2014	D	21.05.2014	S2154	14.2	23.5	Fam-54_2009_	S2154	2154	5004	54	2009	W
2929_1719_S2155	2014	D	21.05.2014	S2155	13.2	20.5	Fam-44_2009_	S2155	2155	5005	44	2009	H
2929_1719_S2156	2014	D	21.05.2014	S2156	14.3	24.5	Fam-54_2009_	S2156	2156	5006	54	2009	W
2929_1719_S2157	2014	D	21.05.2014	S2157	13.6	21	Fam-59_2009_	S2157	2157	5007	59	2009	W
2929_1719_S2158	2014	D	21.05.2014	S2158	13.9	22	Fam-60_2009_	S2158	2158	5008	60	2009	W
2929_1719_S2159	2014	D	21.05.2014	S2159	12.5	16.5	Fam-81_2010_	S2159	2159	5009	81	2010	W

2929_1719_S2160	2014	D	21.05.2014	S2160	12.2	16.5	Fam-42_2009_	S2160	2160	5010	42	2009	H
2930_1719_S2161	2014	D	21.05.2014	S2161	14.7	26	Fam-56_2009_	S2161	2161	5011	56	2009	W
2930_1719_S2162	2014	D	21.05.2014	S2162	15.2	29.5	Fam-58_2009_	S2162	2162	5012	58	2009	W
2930_1719_S2164	2014	D	21.05.2014	S2164	16.7	35	Fam-57_2009_	S2164	2164	5013	57	2009	W
2930_1719_S2165	2014	D	21.05.2014	S2165	12.7	18	Fam-90_2010_	S2165	2165	5014	90	2010	W
2930_1719_S2166	2014	D	21.05.2014	S2166	13.9	21	Fam-82_2010_	S2166	2166	5015	82	2010	W
2930_1719_S2167	2014	D	21.05.2014	S2167	13.8	23	Fam-60_2009_	S2167	2167	5016	60	2009	W
2930_1719_S2168	2014	D	21.05.2014	S2168	14.1	23	Fam-60_2009_	S2168	2168	5017	60	2009	W
2930_1719_S2169	2014	D	21.05.2014	S2169	15.8	32	Fam-60_2009_	S2169	2169	5018	60	2009	W
2930_1719_S2170	2014	D	21.05.2014	S2170	13.7	21.5	Fam-60_2009_	S2170	2170	5019	60	2009	W
2930_1719_S2171	2014	D	21.05.2014	S2171	14.1	23.5	Fam-54_2009_	S2171	2171	5020	54	2009	W
2930_1719_S2172	2014	D	21.05.2014	S2172	15.3	29	Fam-32_2009_	S2172	2172	5021	32	2009	H
2930_1719_S2173	2014	D	21.05.2014	S2173	13.6	21.5	Fam-54_2009_	S2173	2173	5022	54	2009	W
2930_1719_S2175	2014	D	21.05.2014	S2175	14.8	26	Fam-54_2009_	S2175	2175	5023	54	2009	W

2930_1719_S2176	2014	D	21.05.2014	S2176	14.6	24.5	Fam-60_2009_	S2176	2176	5024	60	2009	W
2930_1719_S2177	2014	D	21.05.2014	S2177	14.8	26.5	Fam-55_2009_	S2177	2177	5025	55	2009	W
2930_1719_S2178	2014	D	22.05.2014	S2178	12.8	17	Fam-54_2009_	S2178	2178	5026	54	2009	W
2930_1720_S2179	2014	D	22.05.2014	S2179	15	29.5	Fam-44_2009_	S2179	2179	5027	44	2009	H
2930_1720_S2180	2014	D	22.05.2014	S2180	15.4	28.5	Fam-55_2009_	S2180	2180	5028	55	2009	W
2930_1720_S2181	2014	D	22.05.2014	S2181	13.9	21	Fam-54_2009_	S2181	2181	5029	54	2009	W
2930_1720_S2182	2014	D	22.05.2014	S2182	14.4	25	Fam-45_2009_	S2182	2182	5030	45	2009	F
2930_1720_S2183	2014	D	22.05.2014	S2183	15.4	30	Fam-32_2009_	S2183	2183	5031	32	2009	H
2930_1720_S2184	2014	D	22.05.2014	S2184	14.5	24	Fam-57_2009_	S2184	2184	5032	57	2009	W
2930_1720_S2185	2014	D	22.05.2014	S2185	15	28.5	Fam-58_2009_	S2185	2185	5033	58	2009	W
2930_1720_S2186	2014	D	22.05.2014	S2186	14.6	26	Fam-54_2009_	S2186	2186	5034	54	2009	W
2930_1720_S2187	2014	D	22.05.2014	S2187	14.6	26	Fam-59_2009_	S2187	2187	5035	59	2009	W
2930_1720_S2188	2014	D	22.05.2014	S2188	15.7	30	Fam-59_2009_	S2188	2188	5036	59	2009	W
2930_1720_S2189	2014	D	22.05.2014	S2189	14.2	24	Fam-55_2009_	S2189	2189	5037	55	2009	W

2930_1720_S219(	2014	D	22.05.2014	S2190	15	29	Fam-34_2009_	S2190	2190	5038	34	2009	H
2930_1720_S2191	2014	D	22.05.2014	S2191	14.2	24	Fam-60_2009_	S2191	2191	5039	60	2009	W
2930_1720_S2192	2014	D	22.05.2014	S2192	16	32	Fam-52_2009_	S2192	2192	5040	52	2009	W
2930_1720_S219:	2014	D	22.05.2014	S2193	14.5	24.5	Fam-51_2009_	S2193	2193	5041	51	2009	W
2930_1720_S2194	2014	D	22.05.2014	S2194	15	27	Fam-74_2010_	S2194	2194	5042	74	2010	H
2930_1720_S219!	2014	D	22.05.2014	S2195	14.5	25	Fam-60_2009_	S2195	2195	5043	60	2009	W
2930_1720_S219€	2014	D	22.05.2014	S2196	16	33	Fam-54_2009_	S2196	2196	5044	54	2009	W
2930_1720_S2197	2014	D	22.05.2014	S2197	14	24	Fam-55_2009_	S2197	2197	5045	55	2009	W
2930_1720_S219{	2014	D	22.05.2014	S2198	11.9	14.5	Fam-59_2009_	S2198	2198	5046	59	2009	W
2930_1720_S219{	2014	D	22.05.2014	S2199	15.5	29	Fam-40_2009_	S2199	2199	5047	40	2009	H
2930_1720_S220(	2014	D	22.05.2014	S2200	14	19	Fam-59_2009_	S2200	2200	5048	59	2009	W
2930_1720_S2201	2014	D	22.05.2014	S2201	18	47	Fam-52_2009_	S2201	2201	5049	52	2009	W
2930_1720_S2202	2014	D	22.05.2014	S2202	13.8	24	Fam-42_2009_	S2202	2202	5050	42	2009	H
2930_1720_S220:	2014	D	22.05.2014	S2203	13.5	20	Fam-89_2010_	S2203	2203	5051	89	2010	W

2930_1720_S2204	2014	D	22.05.2014	S2204	15	28	Fam-41_2009_	S2204	2204	5052	41	2009	F
2930_1720_S2205	2014	D	22.05.2014	S2205	13.7	21	Fam-87_2010_	S2205	2205	5053	87	2010	W
2930_1720_S2206	2014	D	22.05.2014	S2206	16.3	34.5	Fam-43_2009_	S2206	2206	5054	43	2009	F
2930_1720_S2208	2014	D	22.05.2014	S2208	14	25	Fam-90_2010_	S2208	2208	5055	90	2010	W
2930_1720_S2209	2014	D	22.05.2014	S2209	15	28	Fam-57_2009_	S2209	2209	5056	57	2009	W
2930_1720_S2210	2014	D	22.05.2014	S2210	13.7	21	Fam-59_2009_	S2210	2210	5057	59	2009	W
2930_1720_S2211	2014	D	22.05.2014	S2211	14.8	26	Fam-51_2009_	S2211	2211	5058	51	2009	W
2930_1720_S2212	2014	D	22.05.2014	S2212	13.2	19.5	Fam-42_2009_	S2212	2212	5059	42	2009	H
2930_1720_S2213	2014	D	22.05.2014	S2213	14.2	23	Fam-54_2009_	S2213	2213	5060	54	2009	W
2930_1720_S2214	2014	D	22.05.2014	S2214	14.8	26	Fam-55_2009_	S2214	2214	5061	55	2009	W
2930_1720_S2215	2014	D	22.05.2014	S2215	15.3	28.5	Fam-55_2009_	S2215	2215	5062	55	2009	W
2930_1720_S2216	2014	D	22.05.2014	S2216	15.1	29	Fam-87_2010_	S2216	2216	5063	87	2010	W
2930_1720_S2217	2014	D	22.05.2014	S2217	13.1	19	Fam-85_2010_	S2217	2217	5064	85	2010	W
2930_1720_S2218	2014	D	22.05.2014	S2218	14.3	22.5	Fam-54_2009_	S2218	2218	5065	54	2009	W

2930_1720_S2219	2014	D	22.05.2014	S2219	14.7	28.5	Fam-85_2010_	S2219	2219	5066	85	2010	W
2930_1720_S2220	2014	D	22.05.2014	S2220	14.8	26	Fam-55_2009_	S2220	2220	5067	55	2009	W
2930_1720_S2221	2014	D	22.05.2014	S2221	14.1	23	Fam-54_2009_	S2221	2221	5068	54	2009	W
2930_1720_S2222	2014	D	22.05.2014	S2222	15.6	30	Fam-53_2009_	S2222	2222	5069	53	2009	W
2930_1720_S2223	2014	D	22.05.2014	S2223	15.7	22	Fam-55_2009_	S2223	2223	5070	55	2009	W
2930_1720_S2224	2014	D	22.05.2014	S2224	15.3	28	Fam-57_2009_	S2224	2224	5071	57	2009	W
2930_1720_S2225	2014	D	22.05.2014	S2225	13.7	20	Fam-58_2009_	S2225	2225	5072	58	2009	W
2930_1720_S2226	2014	D	22.05.2014	S2226	15.6	31	Fam-34_2009_	S2226	2226	5073	34	2009	H
2930_1720_S2227	2014	D	22.05.2014	S2227	12.6	16.5	Fam-59_2009_	S2227	2227	5074	59	2009	W
2930_1720_S2228	2014	D	22.05.2014	S2228	13.5	21	Fam-54_2009_	S2228	2228	5075	54	2009	W
2930_1720_S2229	2014	D	22.05.2014	S2229	15.3	26	Fam-40_2009_	S2229	2229	5076	40	2009	H
2930_1720_S2230	2014	D	22.05.2014	S2230	13.2	19	Fam-42_2009_	S2230	2230	5077	42	2009	H
2930_1720_S2231	2014	D	22.05.2014	S2231	13.8	20	Fam-89_2010_	S2231	2231	5078	89	2010	W
2930_1720_S2232	2014	D	22.05.2014	S2232	14.7	26	Fam-54_2009_	S2232	2232	5079	54	2009	W

2930_1720_S2233	2014	D	26.05.2014	S2233	14.7	23.5	Fam-89_2010_	S2233	2233	5080	89	2010	W
2930_1720_S2234	2014	D	26.05.2014	S2234	14.5	23.5	Fam-52_2009_	S2234	2234	5081	52	2009	W
2930_1720_S2235	2014	D	26.05.2014	S2235	15.9	30.5	Fam-34_2009_	S2235	2235	5082	34	2009	H
2930_1720_S2236	2014	D	26.05.2014	S2236	14.5	24.5	Fam-34_2009_	S2236	2236	5083	34	2009	H
2930_1720_S2237	2014	D	26.05.2014	S2237	13.8	21.5	Fam-54_2009_	S2237	2237	5084	54	2009	W
2930_1720_S2238	2014	D	26.05.2014	S2238	15.2	27.5	Fam-55_2009_	S2238	2238	5085	55	2009	W
2930_1720_S2239	2014	D	26.05.2014	S2239	16.4	36	Fam-51_2009_	S2239	2239	5086	51	2009	W
2931_1720_S2241	2014	D	26.05.2014	S2241	16.6	24.5	Fam-54_2009_	S2241	2241	5087	54	2009	W
2931_1720_S2242	2014	D	26.05.2014	S2242	15.6	31.5	Fam-55_2009_	S2242	2242	5088	55	2009	W
2931_1720_S2243	2014	D	26.05.2014	S2243	14	23.5	Fam-59_2009_	S2243	2243	5089	59	2009	W
2931_1720_S2244	2014	D	26.05.2014	S2244	13.1	19.5	Fam-42_2009_	S2244	2244	5090	42	2009	H
2931_1720_S2245	2014	D	26.05.2014	S2245	13.3	19.5	Fam-82_2010_	S2245	2245	5091	82	2010	W
2931_1720_S2246	2014	D	26.05.2014	S2246	14.4	26	Fam-54_2009_	S2246	2246	5092	54	2009	W
2931_1720_S2247	2014	D	26.05.2014	S2247	13.9	21	Fam-59_2009_	S2247	2247	5093	59	2009	W

2931_1720_S2248	2014	D	26.05.2014	S2248	13.3	18.5	Fam-55_2009_	S2248	2248	5094	55	2009	W
2931_1720_S2249	2014	D	26.05.2014	S2249	13.9	23.5	Fam-05_2007_	S2249	2249	5095	5	2007	W
2931_1720_S2250	2014	D	26.05.2014	S2250	15.3	28	Fam-57_2009_	S2250	2250	5096	57	2009	W
2931_1720_S2251	2014	D	26.05.2014	S2251	13.7	21.5	Fam-59_2009_	S2251	2251	5097	59	2009	W
2931_1720_S2252	2014	D	26.05.2014	S2252	13.9	22	Fam-54_2009_	S2252	2252	5098	54	2009	W
2931_1720_S2253	2014	D	26.05.2014	S2253	12.2	15	Fam-86_2010_	S2253	2253	5099	86	2010	W
2931_1720_S2254	2014	D	26.05.2014	S2254	14.7	27.5	Fam-60_2009_	S2254	2254	5100	60	2009	W
2931_1720_S2255	2014	D	26.05.2014	S2255	14.6	25	Fam-80_2010_	S2255	2255	5101	80	2010	H
2931_1720_S2256	2014	D	26.05.2014	S2256	13.9	22	Fam-42_2009_	S2256	2256	5102	42	2009	H
2931_1720_S2257	2014	D	26.05.2014	S2257	13.6	21.5	Fam-60_2009_	S2257	2257	5103	60	2009	W
2931_1720_S2259	2014	D	26.05.2014	S2259	12.6	15.5	Fam-55_2009_	S2259	2259	5104	55	2009	W
2931_1720_S2260	2014	D	26.05.2014	S2260	13.7	21.5	Fam-57_2009_	S2260	2260	5105	57	2009	W
2931_1720_S2261	2014	D	26.05.2014	S2261	13.9	23	Fam-54_2009_	S2261	2261	5106	54	2009	W
2931_1720_S2262	2014	D	26.05.2014	S2262	14	24.5	Fam-44_2009_	S2262	2262	5107	44	2009	H

2931_1720_S2263	2014	D	26.05.2014	S2263	15.3	28.5	Fam-60_2009_	S2263	2263	5108	60	2009	W
2931_1720_S2264	2014	D	26.05.2014	S2264	13.7	21.5	Fam-55_2009_	S2264	2264	5109	55	2009	W
2931_1720_S2265	2014	D	26.05.2014	S2265	14.8	27.5	Fam-60_2009_	S2265	2265	5110	60	2009	W
2931_1720_S2267	2014	D	28.05.2014	S2267	16.6	37.5	Fam-54_2009_	S2267	2267	5111	54	2009	W
2931_1720_S2269	2014	D	28.05.2014	S2269	14.5	27	Fam-42_2009_	S2269	2269	5112	42	2009	H
2931_1720_S2270	2014	D	28.05.2014	S2270	15.2	27	Fam-57_2009_	S2270	2270	5113	57	2009	W
2931_1720_S2271	2014	D	28.05.2014	S2271	14.5	23.5	Fam-59_2009_	S2271	2271	5114	59	2009	W
2931_1720_S2272	2014	D	28.05.2014	S2272	13.8	19	Fam-57_2009_	S2272	2272	5115	57	2009	W
2931_1721_S2272	2014	D	28.05.2014	S2272	13.8	19	Fam-57_2009_	S2272	2272	5116	57	2009	W
2931_1721_S2273	2014	D	29.05.2014	S2273	15.2	29	Fam-60_2009_	S2273	2273	5117	60	2009	W
2931_1721_S2275	2014	D	29.05.2014	S2275	15.3	27	Fam-40_2009_	S2275	2275	5118	40	2009	H
2931_1721_S2276	2014	D	31.05.2014	S2276	15.2	28	Fam-54_2009_	S2276	2276	5119	54	2009	W
2931_1721_S2277	2014	D	31.05.2014	S2277	14.8	27	Fam-54_2009_	S2277	2277	5120	54	2009	W
2931_1721_S2278	2014	D	31.05.2014	S2278	14	23.5	Fam-42_2009_	S2278	2278	5121	42	2009	H

2931_1721_S2279	2014	D	31.05.2014	S2279	15.2	26.5	Fam-55_2009_	S2279	2279	5122	55	2009	W
2931_1721_S2280	2014	D	31.05.2014	S2280	16.1	34.5	Fam-60_2009_	S2280	2280	5123	60	2009	W
2931_1721_S2281	2014	D	31.05.2014	S2281	16.2	31.5	Fam-55_2009_	S2281	2281	5124	55	2009	W
2931_1721_S2282	2014	D	31.05.2014	S2282	13.7	21.5	Fam-60_2009_	S2282	2282	5125	60	2009	W
2931_1721_S2283	2014	D	31.05.2014	S2283	15.6	31.5	Fam-40_2009_	S2283	2283	5126	40	2009	H
2931_1721_S2284	2014	D	31.05.2014	S2284	14.8	26	Fam-55_2009_	S2284	2284	5127	55	2009	W
2931_1721_S2285	2014	D	31.05.2014	S2285	15.2	29	Fam-60_2009_	S2285	2285	5128	60	2009	W
2931_1721_S2286	2014	D	31.05.2014	S2286	15.8	32	Fam-60_2009_	S2286	2286	5129	60	2009	W
2931_1721_S2287	2014	D	31.05.2014	S2287	14.4	24.5	Fam-87_2010_	S2287	2287	5130	87	2010	W
2931_1721_S2288	2014	D	31.05.2014	S2288	13.2	18.5	Fam-56_2009_	S2288	2288	5131	56	2009	W
2931_1721_S2289	2014	D	31.05.2014	S2289	13.5	19	Fam-59_2009_	S2289	2289	5132	59	2009	W
2931_1721_S2290	2014	D	31.05.2014	S2290	13.9	25	Fam-53_2009_	S2290	2290	5133	53	2009	W
2931_1721_S2291	2014	D	31.05.2014	S2291	14.6	25	Fam-59_2009_	S2291	2291	5134	59	2009	W
2931_1721_S2292	2014	D	31.05.2014	S2292	15.4	28.5	Fam-87_2010_	S2292	2292	5135	87	2010	W

2931_1721_S2293	2014	D	31.05.2014	S2293	14.6	24	Fam-59_2009_	S2293	2293	5136	59	2009	W
2931_1721_S2294	2014	D	31.05.2014	S2294	12.8	17.5	Fam-55_2009_	S2294	2294	5137	55	2009	W
2931_1721_S2295	2014	D	31.05.2014	S2295	13.5	21	Fam-87_2010_	S2295	2295	5138	87	2010	W
2931_1721_S2296	2014	D	31.05.2014	S2296	13	18.5	Fam-60_2009_	S2296	2296	5139	60	2009	W
2931_1721_S2297	2014	D	31.05.2014	S2297	14.8	25	Fam-55_2009_	S2297	2297	5140	55	2009	W
2931_1721_S2298	2014	D	31.05.2014	S2298	13.9	23	Fam-55_2009_	S2298	2298	5141	55	2009	W
2931_1721_S2299	2014	D	31.05.2014	S2299	14.1	25	Fam-90_2010_	S2299	2299	5142	90	2010	W
2931_1721_S2300	2014	D	31.05.2014	S2300	14	23	Fam-42_2009_	S2300	2300	5143	42	2009	H
2931_1721_S2301	2014	D	02.06.2014	S2301	14.3	24	Fam-57_2009_	S2301	2301	5144	57	2009	W
2931_1721_S2302	2014	D	02.06.2014	S2302	13	18	Fam-59_2009_	S2302	2302	5145	59	2009	W
2931_1721_S2303	2014	D	02.06.2014	S2303	13.9	23	Fam-42_2009_	S2303	2303	5146	42	2009	H
2931_1721_S2304	2014	D	02.06.2014	S2304	14.2	23	Fam-42_2009_	S2304	2304	5147	42	2009	H
2931_1721_S2305	2014	D	02.06.2014	S2305	14.8	25	Fam-55_2009_	S2305	2305	5148	55	2009	W
2931_1721_S2306	2014	D	02.06.2014	S2306	15.2	28	Fam-56_2009_	S2306	2306	5149	56	2009	W

2931_1721_S2308	2014	D	02.06.2014	S2308	13.5	19	Fam-60_2009_ S2308	2308	5150	60	2009	W
2931_1721_S2309	2014	D	02.06.2014	S2309	12.4	16.5	Fam-87_2010_ S2309	2309	5151	87	2010	W
2931_1721_S2310	2014	D	02.06.2014	S2310	13.8	20.5	Fam-53_2009_ S2310	2310	5152	53	2009	W
2931_1721_S2311	2014	D	02.06.2014	S2311	13.9	21.5	Fam-32_2009_ S2311	2311	5153	32	2009	H
2931_1721_S2312	2014	D	02.06.2014	S2312	14.6	26	Fam-60_2009_ S2312	2312	5154	60	2009	W
2931_1721_S2313	2014	D	02.06.2014	S2313	14.5	26.5	Fam-56_2009_ S2313	2313	5155	56	2009	W
2931_1721_S2314	2014	D	02.06.2014	S2314	15.6	30	Fam-59_2009_ S2314	2314	5156	59	2009	W
2931_1721_S2315	2014	D	02.06.2014	S2315	14.8	25	Fam-32_2009_ S2315	2315	5157	32	2009	H
2931_1721_S2316	2014	D	02.06.2014	S2316	13.7	21	Fam-51_2009_ S2316	2316	5158	51	2009	W
2931_1721_S2317	2014	D	02.06.2014	S2317	15	27	Fam-54_2009_ S2317	2317	5159	54	2009	W
2931_1721_S2318	2014	D	02.06.2014	S2318	15.5	29	Fam-54_2009_ S2318	2318	5160	54	2009	W
2931_1721_S2319	2014	D	02.06.2014	S2319	15.4	30	Fam-60_2009_ S2319	2319	5161	60	2009	W
2931_1721_S2320	2014	D	02.06.2014	S2320	13.3	19	Fam-60_2009_ S2320	2320	5162	60	2009	W
2938_1731_S2321	2014	D	02.06.2014	S2321	14.3	24	Fam-44_2009_ S2321	2321	5163	44	2009	H

2938_1731_S2322	2014	D	02.06.2014	S2322	13.2	18.5	Fam-57_2009_S2322	2322	5164	57	2009	W
2938_1731_S2323	2014	D	02.06.2014	S2323	14.1	22	Fam-60_2009_S2323	2323	5165	60	2009	W
2938_1731_S2324	2014	D	02.06.2014	S2324	12.8	18	Fam-59_2009_S2324	2324	5166	59	2009	W
2938_1731_S2325	2014	D	02.06.2014	S2325	13.8	21	Fam-59_2009_S2325	2325	5167	59	2009	W
2938_1731_S2326	2014	D	06.06.2014	S2326	15.1	27	Fam-54_2009_S2326	2326	5168	54	2009	W
2938_1731_S2327	2014	D	06.06.2014	S2327	14.8	24.5	Fam-60_2009_S2327	2327	5169	60	2009	W
2938_1731_S2328	2014	D	06.06.2014	S2328	14.8	24	Fam-57_2009_S2328	2328	5170	57	2009	W
2938_1731_S2331	2014	D	06.06.2014	S2331	15.4	26.5	Fam-53_2009_S2331	2331	5171	53	2009	W
2938_1731_S2332	2014	D	06.06.2014	S2332	14.5	23	Fam-70_2010_S2332	2332	5172	70	2010	H
2938_1731_S2333	2014	D	06.06.2014	S2333	12.7	16	Fam-76_2010_S2333	2333	5173	76	2010	H
2938_1731_S2334	2014	D	06.06.2014	S2334	14	21.5	Fam-54_2009_S2334	2334	5174	54	2009	W
2938_1731_S2335	2014	D	06.06.2014	S2335	13.5	19.5	Fam-54_2009_S2335	2335	5175	54	2009	W
2938_1731_S2336	2014	D	06.06.2014	S2336	15	25	Fam-53_2009_S2336	2336	5176	53	2009	W
2938_1731_S2337	2014	D	10.06.2014	S2337	14.1	21	Fam-53_2009_S2337	2337	5177	53	2009	W

2938_1731_S2338	2014	D	10.06.2014	S2338	15.8	29.5	Fam-44_2009_	S2338	2338	5178	44	2009 H
2938_1731_S2339	2014	D	10.06.2014	S2339	14.5	23	Fam-40_2009_	S2339	2339	5179	40	2009 H
2938_1731_S2340	2014	D	10.06.2014	S2340	14.1	21.5	Fam-53_2009_	S2340	2340	5180	53	2009 W
2938_1731_S2341	2014	D	10.06.2014	S2341	15.6	29	Fam-54_2009_	S2341	2341	5181	54	2009 W
2938_1731_S2342	2014	D	10.06.2014	S2342	14.1	22	Fam-56_2009_	S2342	2342	5182	56	2009 W
2938_1731_S2343	2014	D	10.06.2014	S2343	14.2	23.5	Fam-56_2009_	S2343	2343	5183	56	2009 W
2938_1731_S2344	2014	D	10.06.2014	S2344	13.7	20.5	Fam-53_2009_	S2344	2344	5184	53	2009 W
2938_1731_S2345	2014	D	13.06.2014	S2345	15.1	27	Fam-60_2009_	S2345	2345	5185	60	2009 W
2938_1731_S2346	2014	D	13.06.2014	S2346	15.6	27	Fam-34_2009_	S2346	2346	5186	34	2009 H
2938_1731_S2347	2014	D	13.06.2014	S2347	14.2	23	Fam-42_2009_	S2347	2347	5187	42	2009 H
2938_1731_S2348	2014	D	13.06.2014	S2348	15.3	28	Fam-55_2009_	S2348	2348	5188	55	2009 W
2938_1731_S2350	2014	D	13.06.2014	S2350	14.6	22	Fam-53_2009_	S2350	2350	5189	53	2009 W
2938_1731_S2351	2014	D	13.06.2014	S2351	16	31	Fam-60_2009_	S2351	2351	5190	60	2009 W
2938_1731_S2353	2014	D	13.06.2014	S2353	14.7	23.5	Fam-55_2009_	S2353	2353	5191	55	2009 W

2938_1731_S2354	2014	D	13.06.2014	S2354	14	21.5	Fam-60_2009_	S2354	2354	5192	60	2009	W
2938_1731_S2355	2014	D	13.06.2014	S2355	15.5	28	Fam-57_2009_	S2355	2355	5193	57	2009	W
2938_1731_S2356	2014	D	13.06.2014	S2356	15.6	29	Fam-81_2010_	S2356	2356	5194	81	2010	W
2938_1731_S2357	2014	D	13.06.2014	S2357	14.1	22.5	Fam-56_2009_	S2357	2357	5195	56	2009	W
2938_1731_S2358	2014	D	13.06.2014	S2358	16.1	32.5	Fam-60_2009_	S2358	2358	5196	60	2009	W
2938_1731_S2359	2014	D	13.06.2014	S2359	15.2	26	Fam-60_2009_	S2359	2359	5197	60	2009	W
2938_1731_S2360	2014	D	13.06.2014	S2360	15.6	29	Fam-52_2009_	S2360	2360	5198	52	2009	W
2938_1731_S2361	2014	D	13.06.2014	S2361	15.9	32	Fam-60_2009_	S2361	2361	5199	60	2009	W
2938_1731_S2362	2014	D	13.06.2014	S2362	15.1	26	Fam-60_2009_	S2362	2362	5200	60	2009	W
2938_1731_S2363	2014	D	13.06.2014	S2363	14.5	23	Fam-60_2009_	S2363	2363	5201	60	2009	W
2938_1731_S2364	2014	D	13.06.2014	S2364	15.6	30	Fam-60_2009_	S2364	2364	5202	60	2009	W
2938_1731_S2365	2014	D	13.06.2014	S2365	15	26.5	Fam-52_2009_	S2365	2365	5203	52	2009	W
2938_1731_S2366	2014	D	13.06.2014	S2366	14.4	23	Fam-60_2009_	S2366	2366	5204	60	2009	W
2938_1731_S2367	2014	D	13.06.2014	S2367	14.5	24	Fam-55_2009_	S2367	2367	5205	55	2009	W

2938_1731_S2368	2014	D	13.06.2014	S2368	16.5	33.5	Fam-53_2009_ S2368	2368	5206	53	2009	W
2938_1731_S2369	2014	D	13.06.2014	S2369	14	23.5	Fam-90_2010_ S2369	2369	5207	90	2010	W
2938_1731_S2370	2014	D	13.06.2014	S2370	14.5	22.5	Fam-60_2009_ S2370	2370	5208	60	2009	W
2938_1731_S2371	2014	D	13.06.2014	S2371	14.6	23	Fam-54_2009_ S2371	2371	5209	54	2009	W
2938_1731_S2372	2014	D	13.06.2014	S2372	14.8	25	Fam-56_2009_ S2372	2372	5210	56	2009	W
2938_1731_S2373	2014	D	13.06.2014	S2373	14.5	22	Fam-57_2009_ S2373	2373	5211	57	2009	W
2938_1731_S2374	2014	D	13.06.2014	S2374	14.3	22	Fam-53_2009_ S2374	2374	5212	53	2009	W
2938_1731_S2375	2014	D	13.06.2014	S2375	15.6	30	Fam-60_2009_ S2375	2375	5213	60	2009	W
2938_1731_S2376	2014	D	13.06.2014	S2376	14.5	23	Fam-54_2009_ S2376	2376	5214	54	2009	W
2938_1731_S2377	2014	D	13.06.2014	S2377	15.9	31	Fam-34_2009_ S2377	2377	5215	34	2009	H
2938_1731_S2378	2014	D	13.06.2014	S2378	14.7	24	Fam-55_2009_ S2378	2378	5216	55	2009	W
2938_1731_S2379	2014	D	13.06.2014	S2379	14.2	22	Fam-60_2009_ S2379	2379	5217	60	2009	W
2938_1731_S2380	2014	D	13.06.2014	S2380	13.3	17	Fam-55_2009_ S2380	2380	5218	55	2009	W
2938_1731_S2381	2014	D	13.06.2014	S2381	14.2	23	Fam-60_2009_ S2381	2381	5219	60	2009	W

2938_1731_S2383	2014	D	13.06.2014	S2383	15	25	Fam-55_2009_	S2383	2383	5220	55	2009	W
2938_1731_S2384	2014	D	13.06.2014	S2384	14.5	25.5	Fam-60_2009_	S2384	2384	5221	60	2009	W
2938_1731_S2386	2014	D	13.06.2014	S2386	14.7	23.5	Fam-55_2009_	S2386	2386	5222	55	2009	W
2938_1731_S2387	2014	D	15.06.2014	S2387	13.9	20.5	Fam-34_2009_	S2387	2387	5223	34	2009	H
2938_1731_S2388	2014	D	15.06.2014	S2388	14.9	25.5	Fam-60_2009_	S2388	2388	5224	60	2009	W
2938_1731_S2390	2014	D	15.06.2014	S2390	14.5	22	Fam-60_2009_	S2390	2390	5225	60	2009	W
2938_1731_S2391	2014	D	15.06.2014	S2391	14.8	25	Fam-32_2009_	S2391	2391	5226	32	2009	H
2938_1731_S2392	2014	D	15.06.2014	S2392	14.2	20	Fam-53_2009_	S2392	2392	5227	53	2009	W
2938_1731_S2393	2014	D	15.06.2014	S2393	15.2	26	Fam-60_2009_	S2393	2393	5228	60	2009	W
2938_1731_S2394	2014	D	18.06.2014	S2394	14.8	23	Fam-57_2009_	S2394	2394	5229	57	2009	W
2938_1731_S2395	2014	D	18.06.2014	S2395	15.5	27.5	Fam-60_2009_	S2395	2395	5230	60	2009	W
2938_1731_S2396	2014	D	18.06.2014	S2396	15.2	26	Fam-48_2009_	S2396	2396	5231	48	2009	H
2938_1731_S2398	2014	D	18.06.2014	S2398	13.3	16.5	Fam-55_2009_	S2398	2398	5232	55	2009	W
2938_1731_S2399	2014	D	18.06.2014	S2399	14.5	22.5	Fam-60_2009_	S2399	2399	5233	60	2009	W

2938_1731_S240[illegible]	2014	D	18.06.2014	S2400	13.5	16.5	Fam-59_2009_S2400	2400	5234	59	2009	W
2939_1731_S240[illegible]	2014	D	18.06.2014	S2401	14.4	22	Fam-59_2009_S2401	2401	5235	59	2009	W
2939_1731_S240[illegible]	2014	D	18.06.2014	S2402	15.1	27	Fam-60_2009_S2402	2402	5236	60	2009	W
2939_1731_S240[illegible]	2014	D	18.06.2014	S2403	14.8	21	Fam-60_2009_S2403	2403	5237	60	2009	W
2939_1731_S240[illegible]	2014	D	20.06.2014	S2404	15.8	28	Fam-60_2009_S2404	2404	5238	60	2009	W
2939_1731_S240[illegible]	2014	D	20.06.2014	S2405	14.9	24.5	Fam-60_2009_S2405	2405	5239	60	2009	W
2939_1731_S240[illegible]	2014	D	20.06.2014	S2406	14	19.5	Fam-55_2009_S2406	2406	5240	55	2009	W
2939_1731_S240[illegible]	2014	D	20.06.2014	S2407	15.2	26	Fam-60_2009_S2407	2407	5241	60	2009	W
2939_1731_S240[illegible]	2014	D	20.06.2014	S2408	14.5	23.5	Fam-60_2009_S2408	2408	5242	60	2009	W
2939_1731_S240[illegible]	2014	D	20.06.2014	S2409	14.2	20.5	Fam-55_2009_S2409	2409	5243	55	2009	W
2939_1731_S241[illegible]	2014	D	20.06.2014	S2410	15.9	28	Fam-60_2009_S2410	2410	5244	60	2009	W
2939_1731_S241[illegible]	2014	D	20.06.2014	S2411	14.7	22	Fam-55_2009_S2411	2411	5245	55	2009	W
2939_1731_S241[illegible]	2014	D	20.06.2014	S2412	16.2	31.5	Fam-55_2009_S2412	2412	5246	55	2009	W
2939_1731_S241[illegible]	2014	D	20.06.2014	S2413	14.5	21.5	Fam-55_2009_S2413	2413	5247	55	2009	W

2939_1731_S2414	2014	D	20.06.2014	S2414	14.6	22.5	Fam-55_2009_	S2414	2414	5248	55	2009	W
2939_1732_S2415	2014	D	20.06.2014	S2415	15.7	27	Fam-60_2009_	S2415	2415	5249	60	2009	W
2939_1732_S2416	2014	D	20.06.2014	S2416	15.5	28	Fam-54_2009_	S2416	2416	5250	54	2009	W
2939_1732_S2417	2014	D	20.06.2014	S2417	13.3	17	Fam-54_2009_	S2417	2417	5251	54	2009	W
2939_1732_S2418	2014	D	20.06.2014	S2418	14.5	21	Fam-55_2009_	S2418	2418	5252	55	2009	W
2939_1732_S2419	2014	D	20.06.2014	S2419	13.4	17	Fam-55_2009_	S2419	2419	5253	55	2009	W
2939_1732_S2420	2014	D	20.06.2014	S2420	16.3	29.5	Fam-55_2009_	S2420	2420	5254	55	2009	W
2939_1732_S2421	2014	D	20.06.2014	S2421	14.8	23	Fam-54_2009_	S2421	2421	5255	54	2009	W
2939_1732_S2422	2014	D	20.06.2014	S2422	13.9	24.5	Fam-55_2009_	S2422	2422	5256	55	2009	W
2939_1732_S2423	2014	D	20.06.2014	S2423	16.4	29	Fam-60_2009_	S2423	2423	5257	60	2009	W
2939_1732_S2424	2014	D	20.06.2014	S2424	15.7	27	Fam-44_2009_	S2424	2424	5258	44	2009	H
2939_1732_S2425	2014	D	20.06.2014	S2425	16	30.5	Fam-55_2009_	S2425	2425	5259	55	2009	W
2939_1732_S2426	2014	D	20.06.2014	S2426	14	20.5	Fam-51_2009_	S2426	2426	5260	51	2009	W
2939_1732_S2427	2014	D	20.06.2014	S2427	15	23.5	Fam-60_2009_	S2427	2427	5261	60	2009	W

2939_1732_S2428	2014	D	20.06.2014	S2428	15	24	Fam-55_2009_	S2428	2428	5262	55	2009	W
2939_1732_S2429	2014	D	20.06.2014	S2429	14.7	23	Fam-55_2009_	S2429	2429	5263	55	2009	W
2939_1732_S2431	2014	D	20.06.2014	S2431	13.9	21	Fam-76_2010_	S2431	2431	5264	76	2010	H
2939_1732_S2432	2014	D	20.06.2014	S2432	14.1	21	Fam-55_2009_	S2432	2432	5265	55	2009	W
2939_1732_S2433	2014	D	20.06.2014	S2433	14.7	23.5	Fam-54_2009_	S2433	2433	5266	54	2009	W
2939_1732_S2434	2014	D	20.06.2014	S2434	14.7	24	Fam-68_2010_	S2434	2434	5267	68	2010	H
2939_1732_S2435	2014	D	20.06.2014	S2435	16.5	34.5	Fam-60_2009_	S2435	2435	5268	60	2009	W
2939_1732_S2436	2014	D	20.06.2014	S2436	15.9	31	Fam-55_2009_	S2436	2436	5269	55	2009	W
2939_1732_S2437	2014	D	20.06.2014	S2437	16.5	33.5	Fam-55_2009_	S2437	2437	5270	55	2009	W
2939_1732_S2438	2014	D	20.06.2014	S2438	13.7	20.5	Fam-54_2009_	S2438	2438	5271	54	2009	W
2939_1732_S2439	2014	D	20.06.2014	S2439	14.5	23.5	Fam-53_2009_	S2439	2439	5272	53	2009	W
2939_1732_S2440	2014	D	20.06.2014	S2440	17.6	40.5	Fam-55_2009_	S2440	2440	5273	55	2009	W
2939_1732_S2441	2014	D	20.06.2014	S2441	16	29.5	Fam-82_2010_	S2441	2441	5274	82	2010	W
2939_1732_S2442	2014	D	20.06.2014	S2442	14.3	23.5	Fam-55_2009_	S2442	2442	5275	55	2009	W

2939_1732_S2443	2014	D	20.06.2014	S2443	15.4	27.5	Fam-55_2009_	S2443	2443	5276	55	2009	W
2939_1732_S2444	2014	D	20.06.2014	S2444	15	27	Fam-60_2009_	S2444	2444	5277	60	2009	W
2939_1732_S2445	2014	D	20.06.2014	S2445	16.3	34.5	Fam-54_2009_	S2445	2445	5278	54	2009	W
2939_1732_S2446	2014	D	20.06.2014	S2446	16.7	34.5	Fam-54_2009_	S2446	2446	5279	54	2009	W
2939_1732_S2447	2014	D	20.06.2014	S2447	13.8	19.5	Fam-59_2009_	S2447	2447	5280	59	2009	W
2939_1732_S2448	2014	D	20.06.2014	S2448	17.5	41.5	Fam-55_2009_	S2448	2448	5281	55	2009	W
2939_1732_S2449	2014	D	20.06.2014	S2449	15.5	27.5	Fam-55_2009_	S2449	2449	5282	55	2009	W
2939_1732_S2450	2014	D	20.06.2014	S2450	14.3	23	Fam-55_2009_	S2450	2450	5283	55	2009	W
2939_1732_S2451	2014	D	23.06.2014	S2451	14	19	Fam-59_2009_	S2451	2451	5284	59	2009	W
2939_1732_S2452	2014	D	23.06.2014	S2452	17.2	36	Fam-55_2009_	S2452	2452	5285	55	2009	W
2939_1732_S2453	2014	D	23.06.2014	S2453	14.8	23.5	Fam-55_2009_	S2453	2453	5286	55	2009	W
2939_1732_S2455	2014	D	23.06.2014	S2455	16	27.5	Fam-60_2009_	S2455	2455	5287	60	2009	W
2939_1732_S2456	2014	D	23.06.2014	S2456	15.5	25	Fam-34_2009_	S2456	2456	5288	34	2009	H
2939_1732_S2457	2014	D	23.06.2014	S2457	15.5	24.5	Fam-55_2009_	S2457	2457	5289	55	2009	W

2939_1732_S2458	2014	D	23.06.2014	S2458	16.8	37	Fam-07_2007_	S2458	2458	5290	7	2007	W
2939_1732_S2459	2014	D	23.06.2014	S2459	14.5	22	Fam-59_2009_	S2459	2459	5291	59	2009	W
2939_1732_S2460	2014	D	23.06.2014	S2460	15.2	24	Fam-53_2009_	S2460	2460	5292	53	2009	W
3325_1993_S2_B0	2015	D	22.04.2015	S2	13.6	20	Fam-61_1_201	S2	2	5293	61	2010	F
3325_1993_S3_C0	2015	D	22.04.2015	S3	13.2	16.5	Fam-82_2010_	S3	3	5294	82	2010	W
3325_1993_S6_F0	2015	D	22.04.2015	S6	14.4	24	Fam-79_2010_	S6	6	5295	79	2010	F
3325_1993_S15_H	2015	D	22.04.2015	S15	12.5	14	Fam-64_2010_	S15	15	5296	64	2010	H
3325_1993_S16_A	2015	D	22.04.2015	S16	13.4	18.5	Fam-89_2010_	S16	16	5297	89	2010	W
3325_1993_S17_E	2015	D	22.04.2015	S17	12.8	17	Fam-85_2010_	S17	17	5298	85	2010	W
3325_1993_S18_C	2015	D	22.04.2015	S18	13.1	18	Fam-45_2009_	S18	18	5299	45	2009	F
3325_1993_S20_E	2015	D	22.04.2015	S20	13.3	18	Fam-63_2010_	S20	20	5300	63	2010	F
3325_1993_S22_G	2015	D	22.04.2015	S22	13.2	16.5	Fam-89_2010_	S22	22	5301	89	2010	W
3325_1993_S26_C	2015	D	24.04.2015	S26	15.7	30	Fam-61_1_201	S26	26	5302	61	2010	F
3325_1993_S28_E	2015	D	24.04.2015	S28	13.6	20.5	Fam-90_2010_	S28	28	5303	90	2010	W

3325_1993_S29_F	2015	D	24.04.2015	S29	14.6	23	Fam-66_2010_	S29	29	5304	66	2010	H
3325_1993_S30_G	2015	D	24.04.2015	S30	13.3	16.5	Fam-67_2010_	S30	30	5305	67	2010	F
3325_1993_S32_A	2015	D	24.04.2015	S32	13.8	19.5	Fam-89_2010_	S32	32	5306	89	2010	W
3325_1993_S35_D	2015	D	24.04.2015	S35	13.1	17	Fam-78_2010_	S35	35	5307	78	2010	H
3325_1993_S36_E	2015	D	24.04.2015	S36	14.4	22	Fam-73_2010_	S36	36	5308	73	2010	F
3325_1993_S37_F	2015	D	24.04.2015	S37	13.3	19	Fam-65_2010_	S37	37	5309	65	2010	F
3325_1993_S39_H	2015	D	24.04.2015	S39	12	15	Fam-61_1_201	S39	39	5310	61	2010	F
3325_1993_S40_A	2015	D	24.04.2015	S40	13.6	20	Fam-66_2010_	S40	40	5311	66	2010	H
3325_1993_S43_D	2015	D	24.04.2015	S43	12.5	17.5	Fam-72_2010_	S43	43	5312	72	2010	H
3325_1993_S44_E	2015	D	24.04.2015	S44	13.4	17	Fam-66_2010_	S44	44	5313	66	2010	H
3325_1993_S45_F	2015	D	24.04.2015	S45	16.1	32.5	Fam-35_2009_	S45	45	5314	35	2009	F
3325_1993_S48_A	2015	D	24.04.2015	S48	13.7	20	Fam-89_2010_	S48	48	5315	89	2010	W
3325_1993_S50_C	2015	D	24.04.2015	S50	12.6	17	Fam-77_2010_	S50	50	5316	77	2010	F
3325_1993_S51_D	2015	D	24.04.2015	S51	14.4	23.5	Fam-63_2010_	S51	51	5317	63	2010	F

3325_1993_S53_F	2015	D	24.04.2015	S53	12.4	15	Fam-85_2010_	S53	53	5318	85	2010	W
3325_1993_S54_G	2015	D	24.04.2015	S54	13.7	19.5	Fam-83_2010_	S54	54	5319	83	2010	W
3325_1993_S57_E	2015	T	24.04.2015	S57	13.3	18	Family-50_200	S57	57	5320	50	2009	H
3325_1993_S58_C	2015	D	24.04.2015	S58	14.1	21.5	Fam-71_2010_	S58	58	5321	71	2010	F
3325_1993_S59_D	2015	D	24.04.2015	S59	14.4	23.5	Fam-71_2010_	S59	59	5322	71	2010	F
3325_1993_S62_G	2015	D	24.04.2015	S62	14.2	22	Fam-71_2010_	S62	62	5323	71	2010	F
3325_1993_S63_H	2015	D	24.04.2015	S63	12.6	16.5	Fam-61_1_201	S63	63	5324	61	2010	F
3325_1993_S64_A	2015	D	24.04.2015	S64	13.9	25	Fam-80_2010_	S64	64	5325	80	2010	H
3325_1993_S66_C	2015	D	24.04.2015	S66	13.6	19	Fam-64_2010_	S66	66	5326	64	2010	H
3325_1993_S70_H	2015	D	24.04.2015	S70	12.8	18	Fam-79_2010_	S70	70	5327	79	2010	F
3325_1993_S72_E	2015	D	24.04.2015	S72	13.9	19	Fam-82_2010_	S72	72	5328	82	2010	W
3325_1993_S73_C	2015	D	27.04.2015	S73	15.6	33	Fam-31_2009_	S73	73	5329	31	2009	F
3325_1993_S74_D	2015	D	27.04.2015	S74	15.2	28	Fam-65_2010_	S74	74	5330	65	2010	F
3325_1993_S76_F	2015	D	27.04.2015	S76	14.8	26.5	Fam-65_2010_	S76	76	5331	65	2010	F

3325_1993_S77_G	2015	D	27.04.2015	S77	13.9	21.5	Fam-77_2010_	S77	77	5332	77	2010	F
3325_1993_S78_H	2015	D	27.04.2015	S78	13.5	19.5	Fam-67_2010_	S78	78	5333	67	2010	F
3325_1993_S80_E	2015	D	27.04.2015	S80	13.6	19	Fam-83_2010_	S80	80	5334	83	2010	W
3326_1993_S82_D	2015	D	27.04.2015	S82	13.9	21.5	Fam-76_2010_	S82	82	5335	76	2010	H
3326_1993_S84_F	2015	D	27.04.2015	S84	13.1	18	Fam-64_2010_	S84	84	5336	64	2010	H
3326_1993_S85_G	2015	D	27.04.2015	S85	14.1	23.5	Fam-71_2010_	S85	85	5337	71	2010	F
3326_1993_S86_H	2015	D	27.04.2015	S86	13.2	17.5	Fam-76_2010_	S86	86	5338	76	2010	H
3326_1993_S89_C	2015	D	27.04.2015	S89	14.4	24.5	Fam-66_2010_	S89	89	5339	66	2010	H
3326_1993_S92_F	2015	D	27.04.2015	S92	12.8	14.5	Fam-82_2010_	S92	92	5340	82	2010	W
3326_1994_S95_A	2015	D	27.04.2015	S95	11.9	14.5	Fam-63_2010_	S95	95	5341	63	2010	F
3326_1994_S97_C	2015	D	27.04.2015	S97	13.5	18.5	Fam-64_2010_	S97	97	5342	64	2010	H
3326_1994_S98_D	2015	D	27.04.2015	S98	13.9	19.5	Fam-67_2010_	S98	98	5343	67	2010	F
3326_1994_S99_E	2015	D	27.04.2015	S99	12.9	17	Fam-66_2010_	S99	99	5344	66	2010	H
3326_1994_S100_	2015	D	27.04.2015	S100	13.4	19.5	Fam-81_2010_	S100	100	5345	81	2010	W

3326_1994_S102_	2015	D	27.04.2015	S102	13.1	19.5	Fam-40_2009_	S102	102	5346	40	2009 H
3326_1994_S105_	2015	D	27.04.2015	S105	14.4	23.5	Fam-63_2010_	S105	105	5347	63	2010 F
3326_1994_S106_	2015	D	27.04.2015	S106	14.1	22.5	Fam-87_2010_	S106	106	5348	87	2010 W
3326_1994_S107_	2015	D	27.04.2015	S107	12.5	16.5	Fam-85_2010_	S107	107	5349	85	2010 W
3326_1994_S110_	2015	D	27.04.2015	S110	14.9	26	Fam-74_2010_	S110	110	5350	74	2010 H
3326_1994_S112_	2015	D	27.04.2015	S112	14.4	23.5	Fam-65_2010_	S112	112	5351	65	2010 F
3326_1994_S114_	2015	D	27.04.2015	S114	13	19	Fam-76_2010_	S114	114	5352	76	2010 H
3326_1994_S115_	2015	D	27.04.2015	S115	13.5	18.5	Fam-89_2010_	S115	115	5353	89	2010 W
3326_1994_S116_	2015	D	27.04.2015	S116	12.9	17.5	Fam-63_2010_	S116	116	5354	63	2010 F
3326_1994_S117_	2015	D	27.04.2015	S117	12.8	16.5	Fam-64_2010_	S117	117	5355	64	2010 H
3326_1994_S120_	2015	D	27.04.2015	S120	12.4	15	Fam-78_2010_	S120	120	5356	78	2010 H
3326_1994_S125_	2015	D	27.04.2015	S125	14	20	Fam-65_2010_	S125	125	5357	65	2010 F
3326_1994_S126_	2015	D	27.04.2015	S126	13.4	18.5	Fam-84_2010_	S126	126	5358	84	2010 W
3326_1994_S127_	2015	D	27.04.2015	S127	13.3	18	Fam-62_1_201	S127	127	5359	62	2010 H

3326_1994_S129_	2015	D	27.04.2015	S129	13.7	22.5	Fam-63_2010_	S129	129	5360	63	2010	F
3326_1994_S130_	2015	D	27.04.2015	S130	14.8	27.5	Fam-65_2010_	S130	130	5361	65	2010	F
3326_1994_S132_	2015	D	27.04.2015	S132	14.5	29	Fam-62_1_201	S132	132	5362	62	2010	H
3326_1994_S133_	2015	D	27.04.2015	S133	14.7	27.5	Fam-79_2010_	S133	133	5363	79	2010	F
3326_1994_S134_	2015	D	27.04.2015	S134	15.3	29.5	Fam-79_2010_	S134	134	5364	79	2010	F
3326_1994_S135_	2015	D	27.04.2015	S135	14.6	26	Fam-71_2010_	S135	135	5365	71	2010	F
3326_1994_S137_	2015	D	27.04.2015	S137	12.5	16	Fam-85_2010_	S137	137	5366	85	2010	W
3326_1994_S138_	2015	D	27.04.2015	S138	14.2	22.5	Fam-63_2010_	S138	138	5367	63	2010	F
3326_1994_S140_	2015	D	27.04.2015	S140	13.5	20.5	Fam-61_1_201	S140	140	5368	61	2010	F
3326_1994_S142_	2015	D	27.04.2015	S142	12.7	16.5	Fam-80_2010_	S142	142	5369	80	2010	H
3326_1994_S143_	2015	D	27.04.2015	S143	12.3	15	Fam-89_2010_	S143	143	5370	89	2010	W
3326_1994_S144_	2015	D	27.04.2015	S144	15.1	28.5	Fam-69_2010_	S144	144	5371	69	2010	F
3326_1994_S145_	2015	D	27.04.2015	S145	14.3	23.5	Fam-65_2010_	S145	145	5372	65	2010	F
3326_1994_S146_	2015	D	27.04.2015	S146	13.8	20.5	Fam-78_2010_	S146	146	5373	78	2010	H

3326_1994_S147_	2015	D	27.04.2015	S147	12.2	15.5	Fam-85_2010_S147	147	5374	85	2010 W
3326_1994_S148_	2015	D	27.04.2015	S148	14.5	24	Fam-72_2010_S148	148	5375	72	2010 H
3326_1994_S149_	2015	D	27.04.2015	S149	14.9	27.5	Fam-72_2010_S149	149	5376	72	2010 H
3326_1994_S151_	2015	D	27.04.2015	S151	13.5	18.5	Fam-69_2010_S151	151	5377	69	2010 F
3326_1994_S152_	2015	D	27.04.2015	S152	12.8	16	Fam-84_2010_S152	152	5378	84	2010 W
3326_1994_S153_	2015	D	27.04.2015	S153	14.8	27.5	Fam-80_2010_S153	153	5379	80	2010 H
3326_1994_S154_	2015	D	27.04.2015	S154	14.5	24	Fam-73_2010_S154	154	5380	73	2010 F
3326_1994_S158_	2015	D	27.04.2015	S158	14.3	24.5	Fam-66_2010_S158	158	5381	66	2010 H
3326_1994_S159_	2015	D	27.04.2015	S159	14.9	26.5	Fam-79_2010_S159	159	5382	79	2010 F
3327_1994_S161_	2015	D	27.04.2015	S161	15	27.5	Fam-77_2010_S161	161	5383	77	2010 F
3327_1994_S163_	2015	D	27.04.2015	S163	13	19	Fam-66_2010_S163	163	5384	66	2010 H
3327_1994_S167_	2015	D	27.04.2015	S167	13	17	Fam-67_2010_S167	167	5385	67	2010 F
3327_1994_S168_	2015	D	27.04.2015	S168	14.1	22.5	Fam-66_2010_S168	168	5386	66	2010 H
3327_1994_S169_	2015	D	27.04.2015	S169	12.8	16.5	Fam-72_2010_S169	169	5387	72	2010 H

3327_1994_S170_	2015	D	27.04.2015	S170	12.4	16	Fam-78_2010_	S170	170	5388	78	2010	H
3327_1994_S173_	2015	D	27.04.2015	S173	12	13.5	Fam-62_1_201	S173	173	5389	62	2010	H
3327_1994_S175_	2015	D	27.04.2015	S175	13.6	20.5	Fam-89_2010_	S175	175	5390	89	2010	W
3327_1994_S176_	2015	D	27.04.2015	S176	12.9	16.5	Fam-85_2010_	S176	176	5391	85	2010	W
3327_1994_S178_	2015	D	27.04.2015	S178	13.6	19.5	Fam-89_2010_	S178	178	5392	89	2010	W
3327_1994_S180_	2015	D	27.04.2015	S180	14.1	24	Fam-65_2010_	S180	180	5393	65	2010	F
3327_1994_S183_	2015	D	27.04.2015	S183	14.5	24	Fam-65_2010_	S183	183	5394	65	2010	F
3327_1994_S184_	2015	D	27.04.2015	S184	13.7	20	Fam-67_2010_	S184	184	5395	67	2010	F
3327_1994_S185_	2015	D	27.04.2015	S185	13.4	20	Fam-61_1_201	S185	185	5396	61	2010	F
3327_1994_S187_	2015	D	27.04.2015	S187	12.1	13.5	Fam-89_2010_	S187	187	5397	89	2010	W
3327_1994_S188_	2015	D	27.04.2015	S188	13.3	19	Fam-76_2010_	S188	188	5398	76	2010	H
3327_1995_S189_	2015	D	27.04.2015	S189	13.5	20	Fam-79_2010_	S189	189	5399	79	2010	F
3327_1995_S190_	2015	D	27.04.2015	S190	13.7	18.5	Fam-82_2010_	S190	190	5400	82	2010	W
3327_1995_S192_	2015	D	27.04.2015	S192	14.5	23.5	Fam-85_2010_	S192	192	5401	85	2010	W

3327_1995_S196_	2015	D	27.04.2015	S196	12.9	16.5	Fam-62_1_201	S196	196	5402	62	2010	H
3327_1995_S197_	2015	D	27.04.2015	S197	13.5	20	Fam-78_2010_	S197	197	5403	78	2010	H
3327_1995_S198_	2015	D	27.04.2015	S198	14.3	23.5	Fam-65_2010_	S198	198	5404	65	2010	F
3327_1995_S199_	2015	D	27.04.2015	S199	13.2	18.5	Fam-87_2010_	S199	199	5405	87	2010	W
3327_1995_S203_	2015	D	27.04.2015	S203	13.6	22.5	Fam-61_1_201	S203	203	5406	61	2010	F
3327_1995_S204_	2015	D	27.04.2015	S204	15.6	30.5	Fam-66_2010_	S204	204	5407	66	2010	H
3327_1995_S205_	2015	D	27.04.2015	S205	12.4	17	Fam-80_2010_	S205	205	5408	80	2010	H
3327_1995_S209_	2015	D	27.04.2015	S209	12.9	17.5	Fam-89_2010_	S209	209	5409	89	2010	W
3327_1995_S212_	2015	D	27.04.2015	S212	15.1	25.5	Fam-66_2010_	S212	212	5410	66	2010	H
3327_1995_S213_	2015	D	27.04.2015	S213	14.6	25.5	Fam-50_2009_	S213	213	5411	50	2009	H
3327_1995_S215_	2015	D	27.04.2015	S215	13.4	18.5	Fam-66_2010_	S215	215	5412	66	2010	H
3327_1995_S217_	2015	D	27.04.2015	S217	12.7	17	Fam-61_1_201	S217	217	5413	61	2010	F
3327_1995_S220_	2015	D	27.04.2015	S220	13.7	20	Fam-59_2009_	S220	220	5414	59	2009	W
3327_1995_S224_	2015	D	27.04.2015	S224	13.5	19	Fam-89_2010_	S224	224	5415	89	2010	W

3327_1995_S225_	2015	D	27.04.2015	S225	13.6	20	Fam-63_2010_	S225	225	5416	63	2010 F
3327_1995_S226_	2015	D	27.04.2015	S226	13.9	21	Fam-63_2010_	S226	226	5417	63	2010 F
3327_1995_S228_	2015	D	27.04.2015	S228	14.2	23	Fam-68_2010_	S228	228	5418	68	2010 H
3327_1995_S229_	2015	D	27.04.2015	S229	14.7	27	Fam-77_2010_	S229	229	5419	77	2010 F
3327_1995_S230_	2015	T	27.04.2015	S230	14.1	22	Family-31_200	S230	230	5420	31	2009 F
3327_1995_S231_	2015	D	27.04.2015	S231	13.4	20	Fam-72_2010_	S231	231	5421	72	2010 H
3327_1995_S232_	2015	D	27.04.2015	S232	12.9	18	Fam-64_2010_	S232	232	5422	64	2010 H
3327_1995_S233_	2015	D	27.04.2015	S233	14	22	Fam-83_2010_	S233	233	5423	83	2010 W
3327_1995_S234_	2015	D	27.04.2015	S234	14.1	23	Fam-64_2010_	S234	234	5424	64	2010 H
3327_1995_S235_	2015	D	27.04.2015	S235	14	23	Fam-89_2010_	S235	235	5425	89	2010 W
3327_1995_S236_	2015	D	27.04.2015	S236	15.5	32	Fam-79_2010_	S236	236	5426	79	2010 F
3327_1995_S238_	2015	D	27.04.2015	S238	13.5	20	Fam-66_2010_	S238	238	5427	66	2010 H
3327_1995_S239_	2015	D	27.04.2015	S239	14.2	21	Fam-75_2010_	S239	239	5428	75	2010 F
3327_1995_S240_	2015	D	27.04.2015	S240	13.7	20.5	Fam-78_2010_	S240	240	5429	78	2010 H

3328_1995_S243_	2015	D	27.04.2015	S243	14.4	24.5	Fam-65_2010_S243	243	5430	65	2010	F
3328_1995_S244_	2015	D	27.04.2015	S244	14	21.5	Fam-63_2010_S244	244	5431	63	2010	F
3328_1995_S245_	2015	D	27.04.2015	S245	13.6	19.5	Fam-71_2010_S245	245	5432	71	2010	F
3328_1995_S247_	2015	D	27.04.2015	S247	13.8	22	Fam-75_2010_S247	247	5433	75	2010	F
3328_1995_S248_	2015	D	27.04.2015	S248	12.9	16.5	Fam-76_2010_S248	248	5434	76	2010	H
3328_1995_S250_	2015	D	27.04.2015	S250	13.9	21.5	Fam-83_2010_S250	250	5435	83	2010	W
3328_1995_S251_	2015	D	27.04.2015	S251	12.8	18	Fam-90_2010_S251	251	5436	90	2010	W
3328_1995_S252_	2015	D	27.04.2015	S252	12.8	16.5	Fam-83_2010_S252	252	5437	83	2010	W
3328_1995_S253_	2015	D	27.04.2015	S253	12.9	16.5	Fam-83_2010_S253	253	5438	83	2010	W
3328_1995_S254_	2015	D	27.04.2015	S254	14.6	24.5	Fam-64_2010_S254	254	5439	64	2010	H
3328_1995_S256_	2015	D	27.04.2015	S256	11	15.5	Fam-67_2010_S256	256	5440	67	2010	F
3328_1995_S257_	2015	D	27.04.2015	S257	13.1	19	Fam-83_2010_S257	257	5441	83	2010	W
3328_1995_S259_	2015	D	27.04.2015	S259	14.1	23.5	Fam-65_2010_S259	259	5442	65	2010	F
3328_1995_S261_	2015	D	27.04.2015	S261	14.7	23	Fam-75_2010_S261	261	5443	75	2010	F

3328_1995_S262_	2015	D	27.04.2015	S262	12.6	15.5	Fam-89_2010_	S262	262	5444	89	2010 W
3328_1995_S264_	2015	D	27.04.2015	S264	15.5	29	Fam-66_2010_	S264	264	5445	66	2010 H
3328_1995_S267_	2015	D	27.04.2015	S267	13.7	21	Fam-80_2010_	S267	267	5446	80	2010 H
3328_1995_S269_	2015	D	27.04.2015	S269	15.9	35	Fam-04_2007_	S269	269	5447	4	2007 W
3328_1995_S271_	2015	D	27.04.2015	S271	13.7	20.5	Fam-65_2010_	S271	271	5448	65	2010 F
3328_1995_S272_	2015	D	27.04.2015	S272	12.8	18.5	Fam-78_2010_	S272	272	5449	78	2010 H
3328_1995_S273_	2015	D	27.04.2015	S273	12.8	17	Fam-85_2010_	S273	273	5450	85	2010 W
3328_1995_S274_	2015	D	27.04.2015	S274	14.7	26	Fam-62_1_201	S274	274	5451	62	2010 H
3328_1995_S275_	2015	D	27.04.2015	S275	12.4	15	Fam-69_2010_	S275	275	5452	69	2010 F
3328_1995_S278_	2015	D	27.04.2015	S278	13.5	20.5	Fam-65_2010_	S278	278	5453	65	2010 F
3328_1995_S279_	2015	D	27.04.2015	S279	13.6	21.5	Fam-62_1_201	S279	279	5454	62	2010 H
3328_1995_S280_	2015	D	27.04.2015	S280	13.6	20.5	Fam-77_2010_	S280	280	5455	77	2010 F
3328_1996_S283_	2015	D	27.04.2015	S283	13.6	19	Fam-61_1_201	S283	283	5456	61	2010 F
3328_1996_S284_	2015	D	27.04.2015	S284	14.2	23.5	Fam-87_2010_	S284	284	5457	87	2010 W

3328_1996_S285_	2015	D	27.04.2015	S285	13.2	19.5	Fam-80_2010_	S285	285	5458	80	2010	H
3328_1996_S287_	2015	D	27.04.2015	S287	12.5	16.5	Fam-63_2010_	S287	287	5459	63	2010	F
3328_1996_S288_	2015	D	27.04.2015	S288	13.6	21	Fam-72_2010_	S288	288	5460	72	2010	H
3328_1996_S289_	2015	D	27.04.2015	S289	13.6	18.5	Fam-89_2010_	S289	289	5461	89	2010	W
3328_1996_S291_	2015	D	27.04.2015	S291	14.4	24	Fam-85_2010_	S291	291	5462	85	2010	W
3328_1996_S292_	2015	D	27.04.2015	S292	13.8	20	Fam-64_2010_	S292	292	5463	64	2010	H
3328_1996_S293_	2015	D	27.04.2015	S293	16.1	34	Fam-65_2010_	S293	293	5464	65	2010	F
3328_1996_S294_	2015	D	27.04.2015	S294	14.4	24.5	Fam-89_2010_	S294	294	5465	89	2010	W
3328_1996_S296_	2015	D	27.04.2015	S296	14.6	23.5	Fam-78_2010_	S296	296	5466	78	2010	H
3328_1996_S298_	2015	D	27.04.2015	S298	13.3	18.5	Fam-85_2010_	S298	298	5467	85	2010	W
3328_1996_S299_	2015	D	27.04.2015	S299	12	15.5	Fam-78_2010_	S299	299	5468	78	2010	H
3328_1996_S301_	2015	D	27.04.2015	S301	14	21.5	Fam-75_2010_	S301	301	5469	75	2010	F
3328_1996_S302_	2015	D	27.04.2015	S302	14.4	23.5	Fam-70_2010_	S302	302	5470	70	2010	H
3328_1996_S303_	2015	D	27.04.2015	S303	13.6	20.5	Fam-65_2010_	S303	303	5471	65	2010	F

3328_1996_S305_	2015	D	27.04.2015	S305	13.6	22.5	Fam-90_2010_	S305	305	5472	90	2010	W
3328_1996_S306_	2015	D	27.04.2015	S306	12.9	18	Fam-70_2010_	S306	306	5473	70	2010	H
3328_1996_S307_	2015	D	27.04.2015	S307	13.3	18.5	Fam-64_2010_	S307	307	5474	64	2010	H
3328_1996_S309_	2015	D	27.04.2015	S309	13.2	18	Fam-83_2010_	S309	309	5475	83	2010	W
3328_1996_S310_	2015	D	27.04.2015	S310	13.7	22.5	Fam-65_2010_	S310	310	5476	65	2010	F
3328_1996_S312_	2015	D	27.04.2015	S312	13.4	18.5	Fam-54_2009_	S312	312	5477	54	2009	W
3328_1996_S313_	2015	D	27.04.2015	S313	13.7	21	Fam-62_1_201	S313	313	5478	62	2010	H
3328_1996_S317_	2015	D	27.04.2015	S317	16.1	31.5	Fam-76_2010_	S317	317	5479	76	2010	H
3328_1996_S318_	2015	D	27.04.2015	S318	13.2	17	Fam-84_2010_	S318	318	5480	84	2010	W
3328_1996_S319_	2015	D	27.04.2015	S319	12.2	15.5	Fam-64_2010_	S319	319	5481	64	2010	H
3328_1996_S320_	2015	D	27.04.2015	S320	12.8	16.5	Fam-61_1_201	S320	320	5482	61	2010	F
3329_1996_S321_	2015	D	27.04.2015	S321	12.9	17	Fam-90_2010_	S321	321	5483	90	2010	W
3329_1996_S322_	2015	D	27.04.2015	S322	13.4	19	Fam-79_2010_	S322	322	5484	79	2010	F
3329_1996_S324_	2015	D	27.04.2015	S324	13.6	21.5	Fam-73_2010_	S324	324	5485	73	2010	F

3329_1996_S326_	2015	D	27.04.2015	S326	13.6	21.5	Fam-72_2010_	S326	326	5486	72	2010	H
3329_1996_S327_	2015	D	27.04.2015	S327	13.5	19.5	Fam-55_2009_	S327	327	5487	55	2009	W
3329_1996_S328_	2015	T	27.04.2015	S328	12.8	14.5	Family-35_200	S328	328	5488	35	2009	F
3329_1996_S330_	2015	D	27.04.2015	S330	14.2	24.5	Fam-71_2010_	S330	330	5489	71	2010	F
3329_1996_S332_	2015	D	27.04.2015	S332	12.9	17.5	Fam-85_2010_	S332	332	5490	85	2010	W
3329_1996_S333_	2015	D	27.04.2015	S333	14	24	Fam-31_2009_	S333	333	5491	31	2009	F
3329_1996_S334_	2015	D	27.04.2015	S334	12.6	15.5	Fam-73_2010_	S334	334	5492	73	2010	F
3329_1996_S337_	2015	D	27.04.2015	S337	14	21.5	Fam-51_2009_	S337	337	5493	51	2009	W
3329_1996_S339_	2015	D	27.04.2015	S339	12.5	16	Fam-85_2010_	S339	339	5494	85	2010	W
3329_1996_S340_	2015	D	27.04.2015	S340	13.1	18.5	Fam-63_2010_	S340	340	5495	63	2010	F
3329_1996_S342_	2015	D	27.04.2015	S342	13.7	20.5	Fam-58_2009_	S342	342	5496	58	2009	W
3329_1996_S344_	2015	D	27.04.2015	S344	13.4	21.5	Fam-65_2010_	S344	344	5497	65	2010	F
3329_1996_S346_	2015	D	27.04.2015	S346	13.8	21	Fam-75_2010_	S346	346	5498	75	2010	F
3329_1996_S347_	2015	D	27.04.2015	S347	14.7	24	Fam-83_2010_	S347	347	5499	83	2010	W

3329_1996_S348_	2015	D	27.04.2015	S348	14.7	28	Fam-64_2010_	S348	348	5500	64	2010	H
3329_1996_S349_	2015	D	27.04.2015	S349	14.1	23	Fam-63_2010_	S349	349	5501	63	2010	F
3329_1996_S350_	2015	D	27.04.2015	S350	14.2	22	Fam-65_2010_	S350	350	5502	65	2010	F
3329_1996_S353_	2015	D	27.04.2015	S353	13.1	19	Fam-65_2010_	S353	353	5503	65	2010	F
3329_1996_S354_	2015	D	27.04.2015	S354	13.2	17.5	Fam-84_2010_	S354	354	5504	84	2010	W
3329_1996_S355_	2015	D	27.04.2015	S355	19	13.9	Fam-89_2010_	S355	355	5505	89	2010	W
3329_1996_S356_	2015	D	27.04.2015	S356	11.8	13	Fam-83_2010_	S356	356	5506	83	2010	W
3329_1996_S357_	2015	D	27.04.2015	S357	13.1	16.5	Fam-84_2010_	S357	357	5507	84	2010	W
3329_1996_S358_	2015	D	27.04.2015	S358	13.7	17	Fam-80_2010_	S358	358	5508	80	2010	H
3329_1996_S366_	2015	D	27.04.2015	S366	12.6	16.5	Fam-77_2010_	S366	366	5509	77	2010	F
3329_1996_S367_	2015	D	27.04.2015	S367	12.2	14	Fam-64_2010_	S367	367	5510	64	2010	H
3329_1996_S368_	2015	D	27.04.2015	S368	14.5	26	Fam-38_2009_	S368	368	5511	38	2009	H
3329_1996_S369_	2015	D	27.04.2015	S369	15	27.5	Fam-78_2010_	S369	369	5512	78	2010	H
3329_1996_S371_	2015	D	27.04.2015	S371	11.9	15	Fam-75_2010_	S371	371	5513	75	2010	F

3329_1996_S372_	2015	D	27.04.2015	S372	14.8	27.5	Fam-66_2010_	S372	372	5514	66	2010 H
3329_1996_S373_	2015	D	27.04.2015	S373	13.7	20.5	Fam-79_2010_	S373	373	5515	79	2010 F
3329_1996_S375_	2015	D	27.04.2015	S375	14.6	23.5	Fam-65_2010_	S375	375	5516	65	2010 F
3329_1996_S376_	2015	D	27.04.2015	S376	13.1	20.5	Fam-38_2009_	S376	376	5517	38	2009 H
3329_1997_S377_	2015	D	27.04.2015	S377	14.2	24.5	Fam-80_2010_	S377	377	5518	80	2010 H
3329_1997_S381_	2015	D	29.04.2015	S381	14.5	24.5	Fam-71_2010_	S381	381	5519	71	2010 F
3329_1997_S383_	2015	D	29.04.2015	S383	14.2	21.5	Fam-82_2010_	S383	383	5520	82	2010 W
3329_1997_S384_	2015	D	29.04.2015	S384	14.3	23.5	Fam-79_2010_	S384	384	5521	79	2010 F
3329_1997_S385_	2015	D	29.04.2015	S385	14.2	21	Fam-83_2010_	S385	385	5522	83	2010 W
3329_1997_S387_	2015	D	29.04.2015	S387	13	18	Fam-61_1_201	S387	387	5523	61	2010 F
3329_1997_S388_	2015	D	29.04.2015	S388	14.6	23.5	Fam-65_2010_	S388	388	5524	65	2010 F
3329_1997_S389_	2015	D	29.04.2015	S389	14.5	24.5	Fam-83_2010_	S389	389	5525	83	2010 W
3329_1997_S390_	2015	D	29.04.2015	S390	15.3	28.5	Fam-69_2010_	S390	390	5526	69	2010 F
3329_1997_S391_	2015	D	29.04.2015	S391	13.9	21.5	Fam-85_2010_	S391	391	5527	85	2010 W

3329_1997_S392_	2015	D	29.04.2015	S392	12.4	16	Fam-80_2010_	S392	392	5528	80	2010 H
3329_1997_S393_	2015	D	29.04.2015	S393	13.3	19	Fam-80_2010_	S393	393	5529	80	2010 H
3329_1997_S394_	2015	D	29.04.2015	S394	13.8	21	Fam-85_2010_	S394	394	5530	85	2010 W
3329_1997_S395_	2015	D	29.04.2015	S395	12.8	17	Fam-64_2010_	S395	395	5531	64	2010 H
3329_1997_S397_	2015	D	29.04.2015	S397	13.8	20.5	Fam-74_2010_	S397	397	5532	74	2010 H
3329_1997_S399_	2015	D	29.04.2015	S399	13	17	Fam-81_2010_	S399	399	5533	81	2010 W
3329_1997_S400_	2015	D	29.04.2015	S400	14.5	22	Fam-36_2009_	S400	400	5534	36	2009 H
3330_1997_S402_	2015	D	29.04.2015	S402	14.1	22	Fam-66_2010_	S402	402	5535	66	2010 H
3330_1997_S403_	2015	D	29.04.2015	S403	13.5	18.5	Fam-58_2009_	S403	403	5536	58	2009 W
3330_1997_S404_	2015	D	29.04.2015	S404	13.9	22.5	Fam-61_1_201	S404	404	5537	61	2010 F
3330_1997_S406_	2015	D	29.04.2015	S406	12.2	14	Fam-89_2010_	S406	406	5538	89	2010 W
3330_1997_S407_	2015	D	29.04.2015	S407	13.8	21	Fam-77_2010_	S407	407	5539	77	2010 F
3330_1997_S409_	2015	D	29.04.2015	S409	13.5	19	Fam-73_2010_	S409	409	5540	73	2010 F
3330_1997_S410_	2015	D	29.04.2015	S410	13.5	18.5	Fam-68_2010_	S410	410	5541	68	2010 H

3330_1997_S411_	2015	D	29.04.2015	S411	12.5	15.5	Fam-63_2010_	S411	411	5542	63	2010 F
3330_1997_S413_	2015	D	29.04.2015	S413	13.4	17.5	Fam-84_2010_	S413	413	5543	84	2010 W
3330_1997_S414_	2015	D	29.04.2015	S414	13.8	21	Fam-80_2010_	S414	414	5544	80	2010 H
3330_1997_S415_	2015	D	29.04.2015	S415	14.2	20.5	Fam-89_2010_	S415	415	5545	89	2010 W
3330_1997_S416_	2015	D	29.04.2015	S416	13	17	Fam-56_2009_	S416	416	5546	56	2009 W
3330_1997_S418_	2015	D	29.04.2015	S418	13.3	19.5	Fam-90_2010_	S418	418	5547	90	2010 W
3330_1997_S419_	2015	D	29.04.2015	S419	13.8	19	Fam-61_1_201	S419	419	5548	61	2010 F
3330_1997_S420_	2015	D	29.04.2015	S420	14.7	26	Fam-65_2010_	S420	420	5549	65	2010 F
3330_1997_S421_	2015	D	29.04.2015	S421	14.6	23	Fam-89_2010_	S421	421	5550	89	2010 W
3330_1997_S422_	2015	D	29.04.2015	S422	13.4	18	Fam-54_2009_	S422	422	5551	54	2009 W
3330_1997_S424_	2015	D	29.04.2015	S424	14.4	22	Fam-35_2009_	S424	424	5552	35	2009 F
3330_1997_S425_	2015	D	29.04.2015	S425	14.5	24	Fam-61_1_201	S425	425	5553	61	2010 F
3330_1997_S426_	2015	D	29.04.2015	S426	15.2	28.5	Fam-77_2010_	S426	426	5554	77	2010 F
3330_1997_S427_	2015	D	29.04.2015	S427	13.9	21	Fam-69_2010_	S427	427	5555	69	2010 F

3330_1997_S430_	2015	D	29.04.2015	S430	14	21	Fam-72_2010_	S430	430	5556	72	2010	H
3330_1997_S431_	2015	D	29.04.2015	S431	12	13.5	Fam-87_2010_	S431	431	5557	87	2010	W
3330_1997_S435_	2015	D	29.04.2015	S435	13.2	18.5	Fam-63_2010_	S435	435	5558	63	2010	F
3330_1997_S436_	2015	D	29.04.2015	S436	13.4	19.5	Fam-70_2010_	S436	436	5559	70	2010	H
3330_1997_S438_	2015	D	29.04.2015	S438	13.6	20	Fam-80_2010_	S438	438	5560	80	2010	H
3330_1997_S439_	2015	D	29.04.2015	S439	12.8	16.5	Fam-62_1_201	S439	439	5561	62	2010	H
3330_1997_S440_	2015	D	29.04.2015	S440	14.5	21.5	Fam-65_2010_	S440	440	5562	65	2010	F
3330_1997_S443_	2015	D	29.04.2015	S443	12.2	15	Fam-85_2010_	S443	443	5563	85	2010	W
3330_1997_S444_	2015	D	29.04.2015	S444	13.8	20	Fam-71_2010_	S444	444	5564	71	2010	F
3330_1997_S445_	2015	D	29.04.2015	S445	13.3	17.5	Fam-72_2010_	S445	445	5565	72	2010	H
3330_1997_S446_	2015	D	29.04.2015	S446	11.9	13.5	Fam-72_2010_	S446	446	5566	72	2010	H
3330_1997_S449_	2015	D	29.04.2015	S449	13.7	20.5	Fam-64_2010_	S449	449	5567	64	2010	H
3330_1997_S450_	2015	D	29.04.2015	S450	16.4	37.5	Fam-79_2010_	S450	450	5568	79	2010	F
3330_1997_S451_	2015	D	29.04.2015	S451	14	20.5	Fam-82_2010_	S451	451	5569	82	2010	W

3330_1997_S452_	2015	D	29.04.2015	S452	14	21.5	Fam-74_2010_S452	452	5570	74	2010 H
3330_1997_S454_	2015	D	29.04.2015	S454	12.6	17	Fam-83_2010_S454	454	5571	83	2010 W
3330_1997_S457_	2015	D	29.04.2015	S457	12.9	17.5	Fam-77_2010_S457	457	5572	77	2010 F
3330_1997_S458_	2015	D	29.04.2015	S458	13.7	21	Fam-72_2010_S458	458	5573	72	2010 H
3330_1997_S459_	2015	D	29.04.2015	S459	14.5	22.5	Fam-69_2010_S459	459	5574	69	2010 F
3330_1997_S460_	2015	D	29.04.2015	S460	13.4	19.5	Fam-90_2010_S460	460	5575	90	2010 W
3330_1997_S463_	2015	D	29.04.2015	S463	14.2	23.5	Fam-70_2010_S463	463	5576	70	2010 H
3330_1997_S464_	2015	D	29.04.2015	S464	14	22	Fam-85_2010_S464	464	5577	85	2010 W
3330_1997_S466_	2015	D	29.04.2015	S466	13.9	20.5	Fam-83_2010_S466	466	5578	83	2010 W
3330_1997_S469_	2015	D	29.04.2015	S469	13	16.5	Fam-76_2010_S469	469	5579	76	2010 H
3330_1998_S471_	2015	D	29.04.2015	S471	12.6	17	Fam-73_2010_S471	471	5580	73	2010 F
3330_1998_S472_	2015	D	29.04.2015	S472	14.7	23.5	Fam-72_2010_S472	472	5581	72	2010 H
3330_1998_S474_	2015	D	29.04.2015	S474	12.9	17.5	Fam-65_2010_S474	474	5582	65	2010 F
3330_1998_S475_	2015	D	29.04.2015	S475	13.2	20	Fam-31_2009_S475	475	5583	31	2009 F

3330_1998_S476_	2015	D	29.04.2015	S476	13.6	19.5	Fam-74_2010_	S476	476	5584	74	2010 H
3330_1998_S477_	2015	D	29.04.2015	S477	14.6	25	Fam-61_1_201	S477	477	5585	61	2010 F
3330_1998_S478_	2015	D	29.04.2015	S478	14.5	24	Fam-90_2010_	S478	478	5586	90	2010 W
3331_1998_S481_	2015	D	29.04.2015	S481	14.1	21.5	Fam-70_2010_	S481	481	5587	70	2010 H
3331_1998_S485_	2015	D	29.04.2015	S485	13.2	18	Fam-85_2010_	S485	485	5588	85	2010 W
3331_1998_S486_	2015	D	29.04.2015	S486	13.6	19	Fam-76_2010_	S486	486	5589	76	2010 H
3331_1998_S487_	2015	D	29.04.2015	S487	14.3	22.5	Fam-69_2010_	S487	487	5590	69	2010 F
3331_1998_S488_	2015	D	29.04.2015	S488	13.1	17.5	Fam-79_2010_	S488	488	5591	79	2010 F
3331_1998_S490_	2015	D	29.04.2015	S490	13.3	18.5	Fam-63_2010_	S490	490	5592	63	2010 F
3331_1998_S491_	2015	D	29.04.2015	S491	12.4	15.5	Fam-78_2010_	S491	491	5593	78	2010 H
3331_1998_S492_	2015	D	29.04.2015	S492	14.4	22.5	Fam-83_2010_	S492	492	5594	83	2010 W
3331_1998_S494_	2015	D	29.04.2015	S494	14.6	25	Fam-65_2010_	S494	494	5595	65	2010 F
3331_1998_S496_	2015	D	29.04.2015	S496	14.1	22.5	Fam-83_2010_	S496	496	5596	83	2010 W
3331_1998_S497_	2015	D	29.04.2015	S497	15	29	Fam-65_2010_	S497	497	5597	65	2010 F

3331_1998_S499_	2015	D	29.04.2015	S499	12.4	16.5	Fam-85_2010_	S499	499	5598	85	2010 W
3331_1998_S501_	2015	D	29.04.2015	S501	12.9	18	Fam-83_2010_	S501	501	5599	83	2010 W
3331_1998_S502_	2015	D	29.04.2015	S502	14.5	24.5	Fam-80_2010_	S502	502	5600	80	2010 H
3331_1998_S503_	2015	D	29.04.2015	S503	13.2	19	Fam-51_2009_	S503	503	5601	51	2009 W
3331_1998_S504_	2015	D	29.04.2015	S504	14.1	20.5	Fam-69_2010_	S504	504	5602	69	2010 F
3331_1998_S505_	2015	D	29.04.2015	S505	13.3	19.5	Fam-83_2010_	S505	505	5603	83	2010 W
3331_1998_S507_	2015	D	29.04.2015	S507	11.2	11.5	Fam-84_2010_	S507	507	5604	84	2010 W
3331_1998_S509_	2015	D	29.04.2015	S509	14.5	24	Fam-72_2010_	S509	509	5605	72	2010 H
3331_1998_S510_	2015	D	29.04.2015	S510	14	21	Fam-65_2010_	S510	510	5606	65	2010 F
3331_1998_S511_	2015	T	29.04.2015	S511	14.4	22	Family-35_200	S511	511	5607	35	2009 F
3331_1998_S512_	2015	D	29.04.2015	S512	14.4	24	Fam-61_1_201	S512	512	5608	61	2010 F
3331_1998_S515_	2015	D	29.04.2015	S515	13.5	19.5	Fam-83_2010_	S515	515	5609	83	2010 W
3331_1998_S518_	2015	D	29.04.2015	S518	13.9	20.5	Fam-66_2010_	S518	518	5610	66	2010 H
3331_1998_S520_	2015	D	29.04.2015	S520	14.3	24	Fam-76_2010_	S520	520	5611	76	2010 H

3331_1998_S525_	2015	D	29.04.2015	S525	12.6	16.5	Fam-65_2010_S525	525	5612	65	2010	F
3331_1998_S526_	2015	D	29.04.2015	S526	13.5	19	Fam-65_2010_S526	526	5613	65	2010	F
3331_1998_S527_	2015	D	29.04.2015	S527	14.6	25.5	Fam-69_2010_S527	527	5614	69	2010	F
3331_1998_S528_	2015	D	29.04.2015	S528	13	17.5	Fam-66_2010_S528	528	5615	66	2010	H
3331_1998_S529_	2015	D	29.04.2015	S529	14.4	24.5	Fam-58_2009_S529	529	5616	58	2009	W
3331_1998_S531_	2015	D	29.04.2015	S531	14.6	25.5	Fam-79_2010_S531	531	5617	79	2010	F
3331_1998_S532_	2015	D	29.04.2015	S532	15	26.5	Fam-64_2010_S532	532	5618	64	2010	H
3331_1998_S535_	2015	D	29.04.2015	S535	14	22.5	Fam-78_2010_S535	535	5619	78	2010	H
3331_1998_S538_	2015	D	29.04.2015	S538	14	20	Fam-64_2010_S538	538	5620	64	2010	H
3331_1998_S539_	2015	D	30.04.2015	S539	14	22.5	Fam-35_2009_S539	539	5621	35	2009	F
3331_1998_S540_	2015	D	30.04.2015	S540	14.1	22	Fam-80_2010_S540	540	5622	80	2010	H
3331_1998_S541_	2015	D	30.04.2015	S541	13.9	20	Fam-82_2010_S541	541	5623	82	2010	W
3331_1998_S543_	2015	D	30.04.2015	S543	13.4	18.5	Fam-83_2010_S543	543	5624	83	2010	W
3331_1998_S544_	2015	D	30.04.2015	S544	13.1	18	Fam-90_2010_S544	544	5625	90	2010	W

3331_1998_S546_	2015	D	30.04.2015	S546	13.3	19.5	Fam-73_2010_	S546	546	5626	73	2010	F
3331_1998_S547_	2015	D	30.04.2015	S547	14.1	22.5	Fam-77_2010_	S547	547	5627	77	2010	F
3331_1998_S548_	2015	D	30.04.2015	S548	14.2	21	Fam-75_2010_	S548	548	5628	75	2010	F
3331_1998_S549_	2015	D	30.04.2015	S549	15.2	27	Fam-54_2009_	S549	549	5629	54	2009	W
3331_1998_S551_	2015	D	30.04.2015	S551	12.9	17.5	Fam-90_2010_	S551	551	5630	90	2010	W
3331_1998_S553_	2015	D	30.04.2015	S553	13.2	20	Fam-77_2010_	S553	553	5631	77	2010	F
3331_1998_S554_	2015	D	30.04.2015	S554	12.4	16	Fam-76_2010_	S554	554	5632	76	2010	H
3331_1998_S555_	2015	D	30.04.2015	S555	13.9	22	Fam-65_2010_	S555	555	5633	65	2010	F
3331_1998_S556_	2015	D	30.04.2015	S556	14.8	27	Fam-65_2010_	S556	556	5634	65	2010	F
3331_1998_S559_	2015	D	30.04.2015	S559	12.7	19.5	Fam-75_2010_	S559	559	5635	75	2010	F
3331_1998_S560_	2015	D	30.04.2015	S560	14.1	21.5	Fam-66_2010_	S560	560	5636	66	2010	H
3332_1998_S561_	2015	D	30.04.2015	S561	14.3	22.5	Fam-87_2010_	S561	561	5637	87	2010	W
3332_1998_S562_	2015	D	30.04.2015	S562	12.7	16.5	Fam-84_2010_	S562	562	5638	84	2010	W
3332_1998_S563_	2015	D	30.04.2015	S563	16.1	33	Fam-75_2010_	S563	563	5639	75	2010	F

3332_1998_S564_	2015	D	30.04.2015	S564	14.3	22	Fam-64_2010_	S564	564	5640	64	2010	H
3332_1999_S565_	2015	D	30.04.2015	S565	13.5	19.5	Fam-89_2010_	S565	565	5641	89	2010	W
3332_1999_S566_	2015	D	30.04.2015	S566	14.4	24	Fam-65_2010_	S566	566	5642	65	2010	F
3332_1999_S567_	2015	D	30.04.2015	S567	14.8	26.5	Fam-71_2010_	S567	567	5643	71	2010	F
3332_1999_S568_	2015	D	30.04.2015	S568	13.2	18.5	Fam-85_2010_	S568	568	5644	85	2010	W
3332_1999_S569_	2015	D	30.04.2015	S569	15.4	30.5	Fam-63_2010_	S569	569	5645	63	2010	F
3332_1999_S570_	2015	D	30.04.2015	S570	13.3	17	Fam-53_2009_	S570	570	5646	53	2009	W
3332_1999_S571_	2015	D	30.04.2015	S571	13.8	21.5	Fam-63_2010_	S571	571	5647	63	2010	F
3332_1999_S573_	2015	D	30.04.2015	S573	13.4	19.5	Fam-67_2010_	S573	573	5648	67	2010	F
3332_1999_S574_	2015	D	30.04.2015	S574	13.6	20	Fam-61_1_201	S574	574	5649	61	2010	F
3332_1999_S575_	2015	D	30.04.2015	S575	13.4	18	Fam-61_1_201	S575	575	5650	61	2010	F
3332_1999_S576_	2015	D	30.04.2015	S576	14.5	25.5	Fam-77_2010_	S576	576	5651	77	2010	F
3332_1999_S579_	2015	D	30.04.2015	S579	14.4	24	Fam-63_2010_	S579	579	5652	63	2010	F
3332_1999_S582_	2015	D	30.04.2015	S582	14.7	25	Fam-89_2010_	S582	582	5653	89	2010	W

3332_1999_S585_	2015	D	30.04.2015	S585	12.4	17	Fam-90_2010_	S585	585	5654	90	2010	W
3332_1999_S586_	2015	D	30.04.2015	S586	14.3	23	Fam-77_2010_	S586	586	5655	77	2010	F
3332_1999_S588_	2015	D	30.04.2015	S588	13.6	19.5	Fam-85_2010_	S588	588	5656	85	2010	W
3332_1999_S589_	2015	D	30.04.2015	S589	14.5	24.5	Fam-77_2010_	S589	589	5657	77	2010	F
3332_1999_S590_	2015	D	30.04.2015	S590	13.4	19.5	Fam-79_2010_	S590	590	5658	79	2010	F
3332_1999_S592_	2015	D	30.04.2015	S592	13.6	20	Fam-83_2010_	S592	592	5659	83	2010	W
3332_1999_S593_	2015	D	30.04.2015	S593	12.9	17.5	Fam-83_2010_	S593	593	5660	83	2010	W
3332_1999_S594_	2015	D	30.04.2015	S594	14.6	26.5	Fam-68_2010_	S594	594	5661	68	2010	H
3332_1999_S596_	2015	T	30.04.2015	S596	13.5	17.5	Family-66_201	S596	596	5662	66	2010	H
3332_1999_S597_	2015	D	30.04.2015	S597	12.8	16	Fam-64_2010_	S597	597	5663	64	2010	H
3332_1999_S598_	2015	D	30.04.2015	S598	14.4	24	Fam-77_2010_	S598	598	5664	77	2010	F
3332_1999_S599_	2015	D	30.04.2015	S599	13.8	21.5	Fam-66_2010_	S599	599	5665	66	2010	H
3332_1999_S601_	2015	D	30.04.2015	S601	13.9	21	Fam-73_2010_	S601	601	5666	73	2010	F
3332_1999_S602_	2015	D	30.04.2015	S602	12.9	15.5	Fam-82_2010_	S602	602	5667	82	2010	W

3332_1999_S603_	2015	D	30.04.2015	S603	13.7	19.5	Fam-68_2010_	S603	603	5668	68	2010 H
3332_1999_S606_	2015	D	30.04.2015	S606	13.3	17	Fam-89_2010_	S606	606	5669	89	2010 W
3332_1999_S607_	2015	D	30.04.2015	S607	13.9	19.5	Fam-64_2010_	S607	607	5670	64	2010 H
3332_1999_S608_	2015	D	30.04.2015	S608	14.1	21	Fam-61_1_201	S608	608	5671	61	2010 F
3332_1999_S610_	2015	D	30.04.2015	S610	13.8	21.5	Fam-65_2010_	S610	610	5672	65	2010 F
3332_1999_S611_	2015	D	30.04.2015	S611	13	17.5	Fam-85_2010_	S611	611	5673	85	2010 W
3332_1999_S613_	2015	D	30.04.2015	S613	12.9	17.5	Fam-83_2010_	S613	613	5674	83	2010 W
3332_1999_S615_	2015	D	30.04.2015	S615	13.3	19.5	Fam-62_1_201	S615	615	5675	62	2010 H
3332_1999_S616_	2015	D	30.04.2015	S616	13.2	19	Fam-80_2010_	S616	616	5676	80	2010 H
3332_1999_S617_	2015	D	30.04.2015	S617	14.5	25	Fam-79_2010_	S617	617	5677	79	2010 F
3332_1999_S618_	2015	D	30.04.2015	S618	13.5	18.5	Fam-83_2010_	S618	618	5678	83	2010 W
3332_1999_S620_	2015	D	30.04.2015	S620	14	23	Fam-31_2009_	S620	620	5679	31	2009 F
3332_1999_S621_	2015	D	30.04.2015	S621	14.1	21	Fam-61_1_201	S621	621	5680	61	2010 F
3332_1999_S624_	2015	D	30.04.2015	S624	14.2	21	Fam-70_2010_	S624	624	5681	70	2010 H

3332_1999_S626_	2015	D	30.04.2015	S626	13.6	20.5	Fam-80_2010_	S626	626	5682	80	2010 H
3332_1999_S627_	2015	D	30.04.2015	S627	13.9	21.5	Fam-87_2010_	S627	627	5683	87	2010 W
3332_1999_S628_	2015	D	30.04.2015	S628	13.4	19	Fam-86_2010_	S628	628	5684	86	2010 W
3332_1999_S629_	2015	D	30.04.2015	S629	12.6	15.5	Fam-62_1_201	S629	629	5685	62	2010 H
3332_1999_S630_	2015	D	30.04.2015	S630	14.5	22	Fam-65_2010_	S630	630	5686	65	2010 F
3332_1999_S632_	2015	D	30.04.2015	S632	13.4	19	Fam-89_2010_	S632	632	5687	89	2010 W
3332_1999_S637_	2015	D	30.04.2015	S637	13.9	21	Fam-84_2010_	S637	637	5688	84	2010 W
3332_1999_S638_	2015	D	30.04.2015	S638	13.6	20	Fam-84_2010_	S638	638	5689	84	2010 W
3332_1999_S639_	2015	D	30.04.2015	S639	14.5	23	Fam-74_2010_	S639	639	5690	74	2010 H
3332_1999_S640_	2015	D	30.04.2015	S640	13.2	18.5	Fam-90_2010_	S640	640	5691	90	2010 W
3333_1999_S641_	2015	D	30.04.2015	S641	14.5	23.5	Fam-75_2010_	S641	641	5692	75	2010 F
3333_1999_S644_	2015	D	30.04.2015	S644	14.6	26	Fam-87_2010_	S644	644	5693	87	2010 W
3333_1999_S645_	2015	D	30.04.2015	S645	13	18	Fam-85_2010_	S645	645	5694	85	2010 W
3333_1999_S646_	2015	D	30.04.2015	S646	12.2	14	Fam-82_2010_	S646	646	5695	82	2010 W

3333_1999_S649_	2015	D	30.04.2015	S649	14.3	22.5	Fam-79_2010_	S649	649	5696	79	2010	F
3333_1999_S650_	2015	D	30.04.2015	S650	13.4	18.5	Fam-79_2010_	S650	650	5697	79	2010	F
3333_1999_S652_	2015	D	30.04.2015	S652	13.9	21	Fam-65_2010_	S652	652	5698	65	2010	F
3333_1999_S653_	2015	D	30.04.2015	S653	13.5	19	Fam-84_2010_	S653	653	5699	84	2010	W
3333_1999_S655_	2015	D	30.04.2015	S655	14.2	23.5	Fam-85_2010_	S655	655	5700	85	2010	W
3333_1999_S656_	2015	D	30.04.2015	S656	13.3	19	Fam-77_2010_	S656	656	5701	77	2010	F
3333_2000_S659_	2015	D	30.04.2015	S659	13.2	17.5	Fam-72_2010_	S659	659	5702	72	2010	H
3333_2000_S660_	2015	D	30.04.2015	S660	13.1	19.5	Fam-90_2010_	S660	660	5703	90	2010	W
3333_2000_S661_	2015	D	30.04.2015	S661	13.4	19	Fam-68_2010_	S661	661	5704	68	2010	H
3333_2000_S662_	2015	D	30.04.2015	S662	13.1	19.5	Fam-63_2010_	S662	662	5705	63	2010	F
3333_2000_S664_	2015	D	30.04.2015	S664	14.4	24.5	Fam-79_2010_	S664	664	5706	79	2010	F
3333_2000_S666_	2015	D	30.04.2015	S666	15.4	28	Fam-66_2010_	S666	666	5707	66	2010	H
3333_2000_S668_	2015	D	30.04.2015	S668	13.5	18	Fam-63_2010_	S668	668	5708	63	2010	F
3333_2000_S669_	2015	D	30.04.2015	S669	14.4	23	Fam-40_2009_	S669	669	5709	40	2009	H

3333_2000_S671_	2015	D	30.04.2015	S671	14.8	25	Fam-61_1_201	S671	671	5710	61	2010 F
3333_2000_S672_	2015	D	30.04.2015	S672	13.5	18.5	Fam-72_2010_	S672	672	5711	72	2010 H
3333_2000_S673_	2015	D	30.04.2015	S673	11.7	14	Fam-62_1_201	S673	673	5712	62	2010 H
3333_2000_S674_	2015	D	30.04.2015	S674	11.8	13	Fam-90_2010_	S674	674	5713	90	2010 W
3333_2000_S675_	2015	D	30.04.2015	S675	12.8	16	Fam-68_2010_	S675	675	5714	68	2010 H
3333_2000_S677_	2015	D	30.04.2015	S677	14.2	23	Fam-90_2010_	S677	677	5715	90	2010 W
3333_2000_S678_	2015	D	30.04.2015	S678	14.5	24	Fam-89_2010_	S678	678	5716	89	2010 W
3333_2000_S681_	2015	D	30.04.2015	S681	14.5	22	Fam-62_1_201	S681	681	5717	62	2010 H
3333_2000_S682_	2015	D	30.04.2015	S682	14.1	21.5	Fam-76_2010_	S682	682	5718	76	2010 H
3333_2000_S683_	2015	D	30.04.2015	S683	12.7	16	Fam-89_2010_	S683	683	5719	89	2010 W
3333_2000_S684_	2015	D	30.04.2015	S684	14	21.5	Fam-76_2010_	S684	684	5720	76	2010 H
3333_2000_S688_	2015	D	30.04.2015	S688	14.3	23.5	Fam-65_2010_	S688	688	5721	65	2010 F
3333_2000_S689_	2015	D	30.04.2015	S689	14	23	Fam-75_2010_	S689	689	5722	75	2010 F
3333_2000_S690_	2015	D	30.04.2015	S690	14.6	24	Fam-71_2010_	S690	690	5723	71	2010 F

3333_2000_S692_	2015	D	30.04.2015	S692	15	27.5	Fam-54_2009_	S692	692	5724	54	2009 W
3333_2000_S693_	2015	D	30.04.2015	S693	13.5	20.5	Fam-65_2010_	S693	693	5725	65	2010 F
3333_2000_S695_	2015	D	30.04.2015	S695	14.2	22.5	Fam-85_2010_	S695	695	5726	85	2010 W
3333_2000_S696_	2015	D	30.04.2015	S696	14.1	22	Fam-85_2010_	S696	696	5727	85	2010 W
3333_2000_S698_	2015	D	30.04.2015	S698	12.9	17.5	Fam-85_2010_	S698	698	5728	85	2010 W
3333_2000_S700_	2015	D	30.04.2015	S700	13.9	21.5	Fam-61_1_201	S700	700	5729	61	2010 F
3333_2000_S701_	2015	D	30.04.2015	S701	13	17	Fam-70_2010_	S701	701	5730	70	2010 H
3333_2000_S702_	2015	D	30.04.2015	S702	12.2	15	Fam-64_2010_	S702	702	5731	64	2010 H
3333_2000_S703_	2015	D	30.04.2015	S703	12.4	15.5	Fam-71_2010_	S703	703	5732	71	2010 F
3333_2000_S704_	2015	D	30.04.2015	S704	13.9	18.5	Fam-53_2009_	S704	704	5733	53	2009 W
3333_2000_S705_	2015	D	30.04.2015	S705	12.9	17.5	Fam-63_2010_	S705	705	5734	63	2010 F
3333_2000_S706_	2015	D	30.04.2015	S706	13.9	21.5	Fam-63_2010_	S706	706	5735	63	2010 F
3333_2000_S707_	2015	D	30.04.2015	S707	13.3	21	Fam-76_2010_	S707	707	5736	76	2010 H
3333_2000_S708_	2015	D	30.04.2015	S708	13.9	20.5	Fam-89_2010_	S708	708	5737	89	2010 W

3333_2000_S710_	2015	D	30.04.2015	S710	13.7	18.5	Fam-65_2010_	S710	710	5738	65	2010	F
3333_2000_S711_	2015	D	30.04.2015	S711	12.3	14	Fam-89_2010_	S711	711	5739	89	2010	W
3333_2000_S712_	2015	D	30.04.2015	S712	13.9	21	Fam-69_2010_	S712	712	5740	69	2010	F
3333_2000_S713_	2015	D	30.04.2015	S713	14	21	Fam-50_2009_	S713	713	5741	50	2009	H
3333_2000_S714_	2015	D	30.04.2015	S714	12.2	14.5	Fam-85_2010_	S714	714	5742	85	2010	W
3333_2000_S715_	2015	D	30.04.2015	S715	13.6	24	Fam-74_2010_	S715	715	5743	74	2010	H
3333_2000_S717_	2015	D	30.04.2015	S717	12.9	18.5	Fam-85_2010_	S717	717	5744	85	2010	W
3333_2000_S718_	2015	D	30.04.2015	S718	13.1	19	Fam-72_2010_	S718	718	5745	72	2010	H
3334_2000_S721_	2015	D	30.04.2015	S721	13.9	20.5	Fam-90_2010_	S721	721	5746	90	2010	W
3334_2000_S722_	2015	D	30.04.2015	S722	13.1	18.5	Fam-90_2010_	S722	722	5747	90	2010	W
3334_2000_S724_	2015	D	30.04.2015	S724	12.7	16	Fam-61_1_201	S724	724	5748	61	2010	F
3334_2000_S725_	2015	D	30.04.2015	S725	13.6	20.5	Fam-87_2010_	S725	725	5749	87	2010	W
3334_2000_S726_	2015	D	30.04.2015	S726	13.5	20.5	Fam-61_1_201	S726	726	5750	61	2010	F
3334_2000_S728_	2015	D	30.04.2015	S728	14.4	24.5	Fam-77_2010_	S728	728	5751	77	2010	F

3334_2000_S729_	2015	D	30.04.2015	S729	13.4	20	Fam-61_1_201	S729	729	5752	61	2010 F
3334_2000_S730_	2015	D	30.04.2015	S730	13.2	20	Fam-87_2010_	S730	730	5753	87	2010 W
3334_2000_S733_	2015	D	30.04.2015	S733	14.8	25.5	Fam-76_2010_	S733	733	5754	76	2010 H
3334_2000_S734_	2015	D	30.04.2015	S734	12.6	15.5	Fam-64_2010_	S734	734	5755	64	2010 H
3334_2000_S735_	2015	D	30.04.2015	S735	13.7	19.5	Fam-74_2010_	S735	735	5756	74	2010 H
3334_2000_S738_	2015	D	30.04.2015	S738	13.9	21	Fam-87_2010_	S738	738	5757	87	2010 W
3334_2000_S739_	2015	D	30.04.2015	S739	14.8	26	Fam-57_2009_	S739	739	5758	57	2009 W
3334_2000_S740_	2015	D	30.04.2015	S740	13.5	20.5	Fam-75_2010_	S740	740	5759	75	2010 F
3334_2000_S741_	2015	D	30.04.2015	S741	13.7	19	Fam-89_2010_	S741	741	5760	89	2010 W
3334_2000_S742_	2015	D	30.04.2015	S742	14.1	23	Fam-64_2010_	S742	742	5761	64	2010 H
3334_2000_S744_	2015	D	30.04.2015	S744	12.2	16.5	Fam-90_2010_	S744	744	5762	90	2010 W
3334_2000_S745_	2015	D	30.04.2015	S745	13.2	18.5	Fam-90_2010_	S745	745	5763	90	2010 W
3334_2000_S746_	2015	D	30.04.2015	S746	13.2	18	Fam-87_2010_	S746	746	5764	87	2010 W
3334_2000_S748_	2015	D	30.04.2015	S748	12.7	18	Fam-77_2010_	S748	748	5765	77	2010 F

3334_2000_S751_	2015	D	30.04.2015	S751	13.5	19	Fam-70_2010_S751	751	5766	70	2010	H
3334_2001_S755_	2015	D	30.04.2015	S755	14.6	22.5	Fam-69_2010_S755	755	5767	69	2010	F
3334_2001_S757_	2015	D	30.04.2015	S757	14	20	Fam-76_2010_S757	757	5768	76	2010	H
3334_2001_S759_	2015	D	30.04.2015	S759	13.3	18.5	Fam-78_2010_S759	759	5769	78	2010	H
3334_2001_S762_	2015	D	30.04.2015	S762	13.2	20	Fam-90_2010_S762	762	5770	90	2010	W
3334_2001_S763_	2015	D	30.04.2015	S763	13.3	18.5	Fam-87_2010_S763	763	5771	87	2010	W
3334_2001_S764_	2015	D	30.04.2015	S764	12.6	16.5	Fam-73_2010_S764	764	5772	73	2010	F
3334_2001_S766_	2015	D	30.04.2015	S766	14.8	26	Fam-69_2010_S766	766	5773	69	2010	F
3334_2001_S767_	2015	D	30.04.2015	S767	14.3	22	Fam-76_2010_S767	767	5774	76	2010	H
3334_2001_S768_	2015	D	30.04.2015	S768	11.4	12.5	Fam-87_2010_S768	768	5775	87	2010	W
3334_2001_S769_	2015	D	30.04.2015	S769	13.1	18.5	Fam-80_2010_S769	769	5776	80	2010	H
3334_2001_S772_	2015	D	30.04.2015	S772	14.8	25.5	Fam-76_2010_S772	772	5777	76	2010	H
3334_2001_S773_	2015	D	30.04.2015	S773	13.7	19.5	Fam-84_2010_S773	773	5778	84	2010	W
3334_2001_S774_	2015	D	30.04.2015	S774	14.2	22	Fam-65_2010_S774	774	5779	65	2010	F

3334_2001_S779_	2015	D	04.05.2015	S779	13.3	18.5	Fam-65_2010_	S779	779	5780	65	2010 F
3334_2001_S780_	2015	D	04.05.2015	S780	14.2	21.5	Fam-71_2010_	S780	780	5781	71	2010 F
3334_2001_S781_	2015	D	04.05.2015	S781	14.6	25	Fam-37_2009_	S781	781	5782	37	2009 F
3334_2001_S782_	2015	D	04.05.2015	S782	16.4	32	Fam-69_2010_	S782	782	5783	69	2010 F
3334_2001_S785_	2015	D	04.05.2015	S785	14.5	23	Fam-63_2010_	S785	785	5784	63	2010 F
3334_2001_S786_	2015	D	04.05.2015	S786	13.4	18.5	Fam-90_2010_	S786	786	5785	90	2010 W
3334_2001_S787_	2015	D	04.05.2015	S787	15.5	28	Fam-71_2010_	S787	787	5786	71	2010 F
3334_2001_S788_	2015	D	04.05.2015	S788	14.3	22.5	Fam-65_2010_	S788	788	5787	65	2010 F
3334_2001_S791_	2015	D	04.05.2015	S791	11.6	12.5	Fam-82_2010_	S791	791	5788	82	2010 W
3334_2001_S792_	2015	D	04.05.2015	S792	14.3	21.5	Fam-72_2010_	S792	792	5789	72	2010 H
3334_2001_S793_	2015	D	04.05.2015	S793	13.4	17.5	Fam-41_2009_	S793	793	5790	41	2009 F
3334_2001_S794_	2015	D	04.05.2015	S794	13.4	19	Fam-85_2010_	S794	794	5791	85	2010 W
3334_2001_S797_	2015	D	04.05.2015	S797	14.1	21	Fam-75_2010_	S797	797	5792	75	2010 F
3334_2001_S798_	2015	D	04.05.2015	S798	13.7	20	Fam-62_1_201	S798	798	5793	62	2010 H

3334_2001_S799_	2015	D	04.05.2015	S799	13.2	17	Fam-66_2010_	S799	799	5794	66	2010 H
3334_2001_S800_	2015	D	04.05.2015	S800	13.6	19	Fam-63_2010_	S800	800	5795	63	2010 F
3335_2001_S801_	2015	D	04.05.2015	S801	13.4	19	Fam-62_1_201	S801	801	5796	62	2010 H
3335_2001_S803_	2015	D	04.05.2015	S803	13.8	18	Fam-85_2010_	S803	803	5797	85	2010 W
3335_2001_S804_	2015	D	04.05.2015	S804	15	24	Fam-71_2010_	S804	804	5798	71	2010 F
3335_2001_S805_	2015	D	04.05.2015	S805	13.3	18.5	Fam-85_2010_	S805	805	5799	85	2010 W
3335_2001_S806_	2015	D	04.05.2015	S806	14.6	24.5	Fam-65_2010_	S806	806	5800	65	2010 F
3335_2001_S807_	2015	D	04.05.2015	S807	13.6	20	Fam-45_2009_	S807	807	5801	45	2009 F
3335_2001_S810_	2015	D	04.05.2015	S810	13	16	Fam-84_2010_	S810	810	5802	84	2010 W
3335_2001_S811_	2015	D	04.05.2015	S811	14.1	21.5	Fam-85_2010_	S811	811	5803	85	2010 W
3335_2001_S815_	2015	D	04.05.2015	S815	11.8	14	Fam-90_2010_	S815	815	5804	90	2010 W
3335_2001_S818_	2015	D	04.05.2015	S818	15.1	26	Fam-68_2010_	S818	818	5805	68	2010 H
3335_2001_S819_	2015	D	04.05.2015	S819	14.4	22.5	Fam-71_2010_	S819	819	5806	71	2010 F
3335_2001_S820_	2015	D	04.05.2015	S820	14.2	22.5	Fam-84_2010_	S820	820	5807	84	2010 W

3335_2001_S822_	2015	D	04.05.2015	S822	13.2	17	Fam-85_2010_	S822	822	5808	85	2010 W
3335_2001_S823_	2015	D	04.05.2015	S823	14.2	23	Fam-71_2010_	S823	823	5809	71	2010 F
3335_2001_S825_	2015	D	04.05.2015	S825	14.4	24.5	Fam-79_2010_	S825	825	5810	79	2010 F
3335_2001_S826_	2015	D	04.05.2015	S826	13.1	17.5	Fam-85_2010_	S826	826	5811	85	2010 W
3335_2001_S827_	2015	D	04.05.2015	S827	14	18.5	Fam-89_2010_	S827	827	5812	89	2010 W
3335_2001_S828_	2015	D	04.05.2015	S828	12.9	17.5	Fam-61_1_201	S828	828	5813	61	2010 F
3335_2001_S830_	2015	D	04.05.2015	S830	12.9	17	Fam-76_2010_	S830	830	5814	76	2010 H
3335_2001_S832_	2015	D	04.05.2015	S832	13.5	18.5	Fam-72_2010_	S832	832	5815	72	2010 H
3335_2001_S833_	2015	D	04.05.2015	S833	12.2	14.5	Fam-72_2010_	S833	833	5816	72	2010 H
3335_2001_S834_	2015	D	04.05.2015	S834	13.1	19	Fam-72_2010_	S834	834	5817	72	2010 H
3335_2001_S835_	2015	D	04.05.2015	S835	14.4	21	Fam-89_2010_	S835	835	5818	89	2010 W
3335_2001_S838_	2015	D	04.05.2015	S838	13	18.5	Fam-39_2009_	S838	838	5819	39	2009 F
3335_2001_S839_	2015	D	04.05.2015	S839	15	26.5	Fam-79_2010_	S839	839	5820	79	2010 F
3335_2001_S840_	2015	D	04.05.2015	S840	14.3	22.5	Fam-66_2010_	S840	840	5821	66	2010 H

3335_2001_S841_	2015	D	04.05.2015	S841	14.3	22	Fam-80_2010_S841	841	5822	80	2010	H
3335_2001_S843_	2015	D	04.05.2015	S843	14.7	24	Fam-69_2010_S843	843	5823	69	2010	F
3335_2001_S845_	2015	D	04.05.2015	S845	15.7	29.5	Fam-69_2010_S845	845	5824	69	2010	F
3335_2001_S846_	2015	D	04.05.2015	S846	14.7	24.5	Fam-77_2010_S846	846	5825	77	2010	F
3335_2002_S847_	2015	D	04.05.2015	S847	12.1	14.5	Fam-90_2010_S847	847	5826	90	2010	W
3335_2002_S848_	2015	D	04.05.2015	S848	12.3	15	Fam-77_2010_S848	848	5827	77	2010	F
3335_2002_S849_	2015	D	04.05.2015	S849	14	21	Fam-79_2010_S849	849	5828	79	2010	F
3335_2002_S850_	2015	D	04.05.2015	S850	14.7	24	Fam-74_2010_S850	850	5829	74	2010	H
3335_2002_S851_	2015	D	04.05.2015	S851	13.4	15.5	Fam-82_2010_S851	851	5830	82	2010	W
3335_2002_S852_	2015	D	04.05.2015	S852	14.2	21.5	Fam-72_2010_S852	852	5831	72	2010	H
3335_2002_S853_	2015	D	04.05.2015	S853	15	26.5	Fam-67_2010_S853	853	5832	67	2010	F
3335_2002_S855_	2015	D	04.05.2015	S855	13.8	20	Fam-83_2010_S855	855	5833	83	2010	W
3335_2002_S856_	2015	D	04.05.2015	S856	14.1	22	Fam-76_2010_S856	856	5834	76	2010	H
3335_2002_S857_	2015	D	04.05.2015	S857	14.8	25	Fam-64_2010_S857	857	5835	64	2010	H

3335_2002_S858_	2015	D	04.05.2015	S858	13.9	22	Fam-57_2009_	S858	858	5836	57	2009	W
3335_2002_S859_	2015	D	04.05.2015	S859	14.5	23.5	Fam-71_2010_	S859	859	5837	71	2010	F
3335_2002_S860_	2015	D	04.05.2015	S860	14.5	22.5	Fam-76_2010_	S860	860	5838	76	2010	H
3335_2002_S862_	2015	D	04.05.2015	S862	13.7	19	Fam-85_2010_	S862	862	5839	85	2010	W
3335_2002_S863_	2015	D	04.05.2015	S863	12.9	17	Fam-78_2010_	S863	863	5840	78	2010	H
3335_2002_S864_	2015	D	04.05.2015	S864	13.7	20.5	Fam-63_2010_	S864	864	5841	63	2010	F
3335_2002_S865_	2015	D	04.05.2015	S865	12	14.5	Fam-79_2010_	S865	865	5842	79	2010	F
3335_2002_S870_	2015	D	04.05.2015	S870	15	21.5	Fam-38_2009_	S870	870	5843	38	2009	H
3335_2002_S873_	2015	D	04.05.2015	S873	13.9	20	Fam-83_2010_	S873	873	5844	83	2010	W
3335_2002_S874_	2015	D	04.05.2015	S874	14.6	22	Fam-70_2010_	S874	874	5845	70	2010	H
3335_2002_S875_	2015	D	04.05.2015	S875	12.3	14.5	Fam-84_2010_	S875	875	5846	84	2010	W
3335_2002_S877_	2015	D	04.05.2015	S877	13.4	19	Fam-77_2010_	S877	877	5847	77	2010	F
3335_2002_S879_	2015	D	04.05.2015	S879	15	26	Fam-75_2010_	S879	879	5848	75	2010	F
3336_2002_S881_	2015	D	04.05.2015	S881	15.8	30	Fam-76_2010_	S881	881	5849	76	2010	H

3336_2002_S882_	2015	D	04.05.2015	S882	13.6	19.5	Fam-64_2010_	S882	882	5850	64	2010 H
3336_2002_S884_	2015	D	04.05.2015	S884	13.3	18	Fam-70_2010_	S884	884	5851	70	2010 H
3336_2002_S888_	2015	D	04.05.2015	S888	13.8	22	Fam-65_2010_	S888	888	5852	65	2010 F
3336_2002_S889_	2015	D	04.05.2015	S889	14.7	25.5	Fam-76_2010_	S889	889	5853	76	2010 H
3336_2002_S890_	2015	D	04.05.2015	S890	13.4	19.5	Fam-85_2010_	S890	890	5854	85	2010 W
3336_2002_S892_	2015	D	04.05.2015	S892	13.6	20.5	Fam-78_2010_	S892	892	5855	78	2010 H
3336_2002_S893_	2015	D	04.05.2015	S893	14.6	23.5	Fam-89_2010_	S893	893	5856	89	2010 W
3336_2002_S896_	2015	D	06.05.2015	S896	13.1	17	Fam-84_2010_	S896	896	5857	84	2010 W
3336_2002_S900_	2015	D	06.05.2015	S900	14.5	24	Fam-67_2010_	S900	900	5858	67	2010 F
3336_2002_S901_	2015	D	06.05.2015	S901	16.2	36.5	Fam-80_2010_	S901	901	5859	80	2010 H
3336_2002_S902_	2015	D	06.05.2015	S902	13.9	22	Fam-83_2010_	S902	902	5860	83	2010 W
3336_2002_S904_	2015	D	06.05.2015	S904	12	13.5	Fam-82_2010_	S904	904	5861	82	2010 W
3336_2002_S905_	2015	D	06.05.2015	S905	13.6	20	Fam-78_2010_	S905	905	5862	78	2010 H
3336_2002_S908_	2015	D	06.05.2015	S908	13.2	22	Fam-62_1_201	S908	908	5863	62	2010 H

3336_2002_S910_	2015	D	06.05.2015	S910	14.3	24	Fam-66_2010_	S910	910	5864	66	2010 H
3336_2002_S911_	2015	D	06.05.2015	S911	13.1	18.5	Fam-79_2010_	S911	911	5865	79	2010 F
3336_2002_S912_	2015	D	06.05.2015	S912	13.2	19	Fam-86_2010_	S912	912	5866	86	2010 W
3336_2002_S913_	2015	D	06.05.2015	S913	12.9	17	Fam-62_1_201	S913	913	5867	62	2010 H
3336_2002_S915_	2015	D	06.05.2015	S915	14.4	23.5	Fam-65_2010_	S915	915	5868	65	2010 F
3336_2002_S916_	2015	D	06.05.2015	S916	13.4	20	Fam-78_2010_	S916	916	5869	78	2010 H
3336_2002_S918_	2015	D	06.05.2015	S918	13	18.5	Fam-59_2009_	S918	918	5870	59	2009 W
3336_2002_S919_	2015	D	06.05.2015	S919	13.1	18	Fam-84_2010_	S919	919	5871	84	2010 W
3336_2002_S921_	2015	D	06.05.2015	S921	14.2	22	Fam-69_2010_	S921	921	5872	69	2010 F
3336_2002_S924_	2015	D	06.05.2015	S924	13.1	18	Fam-67_2010_	S924	924	5873	67	2010 F
3336_2002_S927_	2015	D	06.05.2015	S927	13.5	20.5	Fam-61_1_201	S927	927	5874	61	2010 F
3336_2002_S929_	2015	D	06.05.2015	S929	14.5	27	Fam-74_2010_	S929	929	5875	74	2010 H
3336_2002_S930_	2015	D	06.05.2015	S930	14.1	25.5	Fam-62_1_201	S930	930	5876	62	2010 H
3336_2002_S931_	2015	D	06.05.2015	S931	13.9	23	Fam-77_2010_	S931	931	5877	77	2010 F

3336_2002_S933_	2015	D	06.05.2015	S933	10.8	12.5	Fam-76_2010_	S933	933	5878	76	2010	H
3336_2002_S938_	2015	D	06.05.2015	S938	12.4	15	Fam-85_2010_	S938	938	5879	85	2010	W
3336_2002_S940_	2015	D	06.05.2015	S940	14.1	24.5	Fam-90_2010_	S940	940	5880	90	2010	W
3336_2003_S942_	2015	D	06.05.2015	S942	12.4	15.5	Fam-54_2009_	S942	942	5881	54	2009	W
3336_2003_S947_	2015	D	06.05.2015	S947	14.8	25.5	Fam-75_2010_	S947	947	5882	75	2010	F
3336_2003_S949_	2015	D	06.05.2015	S949	14.1	23.5	Fam-63_2010_	S949	949	5883	63	2010	F
3336_2003_S950_	2015	D	06.05.2015	S950	13	19.5	Fam-80_2010_	S950	950	5884	80	2010	H
3336_2003_S954_	2015	D	06.05.2015	S954	14.1	23	Fam-41_2009_	S954	954	5885	41	2009	F
3336_2003_S956_	2015	D	06.05.2015	S956	12.6	17	Fam-67_2010_	S956	956	5886	67	2010	F
3336_2003_S958_	2015	D	06.05.2015	S958	13.6	20	Fam-83_2010_	S958	958	5887	83	2010	W
3336_2003_S960_	2015	D	06.05.2015	S960	12.4	15.5	Fam-78_2010_	S960	960	5888	78	2010	H
3337_2003_S961_	2015	D	06.05.2015	S961	13.5	22	Fam-87_2010_	S961	961	5889	87	2010	W
3337_2003_S962_	2015	D	06.05.2015	S962	13.9	22	Fam-90_2010_	S962	962	5890	90	2010	W
3337_2003_S963_	2015	D	06.05.2015	S963	12.9	18.5	Fam-62_1_201	S963	963	5891	62	2010	H

3337_2003_S964_	2015	D	06.05.2015	S964	14.3	22	Fam-65_2010_	S964	964	5892	65	2010 F
3337_2003_S965_	2015	D	06.05.2015	S965	13.7	20	Fam-69_2010_	S965	965	5893	69	2010 F
3337_2003_S966_	2015	D	06.05.2015	S966	13.4	18.5	Fam-85_2010_	S966	966	5894	85	2010 W
3337_2003_S967_	2015	D	06.05.2015	S967	14.5	25	Fam-70_2010_	S967	967	5895	70	2010 H
3337_2003_S969_	2015	D	06.05.2015	S969	12.2	18.5	Fam-80_2010_	S969	969	5896	80	2010 H
3337_2003_S971_	2015	D	06.05.2015	S971	12.7	16	Fam-72_2010_	S971	971	5897	72	2010 H
3337_2003_S972_	2015	D	06.05.2015	S972	12.6	17.5	Fam-62_1_201	S972	972	5898	62	2010 H
3337_2003_S973_	2015	D	06.05.2015	S973	12.7	17.5	Fam-66_2010_	S973	973	5899	66	2010 H
3337_2003_S975_	2015	D	06.05.2015	S975	13.2	17.5	Fam-66_2010_	S975	975	5900	66	2010 H
3337_2003_S977_	2015	D	06.05.2015	S977	14.6	24.5	Fam-83_2010_	S977	977	5901	83	2010 W
3337_2003_S978_	2015	D	06.05.2015	S978	13.8	21.5	Fam-70_2010_	S978	978	5902	70	2010 H
3337_2003_S983_	2015	D	06.05.2015	S983	14.4	24.5	Fam-38_2009_	S983	983	5903	38	2009 H
3337_2003_S984_	2015	D	06.05.2015	S984	14.2	23.5	Fam-74_2010_	S984	984	5904	74	2010 H
3337_2003_S985_	2015	D	06.05.2015	S985	14.6	24.5	Fam-65_2010_	S985	985	5905	65	2010 F

3337_2003_S987_	2015	D	06.05.2015	S987	14.5	23.5	Fam-67_2010_	S987	987	5906	67	2010	F
3337_2003_S990_	2015	D	06.05.2015	S990	14.7	24.5	Fam-66_2010_	S990	990	5907	66	2010	H
3337_2003_S991_	2015	D	06.05.2015	S991	13.5	18.5	Fam-63_2010_	S991	991	5908	63	2010	F
3337_2003_S992_	2015	D	06.05.2015	S992	14.5	23.5	Fam-64_2010_	S992	992	5909	64	2010	H
3337_2003_S993_	2015	D	06.05.2015	S993	14.1	22	Fam-63_2010_	S993	993	5910	63	2010	F
3337_2003_S994_	2015	D	06.05.2015	S994	15.4	30	Fam-63_2010_	S994	994	5911	63	2010	F
3337_2003_S999_	2015	D	06.05.2015	S999	14.7	28.5	Fam-63_2010_	S999	999	5912	63	2010	F
3337_2003_S1000	2015	D	06.05.2015	S1000	13.5	20.6	Fam-87_2010_	S1000	1000	5913	87	2010	W
3337_2003_S1001	2015	D	06.05.2015	S1001	14.2	24.5	Fam-78_2010_	S1001	1001	5914	78	2010	H
3337_2003_S1002	2015	D	06.05.2015	S1002	14.5	26	Fam-77_2010_	S1002	1002	5915	77	2010	F
3337_2003_S1003	2015	D	06.05.2015	S1003	13.8	21.5	Fam-87_2010_	S1003	1003	5916	87	2010	W
3337_2003_S1006	2015	D	06.05.2015	S1006	14.1	22	Fam-73_2010_	S1006	1006	5917	73	2010	F
3337_2003_S1008	2015	D	06.05.2015	S1008	14.7	25	Fam-71_2010_	S1008	1008	5918	71	2010	F
3337_2003_S1009	2015	D	06.05.2015	S1009	15.2	28.5	Fam-66_2010_	S1009	1009	5919	66	2010	H

3337_2003_S1010	2015	D	06.05.2015	S1010	14.8	26	Fam-69_2010_ S1010	1010	5920	69	2010	F
3337_2003_S1012	2015	D	06.05.2015	S1012	14.4	24.5	Fam-78_2010_ S1012	1012	5921	78	2010	H
3337_2003_S1014	2015	D	06.05.2015	S1014	14.6	27	Fam-65_2010_ S1014	1014	5922	65	2010	F
3337_2003_S1016	2015	D	06.05.2015	S1016	14.9	24	Fam-89_2010_ S1016	1016	5923	89	2010	W
3337_2003_S1018	2015	D	06.05.2015	S1018	14.3	23	Fam-59_2009_ S1018	1018	5924	59	2009	W
3337_2003_S1019	2015	D	06.05.2015	S1019	13.4	19.5	Fam-32_2009_ S1019	1019	5925	32	2009	H
3337_2003_S1022	2015	D	06.05.2015	S1022	13.1	17.5	Fam-65_2010_ S1022	1022	5926	65	2010	F
3337_2003_S1023	2015	D	06.05.2015	S1023	13.2	19	Fam-63_2010_ S1023	1023	5927	63	2010	F
3337_2003_S1025	2015	D	06.05.2015	S1025	13.4	17	Fam-84_2010_ S1025	1025	5928	84	2010	W
3337_2003_S1026	2015	D	06.05.2015	S1026	12.9	17.5	Fam-71_2010_ S1026	1026	5929	71	2010	F
3337_2003_S1027	2015	D	06.05.2015	S1027	13.4	19	Fam-63_2010_ S1027	1027	5930	63	2010	F
3337_2003_S1029	2015	D	06.05.2015	S1029	15.2	28	Fam-90_2010_ S1029	1029	5931	90	2010	W
3337_2003_S1030	2015	D	06.05.2015	S1030	12.9	16	Fam-81_2010_ S1030	1030	5932	81	2010	W
3337_2003_S1031	2015	D	06.05.2015	S1031	13.7	21	Fam-57_2009_ S1031	1031	5933	57	2009	W

3337_2003_S1033	2015	D	06.05.2015	S1033	15.5	31	Fam-77_2010_	S1033	1033	5934	77	2010 F
3337_2004_S1036	2015	D	06.05.2015	S1036	13.8	22	Fam-62_1_201	S1036	1036	5935	62	2010 H
3337_2004_S1037	2015	D	06.05.2015	S1037	14.3	22.5	Fam-79_2010_	S1037	1037	5936	79	2010 F
3337_2004_S1038	2015	D	06.05.2015	S1038	12.5	17	Fam-61_1_201	S1038	1038	5937	61	2010 F
3337_2004_S1040	2015	D	06.05.2015	S1040	14.7	27	Fam-67_2010_	S1040	1040	5938	67	2010 F
3338_2004_S1043	2015	D	06.05.2015	S1043	14.4	23.5	Fam-84_2010_	S1043	1043	5939	84	2010 W
3338_2004_S1045	2015	D	06.05.2015	S1045	14.2	24	Fam-73_2010_	S1045	1045	5940	73	2010 F
3338_2004_S1046	2015	D	06.05.2015	S1046	14	21	Fam-77_2010_	S1046	1046	5941	77	2010 F
3338_2004_S1048	2015	D	06.05.2015	S1048	13.2	19.5	Fam-70_2010_	S1048	1048	5942	70	2010 H
3338_2004_S1050	2015	D	06.05.2015	S1050	12.6	15	Fam-67_2010_	S1050	1050	5943	67	2010 F
3338_2004_S1054	2015	D	06.05.2015	S1054	13.6	20	Fam-87_2010_	S1054	1054	5944	87	2010 W
3338_2004_S1056	2015	D	06.05.2015	S1056	14.8	28.5	Fam-83_2010_	S1056	1056	5945	83	2010 W
3338_2004_S1057	2015	D	06.05.2015	S1057	16.2	37	Fam-45_2009_	S1057	1057	5946	45	2009 F
3338_2004_S1058	2015	D	06.05.2015	S1058	13.6	23.5	Fam-54_2009_	S1058	1058	5947	54	2009 W

3338_2004_S1059	2015	D	06.05.2015	S1059	13.6	19.5	Fam-89_2010_	S1059	1059	5948	89	2010	W
3338_2004_S1060	2015	D	06.05.2015	S1060	13.4	20	Fam-77_2010_	S1060	1060	5949	77	2010	F
3338_2004_S1061	2015	D	06.05.2015	S1061	14.1	24.5	Fam-57_2009_	S1061	1061	5950	57	2009	W
3338_2004_S1062	2015	D	06.05.2015	S1062	14.2	23.5	Fam-80_2010_	S1062	1062	5951	80	2010	H
3338_2004_S1063	2015	D	06.05.2015	S1063	15.2	27.5	Fam-69_2010_	S1063	1063	5952	69	2010	F
3338_2004_S1064	2015	D	06.05.2015	S1064	12.8	18	Fam-42_2009_	S1064	1064	5953	42	2009	H
3338_2004_S1067	2015	D	06.05.2015	S1067	12.9	18.5	Fam-73_2010_	S1067	1067	5954	73	2010	F
3338_2004_S1068	2015	D	06.05.2015	S1068	14	23	Fam-67_2010_	S1068	1068	5955	67	2010	F
3338_2004_S1070	2015	D	06.05.2015	S1070	13.7	18	Fam-70_2010_	S1070	1070	5956	70	2010	H
3338_2004_S1071	2015	D	06.05.2015	S1071	13.9	22	Fam-83_2010_	S1071	1071	5957	83	2010	W
3338_2004_S1072	2015	D	06.05.2015	S1072	13.6	21	Fam-85_2010_	S1072	1072	5958	85	2010	W
3338_2004_S1076	2015	D	06.05.2015	S1076	14	24	Fam-87_2010_	S1076	1076	5959	87	2010	W
3338_2004_S1077	2015	D	06.05.2015	S1077	13.6	18.5	Fam-84_2010_	S1077	1077	5960	84	2010	W
3338_2004_S1078	2015	D	06.05.2015	S1078	13.5	22.5	Fam-80_2010_	S1078	1078	5961	80	2010	H

3338_2004_S108(	2015	D	06.05.2015	S1080	13.2	20	Fam-90_2010_	S1080	1080	5962	90	2010	W
3338_2004_S1083	2015	D	06.05.2015	S1083	14.4	24.5	Fam-68_2010_	S1083	1083	5963	68	2010	H
3338_2004_S1085	2015	D	06.05.2015	S1085	13.2	18.5	Fam-87_2010_	S1085	1085	5964	87	2010	W
3338_2004_S1086	2015	D	06.05.2015	S1086	14	23	Fam-57_2009_	S1086	1086	5965	57	2009	W
3338_2004_S1087	2015	D	06.05.2015	S1087	12.5	15.5	Fam-54_2009_	S1087	1087	5966	54	2009	W
3338_2004_S1093	2015	D	06.05.2015	S1093	15.1	26	Fam-76_2010_	S1093	1093	5967	76	2010	H
3338_2004_S1094	2015	T	06.05.2015	S1094	14.2	20	Family-65_201	S1094	1094	5968	65	2010	F
3338_2004_S1095	2015	D	06.05.2015	S1095	14.4	25	Fam-69_2010_	S1095	1095	5969	69	2010	F
3338_2004_S1097	2015	D	06.05.2015	S1097	13.1	18.5	Fam-56_2009_	S1097	1097	5970	56	2009	W
3338_2004_S1098	2015	D	06.05.2015	S1098	12.5	15.5	Fam-59_2009_	S1098	1098	5971	59	2009	W
3338_2004_S1099	2015	D	06.05.2015	S1099	12.8	17.5	Fam-68_2010_	S1099	1099	5972	68	2010	H
3338_2004_S1101	2015	D	06.05.2015	S1101	12.6	16.5	Fam-63_2010_	S1101	1101	5973	63	2010	F
3338_2004_S1104	2015	D	06.05.2015	S1104	13.6	19	Fam-61_1_201	S1104	1104	5974	61	2010	F
3338_2004_S1105	2015	D	06.05.2015	S1105	13.1	18.5	Fam-62_1_201	S1105	1105	5975	62	2010	H

3338_2004_S1106	2015	D	06.05.2015	S1106	13.8	20.5	Fam-77_2010_	S1106	1106	5976	77	2010	F
3338_2004_S1107	2015	D	06.05.2015	S1107	13.2	18	Fam-89_2010_	S1107	1107	5977	89	2010	W
3338_2004_S1115	2015	D	06.05.2015	S1115	13.7	22.5	Fam-62_1_201	S1115	1115	5978	62	2010	H
3338_2004_S1117	2015	T	06.05.2015	S1117	14	20.5	Family-65_201	S1117	1117	5979	65	2010	F
3338_2004_S1118	2015	D	06.05.2015	S1118	14.5	26	Fam-85_2010_	S1118	1118	5980	85	2010	W
3338_2004_S1119	2015	D	06.05.2015	S1119	12.8	16.5	Fam-83_2010_	S1119	1119	5981	83	2010	W
3339_2004_S1121	2015	D	06.05.2015	S1121	13.4	20	Fam-76_2010_	S1121	1121	5982	76	2010	H
3339_2004_S1122	2015	D	06.05.2015	S1122	14.5	26	Fam-65_2010_	S1122	1122	5983	65	2010	F
3339_2004_S1123	2015	D	06.05.2015	S1123	12.5	18.5	Fam-76_2010_	S1123	1123	5984	76	2010	H
3339_2004_S1124	2015	D	06.05.2015	S1124	12.9	17.5	Fam-70_2010_	S1124	1124	5985	70	2010	H
3339_2004_S1126	2015	D	06.05.2015	S1126	13.2	21	Fam-60_2009_	S1126	1126	5986	60	2009	W
3339_2004_S1127	2015	D	06.05.2015	S1127	13.6	20	Fam-70_2010_	S1127	1127	5987	70	2010	H
3339_2004_S1128	2015	D	06.05.2015	S1128	13.4	19	Fam-84_2010_	S1128	1128	5988	84	2010	W
3339_2005_S1130	2015	D	06.05.2015	S1130	13.7	20	Fam-87_2010_	S1130	1130	5989	87	2010	W

3339_2005_S113[illegible]	2015	D	06.05.2015	S1132	13.3	18.5	Fam-80_2010_S1132	1132	5990	80	2010	H
3339_2005_S113[illegible]	2015	D	06.05.2015	S1133	13.3	19.5	Fam-64_2010_S1133	1133	5991	64	2010	H
3339_2005_S113[illegible]	2015	D	06.05.2015	S1136	13.6	19	Fam-89_2010_S1136	1136	5992	89	2010	W
3339_2005_S113[illegible]	2015	D	06.05.2015	S1138	13	19	Fam-63_2010_S1138	1138	5993	63	2010	F
3339_2005_S113[illegible]	2015	D	06.05.2015	S1139	13.7	20.5	Fam-83_2010_S1139	1139	5994	83	2010	W
3339_2005_S114[illegible]	2015	D	06.05.2015	S1140	14.1	22.5	Fam-70_2010_S1140	1140	5995	70	2010	H
3339_2005_S1141	2015	D	06.05.2015	S1141	14.5	24.5	Fam-66_2010_S1141	1141	5996	66	2010	H
3339_2005_S114[illegible]	2015	D	06.05.2015	S1143	15.6	31.5	Fam-75_2010_S1143	1143	5997	75	2010	F
3339_2005_S114[illegible]	2015	D	06.05.2015	S1149	13.8	20	Fam-54_2009_S1149	1149	5998	54	2009	W
3339_2005_S115[illegible]	2015	D	06.05.2015	S1150	13.2	17.5	Fam-78_2010_S1150	1150	5999	78	2010	H
3339_2005_S1151	2015	D	06.05.2015	S1151	14	22.5	Fam-64_2010_S1151	1151	6000	64	2010	H
3339_2005_S115[illegible]	2015	D	06.05.2015	S1153	14.3	23.5	Fam-74_2010_S1153	1153	6001	74	2010	H
3339_2005_S1154	2015	D	06.05.2015	S1154	13.8	22.5	Fam-54_2009_S1154	1154	6002	54	2009	W
3339_2005_S115[illegible]	2015	D	06.05.2015	S1155	14.6	24.5	Fam-74_2010_S1155	1155	6003	74	2010	H

3339_2005_S1156	2015	D	06.05.2015	S1156	13.7	19.5	Fam-76_2010_	S1156	1156	6004	76	2010	H
3339_2005_S1157	2015	D	06.05.2015	S1157	14.5	22.5	Fam-54_2009_	S1157	1157	6005	54	2009	W
3339_2005_S1158	2015	D	06.05.2015	S1158	12.3	15	Fam-81_2010_	S1158	1158	6006	81	2010	W
3339_2005_S1161	2015	D	06.05.2015	S1161	13.2	16.5	Fam-54_2009_	S1161	1161	6007	54	2009	W
3339_2005_S1162	2015	D	06.05.2015	S1162	12.3	14.5	Fam-85_2010_	S1162	1162	6008	85	2010	W
3339_2005_S1164	2015	D	06.05.2015	S1164	13.1	17.5	Fam-84_2010_	S1164	1164	6009	84	2010	W
3339_2005_S1165	2015	D	06.05.2015	S1165	13.8	20	Fam-64_2010_	S1165	1165	6010	64	2010	H
3339_2005_S1166	2015	D	06.05.2015	S1166	13.9	20	Fam-87_2010_	S1166	1166	6011	87	2010	W
3339_2005_S1168	2015	D	06.05.2015	S1168	13.9	21.5	Fam-66_2010_	S1168	1168	6012	66	2010	H
3339_2005_S1169	2015	T	06.05.2015	S1169	13.9	21	Family-64_201	S1169	1169	6013	64	2010	H
3339_2005_S1170	2015	D	06.05.2015	S1170	12.4	15.5	Fam-83_2010_	S1170	1170	6014	83	2010	W
3339_2005_S1172	2015	D	06.05.2015	S1172	13.1	18	Fam-85_2010_	S1172	1172	6015	85	2010	W
3339_2005_S1173	2015	D	06.05.2015	S1173	13.9	21.5	Fam-66_2010_	S1173	1173	6016	66	2010	H
3339_2005_S1174	2015	D	06.05.2015	S1174	13.7	21.5	Fam-77_2010_	S1174	1174	6017	77	2010	F

3339_2005_S1175	2015	D	06.05.2015	S1175	14.2	22	Fam-70_2010_	S1175	1175	6018	70	2010 H
3339_2005_S1176	2015	D	06.05.2015	S1176	14.6	25.5	Fam-64_2010_	S1176	1176	6019	64	2010 H
3339_2005_S1177	2015	D	06.05.2015	S1177	14.7	25	Fam-66_2010_	S1177	1177	6020	66	2010 H
3339_2005_S1179	2015	D	06.05.2015	S1179	13.6	20	Fam-85_2010_	S1179	1179	6021	85	2010 W
3339_2005_S1180	2015	D	06.05.2015	S1180	13.5	18.5	Fam-76_2010_	S1180	1180	6022	76	2010 H
3339_2005_S1181	2015	D	06.05.2015	S1181	12.5	16	Fam-66_2010_	S1181	1181	6023	66	2010 H
3339_2005_S1183	2015	D	06.05.2015	S1183	14	22.5	Fam-62_1_201	S1183	1183	6024	62	2010 H
3339_2005_S1184	2015	D	06.05.2015	S1184	14.1	21.5	Fam-80_2010_	S1184	1184	6025	80	2010 H
3339_2005_S1186	2015	D	06.05.2015	S1186	13.9	22	Fam-78_2010_	S1186	1186	6026	78	2010 H
3339_2005_S1188	2015	D	06.05.2015	S1188	14.2	24.5	Fam-38_2009_	S1188	1188	6027	38	2009 H
3339_2005_S1190	2015	D	06.05.2015	S1190	12.5	14.5	Fam-65_2010_	S1190	1190	6028	65	2010 F
3339_2005_S1191	2015	D	06.05.2015	S1191	12.9	17	Fam-65_2010_	S1191	1191	6029	65	2010 F
3339_2005_S1192	2015	D	07.05.2015	S1192	13	18	Fam-64_2010_	S1192	1192	6030	64	2010 H
3339_2005_S1193	2015	D	07.05.2015	S1193	14.5	24	Fam-85_2010_	S1193	1193	6031	85	2010 W

3339_2005_S1194	2015	D	07.05.2015	S1194	13.8	21	Fam-78_2010_	S1194	1194	6032	78	2010	H
3339_2005_S1195	2015	D	07.05.2015	S1195	14.8	26	Fam-83_2010_	S1195	1195	6033	83	2010	W
3339_2005_S1197	2015	D	07.05.2015	S1197	13.6	20.5	Fam-68_2010_	S1197	1197	6034	68	2010	H
3339_2005_S1199	2015	D	07.05.2015	S1199	13.7	19.5	Fam-66_2010_	S1199	1199	6035	66	2010	H
3340_2005_S1201	2015	D	07.05.2015	S1201	13.6	21	Fam-87_2010_	S1201	1201	6036	87	2010	W
3340_2005_S1202	2015	D	07.05.2015	S1202	12.4	15	Fam-62_1_201	S1202	1202	6037	62	2010	H
3340_2005_S1205	2015	D	07.05.2015	S1205	13.4	20.5	Fam-65_2010_	S1205	1205	6038	65	2010	F
3340_2005_S1206	2015	D	07.05.2015	S1206	14.6	28.5	Fam-45_2009_	S1206	1206	6039	45	2009	F
3340_2005_S1207	2015	D	07.05.2015	S1207	12.9	18.5	Fam-87_2010_	S1207	1207	6040	87	2010	W
3340_2005_S1209	2015	D	07.05.2015	S1209	13.9	22.5	Fam-66_2010_	S1209	1209	6041	66	2010	H
3340_2005_S1210	2015	D	07.05.2015	S1210	13.6	19.5	Fam-62_1_201	S1210	1210	6042	62	2010	H
3340_2005_S1214	2015	D	07.05.2015	S1214	14	22	Fam-78_2010_	S1214	1214	6043	78	2010	H
3340_2005_S1216	2015	D	07.05.2015	S1216	13.1	17	Fam-89_2010_	S1216	1216	6044	89	2010	W
3340_2005_S1217	2015	D	07.05.2015	S1217	13.2	16.5	Fam-62_1_201	S1217	1217	6045	62	2010	H

3340_2005_S1219	2015	D	07.05.2015	S1219	13.3	18.5	Fam-76_2010_	S1219	1219	6046	76	2010	H
3340_2005_S1220	2015	D	07.05.2015	S1220	12.2	16	Fam-54_2009_	S1220	1220	6047	54	2009	W
3340_2005_S1222	2015	D	07.05.2015	S1222	14.4	24	Fam-71_2010_	S1222	1222	6048	71	2010	F
3340_2006_S1224	2015	D	07.05.2015	S1224	13.2	20	Fam-83_2010_	S1224	1224	6049	83	2010	W
3340_2006_S1228	2015	D	07.05.2015	S1228	13.9	22	Fam-84_2010_	S1228	1228	6050	84	2010	W
3340_2006_S1229	2015	D	07.05.2015	S1229	13.3	19	Fam-83_2010_	S1229	1229	6051	83	2010	W
3340_2006_S1232	2015	D	07.05.2015	S1232	14	23.5	Fam-50_2009_	S1232	1232	6052	50	2009	H
3340_2006_S1234	2015	T	07.05.2015	S1234	14.5	24.5	Family-42_200	S1234	1234	6053	42	2009	H
3340_2006_S1235	2015	D	07.05.2015	S1235	15.5	31.5	Fam-87_2010_	S1235	1235	6054	87	2010	W
3340_2006_S1236	2015	D	07.05.2015	S1236	13.7	21	Fam-63_2010_	S1236	1236	6055	63	2010	F
3340_2006_S1239	2015	D	07.05.2015	S1239	14.2	23.5	Fam-64_2010_	S1239	1239	6056	64	2010	H
3340_2006_S1240	2015	D	07.05.2015	S1240	15.2	30.5	Fam-78_2010_	S1240	1240	6057	78	2010	H
3340_2006_S1244	2015	D	07.05.2015	S1244	14.1	23	Fam-77_2010_	S1244	1244	6058	77	2010	F
3340_2006_S1245	2015	D	07.05.2015	S1245	12.8	17	Fam-72_2010_	S1245	1245	6059	72	2010	H

3340_2006_S1248	2015	D	07.05.2015	S1248	13.2	18	Fam-84_2010_	S1248	1248	6060	84	2010	W
3340_2006_S1249	2015	D	07.05.2015	S1249	9.8	11	Fam-76_2010_	S1249	1249	6061	76	2010	H
3340_2006_S1250	2015	D	07.05.2015	S1250	12.1	16	Fam-90_2010_	S1250	1250	6062	90	2010	W
3340_2006_S1252	2015	D	07.05.2015	S1252	13.4	18.5	Fam-71_2010_	S1252	1252	6063	71	2010	F
3340_2006_S1254	2015	D	07.05.2015	S1254	13.4	19	Fam-79_2010_	S1254	1254	6064	79	2010	F
3340_2006_S1255	2015	D	07.05.2015	S1255	14.3	25	Fam-61_1_201	S1255	1255	6065	61	2010	F
3340_2006_S1256	2015	D	07.05.2015	S1256	13.2	18.5	Fam-54_2009_	S1256	1256	6066	54	2009	W
3340_2006_S1257	2015	D	07.05.2015	S1257	14.3	25	Fam-77_2010_	S1257	1257	6067	77	2010	F
3340_2006_S1258	2015	D	07.05.2015	S1258	13.2	18	Fam-84_2010_	S1258	1258	6068	84	2010	W
3340_2006_S1259	2015	D	07.05.2015	S1259	14.2	24.5	Fam-38_2009_	S1259	1259	6069	38	2009	H
3340_2006_S1266	2015	D	07.05.2015	S1266	13.4	20.5	Fam-78_2010_	S1266	1266	6070	78	2010	H
3340_2006_S1271	2015	D	07.05.2015	S1271	14.6	24.5	Fam-79_2010_	S1271	1271	6071	79	2010	F
3340_2006_S1273	2015	D	07.05.2015	S1273	13.2	18.5	Fam-83_2010_	S1273	1273	6072	83	2010	W
3340_2006_S1274	2015	D	07.05.2015	S1274	15.2	29.5	Fam-63_2010_	S1274	1274	6073	63	2010	F

3340_2006_S1276	2015	D	07.05.2015	S1276	12.8	18.5	Fam-83_2010_ S1276	1276	6074	83	2010	W
3340_2006_S1278	2015	D	07.05.2015	S1278	14.7	24.5	Fam-69_2010_ S1278	1278	6075	69	2010	F
3340_2006_S1279	2015	D	07.05.2015	S1279	13.5	20	Fam-73_2010_ S1279	1279	6076	73	2010	F
3340_2006_S1280	2015	D	07.05.2015	S1280	13.7	17	Fam-63_2010_ S1280	1280	6077	63	2010	F
3341_2006_S1282	2015	D	07.05.2015	S1282	13.1	18.5	Fam-83_2010_ S1282	1282	6078	83	2010	W
3341_2006_S1284	2015	D	07.05.2015	S1284	11.7	13.5	Fam-87_2010_ S1284	1284	6079	87	2010	W
3341_2006_S1286	2015	D	07.05.2015	S1286	15.2	29.5	Fam-67_2010_ S1286	1286	6080	67	2010	F
3341_2006_S1287	2015	D	07.05.2015	S1287	13.2	19	Fam-83_2010_ S1287	1287	6081	83	2010	W
3341_2006_S1288	2015	D	07.05.2015	S1288	14.3	24	Fam-65_2010_ S1288	1288	6082	65	2010	F
3341_2006_S1289	2015	D	07.05.2015	S1289	13.2	18	Fam-58_2009_ S1289	1289	6083	58	2009	W
3341_2006_S1290	2015	D	07.05.2015	S1290	13.4	22.5	Fam-90_2010_ S1290	1290	6084	90	2010	W
3341_2006_S1292	2015	D	07.05.2015	S1292	14.1	20.5	Fam-84_2010_ S1292	1292	6085	84	2010	W
3341_2006_S1296	2015	D	07.05.2015	S1296	12.2	14	Fam-82_2010_ S1296	1296	6086	82	2010	W
3341_2006_S1298	2015	D	07.05.2015	S1298	14.1	22.5	Fam-66_2010_ S1298	1298	6087	66	2010	H

3341_2006_S1299	2015	D	07.05.2015	S1299	13.6	20	Fam-62_1_201	S1299	1299	6088	62	2010	H
3341_2006_S1300	2015	D	07.05.2015	S1300	13.6	19	Fam-66_2010_	S1300	1300	6089	66	2010	H
3341_2006_S1303	2015	D	07.05.2015	S1303	15.4	29	Fam-37_2009_	S1303	1303	6090	37	2009	F
3341_2006_S1306	2015	D	07.05.2015	S1306	14	21	Fam-63_2010_	S1306	1306	6091	63	2010	F
3341_2006_S1307	2015	D	07.05.2015	S1307	13.7	21	Fam-74_2010_	S1307	1307	6092	74	2010	H
3341_2006_S1308	2015	D	07.05.2015	S1308	13.8	21.5	Fam-87_2010_	S1308	1308	6093	87	2010	W
3341_2006_S1314	2015	D	07.05.2015	S1314	13.5	21.5	Fam-62_1_201	S1314	1314	6094	62	2010	H
3341_2006_S1315	2015	D	07.05.2015	S1315	14.2	25	Fam-69_2010_	S1315	1315	6095	69	2010	F
3341_2007_S1317	2015	D	07.05.2015	S1317	14.6	25	Fam-73_2010_	S1317	1317	6096	73	2010	F
3341_2007_S1319	2015	D	07.05.2015	S1319	12.6	15.5	Fam-64_2010_	S1319	1319	6097	64	2010	H
3341_2007_S1320	2015	D	07.05.2015	S1320	14.1	22.5	Fam-76_2010_	S1320	1320	6098	76	2010	H
3341_2007_S1322	2015	D	07.05.2015	S1322	12.7	18	Fam-62_1_201	S1322	1322	6099	62	2010	H
3341_2007_S1325	2015	D	07.05.2015	S1325	14.2	22	Fam-78_2010_	S1325	1325	6100	78	2010	H
3341_2007_S1327	2015	D	07.05.2015	S1327	13.4	19	Fam-65_2010_	S1327	1327	6101	65	2010	F

3341_2007_S1330	2015	D	07.05.2015	S1330	14.5	26	Fam-67_2010_	S1330	1330	6102	67	2010	F
3341_2007_S1332	2015	D	07.05.2015	S1332	13.9	19	Fam-43_2009_	S1332	1332	6103	43	2009	F
3341_2007_S1335	2015	D	07.05.2015	S1335	13.7	19	Fam-67_2010_	S1335	1335	6104	67	2010	F
3341_2007_S1336	2015	D	07.05.2015	S1336	13.6	16	Fam-83_2010_	S1336	1336	6105	83	2010	W
3341_2007_S1338	2015	D	07.05.2015	S1338	14	23	Fam-87_2010_	S1338	1338	6106	87	2010	W
3341_2007_S1339	2015	D	07.05.2015	S1339	13.7	21.5	Fam-89_2010_	S1339	1339	6107	89	2010	W
3341_2007_S1341	2015	D	07.05.2015	S1341	13.3	18.5	Fam-83_2010_	S1341	1341	6108	83	2010	W
3341_2007_S1342	2015	D	07.05.2015	S1342	13.7	20	Fam-64_2010_	S1342	1342	6109	64	2010	H
3341_2007_S1345	2015	D	07.05.2015	S1345	13.1	20.5	Fam-73_2010_	S1345	1345	6110	73	2010	F
3341_2007_S1347	2015	T	07.05.2015	S1347	13.7	20.5	Family-73_201	S1347	1347	6111	73	2010	F
3341_2007_S1350	2015	D	07.05.2015	S1350	13.5	18.5	Fam-89_2010_	S1350	1350	6112	89	2010	W
3341_2007_S1351	2015	D	07.05.2015	S1351	13.5	19.5	Fam-69_2010_	S1351	1351	6113	69	2010	F
3341_2007_S1355	2015	D	07.05.2015	S1355	14.2	22	Fam-73_2010_	S1355	1355	6114	73	2010	F
3341_2007_S1357	2015	D	07.05.2015	S1357	13.1	19	Fam-90_2010_	S1357	1357	6115	90	2010	W

3341_2007_S1359	2015	D	07.05.2015	S1359	13.5	19.5	Fam-68_2010_	S1359	1359	6116	68	2010 H
3342_2007_S1361	2015	D	07.05.2015	S1361	14.1	23.5	Fam-87_2010_	S1361	1361	6117	87	2010 W
3342_2007_S1362	2015	D	07.05.2015	S1362	15.1	28.5	Fam-90_2010_	S1362	1362	6118	90	2010 W
3342_2007_S1363	2015	D	07.05.2015	S1363	14.7	22	Fam-77_2010_	S1363	1363	6119	77	2010 F
3342_2007_S1364	2015	D	07.05.2015	S1364	13.7	20.5	Fam-63_2010_	S1364	1364	6120	63	2010 F
3342_2007_S1365	2015	D	07.05.2015	S1365	13.3	19	Fam-63_2010_	S1365	1365	6121	63	2010 F
3342_2007_S1366	2015	D	07.05.2015	S1366	13.2	18	Fam-77_2010_	S1366	1366	6122	77	2010 F
3342_2007_S1367	2015	D	07.05.2015	S1367	13.1	19	Fam-64_2010_	S1367	1367	6123	64	2010 H
3342_2007_S1368	2015	D	07.05.2015	S1368	13.4	20	Fam-79_2010_	S1368	1368	6124	79	2010 F
3342_2007_S1369	2015	D	07.05.2015	S1369	14.6	27.5	Fam-87_2010_	S1369	1369	6125	87	2010 W
3342_2007_S1370	2015	D	07.05.2015	S1370	14	23	Fam-86_2010_	S1370	1370	6126	86	2010 W
3342_2007_S1372	2015	D	07.05.2015	S1372	14.7	25.5	Fam-71_2010_	S1372	1372	6127	71	2010 F
3342_2007_S1373	2015	D	07.05.2015	S1373	13.8	22	Fam-75_2010_	S1373	1373	6128	75	2010 F
3342_2007_S1374	2015	D	07.05.2015	S1374	12.9	15	Fam-82_2010_	S1374	1374	6129	82	2010 W

3342_2007_S1375	2015	D	07.05.2015	S1375	14.5	25	Fam-83_2010_	S1375	1375	6130	83	2010 W
3342_2007_S1377	2015	D	07.05.2015	S1377	13.7	21.5	Fam-63_2010_	S1377	1377	6131	63	2010 F
3342_2007_S1378	2015	D	07.05.2015	S1378	13.1	18	Fam-83_2010_	S1378	1378	6132	83	2010 W
3342_2007_S1384	2015	D	07.05.2015	S1384	13.8	22	Fam-87_2010_	S1384	1384	6133	87	2010 W
3342_2007_S1385	2015	D	07.05.2015	S1385	13.9	21	Fam-45_2009_	S1385	1385	6134	45	2009 F
3342_2007_S1386	2015	D	07.05.2015	S1386	13.5	21	Fam-80_2010_	S1386	1386	6135	80	2010 H
3342_2007_S1387	2015	D	07.05.2015	S1387	12.5	17.5	Fam-90_2010_	S1387	1387	6136	90	2010 W
3342_2007_S1390	2015	D	07.05.2015	S1390	15.2	30.5	Fam-77_2010_	S1390	1390	6137	77	2010 F
3342_2007_S1392	2015	D	07.05.2015	S1392	12.7	17	Fam-89_2010_	S1392	1392	6138	89	2010 W
3342_2007_S1393	2015	T	07.05.2015	S1393	15.5	29	Family-77_201	S1393	1393	6139	77	2010 F
3342_2007_S1394	2015	D	07.05.2015	S1394	14.8	26	Fam-75_2010_	S1394	1394	6140	75	2010 F
3342_2007_S1395	2015	D	07.05.2015	S1395	13.2	18	Fam-60_2009_	S1395	1395	6141	60	2009 W
3342_2007_S1397	2015	D	07.05.2015	S1397	14.5	22.5	Fam-89_2010_	S1397	1397	6142	89	2010 W
3342_2007_S1399	2015	D	07.05.2015	S1399	13	17	Fam-84_2010_	S1399	1399	6143	84	2010 W

3342_2007_S140(	2015	D	07.05.2015	S1400	13.1	18	Fam-90_2010_	S1400	1400	6144	90	2010	W
3342_2007_S1401	2015	D	07.05.2015	S1401	13.6	22	Fam-66_2010_	S1401	1401	6145	66	2010	H
3342_2007_S1402	2015	D	07.05.2015	S1402	14.7	25	Fam-55_2009_	S1402	1402	6146	55	2009	W
3342_2007_S1403	2015	D	07.05.2015	S1403	14.4	24.5	Fam-74_2010_	S1403	1403	6147	74	2010	H
3342_2007_S1404	2015	D	07.05.2015	S1404	14.7	26.5	Fam-73_2010_	S1404	1404	6148	73	2010	F
3342_2007_S1405	2015	D	07.05.2015	S1405	13.2	19	Fam-87_2010_	S1405	1405	6149	87	2010	W
3342_2007_S1406	2015	D	07.05.2015	S1406	14.6	26	Fam-66_2010_	S1406	1406	6150	66	2010	H
3342_2007_S1407	2015	D	07.05.2015	S1407	12.7	17.5	Fam-54_2009_	S1407	1407	6151	54	2009	W
3342_2007_S1408	2015	D	07.05.2015	S1408	12.4	15.5	Fam-85_2010_	S1408	1408	6152	85	2010	W
3342_2007_S141(	2015	D	07.05.2015	S1410	13.7	20.5	Fam-74_2010_	S1410	1410	6153	74	2010	H
3342_2008_S1412	2015	D	07.05.2015	S1412	12.9	18	Fam-59_2009_	S1412	1412	6154	59	2009	W
3342_2008_S1413	2015	D	07.05.2015	S1413	13.6	20.5	Fam-78_2010_	S1413	1413	6155	78	2010	H
3342_2008_S1415	2015	D	07.05.2015	S1415	14.3	23	Fam-64_2010_	S1415	1415	6156	64	2010	H
3342_2008_S1416	2015	D	07.05.2015	S1416	12	14.5	Fam-87_2010_	S1416	1416	6157	87	2010	W

3342_2008_S1417	2015	D	07.05.2015	S1417	14.1	23	Fam-76_2010_	S1417	1417	6158	76	2010	H
3342_2008_S1420	2015	D	07.05.2015	S1420	14	21.5	Fam-41_2009_	S1420	1420	6159	41	2009	F
3342_2008_S1424	2015	D	07.05.2015	S1424	13.4	20.5	Fam-87_2010_	S1424	1424	6160	87	2010	W
3342_2008_S1425	2015	D	07.05.2015	S1425	13.8	21.5	Fam-90_2010_	S1425	1425	6161	90	2010	W
3342_2008_S1428	2015	D	07.05.2015	S1428	12.2	16	Fam-58_2009_	S1428	1428	6162	58	2009	W
3342_2008_S1433	2015	D	07.05.2015	S1433	14.2	23.5	Fam-42_2009_	S1433	1433	6163	42	2009	H
3342_2008_S1436	2015	D	07.05.2015	S1436	14.6	25.5	Fam-54_2009_	S1436	1436	6164	54	2009	W
3342_2008_S1437	2015	D	07.05.2015	S1437	13.9	22.5	Fam-79_2010_	S1437	1437	6165	79	2010	F
3343_2008_S1441	2015	D	07.05.2015	S1441	13	18.5	Fam-90_2010_	S1441	1441	6166	90	2010	W
3343_2008_S1442	2015	D	07.05.2015	S1442	14.4	24.5	Fam-89_2010_	S1442	1442	6167	89	2010	W
3343_2008_S1443	2015	D	07.05.2015	S1443	13.1	18	Fam-62_1_201	S1443	1443	6168	62	2010	H
3343_2008_S1444	2015	D	07.05.2015	S1444	12.4	15.5	Fam-84_2010_	S1444	1444	6169	84	2010	W
3343_2008_S1449	2015	D	07.05.2015	S1449	12.1	15	Fam-59_2009_	S1449	1449	6170	59	2009	W
3343_2008_S1450	2015	D	07.05.2015	S1450	13.4	18.5	Fam-83_2010_	S1450	1450	6171	83	2010	W

3343_2008_S1451	2015	D	07.05.2015	S1451	13.1	20	Fam-90_2010_	S1451	1451	6172	90	2010 W
3343_2008_S1452	2015	D	07.05.2015	S1452	13.3	21	Fam-79_2010_	S1452	1452	6173	79	2010 F
3343_2008_S1453	2015	D	07.05.2015	S1453	13.2	19	Fam-85_2010_	S1453	1453	6174	85	2010 W
3343_2008_S1455	2015	T	07.05.2015	S1455	13.2	16	Family-40_200	S1455	1455	6175	40	2009 H
3343_2008_S1457	2015	D	07.05.2015	S1457	13.7	20	Fam-79_2010_	S1457	1457	6176	79	2010 F
3343_2008_S1458	2015	D	07.05.2015	S1458	12.5	16	Fam-54_2009_	S1458	1458	6177	54	2009 W
3343_2008_S1459	2015	D	07.05.2015	S1459	12.7	18	Fam-78_2010_	S1459	1459	6178	78	2010 H
3343_2008_S1461	2015	D	07.05.2015	S1461	15	25.5	Fam-53_2009_	S1461	1461	6179	53	2009 W
3343_2008_S1462	2015	D	07.05.2015	S1462	11.7	12.5	Fam-71_2010_	S1462	1462	6180	71	2010 F
3343_2008_S1463	2015	D	07.05.2015	S1463	13.8	21	Fam-70_2010_	S1463	1463	6181	70	2010 H
3343_2008_S1464	2015	D	07.05.2015	S1464	14.9	26	Fam-89_2010_	S1464	1464	6182	89	2010 W
3343_2008_S1466	2015	D	07.05.2015	S1466	13.2	18	Fam-64_2010_	S1466	1466	6183	64	2010 H
3343_2008_S1468	2015	D	07.05.2015	S1468	15.4	29.5	Fam-71_2010_	S1468	1468	6184	71	2010 F
3343_2008_S1472	2015	D	07.05.2015	S1472	13.1	18	Fam-66_2010_	S1472	1472	6185	66	2010 H

3343_2008_S1473	2015	D	07.05.2015	S1473	14.2	24.5	Fam-78_2010_	S1473	1473	6186	78	2010	H
3343_2008_S1478	2015	D	07.05.2015	S1478	12.4	15.5	Fam-87_2010_	S1478	1478	6187	87	2010	W
3343_2008_S1484	2015	D	07.05.2015	S1484	13.9	22	Fam-66_2010_	S1484	1484	6188	66	2010	H
3343_2008_S1485	2015	D	07.05.2015	S1485	13.1	18	Fam-84_2010_	S1485	1485	6189	84	2010	W
3343_2008_S1486	2015	D	07.05.2015	S1486	13.2	19.5	Fam-85_2010_	S1486	1486	6190	85	2010	W
3343_2008_S1487	2015	D	07.05.2015	S1487	13.2	18	Fam-79_2010_	S1487	1487	6191	79	2010	F
3343_2008_S1490	2015	D	07.05.2015	S1490	12.8	17.5	Fam-87_2010_	S1490	1490	6192	87	2010	W
3343_2008_S1492	2015	D	07.05.2015	S1492	12.6	16.5	Fam-86_2010_	S1492	1492	6193	86	2010	W
3343_2008_S1494	2015	D	07.05.2015	S1494	13.3	18	Fam-84_2010_	S1494	1494	6194	84	2010	W
3343_2008_S1497	2015	D	07.05.2015	S1497	13.5	22.5	Fam-50_2009_	S1497	1497	6195	50	2009	H
3343_2008_S1499	2015	D	07.05.2015	S1499	13.2	18.5	Fam-80_2010_	S1499	1499	6196	80	2010	H
3343_2008_S1501	2015	D	07.05.2015	S1501	14.1	23	Fam-66_2010_	S1501	1501	6197	66	2010	H
3343_2009_S1505	2015	D	07.05.2015	S1505	13.9	23.5	Fam-80_2010_	S1505	1505	6198	80	2010	H
3343_2009_S1506	2015	D	11.05.2015	S1506	14.1	22	Fam-66_2010_	S1506	1506	6199	66	2010	H

3343_2009_S1507	2015	D	11.05.2015	S1507	14.2	23.5	Fam-83_2010_	S1507	1507	6200	83	2010	W
3343_2009_S1508	2015	D	11.05.2015	S1508	12.2	14.5	Fam-78_2010_	S1508	1508	6201	78	2010	H
3343_2009_S1510	2015	D	11.05.2015	S1510	15.2	27	Fam-68_2010_	S1510	1510	6202	68	2010	H
3343_2009_S1511	2015	D	11.05.2015	S1511	13.4	21	Fam-63_2010_	S1511	1511	6203	63	2010	F
3343_2009_S1514	2015	D	11.05.2015	S1514	14.2	21.5	Fam-84_2010_	S1514	1514	6204	84	2010	W
3343_2009_S1515	2015	D	11.05.2015	S1515	13.8	19.5	Fam-89_2010_	S1515	1515	6205	89	2010	W
3343_2009_S1517	2015	D	11.05.2015	S1517	14.8	24	Fam-84_2010_	S1517	1517	6206	84	2010	W
3343_2009_S1518	2015	D	11.05.2015	S1518	13.8	21	Fam-86_2010_	S1518	1518	6207	86	2010	W
3344_2009_S1521	2015	D	11.05.2015	S1521	13.6	22	Fam-62_1_201	S1521	1521	6208	62	2010	H
3344_2009_S1523	2015	D	11.05.2015	S1523	15.3	29.5	Fam-45_2009_	S1523	1523	6209	45	2009	F
3344_2009_S1525	2015	D	11.05.2015	S1525	13.5	20	Fam-83_2010_	S1525	1525	6210	83	2010	W
3344_2009_S1526	2015	D	11.05.2015	S1526	14.4	22	Fam-70_2010_	S1526	1526	6211	70	2010	H
3344_2009_S1527	2015	D	11.05.2015	S1527	13.6	20.5	Fam-85_2010_	S1527	1527	6212	85	2010	W
3344_2009_S1529	2015	D	11.05.2015	S1529	14	23	Fam-90_2010_	S1529	1529	6213	90	2010	W

3344_2009_S153(	2015	D	11.05.2015	S1530	13.5	21	Fam-66_2010_	S1530	1530	6214	66	2010	H
3344_2009_S1531	2015	T	11.05.2015	S1531	14.5	23	Family-34_200	S1531	1531	6215	34	2009	H
3344_2009_S1532	2015	D	11.05.2015	S1532	13.1	19	Fam-90_2010_	S1532	1532	6216	90	2010	W
3344_2009_S1533	2015	D	11.05.2015	S1533	13.5	20	Fam-85_2010_	S1533	1533	6217	85	2010	W
3344_2009_S1534	2015	D	11.05.2015	S1534	14.6	23.5	Fam-82_2010_	S1534	1534	6218	82	2010	W
3344_2009_S1537	2015	D	11.05.2015	S1537	14.1	22	Fam-85_2010_	S1537	1537	6219	85	2010	W
3344_2009_S1538	2015	D	11.05.2015	S1538	11.3	13	Fam-82_2010_	S1538	1538	6220	82	2010	W
3344_2009_S1539	2015	D	11.05.2015	S1539	12.5	15	Fam-54_2009_	S1539	1539	6221	54	2009	W
3344_2009_S1541	2015	D	11.05.2015	S1541	14.6	24.5	Fam-77_2010_	S1541	1541	6222	77	2010	F
3344_2009_S1542	2015	D	11.05.2015	S1542	15.9	33	Fam-65_2010_	S1542	1542	6223	65	2010	F
3344_2009_S1543	2015	D	11.05.2015	S1543	12.6	17	Fam-90_2010_	S1543	1543	6224	90	2010	W
3344_2009_S1544	2015	D	11.05.2015	S1544	13.9	21	Fam-76_2010_	S1544	1544	6225	76	2010	H
3344_2009_S1546	2015	D	11.05.2015	S1546	12.5	20	Fam-66_2010_	S1546	1546	6226	66	2010	H
3344_2009_S1547	2015	D	11.05.2015	S1547	14	21.5	Fam-64_2010_	S1547	1547	6227	64	2010	H

3344_2009_S1548	2015	D	11.05.2015	S1548	12.7	16.5	Fam-62_1_201	S1548	1548	6228	62	2010	H
3344_2009_S1549	2015	D	11.05.2015	S1549	15	25.5	Fam-52_2009_	S1549	1549	6229	52	2009	W
3344_2009_S1550	2015	D	11.05.2015	S1550	14.5	25.5	Fam-83_2010_	S1550	1550	6230	83	2010	W
3344_2009_S1551	2015	D	11.05.2015	S1551	14	22	Fam-78_2010_	S1551	1551	6231	78	2010	H
3344_2009_S1552	2015	D	11.05.2015	S1552	14.1	22	Fam-89_2010_	S1552	1552	6232	89	2010	W
3344_2009_S1553	2015	D	11.05.2015	S1553	12.2	18	Fam-68_2010_	S1553	1553	6233	68	2010	H
3344_2009_S1555	2015	D	11.05.2015	S1555	15	26.5	Fam-76_2010_	S1555	1555	6234	76	2010	H
3344_2009_S1557	2015	D	11.05.2015	S1557	13	18	Fam-78_2010_	S1557	1557	6235	78	2010	H
3344_2009_S1559	2015	D	11.05.2015	S1559	13.9	20.5	Fam-69_2010_	S1559	1559	6236	69	2010	F
3344_2009_S1561	2015	D	11.05.2015	S1561	13.7	20	Fam-72_2010_	S1561	1561	6237	72	2010	H
3344_2009_S1562	2015	D	11.05.2015	S1562	14.3	24.5	Fam-65_2010_	S1562	1562	6238	65	2010	F
3344_2009_S1563	2015	D	11.05.2015	S1563	13.5	20	Fam-62_1_201	S1563	1563	6239	62	2010	H
3344_2009_S1564	2015	D	11.05.2015	S1564	14.1	21.5	Fam-70_2010_	S1564	1564	6240	70	2010	H
3344_2009_S1565	2015	D	11.05.2015	S1565	14	21	Fam-83_2010_	S1565	1565	6241	83	2010	W

3344_2009_S1567	2015	D	11.05.2015	S1567	13.4	19	Fam-64_2010_	S1567	1567	6242	64	2010	H
3344_2009_S1568	2015	D	11.05.2015	S1568	13.7	22	Fam-73_2010_	S1568	1568	6243	73	2010	F
3344_2009_S1571	2015	D	11.05.2015	S1571	13.1	17.5	Fam-89_2010_	S1571	1571	6244	89	2010	W
3344_2009_S1575	2015	D	11.05.2015	S1575	15.5	30	Fam-77_2010_	S1575	1575	6245	77	2010	F
3344_2009_S1576	2015	D	11.05.2015	S1576	14.3	23	Fam-67_2010_	S1576	1576	6246	67	2010	F
3344_2009_S1577	2015	D	11.05.2015	S1577	10.7	14.5	Fam-59_2009_	S1577	1577	6247	59	2009	W
3344_2009_S1578	2015	D	11.05.2015	S1578	15.5	29	Fam-36_2009_	S1578	1578	6248	36	2009	H
3344_2009_S1579	2015	D	11.05.2015	S1579	13	18	Fam-90_2010_	S1579	1579	6249	90	2010	W
3344_2009_S1581	2015	D	11.05.2015	S1581	14	20	Fam-68_2010_	S1581	1581	6250	68	2010	H
3344_2009_S1582	2015	D	11.05.2015	S1582	13.3	18	Fam-74_2010_	S1582	1582	6251	74	2010	H
3344_2009_S1583	2015	D	11.05.2015	S1583	14.7	24	Fam-82_2010_	S1583	1583	6252	82	2010	W
3344_2009_S1585	2015	D	11.05.2015	S1585	12.7	16	Fam-87_2010_	S1585	1585	6253	87	2010	W
3344_2009_S1586	2015	D	11.05.2015	S1586	14.1	22	Fam-67_2010_	S1586	1586	6254	67	2010	F
3344_2009_S1587	2015	D	11.05.2015	S1587	14.8	26	Fam-67_2010_	S1587	1587	6255	67	2010	F

3344_2009_S1588	2015	D	11.05.2015	S1588	11.8	13	Fam-90_2010_	S1588	1588	6256	90	2010	W
3344_2009_S1589	2015	D	11.05.2015	S1589	14.2	22	Fam-76_2010_	S1589	1589	6257	76	2010	H
3344_2009_S1591	2015	D	11.05.2015	S1591	14	23.5	Fam-59_2009_	S1591	1591	6258	59	2009	W
3344_2009_S1592	2015	D	11.05.2015	S1592	12.7	15	Fam-82_2010_	S1592	1592	6259	82	2010	W
3344_2009_S1593	2015	D	11.05.2015	S1593	15.3	29	Fam-76_2010_	S1593	1593	6260	76	2010	H
3344_2009_S1596	2015	D	11.05.2015	S1596	14	23.5	Fam-62_1_201	S1596	1596	6261	62	2010	H
3344_2009_S1597	2015	D	11.05.2015	S1597	14.1	21	Fam-76_2010_	S1597	1597	6262	76	2010	H
3344_2009_S1598	2015	D	11.05.2015	S1598	14.5	25.5	Fam-47_2009_	S1598	1598	6263	47	2009	F
3344_2010_S1599	2015	D	11.05.2015	S1599	14.5	24.5	Fam-55_2009_	S1599	1599	6264	55	2009	W
3345_2010_S1601	2015	D	11.05.2015	S1601	13.4	17.5	Fam-82_2010_	S1601	1601	6265	82	2010	W
3345_2010_S1602	2015	D	11.05.2015	S1602	15.4	30	Fam-50_2009_	S1602	1602	6266	50	2009	H
3345_2010_S1603	2015	D	11.05.2015	S1603	13.5	21	Fam-85_2010_	S1603	1603	6267	85	2010	W
3345_2010_S1604	2015	D	11.05.2015	S1604	13.7	22	Fam-71_2010_	S1604	1604	6268	71	2010	F
3345_2010_S1605	2015	D	11.05.2015	S1605	14.3	24	Fam-83_2010_	S1605	1605	6269	83	2010	W

3345_2010_S1606	2015	D	11.05.2015	S1606	13	16.5	Fam-89_2010_S1606	1606	6270	89	2010	W
3345_2010_S1607	2015	D	11.05.2015	S1607	12.2	13	Fam-82_2010_S1607	1607	6271	82	2010	W
3345_2010_S1609	2015	D	11.05.2015	S1609	14	19	Fam-89_2010_S1609	1609	6272	89	2010	W
3345_2010_S1611	2015	D	11.05.2015	S1611	12	13.5	Fam-90_2010_S1611	1611	6273	90	2010	W
3345_2010_S1612	2015	D	11.05.2015	S1612	12.2	14.5	Fam-64_2010_S1612	1612	6274	64	2010	H
3345_2010_S1613	2015	D	11.05.2015	S1613	14.2	23.5	Fam-58_2009_S1613	1613	6275	58	2009	W
3345_2010_S1614	2015	D	11.05.2015	S1614	13.9	20.5	Fam-86_2010_S1614	1614	6276	86	2010	W
3345_2010_S1615	2015	D	11.05.2015	S1615	12.4	15	Fam-89_2010_S1615	1615	6277	89	2010	W
3345_2010_S1616	2015	D	11.05.2015	S1616	13.6	20.5	Fam-74_2010_S1616	1616	6278	74	2010	H
3345_2010_S1620	2015	D	11.05.2015	S1620	12.8	17.5	Fam-86_2010_S1620	1620	6279	86	2010	W
3345_2010_S1621	2015	D	11.05.2015	S1621	13.5	19	Fam-68_2010_S1621	1621	6280	68	2010	H
3345_2010_S1622	2015	D	11.05.2015	S1622	14.2	23	Fam-58_2009_S1622	1622	6281	58	2009	W
3345_2010_S1624	2015	D	11.05.2015	S1624	14.4	23	Fam-87_2010_S1624	1624	6282	87	2010	W
3345_2010_S1625	2015	D	11.05.2015	S1625	13.2	17	Fam-82_2010_S1625	1625	6283	82	2010	W

3345_2010_S1626	2015	D	11.05.2015	S1626	14	21.5	Fam-76_2010_	S1626	1626	6284	76	2010	H
3345_2010_S1627	2015	D	11.05.2015	S1627	13.6	20	Fam-76_2010_	S1627	1627	6285	76	2010	H
3345_2010_S1629	2015	D	11.05.2015	S1629	12.5	14.5	Fam-84_2010_	S1629	1629	6286	84	2010	W
3345_2010_S1630	2015	D	11.05.2015	S1630	14	21	Fam-84_2010_	S1630	1630	6287	84	2010	W
3345_2010_S1631	2015	D	11.05.2015	S1631	15.3	28	Fam-84_2010_	S1631	1631	6288	84	2010	W
3345_2010_S1632	2015	D	11.05.2015	S1632	14.5	24.5	Fam-76_2010_	S1632	1632	6289	76	2010	H
3345_2010_S1634	2015	D	11.05.2015	S1634	14.3	25	Fam-42_2009_	S1634	1634	6290	42	2009	H
3345_2010_S1635	2015	D	11.05.2015	S1635	13.1	19	Fam-85_2010_	S1635	1635	6291	85	2010	W
3345_2010_S1636	2015	D	11.05.2015	S1636	14	22	Fam-66_2010_	S1636	1636	6292	66	2010	H
3345_2010_S1637	2015	D	11.05.2015	S1637	14.7	27	Fam-78_2010_	S1637	1637	6293	78	2010	H
3345_2010_S1639	2015	D	11.05.2015	S1639	13.8	20.5	Fam-51_2009_	S1639	1639	6294	51	2009	W
3345_2010_S1641	2015	D	11.05.2015	S1641	13.5	20	Fam-78_2010_	S1641	1641	6295	78	2010	H
3345_2010_S1642	2015	D	11.05.2015	S1642	14.3	25	Fam-77_2010_	S1642	1642	6296	77	2010	F
3345_2010_S1643	2015	D	11.05.2015	S1643	13	17	Fam-90_2010_	S1643	1643	6297	90	2010	W

3345_2010_S1645	2015	D	11.05.2015	S1645	13.5	19	Fam-70_2010_	S1645	1645	6298	70	2010	H
3345_2010_S1646	2015	D	11.05.2015	S1646	15	29	Fam-39_2009_	S1646	1646	6299	39	2009	F
3345_2010_S1647	2015	D	11.05.2015	S1647	13.1	17.5	Fam-70_2010_	S1647	1647	6300	70	2010	H
3345_2010_S1648	2015	D	11.05.2015	S1648	13.8	21	Fam-85_2010_	S1648	1648	6301	85	2010	W
3345_2010_S1649	2015	D	11.05.2015	S1649	13.3	18	Fam-78_2010_	S1649	1649	6302	78	2010	H
3345_2010_S1651	2015	D	11.05.2015	S1651	15	26	Fam-83_2010_	S1651	1651	6303	83	2010	W
3345_2010_S1652	2015	D	11.05.2015	S1652	13.3	20	Fam-85_2010_	S1652	1652	6304	85	2010	W
3345_2010_S1653	2015	D	11.05.2015	S1653	14.3	23	Fam-83_2010_	S1653	1653	6305	83	2010	W
3345_2010_S1655	2015	D	11.05.2015	S1655	12.6	16	Fam-63_2010_	S1655	1655	6306	63	2010	F
3345_2010_S1657	2015	D	11.05.2015	S1657	14	22	Fam-83_2010_	S1657	1657	6307	83	2010	W
3345_2010_S1659	2015	D	11.05.2015	S1659	13.7	20	Fam-76_2010_	S1659	1659	6308	76	2010	H
3345_2010_S1664	2015	D	11.05.2015	S1664	14	22	Fam-62_1_201	S1664	1664	6309	62	2010	H
3345_2010_S1666	2015	D	11.05.2015	S1666	15.5	29.5	Fam-71_2010_	S1666	1666	6310	71	2010	F
3345_2010_S1667	2015	D	11.05.2015	S1667	14.3	23	Fam-83_2010_	S1667	1667	6311	83	2010	W

3345_2010_S167	2015	D	11.05.2015	S1670	12.7	15	Fam-81_2010_	S1670	1670	6312	81	2010	W
3345_2010_S167	2015	D	11.05.2015	S1671	14	20	Fam-84_2010_	S1671	1671	6313	84	2010	W
3345_2010_S167	2015	D	11.05.2015	S1672	14.9	25.5	Fam-32_2009_	S1672	1672	6314	32	2009	H
3345_2010_S167	2015	D	11.05.2015	S1673	13	16	Fam-82_2010_	S1673	1673	6315	82	2010	W
3345_2010_S167	2015	D	11.05.2015	S1675	15	29.5	Fam-32_2009_	S1675	1675	6316	32	2009	H
3345_2010_S167	2015	D	11.05.2015	S1677	14	20	Fam-89_2010_	S1677	1677	6317	89	2010	W
3345_2010_S167	2015	T	11.05.2015	S1678	13	16	Family-57_200	S1678	1678	6318	57	2009	W
3346_2010_S168	2015	D	11.05.2015	S1681	14.3	23	Fam-83_2010_	S1681	1681	6319	83	2010	W
3346_2010_S168	2015	D	11.05.2015	S1682	12	14.5	Fam-90_2010_	S1682	1682	6320	90	2010	W
3346_2010_S168	2015	D	11.05.2015	S1683	13.7	20	Fam-34_2009_	S1683	1683	6321	34	2009	H
3346_2010_S168	2015	D	11.05.2015	S1684	14.7	24.5	Fam-81_2010_	S1684	1684	6322	81	2010	W
3346_2010_S168	2015	D	11.05.2015	S1685	14.5	22.5	Fam-76_2010_	S1685	1685	6323	76	2010	H
3346_2010_S168	2015	D	11.05.2015	S1686	13.2	16.5	Fam-75_2010_	S1686	1686	6324	75	2010	F
3346_2010_S168	2015	D	11.05.2015	S1687	14	22	Fam-85_2010_	S1687	1687	6325	85	2010	W

3346_2010_S1689	2015	D	11.05.2015	S1689	16	30	Fam-77_2010_	S1689	1689	6326	77	2010	F
3346_2010_S1690	2015	D	11.05.2015	S1690	12.6	14.5	Fam-76_2010_	S1690	1690	6327	76	2010	H
3346_2010_S1691	2015	D	11.05.2015	S1691	14.2	22	Fam-76_2010_	S1691	1691	6328	76	2010	H
3346_2011_S1693	2015	D	11.05.2015	S1693	13.3	18	Fam-84_2010_	S1693	1693	6329	84	2010	W
3346_2011_S1695	2015	D	11.05.2015	S1695	13.4	19	Fam-79_2010_	S1695	1695	6330	79	2010	F
3346_2011_S1696	2015	D	11.05.2015	S1696	13.7	19.7	Fam-90_2010_	S1696	1696	6331	90	2010	W
3346_2011_S1697	2015	D	11.05.2015	S1697	12.2	14.5	Fam-54_2009_	S1697	1697	6332	54	2009	W
3346_2011_S1701	2015	D	11.05.2015	S1701	12.1	11.5	Fam-82_2010_	S1701	1701	6333	82	2010	W
3346_2011_S1703	2015	D	11.05.2015	S1703	14	20.5	Fam-63_2010_	S1703	1703	6334	63	2010	F
3346_2011_S1704	2015	D	11.05.2015	S1704	14.5	24	Fam-83_2010_	S1704	1704	6335	83	2010	W
3346_2011_S1707	2015	D	11.05.2015	S1707	13	17	Fam-89_2010_	S1707	1707	6336	89	2010	W
3346_2011_S1709	2015	D	11.05.2015	S1709	13	18.5	Fam-90_2010_	S1709	1709	6337	90	2010	W
3346_2011_S1710	2015	D	11.05.2015	S1710	14.4	24	Fam-63_2010_	S1710	1710	6338	63	2010	F
3346_2011_S1711	2015	D	11.05.2015	S1711	13.5	19	Fam-57_2009_	S1711	1711	6339	57	2009	W

3346_2011_S1712	2015	D	11.05.2015	S1712	12.5	17.5	Fam-81_2010_	S1712	1712	6340	81	2010	W
3346_2011_S1713	2015	D	11.05.2015	S1713	13.5	19	Fam-76_2010_	S1713	1713	6341	76	2010	H
3346_2011_S1715	2015	D	11.05.2015	S1715	12.7	16	Fam-62_1_201	S1715	1715	6342	62	2010	H
3346_2011_S1716	2015	D	11.05.2015	S1716	13.5	19	Fam-83_2010_	S1716	1716	6343	83	2010	W
3346_2011_S1719	2015	D	11.05.2015	S1719	12.7	16.5	Fam-72_2010_	S1719	1719	6344	72	2010	H
3346_2011_S1720	2015	D	11.05.2015	S1720	14	24.5	Fam-42_2009_	S1720	1720	6345	42	2009	H
3346_2011_S1722	2015	D	11.05.2015	S1722	13.5	16	Fam-84_2010_	S1722	1722	6346	84	2010	W
3346_2011_S1723	2015	D	11.05.2015	S1723	13.8	21.5	Fam-68_2010_	S1723	1723	6347	68	2010	H
3346_2011_S1726	2015	D	11.05.2015	S1726	16.2	34	Fam-40_2009_	S1726	1726	6348	40	2009	H
3346_2011_S1728	2015	D	11.05.2015	S1728	14.5	25	Fam-65_2010_	S1728	1728	6349	65	2010	F
3346_2011_S1729	2015	D	11.05.2015	S1729	13	17.5	Fam-74_2010_	S1729	1729	6350	74	2010	H
3346_2011_S1732	2015	D	11.05.2015	S1732	14.5	33	Fam-85_2010_	S1732	1732	6351	85	2010	W
3346_2011_S1733	2015	D	11.05.2015	S1733	12.8	15	Fam-82_2010_	S1733	1733	6352	82	2010	W
3346_2011_S1734	2015	D	11.05.2015	S1734	13.6	21	Fam-73_2010_	S1734	1734	6353	73	2010	F

3346_2011_S1736	2015	D	11.05.2015	S1736	15.3	29	Fam-67_2010_S1736	1736	6354	67	2010	F
3346_2011_S1737	2015	D	11.05.2015	S1737	12.6	15.5	Fam-85_2010_S1737	1737	6355	85	2010	W
3346_2011_S1738	2015	D	11.05.2015	S1738	14.2	21	Fam-71_2010_S1738	1738	6356	71	2010	F
3346_2011_S1739	2015	D	11.05.2015	S1739	14.1	20	Fam-32_2009_S1739	1739	6357	32	2009	H
3346_2011_S1740	2015	D	11.05.2015	S1740	13.7	20.5	Fam-85_2010_S1740	1740	6358	85	2010	W
3346_2011_S1741	2015	D	11.05.2015	S1741	13	18.5	Fam-87_2010_S1741	1741	6359	87	2010	W
3346_2011_S1742	2015	D	11.05.2015	S1742	14.2	23	Fam-66_2010_S1742	1742	6360	66	2010	H
3346_2011_S1743	2015	D	11.05.2015	S1743	12.6	15	Fam-84_2010_S1743	1743	6361	84	2010	W
3346_2011_S1744	2015	D	11.05.2015	S1744	13.3	18	Fam-68_2010_S1744	1744	6362	68	2010	H
3346_2011_S1745	2015	D	11.05.2015	S1745	13.5	18	Fam-55_2009_S1745	1745	6363	55	2009	W
3346_2011_S1746	2015	D	11.05.2015	S1746	13.5	20	Fam-42_2009_S1746	1746	6364	42	2009	H
3346_2011_S1747	2015	D	11.05.2015	S1747	14.2	24	Fam-67_2010_S1747	1747	6365	67	2010	F
3346_2011_S1748	2015	D	11.05.2015	S1748	15.3	26.5	Fam-83_2010_S1748	1748	6366	83	2010	W
3346_2011_S1749	2015	D	11.05.2015	S1749	13.3	19	Fam-83_2010_S1749	1749	6367	83	2010	W

3346_2011_S1750	2015	D	11.05.2015	S1750	13.2	18.5	Fam-70_2010_	S1750	1750	6368	70	2010	H
3346_2011_S1753	2015	D	11.05.2015	S1753	13.6	21	Fam-73_2010_	S1753	1753	6369	73	2010	F
3346_2011_S1754	2015	D	11.05.2015	S1754	14.5	25	Fam-66_2010_	S1754	1754	6370	66	2010	H
3346_2011_S1756	2015	D	11.05.2015	S1756	14.6	26	Fam-87_2010_	S1756	1756	6371	87	2010	W
3346_2011_S1758	2015	D	12.05.2015	S1758	14.2	21	Fam-64_2010_	S1758	1758	6372	64	2010	H
3346_2011_S1760	2015	D	12.05.2015	S1760	13.9	22	Fam-66_2010_	S1760	1760	6373	66	2010	H
3347_2011_S1761	2015	D	12.05.2015	S1761	14.1	22	Fam-78_2010_	S1761	1761	6374	78	2010	H
3347_2011_S1762	2015	D	12.05.2015	S1762	14.5	23.5	Fam-64_2010_	S1762	1762	6375	64	2010	H
3347_2011_S1764	2015	D	12.05.2015	S1764	15.1	27.5	Fam-54_2009_	S1764	1764	6376	54	2009	W
3347_2011_S1765	2015	D	12.05.2015	S1765	13.4	17.5	Fam-89_2010_	S1765	1765	6377	89	2010	W
3347_2011_S1766	2015	D	12.05.2015	S1766	14.2	22	Fam-66_2010_	S1766	1766	6378	66	2010	H
3347_2011_S1767	2015	D	12.05.2015	S1767	14.5	23	Fam-68_2010_	S1767	1767	6379	68	2010	H
3347_2011_S1768	2015	D	12.05.2015	S1768	13.8	20	Fam-67_2010_	S1768	1768	6380	67	2010	F
3347_2011_S1769	2015	D	12.05.2015	S1769	13.1	19	Fam-89_2010_	S1769	1769	6381	89	2010	W

3347_2011_S1771	2015	D	12.05.2015	S1771	14.2	24	Fam-78_2010_	S1771	1771	6382	78	2010	H
3347_2011_S1772	2015	D	12.05.2015	S1772	14	21.5	Fam-84_2010_	S1772	1772	6383	84	2010	W
3347_2011_S1773	2015	D	12.05.2015	S1773	12	13.5	Fam-82_2010_	S1773	1773	6384	82	2010	W
3347_2011_S1777	2015	D	12.05.2015	S1777	14.5	23	Fam-72_2010_	S1777	1777	6385	72	2010	H
3347_2011_S1778	2015	D	12.05.2015	S1778	14.1	23.5	Fam-54_2009_	S1778	1778	6386	54	2009	W
3347_2011_S1779	2015	D	12.05.2015	S1779	14.3	21	Fam-89_2010_	S1779	1779	6387	89	2010	W
3347_2011_S1780	2015	D	12.05.2015	S1780	15.1	28.5	Fam-33_2009_	S1780	1780	6388	33	2009	F
3347_2011_S1781	2015	D	12.05.2015	S1781	12.7	16	Fam-84_2010_	S1781	1781	6389	84	2010	W
3347_2011_S1782	2015	D	12.05.2015	S1782	14.1	22.5	Fam-67_2010_	S1782	1782	6390	67	2010	F
3347_2011_S1783	2015	D	12.05.2015	S1783	14.1	22	Fam-85_2010_	S1783	1783	6391	85	2010	W
3347_2011_S1784	2015	D	12.05.2015	S1784	15.4	30	Fam-32_2009_	S1784	1784	6392	32	2009	H
3347_2011_S1785	2015	D	12.05.2015	S1785	14.2	24	Fam-83_2010_	S1785	1785	6393	83	2010	W
3347_2012_S1787	2015	D	12.05.2015	S1787	13.4	19	Fam-89_2010_	S1787	1787	6394	89	2010	W
3347_2012_S1788	2015	D	12.05.2015	S1788	14.9	28	Fam-86_2010_	S1788	1788	6395	86	2010	W

3347_2012_S1789	2015	D	12.05.2015	S1789	13.5	20	Fam-57_2009_	S1789	1789	6396	57	2009	W
3347_2012_S1790	2015	D	12.05.2015	S1790	14.4	24	Fam-68_2010_	S1790	1790	6397	68	2010	H
3347_2012_S1791	2015	D	12.05.2015	S1791	14.5	21	Fam-76_2010_	S1791	1791	6398	76	2010	H
3347_2012_S1792	2015	D	12.05.2015	S1792	12.4	16.5	Fam-90_2010_	S1792	1792	6399	90	2010	W
3347_2012_S1793	2015	D	12.05.2015	S1793	13.6	19	Fam-76_2010_	S1793	1793	6400	76	2010	H
3347_2012_S1794	2015	D	12.05.2015	S1794	13.7	18	Fam-89_2010_	S1794	1794	6401	89	2010	W
3347_2012_S1795	2015	D	12.05.2015	S1795	14.2	25	Fam-87_2010_	S1795	1795	6402	87	2010	W
3347_2012_S1796	2015	D	12.05.2015	S1796	12.2	15.5	Fam-81_2010_	S1796	1796	6403	81	2010	W
3347_2012_S1797	2015	D	12.05.2015	S1797	12.6	16.5	Fam-85_2010_	S1797	1797	6404	85	2010	W
3347_2012_S1799	2015	D	12.05.2015	S1799	15	25.5	Fam-53_2009_	S1799	1799	6405	53	2009	W
3347_2012_S1800	2015	D	12.05.2015	S1800	15.2	29	Fam-83_2010_	S1800	1800	6406	83	2010	W
3347_2012_S1801	2015	D	12.05.2015	S1801	12.5	18	Fam-90_2010_	S1801	1801	6407	90	2010	W
3347_2012_S1802	2015	D	12.05.2015	S1802	14.5	26	Fam-59_2009_	S1802	1802	6408	59	2009	W
3347_2012_S1803	2015	D	12.05.2015	S1803	14	23.5	Fam-90_2010_	S1803	1803	6409	90	2010	W

3347_2012_S1805	2015	D	12.05.2015	S1805	15.2	28	Fam-64_2010_	S1805	1805	6410	64	2010	H
3347_2012_S1806	2015	D	12.05.2015	S1806	13.8	20.5	Fam-57_2009_	S1806	1806	6411	57	2009	W
3347_2012_S1807	2015	D	12.05.2015	S1807	13.1	18	Fam-79_2010_	S1807	1807	6412	79	2010	F
3347_2012_S1808	2015	D	12.05.2015	S1808	14.3	23	Fam-85_2010_	S1808	1808	6413	85	2010	W
3347_2012_S1810	2015	D	12.05.2015	S1810	13.5	22.5	Fam-62_1_201	S1810	1810	6414	62	2010	H
3347_2012_S1811	2015	D	12.05.2015	S1811	12.9	18	Fam-87_2010_	S1811	1811	6415	87	2010	W
3347_2012_S1818	2015	D	12.05.2015	S1818	14	22.5	Fam-62_1_201	S1818	1818	6416	62	2010	H
3347_2012_S1821	2015	D	12.05.2015	S1821	14.6	25	Fam-90_2010_	S1821	1821	6417	90	2010	W
3347_2012_S1823	2015	D	12.05.2015	S1823	15	25.5	Fam-53_2009_	S1823	1823	6418	53	2010	W
3347_2012_S1825	2015	D	12.05.2015	S1825	14	21.5	Fam-69_2010_	S1825	1825	6419	69	2010	F
3347_2012_S1826	2015	D	12.05.2015	S1826	14.6	24.5	Fam-83_2010_	S1826	1826	6420	83	2010	W
3347_2012_S1827	2015	D	12.05.2015	S1827	12.6	17	Fam-87_2010_	S1827	1827	6421	87	2010	W
3347_2012_S1828	2015	D	12.05.2015	S1828	13.8	19.5	Fam-62_1_201	S1828	1828	6422	62	2010	H
3347_2012_S1829	2015	D	12.05.2015	S1829	14.3	24.5	Fam-72_2010_	S1829	1829	6423	72	2010	H

3347_2012_S1831	2015	D	12.05.2015	S1831	14.6	26.5	Fam-76_2010_	S1831	1831	6424	76	2010	H
3347_2012_S1833	2015	D	12.05.2015	S1833	14.3	23	Fam-57_2009_	S1833	1833	6425	57	2009	W
3347_2012_S1835	2015	D	12.05.2015	S1835	12.9	18.5	Fam-87_2010_	S1835	1835	6426	87	2010	W
3347_2012_S1837	2015	D	12.05.2015	S1837	13.5	19.5	Fam-65_2010_	S1837	1837	6427	65	2010	F
3347_2012_S1838	2015	D	12.05.2015	S1838	13.3	17.5	Fam-89_2010_	S1838	1838	6428	89	2010	W
3347_2012_S1839	2015	D	12.05.2015	S1839	13.3	19.5	Fam-76_2010_	S1839	1839	6429	76	2010	H
3347_2012_S1840	2015	D	12.05.2015	S1840	14.1	22.5	Fam-74_2010_	S1840	1840	6430	74	2010	H
3348_2012_S1841	2015	D	12.05.2015	S1841	14.3	22.5	Fam-89_2010_	S1841	1841	6431	89	2010	W
3348_2012_S1842	2015	D	12.05.2015	S1842	14	23	Fam-55_2009_	S1842	1842	6432	55	2009	W
3348_2012_S1843	2015	D	12.05.2015	S1843	13.9	21.5	Fam-73_2010_	S1843	1843	6433	73	2010	F
3348_2012_S1844	2015	D	12.05.2015	S1844	13.2	19	Fam-68_2010_	S1844	1844	6434	68	2010	H
3348_2012_S1845	2015	D	12.05.2015	S1845	13.5	21	Fam-80_2010_	S1845	1845	6435	80	2010	H
3348_2012_S1846	2015	D	12.05.2015	S1846	13.1	19	Fam-76_2010_	S1846	1846	6436	76	2010	H
3348_2012_S1849	2015	D	12.05.2015	S1849	12.6	15.5	Fam-86_2010_	S1849	1849	6437	86	2010	W

3348_2012_S1850	2015	D	12.05.2015	S1850	12.5	16	Fam-67_2010_	S1850	1850	6438	67	2010	F
3348_2012_S1851	2015	D	12.05.2015	S1851	13.6	20.5	Fam-85_2010_	S1851	1851	6439	85	2010	W
3348_2012_S1852	2015	D	12.05.2015	S1852	13.1	18.5	Fam-87_2010_	S1852	1852	6440	87	2010	W
3348_2012_S1853	2015	D	12.05.2015	S1853	14.3	22.5	Fam-68_2010_	S1853	1853	6441	68	2010	H
3348_2012_S1854	2015	D	12.05.2015	S1854	14	20	Fam-68_2010_	S1854	1854	6442	68	2010	H
3348_2012_S1855	2015	D	12.05.2015	S1855	15.4	27.5	Fam-53_2009_	S1855	1855	6443	53	2009	W
3348_2012_S1856	2015	D	12.05.2015	S1856	15	26.5	Fam-57_2009_	S1856	1856	6444	57	2009	W
3348_2012_S1857	2015	D	12.05.2015	S1857	13.2	17.5	Fam-68_2010_	S1857	1857	6445	68	2010	H
3348_2012_S1860	2015	D	12.05.2015	S1860	14.5	28	Fam-60_2009_	S1860	1860	6446	60	2009	W
3348_2012_S1862	2015	D	12.05.2015	S1862	13.8	20.5	Fam-90_2010_	S1862	1862	6447	90	2010	W
3348_2012_S1863	2015	D	12.05.2015	S1863	13.5	20	Fam-66_2010_	S1863	1863	6448	66	2010	H
3348_2012_S1864	2015	D	12.05.2015	S1864	14.7	24	Fam-74_2010_	S1864	1864	6449	74	2010	H
3348_2012_S1865	2015	D	12.05.2015	S1865	12.4	15	Fam-54_2009_	S1865	1865	6450	54	2009	W
3348_2012_S1867	2015	D	12.05.2015	S1867	14.5	25	Fam-83_2010_	S1867	1867	6451	83	2010	W

3348_2012_S1872	2015	D	12.05.2015	S1872	14.5	22.5	Fam-89_2010_	S1872	1872	6452	89	2010	W
3348_2012_S1873	2015	D	12.05.2015	S1873	12.8	16.5	Fam-62_1_201	S1873	1873	6453	62	2010	H
3348_2012_S1874	2015	D	12.05.2015	S1874	15.8	29.5	Fam-50_2009_	S1874	1874	6454	50	2010	H
3348_2012_S1878	2015	D	12.05.2015	S1878	12.7	18	Fam-78_2010_	S1878	1878	6455	78	2010	H
3348_2012_S1879	2015	D	12.05.2015	S1879	12.2	15.5	Fam-59_2009_	S1879	1879	6456	59	2009	W
3348_2013_S1881	2015	D	12.05.2015	S1881	12.9	17	Fam-62_1_201	S1881	1881	6457	62	2010	H
3348_2013_S1882	2015	D	12.05.2015	S1882	14.9	25	Fam-83_2010_	S1882	1882	6458	83	2010	W
3348_2013_S1883	2015	D	12.05.2015	S1883	15.2	27.5	Fam-34_2009_	S1883	1883	6459	34	2010	H
3348_2013_S1884	2015	D	12.05.2015	S1884	13.3	20.5	Fam-73_2010_	S1884	1884	6460	73	2010	F
3348_2013_S1885	2015	D	12.05.2015	S1885	13.6	21.5	Fam-73_2010_	S1885	1885	6461	73	2010	F
3348_2013_S1886	2015	D	12.05.2015	S1886	15.9	31	Fam-82_2010_	S1886	1886	6462	82	2010	W
3348_2013_S1888	2015	D	12.05.2015	S1888	12.6	16.5	Fam-81_2010_	S1888	1888	6463	81	2010	W
3348_2013_S1890	2015	D	12.05.2015	S1890	14	23.5	Fam-62_1_201	S1890	1890	6464	62	2010	H
3348_2013_S1891	2015	D	12.05.2015	S1891	14.4	23.5	Fam-89_2010_	S1891	1891	6465	89	2010	W

3348_2013_S1892	2015	D	12.05.2015	S1892	13.7	21.5	Fam-76_2010_	S1892	1892	6466	76	2010 H
3348_2013_S1893	2015	D	12.05.2015	S1893	12.2	15.5	Fam-90_2010_	S1893	1893	6467	90	2010 W
3348_2013_S1900	2015	D	12.05.2015	S1900	13.5	22.5	Fam-83_2010_	S1900	1900	6468	83	2010 W
3348_2013_S1901	2015	D	12.05.2015	S1901	14	25	Fam-78_2010_	S1901	1901	6469	78	2010 H
3348_2013_S1904	2015	D	13.05.2015	S1904	16.7	36	Fam-04_2007_	S1904	1904	6470	4	2007 W
3348_2013_S1905	2015	D	13.05.2015	S1905	14.9	27.5	Fam-87_2010_	S1905	1905	6471	87	2010 W
3348_2013_S1906	2015	D	13.05.2015	S1906	12.2	15.5	Fam-87_2010_	S1906	1906	6472	87	2010 W
3348_2013_S1907	2015	D	13.05.2015	S1907	13.6	20.5	Fam-85_2010_	S1907	1907	6473	85	2010 W
3348_2013_S1908	2015	D	13.05.2015	S1908	15.1	29.5	Fam-76_2010_	S1908	1908	6474	76	2010 H
3348_2013_S1909	2015	D	13.05.2015	S1909	13.7	19	Fam-68_2010_	S1909	1909	6475	68	2010 H
3348_2013_S1910	2015	D	13.05.2015	S1910	14.4	22	Fam-83_2010_	S1910	1910	6476	83	2010 W
3348_2013_S1914	2015	D	13.05.2015	S1914	12.6	17	Fam-90_2010_	S1914	1914	6477	90	2010 W
3348_2013_S1916	2015	D	13.05.2015	S1916	13.7	19.5	Fam-62_1_201	S1916	1916	6478	62	2010 H
3348_2013_S1917	2015	D	13.05.2015	S1917	14	22	Fam-87_2010_	S1917	1917	6479	87	2010 W

3348_2013_S1918	2015	D	13.05.2015	S1918	11.9	14.5	Fam-68_2010_	S1918	1918	6480	68	2010	H
3348_2013_S1920	2015	D	13.05.2015	S1920	13.6	21.5	Fam-85_2010_	S1920	1920	6481	85	2010	W
3349_2013_S1921	2015	D	13.05.2015	S1921	14.7	27.5	Fam-67_2010_	S1921	1921	6482	67	2010	F
3349_2013_S1922	2015	D	13.05.2015	S1922	13.2	19.5	Fam-90_2010_	S1922	1922	6483	90	2010	W
3349_2013_S1926	2015	D	13.05.2015	S1926	13.9	21	Fam-86_2010_	S1926	1926	6484	86	2010	W
3349_2013_S1927	2015	D	13.05.2015	S1927	13.4	19.5	Fam-86_2010_	S1927	1927	6485	86	2010	W
3349_2013_S1931	2015	D	13.05.2015	S1931	13.7	22	Fam-84_2010_	S1931	1931	6486	84	2010	W
3349_2013_S1934	2015	D	13.05.2015	S1934	13.9	22	Fam-78_2010_	S1934	1934	6487	78	2010	H
3349_2013_S1935	2015	D	18.05.2015	S1935	14.9	25.5	Fam-32_2009_	S1935	1935	6488	32	2009	H
3349_2013_S1937	2015	D	18.05.2015	S1937	11.8	12.5	Fam-82_2010_	S1937	1937	6489	82	2010	W
3349_2013_S1938	2015	D	18.05.2015	S1938	13.4	20	Fam-78_2010_	S1938	1938	6490	78	2010	H
3349_2013_S1941	2015	D	18.05.2015	S1941	12.9	18	Fam-68_2010_	S1941	1941	6491	68	2010	H
3349_2013_S1942	2015	T	18.05.2015	S1942	14.5	24.5	Family-74_201	S1942	1942	6492	74	2010	H
3349_2013_S1945	2015	D	18.05.2015	S1945	13.5	20	Fam-90_2010_	S1945	1945	6493	90	2010	W

3349_2013_S1950	2015	D	26.05.2015	S1950	14.5	23	Fam-83_2010_	S1950	1950	6494	83	2010	W
3349_2013_S1951	2015	D	26.05.2015	S1951	13.5	20	Fam-59_2009_	S1951	1951	6495	59	2009	W
3349_2013_S1952	2015	D	26.05.2015	S1952	14.4	23.5	Fam-86_2010_	S1952	1952	6496	86	2010	W
3349_2013_S1956	2015	D	26.05.2015	S1956	13.5	20	Fam-90_2010_	S1956	1956	6497	90	2010	W
3349_2013_S1957	2015	D	26.05.2015	S1957	12.7	17.5	Fam-65_2010_	S1957	1957	6498	65	2010	F
3349_2013_S1961	2015	D	29.05.2015	S1961	15	26	Fam-83_2010_	S1961	1961	6499	83	2010	W
3349_2013_S1962	2015	D	29.05.2015	S1962	13.5	19	Fam-59_2009_	S1962	1962	6500	59	2009	W
3349_2013_S1964	2015	D	29.05.2015	S1964	14.9	27.5	Fam-73_2010_	S1964	1964	6501	73	2010	F
3349_2013_S1965	2015	D	29.05.2015	S1965	14.7	24.5	Fam-83_2010_	S1965	1965	6502	83	2010	W
3349_2013_S1967	2015	D	29.05.2015	S1967	12.6	17	Fam-59_2009_	S1967	1967	6503	59	2009	W
3349_2013_S1970	2015	D	29.05.2015	S1970	13.7	20	Fam-83_2010_	S1970	1970	6504	83	2010	W
3349_2013_S1972	2015	D	29.05.2015	S1972	13.1	19.5	Fam-90_2010_	S1972	1972	6505	90	2010	W
3349_2014_S1979	2015	D	29.05.2015	S1979	15.2	27.5	Fam-89_2010_	S1979	1979	6506	89	2010	W
3349_2014_S1981	2015	D	01.06.2015	S1981	14.3	23	Fam-83_2010_	S1981	1981	6507	83	2010	W

3349_2014_S1982	2015	D	01.06.2015	S1982	15.9	30.5	Fam-51_2009_	S1982	1982	6508	51	2009	W
3349_2014_S1984	2015	D	01.06.2015	S1984	14.6	24	Fam-84_2010_	S1984	1984	6509	84	2010	W
3349_2014_S1985	2015	D	01.06.2015	S1985	13.8	22.5	Fam-86_2010_	S1985	1985	6510	86	2010	W
3349_2014_S1986	2015	D	01.06.2015	S1986	15	27.5	Fam-83_2010_	S1986	1986	6511	83	2010	W
3349_2014_S1987	2015	D	01.06.2015	S1987	15.1	28.5	Fam-87_2010_	S1987	1987	6512	87	2010	W
3349_2014_S1988	2015	D	01.06.2015	S1988	16.5	35.5	Fam-89_2010_	S1988	1988	6513	89	2010	W
3349_2014_S1989	2015	D	01.06.2015	S1989	15.1	29.5	Fam-87_2010_	S1989	1989	6514	87	2010	W
3349_2014_S1992	2015	D	01.06.2015	S1992	15.4	29.5	Fam-87_2010_	S1992	1992	6515	87	2010	W
3349_2014_S1995	2015	D	01.06.2015	S1995	14.7	27	Fam-87_2010_	S1995	1995	6516	87	2010	W
3349_2014_S1997	2015	D	01.06.2015	S1997	14	21.5	Fam-83_2010_	S1997	1997	6517	83	2010	W
3349_2014_S1999	2015	D	01.06.2015	S1999	13.7	22	Fam-54_2009_	S1999	1999	6518	54	2009	W
3349_2014_S2000	2015	D	01.06.2015	S2000	13.8	20.5	Fam-89_2010_	S2000	2000	6519	89	2010	W
3350_2014_S2002	2015	D	01.06.2015	S2002	14.7	27	Fam-70_2010_	S2002	2002	6520	70	2010	H
3350_2014_S2003	2015	D	01.06.2015	S2003	14.5	25.5	Fam-66_2010_	S2003	2003	6521	66	2010	H

3350_2014_S2004	2015	D	01.06.2015	S2004	13.8	25.5	Fam-32_2009_S2004	2004	6522	32	2009	H
3350_2014_S2006	2015	D	01.06.2015	S2006	14.7	27	Fam-87_2010_S2006	2006	6523	87	2010	W
3350_2014_S2007	2015	D	01.06.2015	S2007	13.6	19	Fam-82_2010_S2007	2007	6524	82	2010	W
3350_2014_S2008	2015	D	01.06.2015	S2008	14.7	26.5	Fam-90_2010_S2008	2008	6525	90	2010	W
3350_2014_S2009	2015	D	01.06.2015	S2009	13.8	22.5	Fam-90_2010_S2009	2009	6526	90	2010	W
3350_2014_S2010	2015	D	01.06.2015	S2010	14.1	25.5	Fam-90_2010_S2010	2010	6527	90	2010	W
3350_2014_S2012	2015	D	01.06.2015	S2012	13.9	21.5	Fam-86_2010_S2012	2012	6528	86	2010	W
3350_2014_S2013	2015	D	01.06.2015	S2013	14.4	23.5	Fam-87_2010_S2013	2013	6529	87	2010	W
3350_2014_S2014	2015	D	01.06.2015	S2014	16.4	34.5	Fam-89_2010_S2014	2014	6530	89	2010	W
3350_2014_S2015	2015	D	01.06.2015	S2015	13.9	22	Fam-86_2010_S2015	2015	6531	86	2010	W
3350_2014_S2017	2015	D	01.06.2015	S2017	14.1	23.5	Fam-68_2010_S2017	2017	6532	68	2010	H
3350_2014_S2018	2015	D	01.06.2015	S2018	13.6	19.5	Fam-81_2010_S2018	2018	6533	81	2010	W
3350_2014_S2020	2015	D	01.06.2015	S2020	14.1	25	Fam-89_2010_S2020	2020	6534	89	2010	W
3350_2014_S2021	2015	D	01.06.2015	S2021	13.3	18.5	Fam-89_2010_S2021	2021	6535	89	2010	W

3350_2014_S2022	2015	D	01.06.2015	S2022	13	16.5	Fam-59_2009_S2022	2022	6536	59	2009 W
3350_2014_S2023	2015	D	01.06.2015	S2023	14	21.5	Fam-83_2010_S2023	2023	6537	83	2010 W
3350_2014_S2026	2015	D	01.06.2015	S2026	13.8	25	Fam-90_2010_S2026	2026	6538	90	2010 W
3350_2014_S2030	2015	D	01.06.2015	S2030	14.5	24	Fam-70_2010_S2030	2030	6539	70	2010 H
3350_2014_S2031	2015	D	01.06.2015	S2031	13.3	20.5	Fam-90_2010_S2031	2031	6540	90	2010 W
3350_2014_S2032	2015	D	01.06.2015	S2032	14.4	25.5	Fam-73_2010_S2032	2032	6541	73	2010 F
3350_2014_S2033	2015	D	01.06.2015	S2033	15.7	30.5	Fam-74_2010_S2033	2033	6542	74	2010 H
3350_2014_S2034	2015	D	01.06.2015	S2034	14.1	24	Fam-87_2010_S2034	2034	6543	87	2010 W
3350_2014_S2035	2015	D	01.06.2015	S2035	14.9	27.5	Fam-76_2010_S2035	2035	6544	76	2010 H
3350_2014_S2036	2015	D	01.06.2015	S2036	13	18	Fam-84_2010_S2036	2036	6545	84	2010 W
3350_2014_S2037	2015	D	01.06.2015	S2037	14.3	24.5	Fam-84_2010_S2037	2037	6546	84	2010 W
3350_2014_S2038	2015	D	01.06.2015	S2038	13.6	22.5	Fam-90_2010_S2038	2038	6547	90	2010 W
3350_2014_S2039	2015	D	01.06.2015	S2039	13.3	21	Fam-59_2009_S2039	2039	6548	59	2009 W
3350_2014_S2042	2015	D	01.06.2015	S2042	14.8	28	Fam-86_2010_S2042	2042	6549	86	2010 W

3350_2014_S2043	2015	D	01.06.2015	S2043	15.1	29	Fam-74_2010_	S2043	2043	6550	74	2010	H
3350_2014_S2044	2015	D	01.06.2015	S2044	13.9	20.5	Fam-70_2010_	S2044	2044	6551	70	2010	H
3350_2014_S2045	2015	D	01.06.2015	S2045	14.4	24.5	Fam-66_2010_	S2045	2045	6552	66	2010	H
3350_2014_S2046	2015	D	01.06.2015	S2046	14	25	Fam-83_2010_	S2046	2046	6553	83	2010	W
3350_2014_S2047	2015	D	01.06.2015	S2047	15.7	33.5	Fam-87_2010_	S2047	2047	6554	87	2010	W
3350_2014_S2048	2015	D	01.06.2015	S2048	14.6	27.5	Fam-73_2010_	S2048	2048	6555	73	2010	F
3350_2014_S2049	2015	D	01.06.2015	S2049	13.3	20	Fam-59_2009_	S2049	2049	6556	59	2009	W
3350_2014_S2050	2015	D	01.06.2015	S2050	14.6	24.5	Fam-82_2010_	S2050	2050	6557	82	2010	W
3350_2014_S2053	2015	D	01.06.2015	S2053	14.2	25	Fam-78_2010_	S2053	2053	6558	78	2010	H
3350_2014_S2056	2015	D	01.06.2015	S2056	14.9	27.5	Fam-89_2010_	S2056	2056	6559	89	2010	W
3350_2014_S2057	2015	D	01.06.2015	S2057	12.8	18	Fam-90_2010_	S2057	2057	6560	90	2010	W
3350_2014_S2058	2015	D	01.06.2015	S2058	14.3	23	Fam-89_2010_	S2058	2058	6561	89	2010	W
3350_2014_S2060	2015	D	01.06.2015	S2060	15.8	33.5	Fam-40_2009_	S2060	2060	6562	40	2009	H
3350_2014_S2062	2015	D	02.06.2015	S2062	13.7	20	Fam-74_2010_	S2062	2062	6563	74	2010	H

3350_2014_S2064	2015	D	02.06.2015	S2064	15.1	30	Fam-54_2009_	S2064	2064	6564	54	2009	W
3350_2014_S2066	2015	D	02.06.2015	S2066	13.6	20	Fam-90_2010_	S2066	2066	6565	90	2010	W
3350_2016_S2069	2015	D	02.06.2015	S2069	14	21.5	Fam-89_2010_	S2069	2069	6566	89	2010	W
3350_2016_S2070	2015	D	02.06.2015	S2070	14.2	23.5	Fam-87_2010_	S2070	2070	6567	87	2010	W
3350_2016_S2072	2015	D	02.06.2015	S2072	13.4	19.5	Fam-81_2010_	S2072	2072	6568	81	2010	W
3350_2016_S2073	2015	D	02.06.2015	S2073	15.4	32	Fam-87_2010_	S2073	2073	6569	87	2010	W
3350_2016_S2075	2015	D	02.06.2015	S2075	13.4	21	Fam-62_1_201	S2075	2075	6570	62	2010	H
3350_2016_S2077	2015	D	02.06.2015	S2077	14.1	23	Fam-78_2010_	S2077	2077	6571	78	2010	H
3350_2016_S2078	2015	D	02.06.2015	S2078	14.7	24.5	Fam-89_2010_	S2078	2078	6572	89	2010	W
3350_2016_S2079	2015	D	02.06.2015	S2079	13.6	21.5	Fam-87_2010_	S2079	2079	6573	87	2010	W
3351_2016_S2081	2015	D	02.06.2015	S2081	13.7	23	Fam-90_2010_	S2081	2081	6574	90	2010	W
3351_2016_S2082	2015	D	02.06.2015	S2082	14.4	25	Fam-55_2009_	S2082	2082	6575	55	2009	W
3351_2016_S2084	2015	D	02.06.2015	S2084	13.6	20	Fam-84_2010_	S2084	2084	6576	84	2010	W
3351_2016_S2086	2015	D	02.06.2015	S2086	15.1	28.5	Fam-76_2010_	S2086	2086	6577	76	2010	H

3351_2016_S2087	2015	D	02.06.2015	S2087	13.2	20	Fam-90_2010_	S2087	2087	6578	90	2010	W
3351_2016_S2088	2015	D	02.06.2015	S2088	13.3	17	Fam-84_2010_	S2088	2088	6579	84	2010	W
3351_2016_S2090	2015	D	02.06.2015	S2090	13.1	19	Fam-86_2010_	S2090	2090	6580	86	2010	W
3351_2016_S2093	2015	D	02.06.2015	S2093	14.6	22.5	Fam-89_2010_	S2093	2093	6581	89	2010	W
3351_2016_S2095	2015	D	02.06.2015	S2095	15.4	32	Fam-58_2009_	S2095	2095	6582	58	2009	W
3351_2016_S2096	2015	D	02.06.2015	S2096	14.5	25	Fam-87_2010_	S2096	2096	6583	87	2010	W
3351_2016_S2097	2015	D	02.06.2015	S2097	14	23.5	Fam-90_2010_	S2097	2097	6584	90	2010	W
3351_2016_S2099	2015	D	02.06.2015	S2099	15	27.5	Fam-84_2010_	S2099	2099	6585	84	2010	W
3351_2016_S2101	2015	D	02.06.2015	S2101	15.5	30.5	Fam-90_2010_	S2101	2101	6586	90	2010	W
3351_2016_S2103	2015	D	02.06.2015	S2103	12	13	Fam-59_2009_	S2103	2103	6587	59	2009	W
3351_2016_S2105	2015	D	02.06.2015	S2105	13.6	21	Fam-90_2010_	S2105	2105	6588	90	2010	W
3351_2016_S2107	2015	D	02.06.2015	S2107	15	27.5	Fam-85_2010_	S2107	2107	6589	85	2010	W
3351_2016_S2110	2015	D	02.06.2015	S2110	15.2	26.5	Fam-74_2010_	S2110	2110	6590	74	2010	H
3351_2016_S2111	2015	D	02.06.2015	S2111	14.2	22.5	Fam-53_2009_	S2111	2111	6591	53	2009	W

3351_2016_S2112	2015	D	02.06.2015	S2112	13	18.5	Fam-87_2010_ S2112	2112	6592	87	2010	W
3351_2016_S2113	2015	D	02.06.2015	S2113	13.6	19	Fam-84_2010_ S2113	2113	6593	84	2010	W
3351_2016_S2114	2015	D	02.06.2015	S2114	13.7	21.5	Fam-90_2010_ S2114	2114	6594	90	2010	W
3351_2016_S2116	2015	D	02.06.2015	S2116	15.4	32	Fam-87_2010_ S2116	2116	6595	87	2010	W
3351_2016_S2117	2015	D	02.06.2015	S2117	13.5	23	Fam-87_2010_ S2117	2117	6596	87	2010	W
3351_2016_S2118	2015	D	02.06.2015	S2118	13.5	19.5	Fam-68_2010_ S2118	2118	6597	68	2010	H
3351_2016_S2119	2015	D	02.06.2015	S2119	14.6	26	Fam-83_2010_ S2119	2119	6598	83	2010	W
3351_2016_S2120	2015	D	02.06.2015	S2120	12.7	18	Fam-76_2010_ S2120	2120	6599	76	2010	H
3351_2016_S2121	2015	D	02.06.2015	S2121	14	21	Fam-83_2010_ S2121	2121	6600	83	2010	W
3351_2016_S2122	2015	D	02.06.2015	S2122	13.9	22.5	Fam-87_2010_ S2122	2122	6601	87	2010	W
3351_2016_S2123	2015	D	02.06.2015	S2123	14.1	23.5	Fam-86_2010_ S2123	2123	6602	86	2010	W
3351_2016_S2133	2015	D	05.06.2015	S2133	13.6	21.5	Fam-70_2010_ S2133	2133	6603	70	2010	H
3351_2016_S2134	2015	D	05.06.2015	S2134	14.7	27.5	Fam-80_2010_ S2134	2134	6604	80	2010	H
3351_2016_S2135	2015	D	05.06.2015	S2135	14.4	25	Fam-87_2010_ S2135	2135	6605	87	2010	W

3351_2016_S2136	2015	D	05.06.2015	S2136	15	26	Fam-68_2010_ S2136	2136	6606	68	2010	H
3351_2016_S2138	2015	D	05.06.2015	S2138	13.1	18	Fam-86_2010_ S2138	2138	6607	86	2010	W
3351_2016_S2141	2015	D	05.06.2015	S2141	14.3	24	Fam-59_2009_ S2141	2141	6608	59	2009	W
3351_2016_S2142	2015	D	05.06.2015	S2142	14.6	25.5	Fam-54_2009_ S2142	2142	6609	54	2009	W
3351_2016_S2144	2015	D	05.06.2015	S2144	15.5	28	Fam-70_2010_ S2144	2144	6610	70	2010	H
3351_2016_S2145	2015	D	05.06.2015	S2145	14.2	23	Fam-87_2010_ S2145	2145	6611	87	2010	W
3351_2016_S2146	2015	D	05.06.2015	S2146	13.8	20.5	Fam-83_2010_ S2146	2146	6612	83	2010	W
3351_2016_S2147	2015	D	05.06.2015	S2147	14	23	Fam-68_2010_ S2147	2147	6613	68	2010	H
3351_2016_S2148	2015	D	05.06.2015	S2148	15	26	Fam-86_2010_ S2148	2148	6614	86	2010	W
3351_2016_S2149	2015	D	05.06.2015	S2149	14.3	21.5	Fam-84_2010_ S2149	2149	6615	84	2010	W
3351_2016_S2150	2015	D	05.06.2015	S2150	14.5	27	Fam-42_2009_ S2150	2150	6616	42	2009	H
3351_2016_S2151	2015	D	05.06.2015	S2151	15	25	Fam-82_2010_ S2151	2151	6617	82	2010	W
3351_2016_S2152	2015	D	05.06.2015	S2152	13.9	22	Fam-84_2010_ S2152	2152	6618	84	2010	W
3351_2016_S2153	2015	D	05.06.2015	S2153	13.7	20	Fam-74_2010_ S2153	2153	6619	74	2010	H

3351_2016_S2158	2015	D	05.06.2015	S2158	14	23	Fam-90_2010_	S2158	2158	6620	90	2010	W
3351_2016_S2159	2015	D	05.06.2015	S2159	14	22.5	Fam-87_2010_	S2159	2159	6621	87	2010	W
3352_2017_S2164	2015	D	05.06.2015	S2164	14.7	25.5	Fam-66_2010_	S2164	2164	6622	66	2010	H
3352_2017_S2165	2015	D	05.06.2015	S2165	14	29.5	Fam-42_2009_	S2165	2165	6623	42	2009	H
3352_2017_S2166	2015	D	05.06.2015	S2166	13.9	21	Fam-86_2010_	S2166	2166	6624	86	2010	W
3352_2017_S2167	2015	D	05.06.2015	S2167	14.5	24	Fam-76_2010_	S2167	2167	6625	76	2010	H
3352_2017_S2168	2015	D	05.06.2015	S2168	14.3	23.5	Fam-86_2010_	S2168	2168	6626	86	2010	W
3352_2017_S2169	2015	D	05.06.2015	S2169	15.7	33	Fam-60_2009_	S2169	2169	6627	60	2009	W
3352_2017_S2174	2015	D	05.06.2015	S2174	15	26	Fam-84_2010_	S2174	2174	6628	84	2010	W
3352_2017_S2180	2015	D	05.06.2015	S2180	12.7	16.5	Fam-86_2010_	S2180	2180	6629	86	2010	W
3352_2017_S2182	2015	D	05.06.2015	S2182	13.6	21.5	Fam-90_2010_	S2182	2182	6630	90	2010	W
3352_2017_S2185	2015	D	05.06.2015	S2185	14	22.5	Fam-86_2010_	S2185	2185	6631	86	2010	W
3352_2017_S2189	2015	D	05.06.2015	S2189	13.5	20.5	Fam-86_2010_	S2189	2189	6632	86	2010	W
3352_2017_S2191	2015	D	08.06.2015	S2191	14	21	Fam-82_2010_	S2191	2191	6633	82	2010	W

3352_2017_S2192	2015	D	08.06.2015	S2192	15	26	Fam-57_2009_S2192	2192	6634	57	2009 W
3352_2017_S2198	2015	D	08.06.2015	S2198	14.5	28	Fam-87_2010_S2198	2198	6635	87	2010 W
3352_2017_S2200	2015	D	08.06.2015	S2200	14.9	26.5	Fam-82_2010_S2200	2200	6636	82	2010 W
3352_2017_S2202	2015	D	08.06.2015	S2202	14.5	26	Fam-87_2010_S2202	2202	6637	87	2010 W
3352_2017_S2204	2015	D	08.06.2015	S2204	13.9	24.5	Fam-87_2010_S2204	2204	6638	87	2010 W
3352_2017_S2206	2015	D	08.06.2015	S2206	14.5	23	Fam-76_2010_S2206	2206	6639	76	2010 H
3352_2017_S2207	2015	D	08.06.2015	S2207	14.2	22	Fam-82_2010_S2207	2207	6640	82	2010 W
3352_2017_S2208	2015	D	08.06.2015	S2208	15.5	32	Fam-32_2009_S2208	2208	6641	32	2009 H
3352_2017_S2210	2015	D	08.06.2015	S2210	14.3	23.5	Fam-68_2010_S2210	2210	6642	68	2010 H
3352_2017_S2211	2015	D	08.06.2015	S2211	14	23	Fam-86_2010_S2211	2211	6643	86	2010 W
3352_2017_S2213	2015	D	08.06.2015	S2213	14.2	24.5	Fam-90_2010_S2213	2213	6644	90	2010 W
3352_2017_S2214	2015	D	08.06.2015	S2214	15.7	29.5	Fam-84_2010_S2214	2214	6645	84	2010 W
3352_2017_S2217	2015	D	08.06.2015	S2217	13.5	21.5	Fam-90_2010_S2217	2217	6646	90	2010 W
3352_2017_S2218	2015	D	08.06.2015	S2218	15.9	32	Fam-40_2009_S2218	2218	6647	40	2009 H

3352_2017_S2219	2015	D	08.06.2015	S2219	14	23	Fam-54_2009_	S2219	2219	6648	54	2009	W
3352_2017_S2220	2015	D	08.06.2015	S2220	13.6	21	Fam-59_2009_	S2220	2220	6649	59	2009	W
3352_2017_S2222	2015	D	08.06.2015	S2222	15.6	32.5	Fam-68_2010_	S2222	2222	6650	68	2010	H
3352_2017_S2223	2015	D	08.06.2015	S2223	14.5	24.5	Fam-89_2010_	S2223	2223	6651	89	2010	W
3352_2017_S2226	2015	D	08.06.2015	S2226	14.5	25	Fam-90_2010_	S2226	2226	6652	90	2010	W
3352_2017_S2227	2015	D	08.06.2015	S2227	15	30	Fam-60_2009_	S2227	2227	6653	60	2009	W
3352_2017_S2228	2015	D	08.06.2015	S2228	14.3	23	Fam-68_2010_	S2228	2228	6654	68	2010	H
3352_2017_S2229	2015	D	08.06.2015	S2229	13.6	21	Fam-86_2010_	S2229	2229	6655	86	2010	W
3352_2017_S2233	2015	D	08.06.2015	S2233	15	30	Fam-55_2009_	S2233	2233	6656	55	2009	W
3352_2017_S2235	2015	D	08.06.2015	S2235	14.3	22	Fam-52_2009_	S2235	2235	6657	52	2009	W
3352_2017_S2236	2015	D	08.06.2015	S2236	16.2	35	Fam-60_2009_	S2236	2236	6658	60	2009	W
3352_2017_S2237	2015	D	08.06.2015	S2237	15.9	32	Fam-60_2009_	S2237	2237	6659	60	2009	W
3352_2017_S2239	2015	D	08.06.2015	S2239	16.4	38	Fam-52_2009_	S2239	2239	6660	52	2009	W
3353_2017_S2241	2015	D	08.06.2015	S2241	14.1	24	Fam-86_2010_	S2241	2241	6661	86	2010	W

3353_2017_S2246	2015	D	08.06.2015	S2246	14.2	24	Fam-70_2010_	S2246	2246	6662	70	2010	H
3353_2017_S2247	2015	D	08.06.2015	S2247	14.1	22.5	Fam-81_2010_	S2247	2247	6663	81	2010	W
3353_2017_S2248	2015	D	08.06.2015	S2248	14.3	24.5	Fam-54_2009_	S2248	2248	6664	54	2009	W
3353_2017_S2253	2015	D	08.06.2015	S2253	14.5	25.5	Fam-83_2010_	S2253	2253	6665	83	2010	W
3353_2017_S2254	2015	D	08.06.2015	S2254	14.5	22	Fam-84_2010_	S2254	2254	6666	84	2010	W
3353_2017_S2256	2015	D	08.06.2015	S2256	14	21	Fam-84_2010_	S2256	2256	6667	84	2010	W
3353_2018_S2257	2015	D	08.06.2015	S2257	14.9	27.5	Fam-54_2009_	S2257	2257	6668	54	2009	W
3353_2018_S2261	2015	D	08.06.2015	S2261	13.8	20	Fam-82_2010_	S2261	2261	6669	82	2010	W
3353_2018_S2265	2015	D	08.06.2015	S2265	15.2	31	Fam-42_2009_	S2265	2265	6670	42	2009	H
3353_2018_S2270	2015	D	08.06.2015	S2270	15.5	29.5	Fam-86_2010_	S2270	2270	6671	86	2010	W
3353_2018_S2272	2015	D	08.06.2015	S2272	14.4	24	Fam-87_2010_	S2272	2272	6672	87	2010	W
3353_2018_S2273	2015	D	08.06.2015	S2273	14.8	26	Fam-87_2010_	S2273	2273	6673	87	2010	W
3353_2018_S2275	2015	D	08.06.2015	S2275	13.5	19	Fam-84_2010_	S2275	2275	6674	84	2010	W
3353_2018_S2277	2015	D	08.06.2015	S2277	15	27	Fam-40_2009_	S2277	2277	6675	40	2009	H

3353_2018_S2279	2015	D	08.06.2015	S2279	13.4	20	Fam-90_2010_	S2279	2279	6676	90	2010 W
3353_2018_S2288	2015	D	08.06.2015	S2288	14	22	Fam-55_2009_	S2288	2288	6677	55	2009 W
3353_2018_S2291	2015	D	08.06.2015	S2291	15	28	Fam-90_2010_	S2291	2291	6678	90	2010 W
3353_2018_S2294	2015	D	08.06.2015	S2294	14.3	21.5	Fam-84_2010_	S2294	2294	6679	84	2010 W
3353_2018_S2295	2015	D	08.06.2015	S2295	12.8	21.5	Fam-83_2010_	S2295	2295	6680	83	2010 W
3353_2018_S2298	2015	D	08.06.2015	S2298	14.7	23.5	Fam-51_2009_	S2298	2298	6681	51	2009 W
3353_2018_S2299	2015	D	08.06.2015	S2299	14.6	27.5	Fam-42_2009_	S2299	2299	6682	42	2009 H
3353_2018_S2301	2015	D	08.06.2015	S2301	14.5	25	Fam-62_1_201	S2301	2301	6683	62	2010 H
3353_2018_S2302	2015	D	08.06.2015	S2302	14.3	22.5	Fam-76_2010_	S2302	2302	6684	76	2010 H
3353_2018_S2303	2015	D	08.06.2015	S2303	14.1	21.5	Fam-84_2010_	S2303	2303	6685	84	2010 W
3353_2018_S2304	2015	D	08.06.2015	S2304	13.8	21	Fam-54_2009_	S2304	2304	6686	54	2009 W
3353_2018_S2308	2015	D	08.06.2015	S2308	13.2	19	Fam-59_2009_	S2308	2308	6687	59	2009 W
3353_2018_S2310	2015	D	08.06.2015	S2310	14.6	25.5	Fam-86_2010_	S2310	2310	6688	86	2010 W
3353_2018_S2313	2015	D	08.06.2015	S2313	14.5	26.5	Fam-86_2010_	S2313	2313	6689	86	2010 W

3353_2018_S2316	2015	D	08.06.2015	S2316	13	19	Fam-90_2010_	S2316	2316	6690	90	2010	W
3353_2018_S2318	2015	D	11.06.2015	S2318	15.1	29	Fam-55_2009_	S2318	2318	6691	55	2009	W
